# Toward defining the Anthropocene onset using a rapid increase in anthropogenic fingerprints in global geological archives

**DOI:** 10.1073/pnas.2313098121

**Published:** 2024-09-23

**Authors:** Michinobu Kuwae, Yusuke Yokoyama, Stephen Tims, Michaela Froehlich, L. Keith Fifield, Takahiro Aze, Narumi Tsugeki, Hideyuki Doi, Yoshiki Saito

**Affiliations:** ^a^Center for Marine Environmental Studies, Ehime University, Matsuyama 790-8577, Japan; ^b^Atmosphere and Ocean Research Institute, The University of Tokyo, Kashiwa 277-8564, Japan; ^c^Research School of Physics, The Australian National University, Canberra, ACT 2601, Australia; ^d^The Faculty of Law, Matsuyama University, Matsuyama 790-8578, Japan; ^e^Graduate School of Informatics, Kyoto University, Kyoto 606-8501, Japan; ^f^Estuary Research Center, Shimane University, Matsue 690-8504, Japan; ^g^Geological Survey of Japan, The National Institute of Advanced Industrial Science and Technology, Tsukuba 305-8567, Japan

**Keywords:** anthropocene, great acceleration, anthropogenic fingerprints, technosphere, plutonium

## Abstract

One of the remaining issues regarding the Anthropocene is the lack of stratigraphic evidence indicating when the cumulative human pressure from the early Holocene began to fundamentally change the Earth system. Herein, we compile anthropogenic fingerprints from various high-precision-dated proxy records for 137 global sites to determine the age of the unprecedented surge in these records over the last 7700 y. The cumulative number of fingerprints revealed an unprecedented surge in diverse anthropogenic fingerprints starting in 1952 ± 3 CE, corresponding to the onset of the Great Acceleration. Notably, the period from 1953 to 1958 CE saw a nearly simultaneous surge in fingerprints across all regions, including Antarctica, the Arctic, East Asia, Europe, North America, and Oceania. This synchronous upsurge reflects the moment when human impacts led to rapid transformations in various natural processes and cycles, with humans becoming a geological force capable of inscribing abundant and diverse anthropogenic fingerprints in global strata. Following this global fingerprint explosion, profound planetary-scale changes, including deviations from the established natural climatic conditions, begin. This unprecedented surge in anthropogenic signals worldwide suggests that human influences started to match many natural forces controlling the processes and cycles and overwhelm some of the functioning of the Earth system around 1952.

## Significance

In the context of the Anthropocene, identifying the precise moment at which the consequences of fundamental human-induced changes in the Earth system first appear on the planet remains a long-standing challenge. This is due to the lack of a clear stratigraphic marker for the start date, as human impacts on Earth’s environments are significantly time-transgressive and spatiotemporally variable. Our study revealed that the number of anthropogenic fingerprints in global strata began to increase abruptly from 1952 ± 3 CE. This signal may reflect the onset of key human-induced changes in the Earth system, providing unambiguous stratigraphic evidence. This unprecedented synchronous increase has potential significance for defining the start of the Anthropocene in the future.

Human activities have now reached a scale where their impacts on the Earth system can be observed, and they have driven significant changes in much of its state and functioning (1). Paul J. Crutzen and Eugene F. Stoermer referred to this current human-dominated geological epoch as the Anthropocene, defining it as an epoch in which the Earth system deviates from its natural behavior for many millennia to come, such as global warming due to increased anthropogenic carbon dioxide concentrations (2). In 2007, Steffen et al. defined the Anthropocene as the current epoch in which humans and their societies have become geophysical forces (3). Discussions by the Anthropocene Working Group (AWG) within the International Union of Geological Sciences (IUGS) have concluded that it is both reasonable and conservative to consider the Anthropocene as an Epoch/Series on the Geological Time Scale, starting with the “Great Acceleration” (4–6). The Great Acceleration, which began around 1950 CE, is characterized by a range of global and near-synchronous signals, including abrupt changes in socioeconomic factors, biophysical processes, and the resulting environmental and climatic changes (3, 7).

Evidence shown in the AWG's formal proposal for the Anthropocene underscores that the mid-20th century is the only period with an extensive array of globally isochronous geological markers necessary to permit definition as a boundary of the geochronologic unit (8). However, determining the exact point at which humans first began to induce fundamental changes in the Earth system remains a long-standing challenge in the context of the Anthropocene.

One reason for this difficulty is that this point in time predates the mid-20th century by a significant margin (9). Starting approximately 12,000 y ago, with the development of agriculture, humans began to use approximately 75% of the total terrestrial area, according to anthropogenic land use estimates from the History Database of the Global Environment (10). Several studies also provide evidence for early human-induced transformations of the Earth, including the development of agricultural societies around 8,000 y ago (11), the advent of irrigated rice cultivation between 6,500 and 5,000 y ago (12), the restoration of forests following European arrival in the “New World” from 1,492 to approximately 1,800 (13), and the Industrial Revolution (2). These activities resulted in changes to the climate system, including atmospheric CO_2_ and CH_4_ concentrations. Consequently, the exact timing of when human activity began to fundamentally change the Earth System remains debatable. Additionally, these human–environment transformations began in different locations at different times and spread geographically at different rates (14). Thus, establishing an isochronous horizon that reflects a single point in time for the chronostratigraphic boundary is problematic (14). Given the time-transgressive nature of human modifications to Earth systems, it could be argued that the Anthropocene might be better considered as an "event" without a clear isochronous boundary (9, 14). These factors underscore the difficulty in pinpointing a unique moment when human activities began to induce profound changes in the Earth system and partly contributed to the rejection of the AWG’s formal proposal for the Anthropocene as an epoch/series by IUGS-ICS (9).

Notably, the AWG’s formal proposal indicates that the synchronous upturn in plutonium in global strata around 1952 CE can serve as a primary marker of the isochronous horizon (7, 8). This date could therefore be a candidate for when cumulative human pressure from the early Holocene began to induce profound changes in the Earth system. However, the dispersion of radionuclides across the Earth does not necessarily constitute a force inducing irreversible changes throughout the Earth system. Instead, climate changes and biological effects, such as mass extinctions and the global translocation of nonnative species permanently changing the biosphere (8), more closely capture the essence of the Anthropocene as proposed by Crutzen and Steffen. These factors also have been important criteria for chronostratigraphic classification (8, 15).

Nevertheless, it is apparent that there is an increasing deviation in the global mean surface temperatures from natural variability (16), beginning approximately 20 y after the plutonium signal. Furthermore, while mass extinctions have not yet occurred (17), extinction rates began to rise above the previous background level between the 19th and 20th centuries (18). However, the differences in the timings of these profound, human-induced transformations of the Earth system inevitably result in the absence of a clear geological marker that can signify a single date when human activities became a planetary force. Consequently, the lack of a stratigraphic marker indicating when human activities started to profoundly change the Earth system is one of the remaining issues regarding the Anthropocene.

Here, we propose using the rapid increase in the number of anthropogenic fingerprints in strata as a stratigraphic indicator identifying such a unique point in time. These fingerprints are detected by several criteria, including i) the initial emergence of anthropogenic materials (e.g., artificial radionuclides, persistent organic materials, and microplastics) and biological species not previously observed in specific strata, ii) their subsequent rapid increases, iii) the identification of unprecedentedly high or low values in biological, geochemical, and biogeochemical indices, exceeding the historical ranges of natural variability prior to the Industrial Revolution, iv) an inflection point to a higher rate of change if the record shows a long-term trend, and v) alterations in microfossil assemblages, among others.

In the varved marine sediments of Beppu Bay, Japan, designated by the AWG as the standard auxiliary boundary stratotype section for the Anthropocene (19), a hockey stick-like surge in cumulative anthropogenic fingerprint counts is recognized in 1953 CE, capturing clear stratigraphic evidence for the potential onset of the Anthropocene (20). Although this merely indicates a local chronostratigraphic boundary, it reflects the moment when human activities became so significant that cumulative pressures began to rapidly and fundamentally transform various physical, chemical, and biological processes and cycles. Observation of such a surge in cumulative anthropogenic fingerprints in the global strata could allow the inflection point to serve as a promising marker for when human pressures became a dominant force in the Earth system. The explosive spread of anthropogenic fingerprints can be regarded as resulting from the establishment of interconnected technological systems that support modern civilization; the rapid expansion of these technological practices altered various natural processes and cycles. Hence, the global surge in anthropogenic fingerprints can also serve as a marker for the point in time of the ascendancy of a new subsystem, the “Technosphere” (21), or a human society/material culture-included “Anthroposphere” (22) of the Earth system comprising the atmosphere, hydrosphere, lithosphere, cryosphere, and biosphere. Such global fingerprint stratigraphy-based analysis will provide an opportunity to reconsider the initiation of the Anthropocene as suggested by Crutzen (2) and Steffen (3) and will be useful for future considerations of the Anthropocene onset in the geological community.

Here, we integrate open-access datasets (Dataset S01) of high-precision dated, high-resolution records (most <4 y in age error and time resolution) from varved lacustrine and marine sediments, corals, ice, and tree rings from candidate sites (Crawford Lake, Sihailongwan Maar Lake, Searsville Lake, East Gotland Basin, Beppu Bay, Flinders Reef, West Flower Garden Reef, the Palmer Ice Sheet) for the Global Boundary Stratotype Section and Point (GSSP) site of the AWG-proposed Anthropocene (7) and from other locations. These data were then applied to determine the period during which the number of anthropogenic fingerprints rapidly increased in regional and global strata, facilitating the identification of a point at which human activities became a planetary force in the Earth system. Furthermore, we examined the temporal relationship between the determined date of the surge point, obtained using our proposed approach, with the Pu signal—a global correlative marker for the Anthropocene onset proposed by the AWG (8).

## Results and Discussion

### Anthropogenic Fingerprints in the Global Strata.

**Fig. 1.**
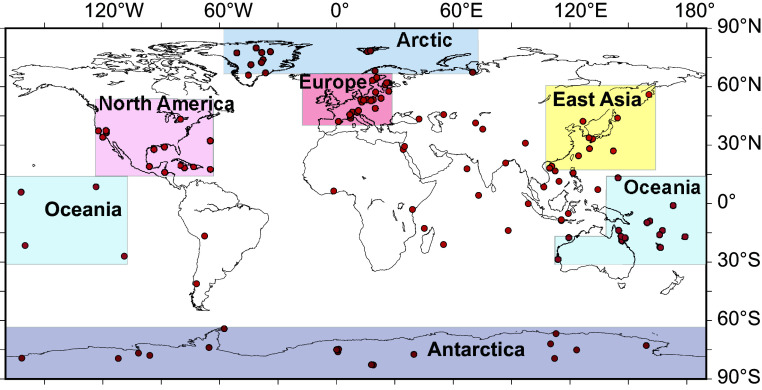
Map of geological archive locations used for the detection of anthropogenic fingerprints. Red circles denote 137 locations of geological archives, including varved marine and lake sediments, coral skeletons, ice cores, and tree ring samples.

**Fig. 2.**
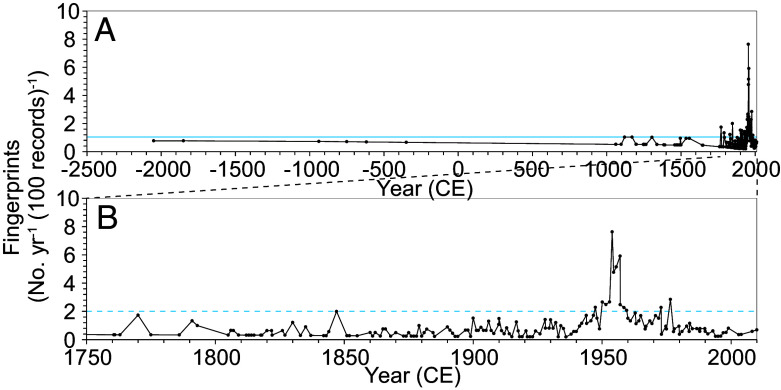
Temporal changes in anthropogenic fingerprints per year per 100 records detected from global geological archives for the last 7,700 y. (*A*) Full-range record from 2050 BCE (not shown before 2500 BCE due to no fingerprints). (*B*) Enlarged record from 1750 to 2010 CE. The light blue line and dashed line denote the maximum values before 1750 CE and between 1750 and the 1930s. Temporal changes in anthropogenic fingerprints per year are shown in *SI Appendix*, Fig. S7. The original proxy records for detecting fingerprints are provided in Dataset S02.

To determine when human activities became a planetary force in the Earth system, we analyzed the number of anthropogenic fingerprints per year per 100 records (numbers year^−1^ (100 proxy records)^−1^) identified in 388 proxy records recovered from 137 sites (Fig. 1 and Dataset S01 for the list of detected anthropogenic fingerprints). The record showed 0 y^−1^ (100 records)^−1^ from 5700 BCE to 2050 BCE and then 1.0 y^−1^ (100 records)^−1^ until 1750 CE (Fig. 2 and Dataset S05). It reached 1.7 y^−1^ (100 records)^−1^ in 1770, the first year to exceed the 1.0 y^−1^ (100 records)^−1^ value for most of the middle and late Holocene. In 1947, the rate exceeded 2 y^−1^ (100 records)^−1^ for the first time, and between 1952 and 1957, it reached 4.8 to 7.6 y^−1^ (100 records)^−1^. Afterward, it declined to less than 2.9 y^−1^ (100 records)^−1^. The decline since 1957 does not necessarily reflect a decrease in human influences because we did not count more than the third or fourth abrupt change in a single record, changes associated with reductions in pollution, or signals showing a continuous upward or downward trend, which could indicate intensifying anthropogenic pressure.

Evaluating the interperiod differences in the number of fingerprints per year is challenging due to the larger gaps in age between the data points for earlier time periods, causing a bias in data density. However, one of the main objectives of this study is to determine when the number of anthropogenic fingerprints in global strata exhibits an unprecedented rapid increase, potentially indicating the point at which human activities began to cause significant changes in the Earth system. Thus, assessing the changes in the number of fingerprints over time, or the density of fingerprints in strata per year, could provide crucial insights regarding the initiation date.

**Fig. 3.**
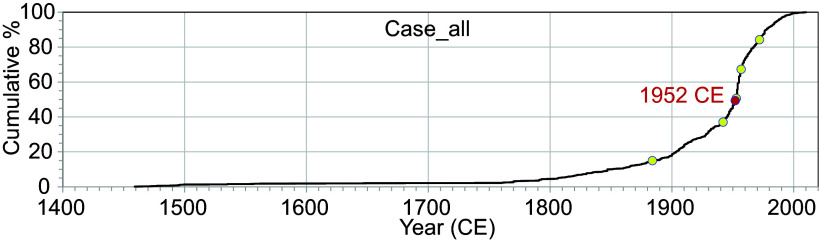
Cumulative percentages of anthropogenic fingerprints for all the data. The red and yellow circles denote the change-point and break-points derived from the change-point and break-point analysis based on one point and five points, respectively, for the last 550 y. The data used for change-point and break-point analysis are provided in Dataset S04. The model settings and parameters are listed in (*SI Appendix*, Tables S9 and S10). Case_all denotes a case ID referred to in *SI Appendix*, Table S1.

The differences in the number of anthropogenic fingerprints per year are more evident when viewed as the cumulative fingerprint percentage (out of a total of 748 fingerprints for the entire period), represented as a slope (Fig. 3). The change points or inflection points objectively capture the moments when the fingerprint number density rapidly changes. We performed both a single-point change point analysis and a five-point linear bifurcation breakpoint analysis based on the likelihood of the cumulative percentages of fingerprints since 1400 CE (the last point of sparse data in the time series).

The single-point change-point analysis showed a change point in 1952 (Fig. 3 and *SI Appendix*, Table S1), while the five-point analysis identified breakpoints (hereinafter referred to as change points) in 1884, 1942, 1953, 1957, and 1972 (Fig. 3 and *SI Appendix*, Table S2). Since the rapid increase in the number of anthropogenic fingerprints is recognized as the maximum slope in an interval between two consecutive change points, the slope of the cumulative percentage between each change point was determined. The results showed that the maximum slope occurred between 1953 and 1957 (*SI Appendix*, Table S2). Notably, the abrupt increases in the 1870s, 1930s, and 1950s did not correspond to increases in the number of paleorecords (*SI Appendix*, Fig. S1). This discrepancy indicates that the sudden increases are solely attributable to the rise in fingerprints, not to data source biases due to changes in the number of paleorecords.

The age estimation of change points is influenced by the selection of data sources. For instance, the age of the sharp increase in anthropogenic fingerprints may vary depending on whether fingerprints with synchronous signals between regions are included, whether the sample size is adjusted if the number of data differs between regions or sites, and even the criteria chosen for detecting anthropogenic fingerprints. To ensure robust results, further change point analyses were conducted in 13 cases with different data sources in which data biases were reduced. Among these cases, a single change-point analysis using data excluding radionuclide signals (Case_all_exc rad, 13% of the total, *SI Appendix*, Fig. S2*B*) which tends to show global synchroneity identified the year 1950. For data excluding only plutonium (^239^Pu, ^239+240^Pu, and ^240^Pu/^239^Pu, Case_exc Pu, *SI Appendix*, Fig. S2*C*), a potential global marker of isochronous stratigraphic correlation, the year 1952 was identified. The year 1953 emerged when excluding the data for Beppu Bay, Japan (Case_all_exc BB, 15% of the total, *SI Appendix*, Fig. S2*D*), and further exclusion of both Beppu Bay data and radionuclides (Case_all_exc BB rad, 26% of the total, *SI Appendix*, Fig. S2*E*) indicated 1946. Five-point change-point analyses in these four cases showed distinct change points in 1865 to 1884, 1909 to 1944, 1944 to 1954, 1956 to 1957, and 1970 to 1975 (*SI Appendix*, Table S2). The maximum slopes were observed between the third (1944 to 1954) and fourth (1956 to 1957) change points in these cases (*SI Appendix*, Table S2). The third change point in each case coincided within two years of the age of the change point for the single change point of each case (*SI Appendix*, Table S1 and S2).

The 13 cases also include scenarios where the impact of selecting criteria for detecting anthropogenic fingerprints on estimating their ages and subsequent change points was minimized. Most fingerprints (470 of 748) were derived from records with an increasing or decreasing trend in the proxy value, which are the ages of the first occurrence of proxy values above or below the previous background levels, along with minor and major changes following the first occurrence detected (Dataset S02). For this fingerprint detection criterion, the choice of an interval determining the given background level will change the detection level of an abnormal value and the resultant age of the detected fingerprint. To eliminate the influence of the choice of the upper age of the interval on the results, change-point analysis was also conducted using the dataset produced with the upper limit set to 1900 or earlier (i.e., excluding anthropogenic fingerprints detected as upper ages 1920 and 1950; Case_exc pre-1950/1920 range, N = 690, *SI Appendix*, Fig. S2*F*) and the dataset using the upper limit set to 1800 or earlier (i.e., excluding anthropogenic fingerprints detected using the upper limit 1900, 1920, and 1950; Case_exc pre-1900/1950/1920 range, N = 600, *SI Appendix*, Fig. S2*G*). As a result, the change points showed 1951 to 1953 for the single-point analysis and the years 1870 to 1881, 1941 to 1942, 1953, 1957, and 1972 to 1973 for the five-point analysis, with a maximum slope in 1953 to 1957 (*SI Appendix*, Table S2).

We also examined change points in a subset with reduced uncertainty in the age of anthropogenic fingerprints, resulting from differences in record resolution. Although most anthropogenic fingerprints have a temporal resolution of 4 y or less in the records around the identified age, the actual signal may be located more than 4 y earlier or later if the records have a resolution greater than 4 y. Here, we analyzed a subset of the data limited to anthropogenic fingerprints with a resolution of less than 4 y (note that this was not the case before 1800) (*SI Appendix*, Fig. S2*H*). The results indicated 1952 for the single-point analysis and 1874, 1935, 1952, 1956, and 1972 for the five-point analysis.

The 13 cases also included scenarios where data from relatively data-rich Anthropocene GSSP candidate sites. These sites (Antarctic Peninsula, Beppu Bay, Crawford Lake, Flinders Reef, Flower Garden Banks, Gotland Basin, Searsville Reservoir, Sihailongwan Maar Lake), which focus on the Great Acceleration, may inevitably concentrate anthropogenic fingerprints around 1950. Cases where data from these GSSP candidate sites were excluded and the number of data points from each region was adjusted to approximately 50 (Cases_1–6, *SI Appendix*, Fig. S3) showed change points in 1874 ± 13, 1935 ± 8, 1952 ± 2, 1956 ± 1, and 1972 ± 1 (1 SD).

Overall, these 13 cases showed change points in 1876 ± 10, 1935 ± 10, 1952 ± 3, 1957 ± 1, and 1971 ± 2 (1 SD), as outlined in *SI Appendix*, Table S2. The maximum slope occurred between 1952 ± 3 and 1957 ± 1, spanning from 1948 to 1957. These results, which account for biases in data representation and differences in criteria for detecting anthropogenic fingerprints, were consistent with the analysis performed on all data (third change point in the five-point analysis: 1953; interval of change points including maximum slope: 1953 to 1957).

### Semisynchronized Upsurges of Fingerprints Across Regions.

**Fig. 4.**
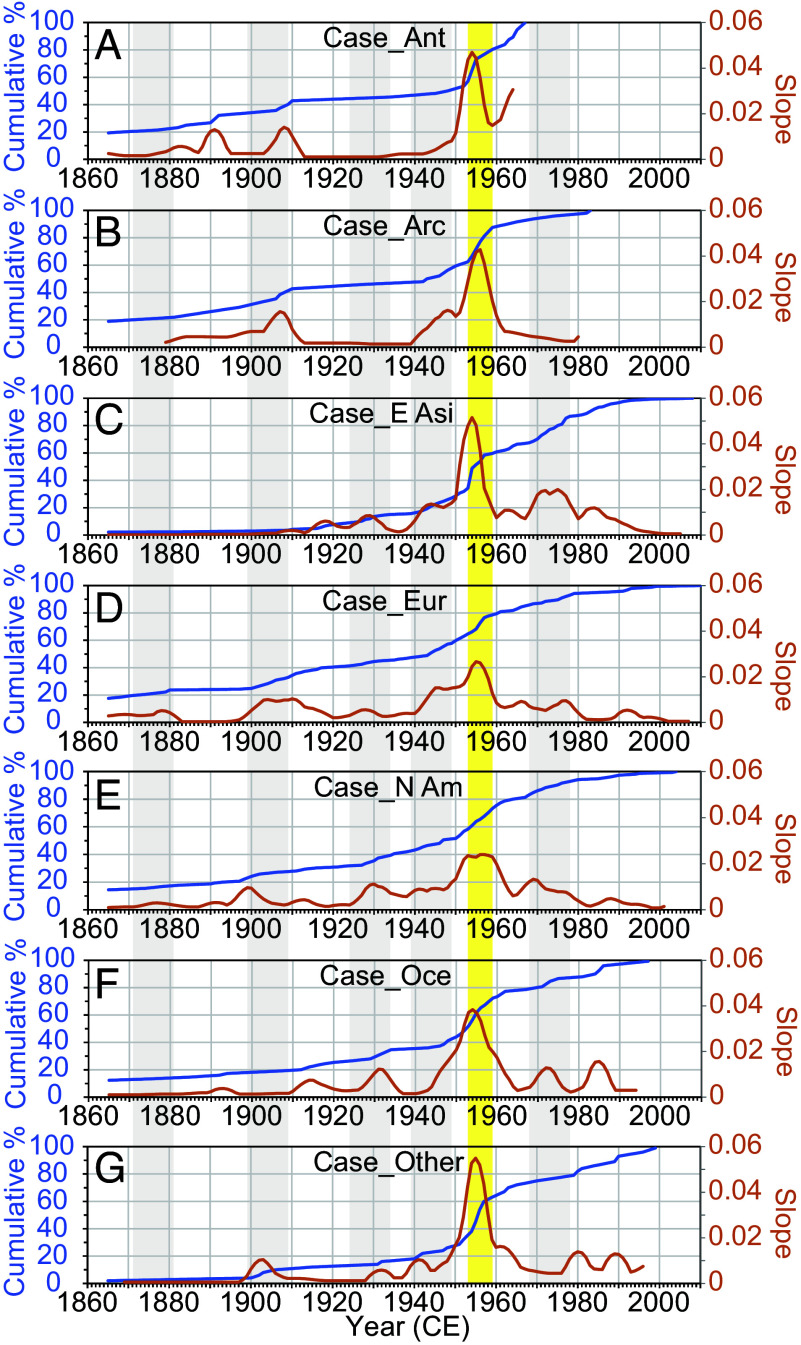
Cumulative percentage of anthropogenic fingerprints (blue) and slope (red) for each region. (*A*) Case_Ant, (*B*) Case_Arc, (*C*) Case_E Asi, (*D*) Case_Eur, (*E*) Case N Am, (*F*) Case_Oce, and (*G*) Case_Other denote subsets of fingerprint data from Antarctica, Arctic, East Asia, Europe, North America, Oceania, and the other regions. The yellow shading denotes nearly simultaneous unprecedented increases in fingerprints with maximum slopes in 1953 to 1958 CE. The gray shading denotes a decade with a potential simultaneous increase in fingerprints between regions.

A key objective of this study was to identify when anthropogenic fingerprints began to exhibit unprecedented synchronous upsurges globally, signaling the point at which human activities became a planetary force in the Earth system. To assess this synchroneity, we calculated the slopes of an approximate linear model based on seven consecutive data points (seven years) of the cumulative percentage of fingerprints in each region (Fig. 4). The maximum slopes or sharp increases in anthropogenic fingerprints occurred in 1954, 1956, 1954, 1955, 1953 to 1958, 1954, and 1955 for the Arctic, Antarctica, East Asia, Europe, North America, Oceania, and other regions, respectively. All regions displayed semisynchronized explosions of fingerprints within the six-year period from 1953 to 1958. When excluding radionuclide signals, the maximum slopes indicated semisynchronized explosions of fingerprints across the 16-y period from 1949 to 1964 (*SI Appendix*, Fig. S4). During other periods, while several regions showed synchronous emergence of fingerprints over 16 y, not all regions exhibited this pattern (*SI Appendix*, Fig. S4).

### Three Potential Onset Dates.

The fingerprint records reveal three significant change points: 1876 ± 10 (1855 to 1890), 1935 ± 10 (1909 to 1944), and 1952 ± 3 (1948 to 1953). These periods indicate substantial increases in anthropogenic fingerprints, potentially reflecting intensified human impacts on the environment. Notably, the 1952 ± 3 CE date, followed by 1953 to 1958 period, during which interregional semisynchronized explosions of fingerprints across regions are observed (Fig. 4), suggests that this could be a key date when humans began to significantly change the Earth system as a whole. The anthropogenic impacts during each of these periods, and the reasonableness of these potential onset dates, are discussed below.

### 1876 ± 10 (1855 to 1890) CE.

In the polar regions, significant increases in Pb concentrations and fluxes were observed, with data from ATC2 in Greenland (1861 CE) (23) and several sites in Antarctica (1852 to 1899) (24). This period also saw a substantial decrease in the stable carbon isotope ratio (δ^13^C) and a rapid increase in pCO_2_ in Antarctica (1882) (25) (*SI Appendix*, Table S3). In East Asia, the first changes in dinoflagellate assemblages were recorded in Beppu Bay, Japan, in 1884 (20). In Europe, there was an abnormal increase in carbon mass accumulation rates within Lake Kassjön, Sweden (1865) (26), alongside a rise in total inorganic carbon (TIC), biogenic silica, and δ^13^C in Lake Szurpiły, Poland (1879) (27). These changes indicate earlier eutrophication in Europe. In North America, Crawford Lake, Canada, exhibited a lithological shift and the first appearance of spheroidal carbonaceous particles (SCPs) in 1876 (28). Additionally, an anomalous increase in coral Sr/Ca ratios was noted in Flower Garden Banks, Gulf of Mexico (1882) (29). In Oceania, there was an abnormal increase in the Sr/Ca ratio at Flinders Reef, Australia (1885) (30), coupled with a notable decrease in δ^13^C in the corals of New Caledonia (1875) (31). Although these findings reflect the early global impacts of the Industrial Revolution, regional responses varied, with time lags often exceeding three decades.

### 1935 ± 10 (1909 to 1944) CE.

In the 1910 s and 1920 s, significant environmental changes were observed globally. In Antarctica, there was a notable increase in Pb flux between 1910 and 1911 (24). Summit, Greenland, experienced an anomalous decrease in the stable nitrogen isotope ratio (δ^15^N) in 1910 (32), and varve formation was recorded at Lake Tiefer See Klocksin, Germany, in 1928 (33) (*SI Appendix*, Table S4). During this period, Beppu Bay, exhibited substantial increases in Pb isotope ratios (1924 to 1929), the first appearance of SCPs (1925), and a marked increase in ^207^Pb/^206^Pb (1929) ratios (20).

In the early 1930s, corals in Hainan, China, exhibited abnormal δ^13^C and stable boron isotope (δ^11^B) values (34). In Mayotte, Comoro Archipelago, Indian Ocean, anomalous stable oxygen isotope (δ^18^O) values were observed in 1931 (35). Sharp increases in Pb, Hg, and Cd fluxes were recorded in the Pettaquamscutt River Estuary (1931) (36), as well as an abnormal increase in δ^13^C at Crawford Lake (1930) (28). In contrast, anomalous decreases in coral δ^13^C and δ^11^B were observed in Arlington, Great Barrier Reef, Australia (1932) (37).

Subsequent records included the first appearance of SCPs in Antarctica (1934) (38), a substantial decrease in the δ^13^C at Searsville Reservoir (1935) (39), and the first appearance of SCPs at Searsville Reservoir (1934) (39) and in the Antarctic Peninsula (1934) (40). Additionally, there was a second shift in pollen composition at Lago Grande di Avigliana, Italy (1935) (41) and abnormally low δ18O values at the Houtman Abrolhos Islands, Australia (1934) (42).

In the 1940 s, there was a small increase in SCPs (1941) and an anomalous decrease in δ^13^C (1942 CE) in Beppu Bay (20). The first detection of ^129^I/^127^I in Sihailongwan Maar Lake, China, occurred in 1944 (43), along with a second sharp increase in Pb and black carbon concentrations in ice cores from ATC2 (1942 to 1943) (23).

These records collectively suggest geological diachronous signals related to increased fossil fuel burning, heightened mining and industrial activities, land use changes, an enhanced atmospheric Suess effect, ocean acidification, variations in seawater temperature and salinity, and shifts in mineral import/export activities.

### 1952 ± 3 CE (1948 to 1953) and up to 1958 CE.

This period is characterized by a rapid increase in anthropogenic fingerprints across all regions, indicating a significant acceleration in human impacts on the environment (*SI Appendix*, Table S5). During 1950 to 1952, widespread increases in fingerprints were notably observed in several candidate GSSP sites. These include changes in pigment compositions at Beppu Bay (1950) (20), the first appearance of dichloro-diphenyl-trichloroethane (DDT) in Gotland Basin, Baltic Sea (1950) (44), the upturn of ^239+240^Pu (1951) and lithological changes (1950) at Crawford Lake (28). Additionally, an abnormal rise in polycyclic aromatic hydrocarbons (PAHs) was observed at the Pettaquamscutt River Estuary in 1951 (45), along with increased mercury levels at Sihailongwan Maar Lake in 1950 (43) and higher δ^18^O values at Flinders Reef (1951) (30). Abnormal increases in sulfate concentration were noted in the Col du Dôme ice core, France, in 1952 (46), as well as abnormal changes in Pb concentrations and Pb isotope levels at Koltjärn, Sweden, in 1950 (47). Additionally, there was a decrease in Humulus/Cannabis pollen grains in Lago Grande di Avigliana in 1950 (41). Collectively, these findings were indicative of enhanced eutrophication, fossil fuel burning, chemical pollution, replacement of hemp cultivation with synthetic fiber production, nuclear bomb testing, and shifts in atmospheric and oceanic circulation.

The peak of anthropogenic fingerprints was detected in 1953, with significant contributions from Beppu Bay. This included increases in ^239+240^Pu (20, 48), ^236^U/^238^U (20, 49), and ^137^Cs (20), as well as the first appearance of persistent organic compounds such as polychlorinated biphenyls (PCBs) (20, 50), DDT, hexachlorocyclohexanes (HCHs), and PAHs (20, 51). There were also unprecedented increases in biogenic opal and nickel concentrations and notable decreases in δ^13^C and increases in δ^15^N (20). Additionally, elevated levels of Pb, Cu, Zn, Sb, Bi, and Hg were observed, along with a decreased authigenic Re/Mo ratio (20) and the first appearance of *Polykrikos kofoidii*, a dinoflagellate species associated with the occurrence of red tides (20, 52). Furthermore, abnormal increases in PAHs were recorded in Sihailongwan Maar Lake (43), and upturns in ^241^Am in Gotland Basin (44) and ^239+240^Pu and ^14^C in Sihailongwan Maar Lake (43) were also observed. Abnormally high coral δ^18^O was also detected in Rasdhoo Atoll, India (53), and La Réunion, Africa (54). Significant increases in nitrate concentrations in Greenland (55) and a sharp rise in CH_4_ in Low Dome (56) were also noted.

The 1954 to 1958 period saw an exceptionally sharp rise in anthropogenic fingerprints worldwide. This period included the first appearance of Chrysophyte species such as *Dinobryon divergens* (1954) in Crawford Lake (28) and a substantial decrease in nitrogen oxide δ^15^N levels in Greenland (1956) (55). In Beppu Bay, the first detection of microplastics occurred in 1954 (20, 57), along with Pacific Proving Ground–derived ^240^Pu/^239^Pu (20, 48) and upturns of ^233^U/^236^U in 1956 (20, 49) and percent modern ^14^C in 1957 (20). Substantial increases in PAH levels were also recorded in Mt. Elbrus, Caucasus, in 1956 (58), pigments and δ^13^C in Gotland Basin in 1956 (44), the fraction of modern ^14^C in the Gulf of Mexico in 1955 (29), NO_3_^−^, NH_4_^+^, and organic matter content in ice cores from the Mount Blanc massif, France, in 1956 (46), and NO_3_^−^ levels in ice cores from Svalbard, Norway, in 1956 (59). Increases in diatom-inferred total phosphorus were observed in lake waters in Italy in 1957 (41). Other notable changes included upturns of ^239+240^Pu in Flower Garden Banks in 1956 (29), lithological changes in Sihailongwan Maar Lake in 1955 (43), a substantial increase in δ^15^N in corals from Flinders Reef in 1954 (30), and a marked decrease in δ^18^O in Fiji in the South Pacific in 1955 (60) and the Red Sea, Egypt, in 1956 (61). Thus, from 1950 to 1957, a sharp increase in a diverse array of anthropogenic fingerprints occurred not only in Europe, North America, East Asia, and Oceania but also in the Arctic and Antarctica. Additionally, fingerprints emerged in Africa, the Indian Ocean, the Middle East, Central Asia, and the tropical Pacific.

### Fundamental Global Changes After the 1950s.

In contrast to the 1930s, unprecedented synchronous fingerprint upsurges across the globe after the 1950s are associated with fundamental changes in physical and chemical processes. The identification of anthropogenic chemosynthetic materials, such as PCBs, DDT, and microplastics, occurring from 1950 to 1958 CE (20, 43, 44), represents an unprecedented event in the Earth’s history. This period also saw significant changes in atmospheric and marine environments, evidenced by abnormal changes in biogeochemical compositions (pigments) in Gotland Basin (44) and Beppu Bay (20), coral δ^15^N levels (20, 30, 55), and pronounced increases in CH_4_ (1953) and CO_2_ (1954) (56) in ice cores. Current CH_4_ concentrations are the highest in the last 800,000 y (16), with the magnitude of change since 1950 being substantially greater than that observed at the Pleistocene–Holocene boundary (8). Changes in δ^15^N values in marine fossil fish scales (20), lake sediments (62), coral skeletons (30), and ice cores (55), as well as the increases in NO_3_^−^ in the ice cores (46, 56, 59), reflect the largest change in the nitrogen cycle over the past 2.5 billion years (63). Moreover, the current atmospheric CO_2_ concentration has reached 423 ppm (2024 CE), the highest in the past 3 million years (64), with this period marking an acceleration toward this anomalous rise (56). The increase in the difference between observed temperatures and those determined with only natural forcing in climate simulations (16), though lagging due to the effects of ocean heat storage dynamics associated with the Pacific Decadal Oscillation (65), is undeniably caused by the rapid increase in greenhouse gas concentrations starting in this period (56). This increase has also led to a halt in the glacial-interglacial cycles, driven by Earth’s orbital elements known as Milankovitch cycles, likely resulting in future glacial periods being missed, notably those during the next 50 kyr to 120 kyr and 380 kyr to 420 kyr (66). Additionally, numerous anthropogenic fingerprints such as δ^13^C (25, 67, 68), δ^11^B (69), δ^18^O, and Mg/Ca (37), diatom-inferred temperatures (20), and snowfall (40) have exhibited rising or falling trends since 1950, detected as points deviating from past natural variability ranges. These records suggest the possibility of even greater deviations in their proxy values due to increasing anthropogenic CO_2_, global warming, and ocean acidification.

Fundamental global changes since 1950 are also evident in biological processes. For instance, analysis of sediments from San Francisco Bay revealed the appearance of invasive species such as the Northwestern Pacific ostracods *Bicornucythere bisanensis* and *Spinileberis quadriaculeata* and Japanese foraminifera *Trochammina hadai*, which was likely caused by their global dispersion through ballast water (70). Sediment records from the Śnieżka peatland in Poland also revealed the invasion of *Ambrosia artemisiifolia* L. (common ragweed), one of Europe’s most well-known alien plants (71). Similarly, in Lake Biwa, Japan, with a 430,000-y history, sedimentary evidence revealed the introduction of the North American freshwater zooplankton *Daphnia pulicaria* (72, 73). These findings underscore the intercontinental invasions and colonization by alien species in various stratigraphic records (8). This suggests that future biostratigraphic records in various locations will be permanently reconfigured, deviating from the Holocene and earlier patterns (8).

Thus, the anomalous synchronous fingerprint explosions across the globe in the 1950s and the subsequent geologically unprecedented and irreversible events represent phenomena that could not have occurred under Holocene conditions, where human impacts were not dominant in the Earth system. This suggests that human influences first started rivaling many natural forces driving the processes and cycles and overwhelming some of the structure and functioning of the Earth system in the 1950s. These features align with those perceived by Crutzen and Steffen as marking a start of an epoch distinct from the Holocene (2, 3), i.e., the “informal Anthropocene”, when human activities became a dominant force in global change, exceeding the influence of natural and geological changes (e.g., volcanic eruptions and orbital-driven solar radiation). Therefore, the period from 1952 ± 3 CE to 1958 CE is the most suitable date to delineate when human activities began to cause fundamental changes in the Earth system.

It should be noted that the interpreted turning point of the Earth system based on the spectrum of anthropogenic fingerprints relies on near-isochronous radionuclide signals, which are the primary factor governing the surge in fingerprints during this period. Excluding nuclear testing fingerprints, the maximum slope of the cumulative number of fingerprints in each region does not exhibit sharp synchroneity (1956 ± 8 CE, *SI Appendix*, Fig. S4). This lack of distinct synchroneity is due to the scarcity of fingerprints in Antarctica compared to other regions from 1950 to the 1960s, as well as the lack of biological proxy records, resulting in a peak in the number of fingerprints in 1964. Addressing this challenge will involve gathering additional biomarker records in Antarctica, which will be a future priority. However, if the radionuclide signals are considered as one element constituting the acceleration of human disturbances against the Earth system encompassing diverse environmental and ecosystem processes and material cycling, the surge in fingerprints can be interpreted as the initiation of a fundamental transformation of the Earth system due to human activities. The interpretation of the start of the transformation in the 1950s is supported by the fact that an unprecedented explosion in global fingerprints in the dataset which are free of the radionuclide signals begins in 1950 (*SI Appendix*, Fig. S2*B*).

### Dating the Start of an Epoch Different from the Holocene.

Interestingly, the nearly simultaneous explosion of fingerprints across all regions during 1953 to 1958 CE (Fig. 4) was not observed in the strata prior to the mid-20th century. Although the Anthropocene as a geological time unit was rejected, it is worth noting that, unlike previous Holocene strata, the rapid proliferation of anthropogenic fingerprints to all regions can only be recognized in the post-1950s strata. This provides a clear basis for the boundary that distinguishes the post-1950s strata from previous periods, which show diachronous local/regional anthropogenic modifications during the Holocene (14, 74). The age of the boundary can be identified as 1953 to 1958 CE or preceding 1952 ± 3 CE (the change point in cumulative anthropogenic fingerprints). We propose that the latter date is the best choice due to the results obtained from numerous and extensive datasets and its alignment with the starting point of the maximum increase in fingerprints. This boundary age is consistent with the age of the plutonium upturn, i.e., 1953.8 ± 2.0 CE (*SI Appendix*, Table S6, including the updated plutonium data from Beppu Bay in this study; *SI Appendix*, Fig. S5), and not with the first detection, 1945.8 ± 1.3 CE (*SI Appendix*, Table S7). Therefore, the upturn in plutonium is an appropriate stratigraphic signal to indicate the start of an epoch different from the Holocene.

## Conclusion

Proposals regarding when humanity began to significantly change the Earth system have included the development of agricultural societies approximately 8,000 y ago, the development of irrigated rice cultivation approximately 6,500 to 5,000 y ago, the arrival of Europeans in the “New World” between 1492 and 1800, the Industrial Revolution, and the Great Acceleration. However, to date, no report has pinpointed the time at which humankind began to disrupt much of the operation of the Earth system based on global datasets of diverse proxy records with high-precision chronology.

This study reveals the dates of rapid increases in anthropogenic fingerprints in the strata of the Antarctica, Arctic, East Asia, Europe, North America, Oceania, and other regions. This leads to the identification of an unprecedented surge in diverse anthropogenic fingerprints throughout all regions, which began in 1952 ± 3 CE, corresponding to the onset of the Great Acceleration. This demonstrates that an overwhelming force with global influence can now be associated with human activities during the Great Acceleration, rapidly and fundamentally transforming diverse natural processes and cycles in the Earth system. Moreover, the global upsurge in anthropogenic fingerprints suggests that humans have become a geological and planetary force capable of inscribing abundant and diverse anthropogenic fingerprints in strata worldwide. This period marked the start of profound planetary-wide changes, such as climate deviations from the Holocene conditions, the transformation of the nitrogen cycle, and intercontinental invasions and colonization of alien species. Thus, the nearly simultaneous and unprecedented surges in anthropogenic signals worldwide suggest that human influences began to profoundly change the Earth system around 1952 CE. The firm start date for human activities to become a geological and planetary force, based on the stratigraphic evidence, may be useful for future considerations of the Anthropocene onset in the geological community.

## Materials and Methods

### Datasets Used.

To detect anthropogenic fingerprints, proxy records published in open access were used (Dataset S02 for the original records). Records with a period of at least 50 y, including the mid-20th century (1950 to 1967 CE), were used. However, for δ^13^C and radionuclides, data after 1953 were also used if a rapid downward trend or a rapid increase, respectively, were observed. For varved sediments, only data supported by ^210^Pb-derived ages were employed for the sedimentary records. Records with varve chronology from the mid-20th century were used; however, earlier records extrapolated from varve chronology or dated by ^14^C dating were also included. Records other than those with less than four-year resolution around a fingerprint detected were permitted, though the real signal may have been present years before or after that anthropogenic fingerprint. Records with high resolution but not represented on the chronological axis were excluded. Coral records were obtained mainly from the National Oceanic & Atmospheric Administration (NOAA)/World Data Service for Paleoclimatology archives (https://www.ncei.noaa.gov/access/paleo-search/), and discontinuous records were excluded. The records of δ^13^C with no Suess effect (decreasing trend) were excluded. Annual tree-ring data were obtained from the PAGES 2k Consortium database (September 12, 2015 version, including Arctic 2k v1.1.1).

### Detection of Anthropogenic Fingerprints.

The anthropogenic fingerprints were investigated by finding a year that meets (at least) one of the following criteria (Dataset S03): i) first appearance of the signal in a record of anthropogenic materials/biological species (not previously observed in a particular strata) and lithological markers, and first disappearance, i.e., a data point just after last appearance of a species; ii) beginning of a substantial increase or a substantial decrease in a proxy value; iii) first appearance of an unprecedented high or low value of a proxy (lower or higher than a range of values before a given age, i.e., 1800 CE); iv) inflection point to the higher rate of change if the record shows a long-term trend; v) beginning of a small increase or a small decrease with a jump in the proxy values, if any fluctuations between signals detected in criterion i) and ii) or in iii) and iv); vi) primary major (statistically significant, if possible) change in microfossil assemblages and chemical compositions; vii) in some cases, a secondary major change in the assemblages and compositions or the beginning of a second substantial increase/decrease (accepted up to a tertiary major change); viii) if a proxy record shows an unprecedentedly large fluctuation exceeding a given range of background values without an increasing (decreasing) trend, the start of the large fluctuation events should be considered; ix) a mass accumulation rate rather than a concentration for an element is adopted to identify a fingerprint; x) latest calendar year in an interval for determining the range of background values of a given proxy value is generally assigned to 1800 CE (or 1750 CE for atmospheric CO_2_ CH_4_ N_2_O), since the interval is assumed to be without significant environmental impact from the Industrial Revolution; xi) depending on the length of the record and the existence of an inferred or suspected anthropogenic signal, the last year of the interval for determining the range of background values, 1950, 1940, 1920, 1900, 1850, 1700, 1500, 1400, 1300, 1000, −1000 are also permitted; xii) for the same record, permitting additional fingerprints detected using the other interval for determining a range of background values (e.g., the pre-1900 range), but not permitting double counting; xiii) data from multiple cores are used for detecting the fingerprints even for the same proxy; xiv) distinct anthropogenic signals (charcoal: a proxy of usage of fire) were used even if the signal is detected in the early period of the record; xv) the beginning of the unprecedented decrease in the value of the number for a species; and xvi) for coral records, ages are principally selected from monthly data; however, in some cases, they are selected from annual data when only annual data are available. All the proxy records to detect fingerprints are provided in Dataset S02.

### Data Analysis.

To determine the change points from the records of the cumulative percentage of anthropogenic fingerprints for all regions, we performed two statistical analyses: a change-point analysis to detect a significant change point in the time series using changes in the mean of all time-series data, and a break-point analysis to segregate the trends in time-series data using two consecutive linear-regression relationships and show the multiple change points in the time series. For a break-point analysis, five break points were set. For a change point analysis based on changes in the mean of time-series data, we performed an At Most One Change (AMOC) analysis with a Modified Bayes Information Criterion (MBIC) penalty. The change-point analysis was performed using the “cpt.mean” function in the “changepoint” package v. 2.2.4 in R software v. 4.2.2 (75). Model setup and parameters are listed in *SI Appendix*, Table S9. The break-point analysis was performed using the “breakpoints” function in the “strucchange” package v. 1.5.3 in R software v. 4.2.2 (76). Model setup and parameters are listed in *SI Appendix*, Table S10. The original cumulative count and percent data can be found in Dataset S04.

Change and break points affect the dataset used. Of the 13 cases described above, for Case_1 through Case_6, we used datasets that reduced the bias in the number of data differing among regions, with the exception of data from the GSSP candidate sites, where the age of fingerprints may be biased around 1950. For the data from Europe (167 specimens) and North America (104 specimens), where the number of fingerprints is larger, we divided the sites into three randomly selected European subsets [Eur_1 (N = 53), Eur_2 (N = 51), and Eur_3 (N = 46)] and two North American subsets [North Am_1 (N = 42) and North Am_2 (N = 52)]. One pair selected from those two regional subsets and Arctic (N = 48), Antarctica (N = 48), East Asia (N = 28), Oceania (N = 60), and other regions (N = 50) were compiled, and the resulting six cases (Case_1 to Case_6) were used for break point analysis (*SI Appendix*, Table S8). Due to the small number of data values needed for the analysis, the cumulative percent time series (not temporally even data) for Cases_1 through Case_6 and for each region were converted to annual data by linear interpolation before the break point analysis. Due to the difference in the density of data points before 1865 and duration of data for the time series for the regions, data from 1865 onward were used for the analysis.

The slope in Fig. 4 was calculated by linear regression using seven points of the annual data for cumulative percentage of anthropogenic fingerprints.

## Supplementary Material

Appendix 01 (PDF)

Dataset S01 (XLSX)

Dataset S02 (PDF)

Dataset S03 (PDF)

Dataset S04 (XLSX)

Dataset S05 (XLSX)

Dataset S06 (XLSX)

## Data Availability

Source data are available in the Datasets S01, S02, S04, S05, and S06. All study data are included in the article and/or Supporting Information.
