# Supplementary material for: Toward defining the Anthropocene onset using a rapid increase in anthropogenic fingerprints in global geological archives
Source: Proc Natl Acad Sci U S A. 2024 Sep 23;121(41):e2313098121. doi: 10.1073/pnas.2313098121 (PMC11474069; doi:10.1073/pnas.2313098121)
Supplement: Supplementary file 1 — Appendix 01 (PDF) [file pnas.2313098121.sapp.pdf]

PNAS

Supporting Information for

Toward defining the Anthropocene onset using a rapid increase in anthropogenic fingerprints in global geological archives

Michinobu Kuwae, Yusuke Yokoyama, Stephen Tims, Michaela Froehlich, L. Keith Fifield, Takahiro Aze, Narumi Tsugeki, Hideyuki Doi, and Yoshiki Saito

Michinobu Kuwae
Email: kuwae.michinobu.mc@ehime-u.ac.jp

This PDF file includes:

Materials and Methods
Results
Scripts for change and break point analysis using R
Figures S1 to S7
Tables S1 to S10
SI References

Other supporting materials for this manuscript include the following:

Dataset S01 to Dataset S06

Materials and Methods

Plutonium analysis. The core samples around the proposed GSSP level were analyzed to examine the precise level and age of the plutonium upturn in the candidate GSSP (or Standard Auxiliary Boundary Stratotype, or SABS) core, BMC21 S1–5, collected from Beppu Bay, the core samples around the proposed GSSP level were analyzed (1). The sampling depths are shown in Fig. S6. The methodology for plutonium measurement has been previously reported (1). Plutonium isotopic ratios and concentrations from core BMC21 S1–5 were determined by Accelerator Mass Spectrometry (AMS) using the 14UD pelletron accelerator at the Australian National University based on the original methods described by Fifield (2), with previously described modifications (1). The sediment material was prepared according to a previously described method (3). Briefly, the sample preparation processes involved adding ^{242}Pu (approximately 4 pg) as a yield tracer, leaching the sediment with HNO_3, and separating Pu using an ion-exchange column. The Pu was then dispersed in an iron oxide matrix, mixed with silver powder, pressed into a sample holder, and subsequently loaded into the ion source of the accelerator.

Results

Plutonium record from the Beppu Bay site. Plutonium records from Beppu Bay site are shown in Fig. S5. A sample taken at 64–65 cm depth for the BMC21 S1–5 core included layers from 1952 and 1953 CE published in (1). Data after 1955 CE for the BMC21 S1–5 core and the BMC19 S1–2 core are obtained from (1, 4). Plutonium radioactivity in Beppu Bay sediments for the candidate GSSP core, BMC21 S1-5, showed low levels (0.0312±0.0039 mBq/g) in the layer including the bottom of 1953 layer and 1952 layer at a core depth of 64.0-65.0 cm (SI Appendix, Fig. S6). The 1952–1953 layer showed values comparative to that in the 1945 CE layer (0.0296±0.0055 mBq/g) which indicates an initial increase in the radioactivity. Therefore, the 1952 CE layer is still the low level and the upturn of plutonium starts in 1953.5 CE as seen in the records from BMC21 S1-5 and BMC19 S1-2. The 63–64 cm sample in the candidate GSSP core includes the potential GSSP level of the Anthropocene (64.6 cm, 1953.5 CE), corresponding the start of the plutonium upturn (SI Appendix, Table S6).

Scripts for change and break point analysis using R

```
#change point analysis
library(tidyverse)
library(changepoint)
set.seed(123)

kuwae<-read.csv("Change point analysis_11_Jan_2024.csv")

cpt.result <- kuwae %>% na.exclude() %>%
  group_by(data) %>%
  do(cpt_result =unlist(cpts(cpt.mean(.$Cumulative..,))))
cpt.result


#break point analysis

library(strucchange)
kuwaebreak<-read.csv("Change point analysis_11_Jan_2024.csv")

kuwaere=kuwaebreak %>% group_by(data) %>% do(changeyear=.[.$breakpoint[[1]],]$Age)
kuwaere
```

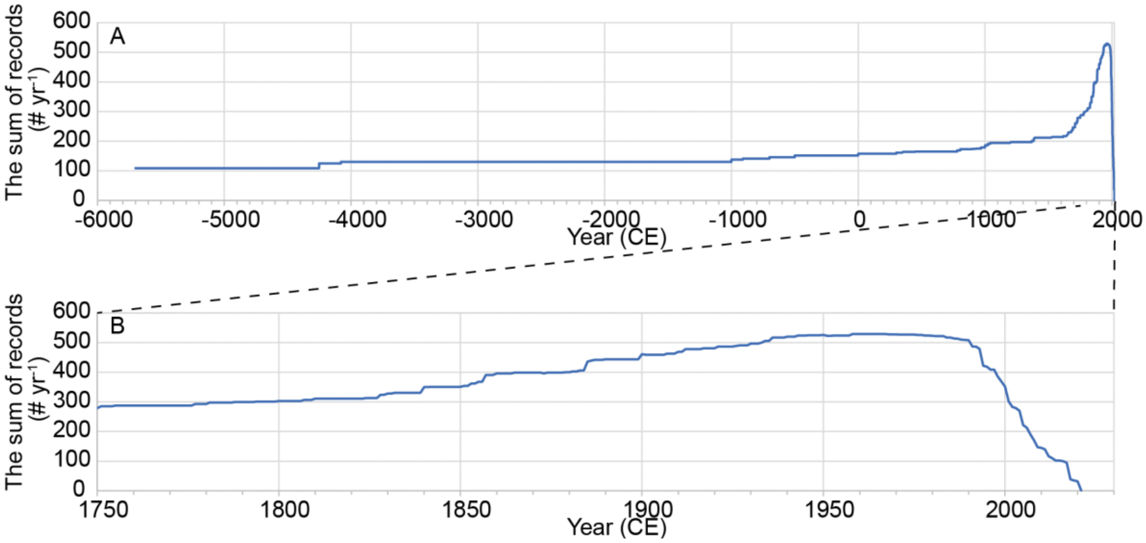

Figure S1. The number of available records for each year used for fingerprints detection. (*A*) Full range of ages and (*B*) the enlarged record from 1750 to 2021 CE. Anthropogenic novel materials and radionuclides as well as new species or species extinction were counted in the number of records per year as records existed up to 5700 BCE, beyond the period when such records exist, since it is certain that there were no anthropogenic novel materials, radionuclides before their first detections, and that species did not appear or become extinct before that time. The data are found in Dataset S06.

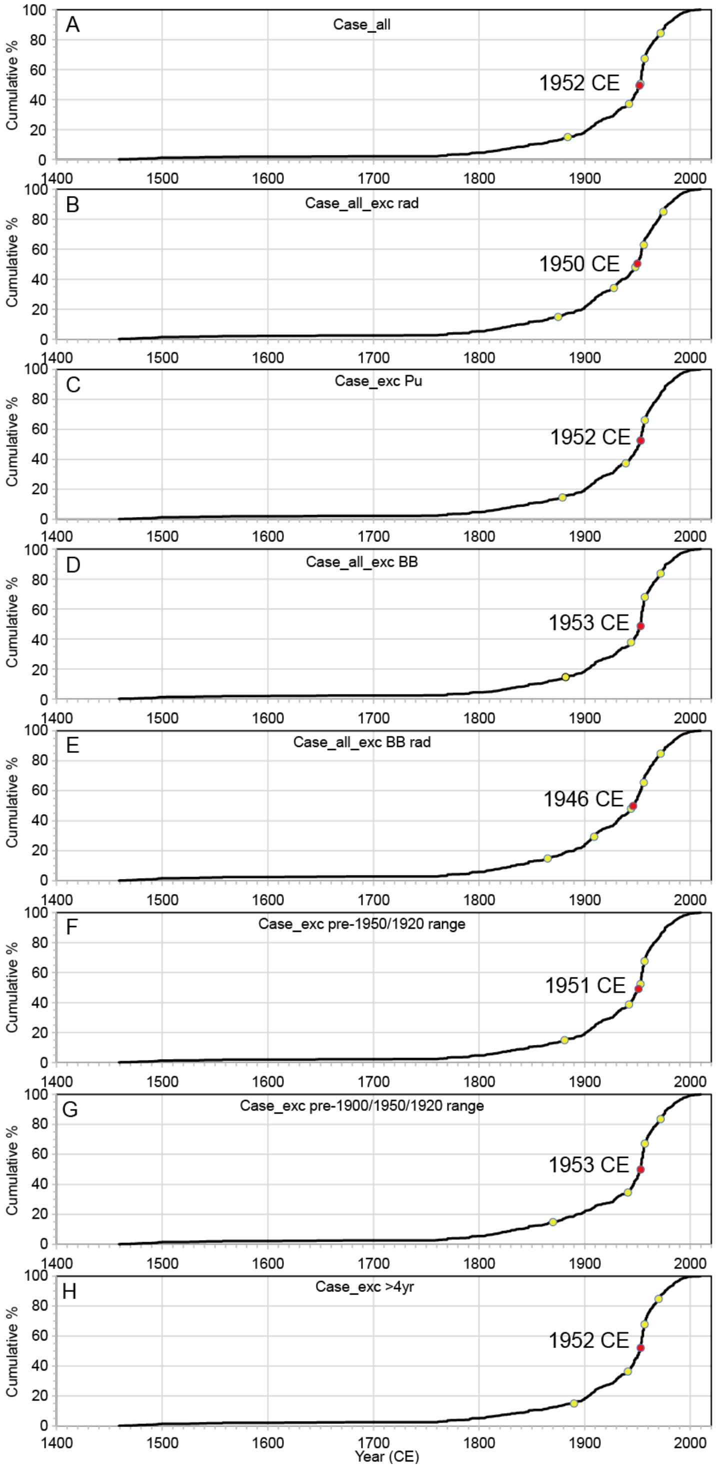

Figure S2. Cumulative percentages of anthropogenic fingerprints for all data and subsets of several cases. Red and yellow circles denote the change points derived from the one-point change-point analysis and five-point break-point analysis, respectively, for the last 550 years. Data used for these analyses are provided in Dataset S04. The model settings and parameters are listed in SI Appendix, Table S9 and S10.

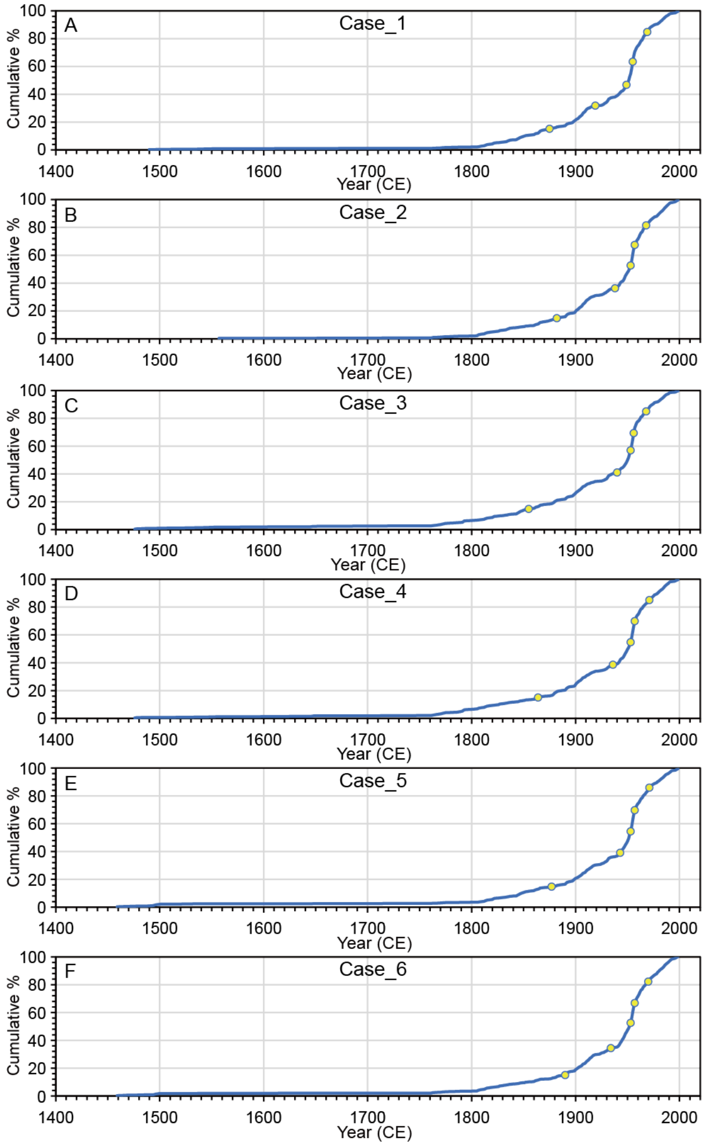

Figure S3. Cumulative percentages of anthropogenic fingerprints for subsets of several cases without the GSSP candidate data. Yellow circle denotes the change point derived from the five-point break-point analysis for the last 550 years. Data used for the analysis are provided in Dataset S04. Combination of regional datasets used for each case for the analysis are shown in Table S8. The model settings and parameters are listed in Table S10.

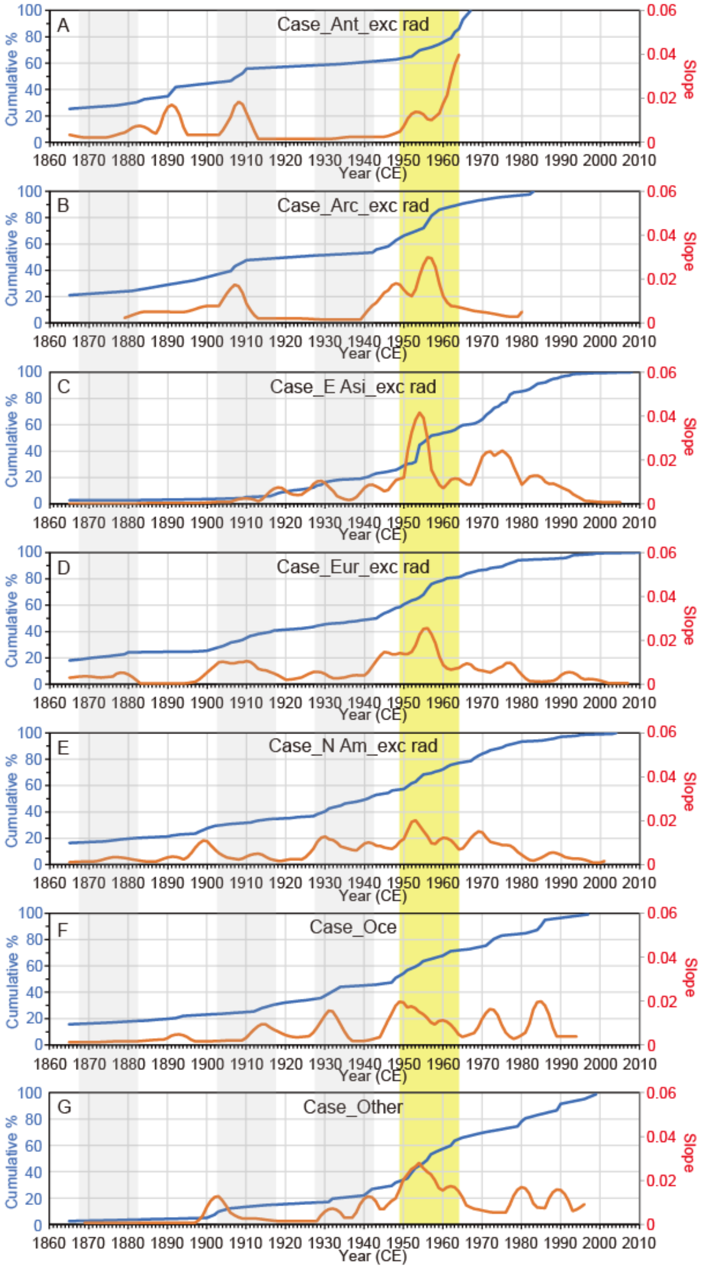

Figure S4. Cumulative percentage of anthropogenic fingerprints and the slope for each region, but excluding the radionuclide data. (A) Case_Ant, (B) Case_Arc, (C) Case_E Asi, (D) Case_Eur, (E) Case N Am, (F) Case_Oce, and (G) Case_Other denotes subsets of fingerprint data from Antarctica, Arctic, East Asia, Europe, North America, Oceania, and the other regions. Yellow shade denotes nearly simultaneous unprecedented increases in fingerprints with maximum slopes in 1949-1964 CE. Gray shade denotes a 16-yr period with the potential simultaneous increase in fingerprints between regions.

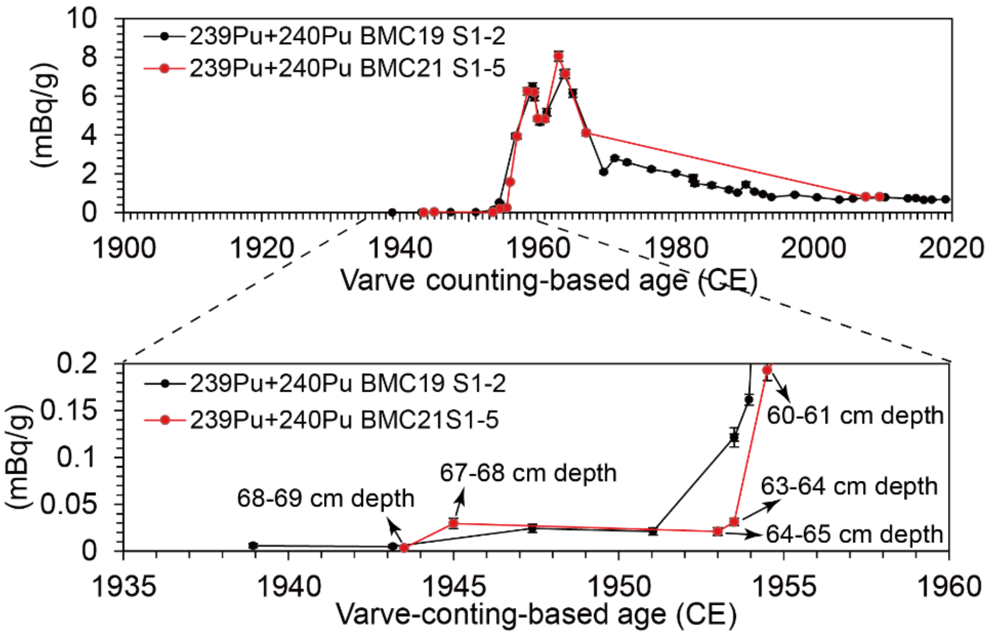

Figure S5. Plutonium records from Beppu Bay site. Red line with a red circle denotes the candidate GSSP core data. A sample of 64–65 cm depth included layers from 1952 and 1953 CE. Data after 1955 CE for the BMC21 S1–5 core and the BMC19 S1–2 core are obtained from (1, 4). Plutonium radioactivity in Beppu Bay sediments for the candidate GSSP core, BMC21 S1-5, showed low levels (0.0312±0.0039 mBq/g) in the layer including the bottom of 1953 layer and 1952 layer at a core depth of 64.0-65.0 cm (SI Appendix, Fig. S6). The 1952–1953 layer showed values comparative to that in the 1945 CE layer (0.0296±0.0055 mBq/g) which indicates an initial increase in the radioactivity. Therefore, the 1952 CE layer remains at low level and the upturn of plutonium begins in 1953.5 CE as seen in the records from BMC21 S1-5 and BMC19 S1-2. The 63–64 cm sample in the candidate GSSP core includes the potential GSSP level of the Anthropocene (64.6 cm, 1953.5 CE), corresponding the start of the plutonium upturn (SI Appendix, Table S6).

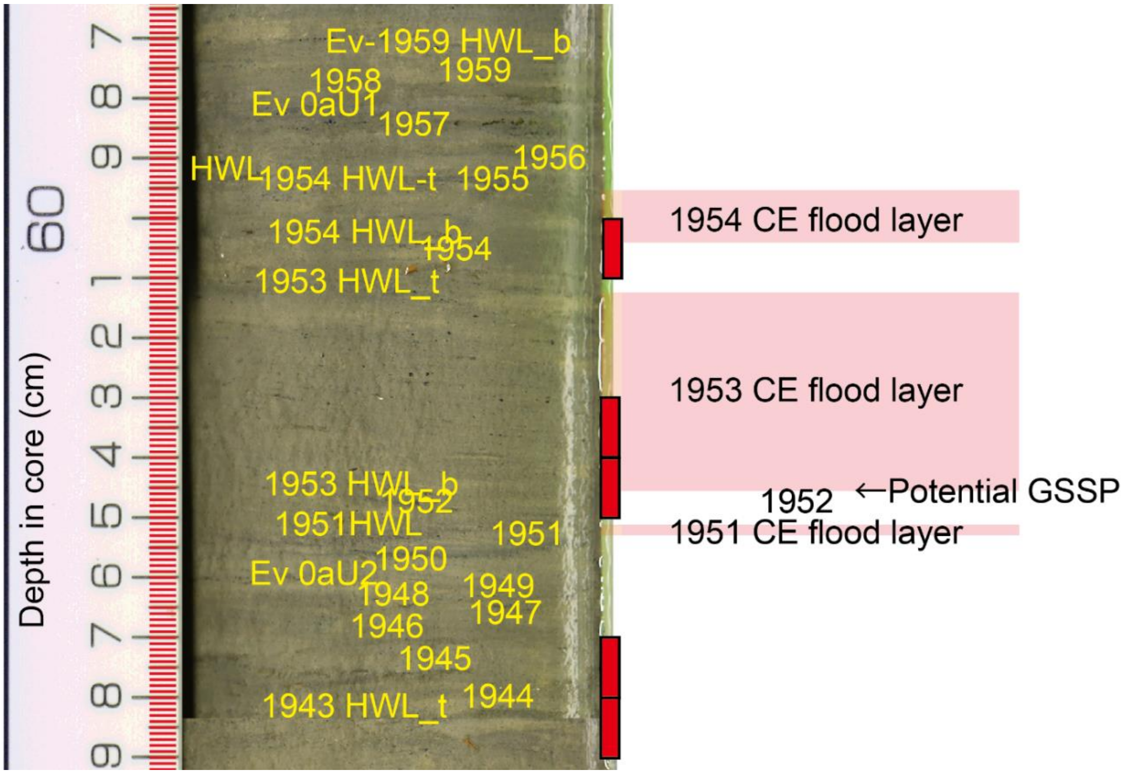

Fig. S6. Sample depth used for plutonium analysis for potential standard auxiliary boundary stratotype core collected from Beppu Bay. Red rectangles denote the sampling depth at which data are reported here.

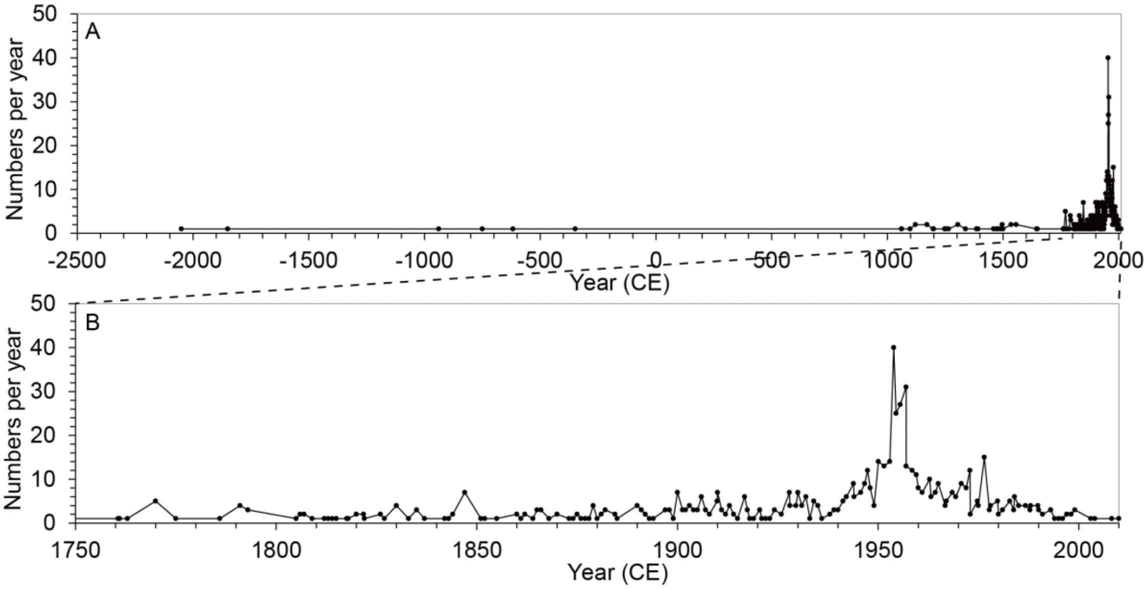

Figure S7. Temporal changes in anthropogenic fingerprints per year detected from global geological archives for the last 7700 years. (*A*) The full-range record from 2050 BCE (not shown before 2500 BCE with no fingerprints). (*B*) the enlarged record from 1700 to 2000 CE. The data are found in Dataset S05.

Table S1. An interval of a record used, the number of fingerprints, and a year detected by one-point change-point analysis for each case.

Case ID	Start year	End year	Number	Change point
Case_all	1459	2010	724	1952
Case_all_exc rad	1459	2010	627	1950
Case_exc Pu	1459	2010	678	1952
Case_all_exc BB	1459	2010	617	1953
Case_all_exc BB rad	1459	2010	539	1946
Case_exc pre-1900/1950/1920 range	1459	2010	690	1953
Case_exc pre-1950/1920 range	1459	2010	600	1951
Case_exc >4yr	1459	2010	620	1952

Table S2. An interval of a record used, a year detected by five-point break-point analysis, and an interval showing the maximum slope for each case.

Case ID	Start year	End year	Change_1	Change_2	Change_3	Change_4	Change_5	Slope_st_1	Slope_1_2	Slope_2_3	Slope_3_4	Slope_4_5	Slope_5_end	Max_slope
Case_all	1459	2010	1884	1942	1953	1957	1972	0.0003	0.0041	0.0135	0.0409	0.0104	0.0049	3-4
Case_all_exc rad	1459	2010	1875	1928	1948	1956	1974	0.0003	0.0040	0.0063	0.0205	0.0111	0.0051	3-4
Case_exc Pu	1459	2010	1879	1939	1953	1957	1972	0.0003	0.0041	0.0109	0.0349	0.0109	0.0050	3-4
Case_all_exc BB	1459	2010	1882	1944	1953	1957	1972	0.0003	0.0039	0.0169	0.0393	0.0108	0.0048	3-4
Case_all_exc BB rad	1459	2010	1865	1909	1944	1956	1972	0.0003	0.0032	0.0049	0.0163	0.0111	0.0050	3-4
Case_exc pre-1900/1950/1920 range	1459	2010	1870	1941	1953	1957	1972	0.0003	0.0028	0.0139	0.0400	0.0108	0.0048	3-4
Case_exc pre-1950/1920 range	1459	2010	1881	1942	1953	1957	1972	0.0003	0.0042	0.0137	0.0376	0.0103	0.0047	3-4
Case_exc >4yr	1459	2010	1890	1941	1953	1957	1970	0.0003	0.0041	0.0139	0.0417	0.0114	0.0050	3-4
Case_1	1490	1999	1875	1919	1949	1955	1969	0.0004	0.0043	0.0054	0.0304	0.0151	0.0050	3-4
Case_2	1557	1999	1882	1938	1953	1956	1968	0.0007	0.0044	0.0117	0.0412	0.0132	0.0062	3-4
Case_3	1476	1999	1855	1940	1953	1956	1968	0.0003	0.0033	0.0125	0.0421	0.0127	0.0050	3-4
Case_4	1476	1999	1864	1936	1953	1957	1971	0.0004	0.0036	0.0115	0.0390	0.0106	0.0057	3-4
Case_5	1459	1999	1877	1943	1953	1957	1971	0.0003	0.0040	0.0154	0.0396	0.0110	0.0051	3-4
Case_6	1459	1999	1890	1934	1953	1957	1970	0.0003	0.0045	0.0121	0.0375	0.0119	0.0065	3-4

Change_1 denotes detected the first break-point year.
Slope_st_1 denotes the slope obtained from the interval from start year to break-point 1.
Max_slope denotes the interval detecting the maximum slope in the timeseries.

Table S3. Anthropogenic fingerprints during 1876±10 (1855-1890) CE.

Fingerprint ID	Proxy	Age	Criteria for fingerprint detection	Site	Country	Regeion	Latitude, longitude
552	$\delta^{13}C$, pCO2	1882	Beginning of a substantial decrease	Low Dome		Antarctica	66.77°S, 112.80°E
573	N_2O concentrations	1877	Beginning of a small increase with a jump	Low Dome		Antarctica	66.73°S, 112.83°E
600	Pb flux	1866	Higher than pre-1800 range	WD059		Antarctica	79.47°S, 112.07°E
608	Pb flux	1884	Beginning of a small increase with a jump	NUS08-5		Antarctica	82.63°S, 17.87°E
609	Pb flux	1860	Higher than pre-1800 range	NUS08-5		Antarctica	82.63°S, 17.87°E
615	Pb flux	1890	Beginning of a small increase with a jump	NUS07-1		Antarctica	74.72°S, 1.05°E
559	$\delta^{15}N$	1860	Lower than pre-1800 range	Greenland		Arctic	72.60ºN, 38.50ºW
588	Pb conentrations	1881	Beginning of a small increase with a jump	ATC2, Greenland		Arctic	66.0°N, 45.2°W
589	Pb conentrations	1861	Higher than pre-1800 range	ATC2, Greenland		Arctic	66.0°N, 45.2°W
592	Cd concentrations	1890	Beginning of a small increase with a jump	ATC2, Greenland		Arctic	66.0°N, 45.2°W
593	Cd concentrations	1864	Higher than pre-1800 range	ATC2, Greenland		Arctic	66.0°N, 45.2°W
595	Black carbon	1890	Beginning of a substantial increase	ATC2, Greenland		Arctic	66.0°N, 45.2°W
596	Black carbon	1881	Beginning of a small increase with a jump	ATC2, Greenland		Arctic	66.0°N, 45.2°W
83	Dinoflagellate assemblage (%)	1884	First change	Beppu Bay	Japan	East Asia	33.2778ºN, 131.5373ºE
293	Cabon mass accmulation rate	1865	Higher than pre-1800 range	Kassjön	Sweden	Europe	63.93ºN, 20.01ºE
296	Biovolume accumulation rate of a planktonic diatom, Tabellaria flocculosa	1865	Beginning of a substantial increase	Kassjön	Sweden	Europe	63.93ºN, 20.01ºE
326	Tree-ring width	1870	Higher than pre-1800 range	France	France	Europe	44.05ºN, 07.28ºE
341	Sedimentation rate	1868	First appearance	Lake Rõuge Tõ ugjärv	Estonia	Europe	57.74ºN, 26.90ºE
342	pollen grain compositions	1875	Second major change	Lake Rõuge Tõ ugjärv	Estonia	Europe	57.74ºN, 26.90ºE
359	TOC/varve formation	1873	First appearance	Sacrower See	Germany	Europe	52.27ºN, 13.06ºE
365	Biogenic silica	1870	Lower than pre-1800 range	Sacrower See	Germany	Europe	52.27ºN, 13.06ºE
368	Varve formation	1879	First appearance	Lake Wąsoskie	Poland	Europe	52.95ºN,17.74ºE
382	Diatom compositions	1862	Second change	Lake Szurpiły	Poland	Europe	54.23ºN, 22.89ºE
391	Total inorganic carbon (TIC)	1879	Higher than pre-1700 range	Lake Szurpiły	Poland	Europe	54.23ºN, 22.89ºE
392	Biogenic silica	1879	Higher than pre-1700 range	Lake Szurpiły	Poland	Europe	54.23ºN, 22.89ºE
393	$\delta^{13}C$	1879	Higher than pre-1700 range	Lake Szurpiły	Poland	Europe	54.23ºN, 22.89ºE
400	Pollen grain compositions	1880	First major change	Lago Grande di Avigliana	Italy	Europe	45.07ºN, 07.39ºE
662	$\delta^{18}O$	1865	Lower than pre-1800 range	Ras Umm Sidd, Red Sea	Egypt	Middle East	27.85°N,34.31°E
153	Lithological change (colour)	1874	First appearance	Crawford Lake	Canada	North America	43.4686ºN, 79.9487ºW
163	SCP	1876	First appearance	Crawford Lake		North America	43.4686ºN, 79.9487ºW
169	Pollen assemblages	1855	Third change	Crawford Lake		North America	43.4686ºN, 79.9487ºW
175	Zooplankton compositions	1890	Second change	Crawford Lake		North America	43.4686ºN, 79.9487ºW
258	Trace (Sr/Ca)	1882	Higher than pre-1800 range	Flower Garden Banks, Gulf of Mexico	USA	North America	27.8762ºN, 93.8147ºW
332	Tree-ring	1866	Higher than pre-1800 range	Sheep Mountain, California	USA	North America	37.22ºN, -118.13ºW
334	Tree-ring	1866	Beginning of a small increase with a jump	Glass Mountain	USA	North America	37.45ºN, -118.41ºW
548	$\delta^{13}C$	1878	Beginning of a substantial decrease	Rio Bueno and Montego Bay (Ce96-1)	Jamaica	North America	18.456°N, 77.957ºW
724	Pb concentrations	1882	Beginning of a small increase with a jump	John Smith's Bay	Bermuda	North America	32.47°N, 64.77°W
229	Trace (Sr/Ca)	1885	Higher than pre-1800 range	Flinders Reef	Australia	Oceania	17.7179ºS, 148.4510ºE
504	Intensity of luminescent (Running 21-yr standard deviation)	1862	Higher than pre-1800 range	Great Barrier Reef (Havannah)	Australia	Oceania	18.84ºS, 146.54ºE
644	$\delta^{13}C$	1875	Lower than pre-1800 range	New Caledonia	New Caledonia	Oceania	22.5°S, 166.5°E

Table S4. Anthropogenic fingerprints during 1935±10 (1909-1944) CE.

Fingerprint ID	Proxy	Age	Criteria for fingerprint detection	Site	Country	Regeion	Latitude, longitude
483	$\delta^{13}C$	1941.0	Lower than pre-1800 range	Lake Bosumtwi	Ghana	Africa	6.51ºN, -1.41ºE
654	$\delta^{18}O$	1931	Lower than pre-1900 range	Mayotte, Comoro Archipelago, Indian Ocean		Africa	12.65°S, 45.1°E
659	$\delta^{18}O$	1942	Beginning of a small decrease with a jump	La Réunion, Indian Ocean		Africa	21°S, 55°E
260	SCP	1934.0	First appearance	Antarctic Peninsula Ice Core		Antarctica	73.8521°S, 65.4526°W
263	Snowfall	1909.0	Higher than pre-1800 range	Antarctic Peninsula Ice Core		Antarctica	73.8521°S, 65.4526°W
604	Pb flux	1910	Beginning of a substantial increase	NUS08-4		Antarctica	82.82°S, 18.9°E
611	Pb flux	1934	Beginning of a small increase with a jump	NUS08-7		Antarctica	74.88°S, 0.70°E
614	Pb flux	1909	Beginning of a substantial increase	NUS07-1		Antarctica	74.72°S, 1.05°E
558	$\delta^{15}N$	1910	Beginning of a small decrease with a jump	Greenland		Arctic	72.60ºN, 38.50ºW
586	Pb conentrations	1943	Beginning of a second increase with a jump	ATC2, Greenland		Arctic	66.0°N, 45.2°W
594	Black carbon	1942	Beginning of a second increase with a jump	ATC2, Greenland		Arctic	66.0°N, 45.2°W
747	$\delta^{15}N$	1927	Beginning of a small increase with a jump	Summit, Greenland		Arctic	72.5°N, 38.4°W
748	$\delta^{15}N$	1910	Lower than pre-1800 range	Summit, Greenland		Arctic	72.5°N, 38.4°W
11	$^{129}I^{127}I$	1943.9	First appearance	Beppu Bay	Japan	East Asia	33.2778ºN, 131.5373ºE
20	SCPs	1925.9	First appearance	Beppu Bay	Japan	East Asia	33.2733ºN, 131.5395ºE
34	1,4-Dichlorobenzene	1927.7	First appearance	Beppu Bay	Japan	East Asia	33.2778ºN, 131.5373ºE
37	4-Methyl-2,6-di-t-butylphenol	1927.7	First appearance	Beppu Bay	Japan	East Asia	33.2778ºN, 131.5373ºE
47	Total nitrogen (TN)	1909.9	Higher than pre-1900 range	Beppu Bay	Japan	East Asia	33.2778ºN, 131.5373ºE
52	Water content-corrected Br	1927.9	Higher than pre-1900 range	Beppu Bay	Japan	East Asia	33.2778ºN, 131.5373ºE
56	$\delta^{13}C$	1942.1	Lower than pre-1900 range	Beppu Bay	Japan	East Asia	33.2778ºN, 131.5373ºE
59	$\delta^{15}N$	1917.4	Higher than pre-1800 range	Beppu Bay	Japan	East Asia	33.2778ºN, 131.5373ºE
66	^{207}Pb / ^{206}Pb	1929.5	Beginning of a substantial increase	Beppu Bay	Japan	East Asia	33.2778ºN, 131.5373ºE
67	^{207}Pb / ^{206}Pb	1920.5	Higher than pre-1920 range	Beppu Bay	Japan	East Asia	33.2778ºN, 131.5373ºE
69	^{208}Pb / ^{206}Pb	1924.2	Beginning of a substantial increase	Beppu Bay	Japan	East Asia	33.2778ºN, 131.5373ºE
71	*Cryptomeria* pollen assemblages/ deposition rates	1916.8	Third major change	Beppu Bay	Japan	East Asia	33.2778ºN, 131.5373ºE
88	Foraminifera conc.	1941.3	Beginning of a substantial decrease	Beppu Bay	Japan	East Asia	33.2778ºN, 131.5373ºE
91	SCEs	1929.4	Beginning of a small increase with a jump	Beppu Bay	Japan	East Asia	33.2778ºN, 131.5373ºE
92	SCEs	1916.0	Higher than pre-1800 range	Beppu Bay	Japan	East Asia	33.2778ºN, 131.5373ºE
188	$^{129}I/^{1270}I$	1944.0	First appearance	Sihailongwan Maar Lake	China	East Asia	42.2868ºN, 126.6012ºE
191	Soot ^{14}C	1944.0	First appearance	Sihailongwan Maar Lake	China	East Asia	42.2868ºN, 126.6012ºE
197	SCP	1941.0	First appearance	Sihailongwan Maar Lake	China	East Asia	42.2868ºN, 126.6012ºE
199	Trace elements (Pb)	1939.0	Higher than pre-1900 range	Sihailongwan Maar Lake	China	East Asia	42.2868ºN, 126.6012ºE
205	DNA (Shanon Index for phytoplankton)	1922.0	Lower than pre-1900 range	Sihailongwan Maar Lake	China	East Asia	42.2868ºN, 126.6012ºE
209	Black carbon	1934.0	Higher than pre-1900 range	Sihailongwan Maar Lake	China	East Asia	42.2868ºN, 126.6012ºE
213	Charcoal concentration	1932.0	Beginning of a small increase with a jump	Sihailongwan Maar Lake	China	East Asia	42.2868ºN, 126.6012ºE
214	Charcoal concentration	1909.0	Higher than pre-1900 range	Sihailongwan Maar Lake	China	East Asia	42.2868ºN, 126.6012ºE
235	^{239}Pu	1943.0	First appearance	Ishigaki	Japan	East Asia	24.58°N, 124.33°E
316	Fe2O3	1918.0	Higher than pre-1900 range	Lake Mokoto	Japan	East Asia	43.96ºN, E144.32ºE
511	$\delta^{11}B$	1930	Lower than pre-1900 range	Hinan, South China Sea	China	East Asia	19.283°N, 110.65ºE
512	$\delta^{13}C$	1940	Lower than pre-1900 range	Hinan, South China Sea	China	East Asia	19.283°N, 110.65ºE
514	$\delta^{11}B$	1919	Lower than pre-1900 range	South China Sea	China	East Asia	18.2°N, 109.499ºE
666	$\delta^{18}O$	1944	Lower than pre-1800 range	Hainan Island, South China Sea	China	East Asia	19.29°N,110.66°E
667	$\delta^{18}O$	1930	Lower than pre-1900 range	Hainan Island, South China Sea	China	East Asia	19.29°N,110.66°E
668	$\delta^{13}C$	1942	Lower than pre-1900 range	Hainan Island, South China Sea	China	East Asia	19.29°N,110.66°E
118	Total nitrogen (TN)	1943.0	Higher than pre-1900 range	Gotland Basin, Baltic Sea		Europe	57.2830ºN, 20.1204ºE
120	TOC/TN	1917.0	Lower than pre-1900 range	Gotland Basin, Baltic Sea		Europe	57.2830ºN, 20.1204ºE
122	$\delta^{13}C$	1943.0	Higher than pre-1900 range	Gotland Basin, Baltic Sea		Europe	57.2830ºN, 20.1204ºE
124	SCPs	1920.0	Beginning of a small increase with a jump	Gotland Basin, Baltic Sea		Europe	57.2830ºN, 20.1204ºE
127	Trace elements (Hg, Pb, Zn Cu)	1910.0	Higher than pre-1900 range	Gotland Basin, Baltic Sea		Europe	57.2830ºN, 20.1204ºE
269	$\delta^{15}N$	1912.0	Beginning of a small increase with a jump	Baldeggersee		Europe	47.198ºN, 8.262ºE
295	Biovolume accumulation rate of a planktonic diatom, Tabellaria flocculosa	1932.0	Beginning of a substantial decrease	Kassjön	Sweden	Europe	63.93ºN, 20.01ºE
300	Diatom compositions	1927.0	Second major change	Baldeggersee	Switzerland	Europe	47.198ºN, 8.262ºE
307	Grain size	1909.0	Beginning of a small increase with a jump	Baldeggersee	Switzerland	Europe	47.198ºN, 8.262ºE
338	SCPs	1927.0	Beginning of a small increase with a jump	Lake Rõuge Tõugjärv	Estonia	Europe	57.74ºN, 26.90ºE
340	Sedimentation rate	1939.0	Beginning of a substantial increase	Lake Rõuge Tõugjärv	Estonia	Europe	57.74ºN, 26.90ºE
345	amount of mineral matter	1929.0	Higher than pre-1800 range	Lake Korttajärvi	Finland	Europe	61.03ºN, 25.12ºE
346	varve thickness	1910.0	First appearance	Baldeggersee	Switzerland	Europe	47.198ºN, 8.262ºE
349	δ18O (dark layer)	1943.0	Lower than pre-1900 range	Baldeggersee	Switzerland	Europe	47.198ºN, 8.262ºE
351	δ18O (light layer)	1910.0	Higher than pre-1900 range	Baldeggersee	Switzerland	Europe	47.198ºN, 8.262ºE
358	OC mass accmulation rate	1938.0	Higher than pre-1800 range	Sacrower See	Germany	Europe	52.27ºN, 13.06ºE
367	Varve formation	1943.0	First appearance	Lake Salno	Poland	Europe	53.49ºN, 18.96ºE

Table S4 continued.

Fingerprint ID	Proxy	Age	Criteria for fingerprint detection	Site	Country	Regeion	Latitude, longitude
375	Hg mass accmulation rates	1930.0	Beginning of a substantial increase	Lake Montcortès	Spain	Europe	42.32ºN, 0.98ºE
399	Pollen grain compositions	1935.0	Second major change	Lago Grande di Avigliana	Italy	Europe	45.07ºN, 07.39ºE
408	Tree pollen	1936.0	Higher than pre-1900 range	Lago Grande di Avigliana	Italy	Europe	45.07ºN, 07.39ºE
411	Total flux	1913.0	Higher than pre-1900 range	Lake Bourget	French	Europe	45.75ºN, 55.03ºE
414	Large calcite crystals	1944.0	Beginning of a small increase with a jump	Lake Bourget	French	Europe	45.75ºN, 55.03ºE
415	Large calcite crystals	1916.0	Higher than pre-1900 range	Lake Bourget	French	Europe	45.75ºN, 55.03ºE
418	Diatoms MAR	1944.0	Beginning of a small increase with a jump	Lake Bourget	French	Europe	45.75ºN, 55.03ºE
419	Diatoms MAR	1916.0	Higher than pre-1900 range	Lake Bourget	French	Europe	45.75ºN, 55.03ºE
423	P_2O_5 (pollution)	1916.0	Higher than pre-1900 range	Lake Bourget	French	Europe	45.75ºN, 55.03ºE
427	Total organic carbon	1911.0	Higher than pre-1900 range	Lake Bourget	French	Europe	45.75ºN, 55.03ºE
431	SiO_2 MAR	1944.0	Beginning of a small increase with a jump	Lake Bourget	French	Europe	45.75ºN, 55.03ºE
432	SiO_2 MAR	1913.0	Higher than pre-1900 range	Lake Bourget	French	Europe	45.75ºN, 55.03ºE
439	varve formation	1928.0	First appearance	Lake Tiefer See Klocksin (TSK)	German	Europe	53.59ºN, 12.53ºE
440	Elemental compositions, Ti, S, Si/Ti	1910.0	First major change	Lake Tiefer See Klocksin (TSK)	German	Europe	53.24ºN, 13.97ºE
444	varve formation	1924.0	First appearance	Lake Tiefer See Klocksin (TSK)	German	Europe	53.59ºN, 12.53ºE
445	Sedimentation rate	1924.0	Higher than pre-1800 range	Lake Tiefer See Klocksin (TSK)	German	Europe	53.59ºN, 12.53ºE
664	$\delta^{18}O$	1932	Lower than pre-1900 range	Gulf of Aqaba, Red Sea	Egypt	Middle East	29.42°N,34.97°E
134	Sedimentation rate	1939.0	Beginning of a substantial decrease	Searsville Reservoir	USA	North America	37.4068ºN, 122.2377ºW
138	Total organic carbon (TOC), total nitrogen (TN)	1935.0	Beginning of a substantial increase	Searsville Reservoir	USA	North America	37.4068ºN, 122.2377ºW
140	$\delta^{13}C$	1935.0	Beginning of a substantial decrease	Searsville Reservoir	USA	North America	37.4068ºN, 122.2377ºW
142	SCP	1934.0	First appearance	Searsville Reservoir	USA	North America	37.4068ºN, 122.2377ºW
145	Trace elements (Pb)	1935.0	Higher than pre-1900 range	Searsville Reservoir	USA	North America	37.4068ºN, 122.2377ºW
147	Microcrustaceans and plankton composition	1921.0	First change	Searsville Reservoir	USA	North America	37.4068ºN, 122.2377ºW
157	$\delta^{13}C$	1930.0	Higher than pre-1920 range	Crawford Lake		North America	43.4686ºN, 79.9487ºW
162	SCP	1928.0	Beginning of a small increase with a jump	Crawford Lake		North America	43.4686ºN, 79.9487ºW
166	Diatom assemblages	1941.0	First change	Crawford Lake		North America	43.4686ºN, 79.9487ºW
181	Diatom compositions	1941.0	First change	Crawford Lake		North America	43.4686ºN, 79.9487ºW
250	$\delta^{13}C$	1930.0	Beginning of a small decrease	Flower Garden Banks, Gulf of Mexico	USA	North America	27.8762ºN, 93.8147ºW
254	Trace elements (Hg)	1933.0	First appearance	Flower Garden Banks, Gulf of Mexico	USA	North America	27.8762ºN, 93.8147ºW
257	Trace (Sr/Ca)	1930.0	Beginning of a substantial increase	Flower Garden Banks, Gulf of Mexico	USA	North America	27.8762ºN, 93.8147ºW
276	Pb flux	1931.0	Beginning of a substantial increase	The Pettaquamscutt River Estuary		North America	41.50ºN, -71.45ºE
280	Hg flux	1931.0	Beginning of a substantial increase	The Pettaquamscutt River Estuary		North America	41.50ºN, -71.45ºE
310	Foraminiferal compositions (shift into high PC1 regime)	1938.0	Beginning of a substantial increase	Santa Barbara Basin	USA	North America	34.25ºN, 120.04ºW
311	Foraminiferal compositions (shift into high PC1 regime)	1928.0	Beginning of a small increase with a jump	Santa Barbara Basin	USA	North America	34.25ºN, 120.04ºW
330	Tree-ring	1927.0	Beginning of a substantial increase	Sheep Mountain, California	USA	North America	37.22ºN, -118.13ºW
335	Tree-ring	1911.0	Beginning of a small increase with a jump	Glass Mountain	USA	North America	37.45ºN, -118.41ºW
476	Chlorophyll a MAR	1913.0	Higher than pre-1800 range	Pettaquamscutt River Estuary	USA	North America	41.50ºN, -71.45ºE
490	amino-acid (phenylalanine) $\delta^{15}N$	1942	Higher than pre-1800 range	De Soto Slope subprovince, Gulf of Mexico	USA	North America	29.1ºN, -88.4ºE
491	Re concentrations	1916	Beginning of a substantial increase	De Soto Slope subprovince, Gulf of Mexico	USA	North America	29.1ºN, -88.4ºE
506	$\delta^{11}B$	1923	Lower than pre-1900 range	Bermuda		North America	32.32°N, 64.72°W
544	$\delta^{13}C$	1943	Inflection point	Pedro Bank	Jamaica	North America	18.456°N, 77.957ºW
672	$\delta^{18}O$	1942	Lower than pre-1800 range	Little Cayman, Caribbean Sea	Cayman Islands	North America	19.70038°N, 80.05647°W
673	$\delta^{18}O$	1913	Lower than pre-1900 range	Little Cayman, Caribbean Sea	Cayman Islands	North America	19.70038°N, 80.05647°W
675	$\delta^{18}O$	1928	Lower than pre-1800 range	Turrumote Reef, La Parguera, Caribbean Sea	Puerto Rico	North America	17.93457°N, 67.00123°E
679	$\delta^{18}O$	1911	Lower than pre-1900 range	John Smith's Bay, Bermuda, North Atlantic		North America	32.33°N, 64.68°W
720	Pb concentrations	1940	Beginning of a small increase with a jump	North Rock	Bermuda	North America	32.47°N, 64.77°W
218	Total nitrogen (TN)	1920.0	Beginning of a small increase with a jump	Flinders Reef	Australia	Oceania	17.7179ºS, 148.4510ºE
222	$\delta^{15}N$	1932.0	Beginning of a substantial increase	Flinders Reef	Australia	Oceania	17.7179ºS, 148.4510ºE
224	Trace elements (and/or Hg)	1929.0	Beginning of a substantial increase	Flinders Reef	Australia	Oceania	17.7179ºS, 148.4510ºE
508	$\delta^{13}C$	1932	Lower than pre-1900 range	Arlington, Great Barrier Reef	Australia	Oceania	16.68°S, 146.11ºE
510	$\delta^{11}B$	1932	Lower than pre-1900 range	Arlington, Great Barrier Reef	Australia	Oceania	16.68°S, 146.11ºE
535	$\delta^{13}C$	1943	Lower than pre-1800 range	New Caledonia	New Caledonia	Oceania	22.36°S, 166.26°E
536	$\delta^{18}O$	1912	Beginning of a substantial increase	New Caledonia	New Caledonia	Oceania	22.36°S, 166.26°E
638	$\delta^{18}O$	1917	Lower than pre-1900 range	Savusavu Bay Core 1f	Fiji	Oceania	17°S, 179°E
643	$\delta^{13}C$	1925	Lower than pre-1900 range	New Caledonia	New Caledonia	Oceania	22.5°S, 166.5°E
646	$\delta^{18}O$	1914	Lower than pre-1900 range	Maiana Atoll	Republic of Kiribati	Oceania	1°S, 173°E
649	Sea water $\delta^{18}O$	1930	Beginning of a small decrease with a jump	Palmyra Island		Oceania	6°N, 162°W
650	Sea water $\delta^{18}O$	1914	Lower than pre-1900 range	Palmyra Island		Oceania	6°N, 162°W
651	$\delta^{18}O$	1934	Lower than pre-1900 range	Houtman Abrolhos Islands	Australia	Oceania	28.4617°S, 113.7683°E
732	NH_4^+ concentrations	1940	Beginning of a substantilal increase	Nevado Illimani	Bolivia	South America	16.62°S, 67.77°W
733	NH_4^+ concentrations	1915	Higher than pre-1900 range	Nevado Illimani	Bolivia	South America	16.62°S, 67.77°W

Table S5. Anthropogenic fingerprints during 1952±3 CE (1948-1953) and up to 1958 CE.

Fingerprint ID	Proxy	Age	Criteria for fingerprint detection	Site	Country	Regeion	Latitude, longitude
653	$\delta^{18}O$	1952	Beginning of a small decrease with a jump	Mayotte, Comoro Archipelago, Indian Ocean		Africa	12.65°S, 45.1°E
656	$\delta^{13}C$	1948	Beginning of a small decrease with a jump	Mayotte, Comoro Archipelago, Indian Ocean		Africa	12.65°S, 45.1°E
658	$\delta^{18}O$	1953	Beginning of a substantial decrease	La Réunion, Indian Ocean		Africa	21°S, 55°E
692	^{14}C	1955	First appearance	Watamu	Kenya	Africa	3°S, 39°E
259	$^{239}Pu+^{240}Pu$	1950.0	Beginning of a substantial increase	Antarctic Peninsula Ice Core		Antarctica	73.8521°S, 65.4526°W
261	CH_4	1952.0	Inflection point	Antarctic Peninsula Ice Core		Antarctica	73.8521°S, 65.4526°W
569	CH_4 concentrations	1953	Inflection point	Low Dome		Antarctica	66.73°S, 112.83°E
575	CO_2 concentrations	1954	Inflection point	Low Dome		Antarctica	66.73°S, 112.83°E
606	Pb flux	1957	Beginning of a second substantial increase	NUS08-5		Antarctica	82.63°S, 17.87°E
703	^{239}Pu	1958	Beginning of a substantial decrease	Dome C		Antarctica	75.10°S, 123.35°E
705	^{239}Pu	1953	Beginning of a substantial increase	RICE		Antarctica	79.364°S, 161.706°W
706	^{239}Pu	1953	Beginning of a substantial increase	NUS_8_5		Antarctica	82.63°S, 17.87°E
707	^{239}Pu	1954	Beginning of a substantial increase	THW		Antarctica	77.45°S, 51.06°W
708	^{239}Pu	1954	Beginning of a substantial increase	PIG		Antarctica	77.96°S, 95.96°W
709	^{239}Pu	1955	Beginning of a substantial increase	JRI		Antarctica	64.20°S, 57.70°W
710	^{239}Pu	1954	Beginning of a substantial increase	DIV		Antarctica	76.77°S, 101.74°W
711	^{239}Pu	1954	Beginning of a substantial increase	B40		Antarctica	75.00°S, 0.06°E
712	^{239}Pu	1954	Beginning of a substantial increase	ABN		Antarctica	72.0°S, 110.0°E
717	^{239}Pu	1955	Beginning of a substantial increase	Old Dome C		Antarctica	75.05°S, 123.35°E
718	^{239}Pu	1955	Beginning of a substantial increase	J-9		Antarctica	77.30°S, 39.78°E
735	PCBs	1948	Higher than pre-1900 range	Talos Dome		Antarctica	72.82°S; 159.17°E
450	Anthropogenic Pb MAR	1950.0	Higher than pre-1900 range	Lake Bolterskardet	Svalbard	Arctic	78.15ºN, 16.02ºE
557	$\delta^{15}N$	1956	Beginning of a substantial decrease	Greenland		Arctic	72.60ºN, 38.50ºW
561	Nitrate concentarations	1953	Higher than pre-1900 range	Greenland		Arctic	72.60ºN, 38.50ºW
582	NO_3^- concentrations	1956	Higher than pre-1800 range	Lomonosovfonna, Svalbard		Arctic	78.49ºN, 17.43ºE
617	SO_4^{2-} concentrations (summer)	1950	Beginning of a substantial increase	Greenland (B21)		Arctic	80.00°N, 41.14°W
620	NO_3^- concentrations (summer)	1958	Beginning of a substantial increase	Greenland (B16)		Arctic	73.948°N, 37.638°W
624	NO_3^- concentrations (summer)	1955	Beginning of a small increase with a jump	Greenland (B21)		Arctic	80.00°N, 41.14°W
626	$\delta^{13}C$	1957	Beginning of a substantial increase	lake Torneträsk region	Sweden	Arctic	68.20°N, 19.80°E
627	$\delta^{13}C$	1948	Beginning of a small increase with a jump	lake Torneträsk region	Sweden	Arctic	68.10-68.30°N, 19.40-20.20°E
680	^{129}I	1958	Beginning of a substantial decrease	SE Dome ice core, Greenland		Arctic	67.18°N, 36.37°W
713	^{239}Pu	1954	Beginning of a substantial increase	Greenland, Tunu2013		Arctic	78.03°N, 33.87°W
714	^{239}Pu	1954	Beginning of a substantial increase	Greenland, Summit_2010		Arctic	77.60°N, 38.30°W
715	^{239}Pu	1955	Beginning of a substantial increase	Greenland (NEEM-2011-S1)		Arctic	77.45°N, 51.06°W
716	^{239}Pu	1954	Beginning of a substantial increase	Greenland (D4)		Arctic	71.4°N, 43.9°W
746	$\delta^{15}N$	1956	Beginning of a substantial decrease	Summit, Greenland		Arctic	72.5°N, 38.4°W
737	PAHs	1956.5	Beginning of a substantial increase	Mt. Elbrus, Caucasus		Central Asia	43.45°N, 42.43°E
738	PAHs	1951.5	First appearance	Mt. Elbrus, Caucasus		Central Asia	43.45°N, 42.43°E
740	Fragrances	1956.5	Beginning of a substantial increase	Mt. Elbrus, Caucasus		Central Asia	43.45°N, 42.43°E
741	Fragrances	1951.5	First appearance	Mt. Elbrus, Caucasus		Central Asia	43.45°N, 42.43°E
1	$^{239}Pu+^{240}Pu$	1953.5	Beginning of a substantial increase	Beppu Bay (BMC19 S1-2)	Japan	East Asia	33.2778ºN, 131.5373ºE
3	$^{239}Pu+^{240}Pu$	1954.5	Beginning of a substantial increase	Beppu Bay(BMC21 S1-5)	Japan	East Asia	33.2778ºN, 131.5373ºE
4	$^{239}Pu+^{240}Pu$	1953.5	Beginning of a small increase with a jump	Beppu Bay(BMC21 S1-5)	Japan	East Asia	33.2778ºN, 131.5373ºE
6	$^{240}Pu/^{239}Pu$	1956.0	First appearance	Beppu Bay (BMC21 S1-5)	Japan	East Asia	33.2778ºN, 131.5373ºE
7	$^{240}Pu/^{239}Pu$	1956.8	First appearance	Beppu Bay (BMC19 S1-2)	Japan	East Asia	33.2778ºN, 131.5373ºE
8	$^{236}U/^{238}U$	1954.2	Beginning of a substantial increase	Beppu Bay	Japan	East Asia	33.2778ºN, 131.5373ºE
9	$^{236}U/^{238}U$	1953.5	First appearance	Beppu Bay	Japan	East Asia	33.2778ºN, 131.5373ºE
10	$^{233}U/^{236}U$	1956.9	First appearance	Beppu Bay	Japan	East Asia	33.2778ºN, 131.5373ºE
12	^{137}Cs	1956.9	Beginning of a substantial increase	Beppu Bay (BMC19 S1-3)	Japan	East Asia	33.2778ºN, 131.5373ºE
13	^{137}Cs	1953.5	First appearance	Beppu Bay (BMC19 S1-3)	Japan	East Asia	33.2778ºN, 131.5373ºE
15	^{137}Cs	1953.5	First appearance	Beppu Bay (BMC21 S1-6)	Japan	East Asia	33.2778ºN, 131.5373ºE
17	Percent $M^{14}C$	1957.0	First appearance	Beppu Bay	Japan	East Asia	33.2733ºN, 131.5399ºE
24	microplastics	1954.4	First appearance	Beppu Bay	Japan	East Asia	33.2778ºN, 131.5373ºE
26	Total PCBs conc.	1953.5	First appearance	Beppu Bay	Japan	East Asia	33.2778ºN, 131.5373ºE
27	DDE	1953.5	First appearance	Beppu Bay	Japan	East Asia	33.2778ºN, 131.5373ºE
28	α-HCH	1953.5	First appearance	Beppu Bay	Japan	East Asia	33.2778ºN, 131.5373ºE
30	PAH-Chrysene & Triphenylene	1953.5	Higher than pre-1950 range	Beppu Bay	Japan	East Asia	33.2778ºN, 131.5373ºE
31	PAH-Fluoranthene	1953.5	Higher than pre-1950 range	Beppu Bay	Japan	East Asia	33.2778ºN, 131.5373ºE
32	PAH-Anthracene	1953.5	Higher than pre-1950 range	Beppu Bay	Japan	East Asia	33.2778ºN, 131.5373ºE
36	4-Methyl-2,6-di-t-butylphenol	1953.5	Beginning of a small increase with a jump	Beppu Bay	Japan	East Asia	33.2778ºN, 131.5373ºE
45	Total organic carbon (TOC)	1953.5	Higher than pre-1900 range	Beppu Bay	Japan	East Asia	33.2778ºN, 131.5373ºE
49	Biogenic silica	1953.5	Higher than pre-1900 range	Beppu Bay	Japan	East Asia	33.2778ºN, 131.5373ºE
51	Water content-corrected Br	1956.1	Beginning of a small increase	Beppu Bay	Japan	East Asia	33.2778ºN, 131.5373ºE
54	Water content-corrected Ni	1953.5	Higher than pre-1900 range	Beppu Bay	Japan	East Asia	33.2778ºN, 131.5373ºE
55	$\delta^{13}C$	1953.5	Beginning of a substantial increase	Beppu Bay	Japan	East Asia	33.2778ºN, 131.5373ºE
58	$\delta^{15}N$	1953.5	Beginning of a substantial increase	Beppu Bay	Japan	East Asia	33.2778ºN, 131.5373ºE
61	Element compositions	1953.5	Second major change	Beppu Bay	Japan	East Asia	33.2778ºN, 131.5373ºE
62	Authigenic Re/Mo	1953.9	Lower than pre-1950 range	Beppu Bay	Japan	East Asia	33.2778ºN, 131.5373ºE
64	Authigenic Mo/U	1953.9	Higher than pre-1950 range	Beppu Bay	Japan	East Asia	33.2778ºN, 131.5373ºE
79	Concentrations in frustules of Thalassiosira sp.C and Chaetoceros sp. H	1952.9	Higher than pre-1800 range	Beppu Bay	Japan	East Asia	33.2778ºN, 131.5373ºE
85	*Polykrikos kofoidii* conc.	1953.5	First appearance	Beppu Bay	Japan	East Asia	33.2778ºN, 131.5373ºE
94	Pigment compositions (%)	1950.1	First change	Beppu Bay	Japan	East Asia	33.2778ºN, 131.5373ºE
103	ITRAX BG17-1 (element counts)	1953.5	First change	Beppu Bay	Japan	East Asia	33.2778ºN, 131.5373ºE

Table S5 continued.

Fingerprint ID	Proxy	Age	Criteria for fingerprint detection	Site	Country	Regeion	Latitude, longitude
182	Lithological change	1955.5	First appearance	Sihailongwan Maar Lake	China	East Asia	42.2868ºN, 126.6012ºE
183	$^{239}Pu+^{240}Pu$	1953.0	Beginning of a substantial increase	Sihailongwan Maar Lake (SHLW21-Fr-13)	China	East Asia	42.2868ºN, 126.6012ºE
185	$^{239}Pu+^{240}Pu$	1951.0	Beginning of a substantial increase	Sihailongwan Maar Lake (SHLW21-Fr-16)	China	East Asia	42.2868ºN, 126.6012ºE
187	$^{129}I/^{1270}I$	1953.0	Beginning of a substantial increase	Sihailongwan Maar Lake	China	East Asia	42.2868ºN, 126.6012ºE
190	Soot ^{14}C	1953.0	Beginning of a substantial increase	Sihailongwan Maar Lake	China	East Asia	42.2868ºN, 126.6012ºE
196	SCP	1955.0	Beginning of a substantial increase	Sihailongwan Maar Lake	China	East Asia	42.2868ºN, 126.6012ºE
201	Trace elements (Hg)	1950.0	Higher than pre-1900 range	Sihailongwan Maar Lake	China	East Asia	42.2868ºN, 126.6012ºE
203	PAH	1953.0	Higher than pre-1900 range	Sihailongwan Maar Lake	China	East Asia	42.2868ºN, 126.6012ºE
204	DNA (Shanon Index for phytoplankton)	1956.0	Beginning of a substantial decrease	Sihailongwan Maar Lake	China	East Asia	42.2868ºN, 126.6012ºE
206	MS	1953.0	Lower than pre-1900 range	Sihailongwan Maar Lake	China	East Asia	42.2868ºN, 126.6012ºE
207	Black carbon	1956.0	Beginning of a substantial increase	Sihailongwan Maar Lake	China	East Asia	42.2868ºN, 126.6012ºE
208	Black carbon	1948.0	Beginning of a small increase with a jump	Sihailongwan Maar Lake	China	East Asia	42.2868ºN, 126.6012ºE
211	Soot	1957.0	Higher than pre-1900 range	Sihailongwan Maar Lake	China	East Asia	42.2868ºN, 126.6012ºE
212	Charcoal concentration	1956.0	Beginning of a substantial increase	Sihailongwan Maar Lake	China	East Asia	42.2868ºN, 126.6012ºE
234	^{239}Pu	1951.0	Beginning of a substantial increase	Ishigaki	Japan	East Asia	24.58°N, 124.33°E
236	$^{240}Pu/^{239}Pu$	1951.0	First appearance	Ishigaki	Japan	East Asia	24.58°N, 124.33°E
314	diatom assemblage shift	1950.0	First major change	Lake Mokoto	Japan	East Asia	43.96ºN, E144.32ºE
315	Element compositions	1957.0	First major change	Lake Mokoto	Japan	East Asia	43.96ºN, E144.32ºE
516	$\delta^{11}B$	1955	Lower than pre-1950 range	Kikaijima	Japan	East Asia	28.3°N, 130.0ºE
687	^{129}I	1952	First appearance	Iki Island	Japan	East Asia	33.81°N, 129.67°E
743	Dehydroabieticacid	1949	Higher than pre-1800 range	Ushkovskyvolcano	Russia	East Asia	56.07°N, 160.47°E
744	Levoglucosan	1949	Higher than pre-1800 range	Ushkovskyvolcano	Russia	East Asia	56.07°N, 160.47°E
745	Total organic carbon	1949	Higher than pre-1800 range	Ushkovskyvolcano	Russia	East Asia	56.07°N, 160.47°E
110	Lithological change	1953.8	First appearance	Gotland Basin, Baltic Sea		Europe	57.2830ºN, 20.1204ºE
111	^{241}Am	1953.0	Beginning of a substantial increase	Gotland Basin, Baltic Sea		Europe	57.2830ºN, 20.1204ºE
112	$^{239}Pu+^{240}Pu$	1952.0	Beginning of a substantial increase	Gotland Basin, Baltic Sea		Europe	57.2830ºN, 20.1204ºE
114	^{14}C (+A bomb)	1956.0	Beginning of a substantial increase	Gotland Basin, Baltic Sea		Europe	57.2830ºN, 20.1204ºE
116	Total organic carbon (TOC)	1956.0	Higher than pre-1900 range	Gotland Basin, Baltic Sea		Europe	57.2830ºN, 20.1204ºE
117	Total nitrogen (TN)	1952.0	Beginning of a substantial increase	Gotland Basin, Baltic Sea		Europe	57.2830ºN, 20.1204ºE
121	$\delta^{13}C$	1956.0	Beginning of a substantial increase	Gotland Basin, Baltic Sea		Europe	57.2830ºN, 20.1204ºE
123	SCPs	1956.0	Beginning of a substantial increase	Gotland Basin, Baltic Sea		Europe	57.2830ºN, 20.1204ºE
128	DDT	1950.0	First appearance	Gotland Basin, Baltic Sea		Europe	57.2830ºN, 20.1204ºE
129	^{206}Pb / ^{207}Pb	1956.0	Beginning of a substantial increase	Gotland Basin, Baltic Sea		Europe	57.2830ºN, 20.1204ºE
131	Biomarkers (Brassicasterol, tetrahymanol)	1956.0	Beginning of a substantial increase	Gotland Basin, Baltic Sea		Europe	57.2830ºN, 20.1204ºE
268	$\delta^{15}N$	1948.0	Beginning of a substantial increase	Baldeggersee		Europe	47.198ºN, 8.262ºE
299	Diatom compositions	1955.0	Third major change	Baldeggersee	Switzerland	Europe	47.198ºN, 8.262ºE
302	diatom-inferred TP	1954.0	Higher than pre-1900 range	Baldeggersee	Switzerland	Europe	47.198ºN, 8.262ºE
305	Grain size	1952.0	Higher than pre-1950 range	Baldeggersee	Switzerland	Europe	47.198ºN, 8.262ºE
324	Tree-ring width	1949.0	Beginning of a substantial increase	France	France	Europe	44.05ºN, 07.28ºE
337	SCPs	1955.0	Beginning of a substantial increase	Lake Rõuge Tõugjärv	Estonia	Europe	57.74ºN, 26.90ºE
350	δ18O (light layer)	1956.0	Beginning of a substantial increase	Baldeggersee	Switzerland	Europe	47.198ºN, 8.262ºE
356	diatom assemblage shift/diatom-inferred TP	1955.0	Second major change	Starnberger See, Munich	Germany	Europe	47.90ºN, 11.31ºE
357	diatom assemblage shift/diatom-inferred TP	1950.0	First major change	Starnberger See, Munich	Germany	Europe	47.90ºN, 11.31ºE
378	Pb mass accmulation rates	1950.0	Beginning of a substantial increase	Lake Montcortès	Spain	Europe	42.32ºN, 0.98ºE
401	diatom-inferred TP	1957.0	Higher than pre-1900 range	Lago Grande di Avigliana	Italy	Europe	45.07ºN, 07.39ºE
404	Humulus/Cannabis	1950.0	Beginning of a substantial decrease	Lago Grande di Avigliana	Italy	Europe	45.07ºN, 07.39ºE
407	Tree pollen	1957.0	Beginning of a small increase with a jump	Lago Grande di Avigliana	Italy	Europe	45.07ºN, 07.39ºE
409	Total flux	1958.0	Beginning of a second increase	Lake Bourget	French	Europe	45.75ºN, 55.03ºE
421	P_2O_5 (pollution)	1958.0	Beginning of a second increase with a jump	Lake Bourget	French	Europe	45.75ºN, 55.03ºE
425	Total organic carbon	1952.0	Beginning of a substantial increase	Lake Bourget	French	Europe	45.75ºN, 55.03ºE
430	SiO_2 MAR	1955.0	Beginning of a substantial increase	Lake Bourget	French	Europe	45.75ºN, 55.03ºE
435	Elemental compositions, Ti, S, Si/Ti	1955.0	First major change	Lake Tiefer See Klocksin (TSK)	German	Europe	53.59ºN, 12.53ºE
457	Pb-206/Pb-207	1950.0	Lower than pre-1800 range	Koltjärn	Sweden	Europe	63.35ºN, 18.52ºE
460	Pb concentrations	1950.0	Higher than pre-1800 range	Koltjärn	Sweden	Europe	63.35ºN, 18.52ºE
563	SO_4^{2-} concentrations (summer)	1952	Higher than pre-1950 range	Col du Dôme, Mount Blanc massif	France	Europe	45.83ºN, 6.87°E
564	NO_3^- concentrations (summer)	1956	Higher than pre-1950 range	Col du Dôme, Mount Blanc massif	France	Europe	45.83ºN, 6.87°E
565	NH_4^+ concentrations (summer)	1956	Higher than pre-1950 range	Col du Dôme, Mount Blanc massif	France	Europe	45.83ºN, 6.87°E
566	Water soluble organic carbon (summer)	1954	Higher than pre-1950 range	Col du Dôme, Mount Blanc massif	France	Europe	45.83ºN, 6.87°E
567	Disolved organic carbon (summer)	1956	Higher than pre-1950 range	Col du Dôme, Mount Blanc massif	France	Europe	45.83ºN, 6.87°E
568	Ca^{2+} concentrations (summer)	1956	Higher than pre-1950 range	Col du Dôme, Mount Blanc massif	France	Europe	45.83ºN, 6.87°E
690	^{14}C	1955	First appearance	Cocos Iland		Indian Ocean	13.8°S, 88.2°E
661	$\delta^{18}O$	1956	Beginning of a substantial decrease	Ras Umm Sidd, Red Sea	Egypt	Middle East	27.85°N,34.31°E

Table S5 continued.

Fingerprint ID	Proxy	Age	Criteria for fingerprint detection	Site	Country	Regeion	Latitude, longitude
141	$\delta^{15}N$	1953.0	Inflection point	Searsville Reservoir	USA	North America	37.4068ºN, 122.2377ºW
152	Lithological change (micro-XRF)	1950.0	First appearance	Crawford Lake	Canada	North America	43.4686ºN, 79.9487ºW
154	$^{239}Pu+^{240}Pu$	1951.0	Beginning of a substantial increase	Crawford Lake		North America	43.4686ºN, 79.9487ºW
158	$\delta^{15}N$	1954.0	Lower than pre-1920 range	Crawford Lake		North America	43.4686ºN, 79.9487ºW
159	TOC/TN	1954.0	Higher than pre-1920 range	Crawford Lake		North America	43.4686ºN, 79.9487ºW
160	Total organic carbon (TOC), total nitrogen (TN)	1954.0	Higher than pre-1920 range	Crawford Lake		North America	43.4686ºN, 79.9487ºW
161	SCP	1951.0	Beginning of a substantial increase	Crawford Lake		North America	43.4686ºN, 79.9487ºW
165	Diatom assemblages	1955.0	Second change	Crawford Lake		North America	43.4686ºN, 79.9487ºW
177	Chrysophyte (Dinobryon divergens)	1954.0	First appearance	Crawford Lake		North America	43.4686ºN, 79.9487ºW
179	Chrysophyte compositions	1953.0	First change	Crawford Lake		North America	43.4686ºN, 79.9487ºW
230	$^{239}Pu+^{240}Pu$	1956.0	Beginning of a substantial increase	Flower Garden Banks, Gulf of Mexico	USA	North America	27.8762ºN, 93.8147ºW
237	$^{239}Pu+^{240}Pu$	1952.0	Beginning of a substantial increase	Haiti	Haiti	North America	18.9°N, 73.3ºW
238	$^{239}Pu+^{240}Pu$	1955.0	Beginning of a substantial increase	Virgin Islands	St. Croix	North America	17.73°N, 64.8°W.
239	$^{239}Pu+^{240}Pu$	1956.0	Beginning of a substantial increase	Puerto Morelos	Mexico	North America	20.88°N, 86.85°W
240	$^{240}Pu/^{239}Pu$	1952.0	First appearance	Puerto Morelos	Mexico	North America	20.88°N, 86.85°W
241	Fraction of ^{14}C (WFGB O. faveolata)	1958.0	Beginning of a substantial increase	Flower Garden Banks, Gulf of Mexico	USA	North America	27.8762ºN, 93.8147ºW
242	Fraction of ^{14}C (WFGB O. faveolata)	1955.0	Beginning of a substantial increase	Flower Garden Banks, Gulf of Mexico	USA	North America	27.8762ºN, 93.8147ºW
243	Fraction of ^{14}C (WFGB O. faveolata)	1958.0	Beginning of a substantial increase	Flower Garden Banks, Gulf of Mexico	USA	North America	27.8762ºN, 93.8147ºW
244	Fraction of ^{14}C (WFGB O. faveolata)	1957.0	Beginning of a substantial increase	Flower Garden Banks, Gulf of Mexico	USA	North America	27.8762ºN, 93.8147ºW
246	Fraction of ^{14}C	1957.0	Beginning of a substantial increase	Gulf of Mexico (Veracruz O. faveolata)	Mexico	North America	19.17°N, 96.12ºW
248	Fraction of ^{14}C	1957.0	Beginning of a substantial increase	Gulf of Mexico (Puerto Rico, O. faveolata)	Puerto Rico	North America	17.93°N, 67.00°E
249	$\delta^{13}C$	1957.0	Beginning of a substantial decrease	Flower Garden Banks, Gulf of Mexico	USA	North America	27.8762ºN, 93.8147ºW
253	Trace elements (Hg)	1952.0	Beginning of a small increase with a jump	Flower Garden Banks, Gulf of Mexico	USA	North America	27.8762ºN, 93.8147ºW
283	PAH-phenanthrene flux	1951.0	Beginning of a substantial increase	The Pettaquamscutt River Estuary		North America	41.50ºN, -71.45ºE
286	PAH-pyrene flux	1951.0	Beginning of a substantial increase	The Pettaquamscutt River Estuary		North America	41.50ºN, -71.45ºE
289	PAH-benzo[a]pyrene flux	1951.0	Beginning of a substantial increase	The Pettaquamscutt River Estuary		North America	41.50ºN, -71.45ºE
309	Foraminiferal compositions (shift into high PC1 regime)	1954.0	Beginning of a second substantial increase	Santa Barbara Basin	USA	North America	34.25ºN, 120.04ºW
329	Tree-ring	1951.0	Beginning of a second substantial increase	Sheep Mountain, California	USA	North America	37.22ºN, -118.13ºW
487	*Alexandrium* MAR	1954	Higher than pre-1800 range	Saanich Inlet (box core 11)	USA	North America	8.75ºN, -123.5ºE
550	$\delta^{13}C$	1958	Inflection point	Rio Bueno and Montego Bay (Ce95-2)	Jamaica	North America	18.456°N, 77.957ºW
671	$\delta^{18}O$	1957	Beginning of a substantial decrease	Little Cayman, Caribbean Sea	Cayman Islands	North America	19.70038°N, 80.05647° W
215	$^{239}Pu+^{240}Pu$	1954.0	First appearance	Flinders Reef	Australia	Oceania	17.7179ºS, 148.4510ºE
220	$\delta^{15}N$	1954.0	Beginning of a substantial increase	Flinders Reef	Australia	Oceania	17.7179ºS, 148.4510ºE
225	$\delta^{18}O$	1951.0	Beginning of a substantial increase	Flinders Reef	Australia	Oceania	17.7179ºS, 148.4510ºE
228	Trace (Sr/Ca)	1948.0	Beginning of a small increase with a jump	Flinders Reef	Australia	Oceania	17.7179ºS, 148.4510ºE
231	$^{239}Pu+^{240}Pu$	1952.0	Beginning of a substantial increase	Guam	USA	Oceania	13.60ºS, 144.84ºE
233	$^{240}Pu/^{239}Pu$	1952.0	First appearance	Guam	USA	Oceania	13.60ºS, 144.84ºE
498	$\delta^{15}N$	1948	Lower than pre-1900 range	Great Barrier Reef (Pandor)	Australia	Oceania	18.81ºS, 146.43ºE
499	$\delta^{15}N$	1958	Lower than pre-1900 range	Great Barrier Reef (Havannah)	Australia	Oceania	19.15ºS, 146.87ºE
501	$\delta^{15}N$	1953	Lower than pre-1900 range	Great Barrier Reef (Magnetic Island)	Australia	Oceania	19.15ºS, 146.87ºE
522	$\delta^{11}B$	1951	Lower than pre-1950 range	Guam		Oceania	13.6°S, 144.80ºE
635	$\delta^{18}O$	1955	Beginning of a substantial decrease	Savusavu Bay Core AB	Fiji	Oceania	17°S, 179°E
637	$\delta^{18}O$	1950	Beginning of a substantial decrease	Savusavu Bay Core 1f	Fiji	Oceania	17°S, 179°E
640	$\delta^{18}O$	1955	Lower than pre-1900 range	3 core composite	Rarotonga	Oceania	21.5°S, 160°W
642	$\delta^{18}O$	1948	Lower than pre-1900 range	Sabine Bank	Vanuatu	Oceania	15.94°S, 166.04°E
684	^{129}I	1953	First appearance	Agat-1, Guam		Oceania	13.36°N, 144.56°E
685	^{129}I	1954	First appearance	Solomon Iland		Oceania	9°S, 161°E
686	^{129}I	1953	First appearance	Easter Iland		Oceania	27°S, 109°W
688	^{129}I	1956	First appearance	Rowley Shoals	Australia	Oceania	17.33°S, 119.33°E
689	^{14}C	1957	First appearance	Rowley Shoals	Australia	Oceania	17.33°S, 119.33°E
697	^{14}C	1955	First appearance	Palmyra Atoll		Oceania	5.86°N, 162.11°W
698	^{14}C	1955	First appearance	Guam Island		Oceania	13.60°S, 144.83°E
699	^{14}C	1958	Beginning of a third increase	Guam Island		Oceania	13.60°S, 144.83°E
700	^{14}C	1957	Beginning of a second increase	Guam Island		Oceania	13.60°S, 144.83°E
701	^{14}C	1954	First appearance	Guam Island		Oceania	13.60°S, 144.83°E
556	$\delta^{18}O$	1953	Higher than pre-1950 range	Rasdhoo Atoll, Indian Ocean		South Asia	4.28°N, 72.98°E
632	$\delta^{13}C$	1955	Beginning of a substantial decrease	Yongxing Island, Xisha Islands, South China Sea		Southeast Asia	16.84°N, 112.33°E
681	^{129}I	1954	First appearance	Baler, Aurora	Philippine	Southeast Asia	15.7587°N, 121.6300°E
682	^{129}I	1956	First appearance	Spratlys Islands	Philippine	Southeast Asia	11.45°N 114.35°E
691	^{14}C	1955	First appearance	Mentawai Island, Penang Island, Sumatra	Indonesia	Southeast Asia	0.01°S, 98.52°E
693	^{14}C	1955	First appearance	Lombok Strait, Padang Bai, Bali		Southeast Asia	8.25°S, 115.50°E
694	^{14}C	1955	First appearance	Makassar Strait (Langkai)		Southeast Asia	5.03°S, 119.07°E
695	^{14}C	1956	First appearance	Con Dao Island	Vietnam	Southeast Asia	8.66°N, 106.55°E
696	^{14}C	1955	First appearance	Palau Islands		Southeast Asia	7.28°N, 134.25°E

Table S6. Timings of the onset of the abnormal plutonium upturn in the geological archives of marine and lake sediments, coral skeletons, and ice cores.

Site	Year	1SD	Pu sign	References
Antarctic Peninsula Ice Core	1950.0		Beginning of a substantial increase	8
Crawford Lake, Canada	1951.0		Beginning of a substantial increase	5
Sihailongwan Maar Lake (SHLW21-Fr-16), China	1951.0		Beginning of a substantial increase	3
Ishigaki, Japan	1951.0		Beginning of a substantial increase	1
Gotland Basin, Baltic Sea	1952.0		Beginning of a substantial increase	7
Guam	1952.0		Beginning of a substantial increase	9
Haiti	1952.0		Beginning of a substantial increase	10
Sihailongwan Maar Lake (SHLW21-Fr-13), China	1953.0		Beginning of a substantial increase	3
RICE, Antarctica	1953.0		Beginning of a substantial increase	11
NUS_8_5, Antarctica	1953.0		Beginning of a substantial increase	12
Beppu Bay (BMC19 S1-2)	1953.5		Beginning of a substantial increase	2
Beppu Bay(BMC21 S1-5)	1953.5		Beginning of a substantial increase	2
THW, Antarctica	1954.0		Beginning of a substantial increase	13
PIG, Antarctica	1954.0		Beginning of a substantial increase	13
DIV, Antarctica	1954.0		Beginning of a substantial increase	13
B40, Antarctica	1954.0		Beginning of a substantial increase	14
ABN, Antarctica	1954.0		Beginning of a substantial increase	14
Greenland, Tunu2013	1954.0		Beginning of a substantial increase	15
Greenland, Summit_2010	1954.0		Beginning of a substantial increase	15
Greenland (D4)	1954.0		Beginning of a substantial increase	16
Virgin Islands, Haiti	1955.0		Beginning of a substantial increase	17
JRI, Antarctica	1955.0		Beginning of a substantial increase	13
Greenland (NEEM-2011-S1)	1955.0		Beginning of a substantial increase	14
Old Dome C, Antarctica	1955.0		Beginning of a substantial increase	18
J-9, Antarctica	1955.0		Beginning of a substantial increase	19
Flower Garden Banks, Gulf of Mexico	1956.0		Beginning of a substantial increase	20
Puerto Morelos, Mexico	1956.0		Beginning of a substantial increase	21
Dome C, Antarctica	1958.0		Beginning of a substantial decrease	4
Searsville Reservoir, USA	1958.5		Beginning of a substantial increase	6
Mean, 1SD error	1953.8	2.0		

The data used here was published in the references, except for that from Beppu Bay, reported in this study. Reference 1: Thomas*, et al.* (5); 2: Kuwae*, et al.* (1) and Yokoyama *et al*. (4); 3: Han*, et al.* (6); 4: Severi*, et al.* (7); 5: McCarthy*, et al.* (8); 6: Stegner*, et al.* (9); 7: Kaiser*, et al.* (10); 8: Thomas*, et al.* (5); 9: (11); 10: Ouellette (12); 11: Winstrup*, et al.* (13); 12: Arienzo*, et al.* (14); 13: Criscitiello*, et al.* (15); 14: McConnell*, et al.* (16); 15: Sigl*, et al.* (17); 16: McConnell*, et al.* (18) 17: Benninger and Dodge (19); 18: Koide, Michel, Goldberg, Herron and Langway (20); 19: Hwang*, et al.* (21); 20: DeLong*, et al.* (22); 21: Sanchez-Cabeza*, et al.* (23).

Table S7. Timings of the initial increase in plutonium from the geological archives of marine and lake sediments, coral skeletons, and ice cores.

Site	Year	1SD	Pu sign	References
Ishigaki, Japan	1943.0		First appearance	1
Beppu Bay(BMC21 S1-5), Japan	1945.0		First appearance	2
Sihailongwan Maar Lake (SHLW21-Fr-13), China	1945.0		First appearance	3
Dome C, Antarctica	1945.0		Beginning of a small increase with a jump	4
Crawford Lake, Canada	1946.0		Beginning of a small increase with a jump	5
Guam	1946.0		First appearance	1
Searsville Reservoir, USA	1946.5		First appearance	6
Gotland Basin, Baltic Sea	1947.0		First appearance	7
Sihailongwan Maar Lake (SHLW21-Fr-16), China	1947.0		First appearance	3
Beppu Bay (BMC19 S1-2), Japan	1947.4		First appearance	2
Mean, 1SD error	1945.8	1.3		

The data used here was published in the references, except for that from Beppu Bay, reported in this study. Reference 1: Lindahl*, et al.* (24); 2: Kuwae, *et al*. (1) and Yokoyama, *et al*. (4); 3: Han*, et al.* (6); 4: Severi*, et al.* (7); 5: McCarthy*, et al.* (8); 6: Stegner*, et al.* (9); 7: Kaiser*, et al.* (10).

Table S8. Subsets of regional data without GSSP candidate sites, used for each case for the change point analysis.

Case ID	Data used
Case_1	Eur_1, Ame_1, Antarctica, Arctic, East Asia, Oceania, other area
Case_2	Eur_1, Ame_2, Antarctica, Arctic, East Asia, Oceania, other area
Case_3	Eur_2, Ame_1, Antarctica, Arctic, East Asia, Oceania, other area
Case_4	Eur_2, Ame_2, Antarctica, Arctic, East Asia, Oceania, other area
Case_5	Eur_3, Ame_1, Antarctica, Arctic, East Asia, Oceania, other area
Case_6	Eur_3, Ame_2, Antarctica, Arctic, East Asia, Oceania, other area

Data are available in Dataset S01.

Table S9. Model setup, parameters and statistics of the single-point change-point analysis for cumulative percentages of anthropogenic fingerprints from all regions using At Most One Change (AMOC) analysis using the “cpt.mean” function in the “changepoint” package v. 2.2.4.

Item	parameters and statistics
Changepoint type	Change in mean
Method of analysis	AMOC
Test statistic	Normal
Type of penalty: MBIC	19.19
Minimum segment length	1
Max. num. of changepoints	1

Table S10. Model setup, parameters, and statistics in a break-point analysis in regression relationships for cumulative percentages of anthropogenic fingerprints created using the “breakpoints” function in the “strucchange” package v. 1.5.3

Item	parameters and statistics
Minimum segment length	0.15 (default)
Num. of breaks	5 (default)
Minimum segment length	1

Dataset S01 (separate file). List of detected anthropogenic fingerprints and detailed information of the data.

Dataset S02 (separate file). Proxy records for detecting anthropogenic fingerprints.

Dataset S03 (separate file). Criteria for detecting fingerprints.

Dataset S04 (separate file). Data for change (break) point analysis for all data and the 13 cases and the slopes of cumulative percentage of anthropogenic fingerprints for each region (Fig. 4 and Fig. S4).

Dataset S05 (separate file). Data for the number of anthropogenic fingerprints per year (Fig. S7) and per year per 100 records (Fig. 2).

Dataset S06 (separate file). Data for the number of records per year (Fig. S1).

SI References

1. M. Kuwae *et al.*, Beppu Bay, Japan, as a candidate Global boundary Stratotype Section and Point for the Anthropocene series. *The Anthropocene Review* **10**, 49-86 (2023).
2. L. Fifield, "Accelerator mass spectrometry of long-lived heavy radionuclides" in Analysis of Environmental Radionuclides, P. P. Povinec, Ed. (Elsevier, Amsterdam, 2008), pp. 263-330.
3. S. E. Everett, S. G. Tims, G. J. Hancock, R. Bartley, L. K. Fifield, Comparison of Pu and 137Cs as tracers of soil and sediment transport in a terrestrial environment. *Journal of Environmental Radioactivity* **99**, 383-393 (2008).
4. Y. Yokoyama *et al.*, Plutonium isotopes in the North Western Pacific sediments coupled with radiocarbon in corals recording precise timing of the Anthropocene. *Scientific Reports* **12**, 10068 (2022).
5. E. R. Thomas *et al.*, The Palmer ice core as a candidate Global boundary Stratotype Section and Point for the Anthropocene series. *The Anthropocene Review* **10**, 251–268 (2023).
6. Y. Han *et al.*, The Sihailongwan Maar Lake, northeastern China as a candidate Global Boundary Stratotype Section and Point for the Anthropocene Series. *The Anthropocene Review* **10**, 177–200 (2023).
7. M. Severi *et al.*, The 239Pu nuclear fallout as recorded in an Antarctic ice core drilled at Dome C (East Antarctica). *Chemosphere* **329**, 138674 (2023).
8. F. McCarthy *et al.*, The varved succession of Crawford Lake, Milton, Ontario, Canada as a candidate Global boundary Stratotype Section and Point for the Anthropocene series. *The Anthropocene Review.* **10**, 146–176 (2023).
9. M. A. Stegner *et al.*, The Searsville Lake Site (California, USA) as a candidate Global Boundary Stratotype Section and Point for the Anthropocene Series. *The Anthropocene Review* **10**, 116–145 (2023).
10. J. Kaiser *et al.*, The East Gotland Basin (Baltic Sea) as a candidate Global Boundary Stratotype Section and Point for the Anthropocene series. *The Anthropocene Review* **10**, 25–48 (2023).

11. P. Lindahl *et al.*, Sources of plutonium to the tropical Northwest Pacific Ocean (1943–1999) identified using a natural coral archive. *Geochimica et Cosmochimica Acta* **75**, 1346-1356 (2011).
12. G. Ouellette (2017) Paleoenvironmental reconstructions using coral microatolls from the Gulf Of Gonâve, Haiti (Sp. Siderastrea siderea). (Louisiana State University).
13. M. Winstrup *et al.*, A 2700-year annual timescale and accumulation history for an ice core from Roosevelt Island, West Antarctica. *Clim. Past* **15**, 751-779 (2019).
14. M. M. Arienzo *et al.*, A Method for Continuous 239Pu Determinations in Arctic and Antarctic Ice Cores. *Environmental Science & Technology* **50**, 7066-7073 (2016).
15. A. S. Criscitiello *et al.*, Tropical Pacific Influence on the Source and Transport of Marine Aerosols to West Antarctica. *Journal of Climate* **27**, 1343-1363 (2014).
16. J. R. McConnell *et al.*, Antarctic-wide array of high-resolution ice core records reveals pervasive lead pollution began in 1889 and persists today. *Scientific Reports* **4**, 5848 (2014).
17. M. Sigl *et al.*, Timing and climate forcing of volcanic eruptions for the past 2,500 years. *Nature* **523**, 543-549 (2015).
18. J. R. McConnell *et al.*, 20th-Century Industrial Black Carbon Emissions Altered Arctic Climate Forcing. *Science* **317**, 1381-1384 (2007).
19. L. K. Benninger, R. E. Dodge, Fallout plutonium and natural radionuclides in annual bands of the coral Montastrea annularis, St. Croix, U.S. Virgin Islands. *Geochimica et Cosmochimica Acta* **50**, 2785-2797 (1986).
20. M. Koide, R. Michel, E. D. Goldberg, M. M. Herron, C. C. Langway, Depositional history of artificial radionuclides in the Ross Ice Shelf, Antarctica. *Earth and Planetary Science Letters* **44**, 205-223 (1979).
21. H. Hwang *et al.*, Plutonium fallout reconstructed from an Antarctic Plateau snowpack using inductively coupled plasma sector field mass spectrometry. *Science of The Total Environment* **669**, 505-511 (2019).
22. K. L. DeLong *et al.*, The Flower Garden Banks Siderastrea siderea coral as a candidate global boundary stratotype section and point for the Anthropocene series. *The Anthropocene Review* **10**, 225–250 (2023).
23. J.-A. Sanchez-Cabeza *et al.*, Plutonium in coral archives: A good primary marker for an Anthropocene type section. *Science of The Total Environment* **771**, 145077 (2021).
24. P. Lindahl *et al.*, Spatial and temporal distribution of Pu in the Northwest Pacific Ocean using modern coral archives. *Environment International* **40**, 196-201 (2012).
