# Supplementary material for: Toward defining the Anthropocene onset using a rapid increase in anthropogenic fingerprints in global geological archives
Source: Proc Natl Acad Sci U S A. 2024 Sep 23;121(41):e2313098121. doi: 10.1073/pnas.2313098121 (PMC11474069; doi:10.1073/pnas.2313098121)
Supplement: Supplementary file 3 — Dataset S02 (PDF) [file pnas.2313098121.sd02.pdf]

Dataset S02

Anthropogenic fingerprints detected in each record addressed in this study

Toward defining the Anthropocene onset using a rapid increase in anthropogenic fingerprints in global geological archives

Kuwae et al.

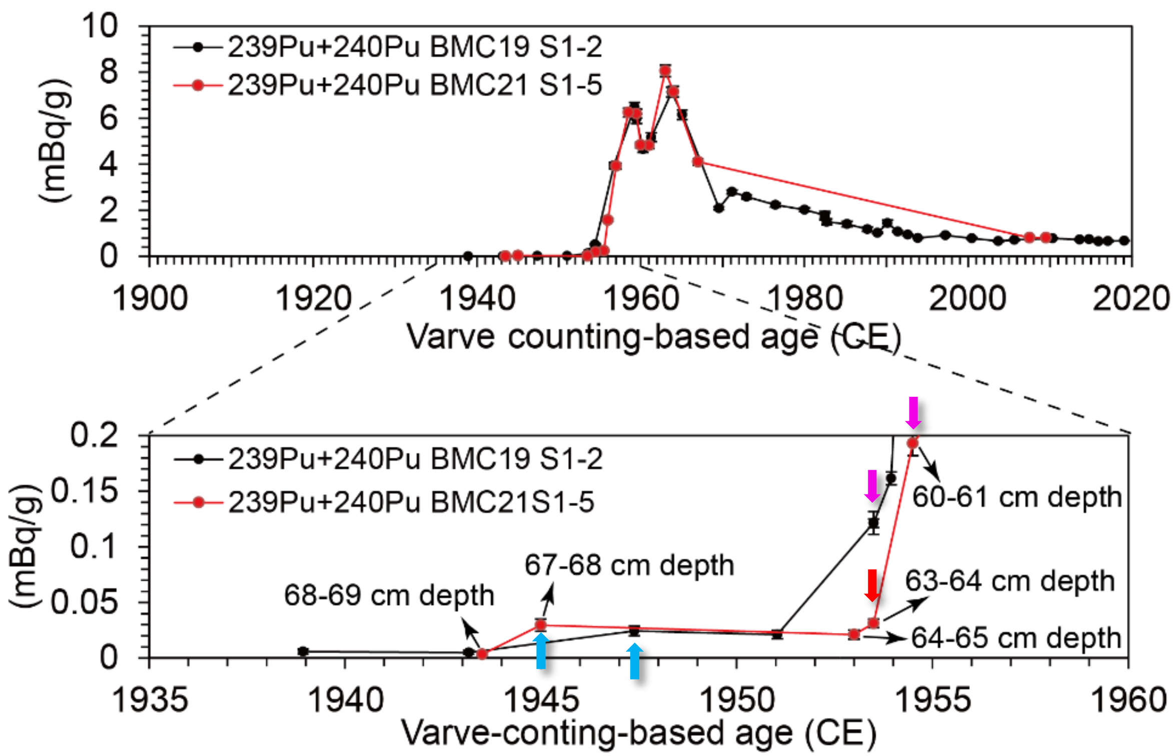

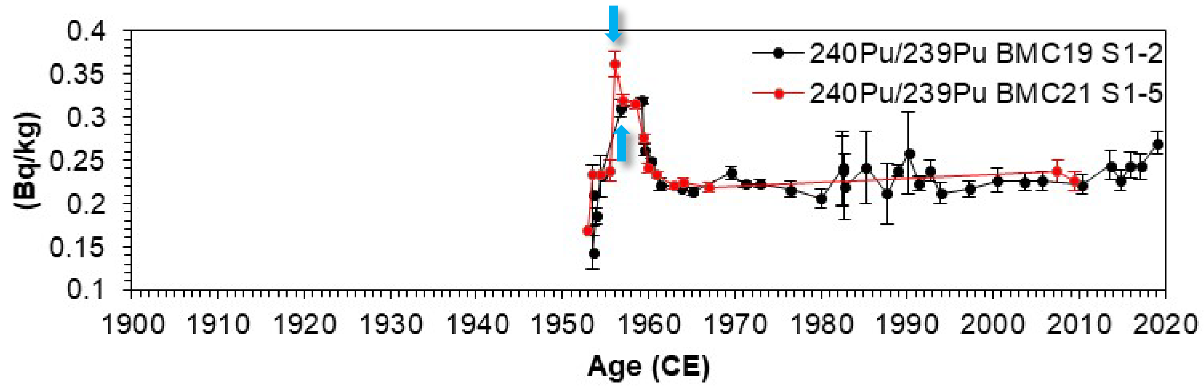

Kuwae et al. 2023

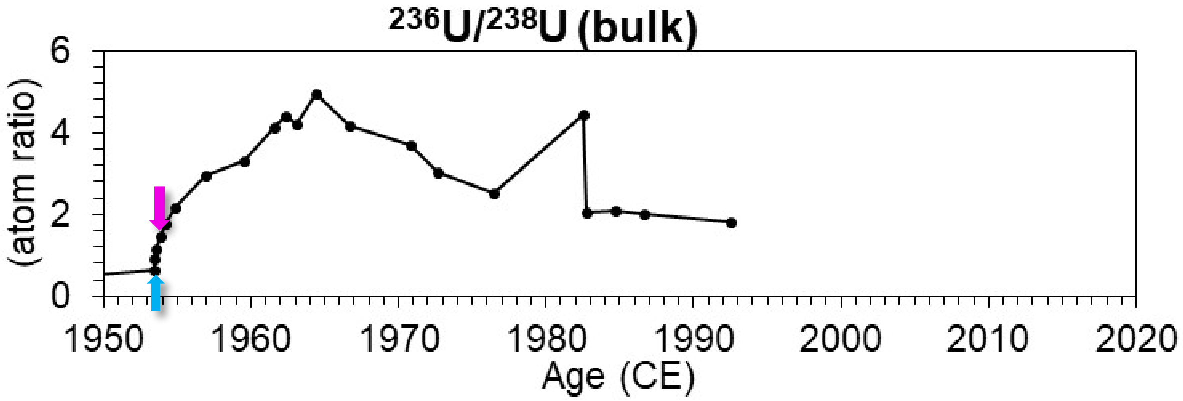

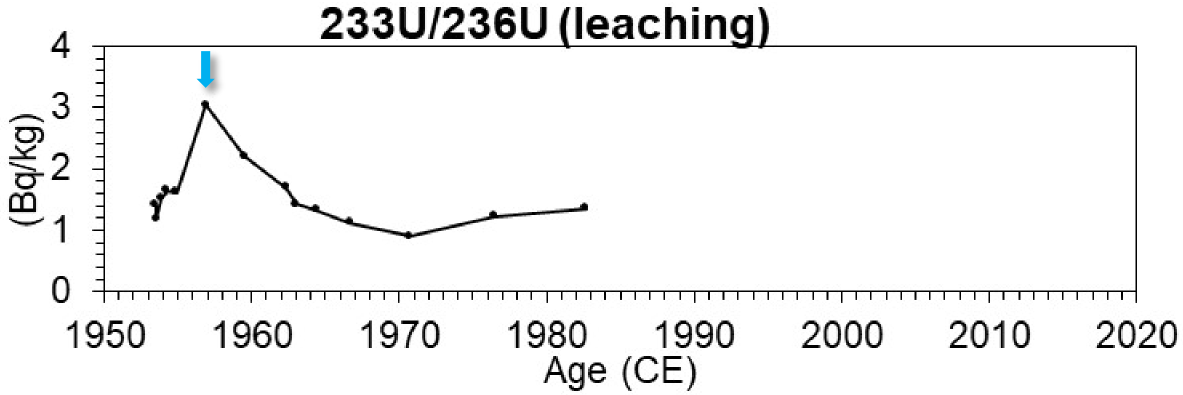

Kuwae et al. 2023

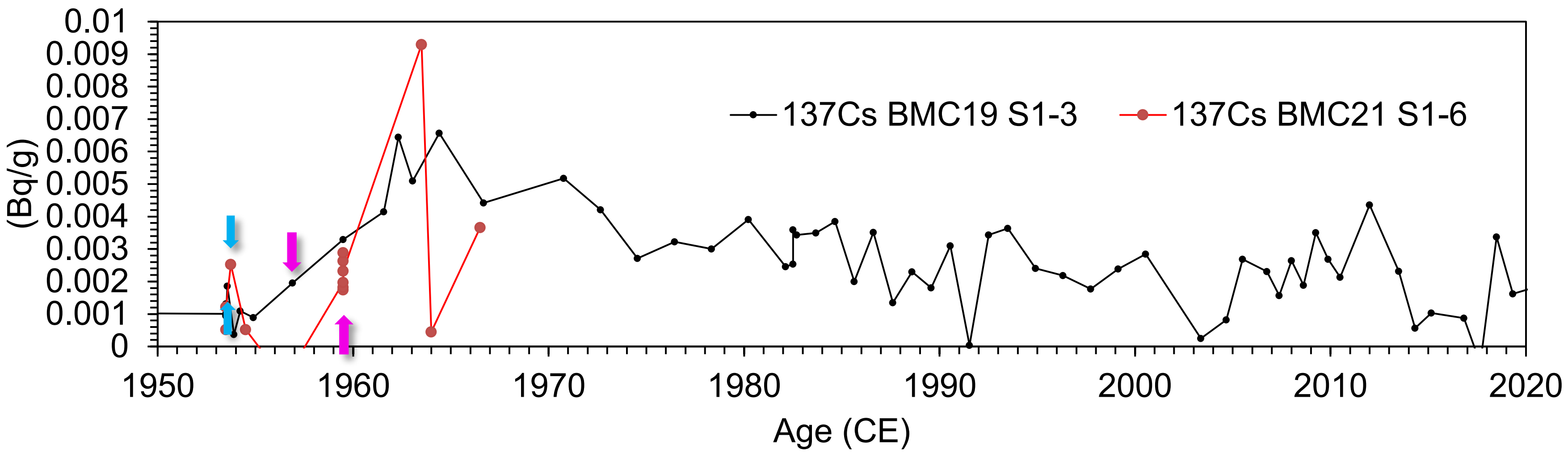

Kuwae et al. 2023

F_{modern} ^{14}C

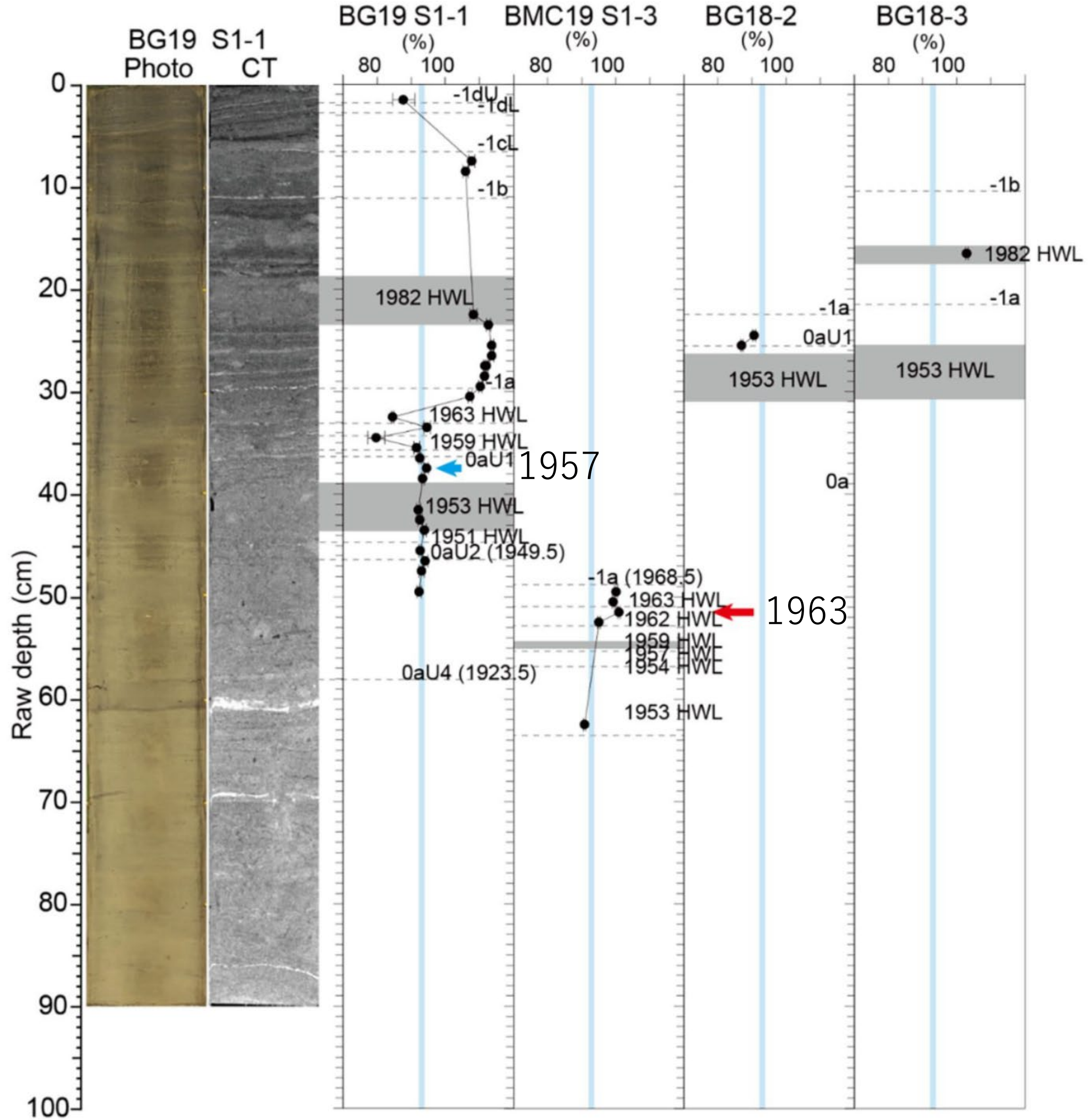

Kuwae et al. 2023

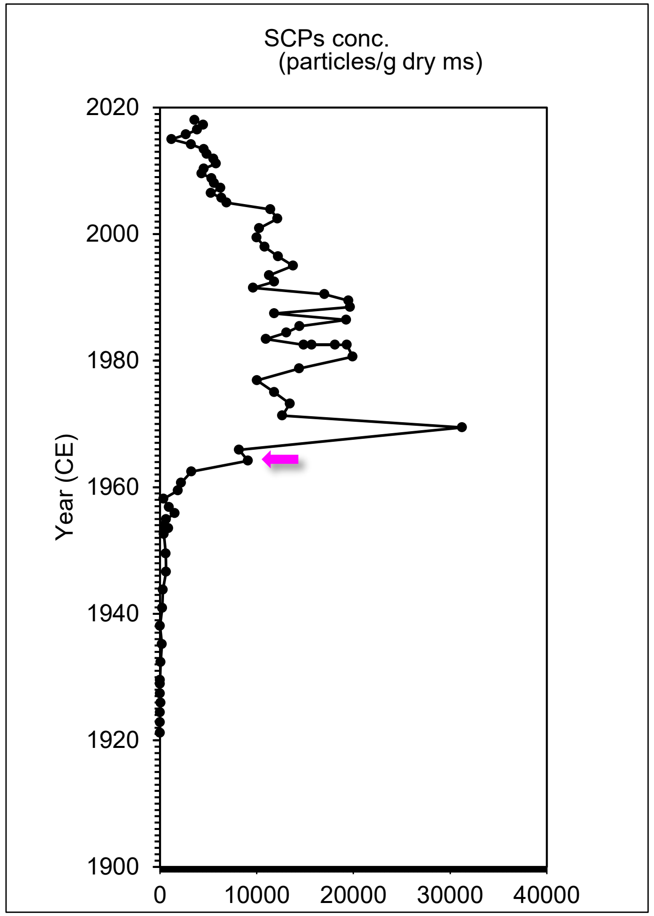

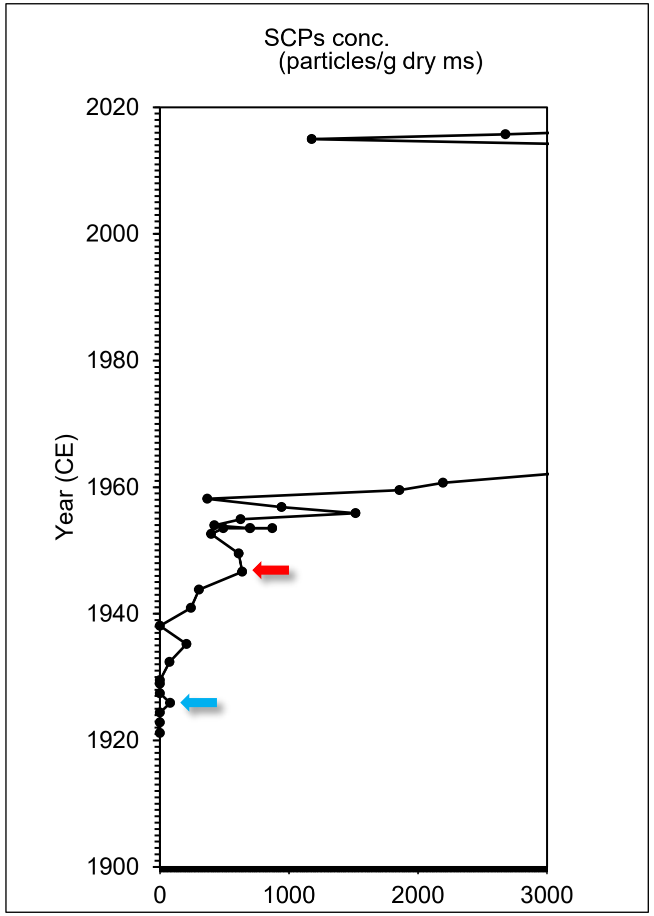

The same as left figure, but enlarged horizontal scale

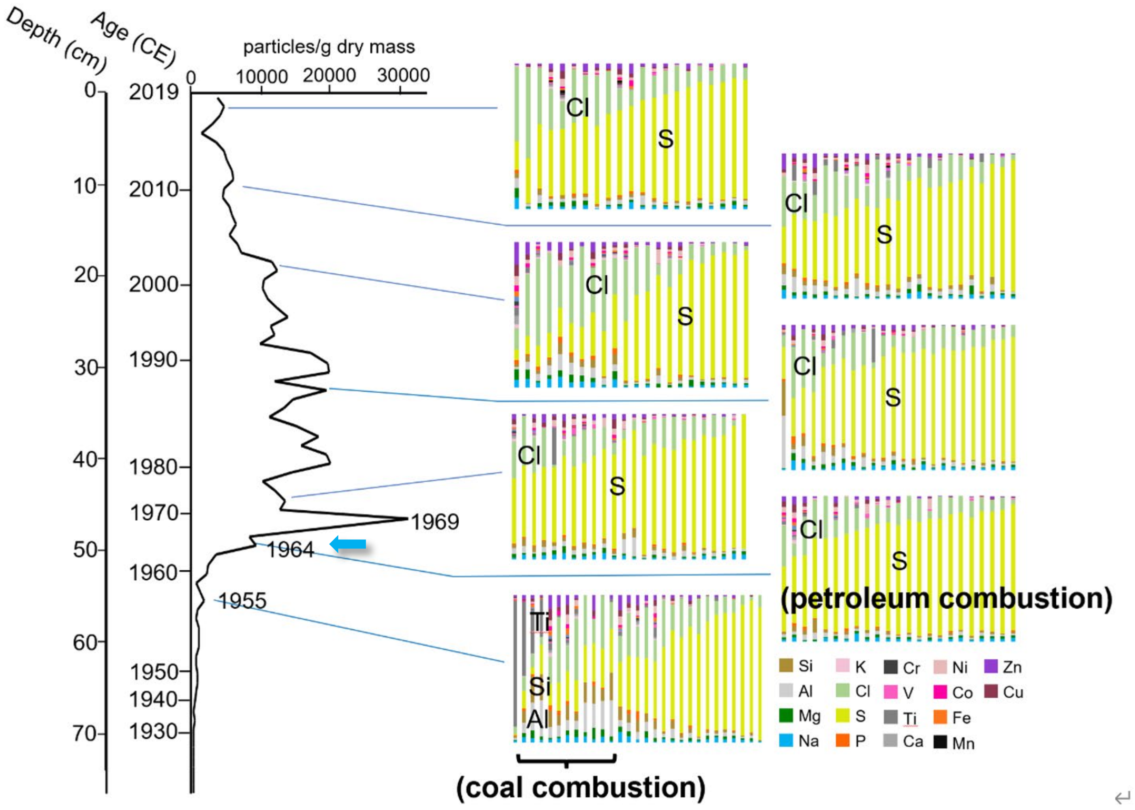

Kuwae et al. 2023

Intercore-averaged microplastic concentrations

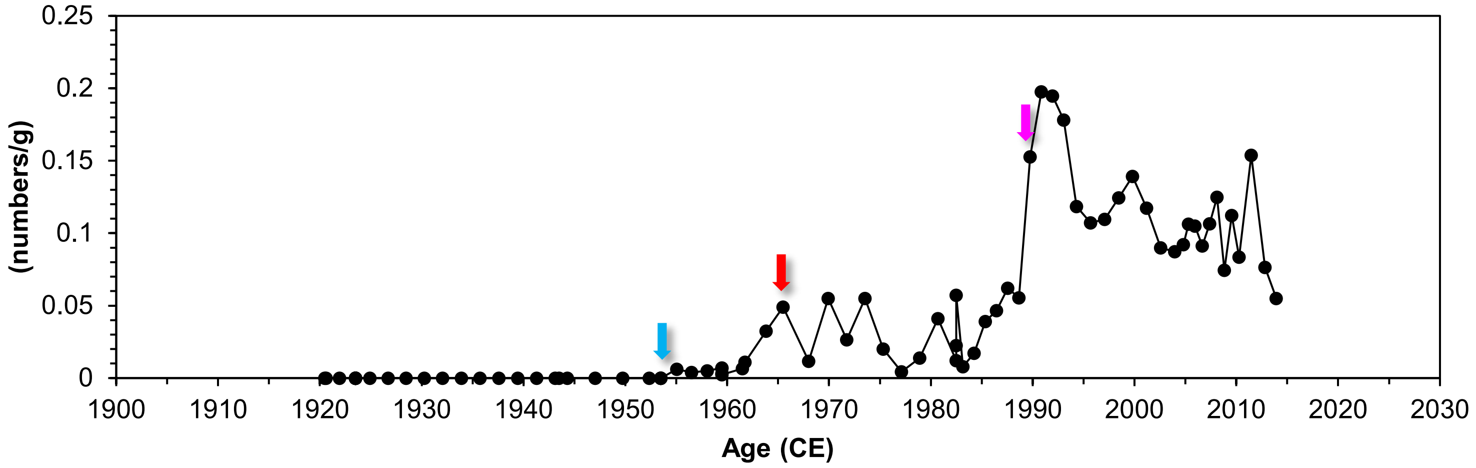

Kuwae et al. 2023

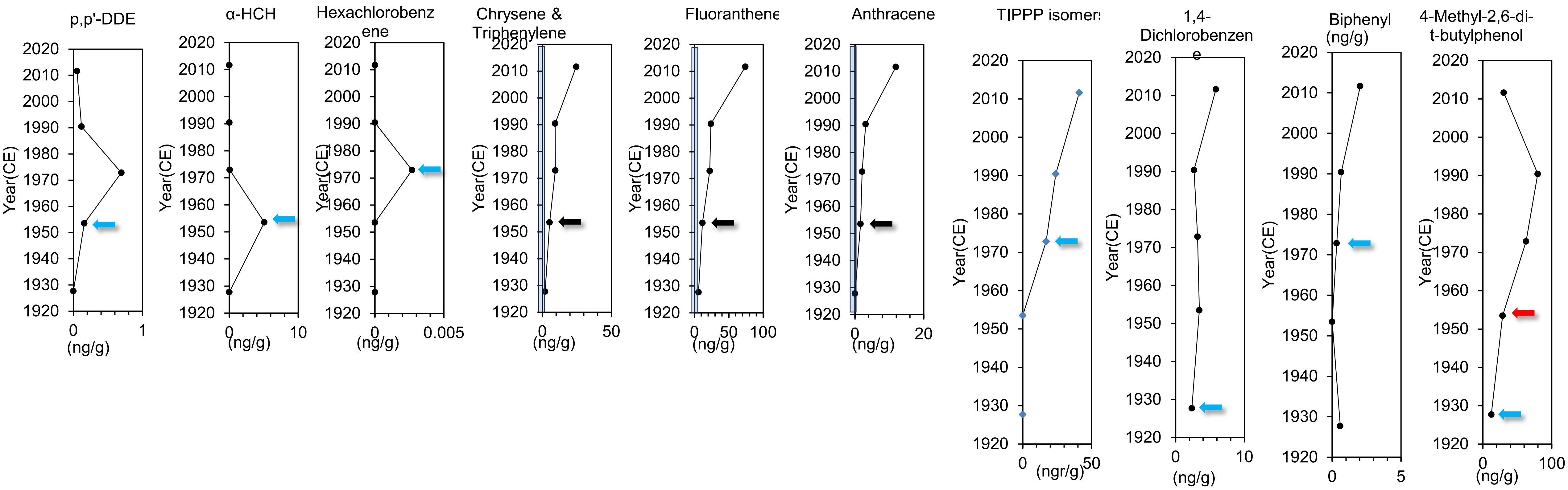

Kuwae et al. 2023

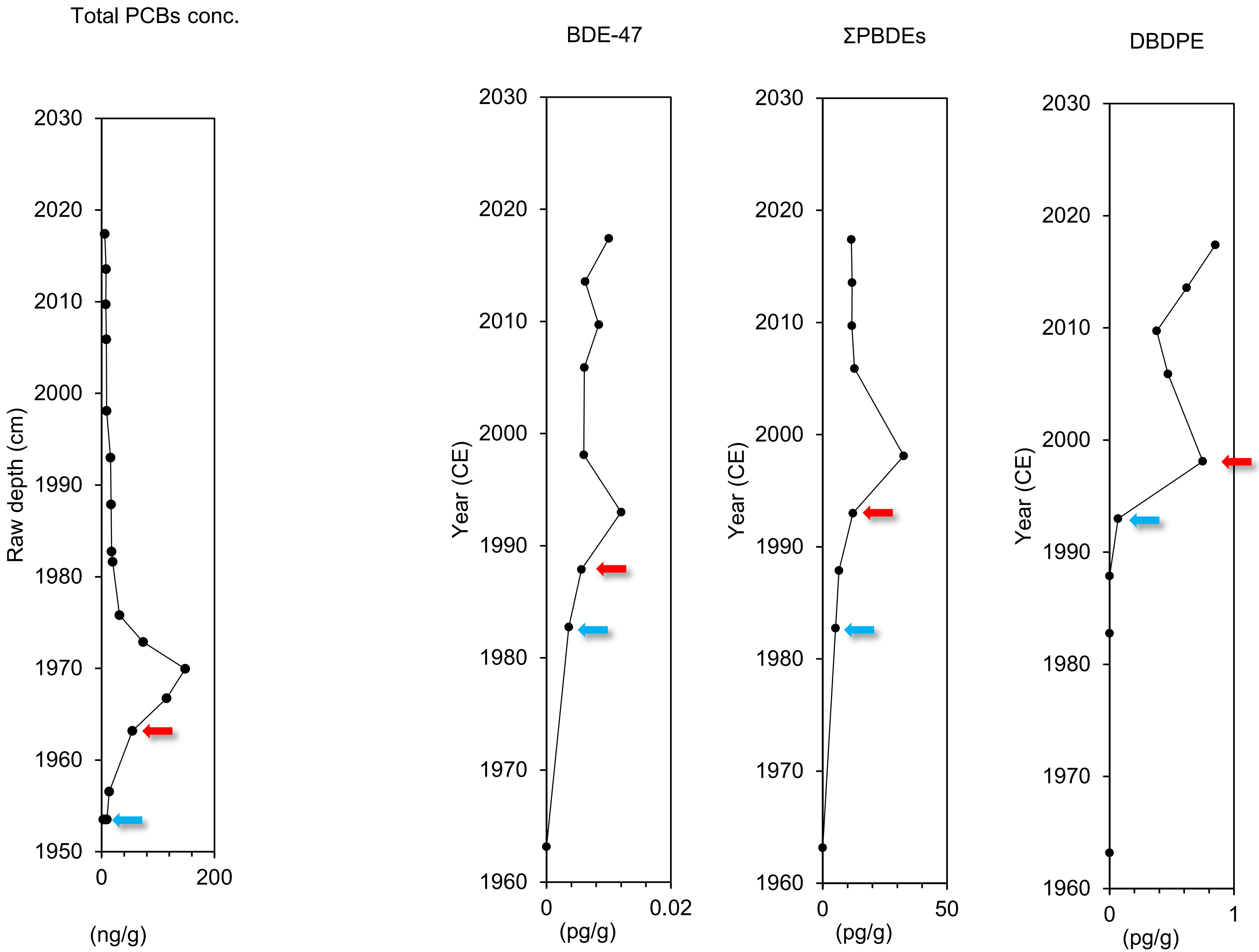

Kuwae et al. 2023

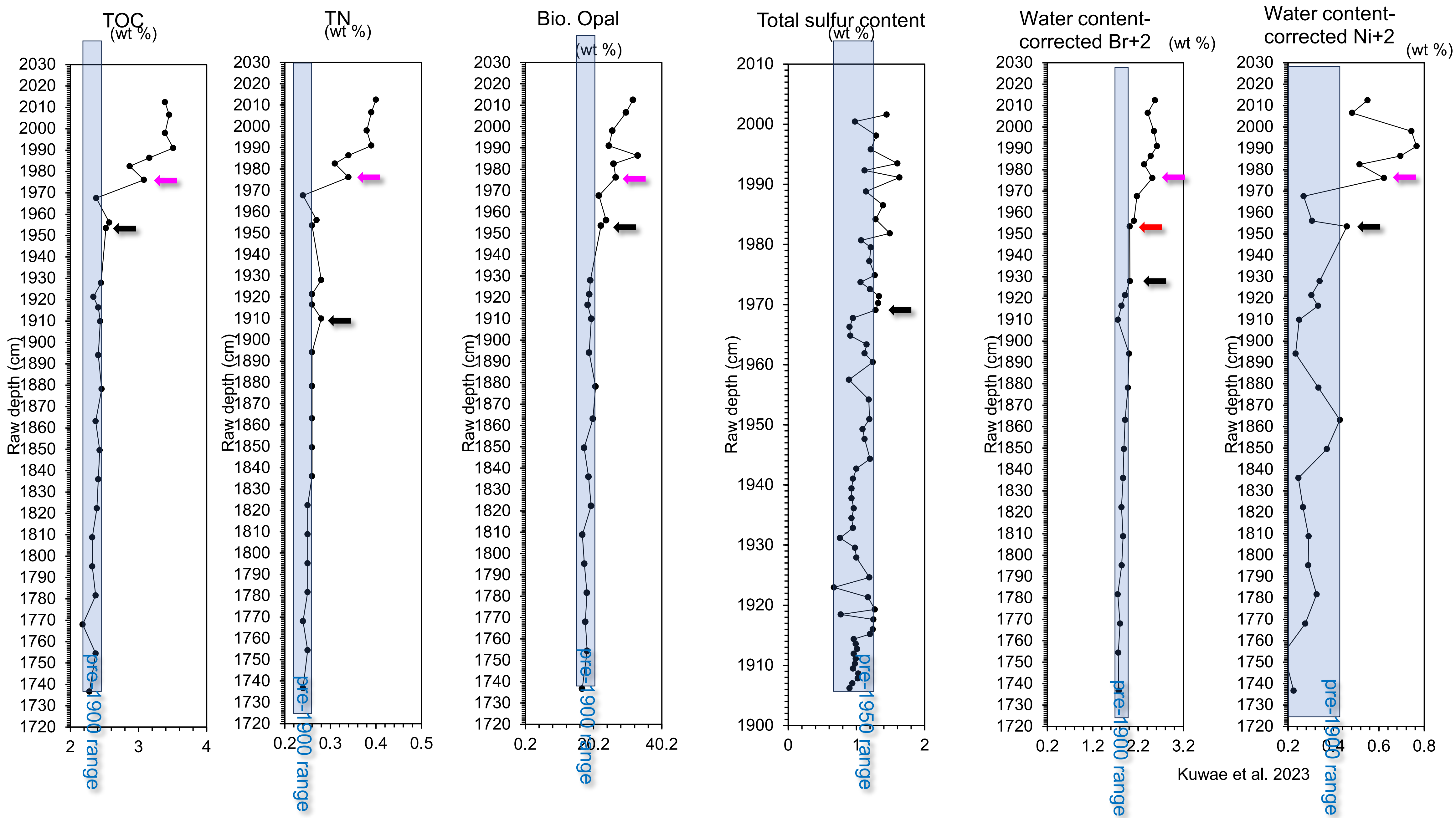
TOC (wt %)
TN (wt %)
Bio. Opal (wt %)
Total sulfur content (wt %)
Water content-corrected Br+2 (wt %)
Water content-corrected Ni+2 (wt %)
Raw depth (cm)
pre-1900 range
pre-1950 range
Kuwae et al. 2023

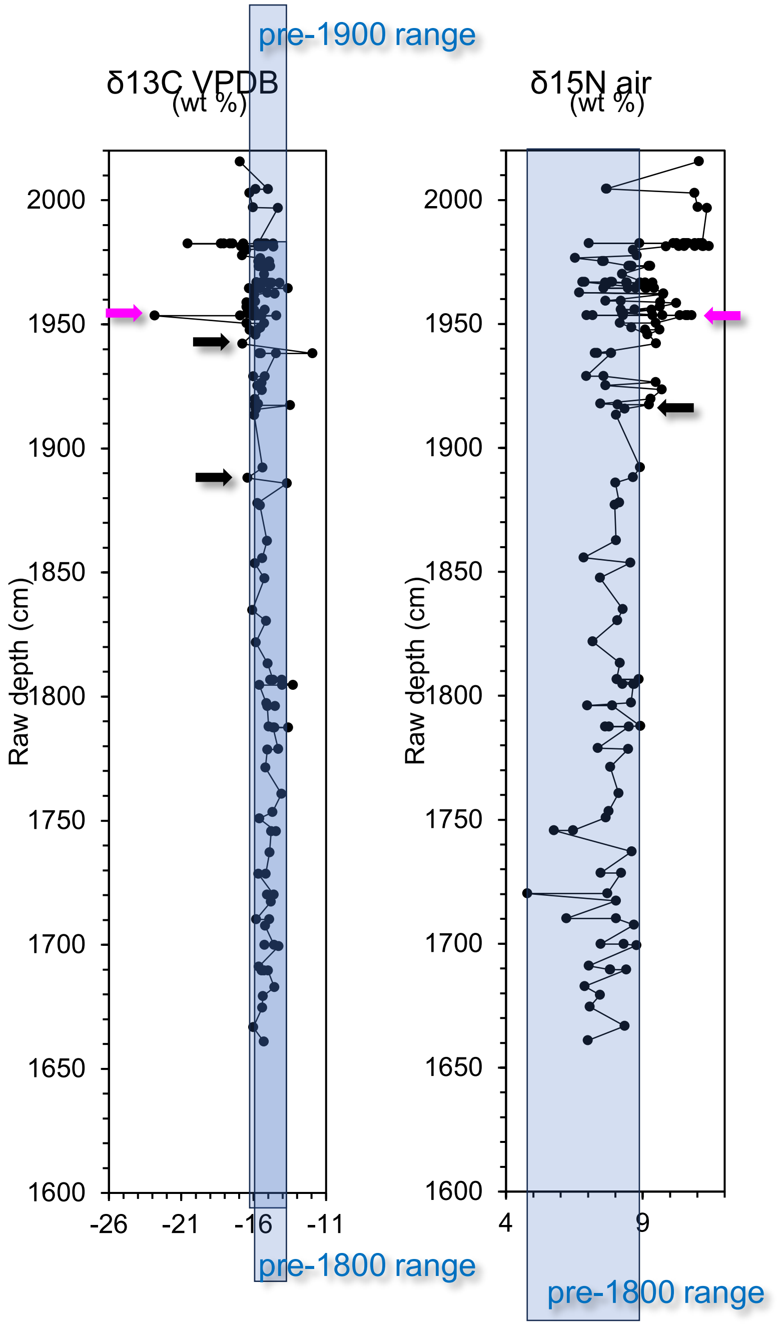

Kuwae et al. 2023

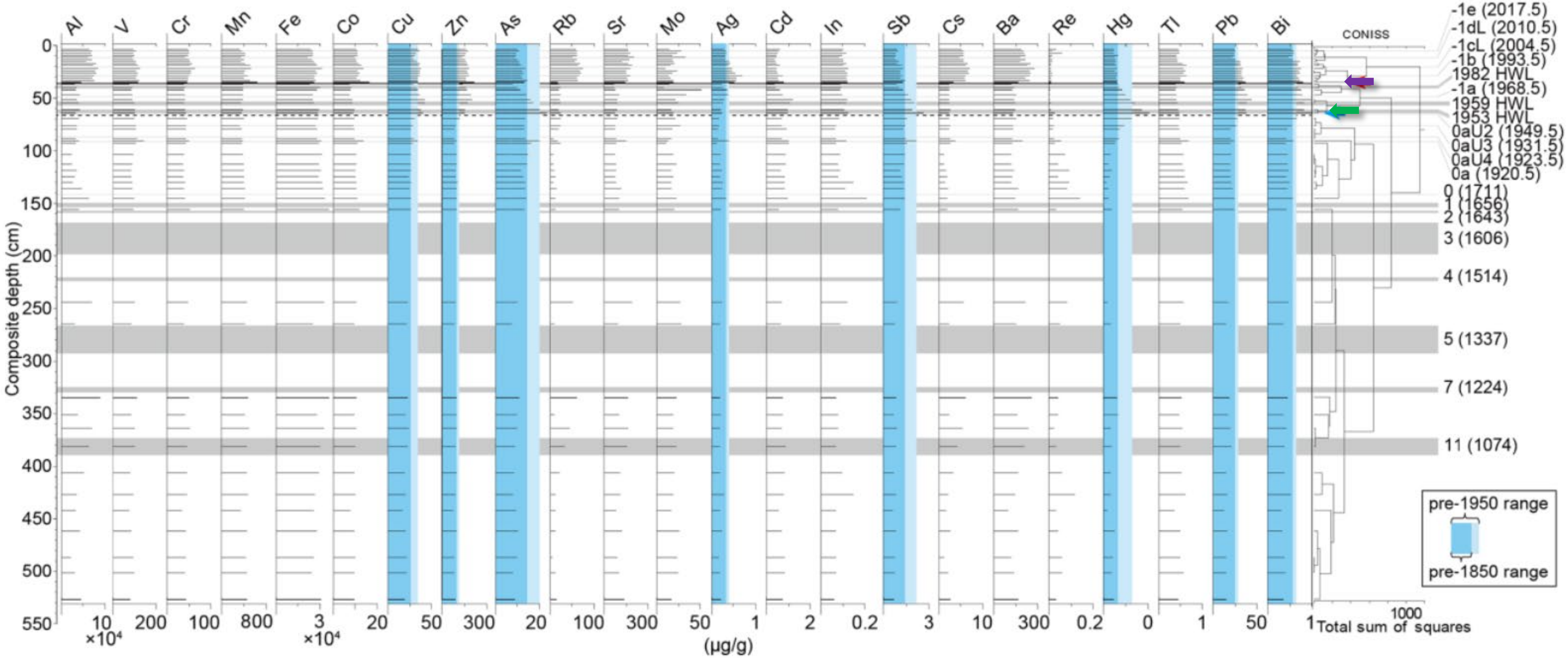

Kuwae et al. 2023

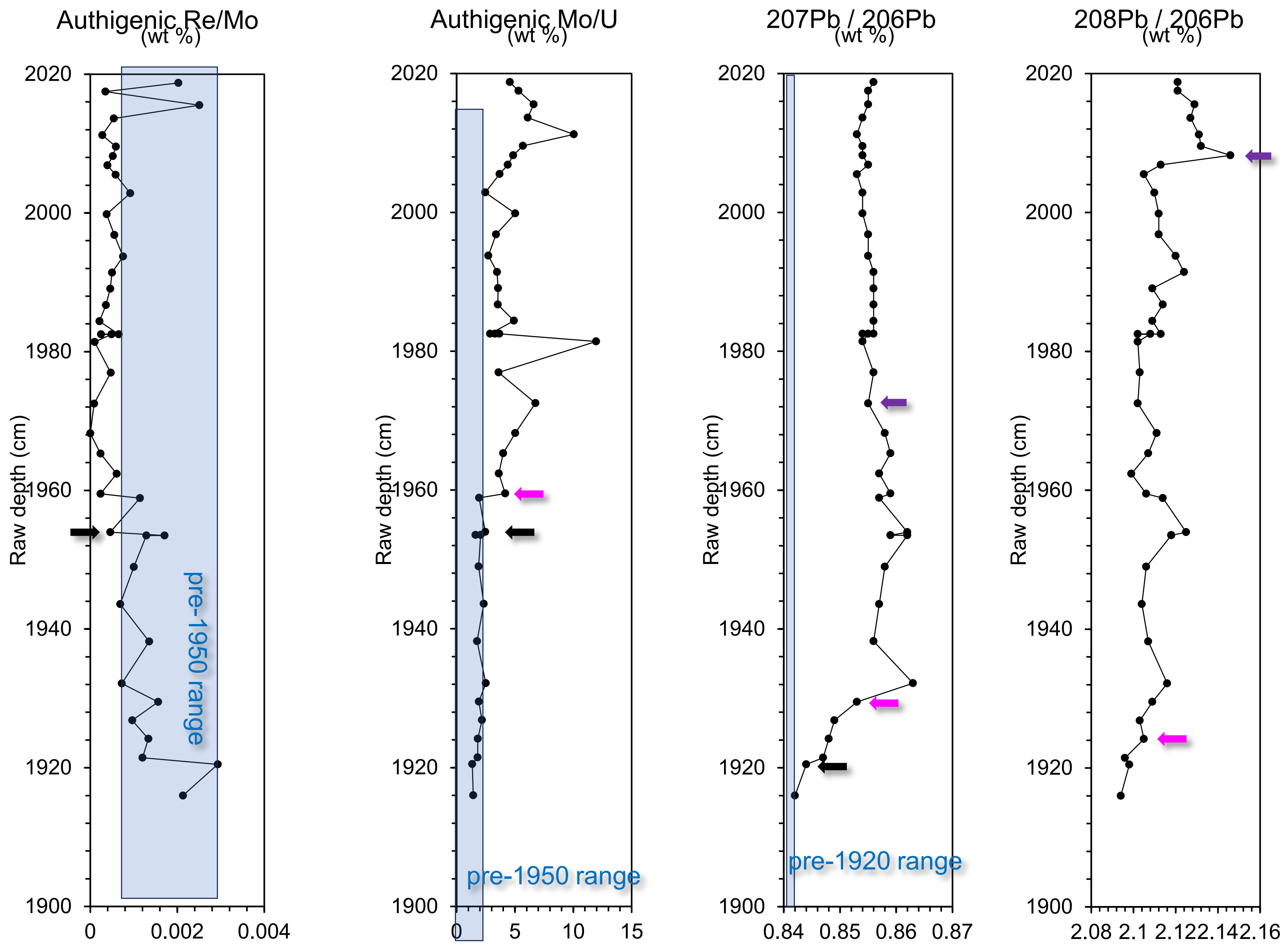

Kuwae et al. 2023

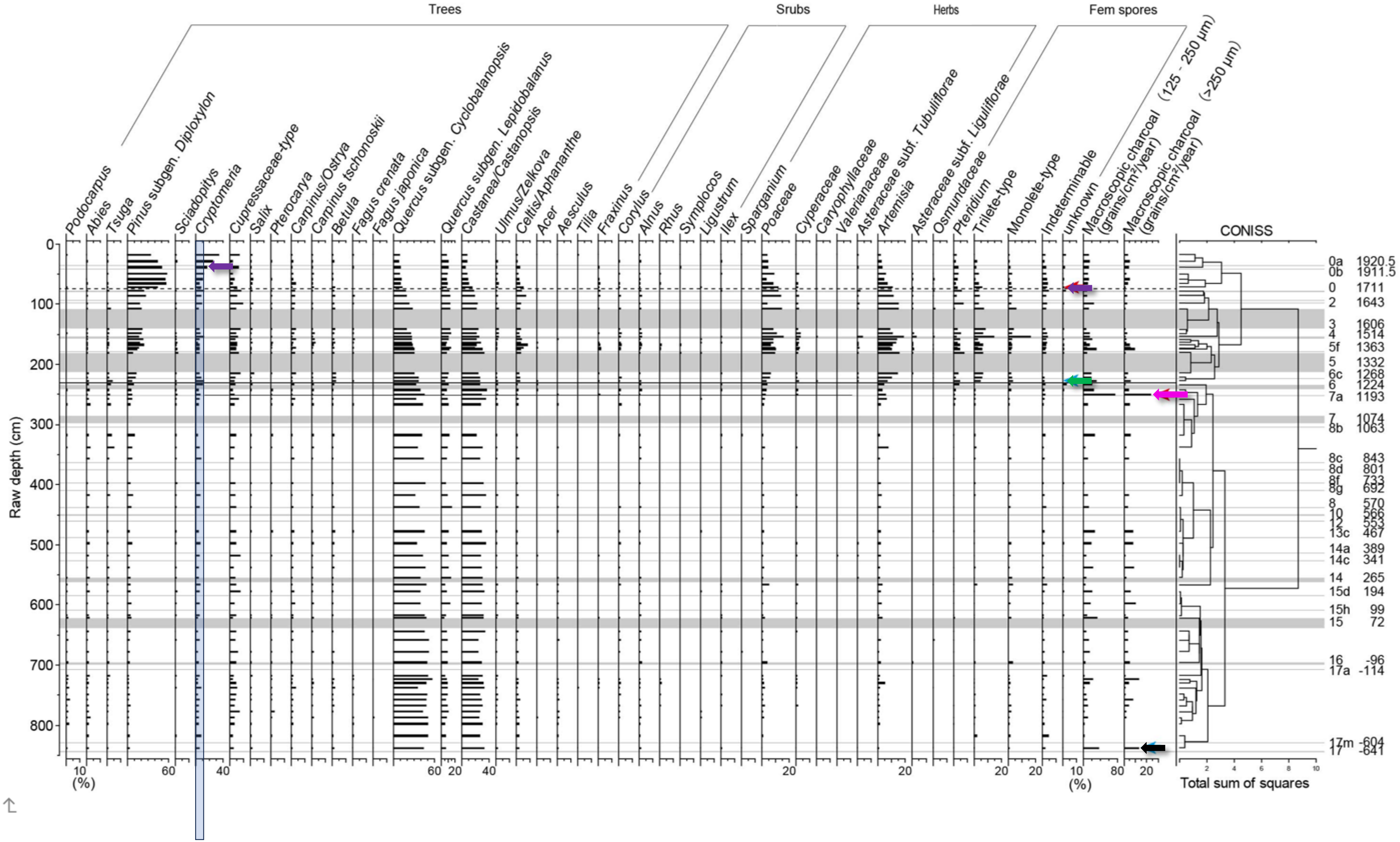

Trees
Srubs
Herbs
Fem spores
Podocarpus
Abies
Tsuga
Pinus subgen. Diploxylon
Sciadopitys
Cryptomeria
Cupressaceae-type
Salix
Pterocarya
Carpinus/Ostrya
Carpinus tschonoskii
Betula
Fagus crenata
Fagus japonica
Quercus subgen. Cyclobalanopsis
Quercus subgen. Lepidobalanus
Castanea/Castanopsis
Ulmus/Zelkova
Celtis/Aphananthe
Acer
Aesculus
Tilia
Fraxinus
Corylus
Alnus
Rhus
Symplocos
Ligustrum
Ilex
Sparganium
Poaceae
Cyperaceae
Caryophyllaceae
Valerianaceae
Asteraceae subf. Tubuliflorae
Artemisia
Asteraceae subf. Liguliflorae
Osmundaceae
Pteridium
Trilete-type
Monolete-type
Indeterminable
unknown
Macroscopic charcoal (125 - 250 μm) (grains/cm²/year)
Macroscopic charcoal (>250 μm) (grains/cm²/year)
CONISS
Raw depth (cm)
Total sum of squares
(%)
0a 1920.5
0b 1911.5
0 1711
2 1643
3 1606
4 1514
5f 1363
5 1332
6c 1268
6 1224
7a 1193
7 1074
8b 1063
8c 843
8d 801
8f 733
8g 692
8 570
10 566
12 553
13c 467
14a 389
14c 341
14 265
15d 194
15h 99
15 72
16 -96
17a -114
17m -604
17 -641

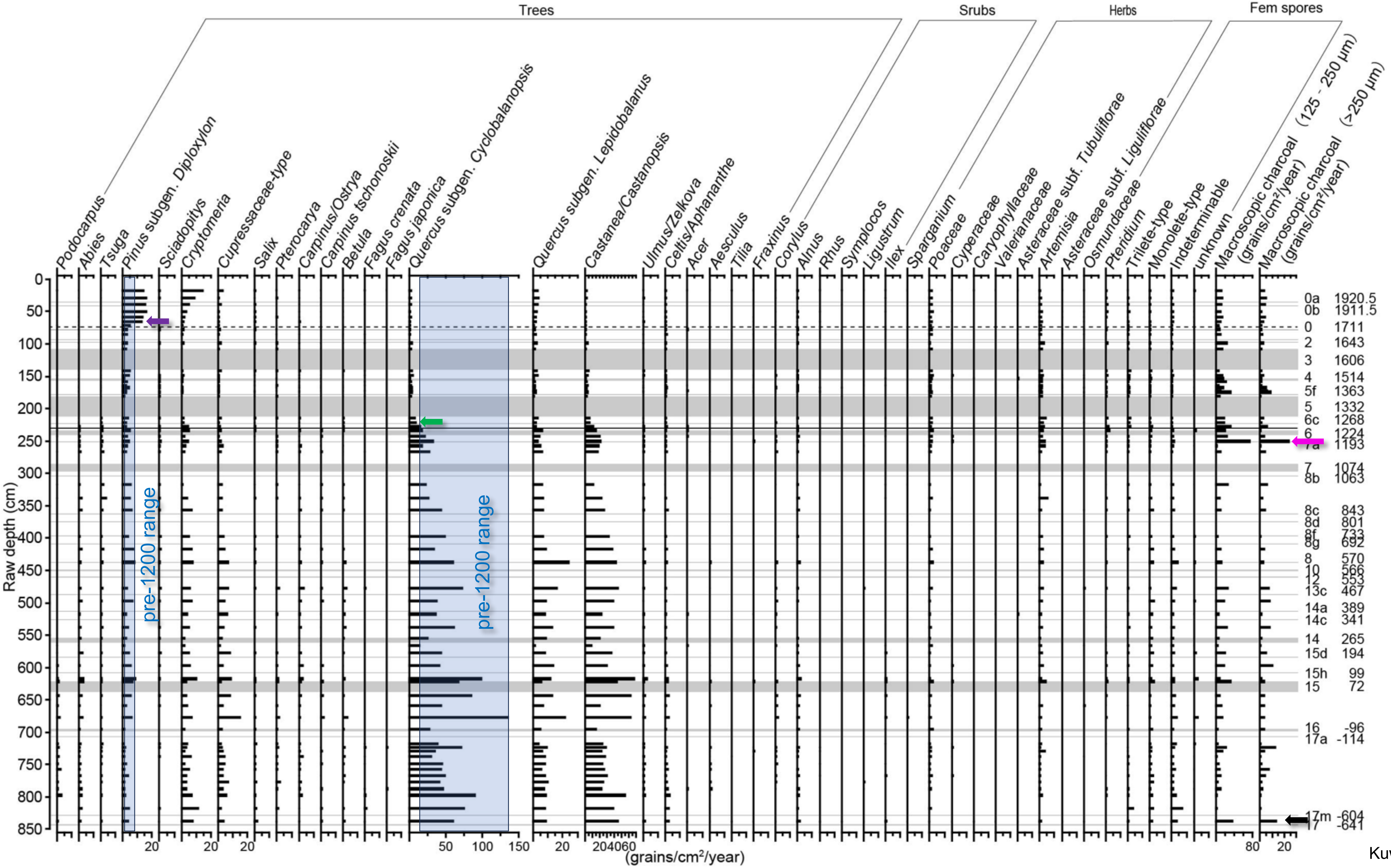

Kuwae et al. 2023

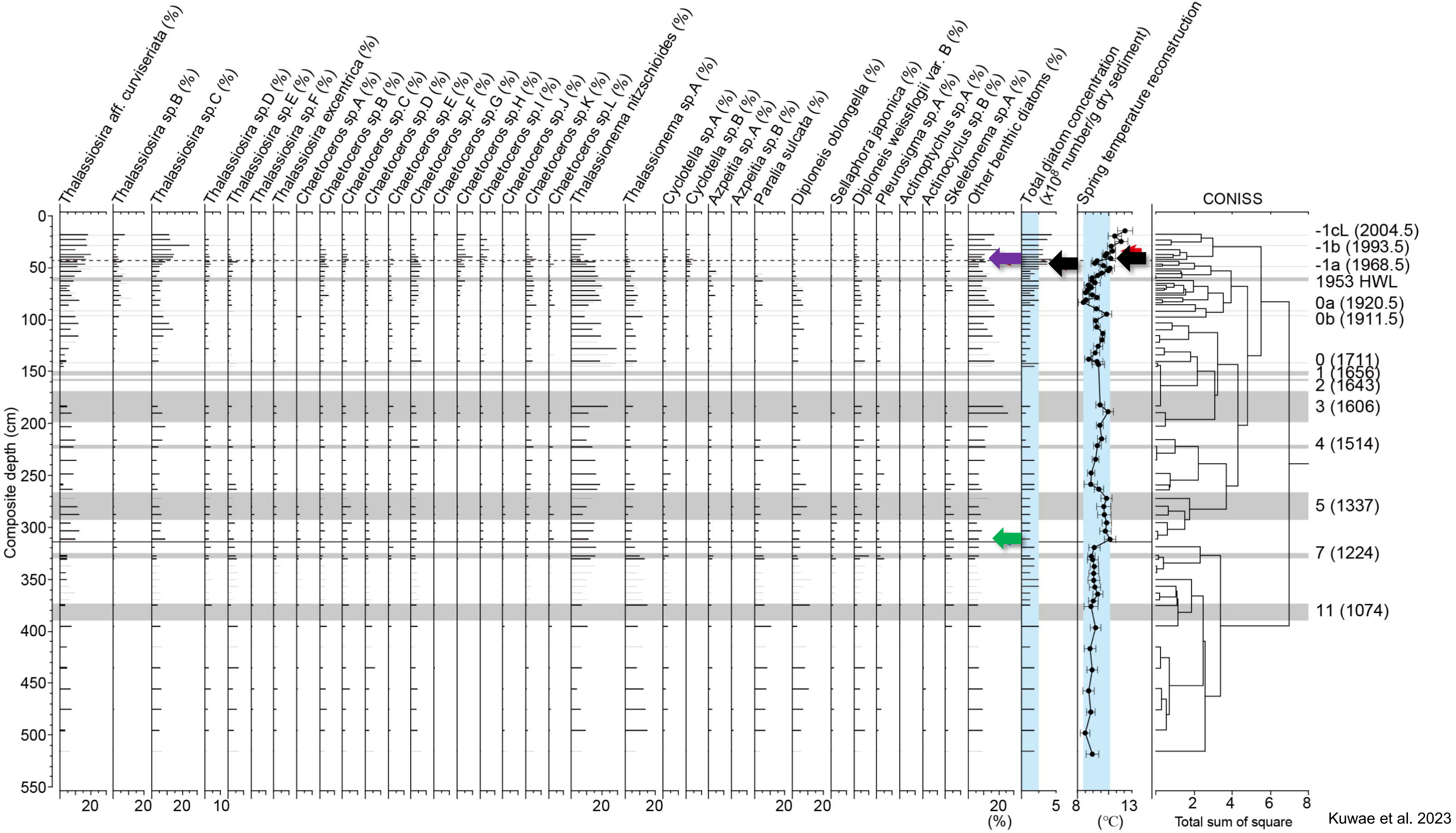

Thalassiosira aff. curviseriata (%)
Thalassiosira sp.B (%)
Thalassiosira sp.C (%)
Thalassiosira sp.D (%)
Thalassiosira sp.E (%)
Thalassiosira sp.F (%)
Thalassiosira excentrica (%)
Chaetoceros sp.A (%)
Chaetoceros sp.B (%)
Chaetoceros sp.C (%)
Chaetoceros sp.D (%)
Chaetoceros sp.E (%)
Chaetoceros sp.F (%)
Chaetoceros sp.G (%)
Chaetoceros sp.H (%)
Chaetoceros sp.I (%)
Chaetoceros sp.J (%)
Chaetoceros sp.K (%)
Chaetoceros sp.L (%)
Thalassionema nitzschioides (%)
Thalassionema sp.A (%)
Cyclotella sp.A (%)
Cyclotella sp.B (%)
Azpeitia sp.A (%)
Azpeitia sp.B (%)
Paralia sulcata (%)
Diploneis oblongella (%)
Sellaphora japonica (%)
Diploneis weissflogii var. B (%)
Pleurosigma sp.A (%)
Actinoptychus sp.A (%)
Actinocyclus sp.B (%)
Skeletonema sp.A (%)
Other benthic diatoms (%)
Total diatom concentration
(x10^8 number/g dry sediment)
Spring temperature reconstruction
CONISS
Composite depth (cm)
0
50
100
150
200
250
300
350
400
450
500
550
-1cL (2004.5)
-1b (1993.5)
-1a (1968.5)
1953 HWL
0a (1920.5)
0b (1911.5)
0 (1711)
1 (1656)
2 (1643)
3 (1606)
4 (1514)
5 (1337)
7 (1224)
11 (1074)
20
20
20
10
20
20
20
20
20
(%)
5
8
13
(°C)
2
4
6
8
Total sum of square
Kuwae et al. 2023

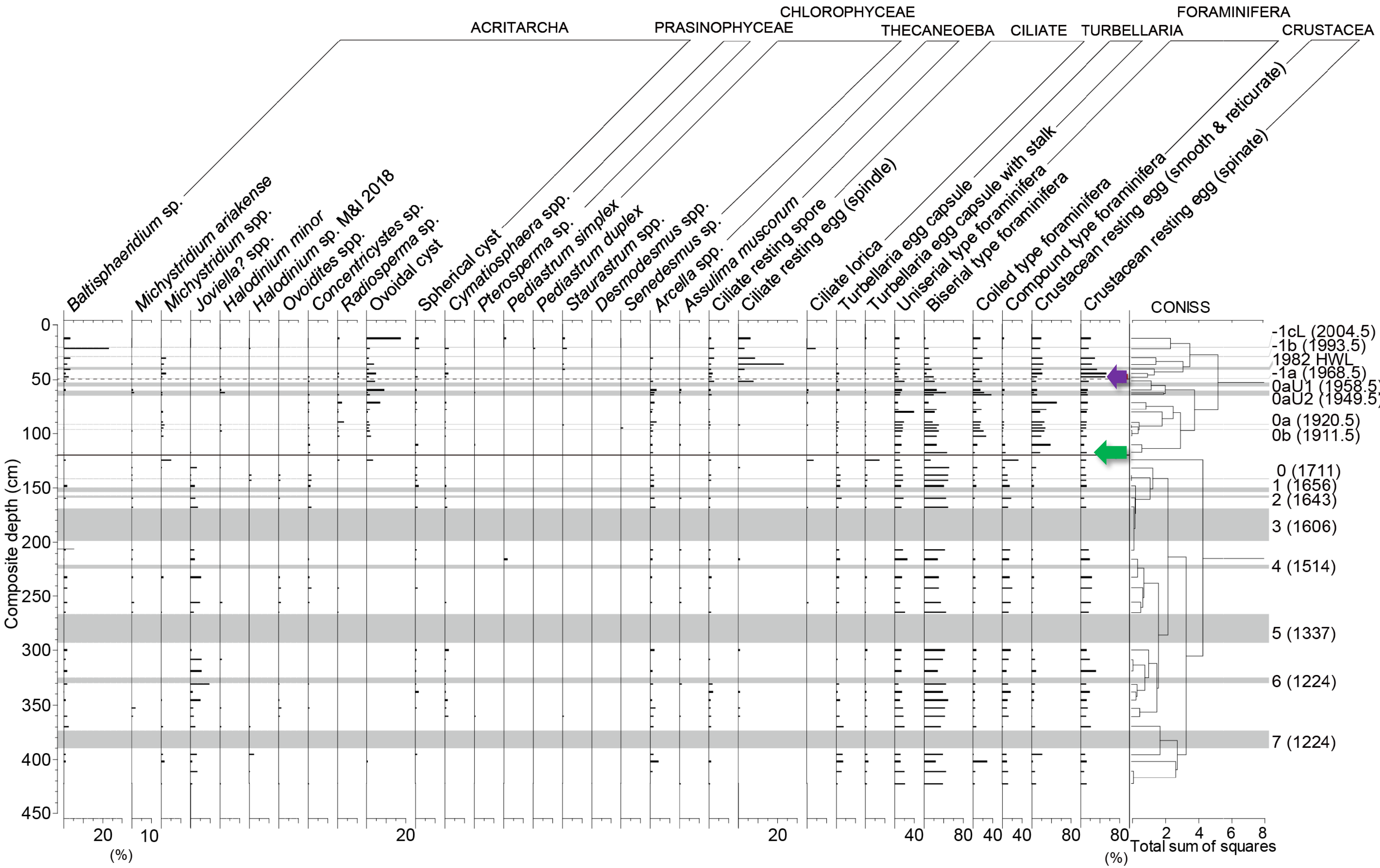

Kuwae et al. 2023

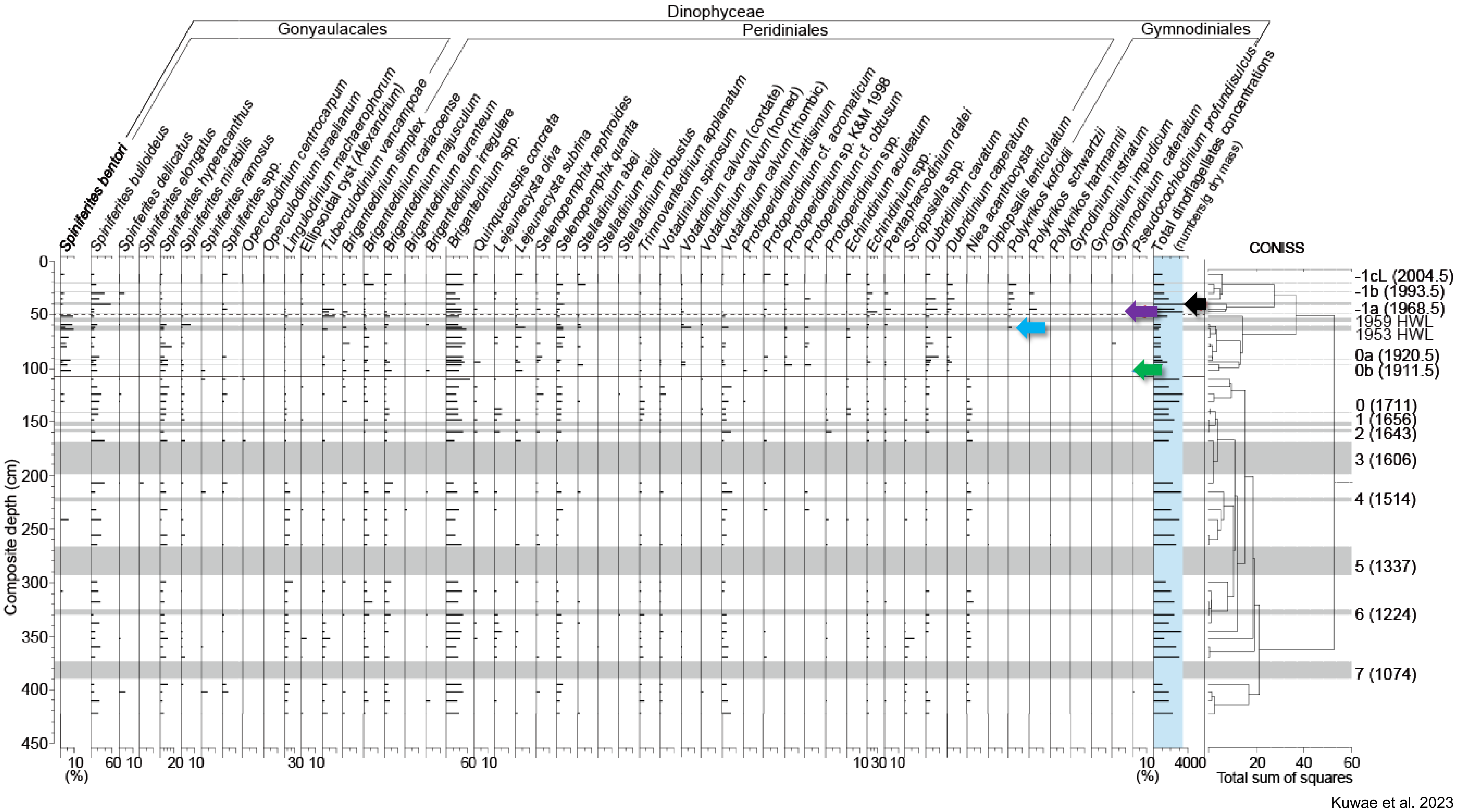

Dinophyceae
Gonyaulacales
Peridiniales
Gymnodiniales
Spiniferites bentori
Spiniferites bulloideus
Spiniferites delicatus
Spiniferites elongatus
Spiniferites hyperacanthus
Spiniferites mirabilis
Spiniferites ramosus
Spiniferites spp.
Operculodinium centrocarpum
Operculodinium israelianum
Lingulodinium machaerophorum
Ellipsoidal cyst (Alexandrium)
Tuberculodinium vancampoae
Brigantedinium simplex
Brigantedinium cariacoense
Brigantedinium majusculum
Brigantedinium auranteum
Brigantedinium irregulare
Brigantedinium spp.
Quinquecuspis concreta
Lejeunecysta oliva
Lejeunecysta subrina
Selenopemphix nephroides
Selenopemphix quanta
Stelladinium abei
Stelladinium reidii
Stelladinium robustus
Trinnovantedinium applanatum
Votadinium spinosum
Votadinium calvum (cordate)
Votadinium calvum (horned)
Votadinium calvum (rhombic)
Protoperidinium latissimum
Protoperidinium cf. acromaticum
Protoperidinium sp. K&M 1998
Protoperidinium cf. obtusum
Protoperidinium spp.
Echinidinium aculeatum
Echinidinium spp.
Pentapharsodinium dalei
Scrippsiella spp.
Dubridinium cavatum
Dubridinium caperatum
Niea acanthocysta
Diplopsalis lenticulatum
Polykrikos kofoidii
Polykrikos schwartzii
Polykrikos hartmannii
Gyrodinium instriatum
Gyrodinium impudicum
Gymnodinium catenatum
Pseudocochlodinium profundisulcus
Total dinoflagellates concentrations (numbers/g dry mass)
CONISS
Composite depth (cm)
0
50
100
150
200
250
300
350
400
450
10
(%)
60
10
20
10
30
10
60
10
10
30
10
10
(%)
4000
20
40
60
Total sum of squares
-1cL (2004.5)
-1b (1993.5)
-1a (1968.5)
1959 HWL
1953 HWL
0a (1920.5)
0b (1911.5)
0 (1711)
1 (1656)
2 (1643)
3 (1606)
4 (1514)
5 (1337)
6 (1224)
7 (1074)
Kuwae et al. 2023

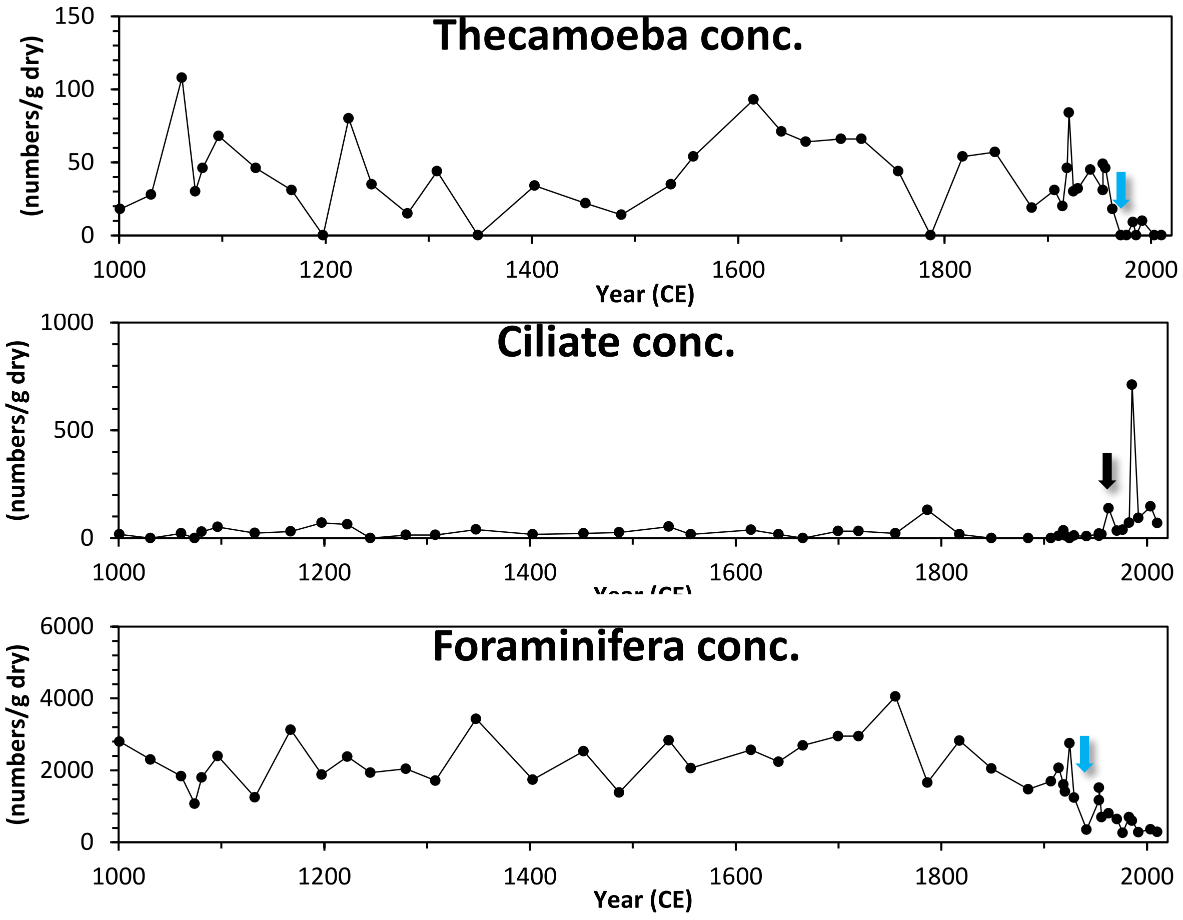

Kuwae et al. 2023

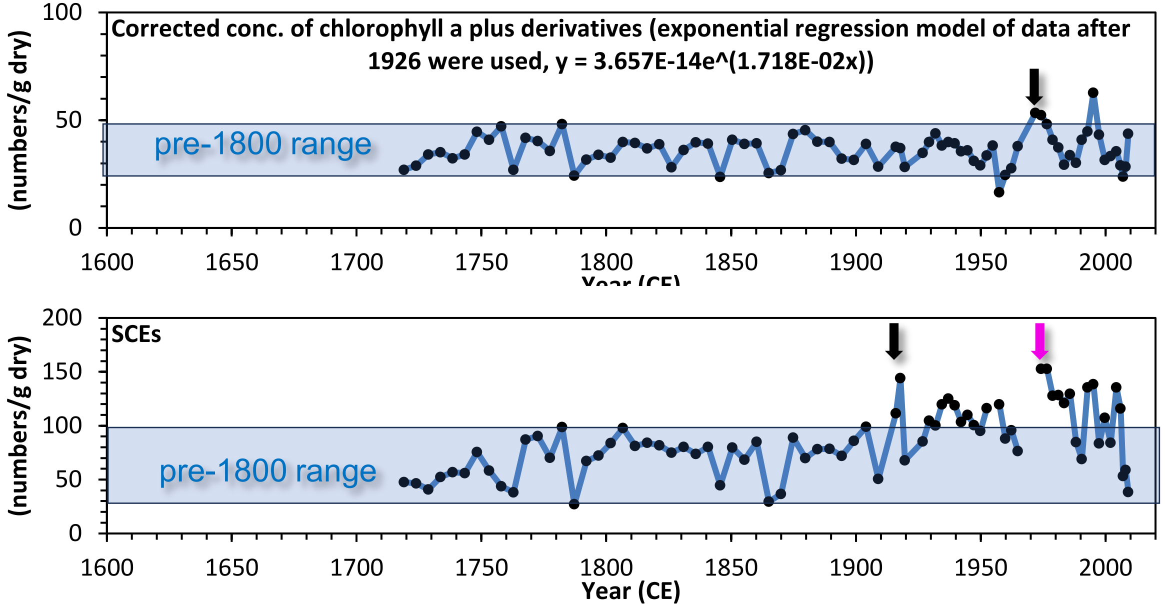

Kuwae et al. 2023

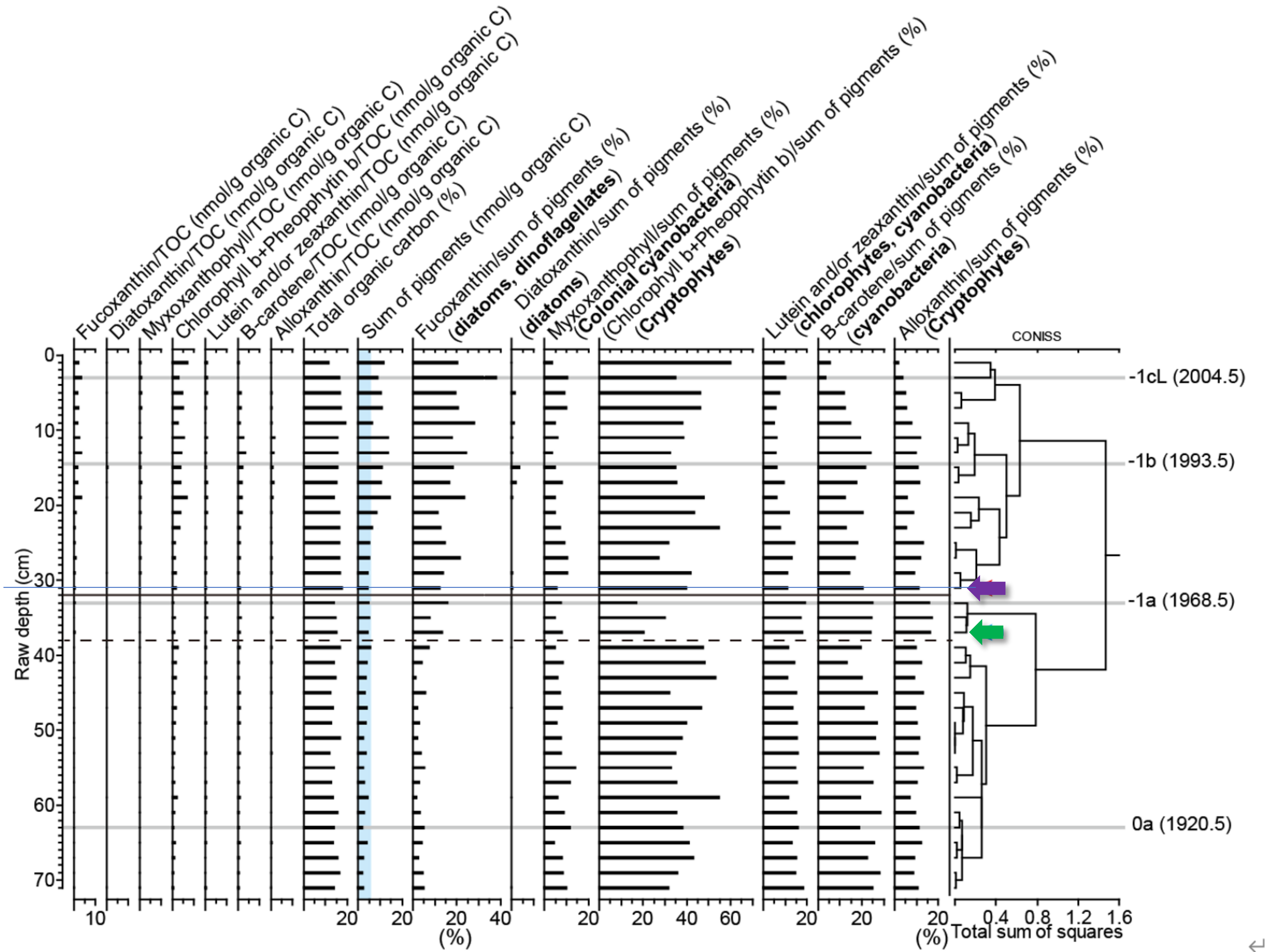

Kuwae et al. 2023

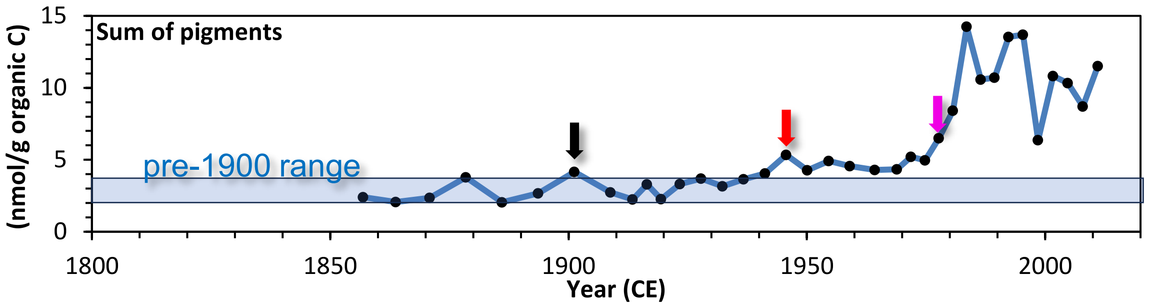

Kuwae et al. 2023

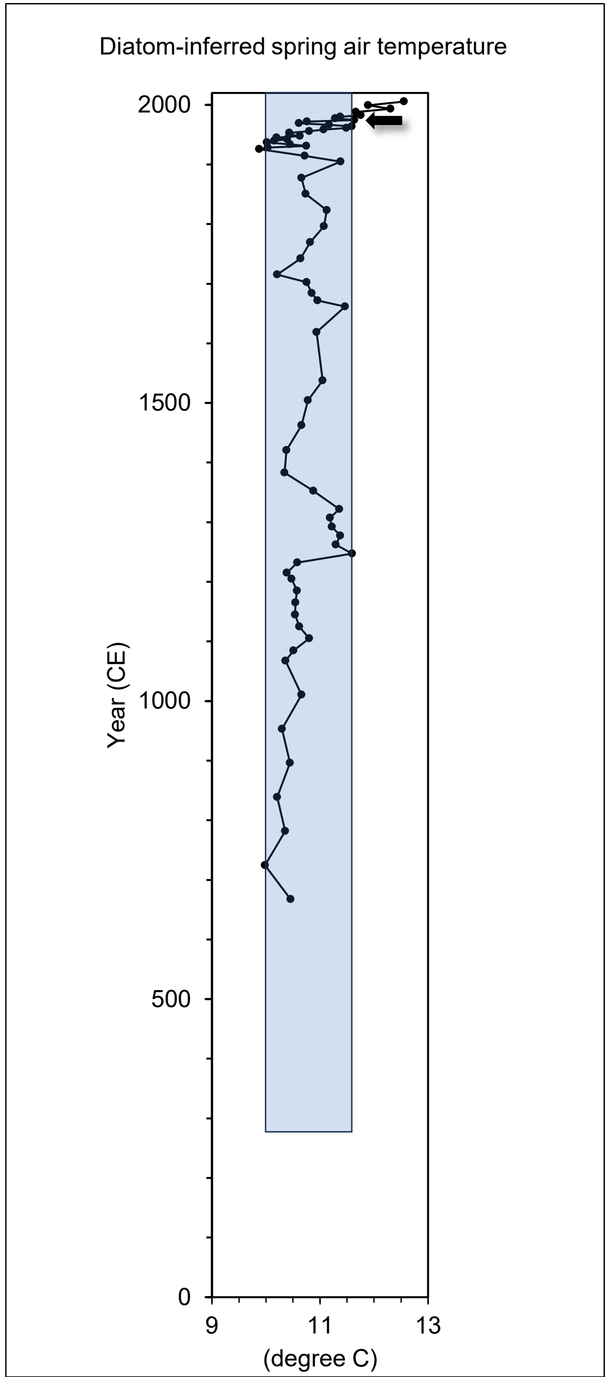

Kuwae et al. 2023

Depth profile of ITRAX-derived element contents in BMC 19 S1-1

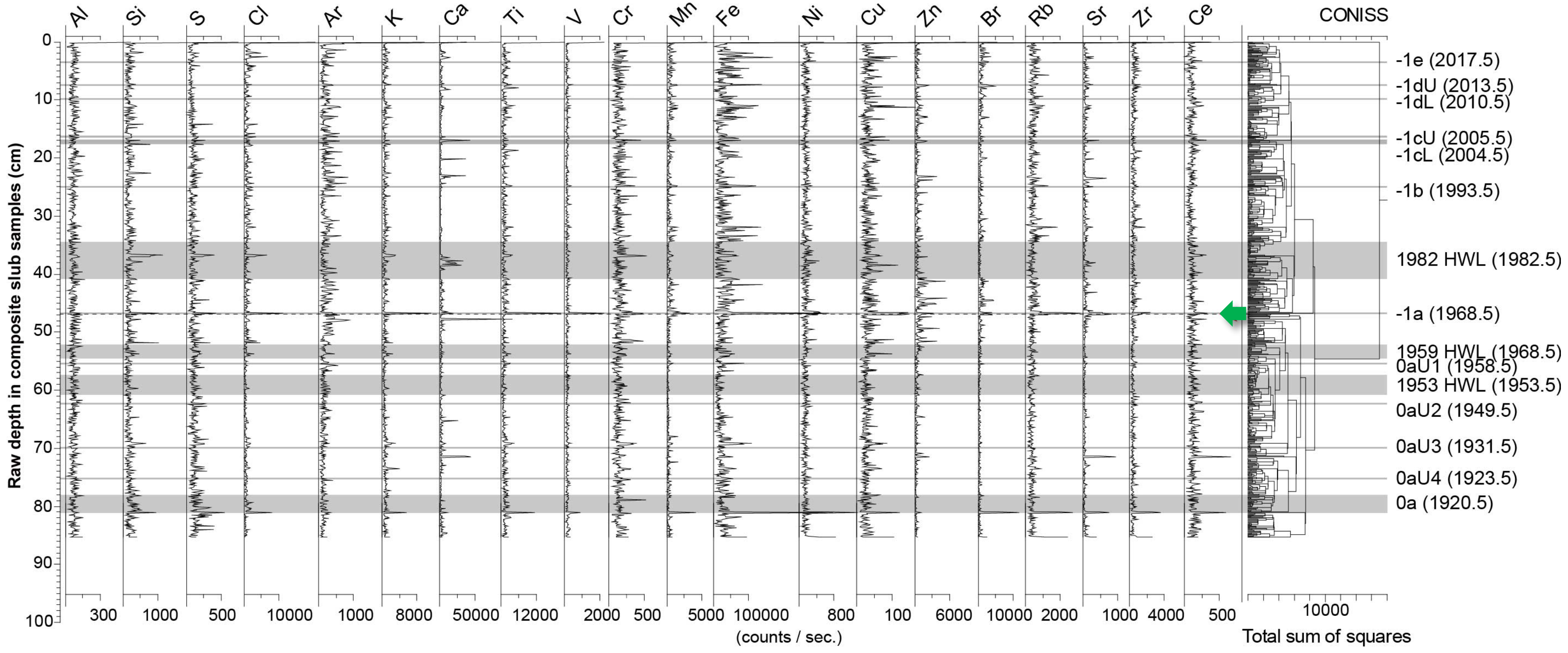

Kuwae et al. 2023

Depth profile of ITRAX-derived element contents in BMC 19 S1-3

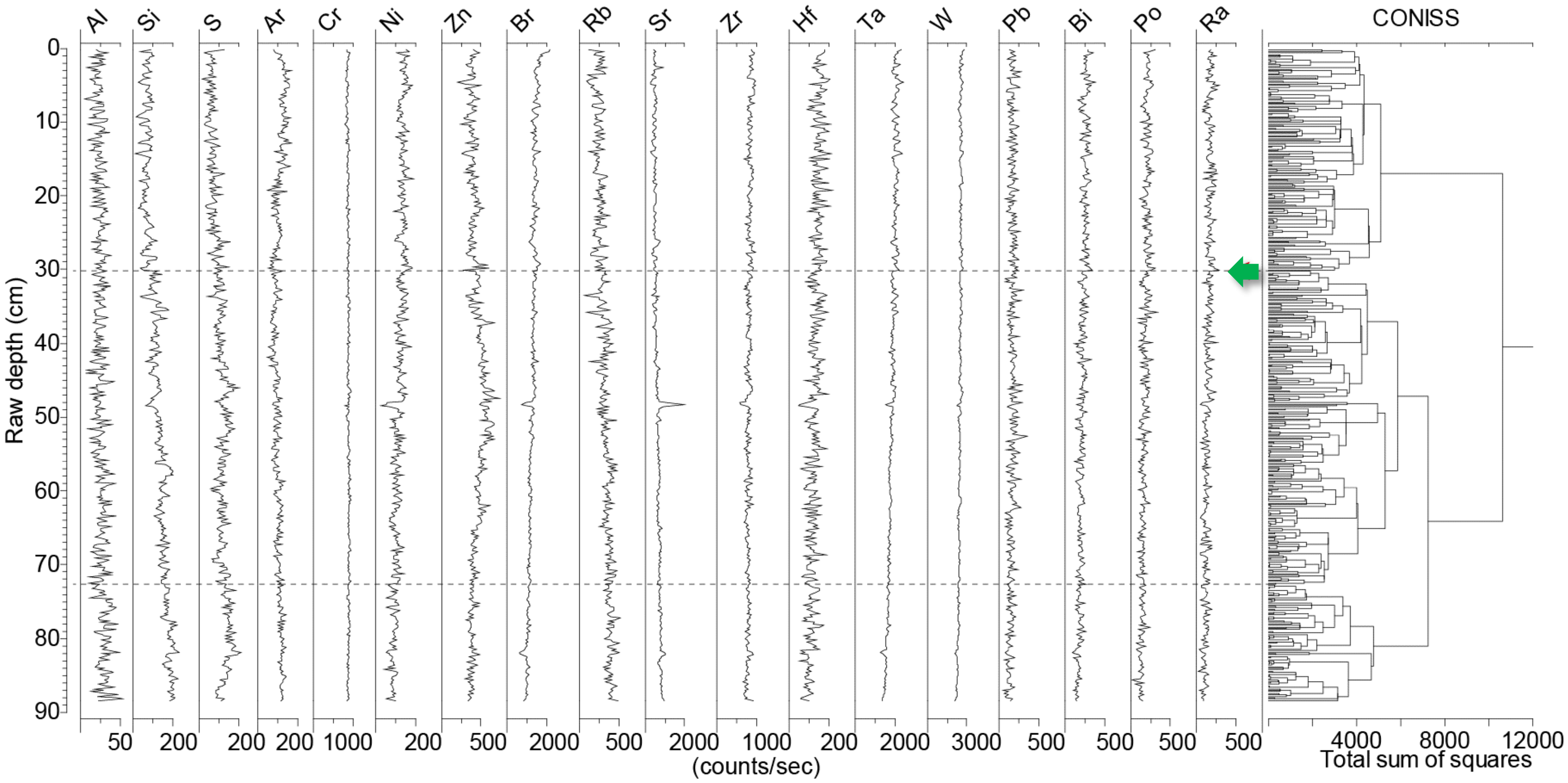

Kuwae et al. 2023

Depth profile of ITRAX-derived element contents in BMC 21 S1-4

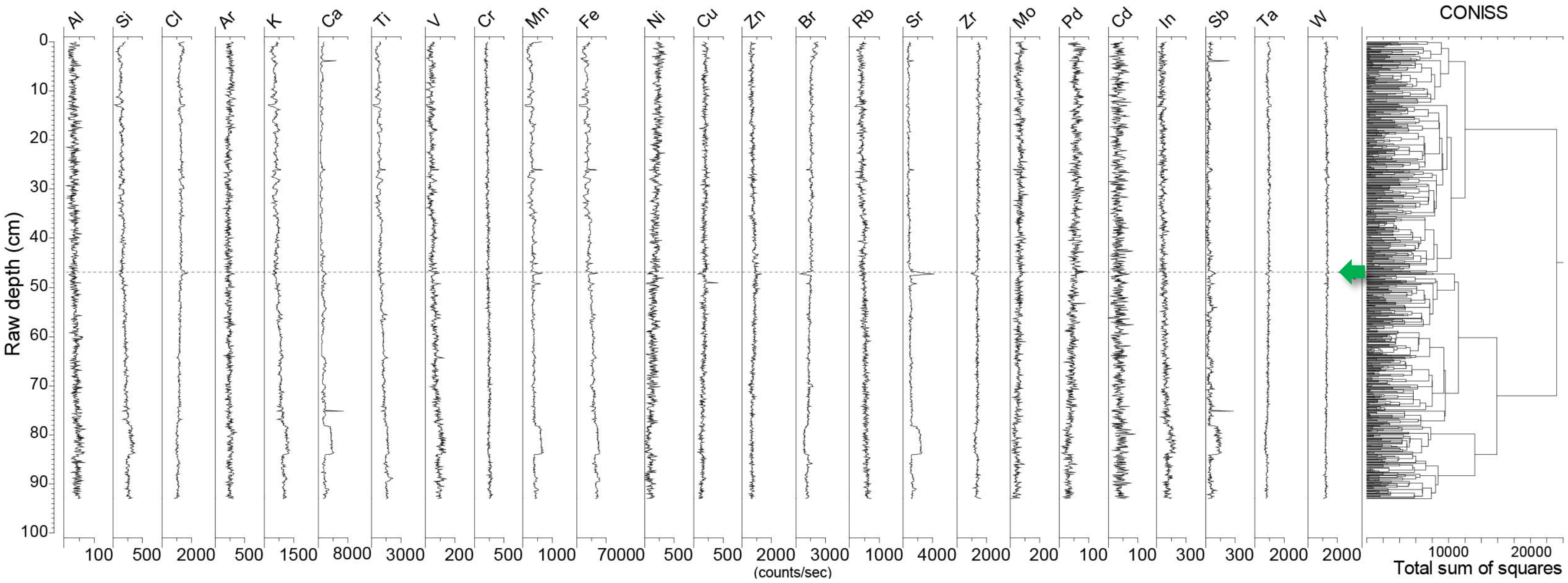

Kuwae et al. 2023

Depth profile of ITRAX-derived element contents in BMC 21 S1-5

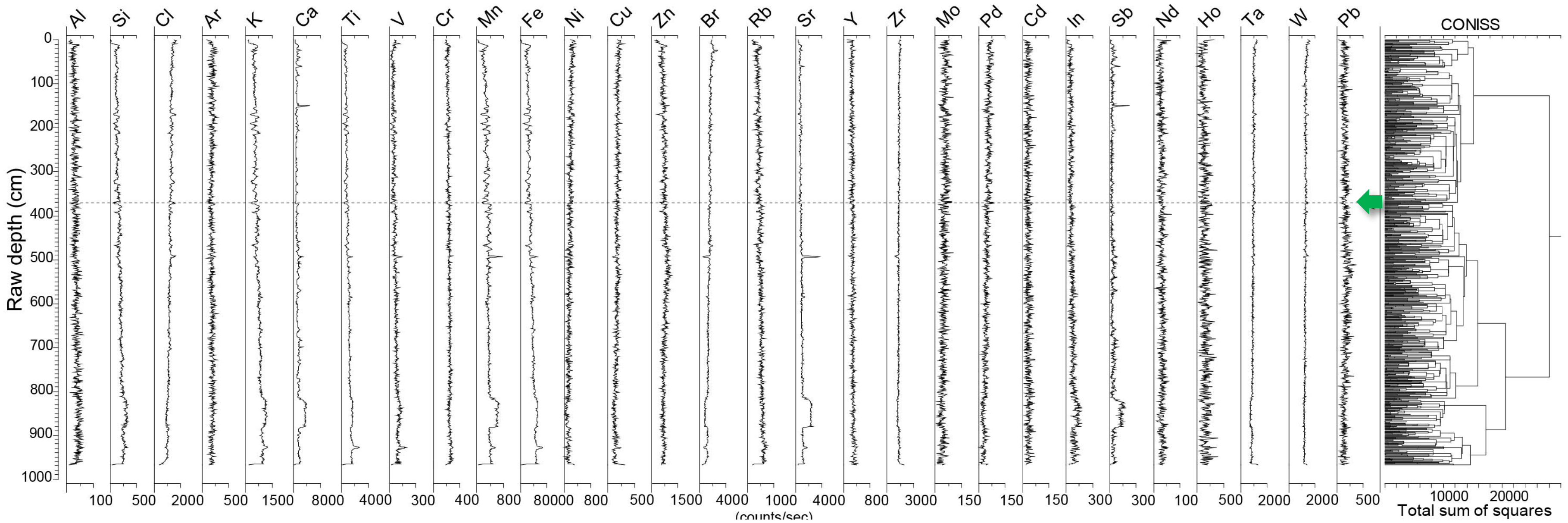

Kuwae et al. 2023

Depth profile of ITRAX-derived element contents in BG17-1

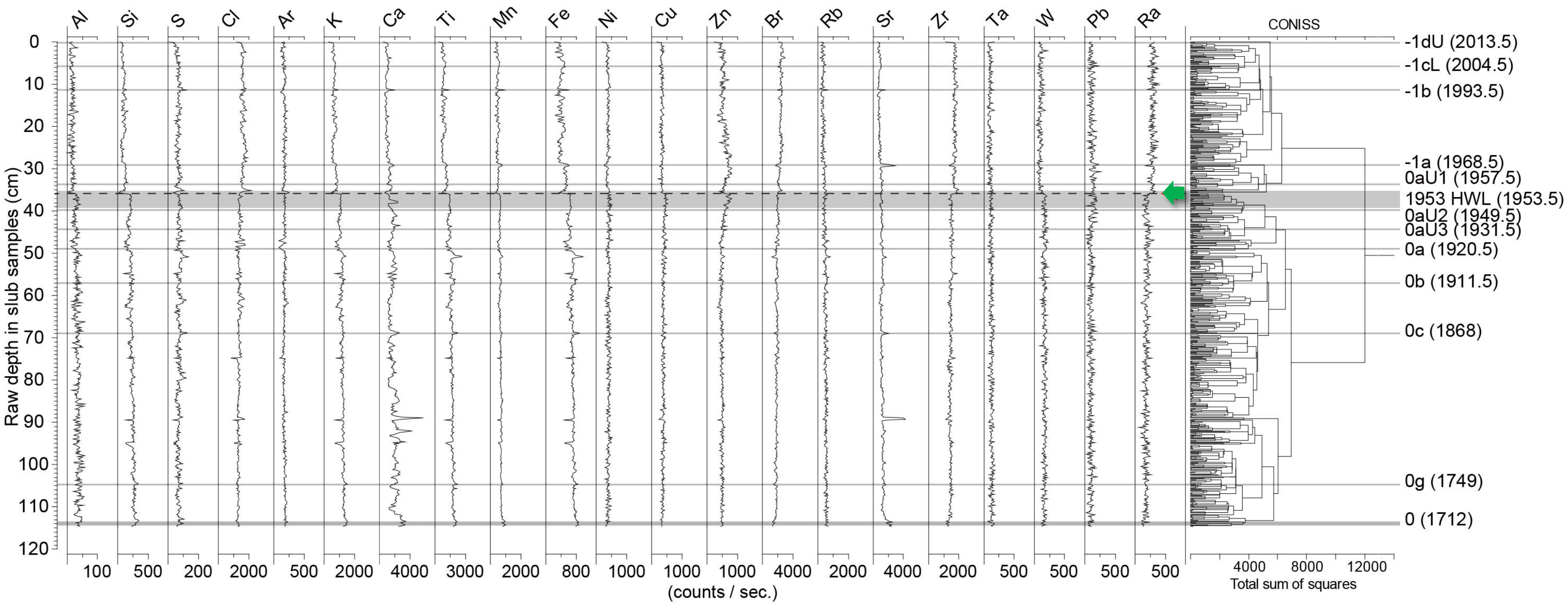

Kuwae et al. 2023

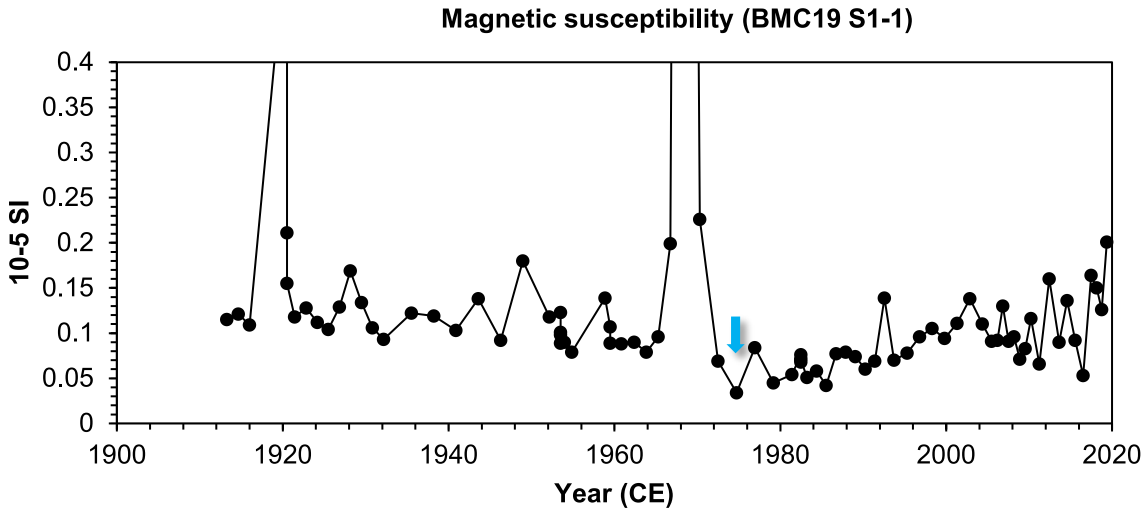

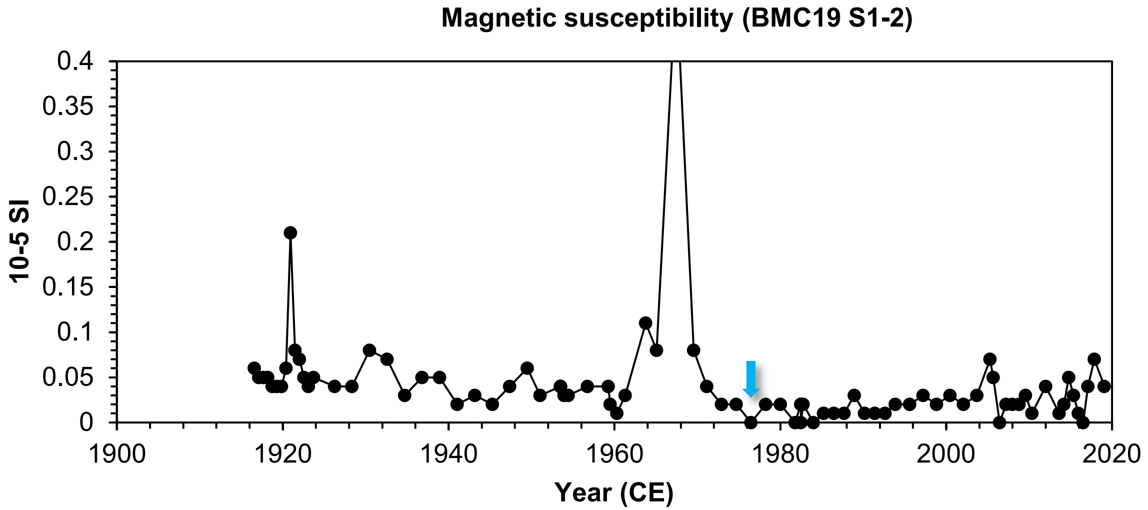

Kuwae et al. 2023

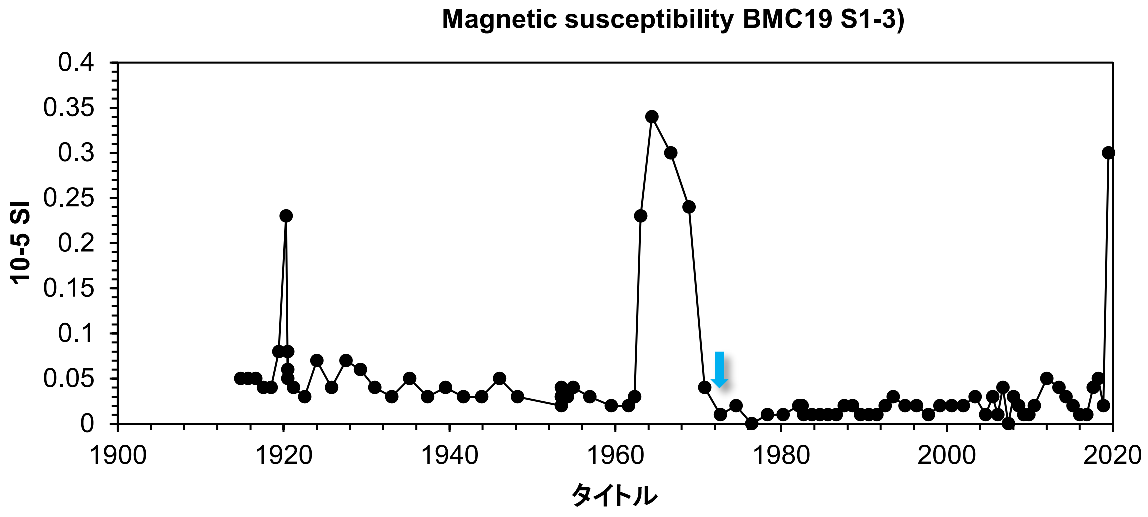

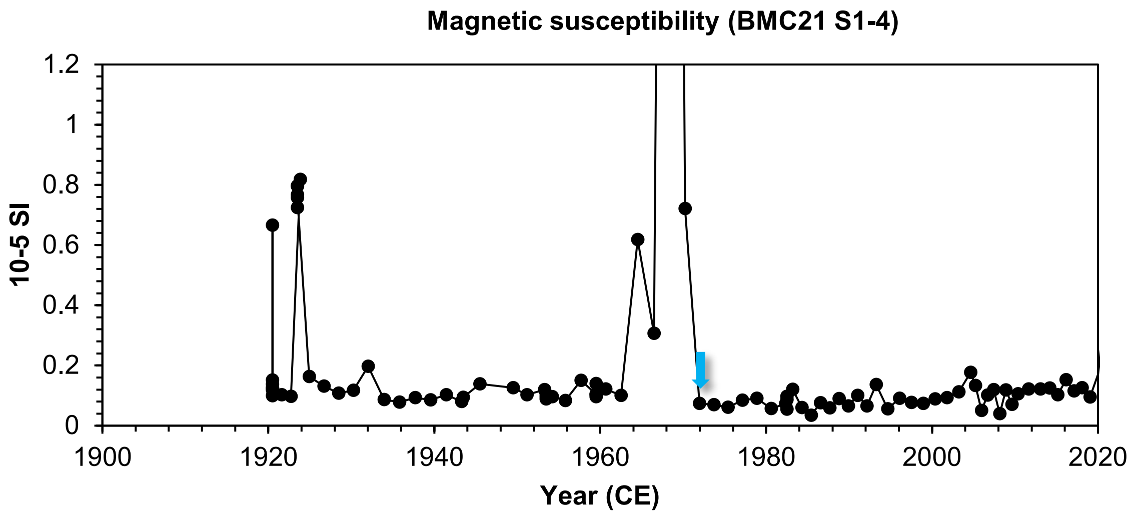

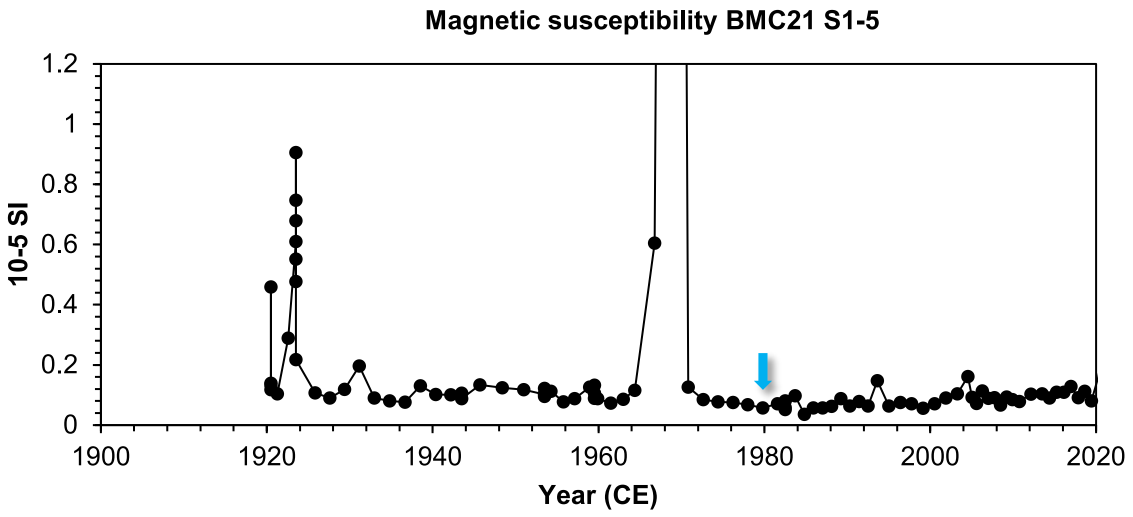

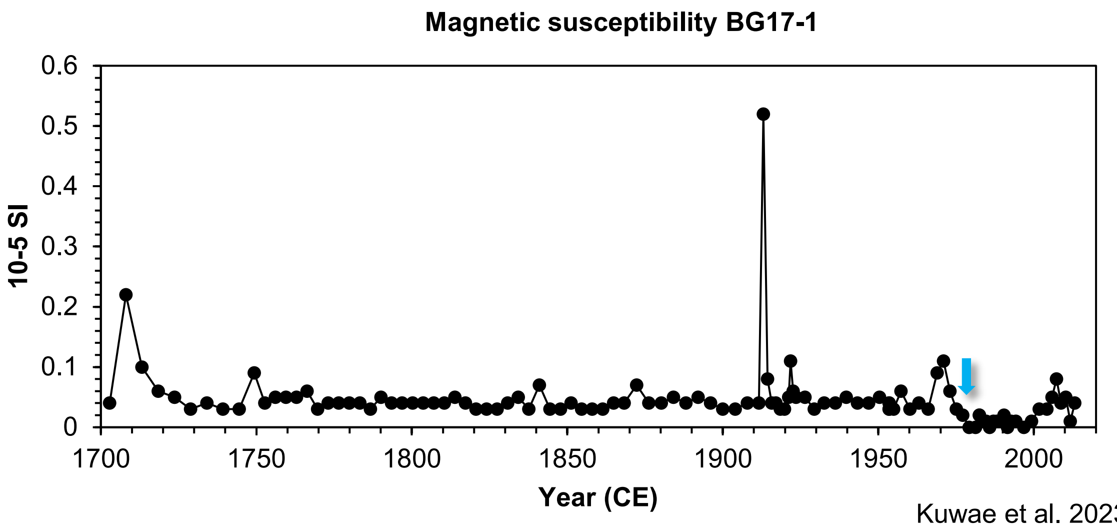

Kuwae et al. 2023

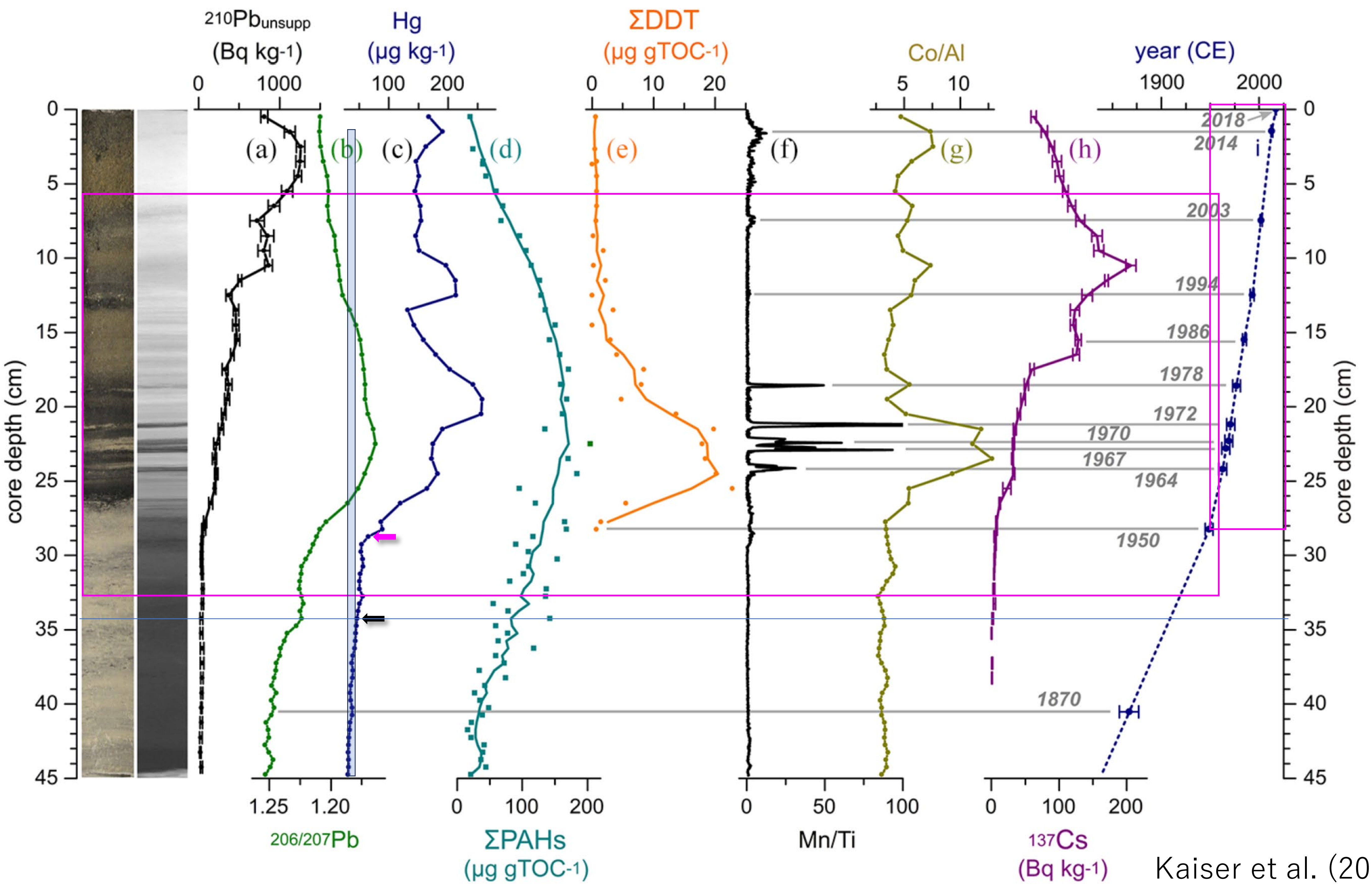

Kaiser et al. (2023)

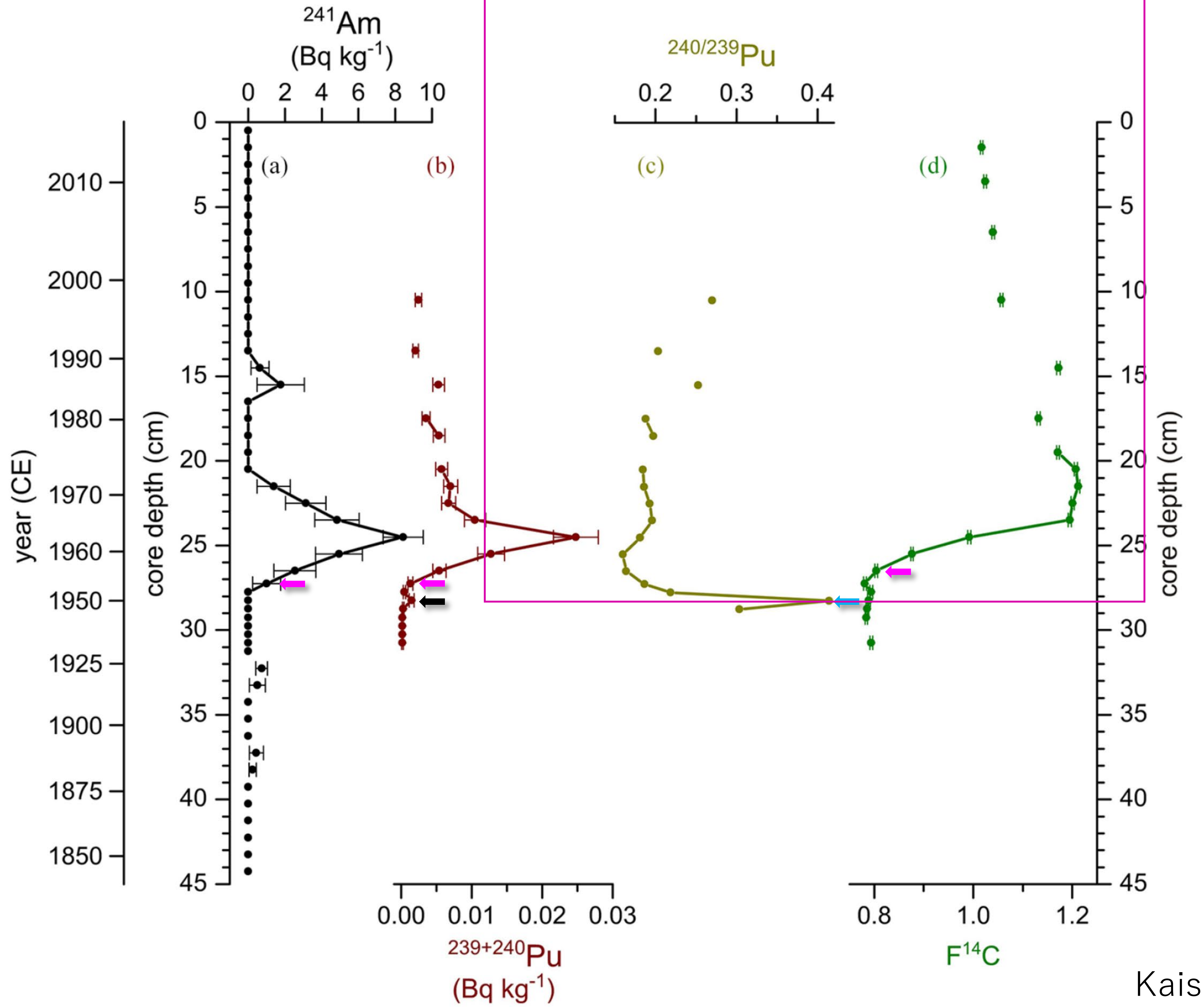

Kaiser et al. (2023)

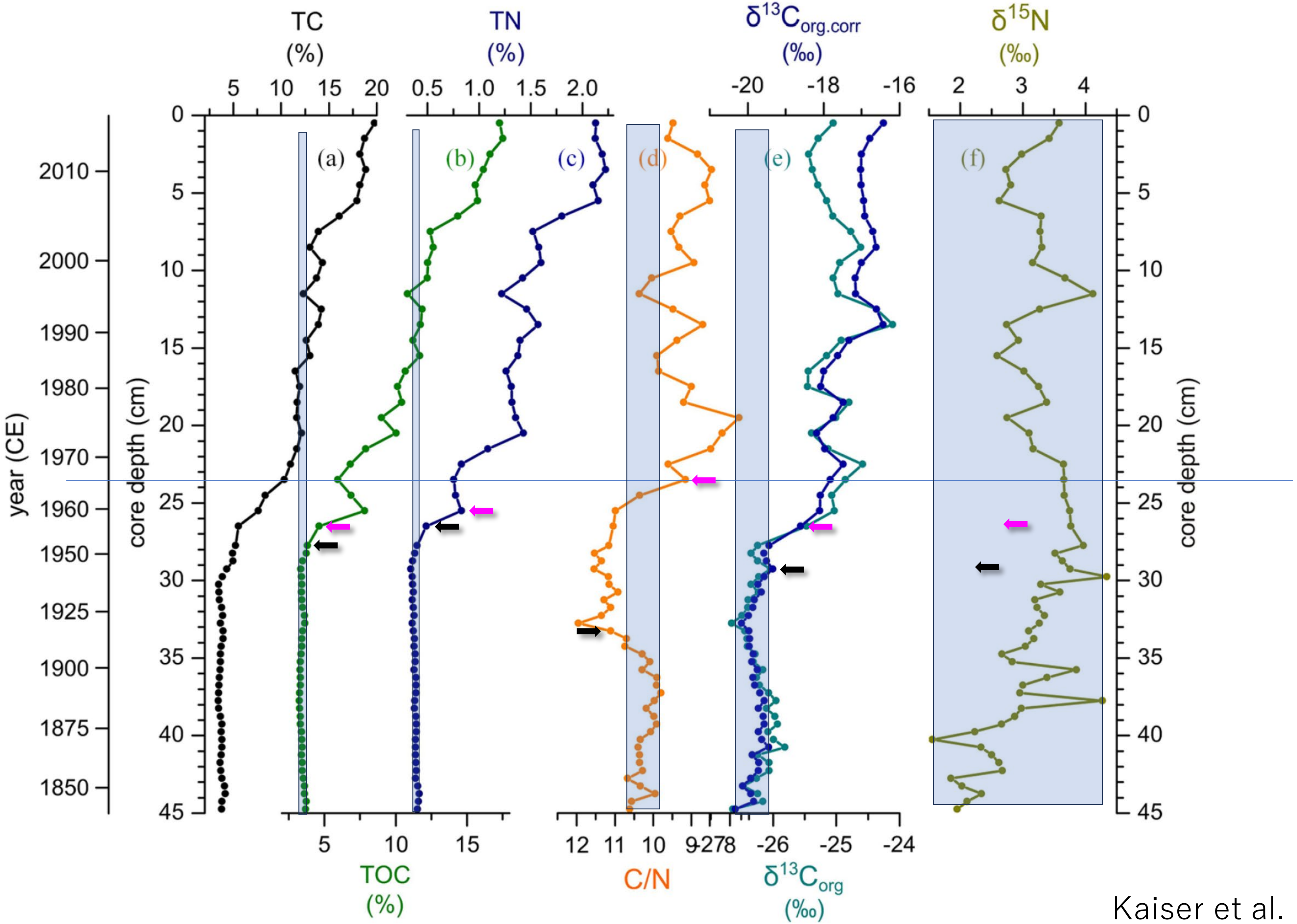

Kaiser et al. (2023)

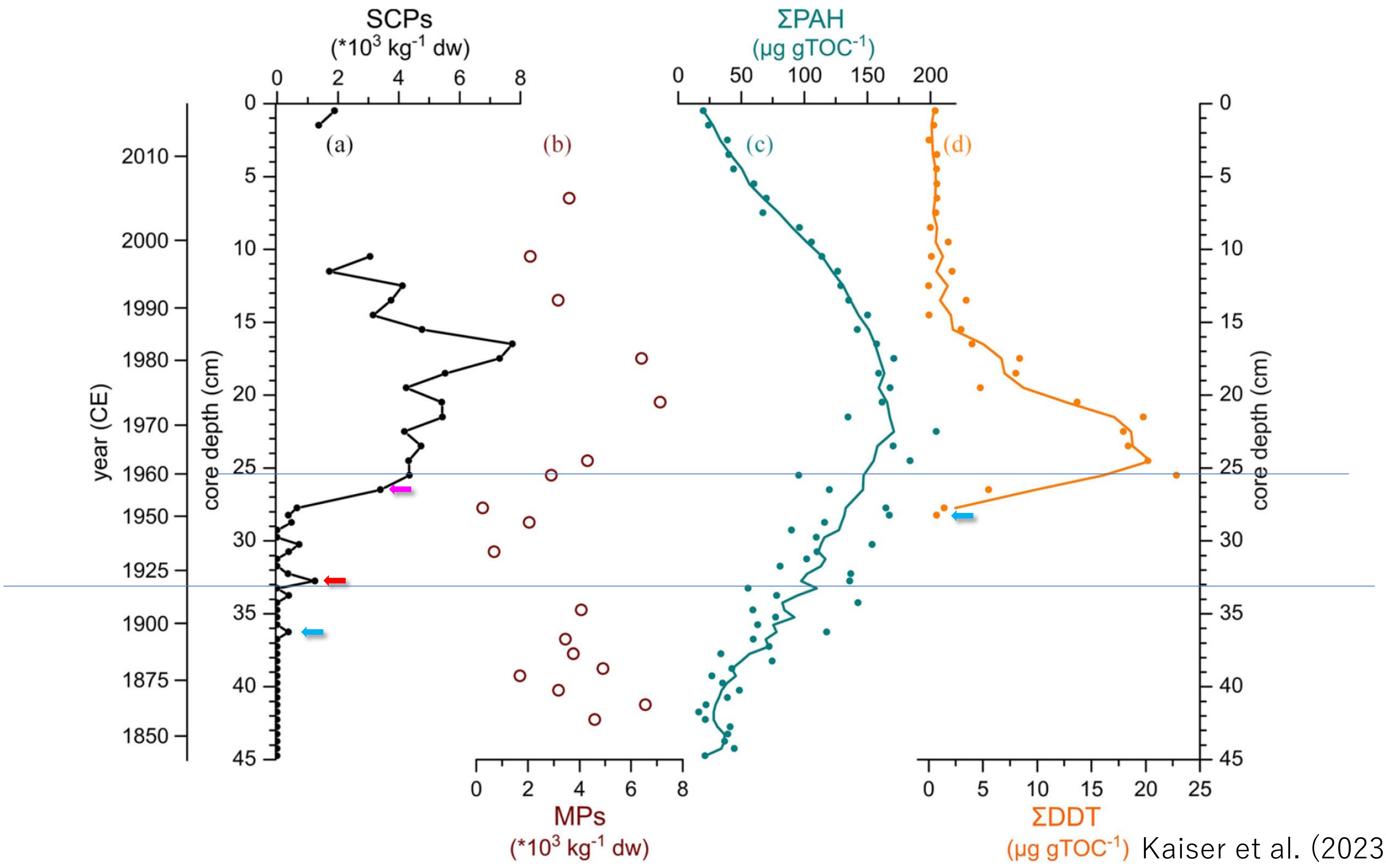

Kaiser et al. (2023)

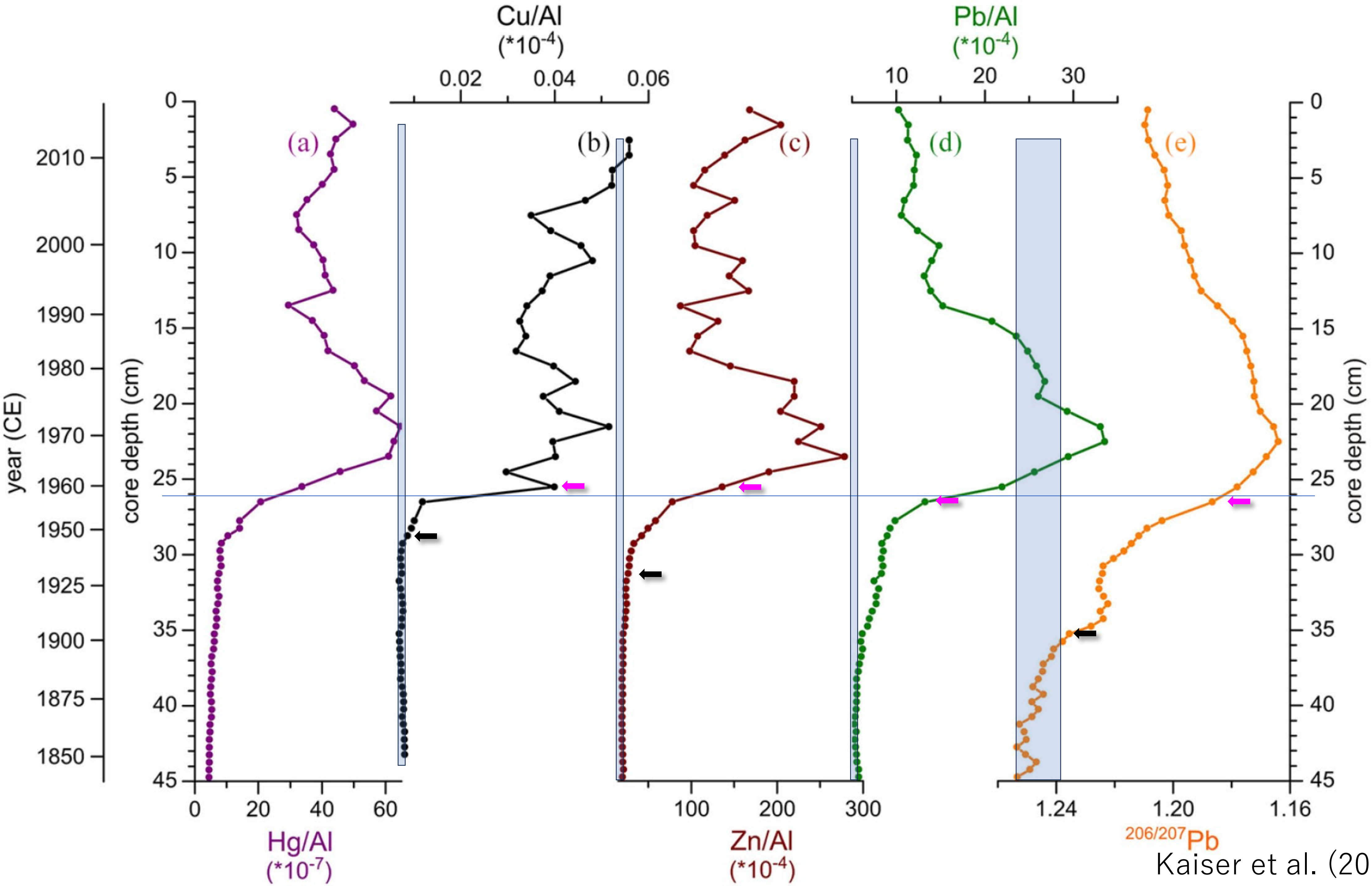

Kaiser et al. (2023)

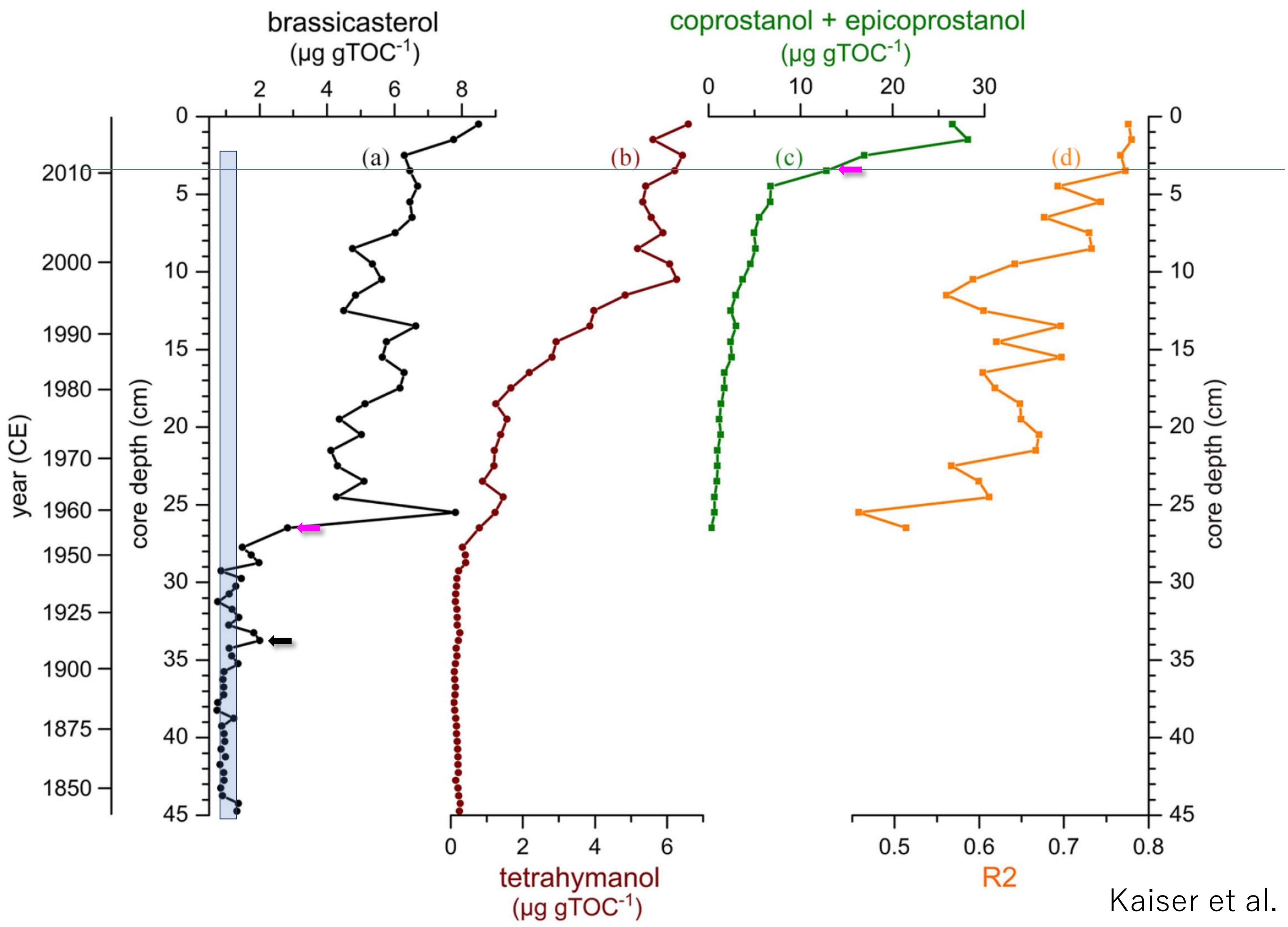

Kaiser et al. (2023)

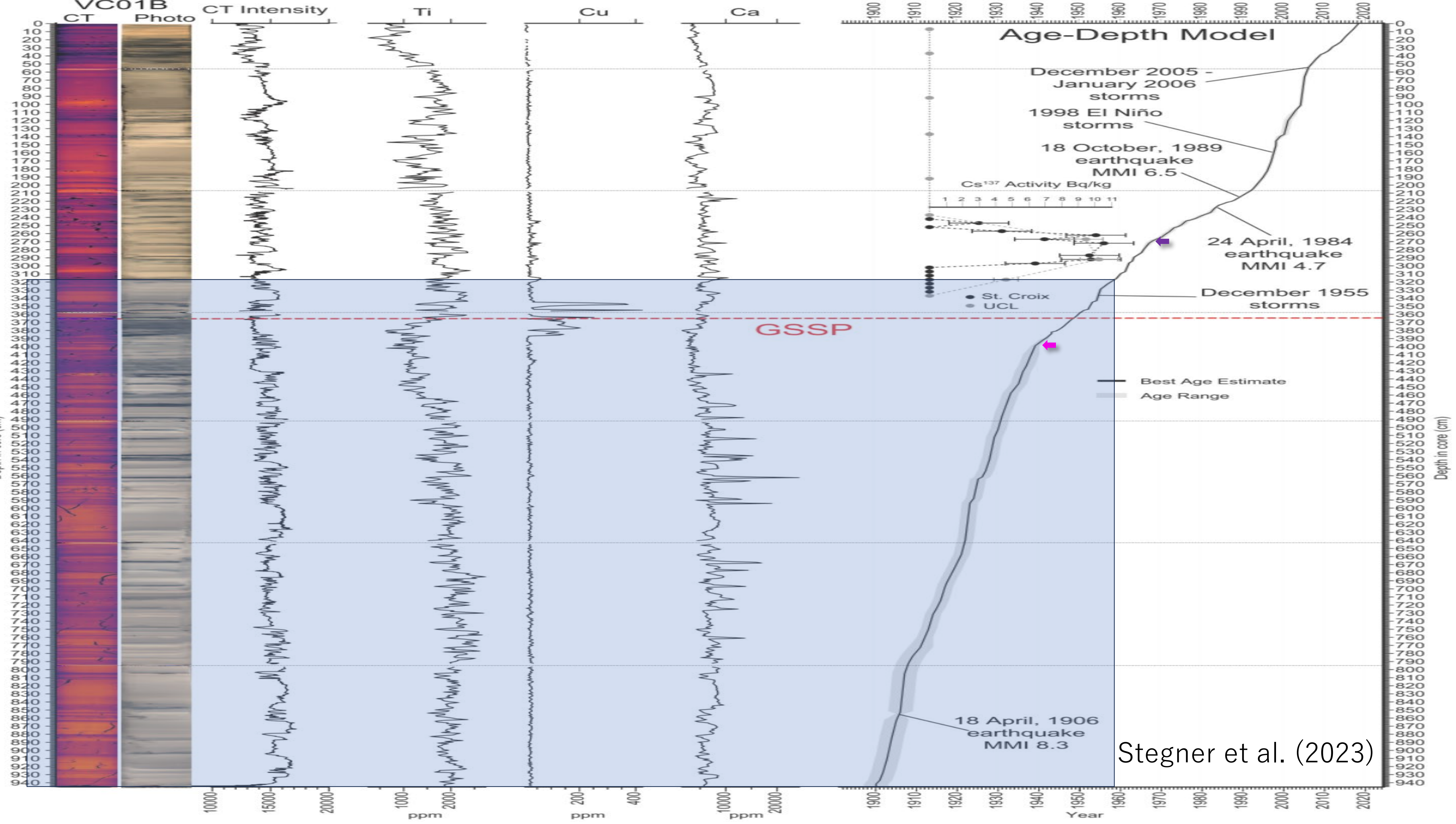

Stegner et al. (2023)

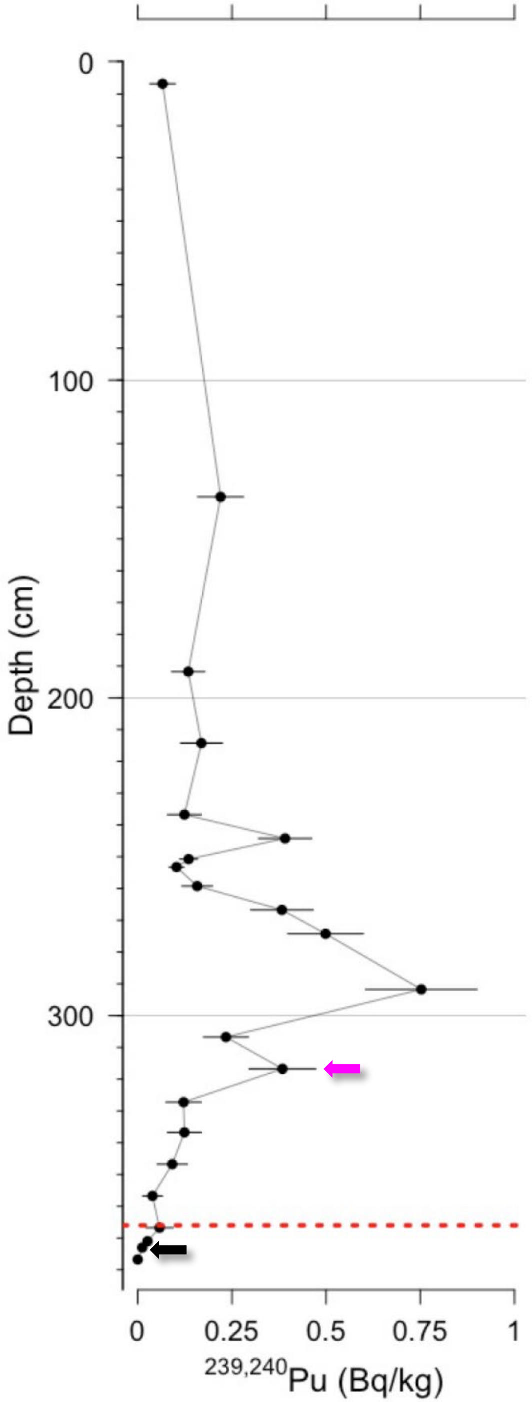

Stegner et al. (2023)

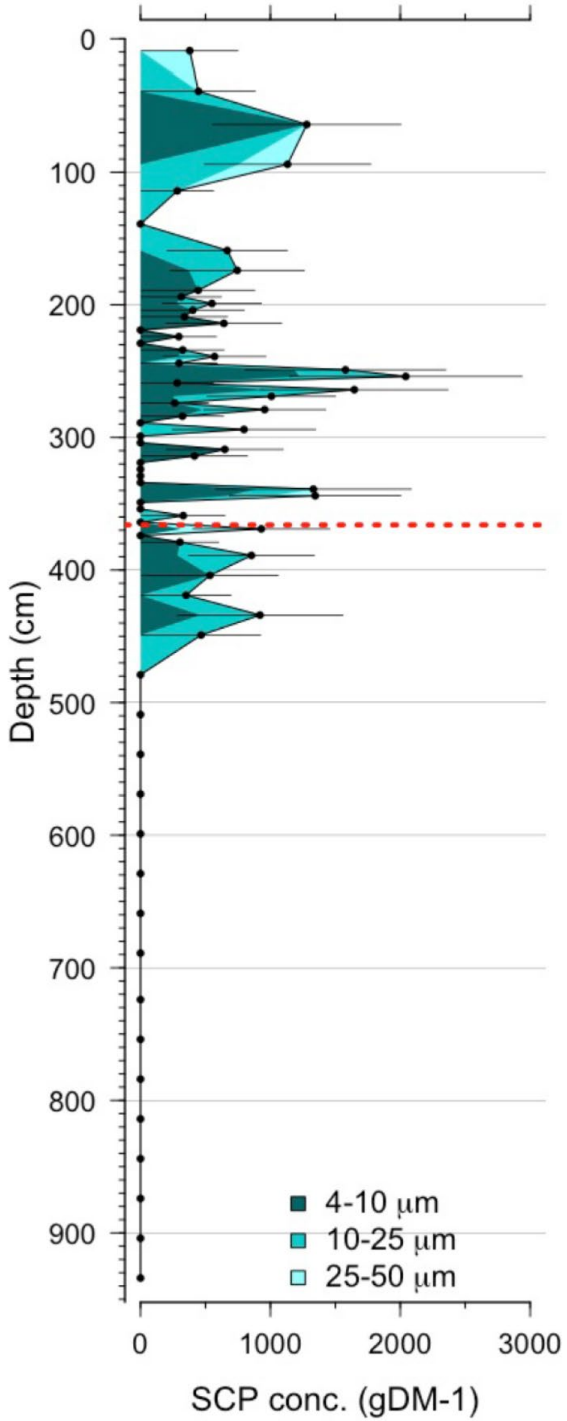

Stegner et al. (2023)

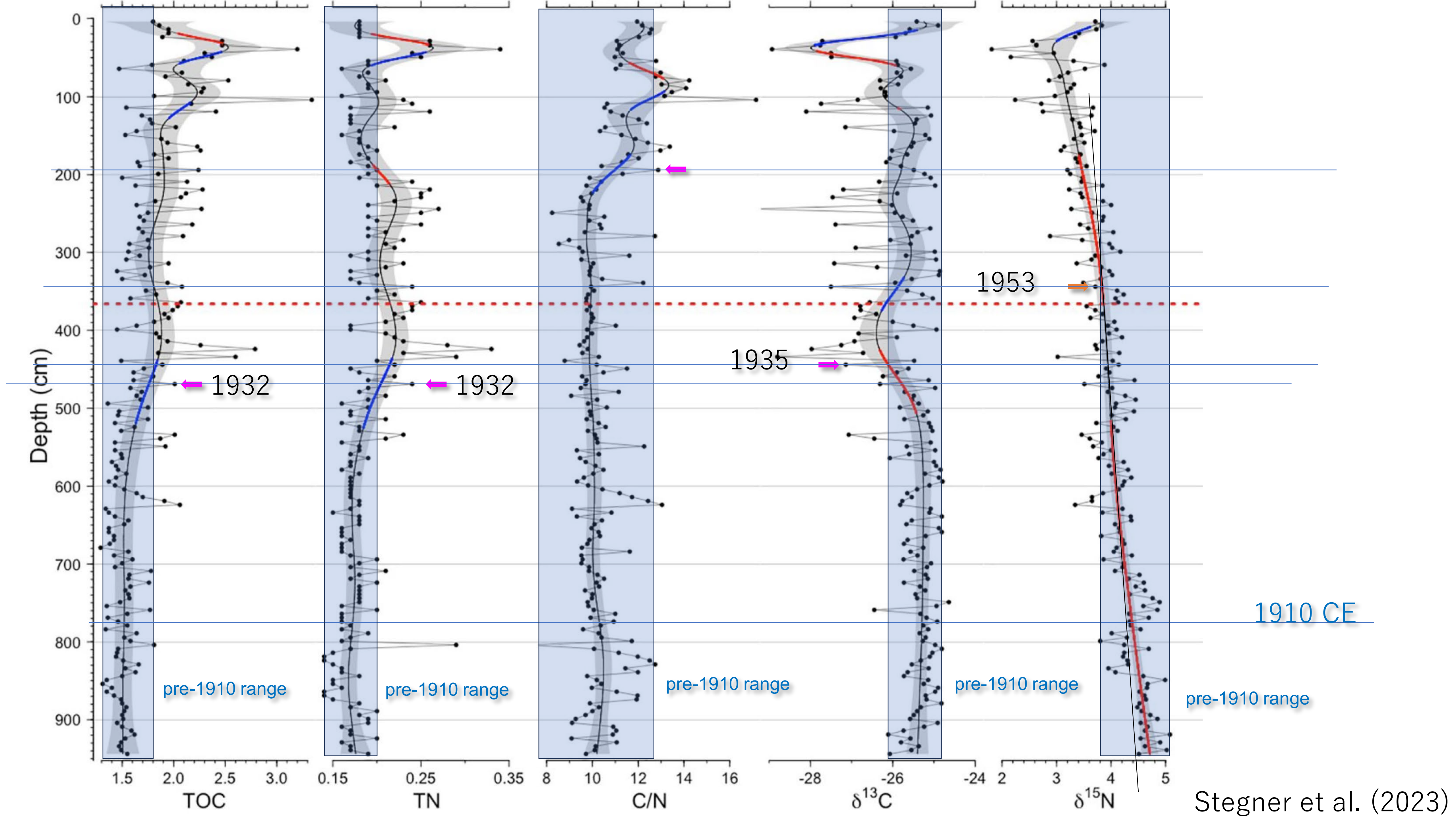

Stegner et al. (2023)

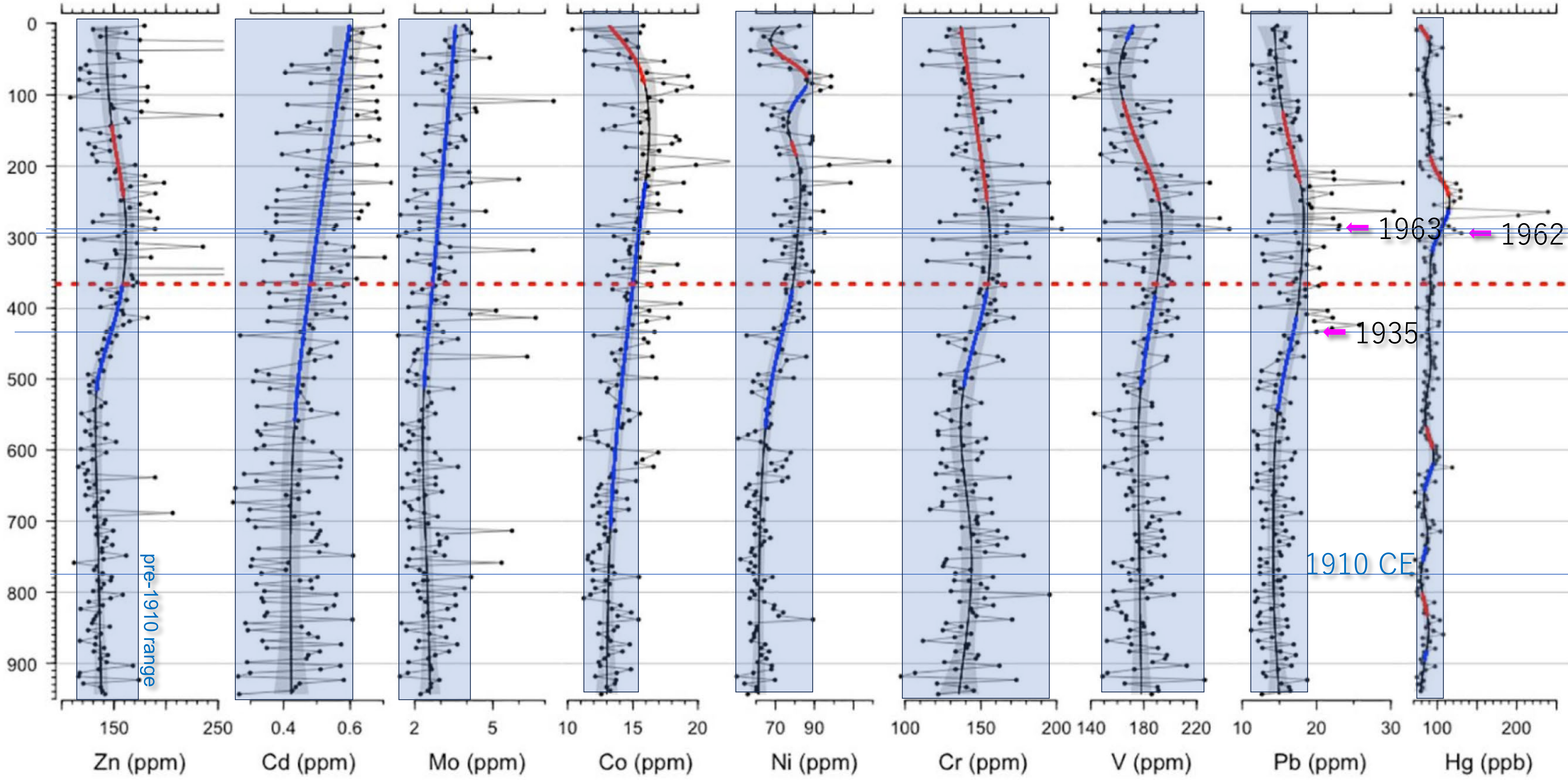

Stegner et al. (2023)

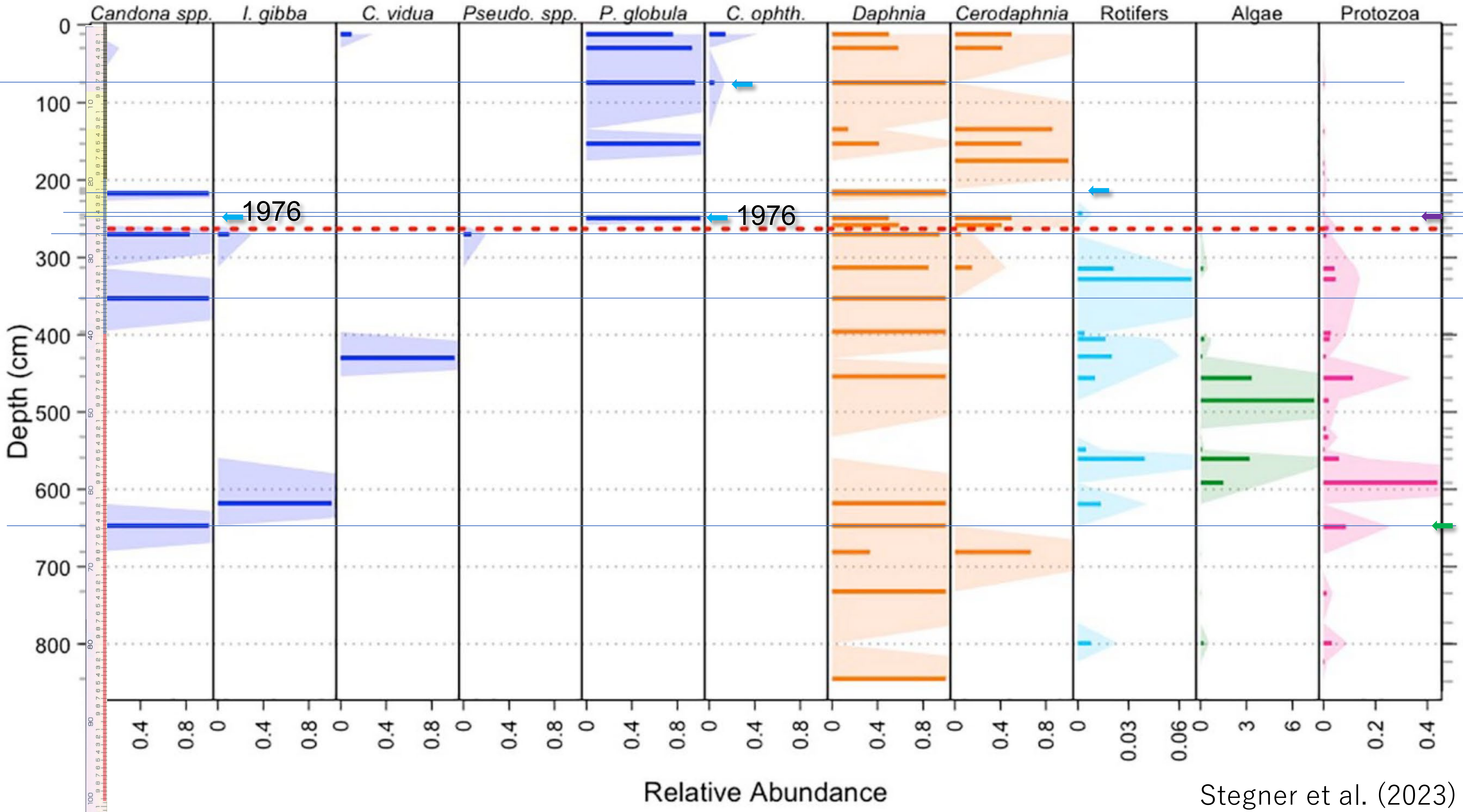

Stegner et al. (2023)

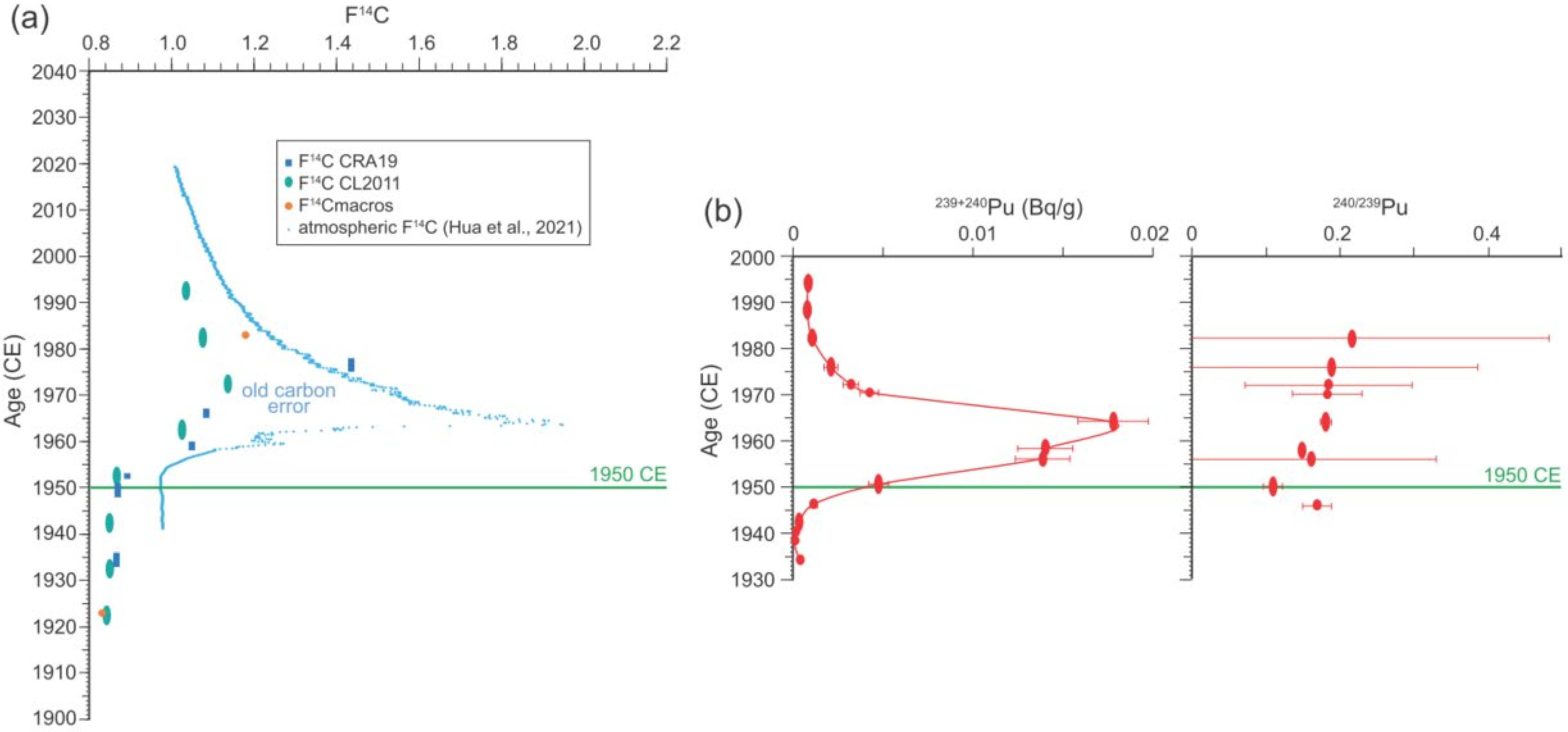

McCarthy et al. (2023)

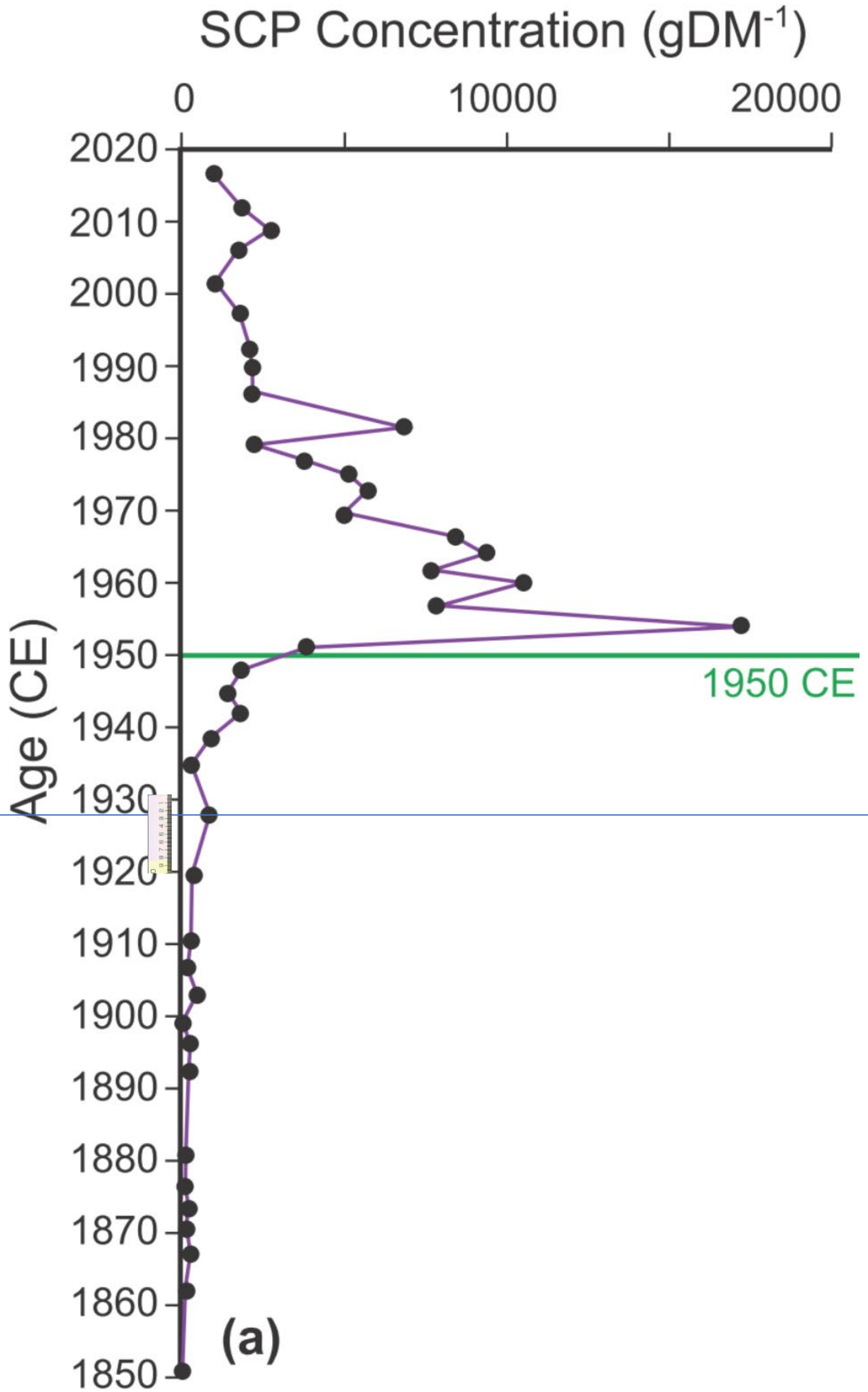

McCarthy et al. (2023)

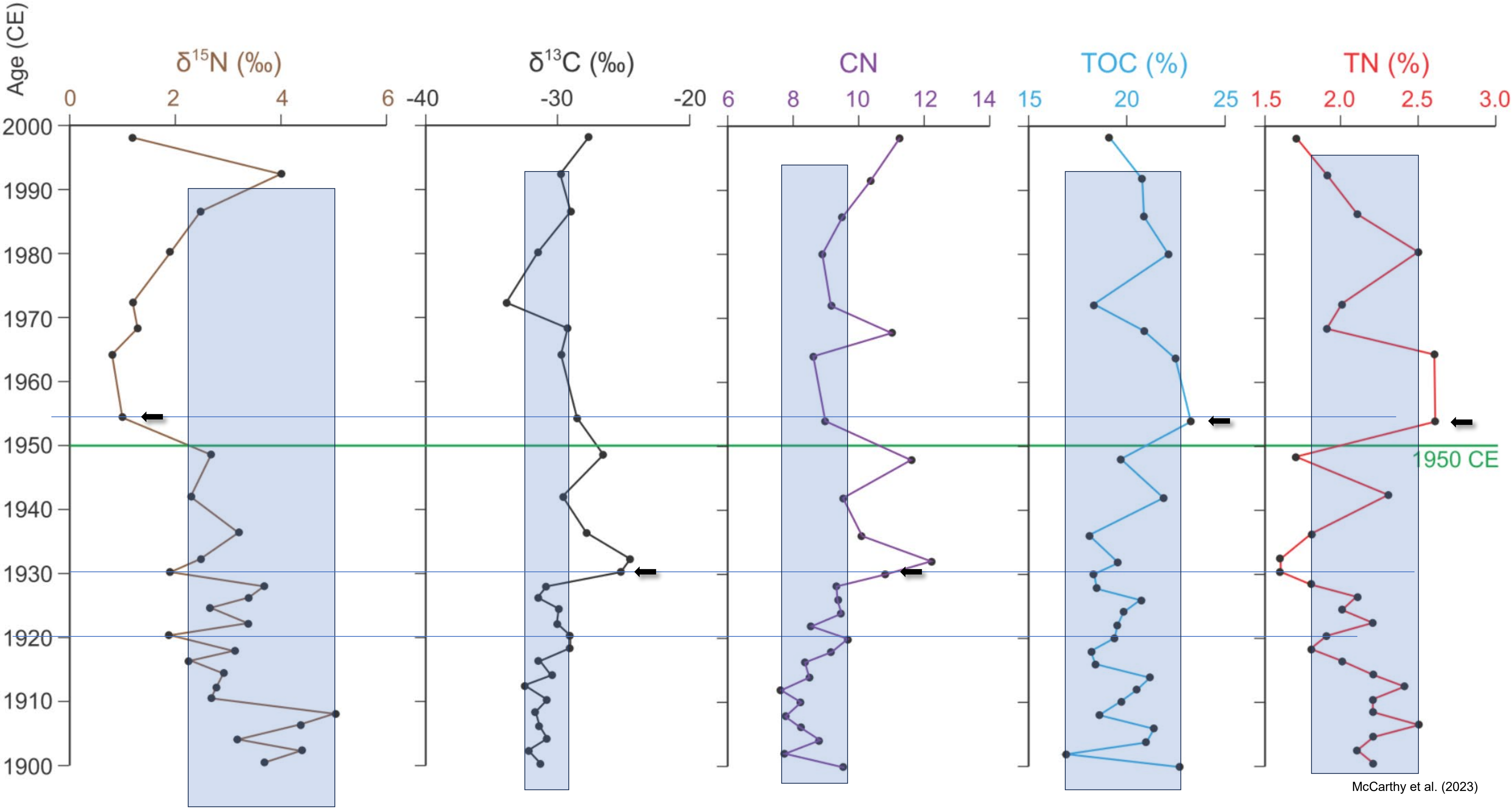
Age (CE)
δ15N (‰)
δ13C (‰)
CN
TOC (%)
TN (%)
1950 CE
McCarthy et al. (2023)

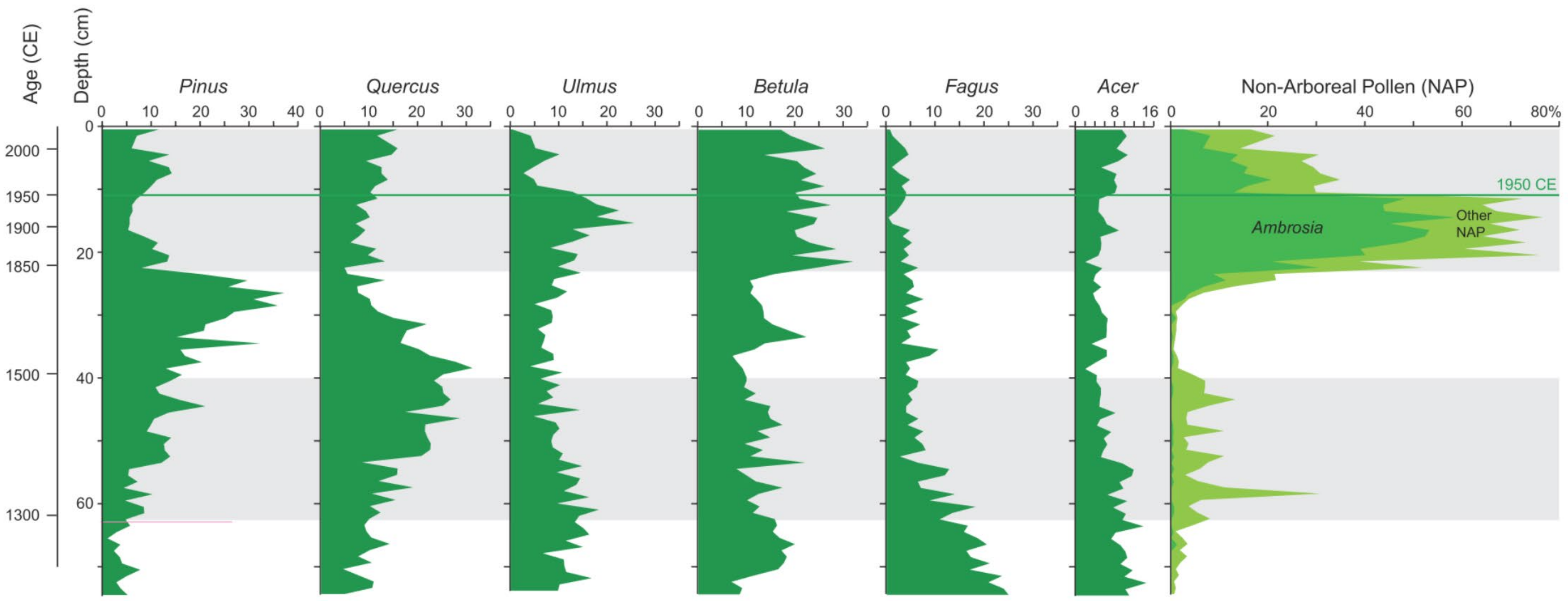
Age (CE)
Depth (cm)
Pinus
Quercus
Ulmus
Betula
Fagus
Acer
Non-Arboreal Pollen (NAP)
0
10
20
30
40
8
16
60
80%
2000
1950
1900
1850
1500
1300
1950 CE
Ambrosia
Other
NAP

core CL-2011

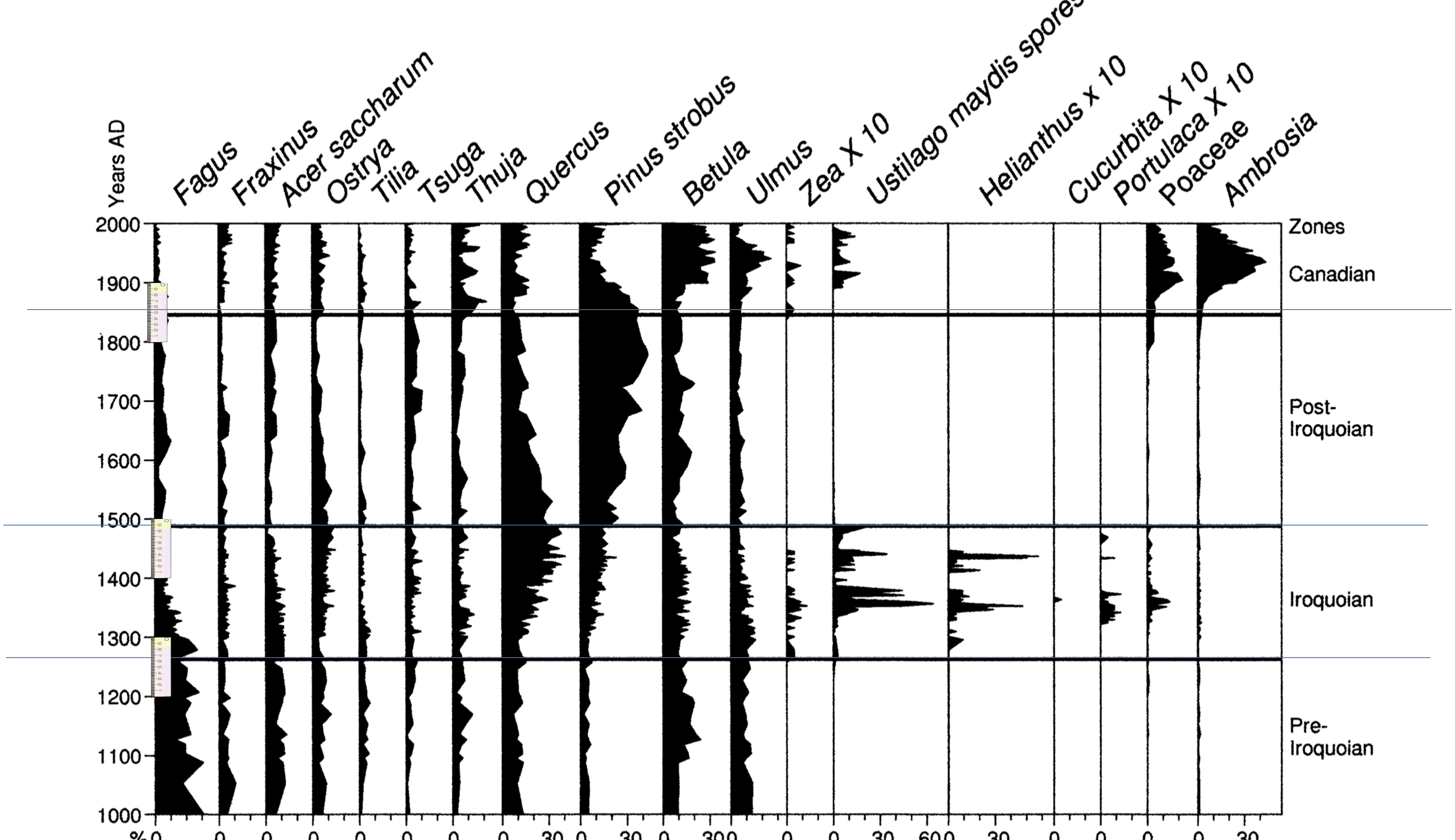

McAndrews and Turton (2010)

Fungal spore compositions

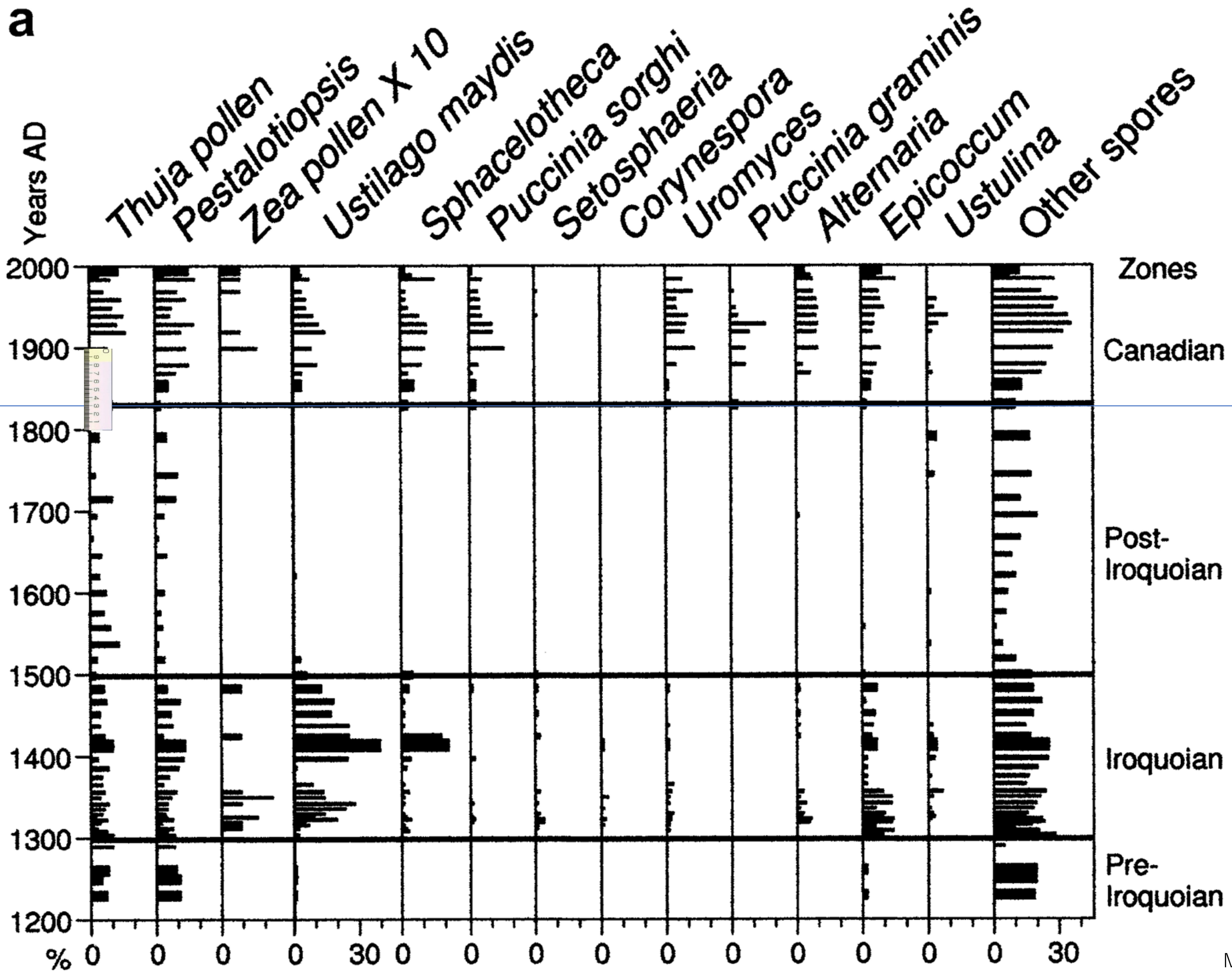

McAndrews and Turton (2010)

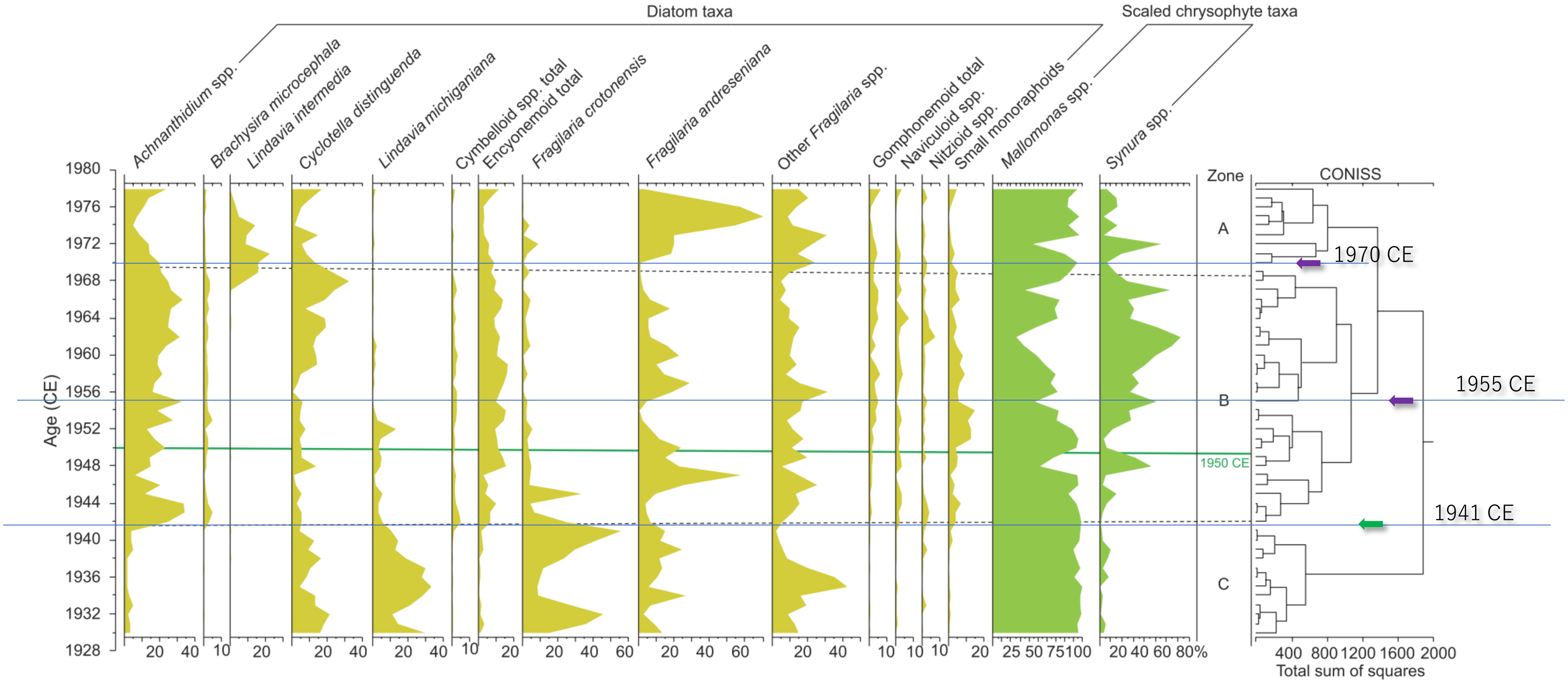

McCarthy et al. (2023)

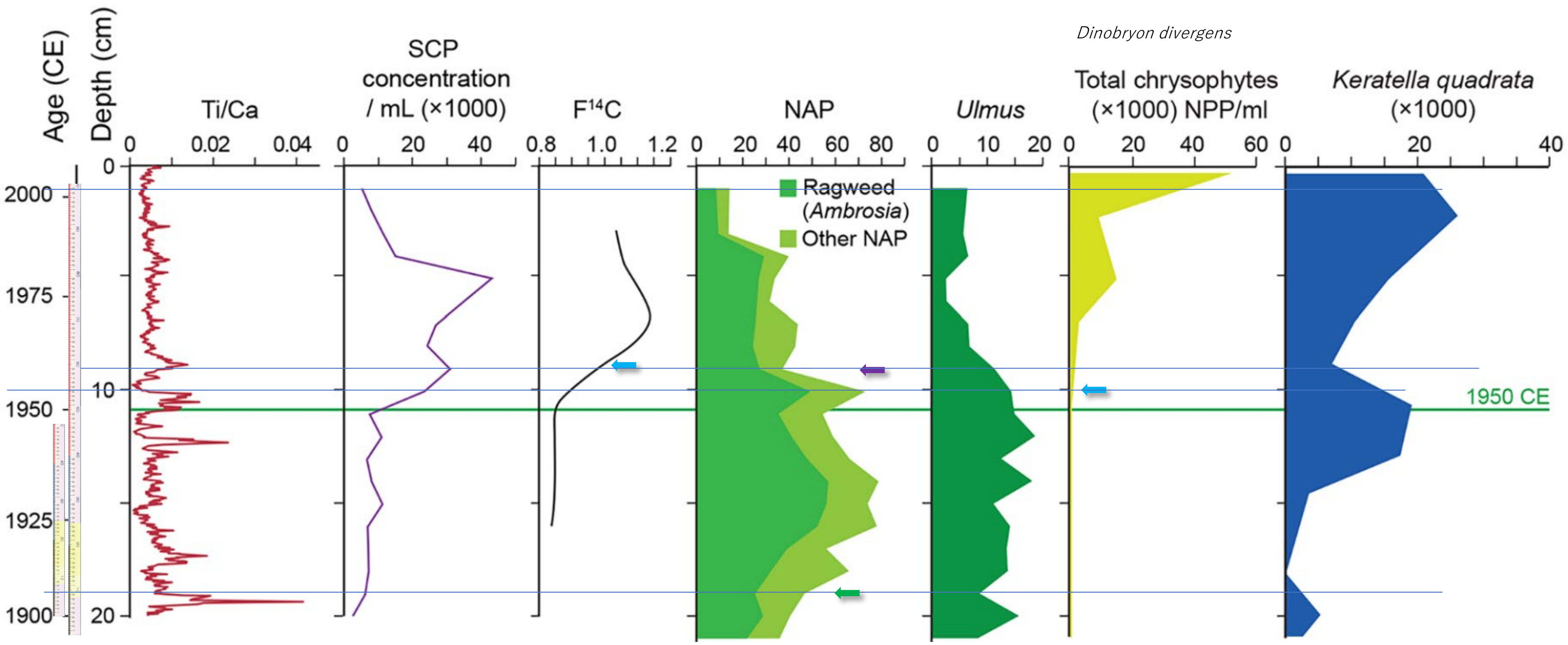

McCarthy et al. (2023)

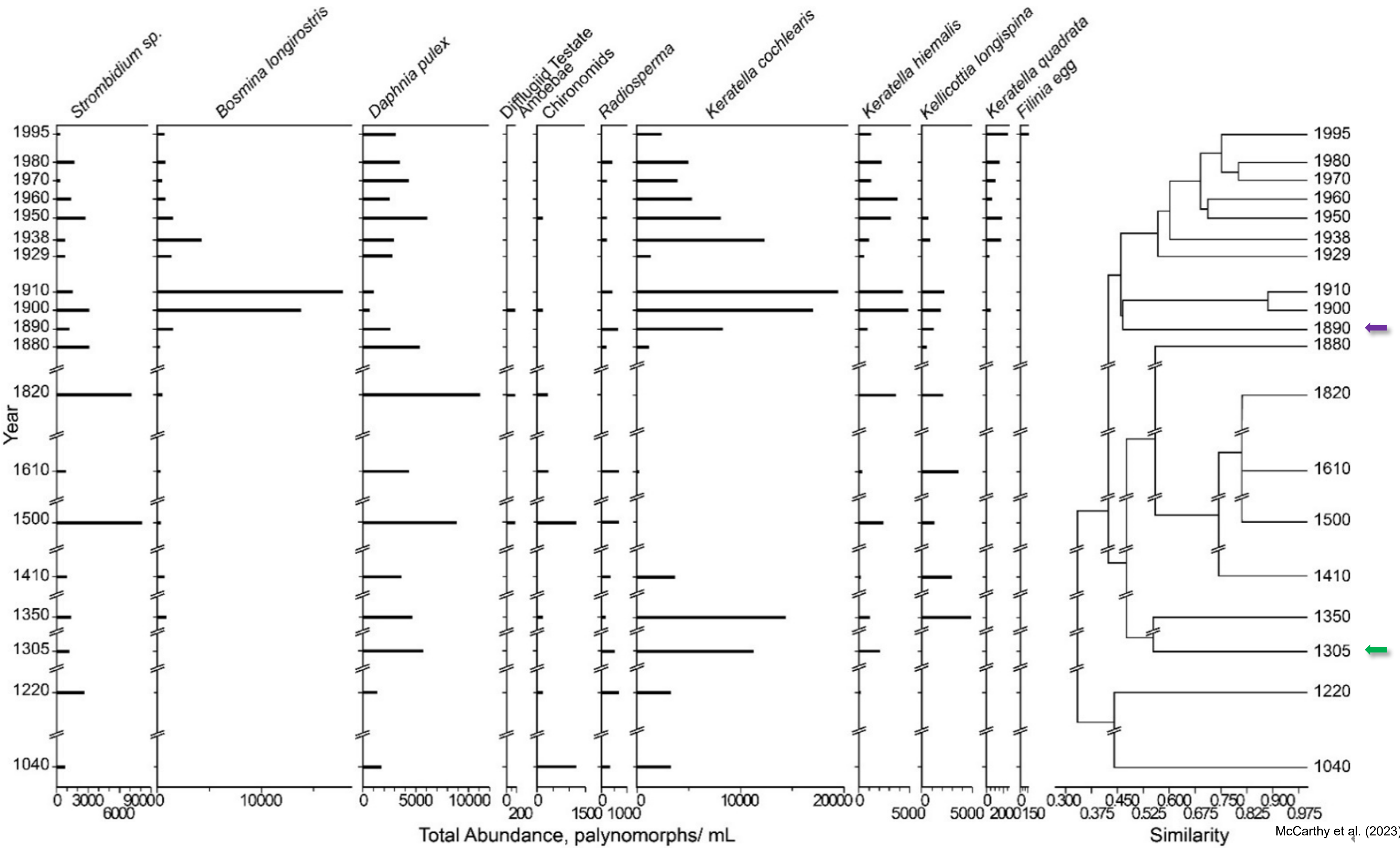

McCarthy et al. (2023)

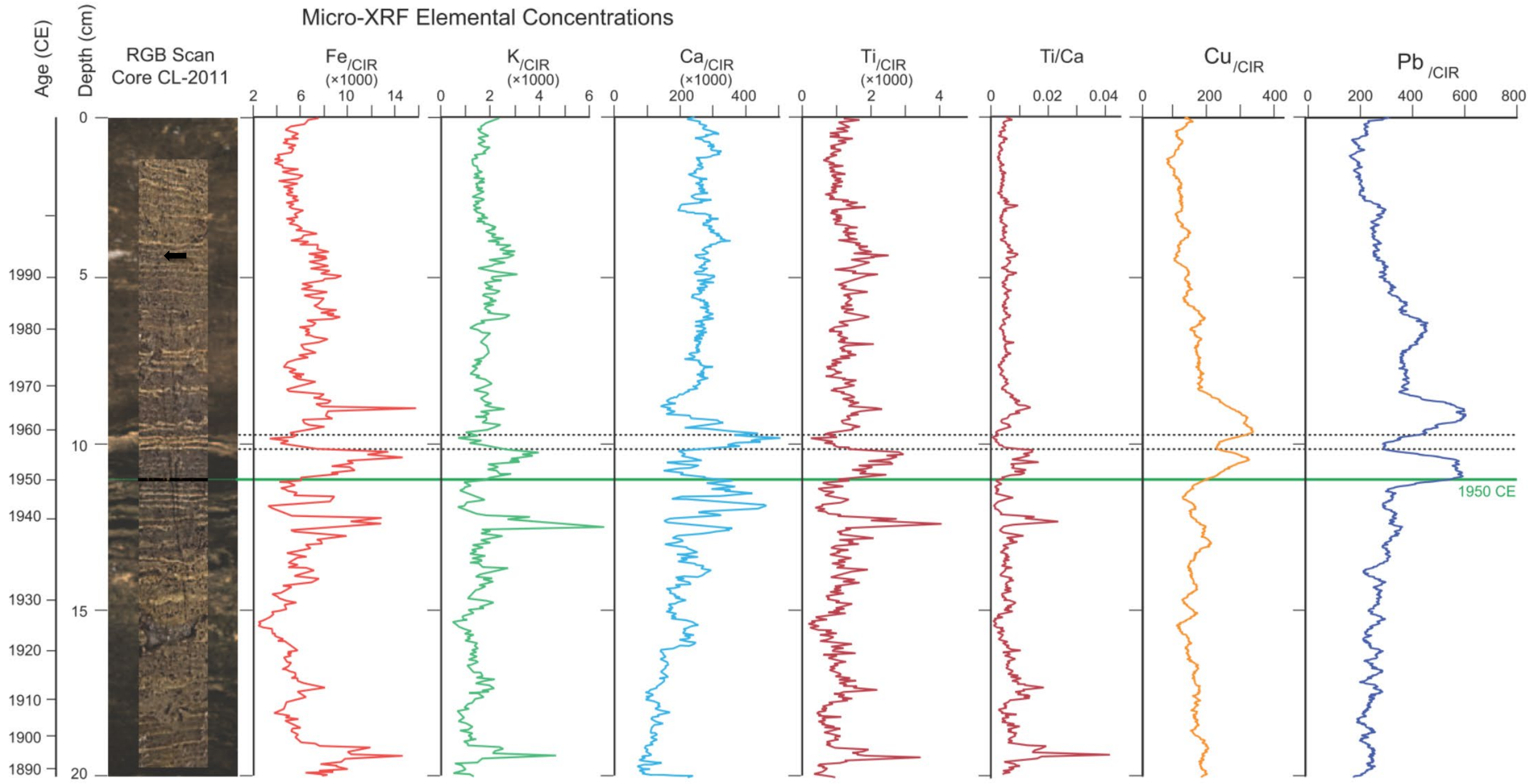

McCarthy et al. (2023)

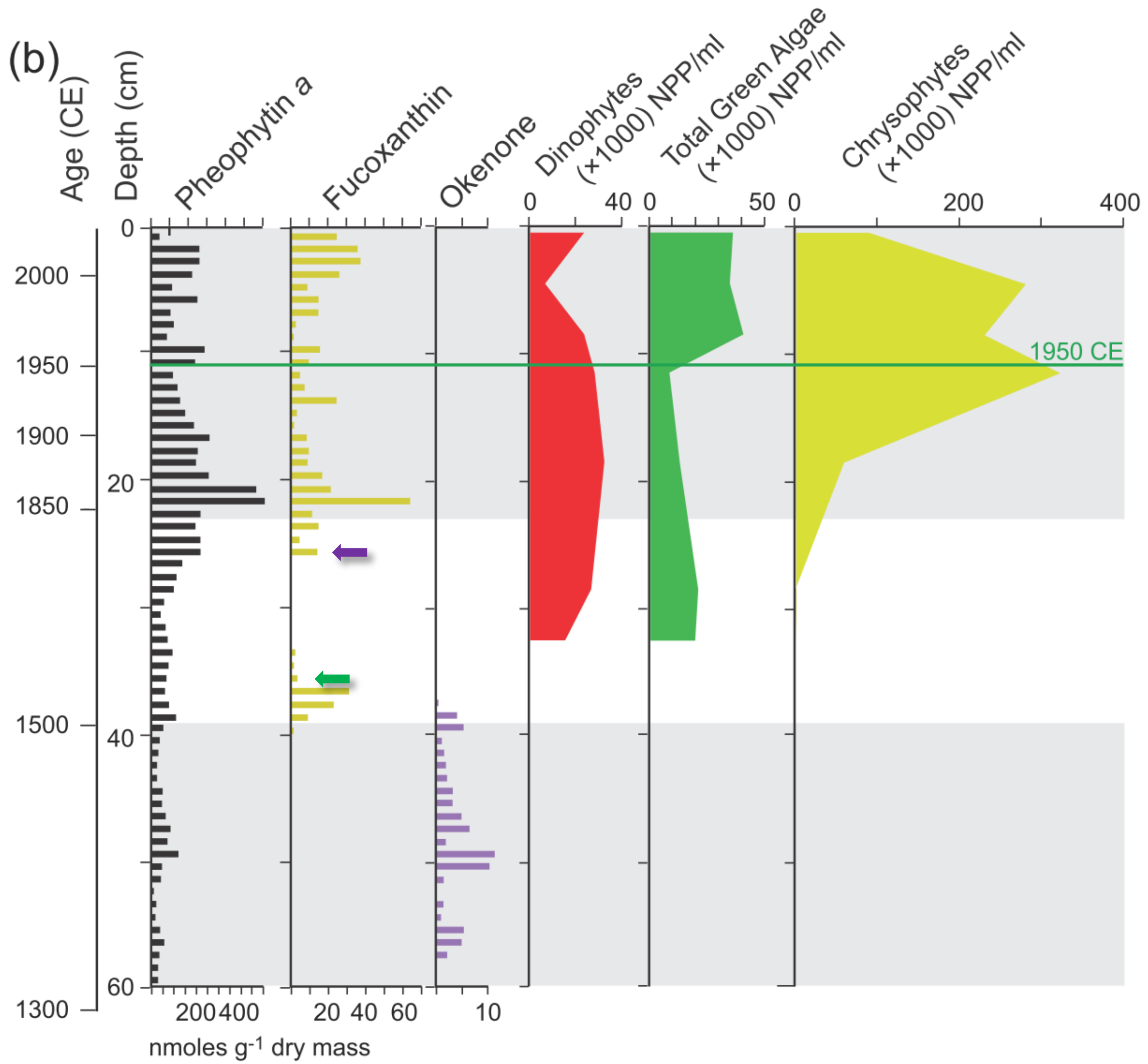

McCarthy et al. (2023)

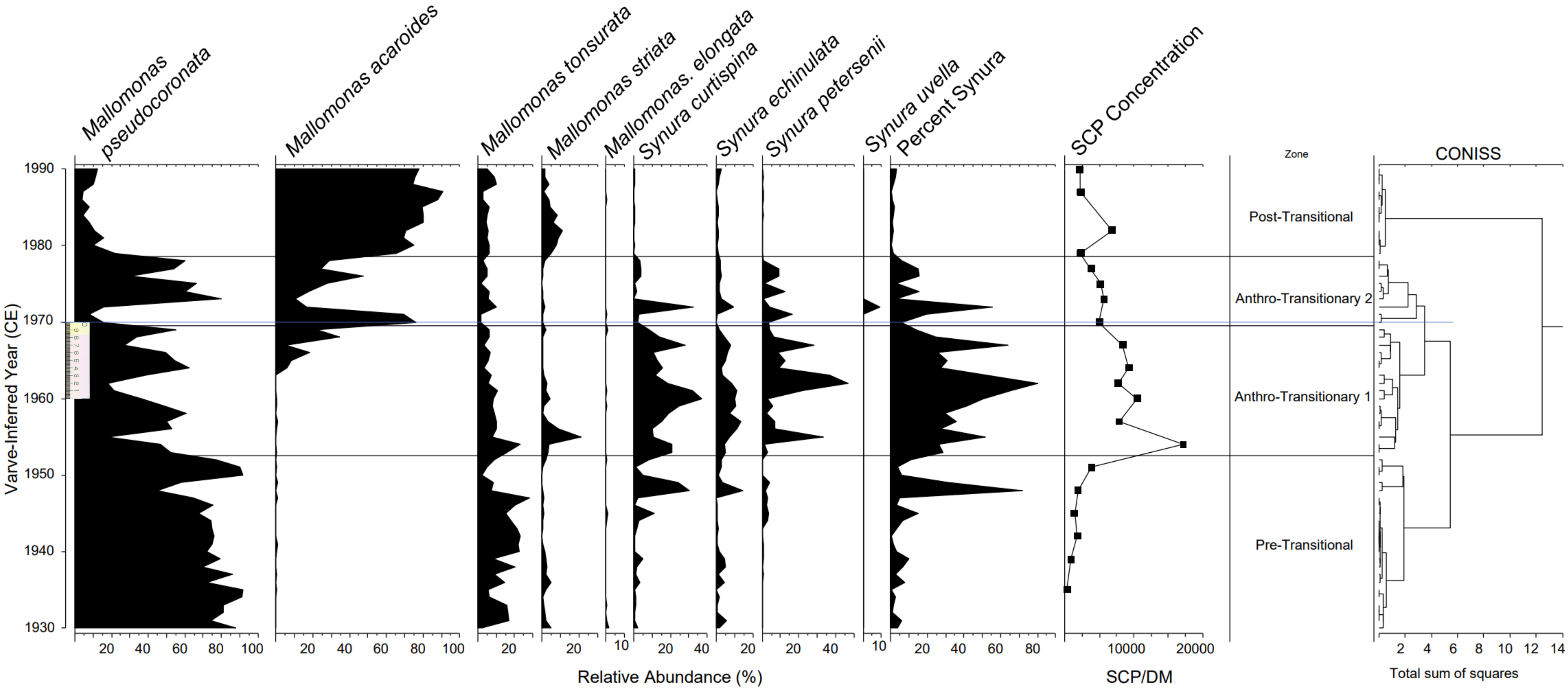

Marshall et al. (2023)

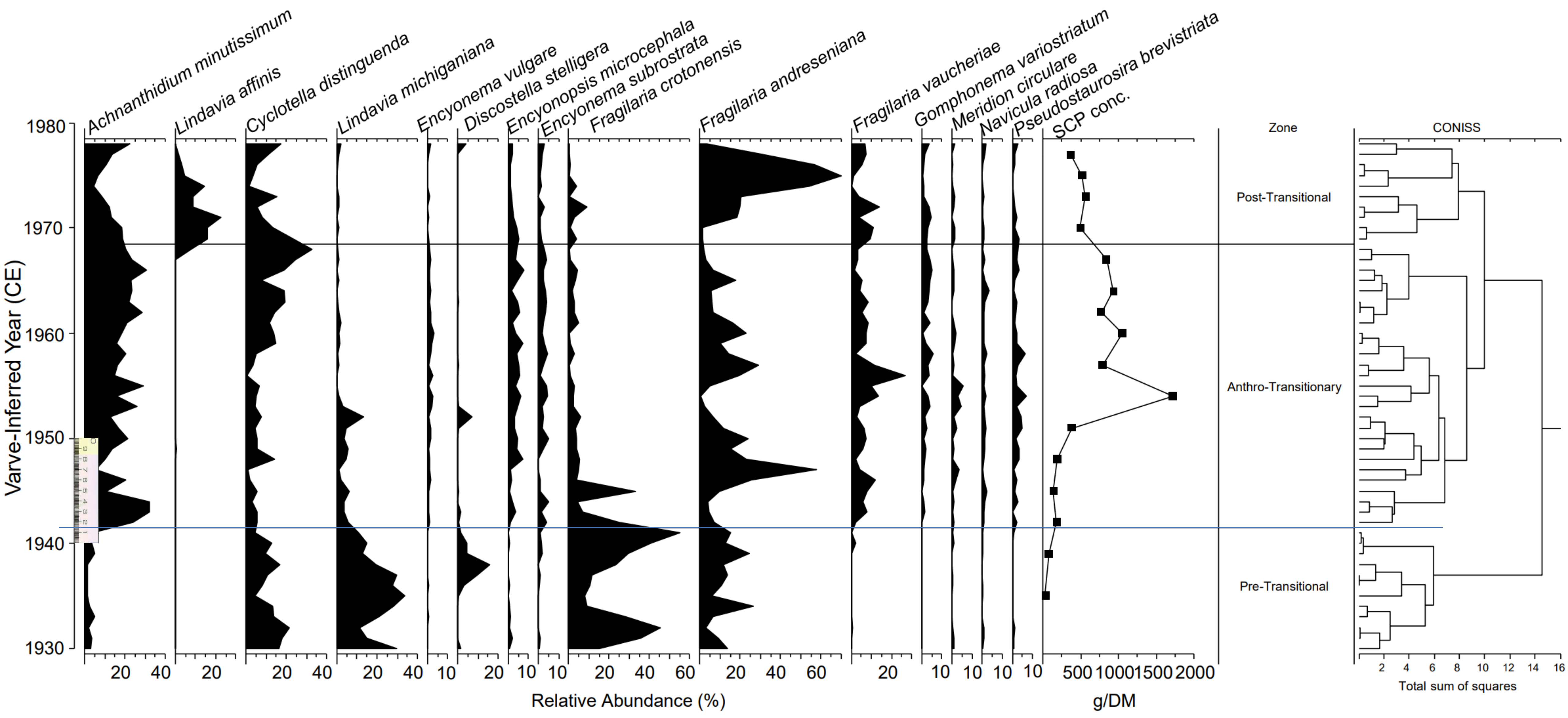

Marshall et al. (2023)

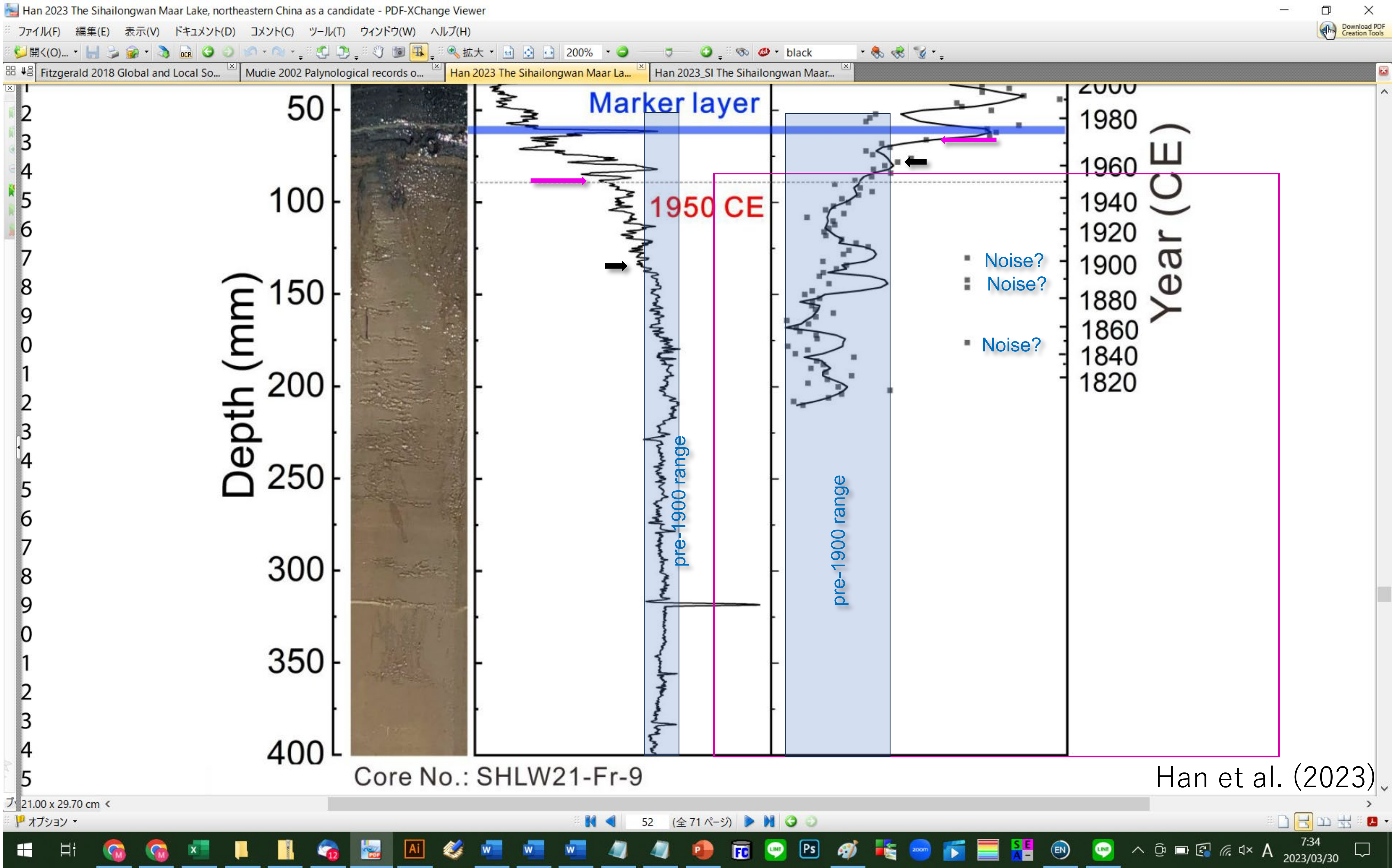

Han et al. (2023)

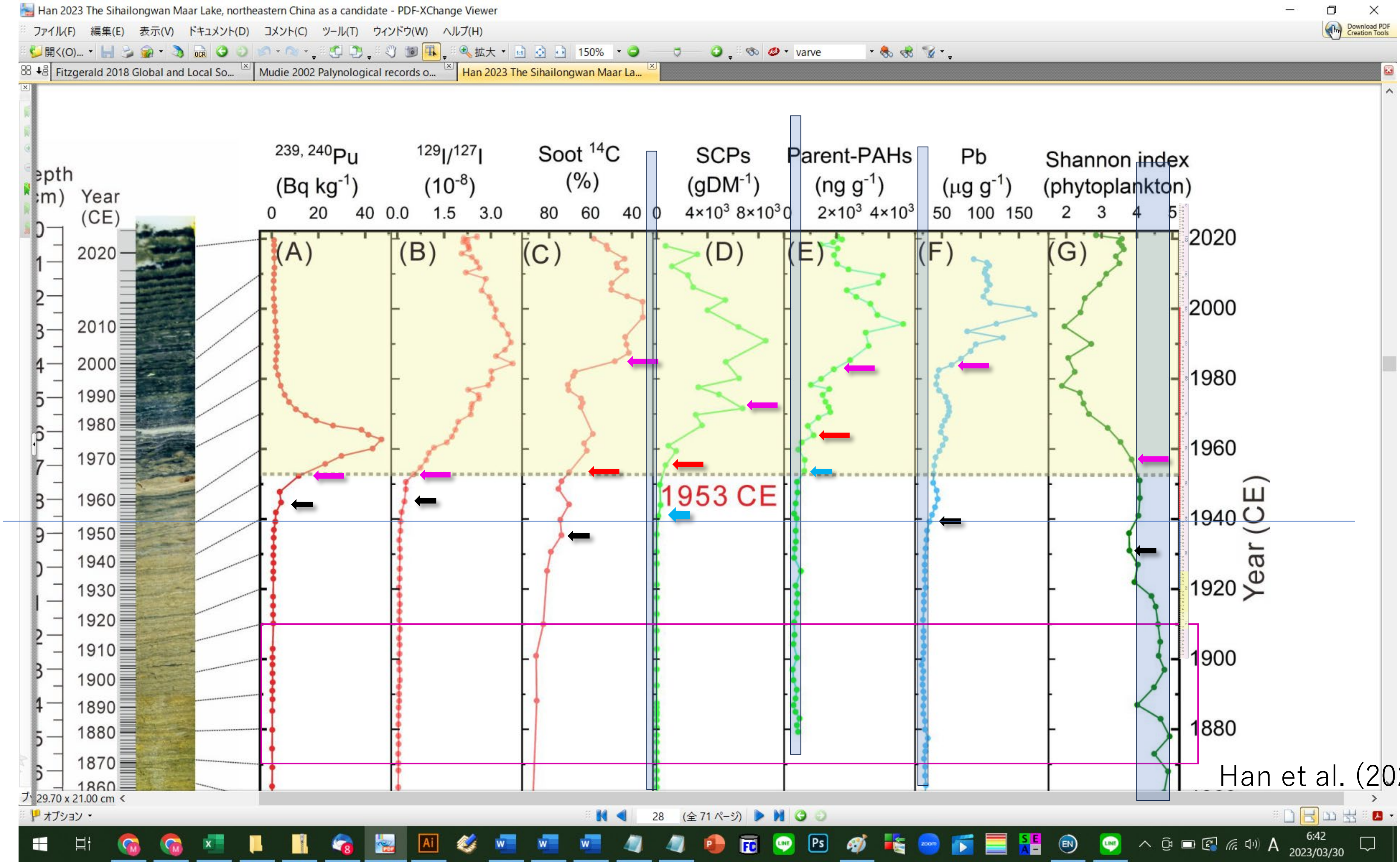

Han et al. (2023)

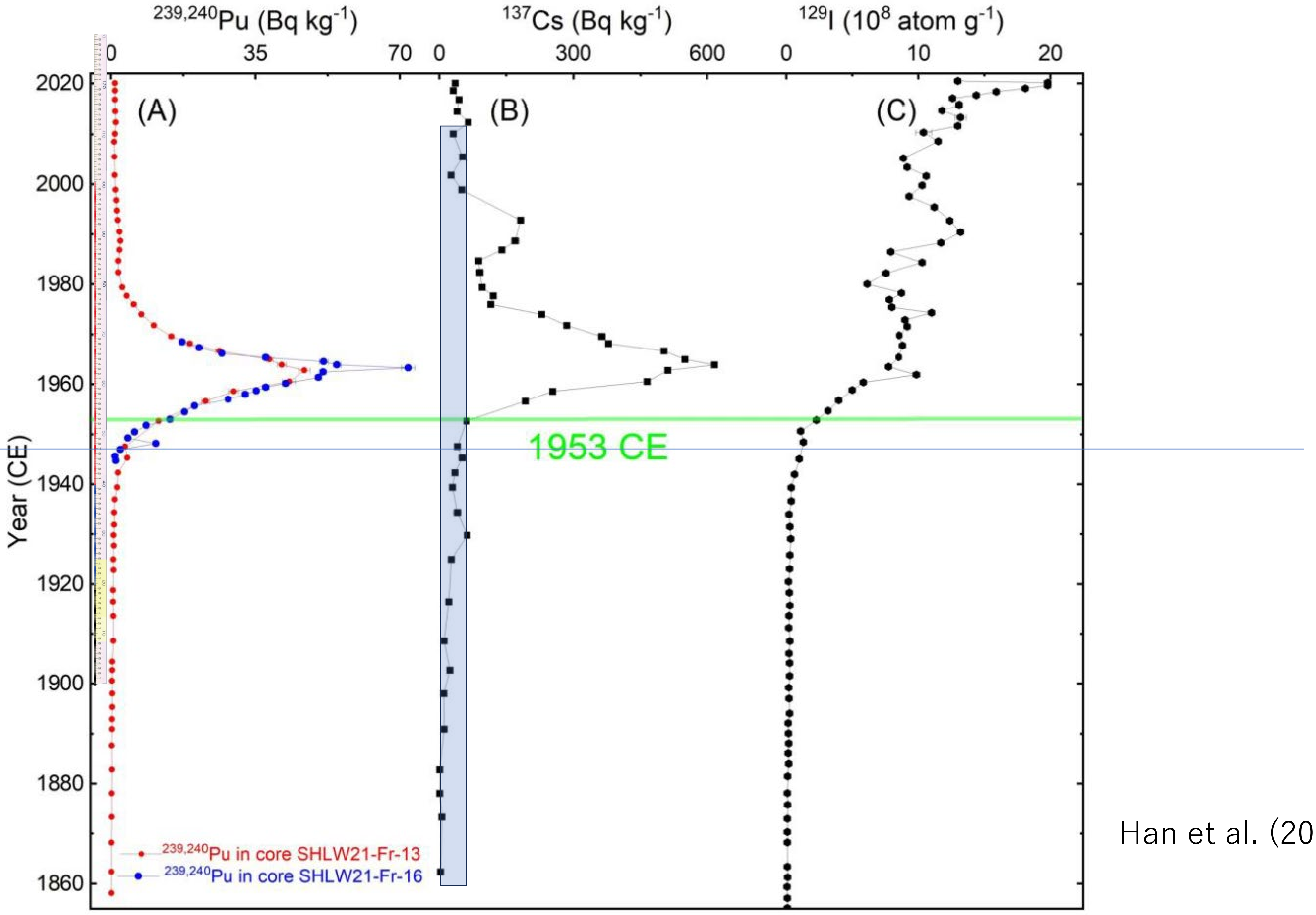

Han et al. (2023)

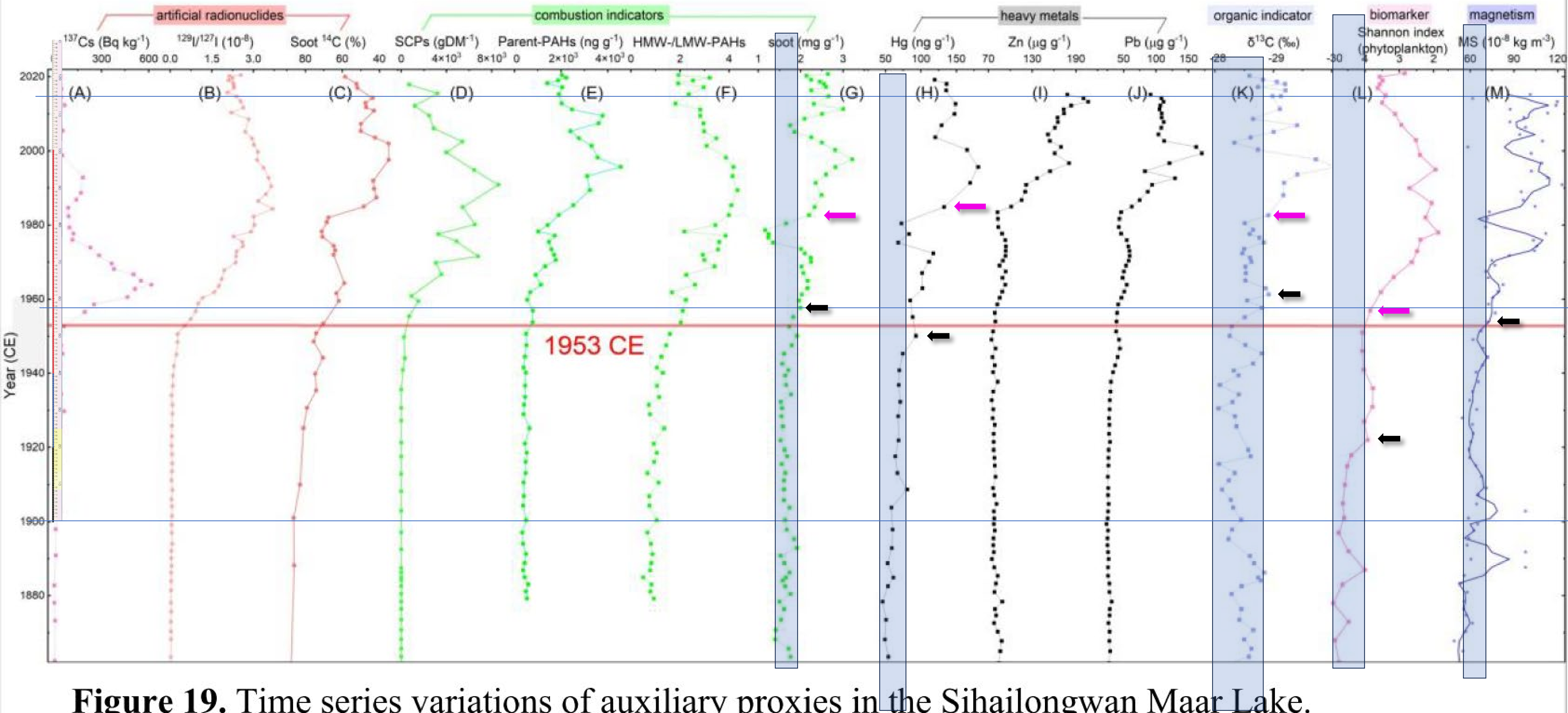

Figure 19. Time series variations of auxiliary proxies in the Sihailongwan Maar Lake.

Han et al. (2023)

Han 2023 The Sihailongwan Maar Lake, northeastern China as a candidate - PDF-XChange Viewer

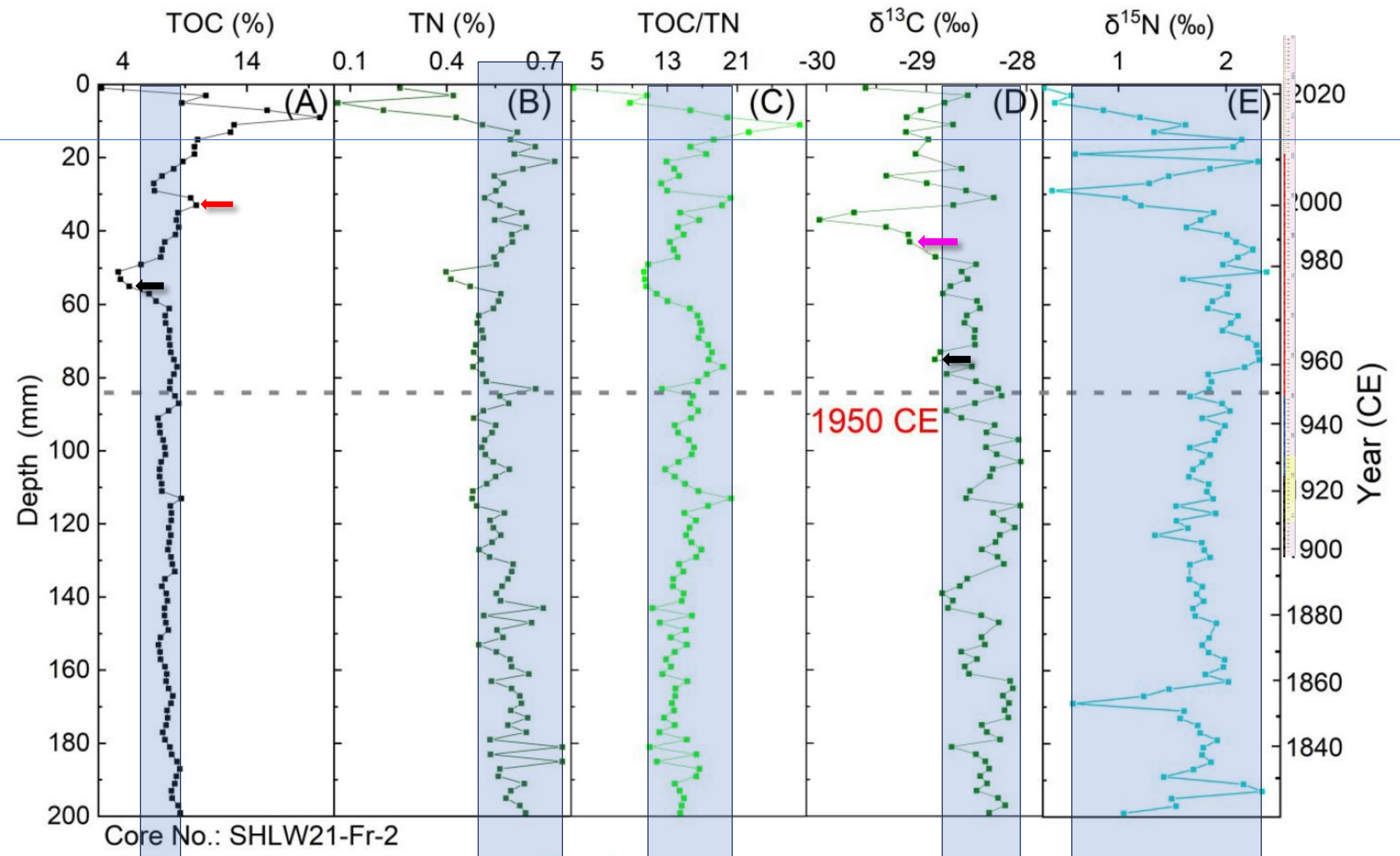

Figure 6. Depth distributions of total organic carbon (TOC), total nitrogen (TN) and their

Han et al. (2023)

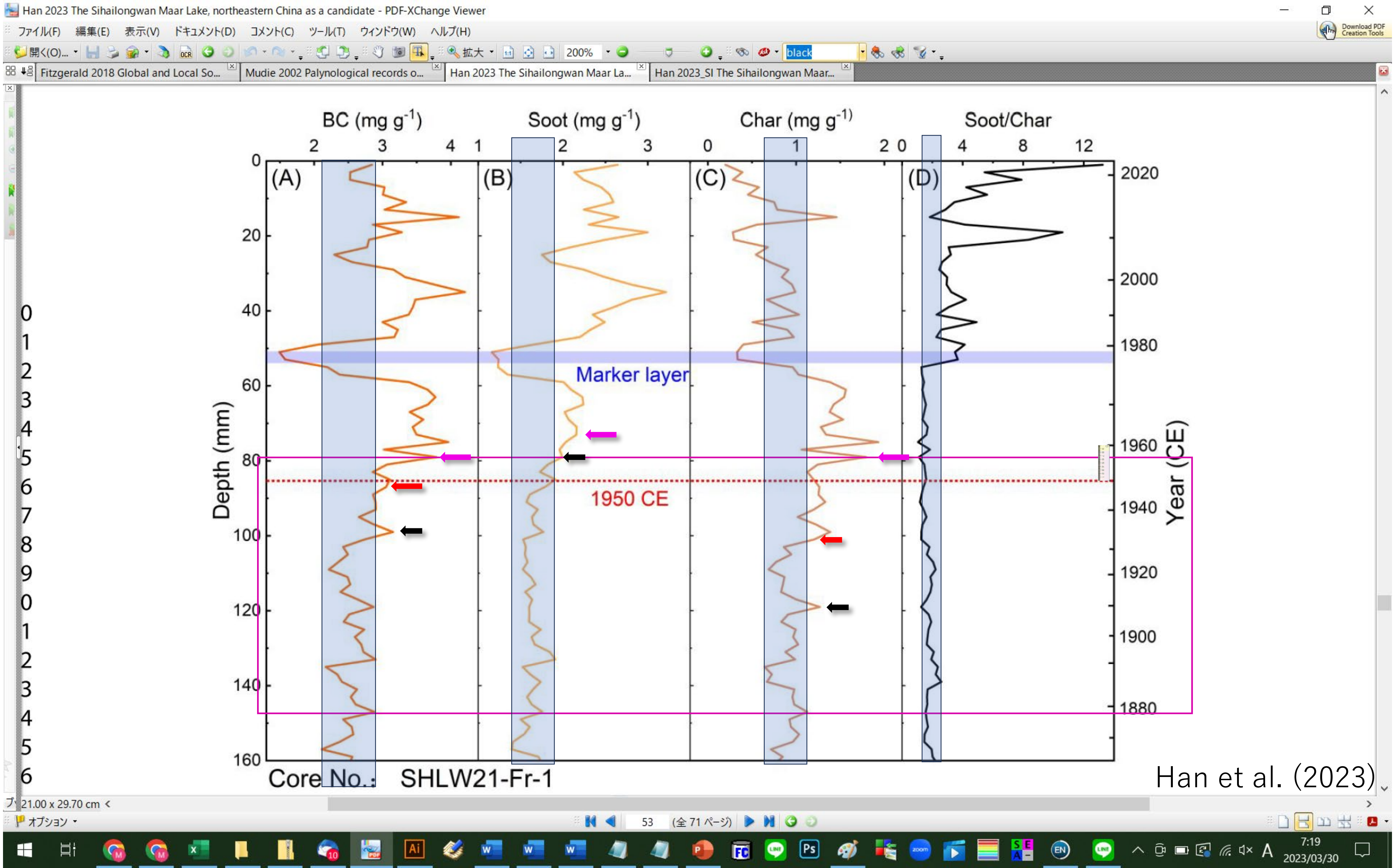

Han et al. (2023)

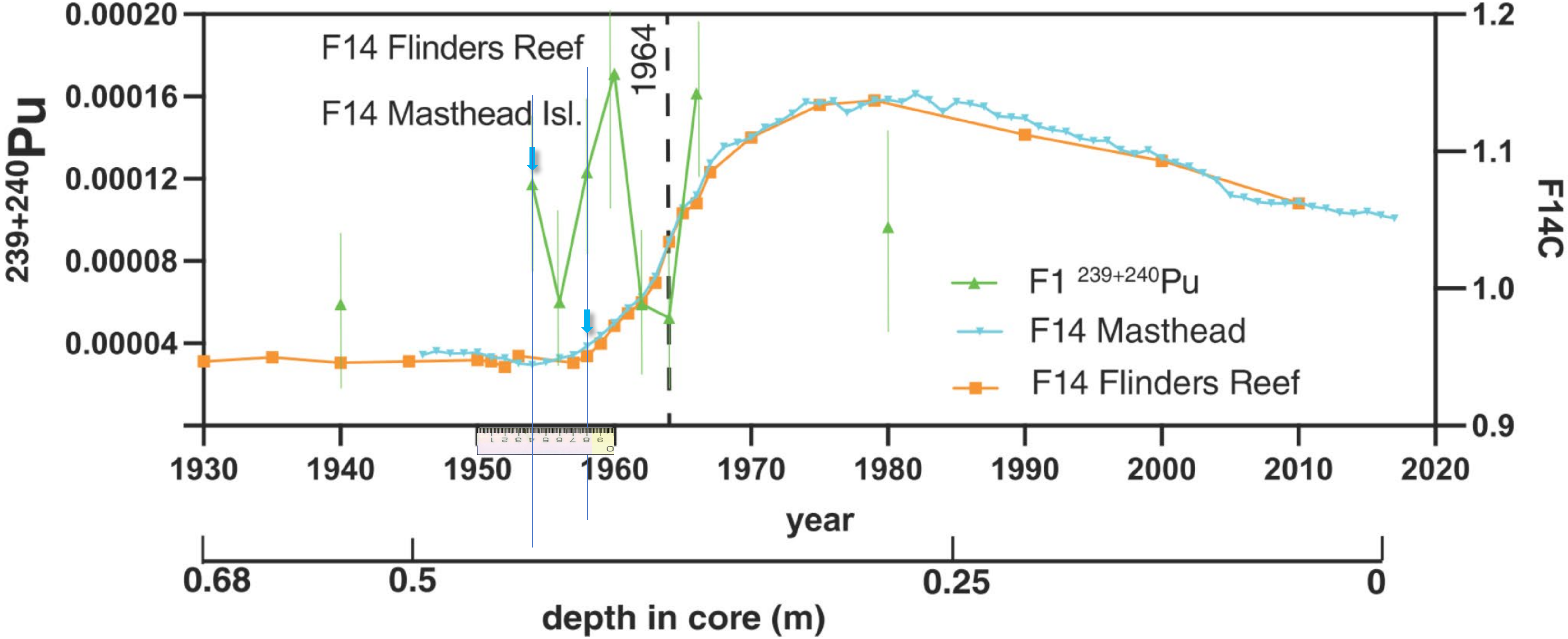

Zinke et al. (2023)

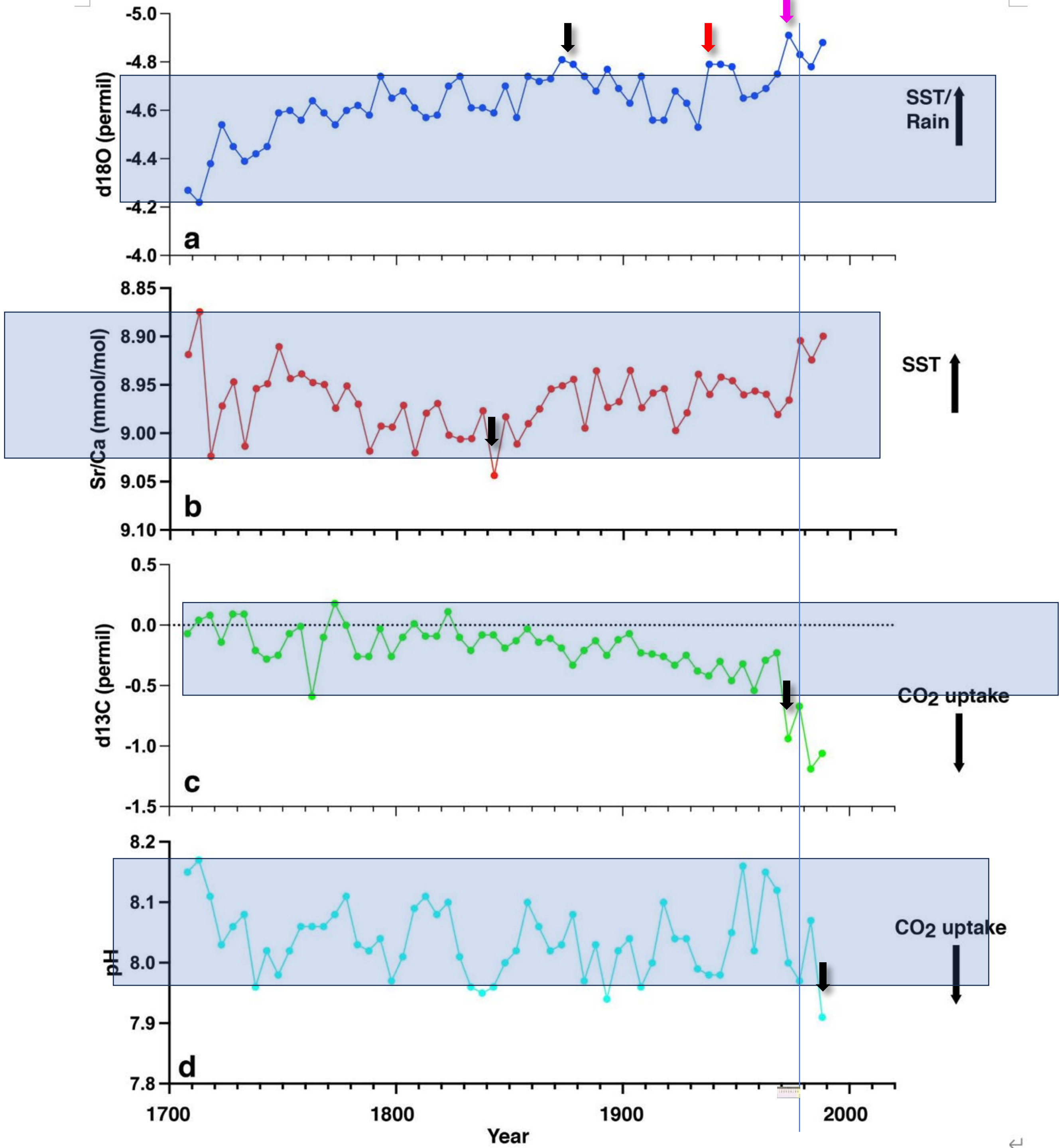

Zinke et al. (2023)

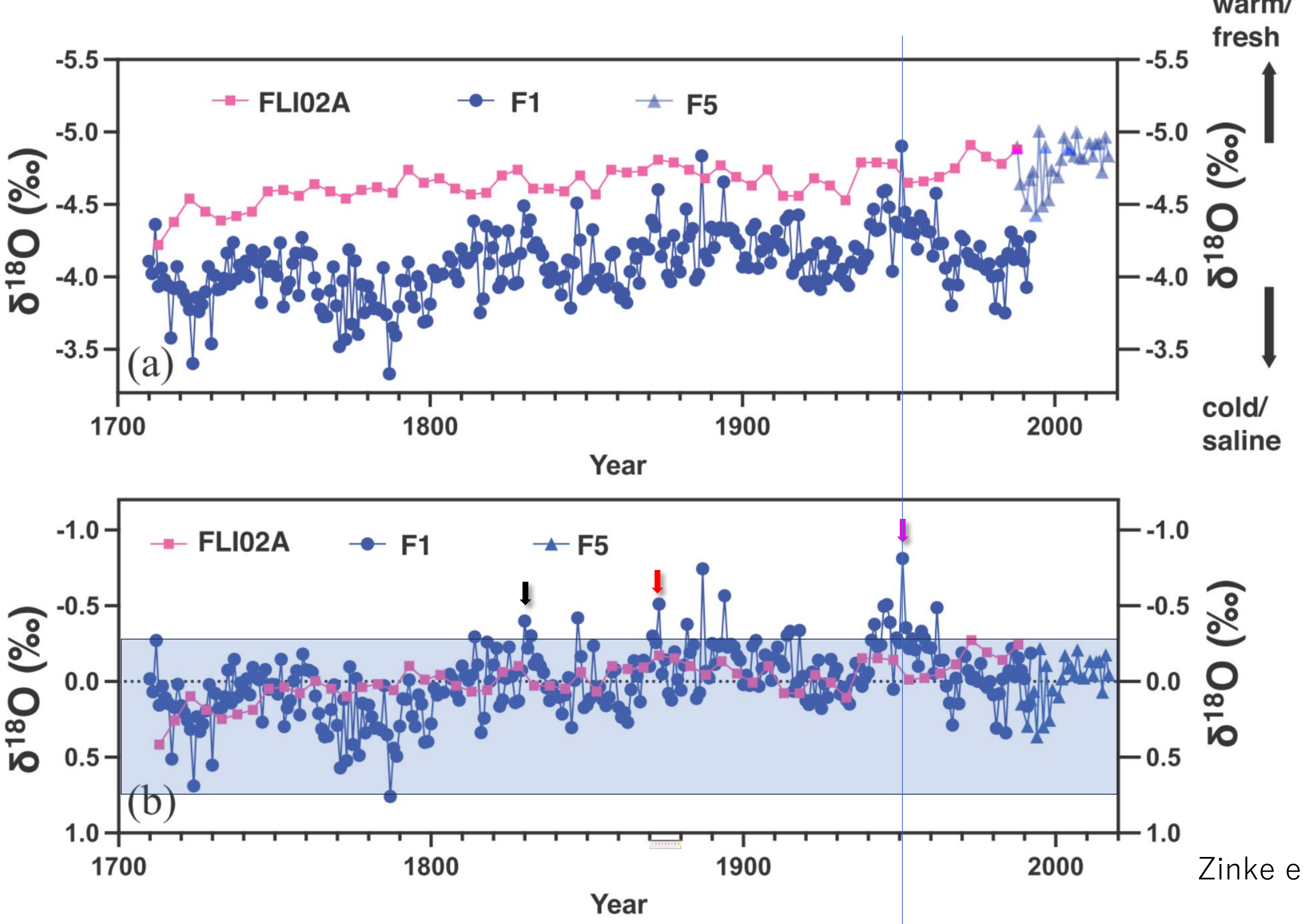

Zinke et al. (2023)

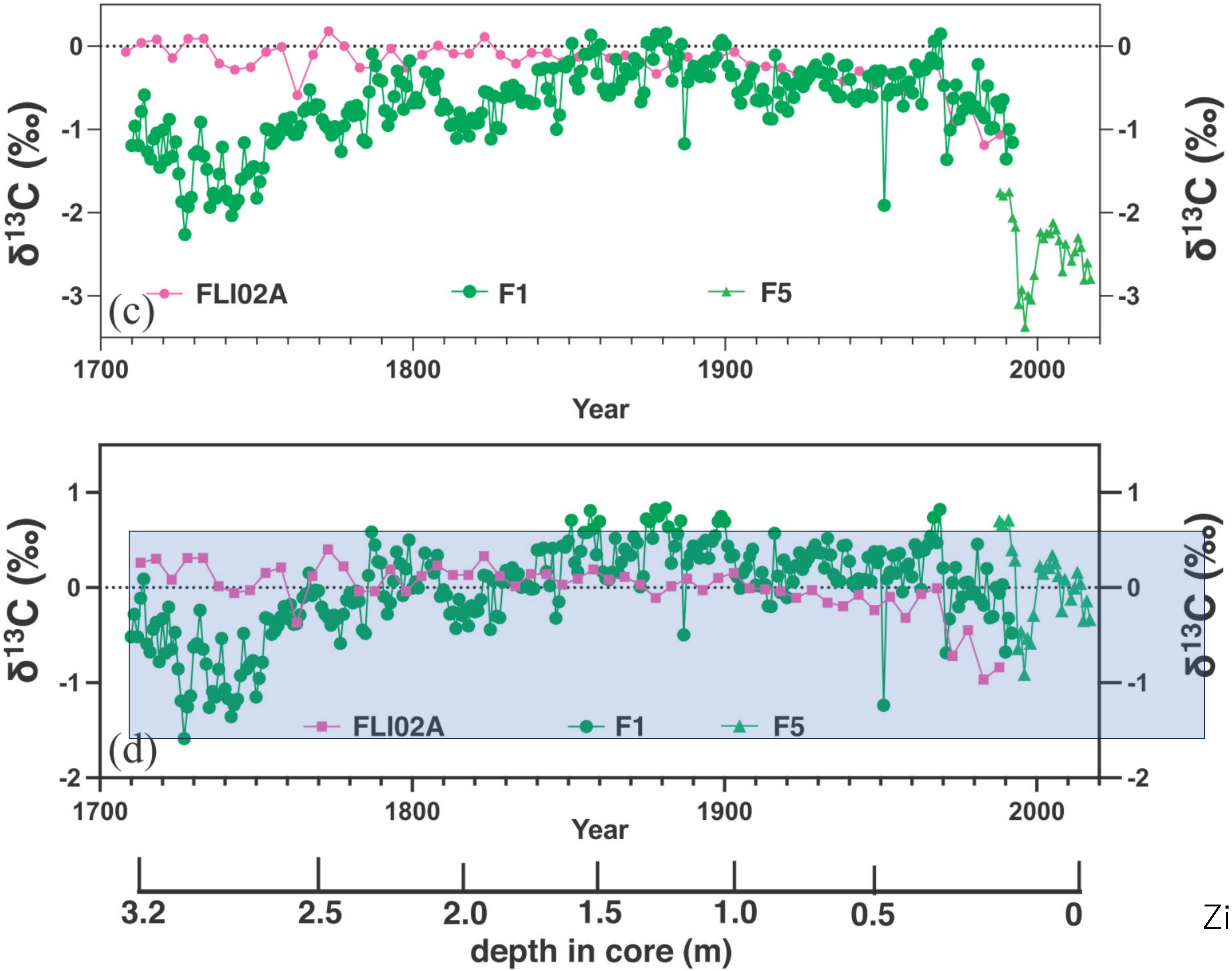

Zinke et al. (2023)

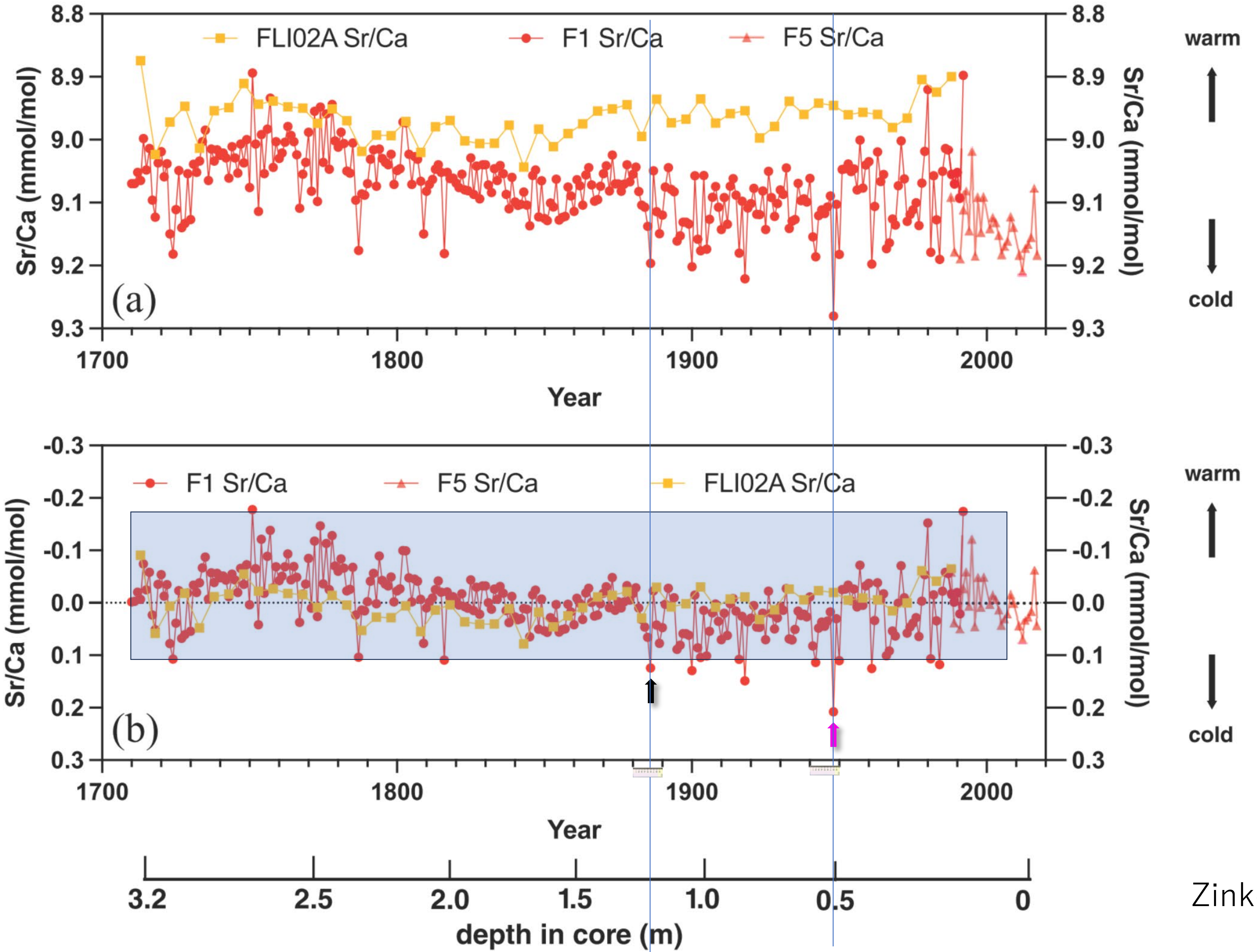

Zinke et al. (2023)

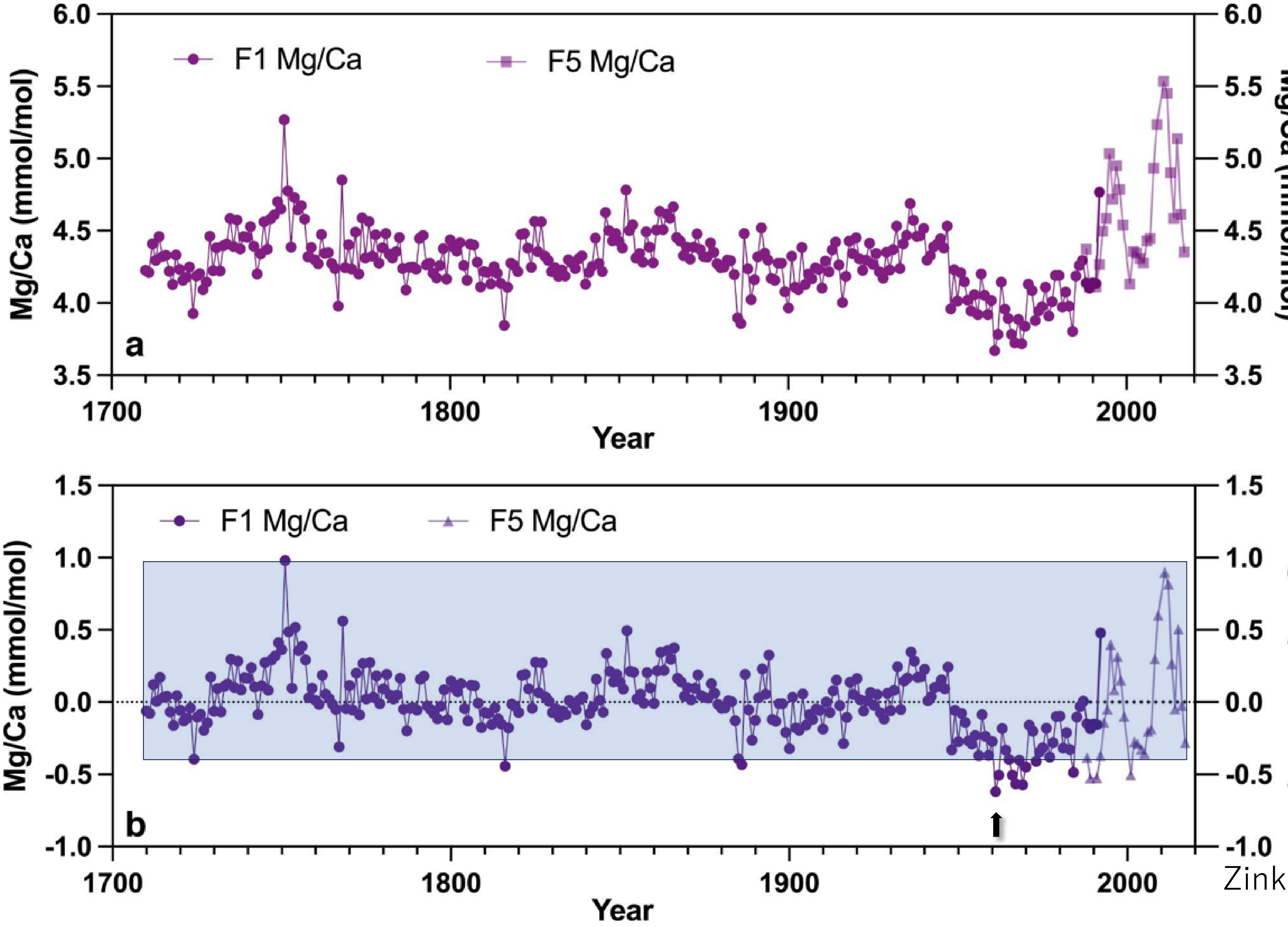

Zinke et al. (2023)

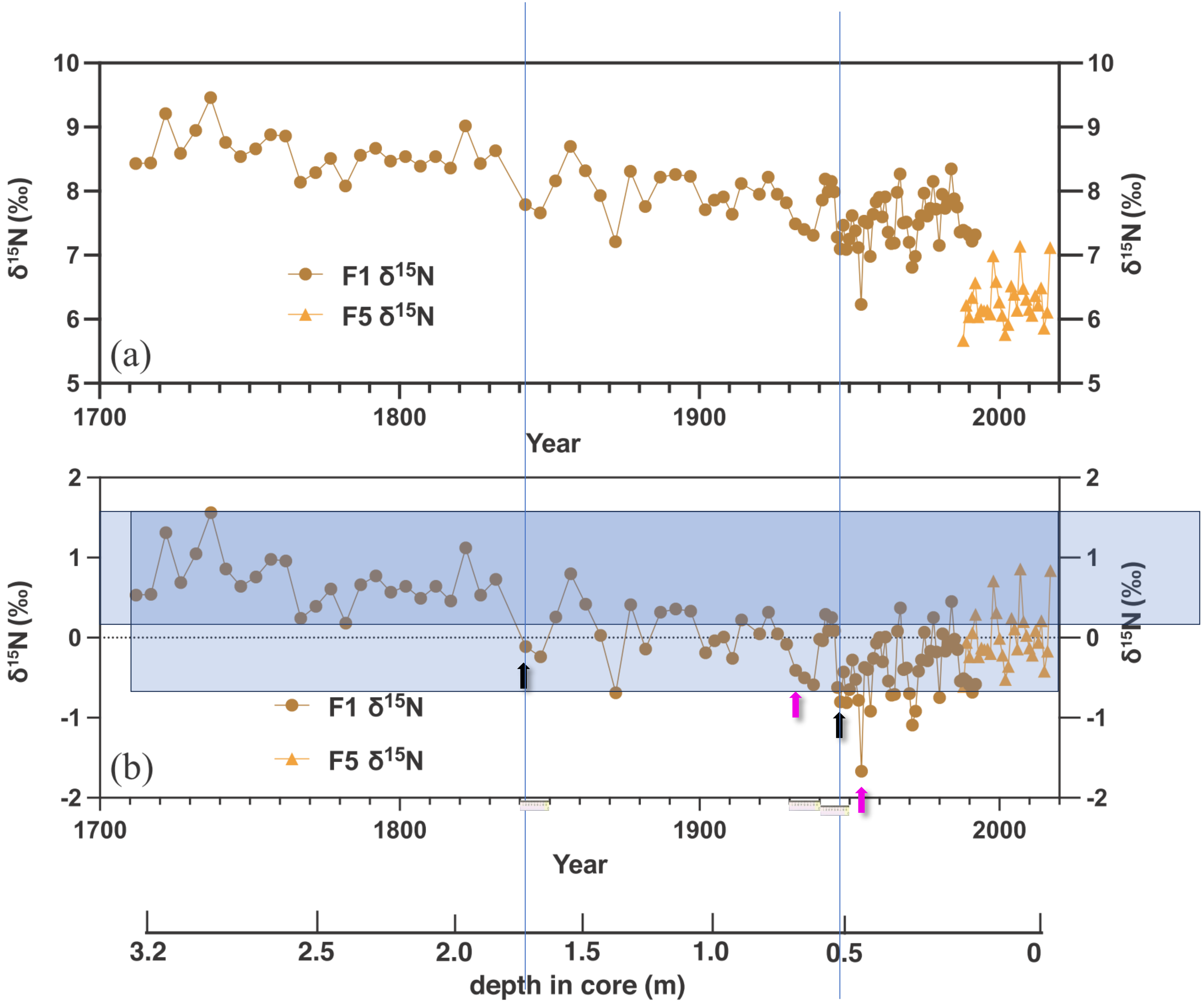

Zinke et al. (2023)

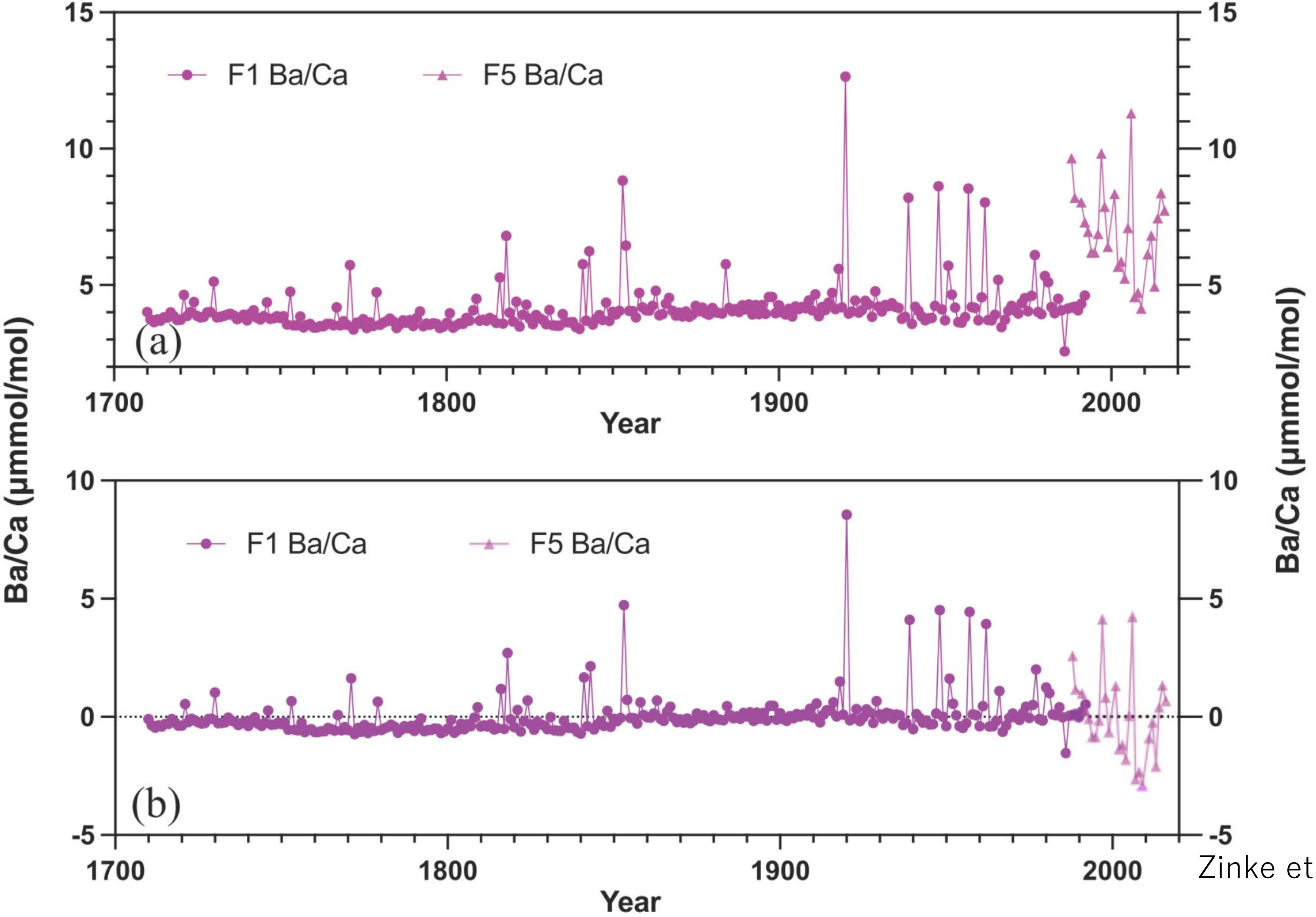

Zinke et al. (2023)

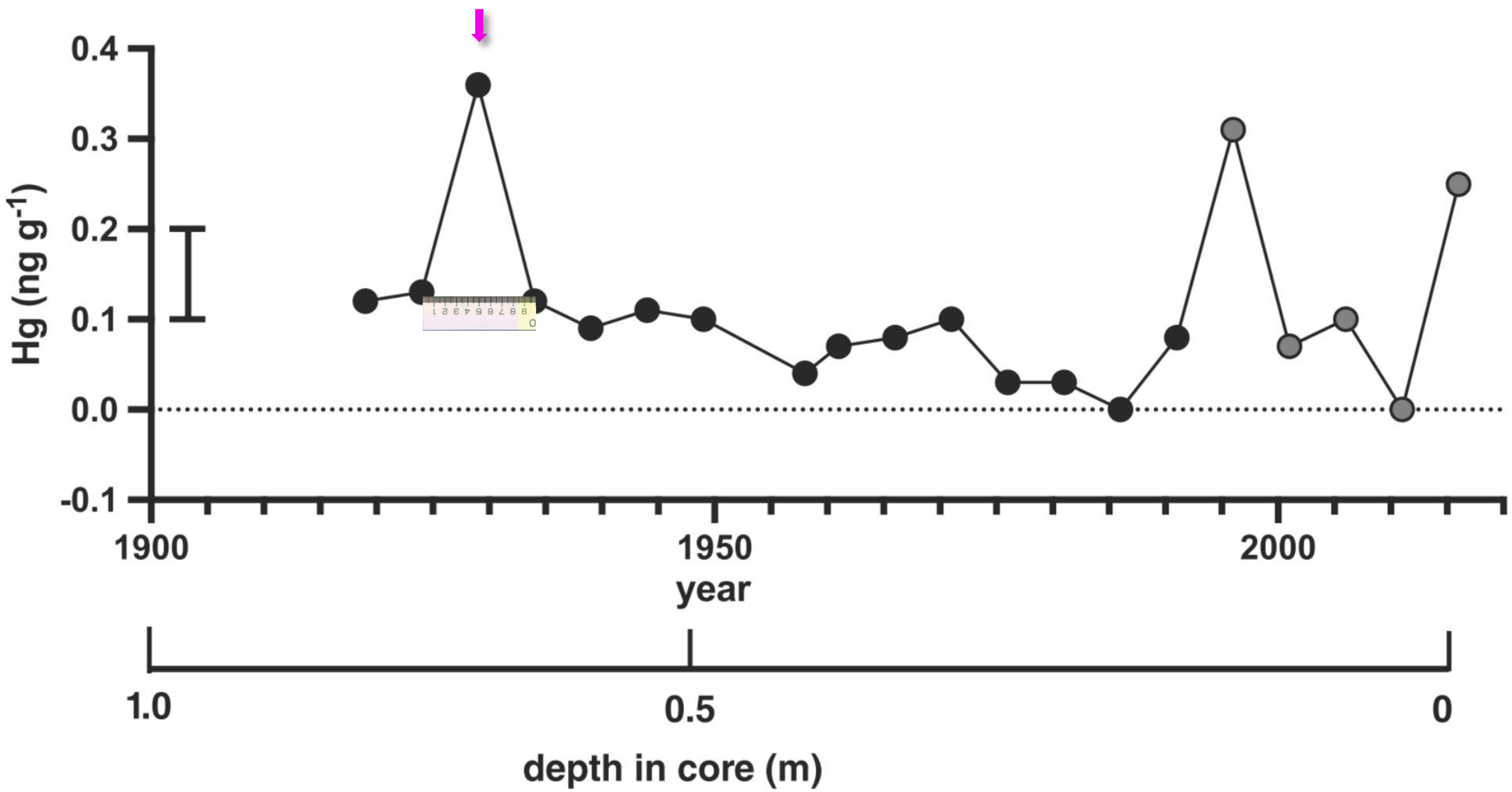

Zinke et al. (2023)

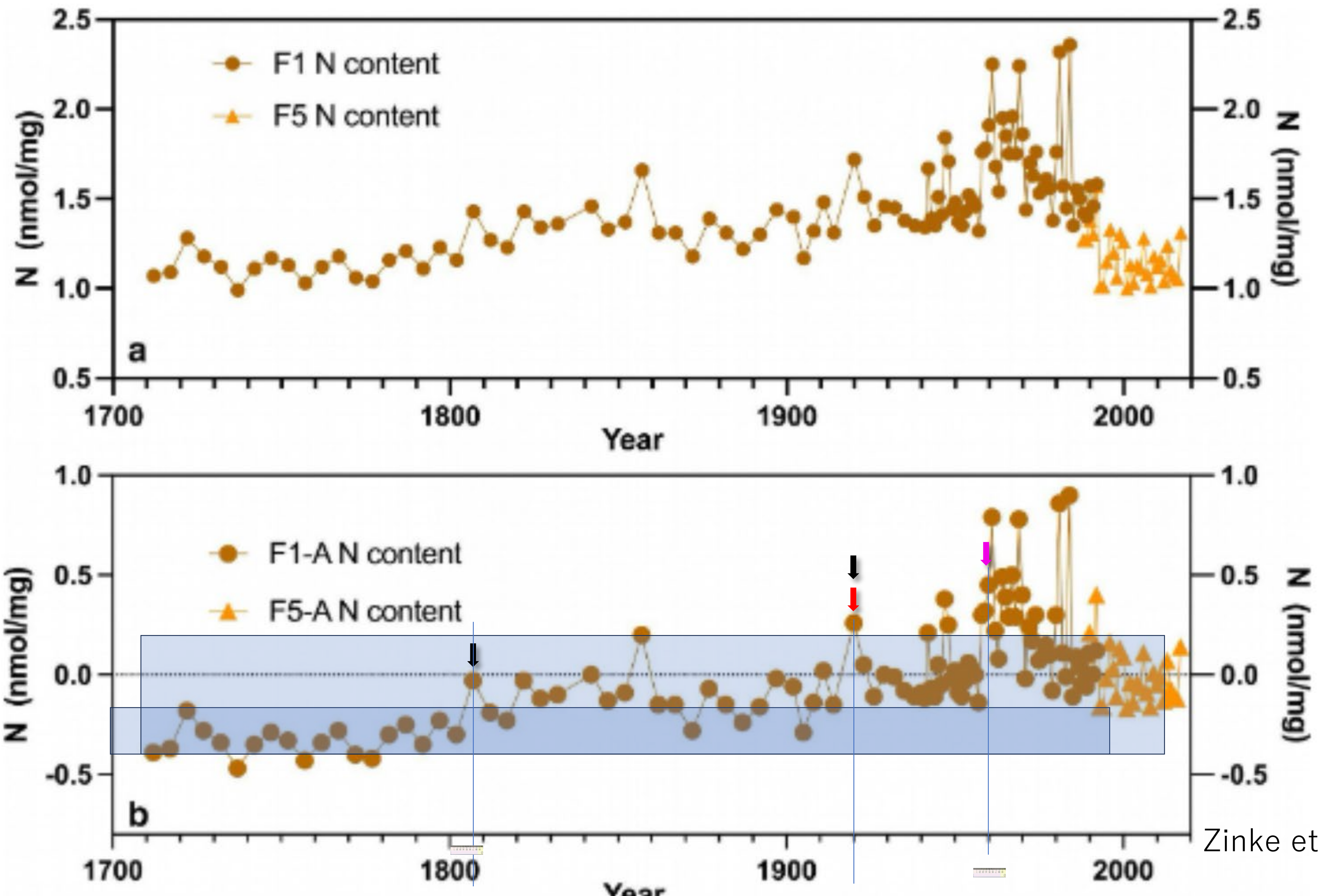

Zinke et al. (2023)

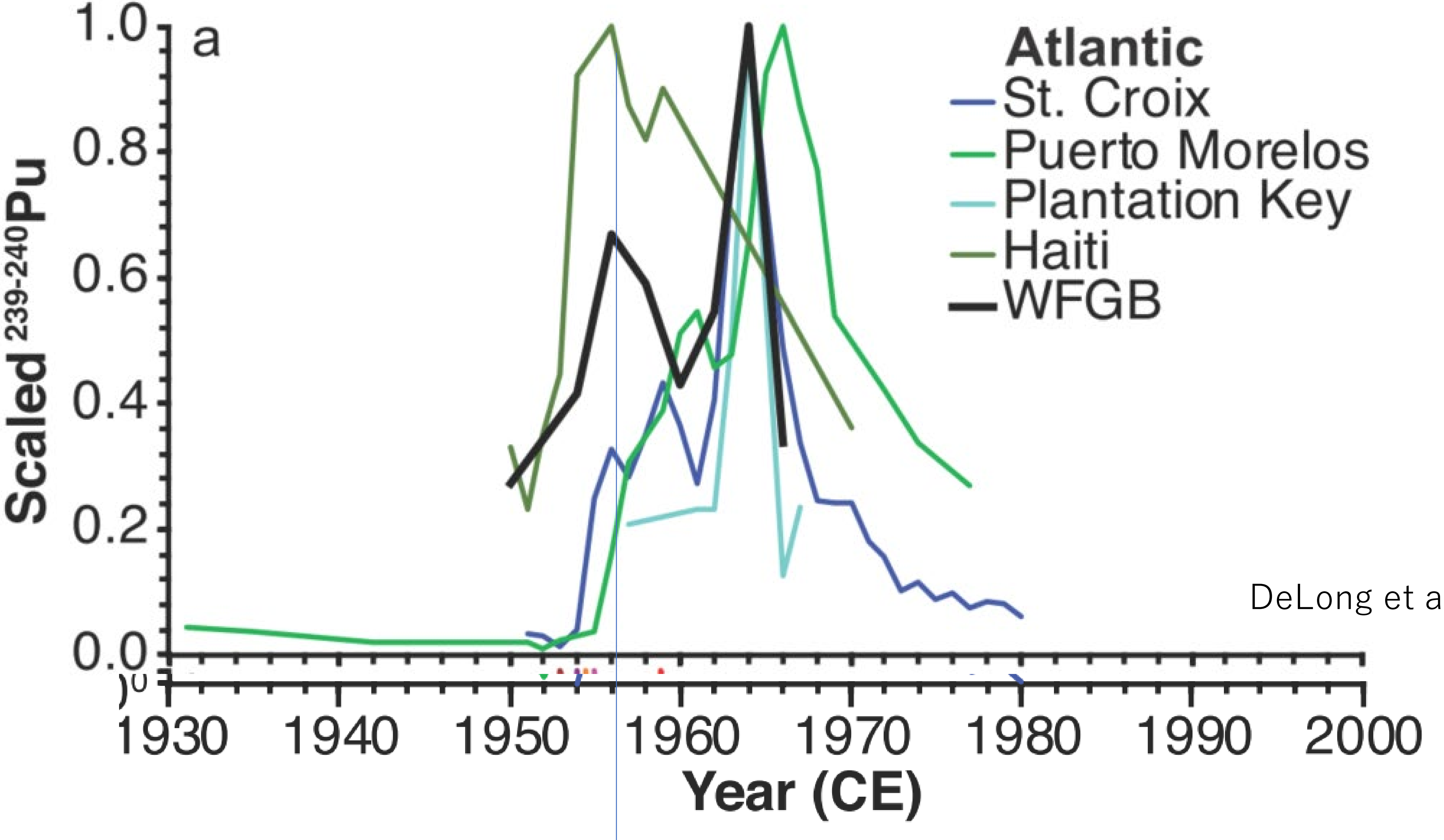

DeLong et al. (2023)

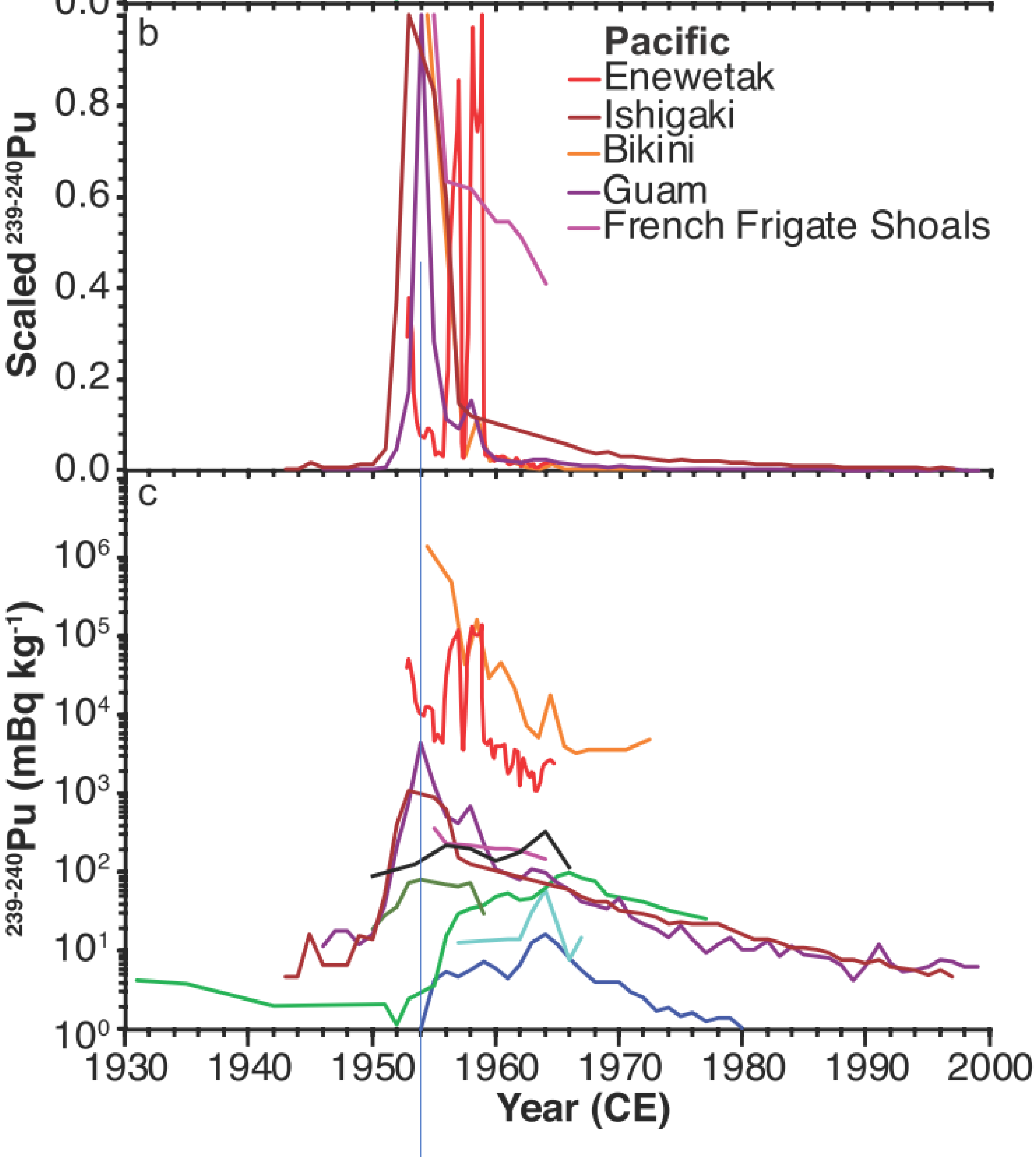

DeLong et al. (2023)

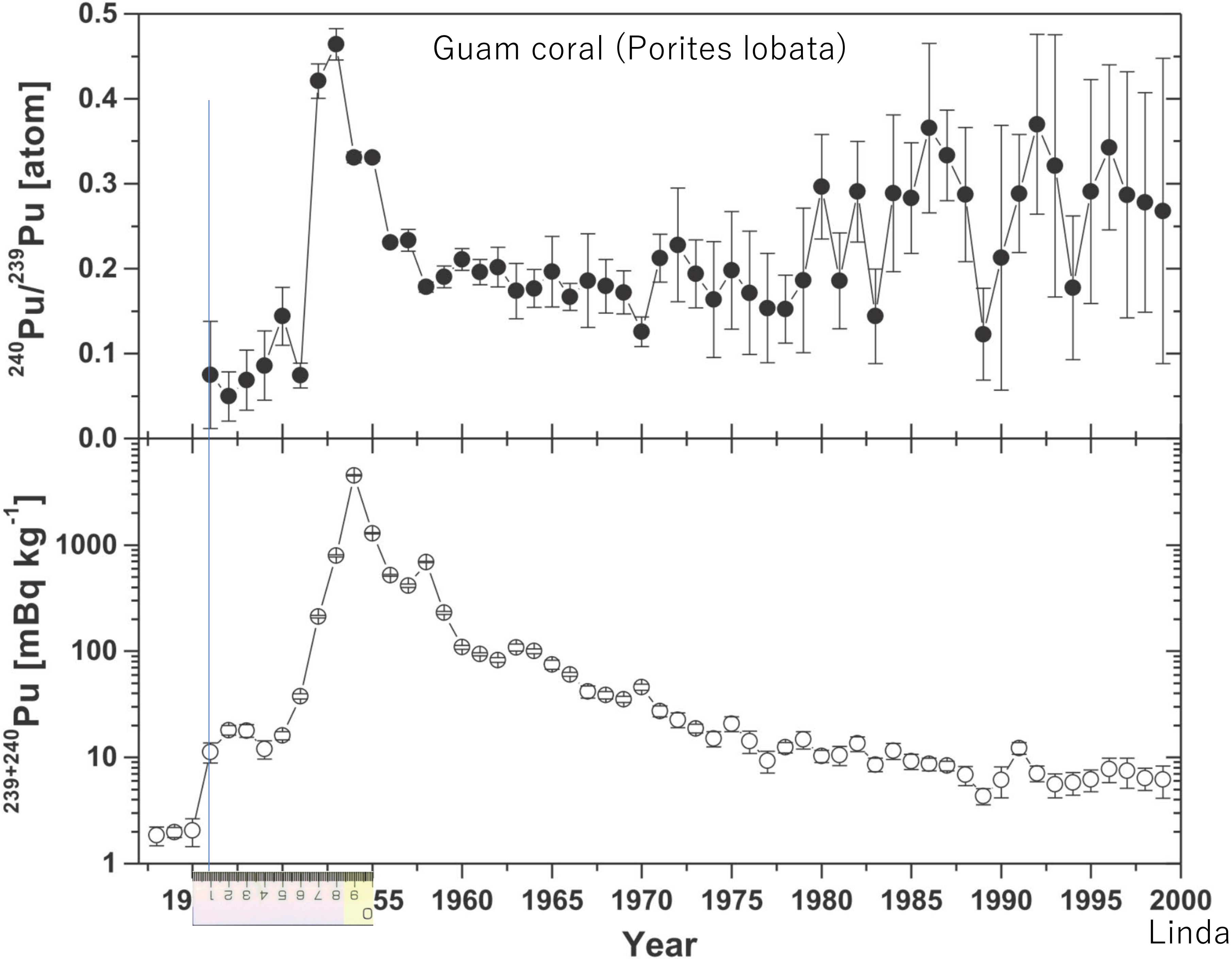

Lindahl et al. (2011)

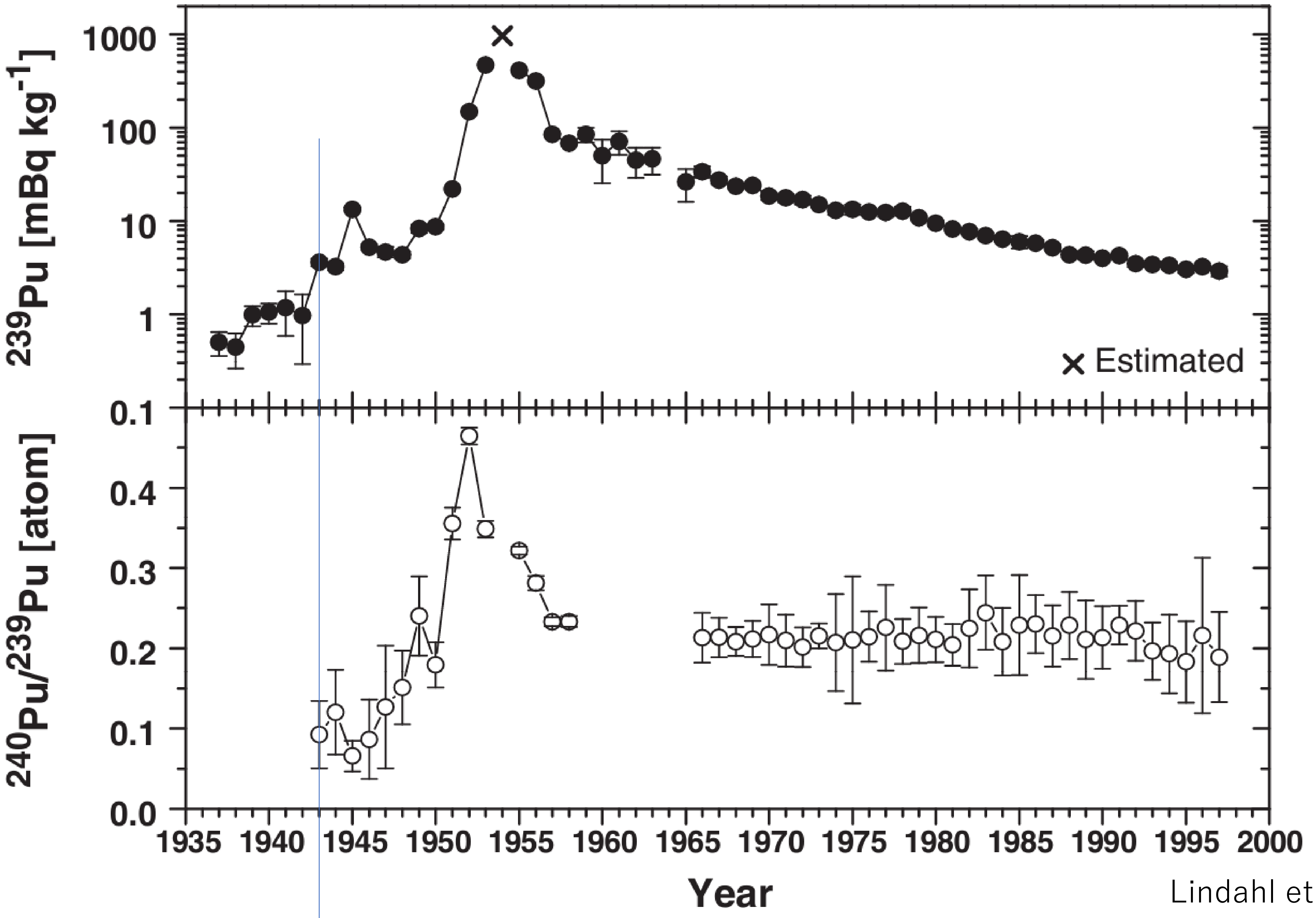

Lindahl et al. (2012)

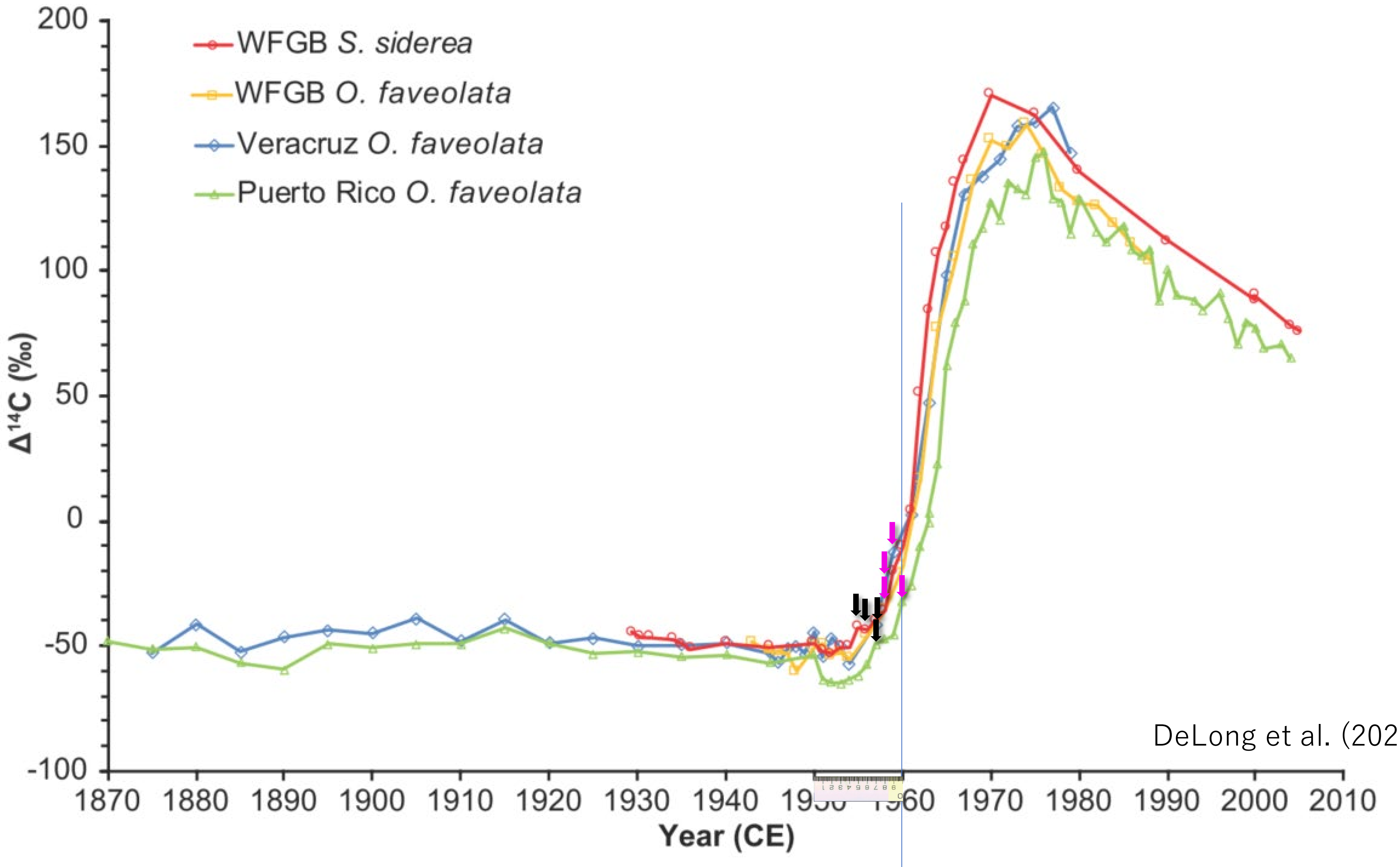

DeLong et al. (2023)

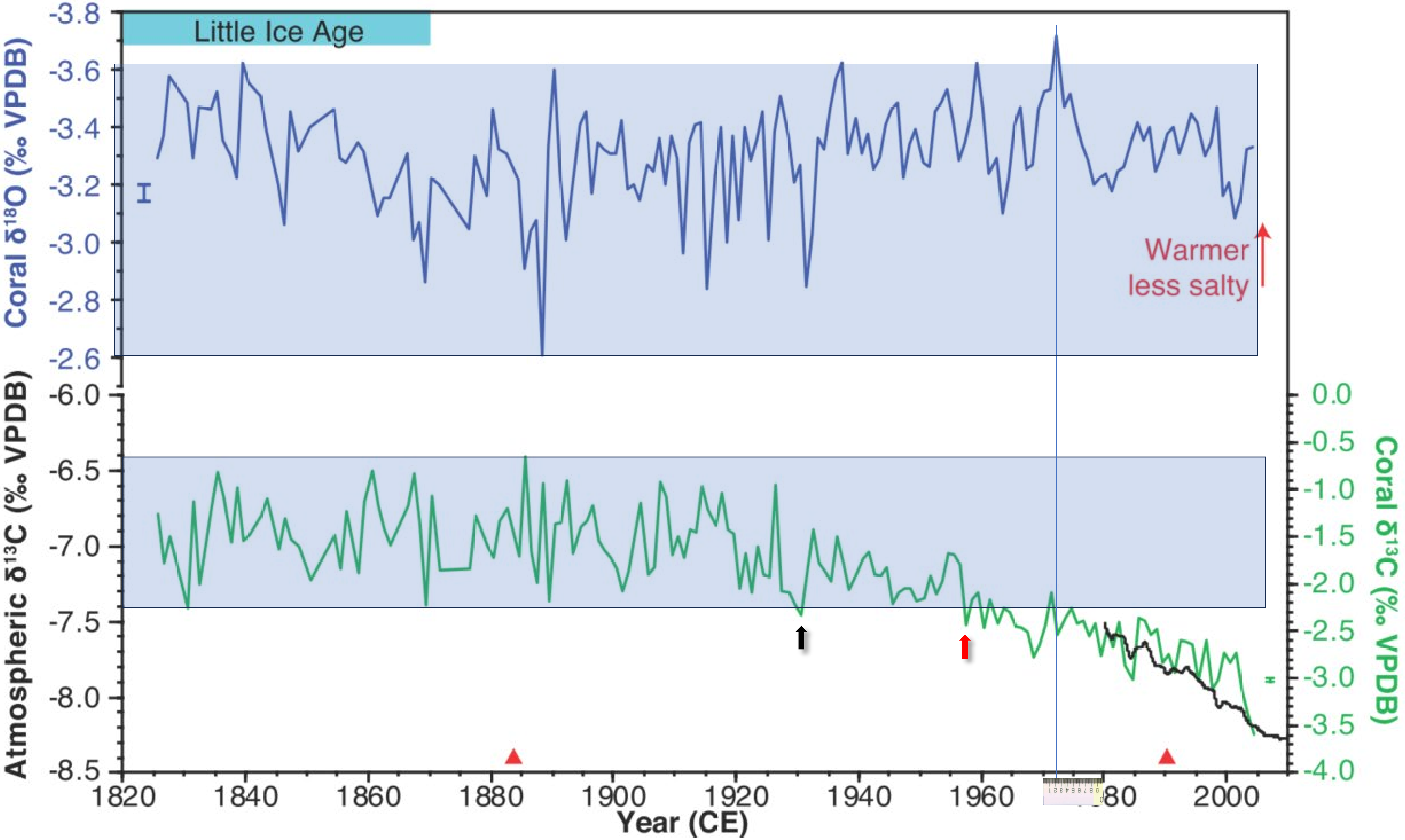

DeLong et al. (2023)

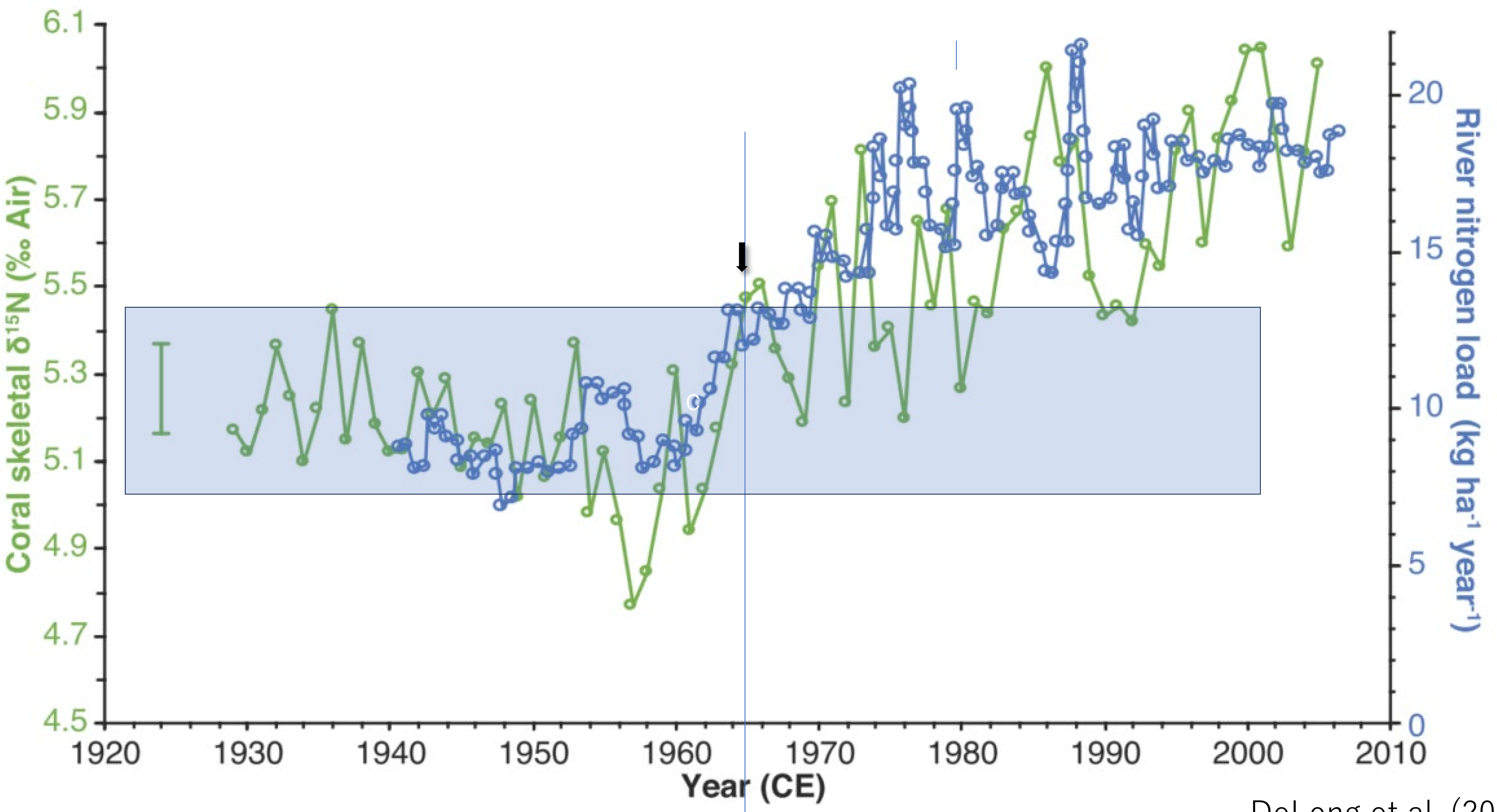

DeLong et al. (2023)

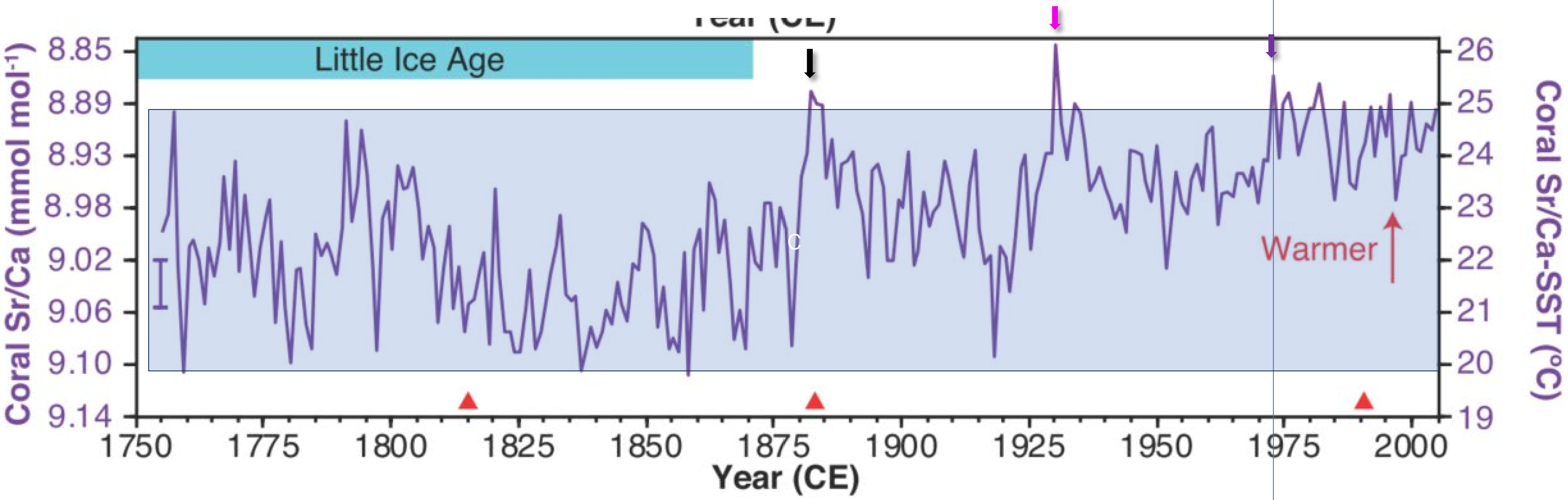

DeLong et al. (2023)

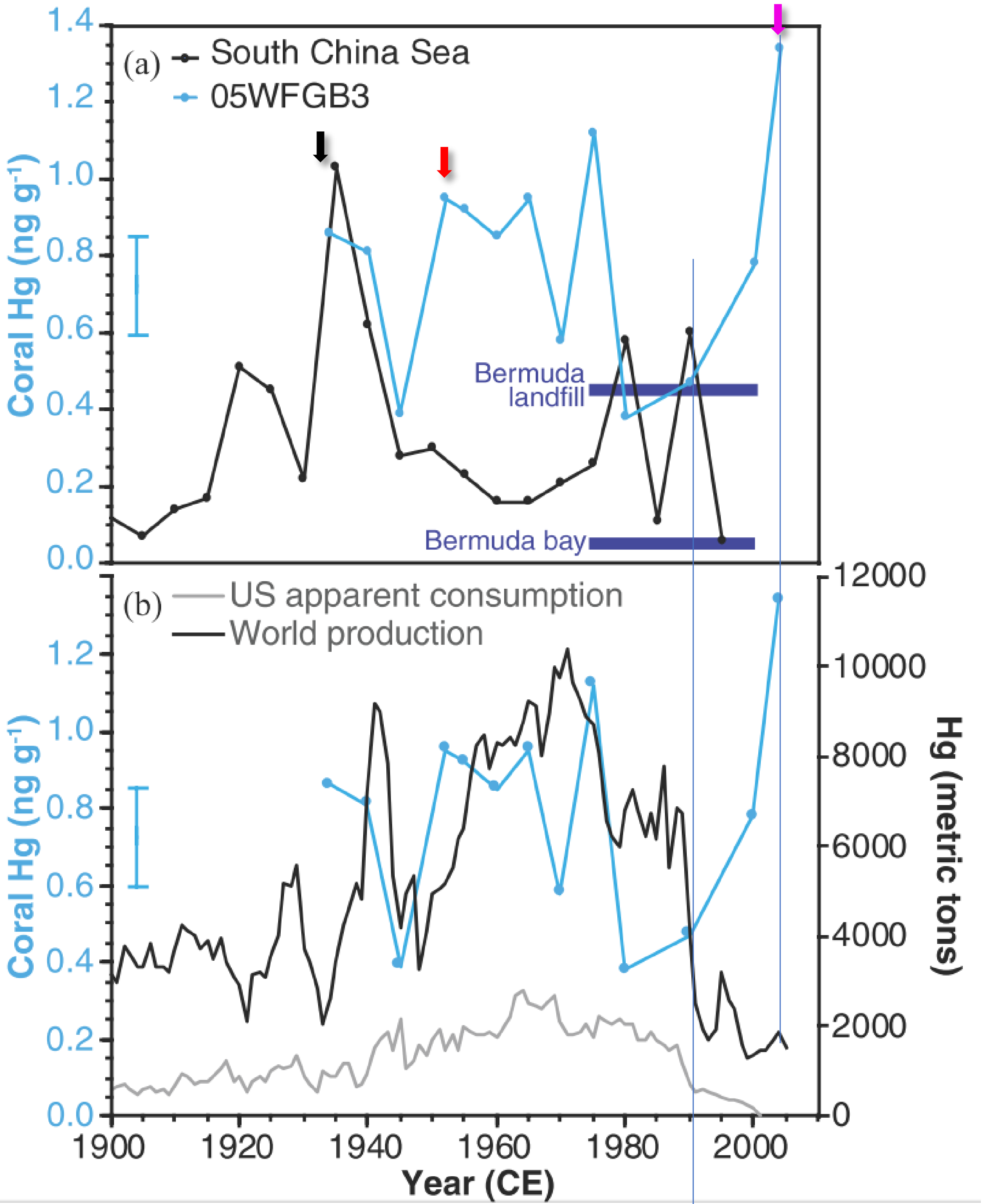

DeLong et al. (2023)

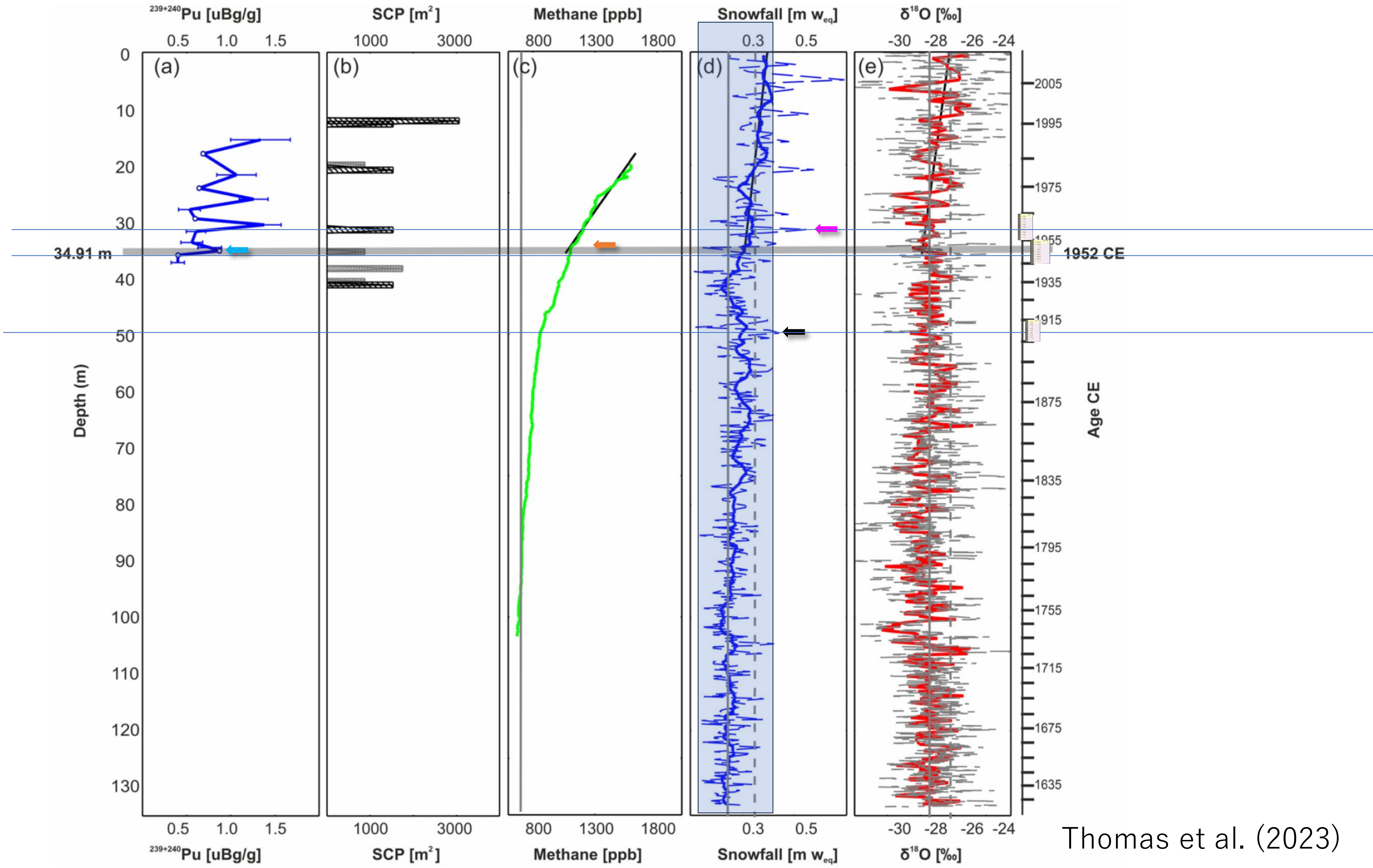

Thomas et al. (2023)

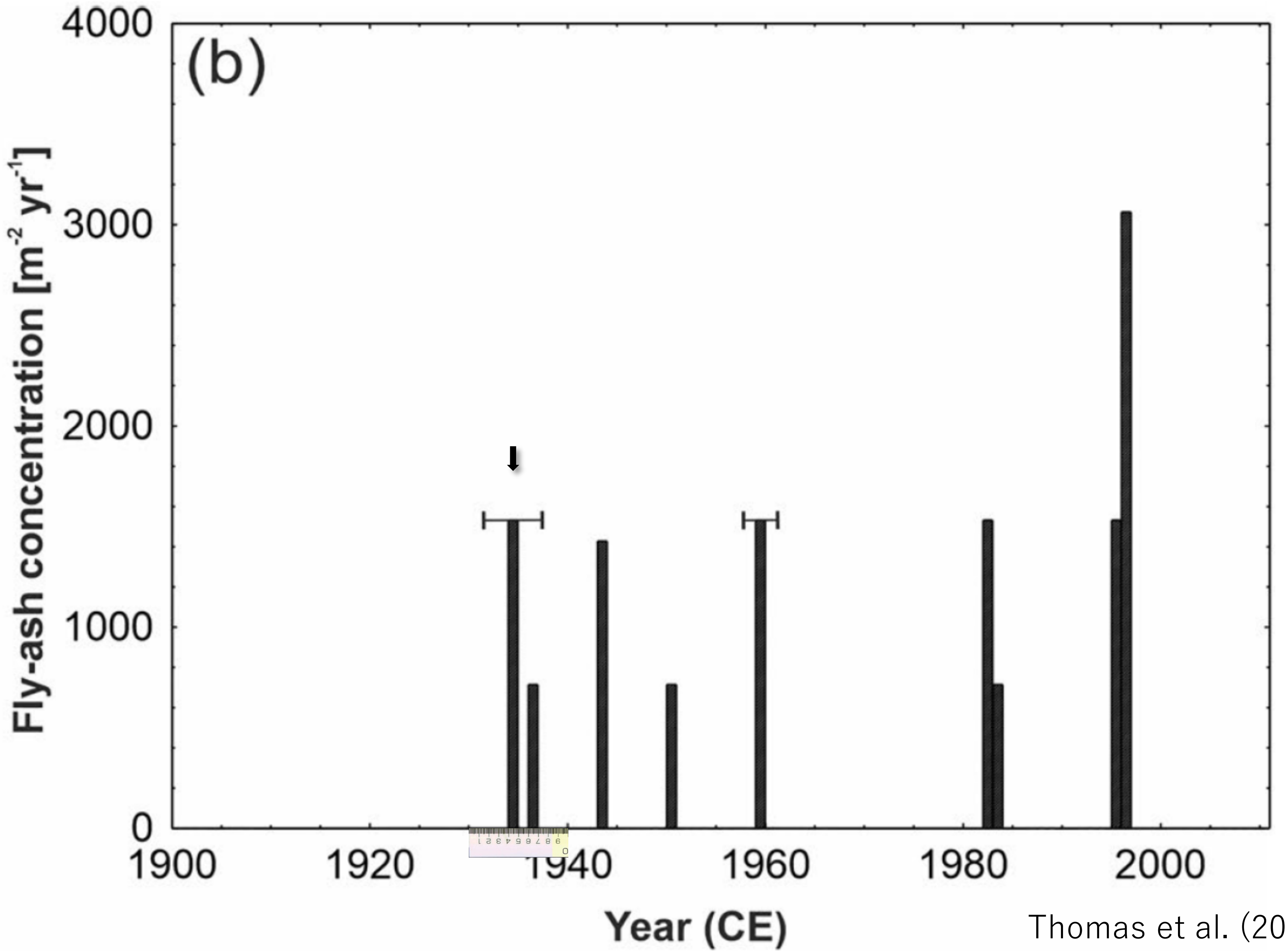

Thomas et al. (2023)

aslopubs.onlinelibrary.wiley.com/doi/pdfdirect/10.4319/lo.2000.45.4.0801

The record of nitrate utilization and productivity limitation provided by δ15N values i... 6 / 13 150%

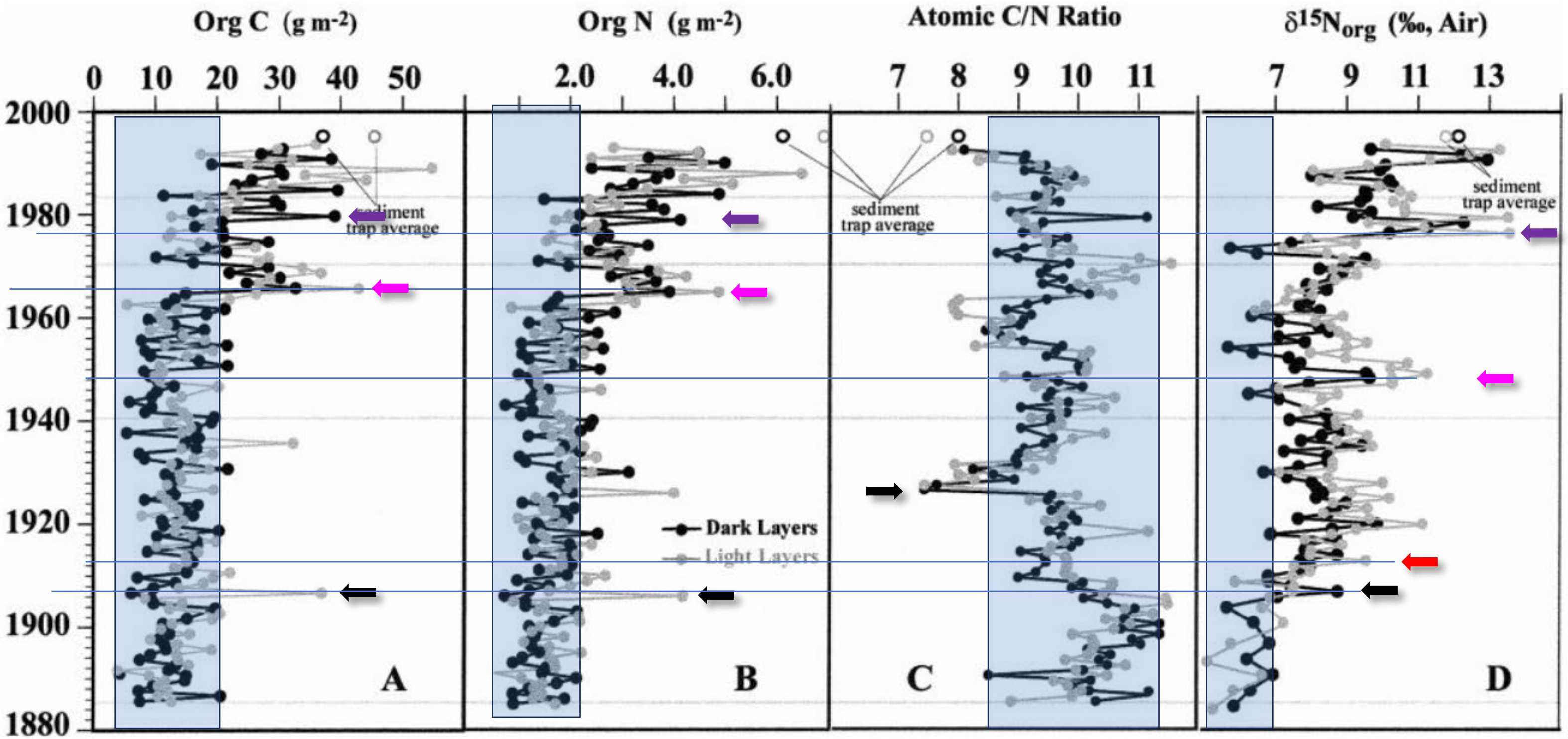

Fig. 5. (A) Stratigraphic plots of Org C accumulation, (B) Total N accumulation, (C) C/N atomic ratios and, (D) $\delta^{15}N$ from the Baldeggersee laminated sequence. Results from the light and dark laminae are plotted separately. Open circles on the graph represent average sediment trap values for theoretical light and dark layers, weighted by mass.

Teranes and Bernasconi (2000)

concentrations in Fig. 4C documents the relationship between nitrate utilization and sediment $\delta^{15}N$ values taken at similar temporal resolution. Nitrate concentrations decreased

72.6 g C m^{-2} and 11.2 g N m^{-2} for May–July 1996 (Fig. 4A). Average atomic C/N ratios, weighted by amount, are 7.2 for May–July 1995; 8.0 for August 1995–March 1996;

2000, 4, Downloaded from https://aslopubs.onlinelibrary.wiley.com/doi/10.4319/lo.2000.45.4.0801 by Cochrane Japan, Wiley Online Library on [27/02/2023]. See the Terms and Co

2023/02/27 23:39

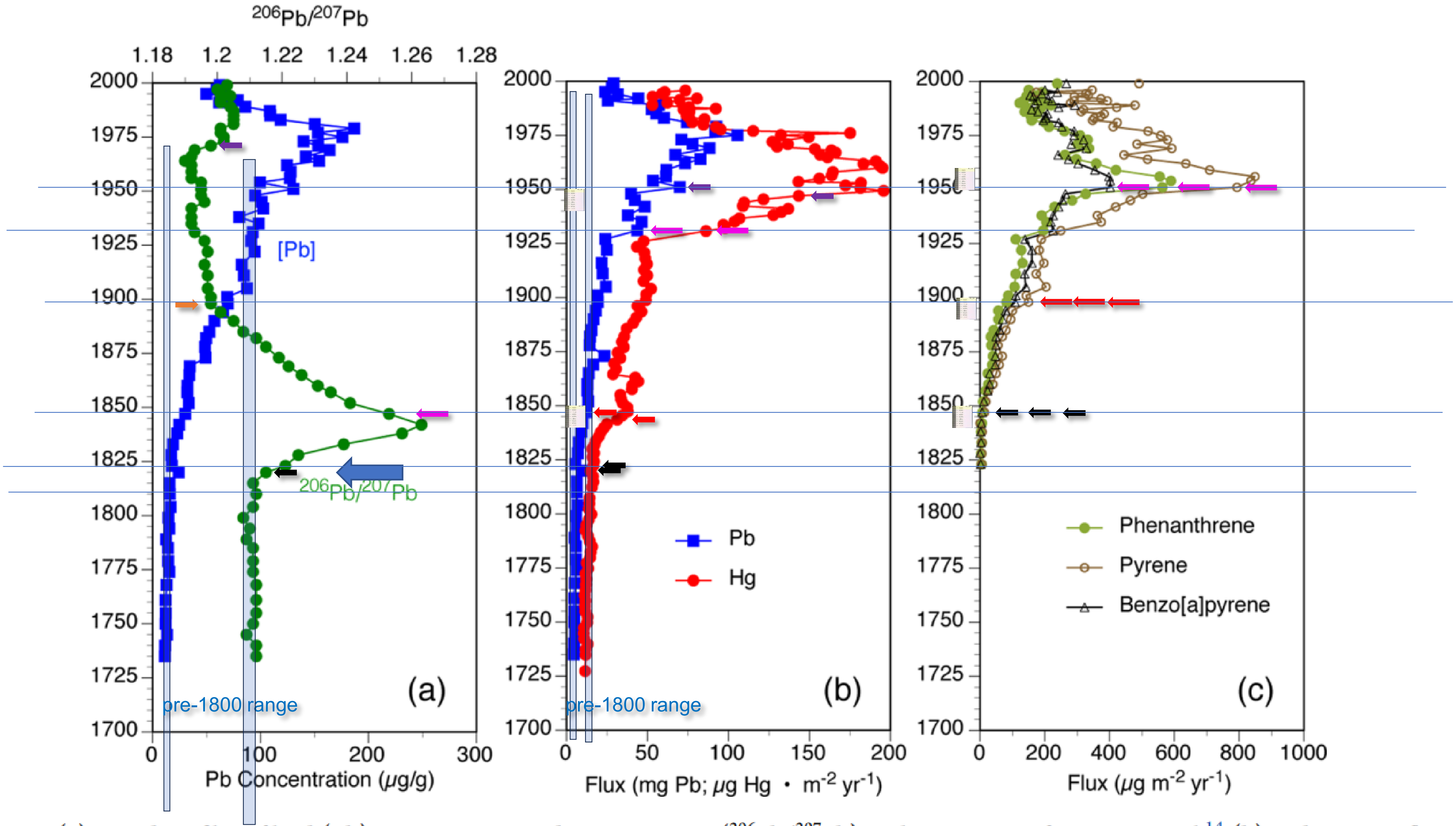

Figure 2. (a) Dated profiles of lead (Pb) concentration and isotopic ratios ($^{206}Pb/^{207}Pb$) in the PRE core; from Lima et al.[14] (b) Sedimentary fluxes of Pb and Hg in the PRE core. (c) Sedimentary fluxes of PAHs in the PRE core; from Lima et al.[15]

Fitzgerald et al. (2018).

Anderson et al. (1995).

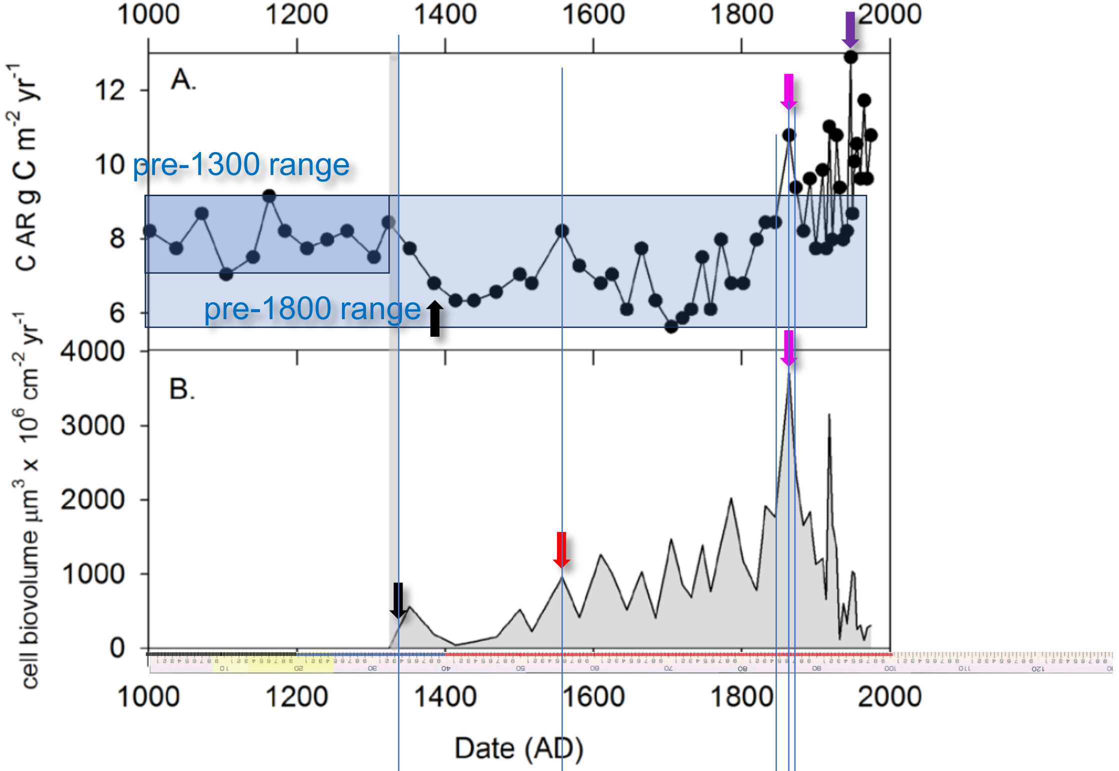

Fig. 3. The impact of development of agriculture in a boreal forest landscape (Kassjön, Northern Sweden) on bulk organic C accumulation rate (A) and the biovolume accumulation rate of a planktonic diatom, *Tabellaria flocculosa* (B). *Tabellaria* was not present in the lake prior to the initial clearance (indicated by the vertical grey bar) but expanded rapidly (<10 yr) and then continued to increase until the early 20th century when agriculture started to decline. Interestingly, the C accumulation rate does not show a similar trend, suggesting there was limited terrestrial C loss with the start of low intensity, subsistence agriculture in this boreal forest catchment. Land clearance would not have been comparable to that observed in Denmark during the Bronze Age (Fig. 6). (Drawn from data in Anderson et al., 1995.)

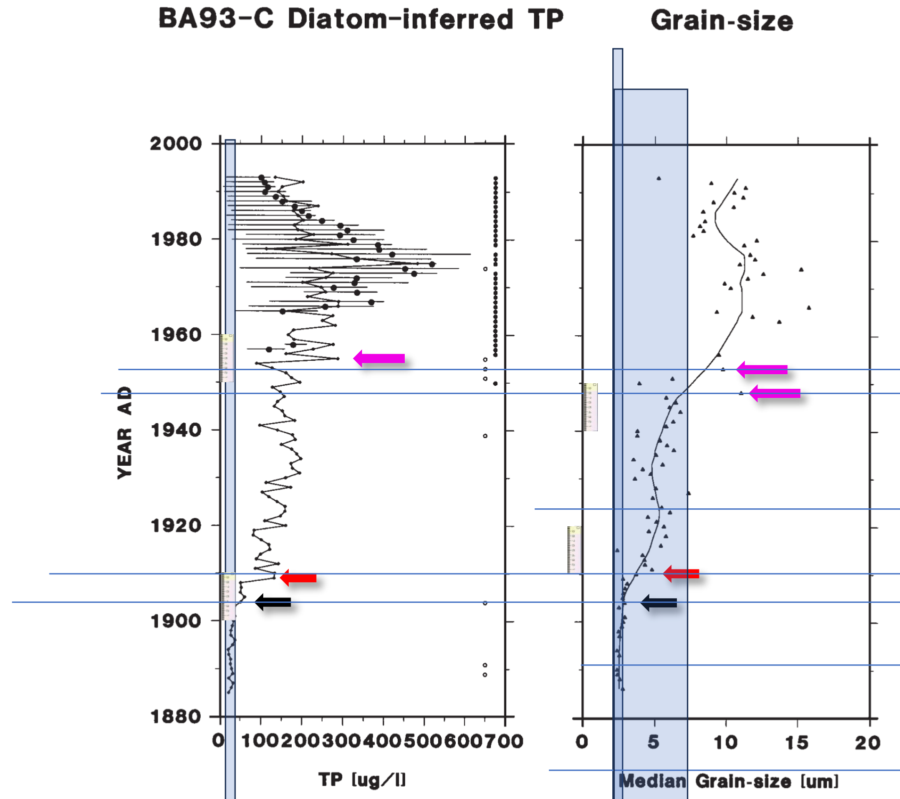

Figure 5 Measured total phosphorus (TP) during spring circulation compared to diatom-inferred TP values and median grain-size distribution in the Baldeggersee annual layers (see Lotter *et al.*, 1997c). The large filled circles show the measured spring circulation TP values for the uppermost 15 m, whereas the horizontal lines represent the annual TP range in the uppermost 15 m of the water column. The dots on the right side of the graph represent samples with close (filled dots; 2nd percentile) and good modern analogues (open dots; 5th percentile).

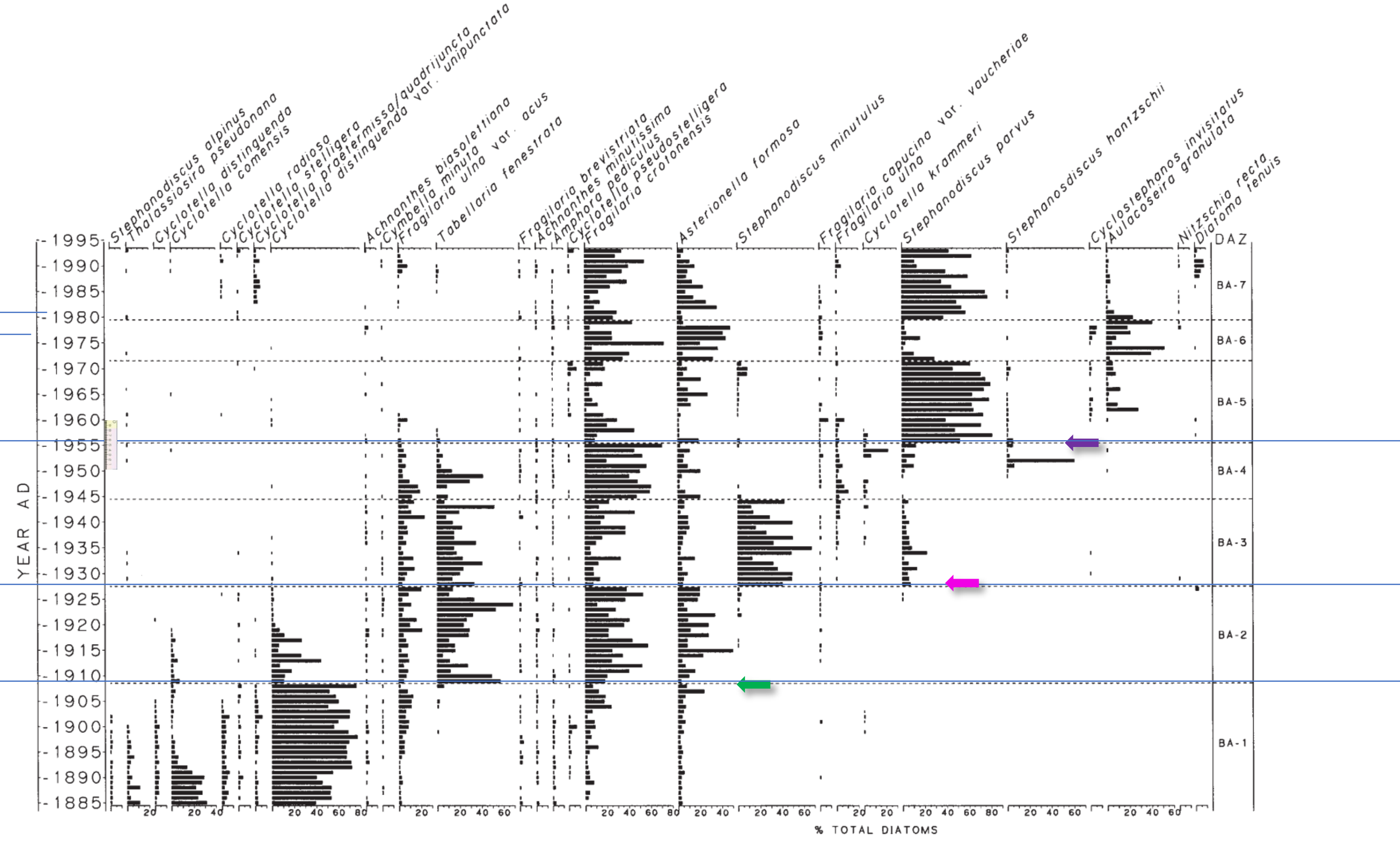

Figure 3 Diatom succession in Baldeggersee freeze-core BA93-C between 1885 and 1993. Only the major taxa are shown.

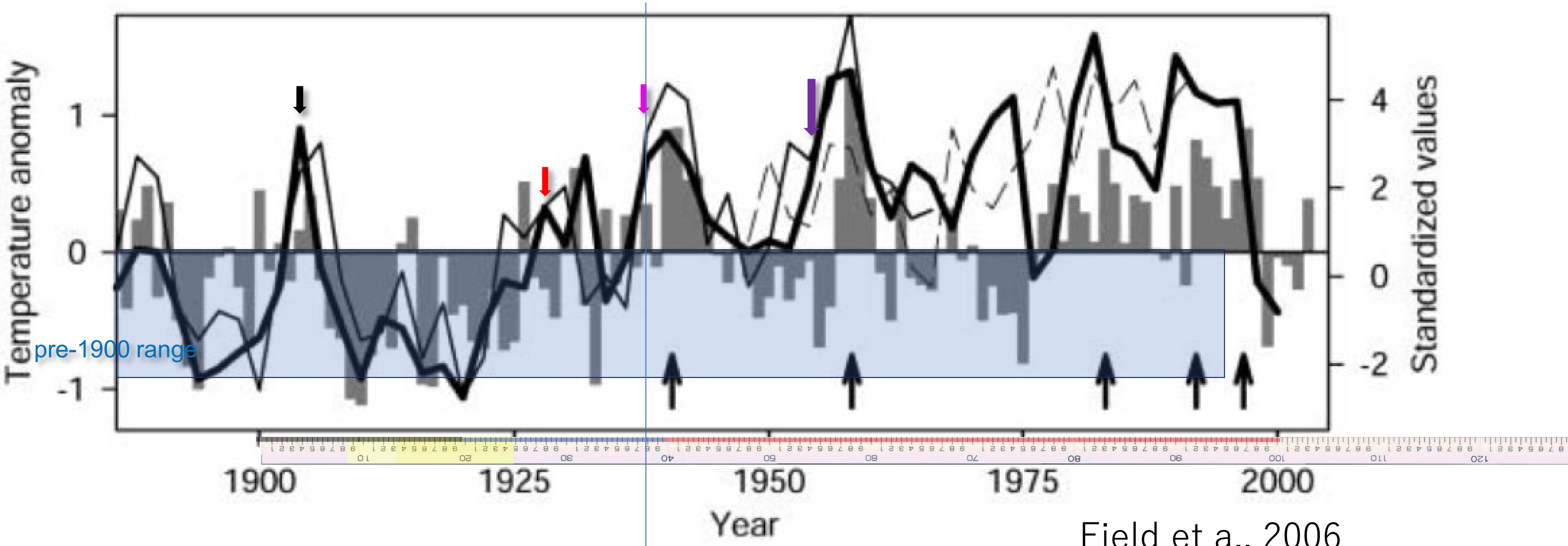

Fig. 3. Comparison of PC1 (bold solid line) with the SST record from the updated Kaplan reconstruction for the 5° × 5° grid centered at 122.5°W, 32.5°N (vertical bars) in the California Current near the SBB. PC1 is correlated with variations in SST from comparable 2-year averages of SSTs ($r^2 = 0.33$; $P < 0.001$). Arrows indicate strong El Niño events in the California Current. Also shown is the agreement of foraminiferal variations from PC1 (core BC3001) with those from two other cores in the SBB. Thin dashed line illustrates variations in averaged abundance of the same species loaded on PC1 from a core taken in 1992 and analyzed in this study. Thin solid line illustrates average of the principal species loaded on PC1 (*G. bulloides*, *N. dutertrei*, *G. ruber*, *O. universa*, and the combination of *G. calida* and *G. siphonifera*) from a core taken in 1969 and analyzed by A. Soutar and W. Berger.

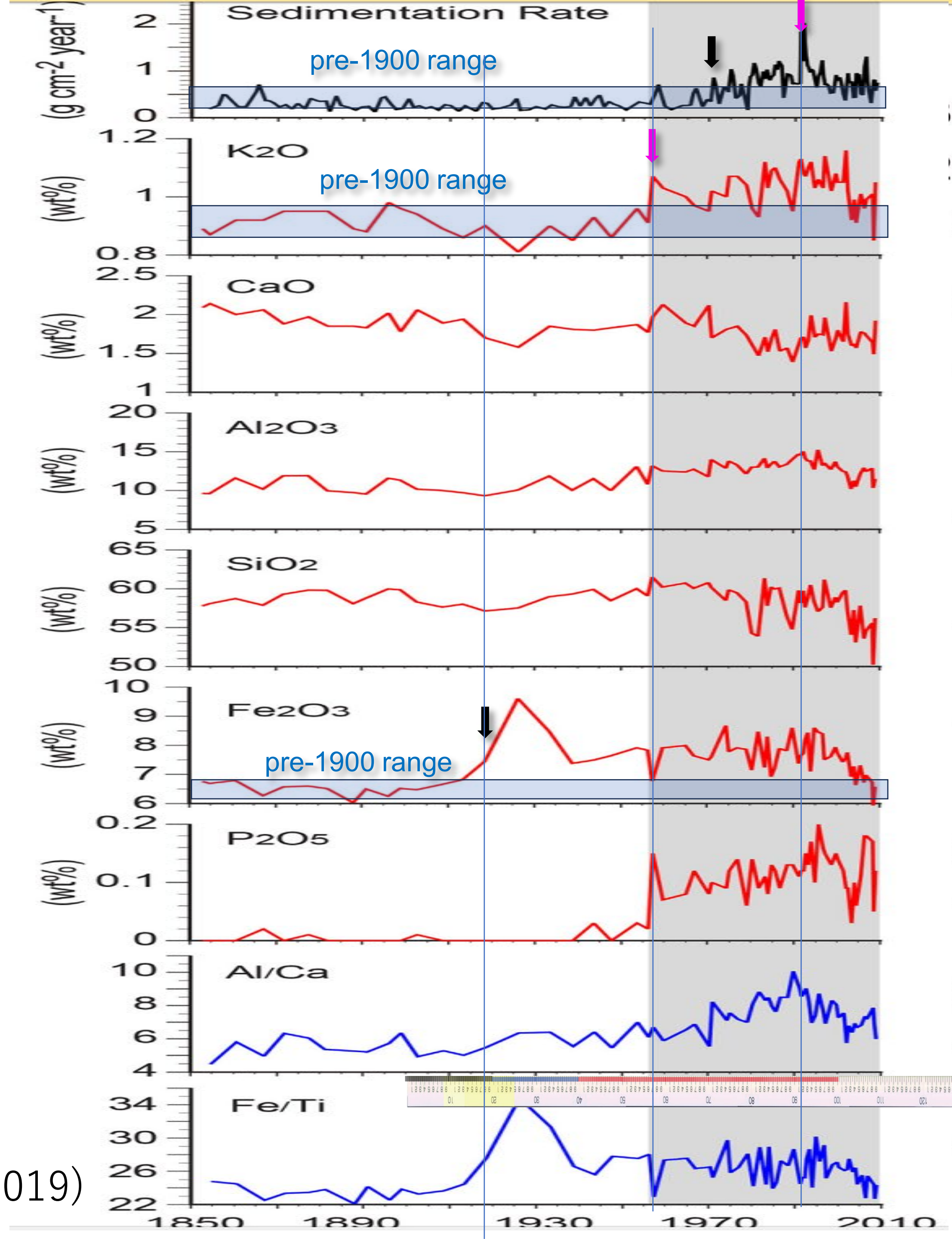

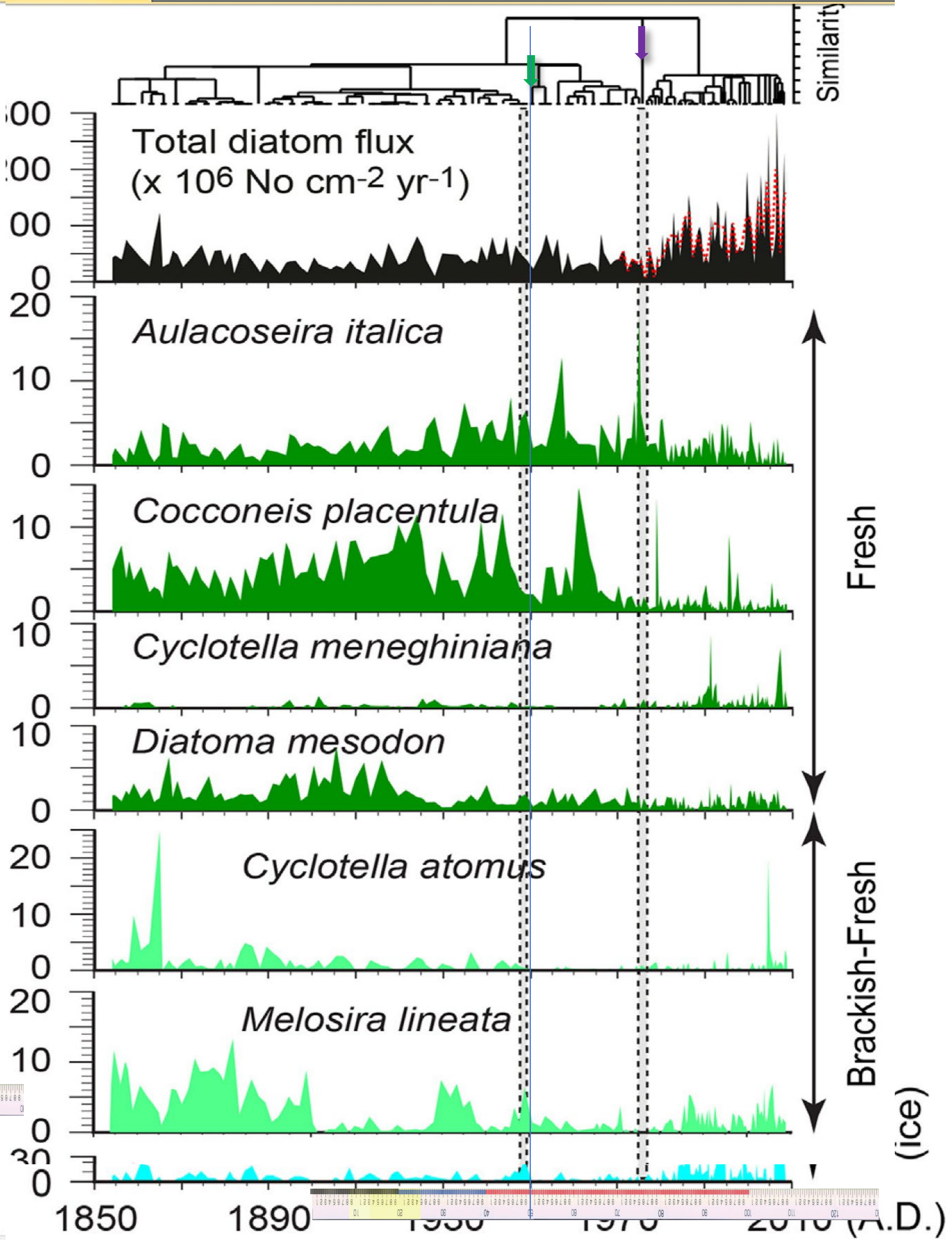

Katsuki et al. (2019)

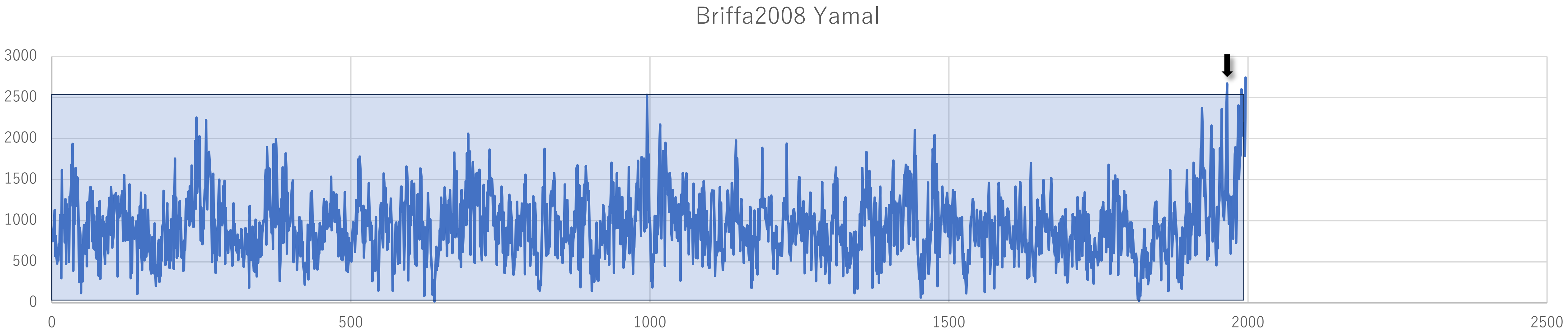

PAGES2K, 10.1098/rstb.2007.2199

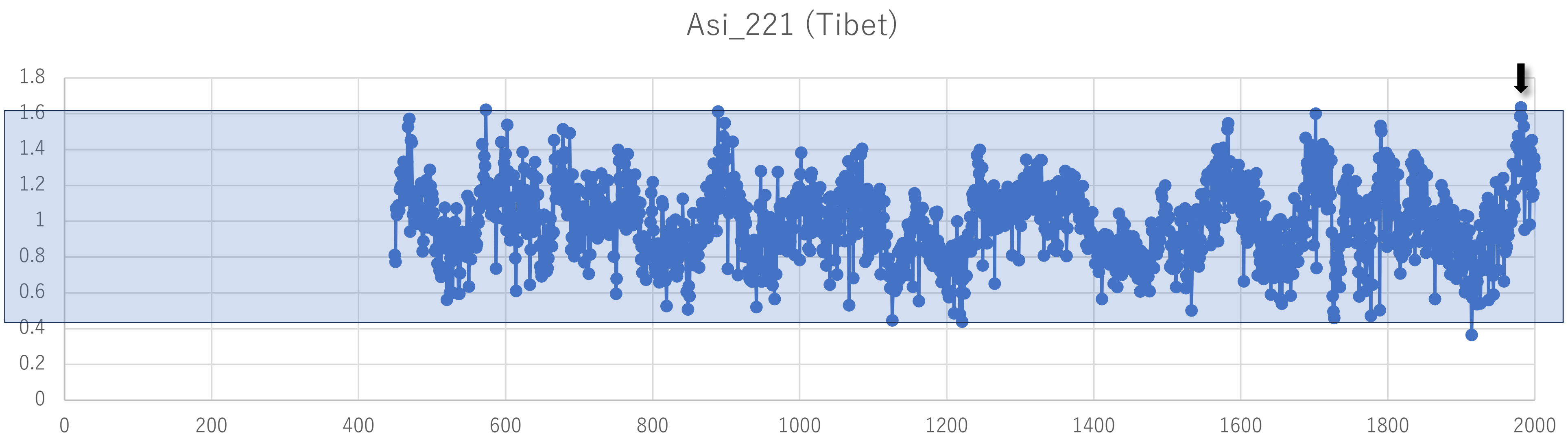

PAGES2K, 10.1098/rstb.2007.2199

Eur_4 (Slovakia)

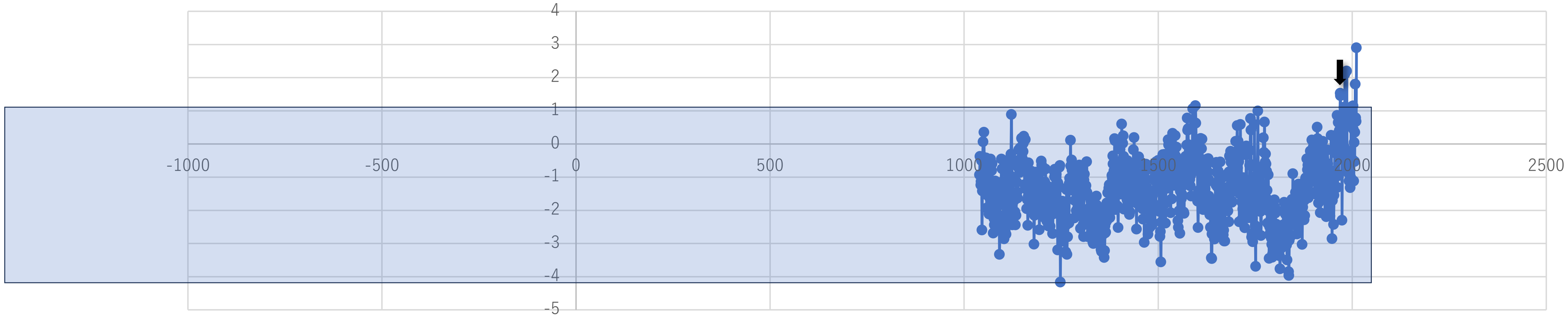

PAGES2K, 10.1098/rstb.2007.2199

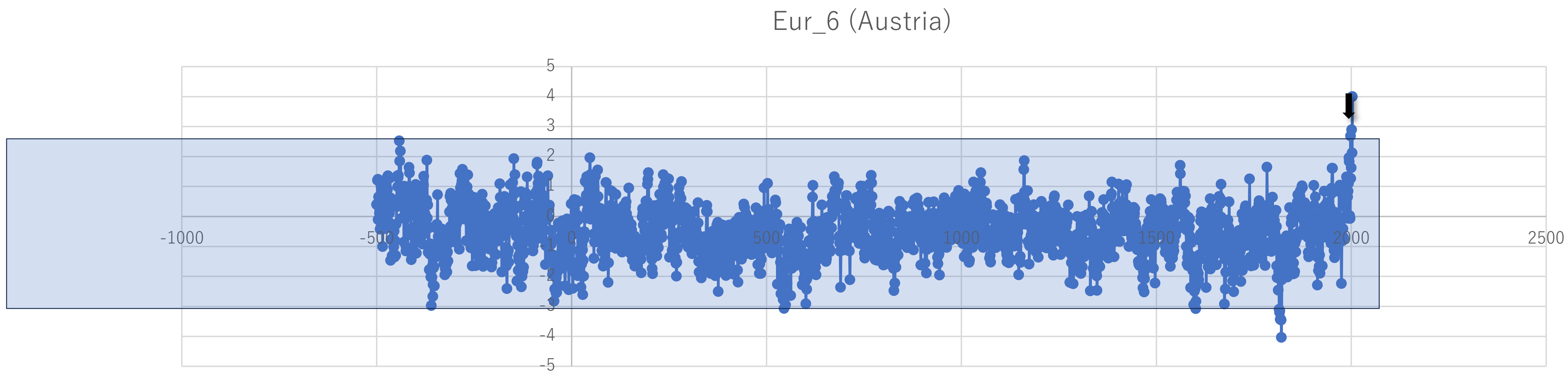

PAGES2K, 10.1098/rstb.2007.2199

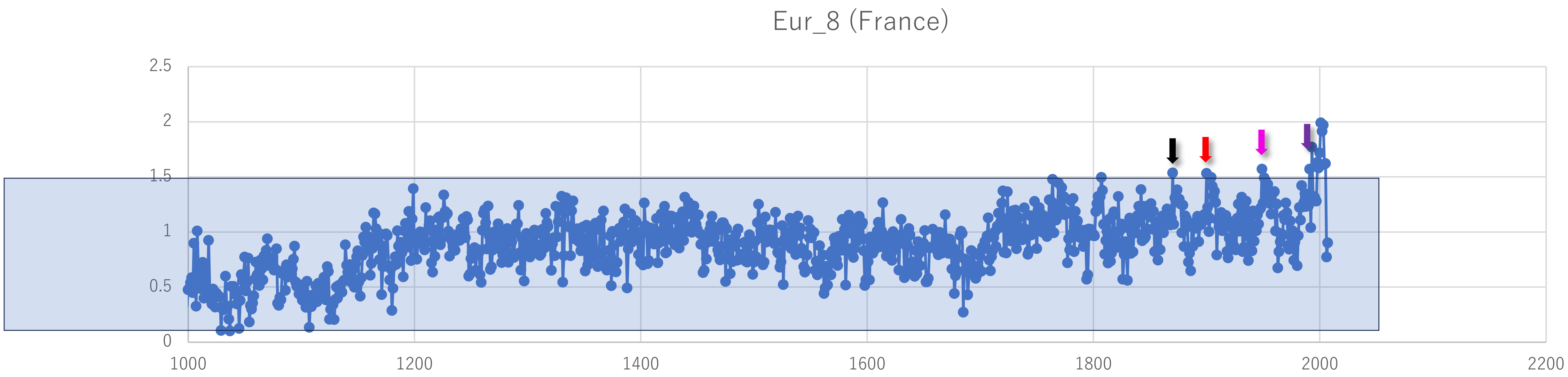

PAGES2K, 10.1098/rstb.2007.2199

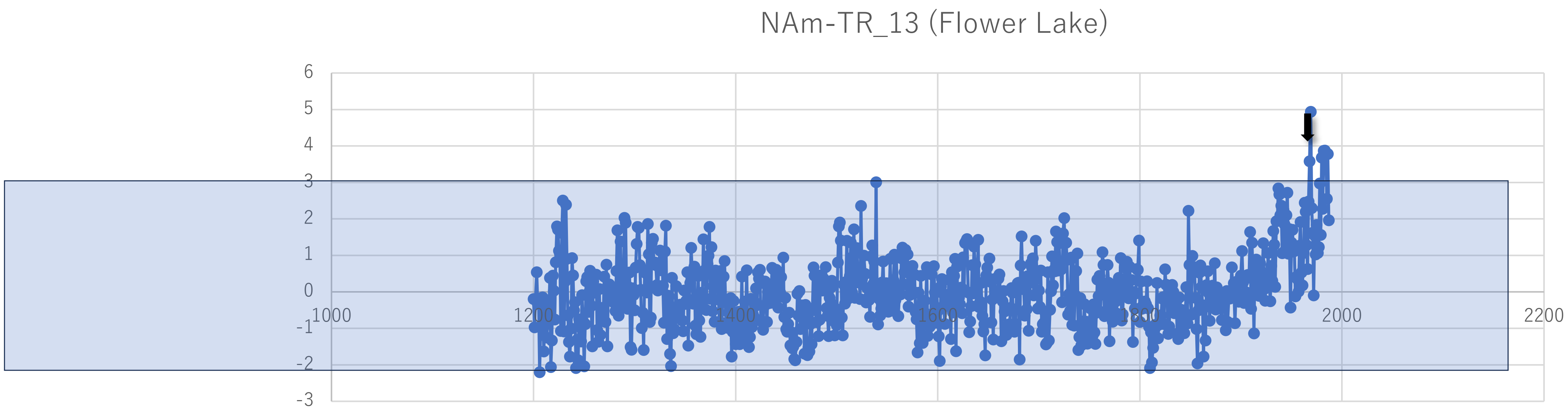

PAGES2K, 10.1098/rstb.2007.2199

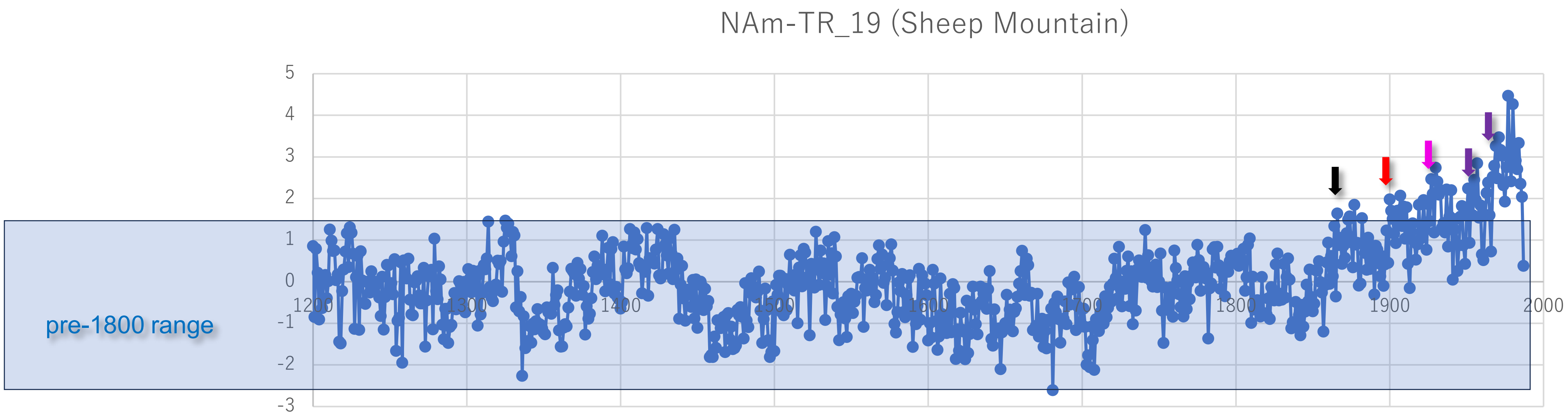

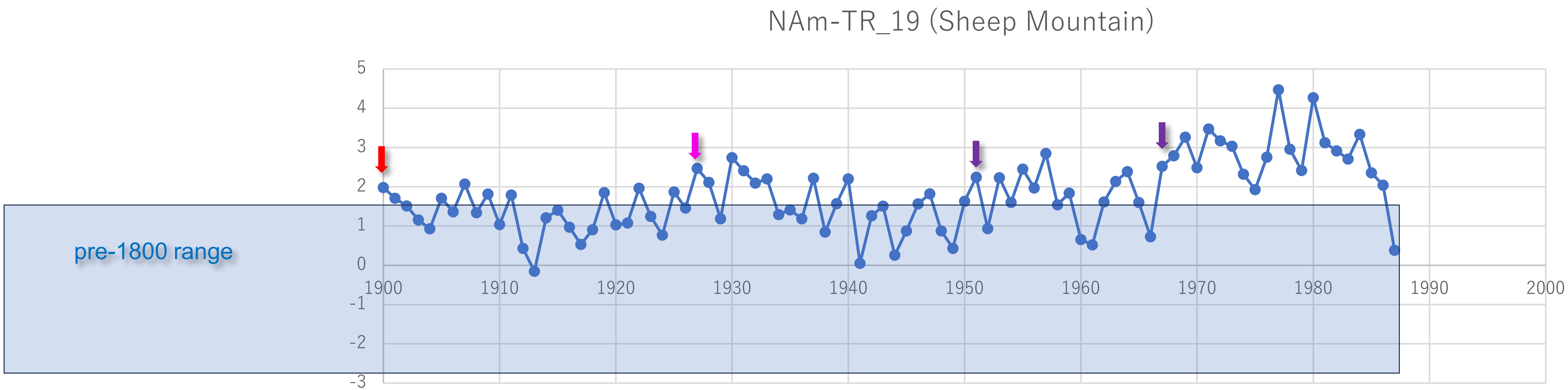

PAGES2K, 10.1098/rstb.2007.2199

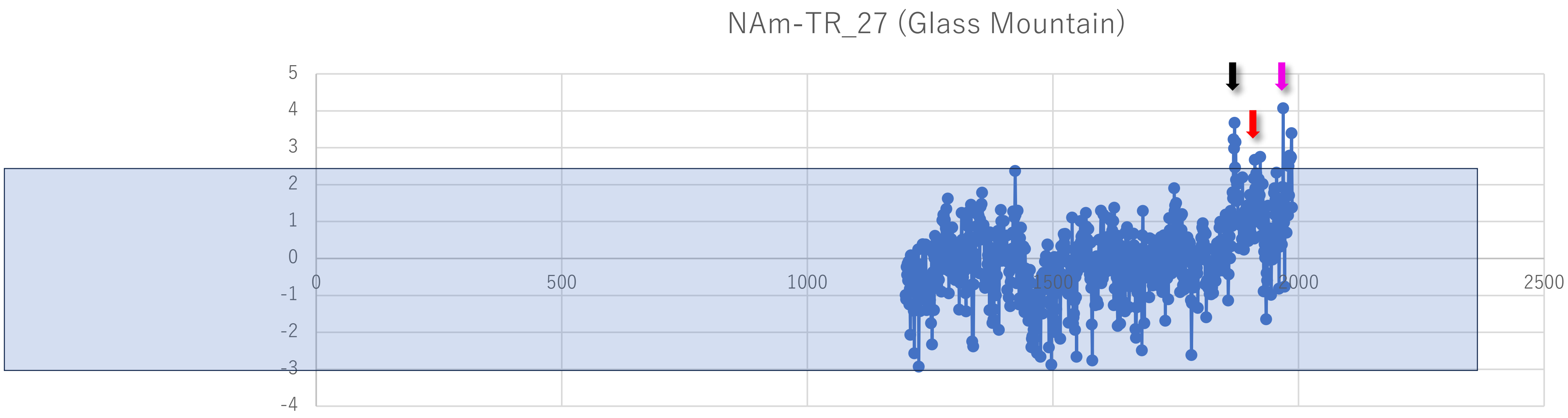

PAGES2K, 10.1098/rstb.2007.2199

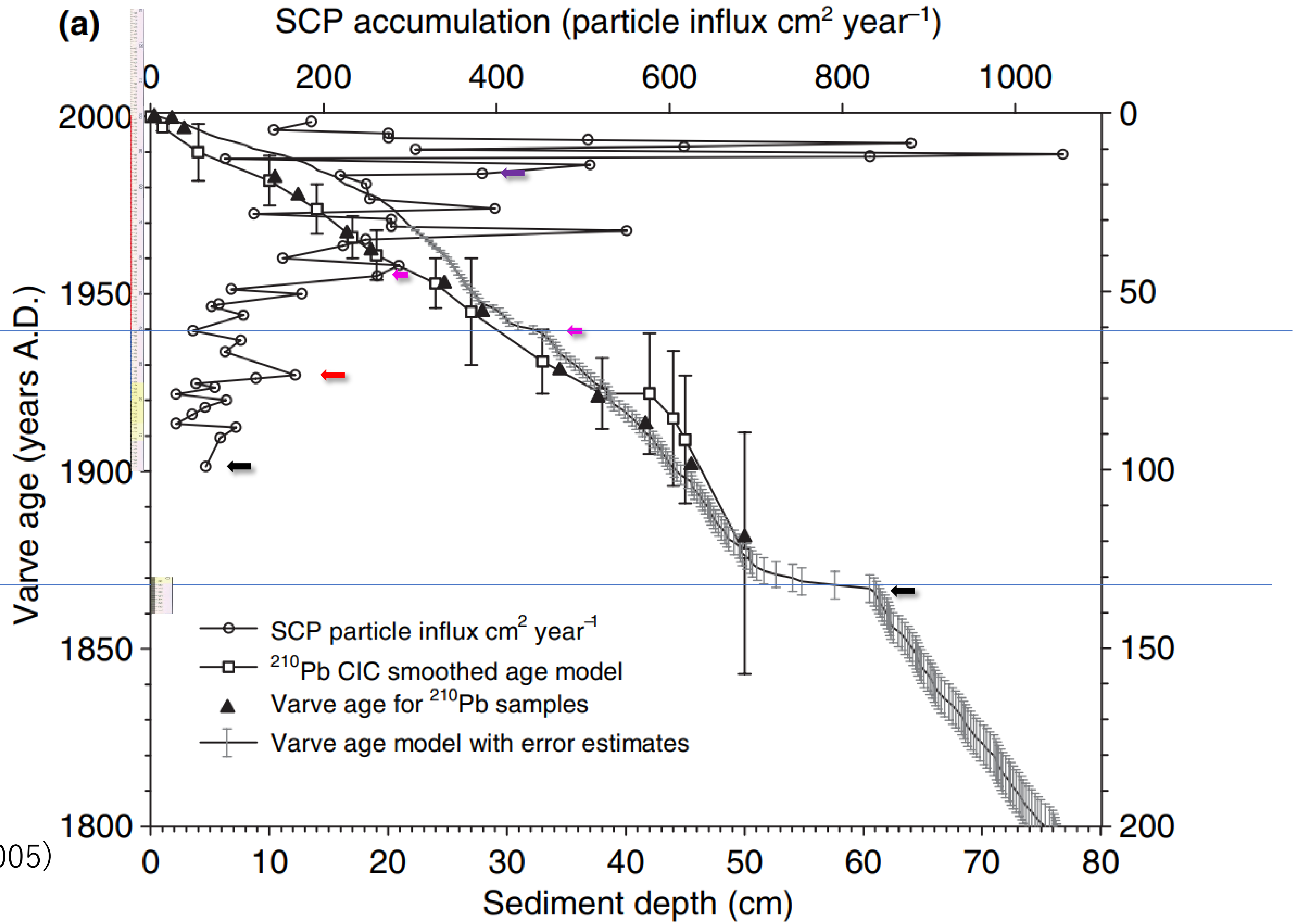

Veski et al. (2005)

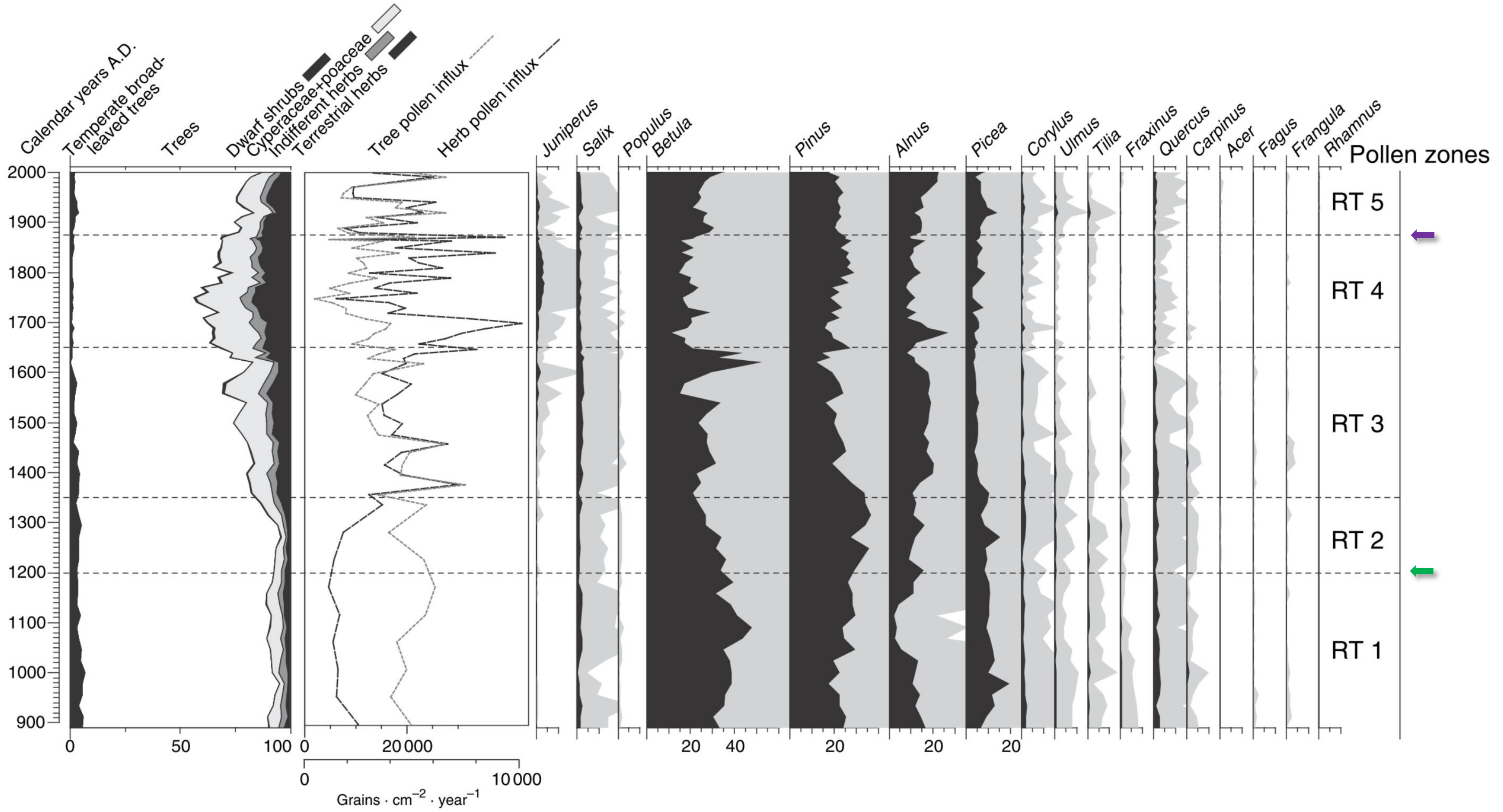

Figure 4 Pollen diagram of selected tree pollen percentages and PARs of tree and herb pollen (grains cm^{-2} year^{-1}). Lower scale for herb PARs. Pollen zones correspond to Table 1.

Veski et al. (2005)

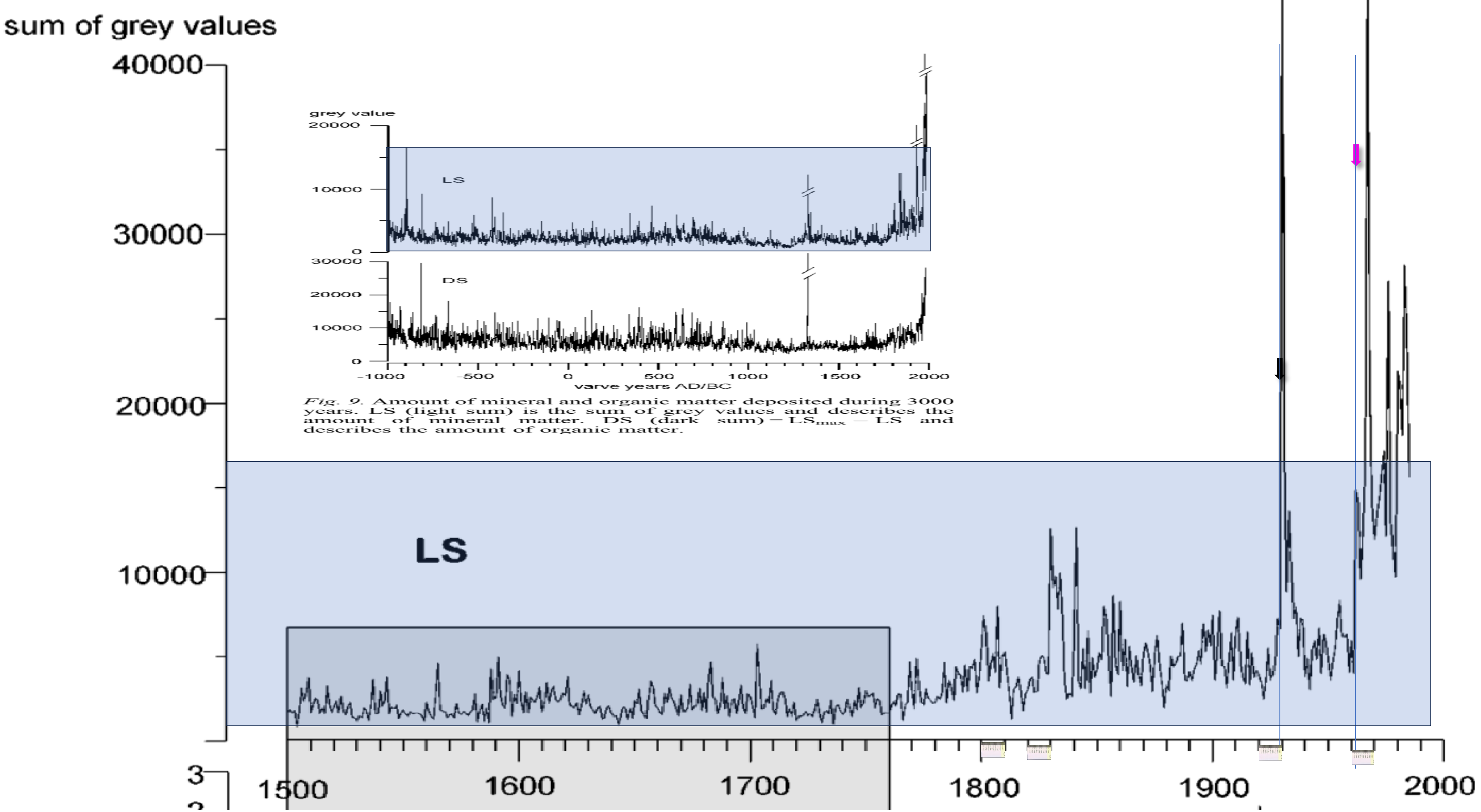

Fig. 9. Amount of mineral and organic matter deposited during 3000 years. LS (light sum) is the sum of grey values and describes the amount of mineral matter. DS (dark sum) = $LS_{max} - LS$ and describes the amount of organic matter.

Veski et al. (2005)

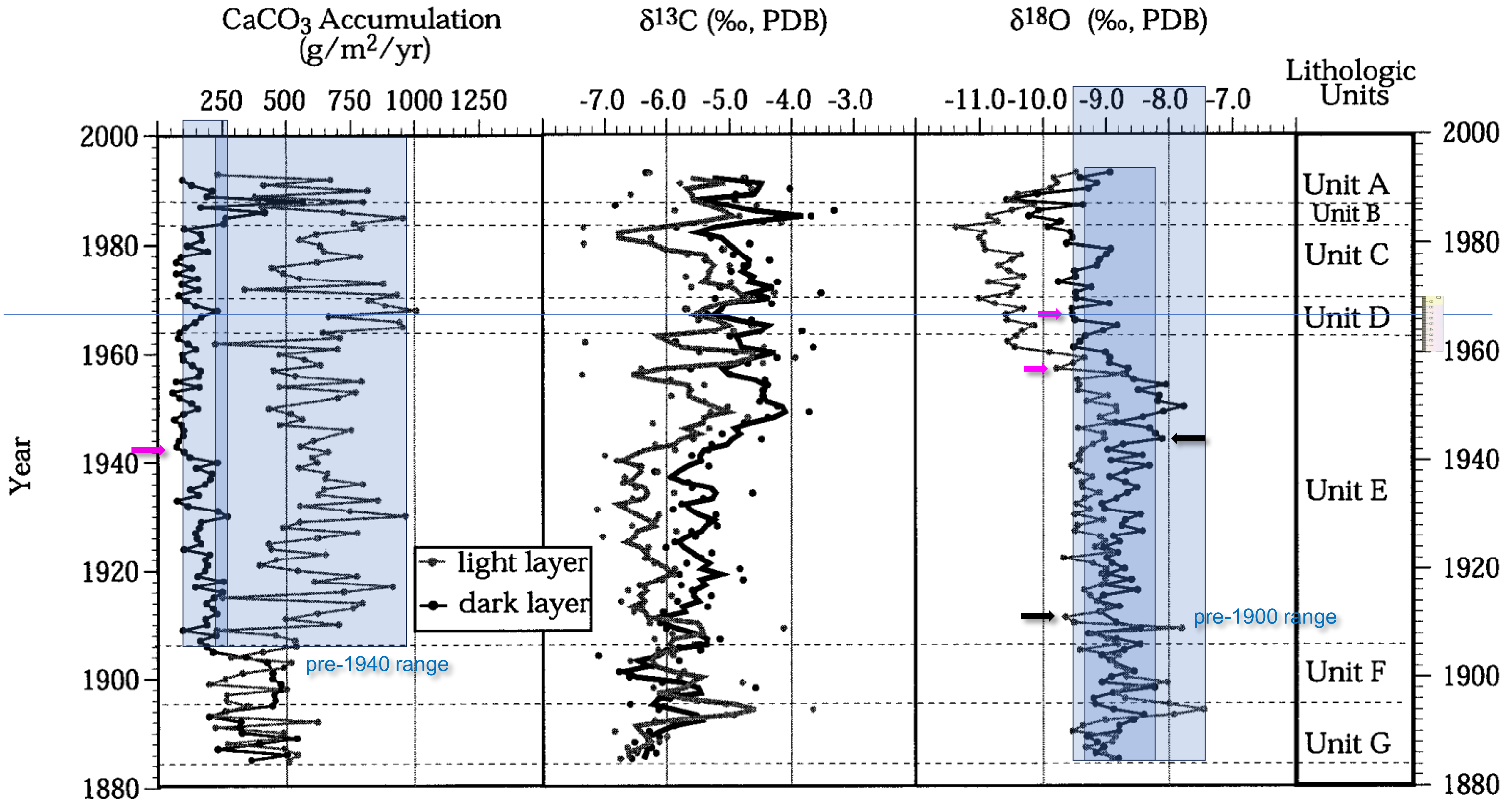

Fig. 6. Stratigraphic plots of $CaCO_3$ accumulation and stable isotope analyses of autochthonous calcite from the Baldeggersee laminated sequence divided into lithologic units. Results from the light and dark laminae are plotted separately to emphasize the distinct seasonal signals. In Units A, F, and G, difficulties in separating seasonal layers resulted in a mixing of the seasonal geochemical signals, most noticeably in the $CaCO_3$ accumulation.

Teranes et al., 1999

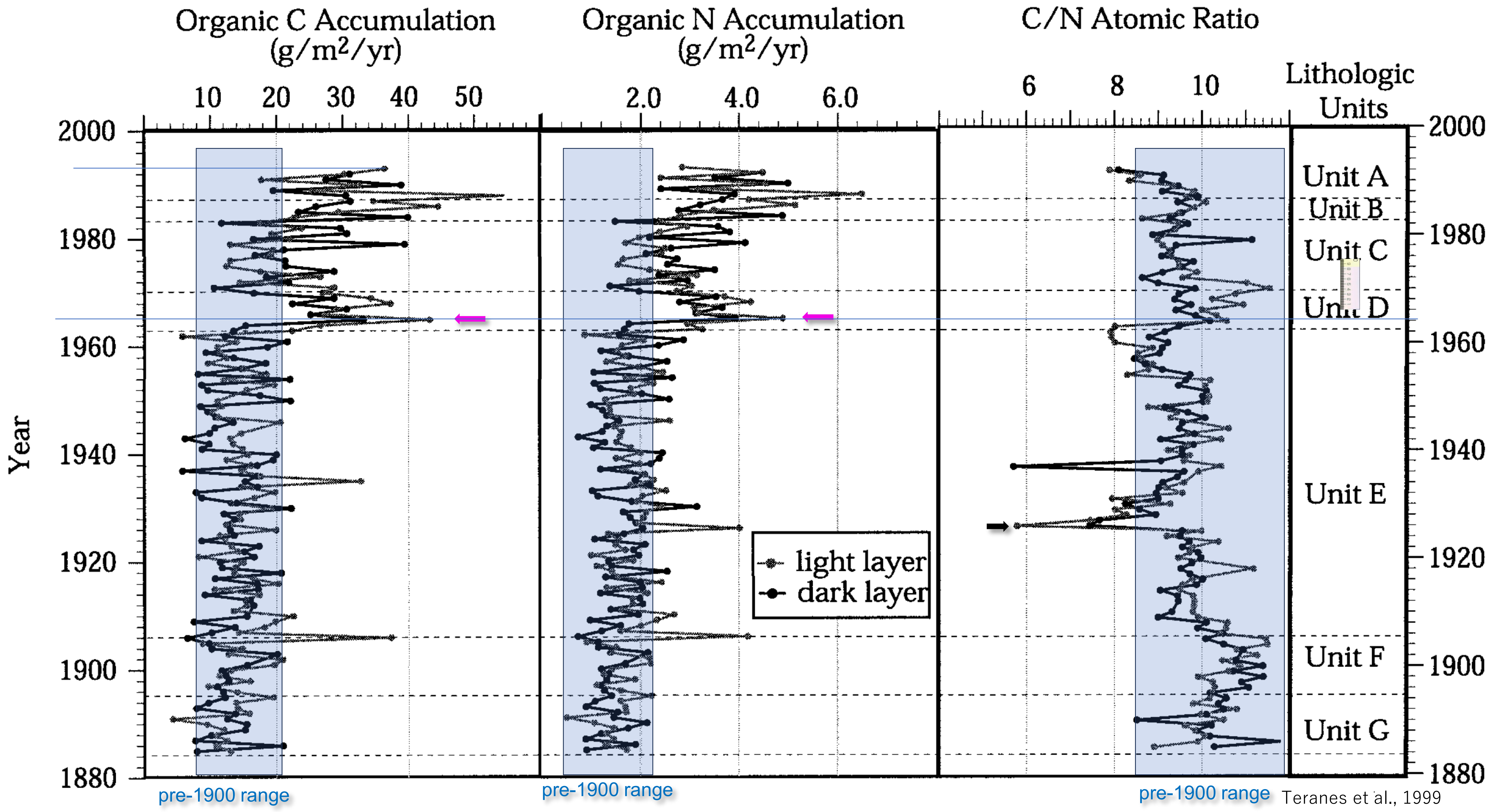

Fig. 7. Stratigraphic plots of organic C and N accumulation and C/N atomic ratios from the Baldeggersee laminated sequence divided

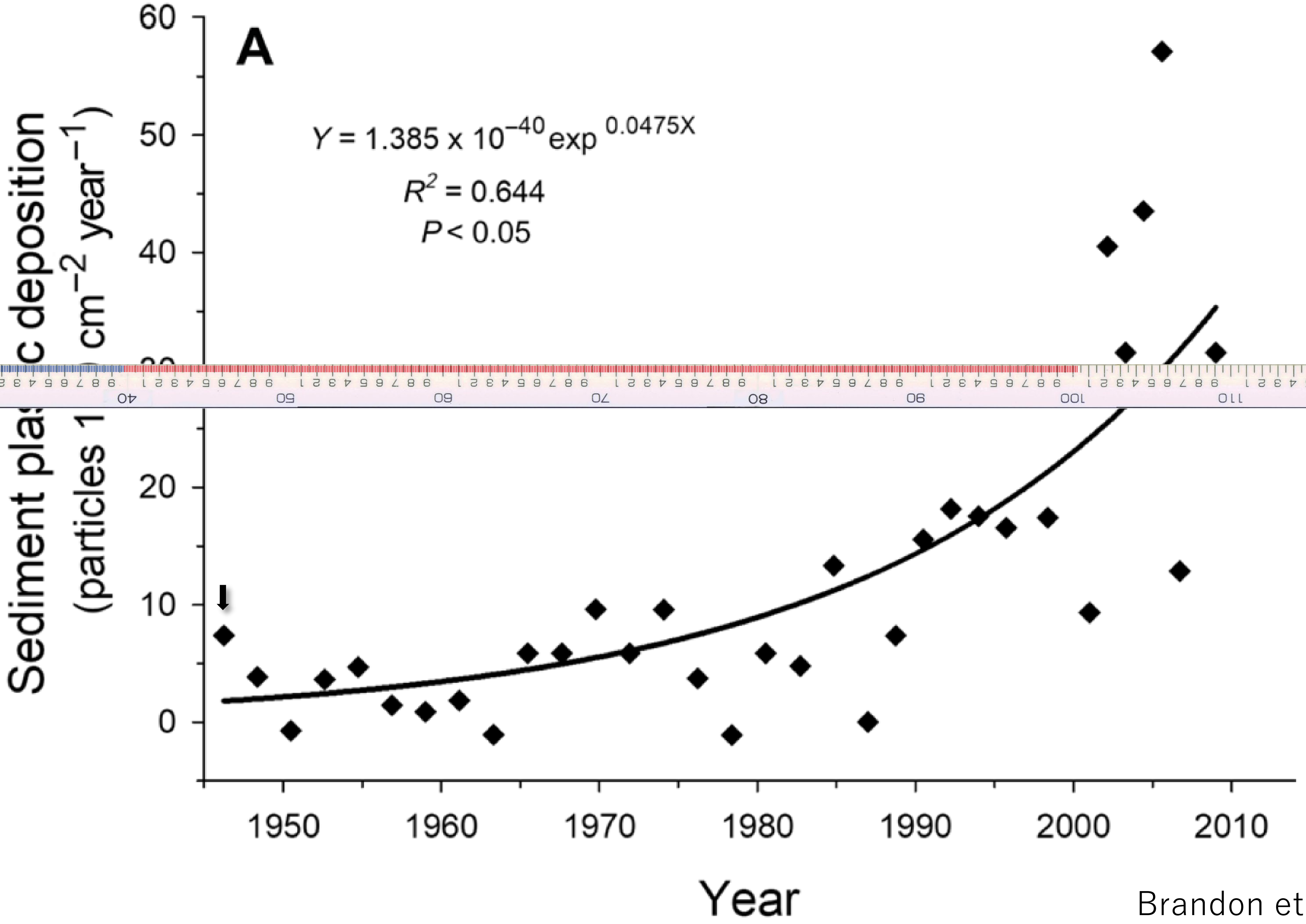

Brandon et al. (2019)

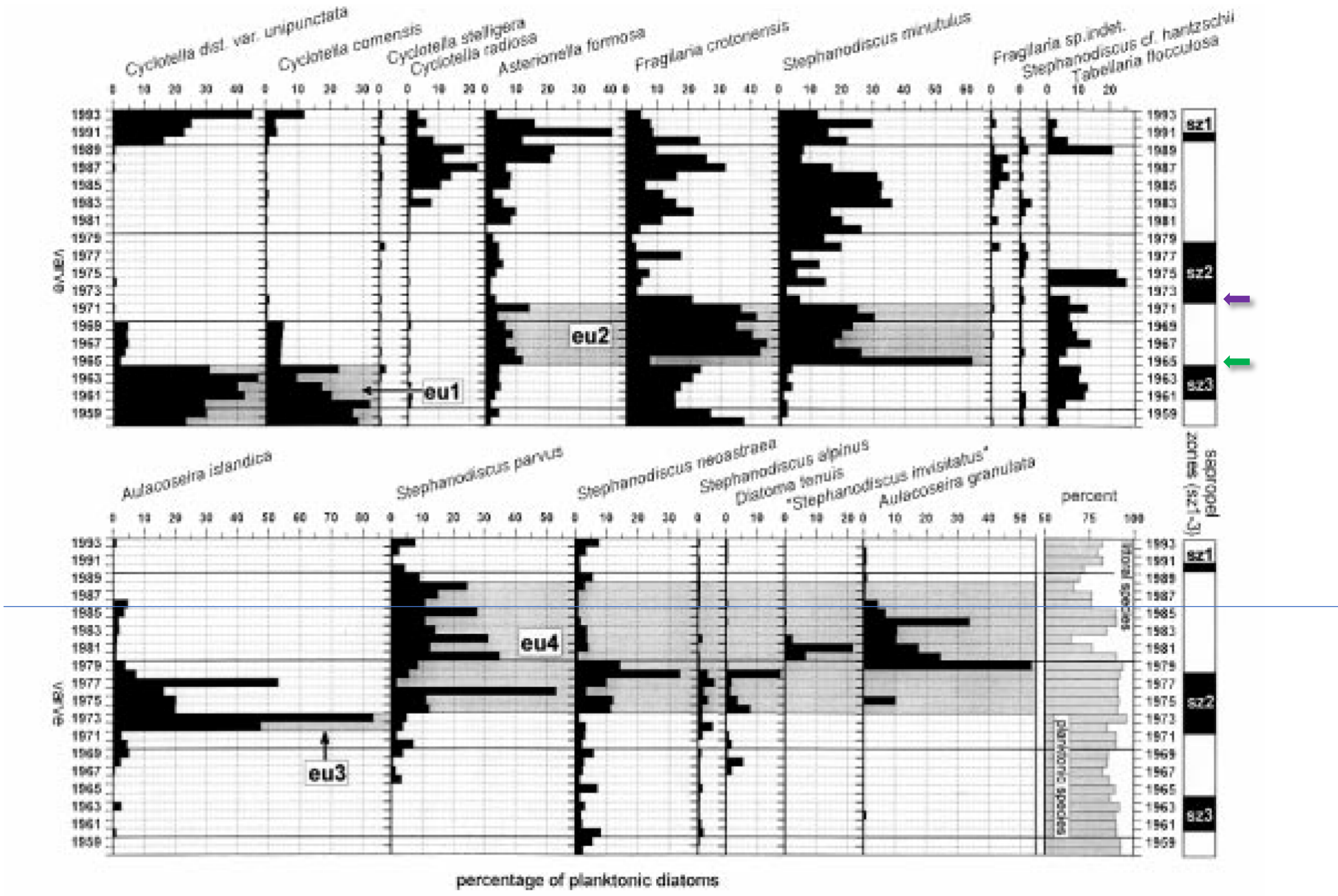

Ammersee

Figure 1. Relative frequency of planktonic diatoms in the varves of Ammersee (expressed as percentage of total planktonic diatoms) and the relative frequency of planktonic and littoral diatom species. Shaded zones 'eu 1' to 'eu 4' represent the four phases of the 'basic species sequence of eutrophication' model (see text). On the right side, three varve periods with sapropel formation are marked (sz 1 to sz 3).

Alefs and Müller (1999)

Starnberger See

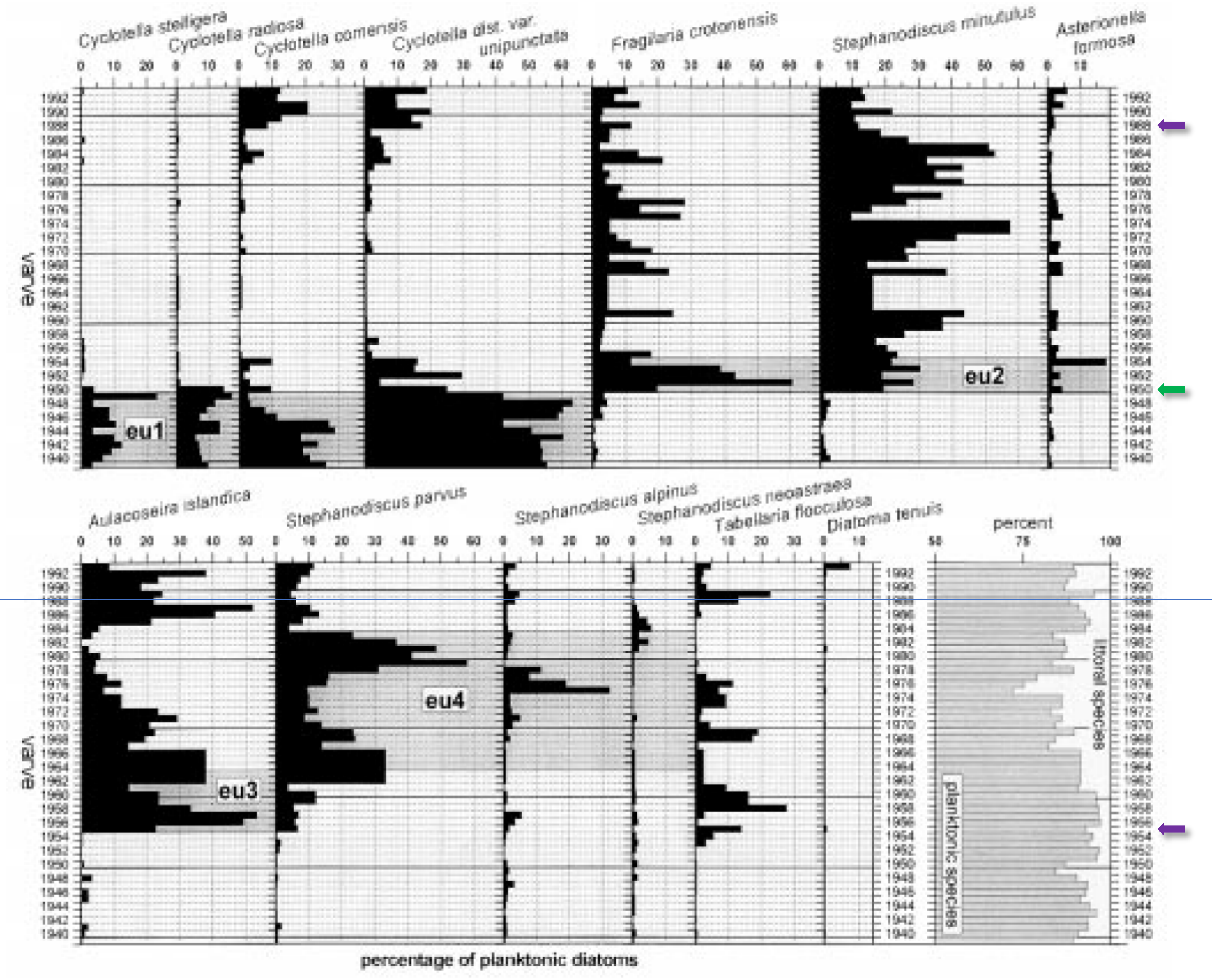

Figure 2. Relative frequency of planktonic diatoms in the varves of Starnberger See (expressed as percentage of total planktonic diatoms) and the relative frequency of planktonic and littoral diatom species. Shaded zones 'eu 1' to 'eu 4' represent the four phases of the 'basic species sequence of eutrophication' model (see text).

Alefs and Müller (1999)

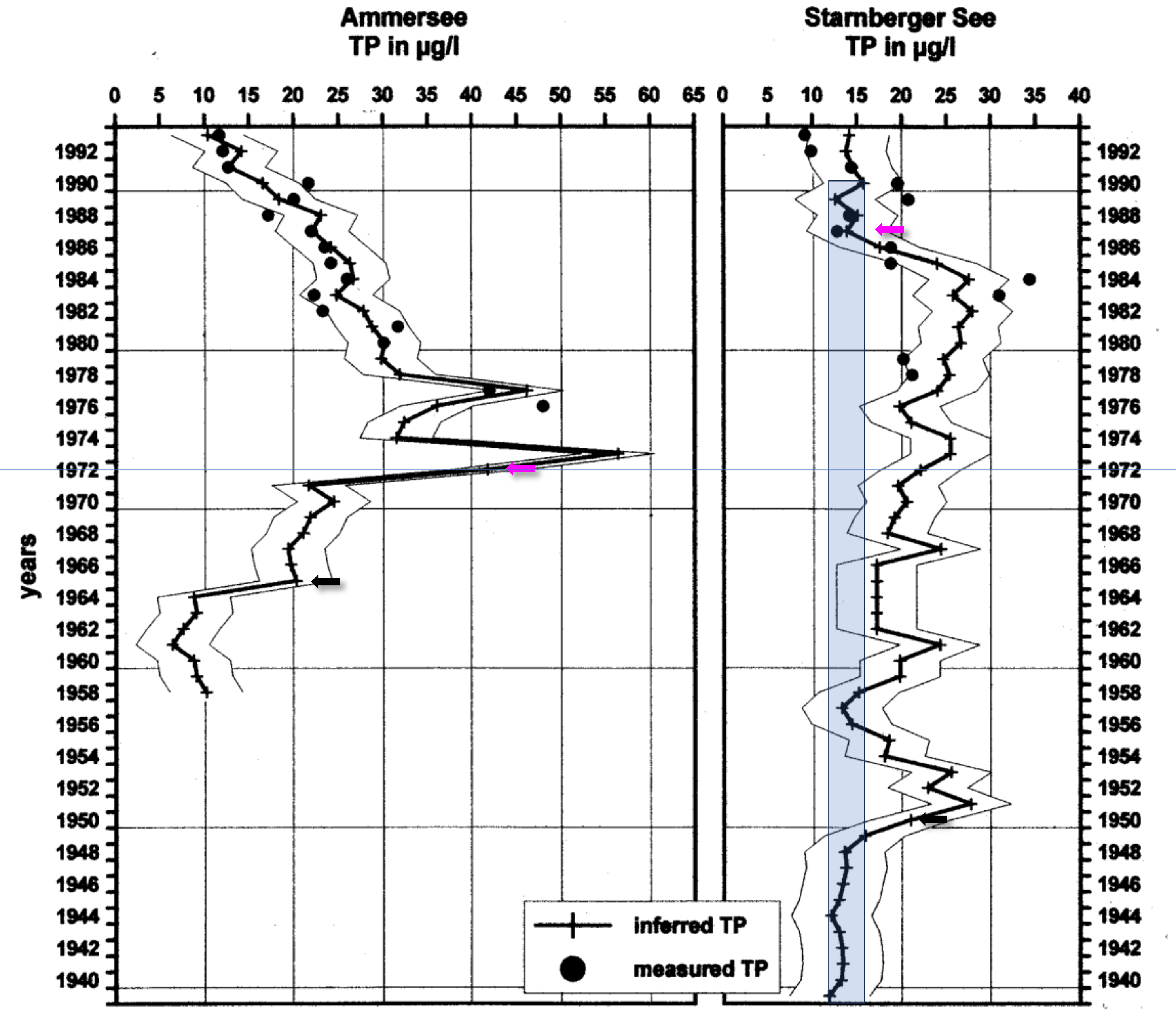

Figure 5. Diatom-inferred TP concentrations (WA-value, inverse regression) of Ammersee and Starnberger See with the respective error of prediction interval. Measured annual means of epilimnetic TP concentrations are plotted as dots. The correlation coefficient between measured and inferred TP is 0.91 in Ammersee and 0.77 in Starnberger See.

Alefs and Müller (1999)

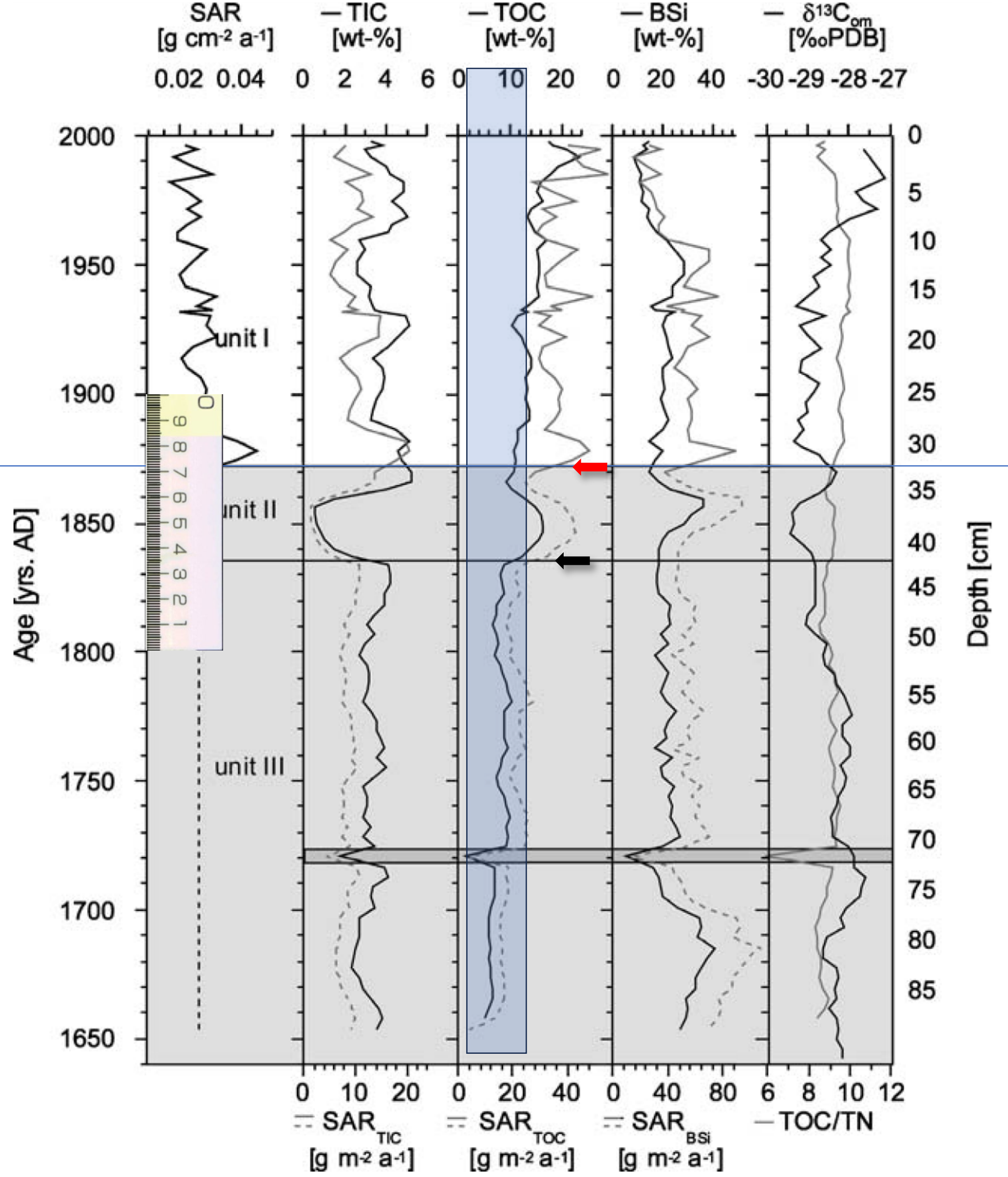

Figure 6. Diagram of sediment mass accumulation rates (SAR), inorganic and organic carbon (TIC, TOC) with respective influx rates (SAR_{TIC}, SAR_{TOC}), biogenic silica (BSi, given as SiO_2) with influx rates (SAR_{BSi}), TOC/TN weight ratio and $\delta^{13}C$ of organic matter ($\delta^{13}C_{om}$). Dashed lines indicate sediment mass accumulation rates based on linearly interpolation. Sediment units I to III are indicated. Shading points to the non-laminated part of the profile, where ages are linearly extrapolated. The dark shaded bar marks a sandy layer only occurring in core Sac 99-4.

Lüder (2006)

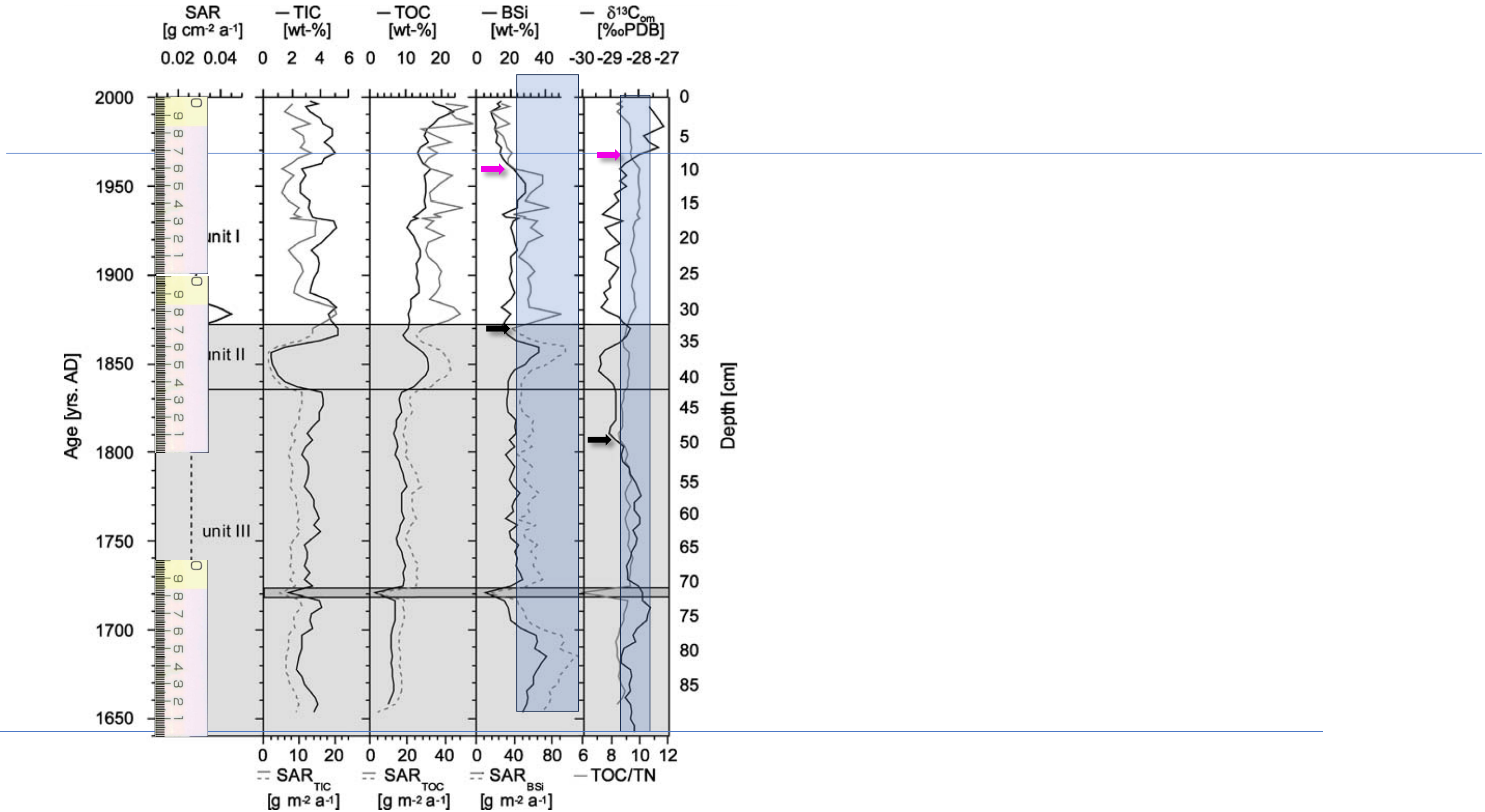

Figure 6. Diagram of sediment mass accumulation rates (SAR), inorganic and organic carbon (TIC, TOC) with respective influx rates (SAR_{TIC}, SAR_{TOC}), biogenic silica (BSi, given as SiO_2) with influx rates (SAR_{BSi}), TOC/TN weight ratio and $\delta^{13}C$ of organic matter ($\delta^{13}C_{om}$). Dashed lines indicate sediment mass accumulation rates based on linearly interpolation. Sediment units I to III are indicated. Shading points to the non-laminated part of the profile, where ages are linearly extrapolated. The dark shaded bar marks a sandy layer only occurring in core Sac 99-4.

Lüder (2006)

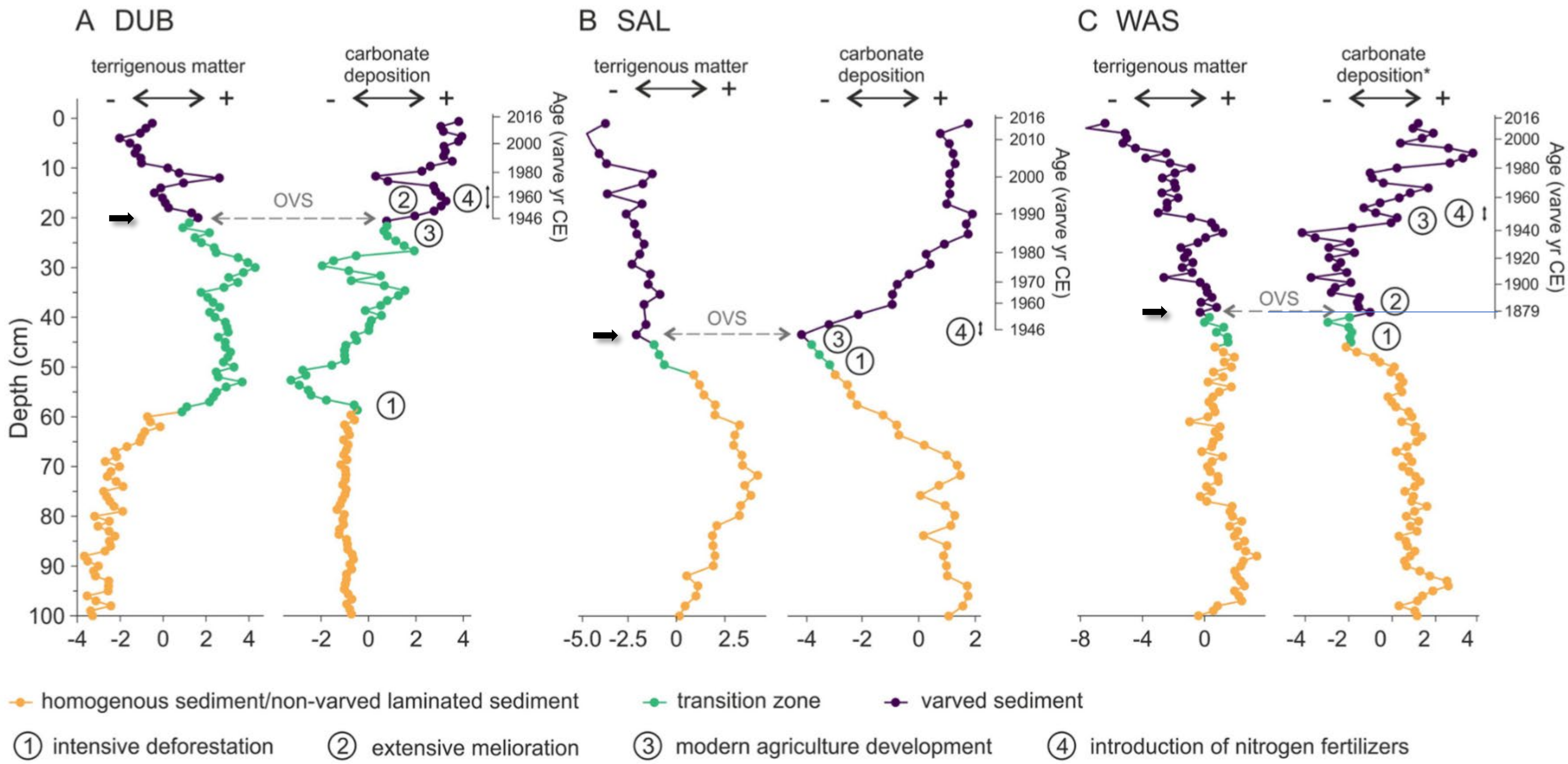

Fig. 6. Variability of terrigenous inputs (PC1) and deposition of carbonates (PC2) plotted against depth with additional age scale for DUB (A), SAL (B), and WAS (C). Visualization of carbonates deposition in WAS (*) was inverted to unify descriptions. OVS stands for the onset of varved sediments.

Poraj-Górska et al., 2021

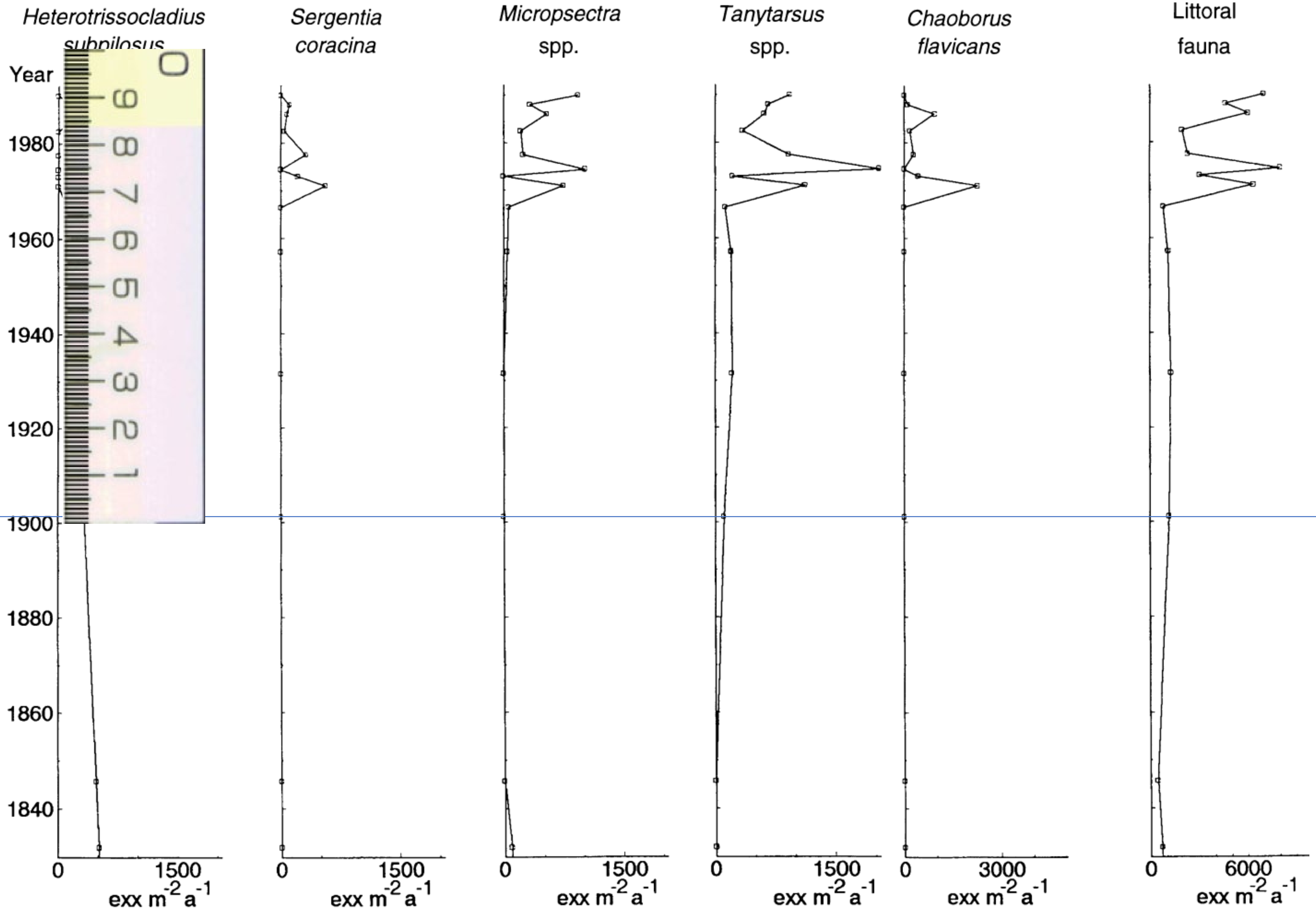

Figure 7. Annual accumulation of certain chironomid species, *Chaoborus flavicans* and littoral chironomids during the past 150 years (core RS3).

Itkonen et al. (1999)

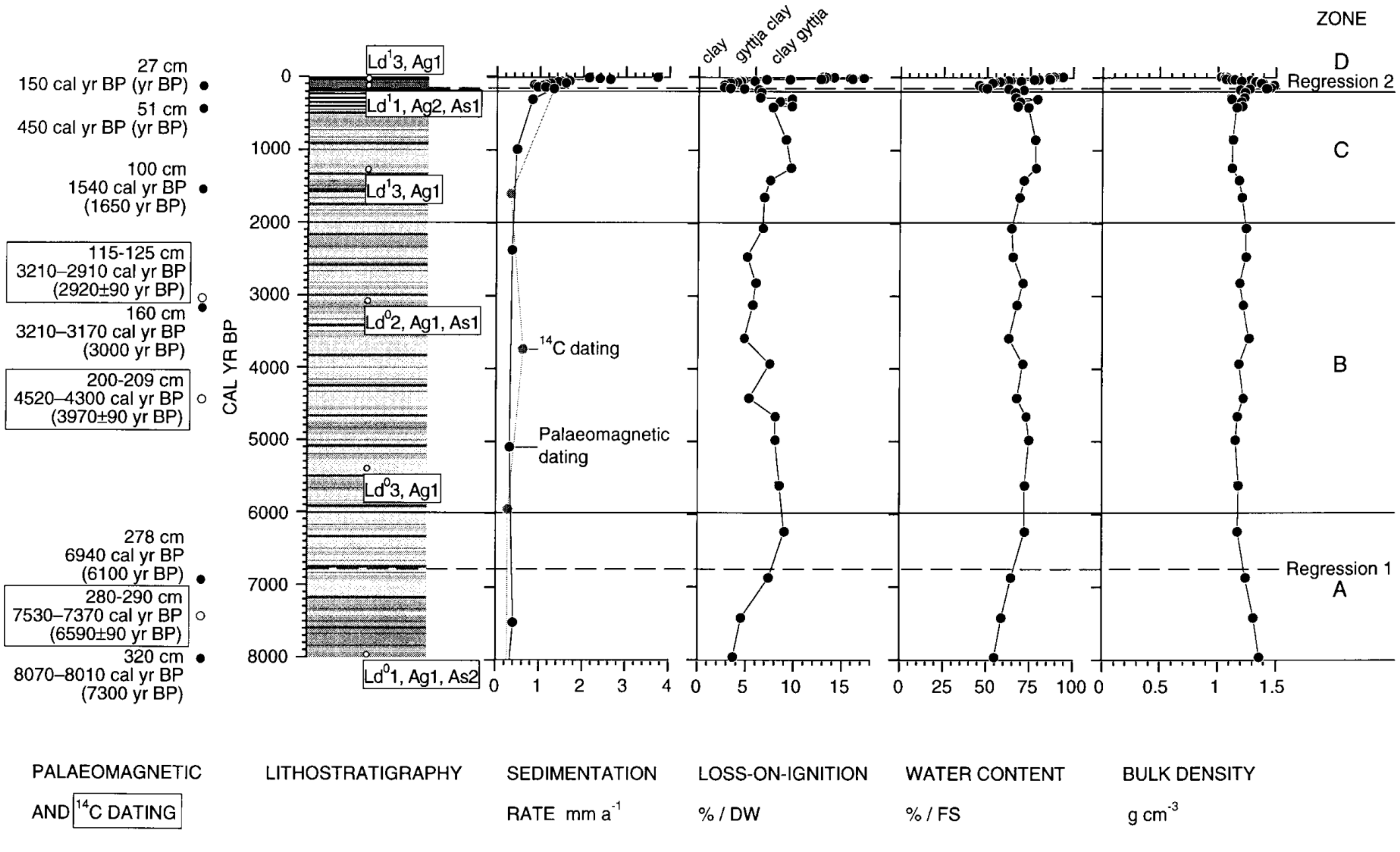

Figure 2. Palaeomagnetic dating, lithostratigraphy (Troels-Smith system; Aaby & Berglund, 1986) and physical properties of the sediment core RS2 from Lake Päijänne. The lithostratigraphy is as follows: banded gyttja clay (up to ca. 7500 cal yr BP); banded clay gyttja (ca. 7500–440 cal yr BP); varved clay gyttja (ca. 440–159 years ago); varved gyttja clay (159–45 years ago); and varved black-coloured clay gyttja (45–0 years ago). The broken lines indicate regressions: (1) the natural discharge of the Ancient Lake Päijänne ca. 7000 cal yr BP and (2) the artificial lowering of the water level by 1.2 m in 1832–1838. Zones A–D: see phases in discussion.

Itkonen et al. (1999)

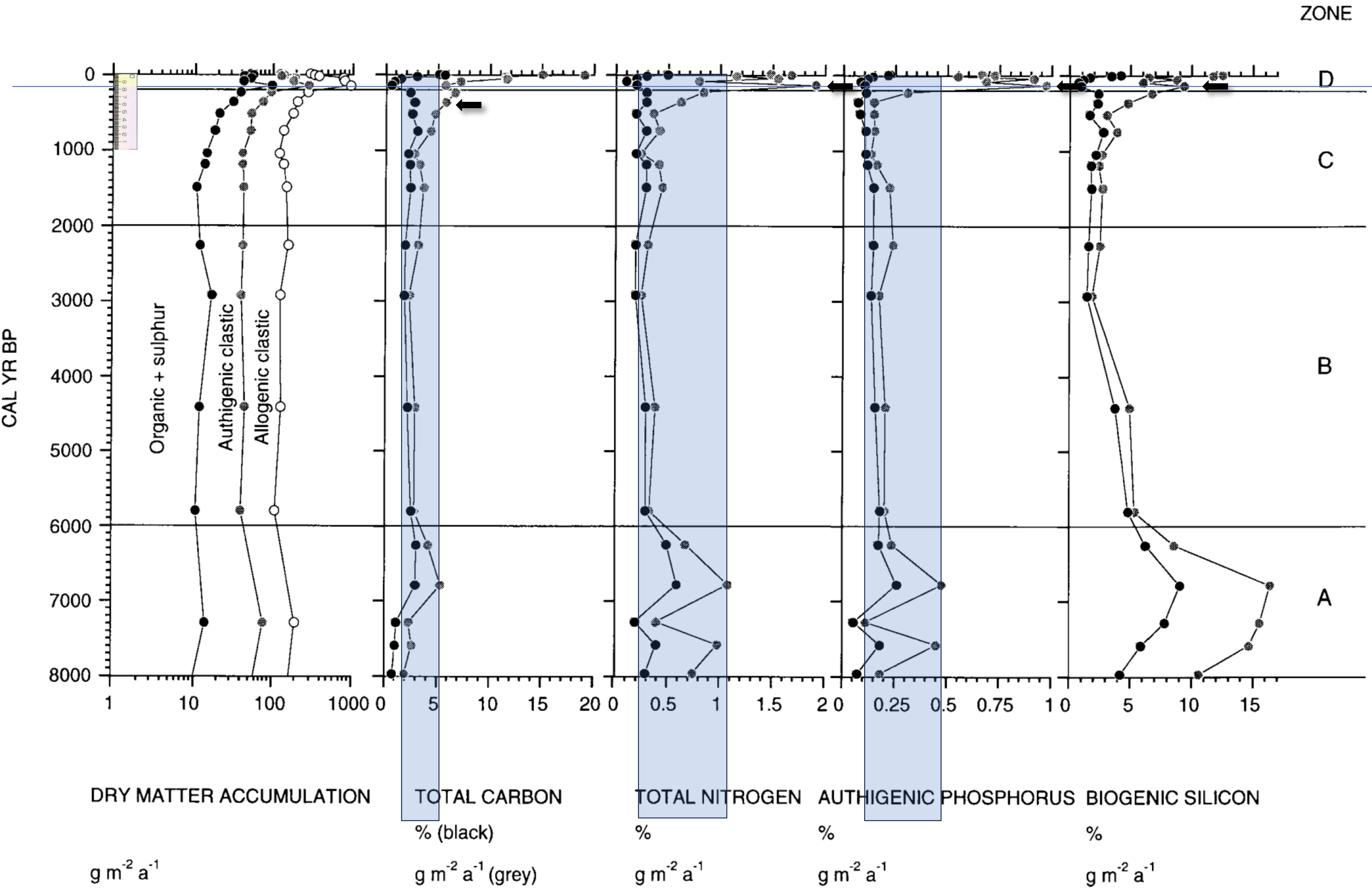

Figure 3. Components of dry matter accumulation and selected elements in core RS2.

Itkonen et al. (1999)

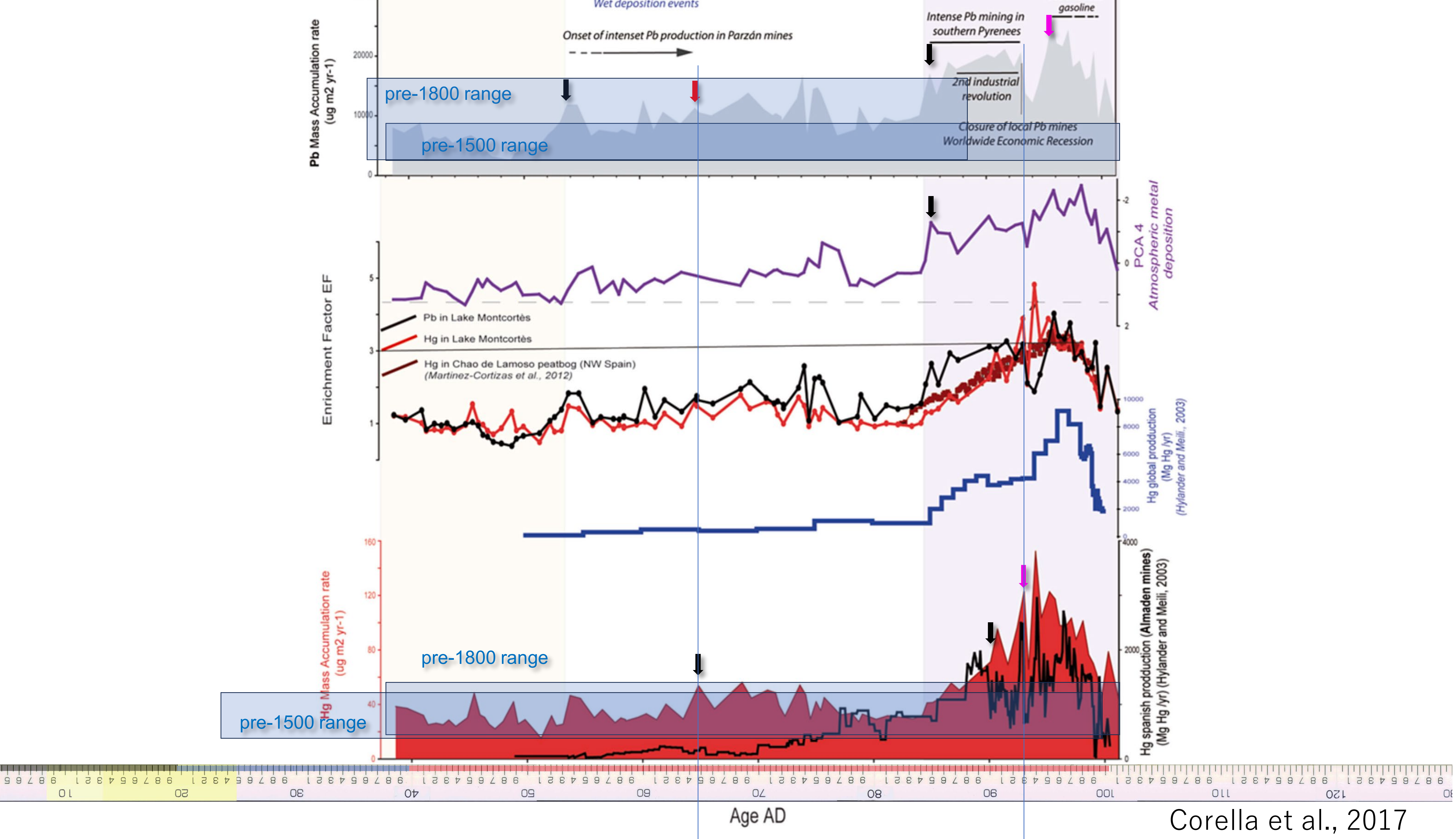

Corella et al., 2017

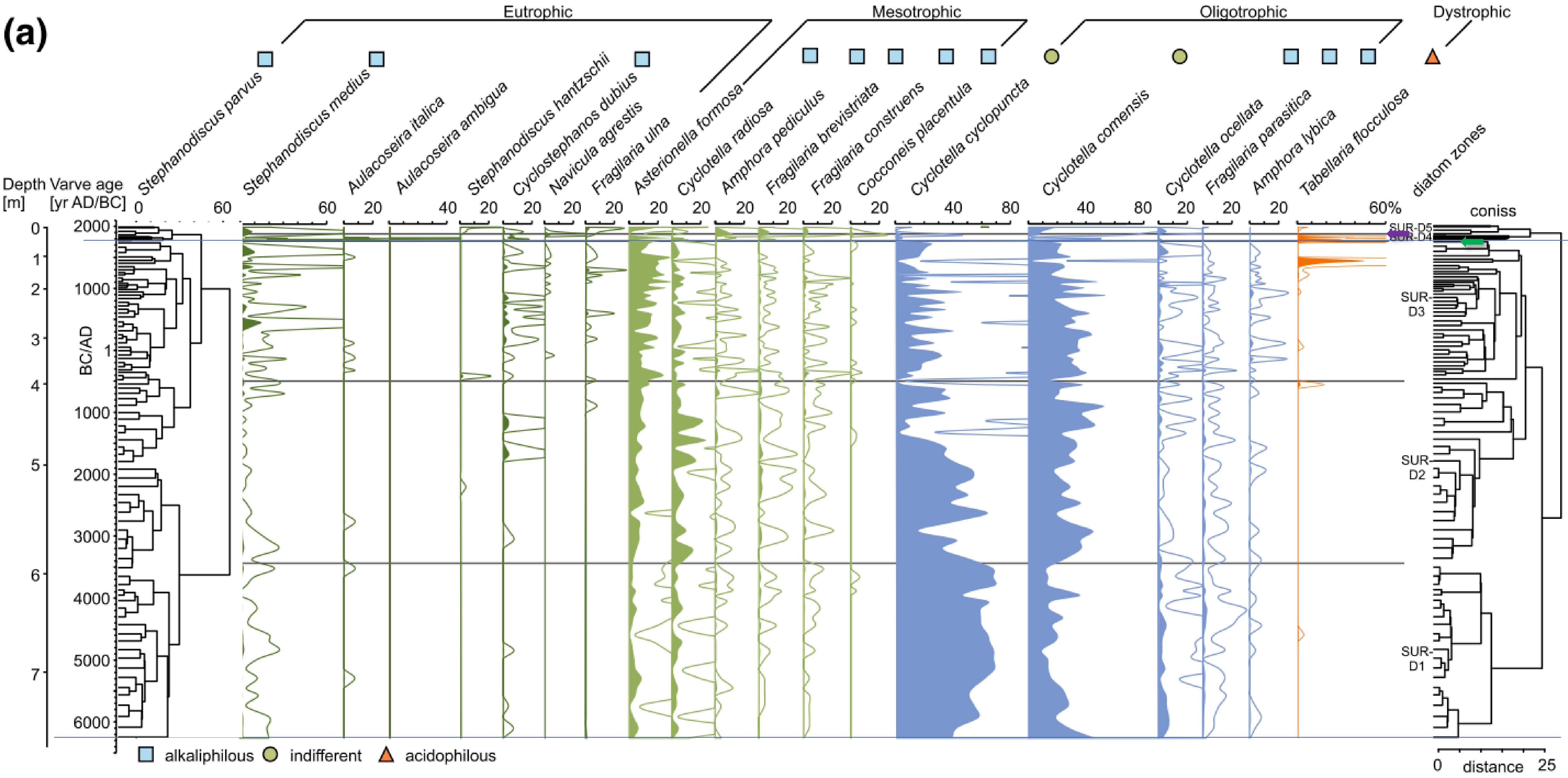

Kinder et al. (2019)

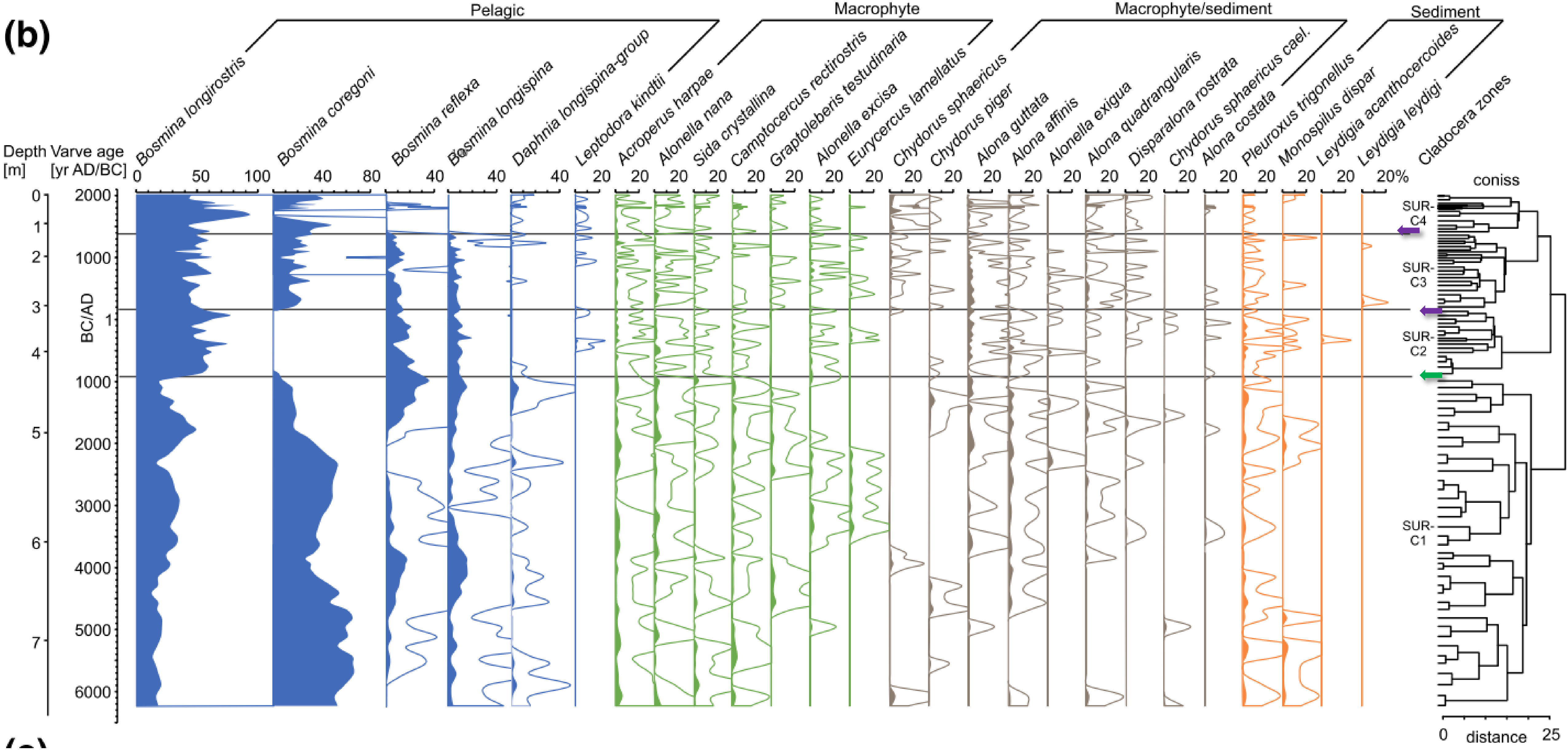

Kinder et al. (2019)

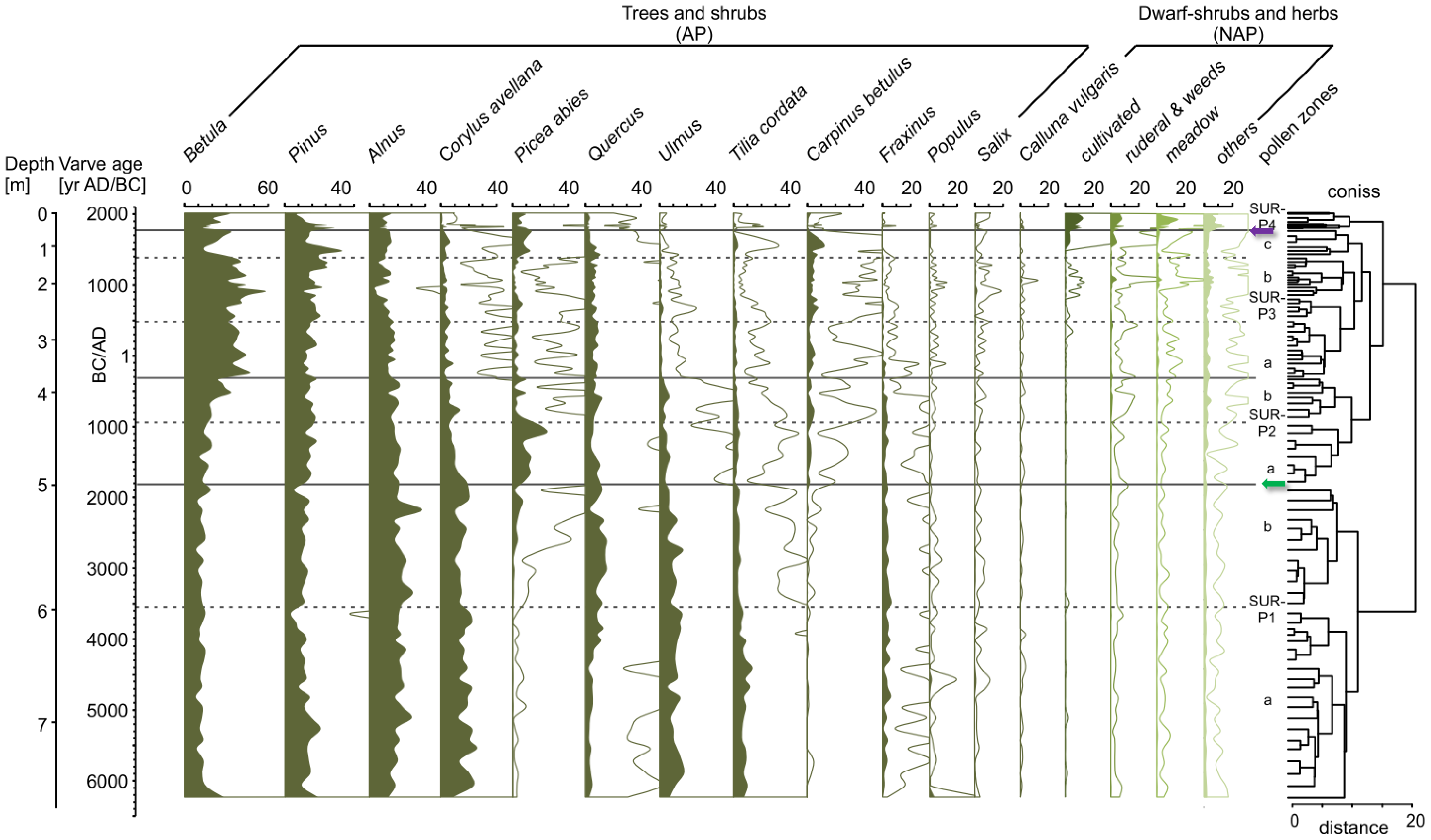

Fig. 3 Simplified pollen percentage diagram (unshaded areas show a 10 × exaggeration) and results of cluster analysis with pollen zones SUR-P1 to SUR-P4

Kinder et al. (2019)

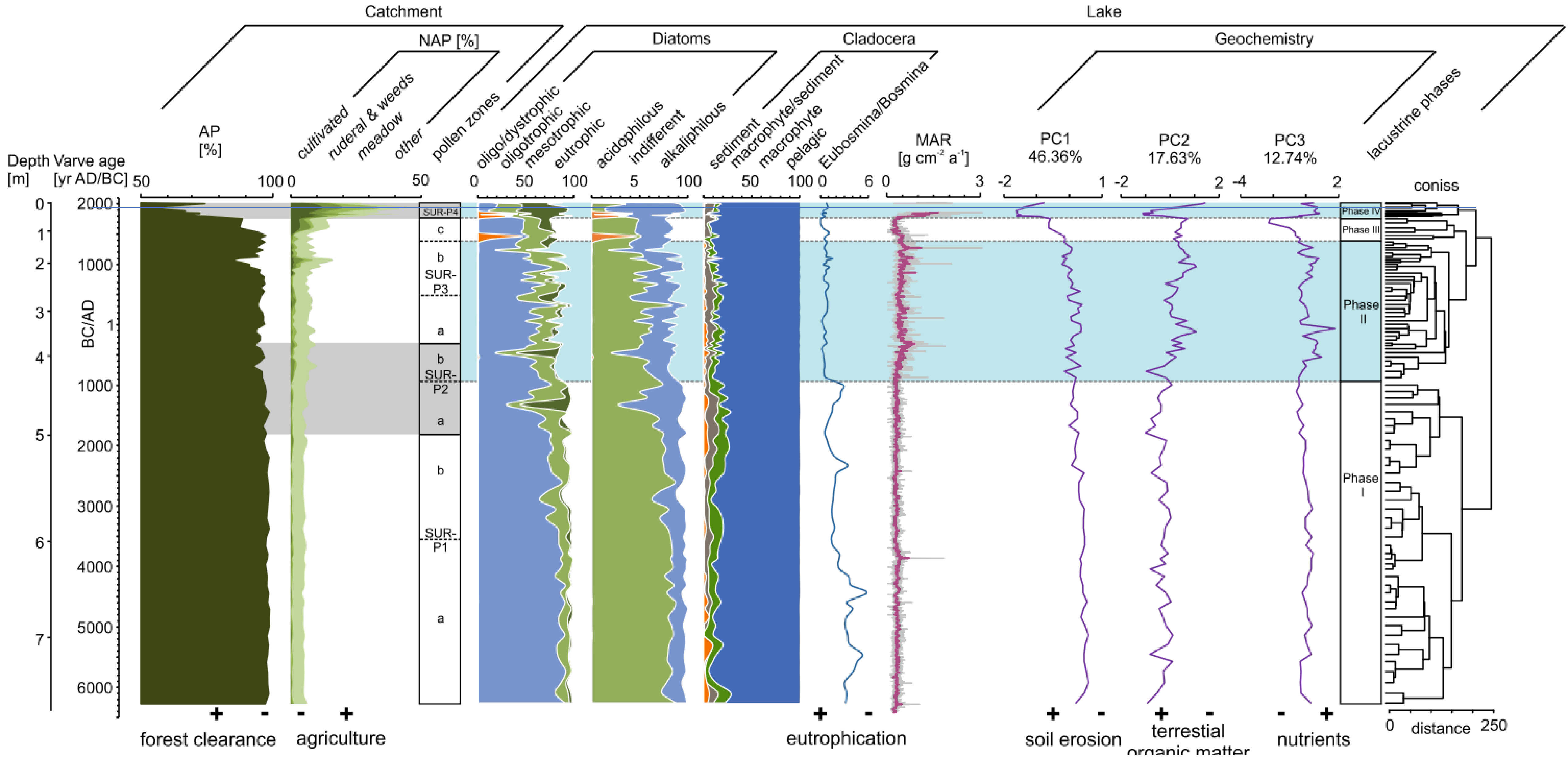

Kinder et al. (2019)

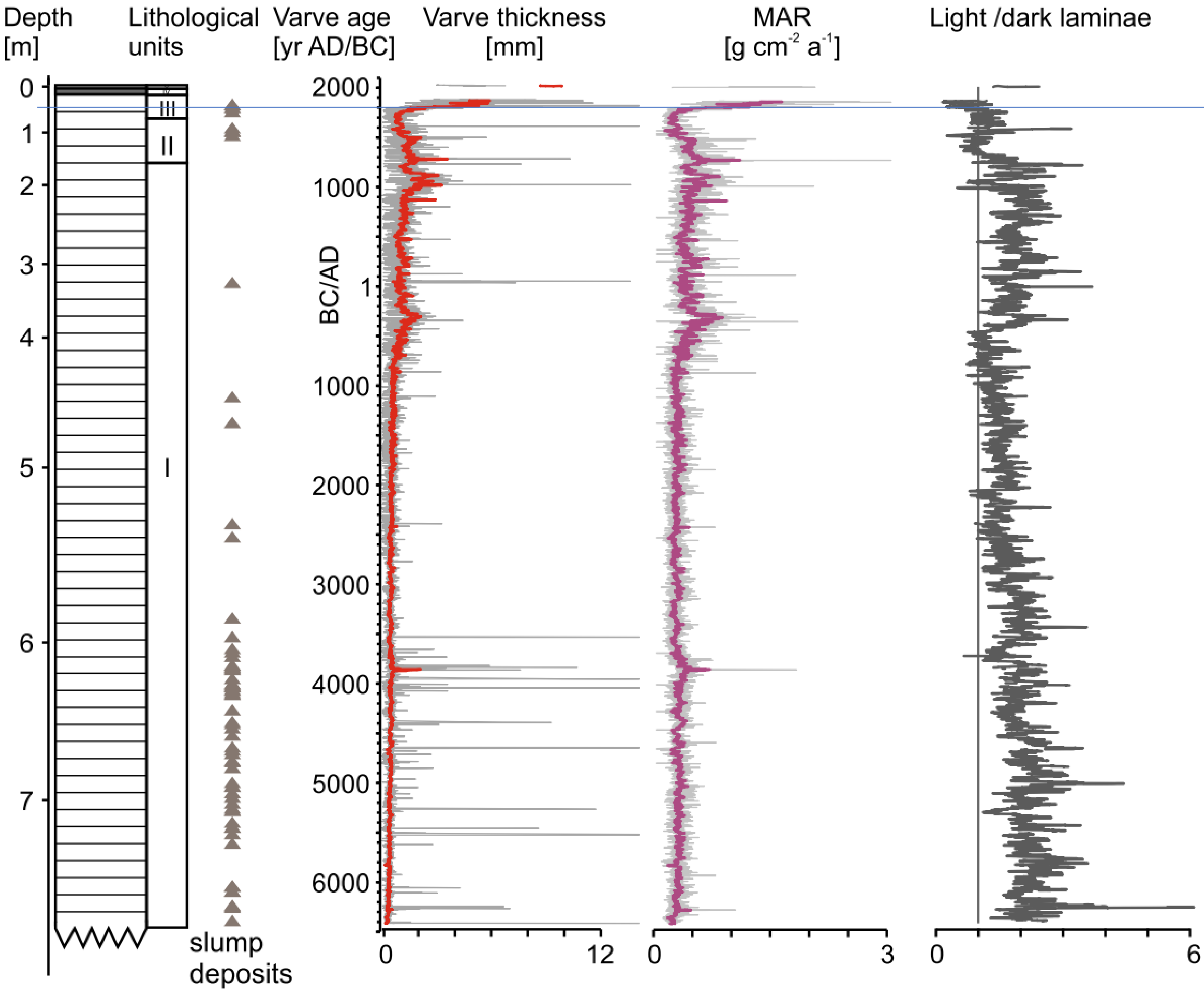

Kinder et al. (2019)

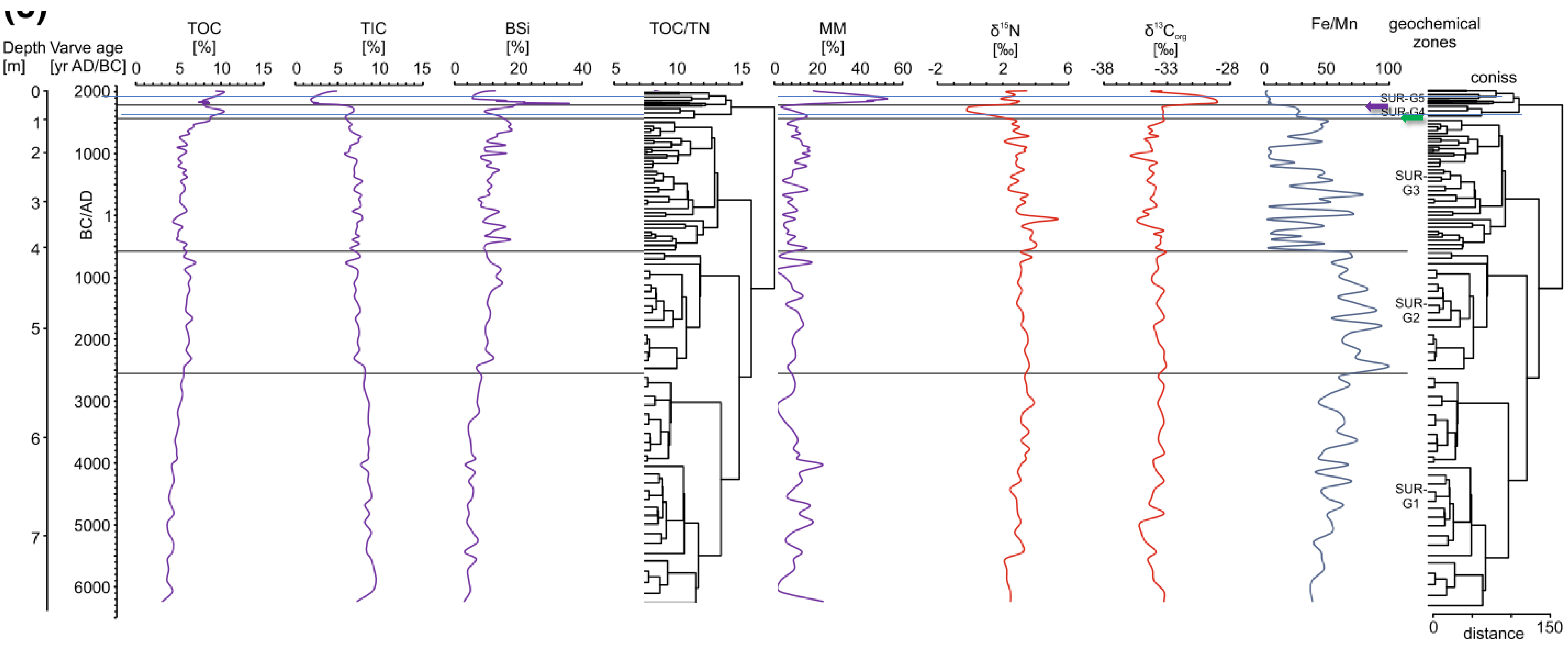

Kinder et al. (2019)

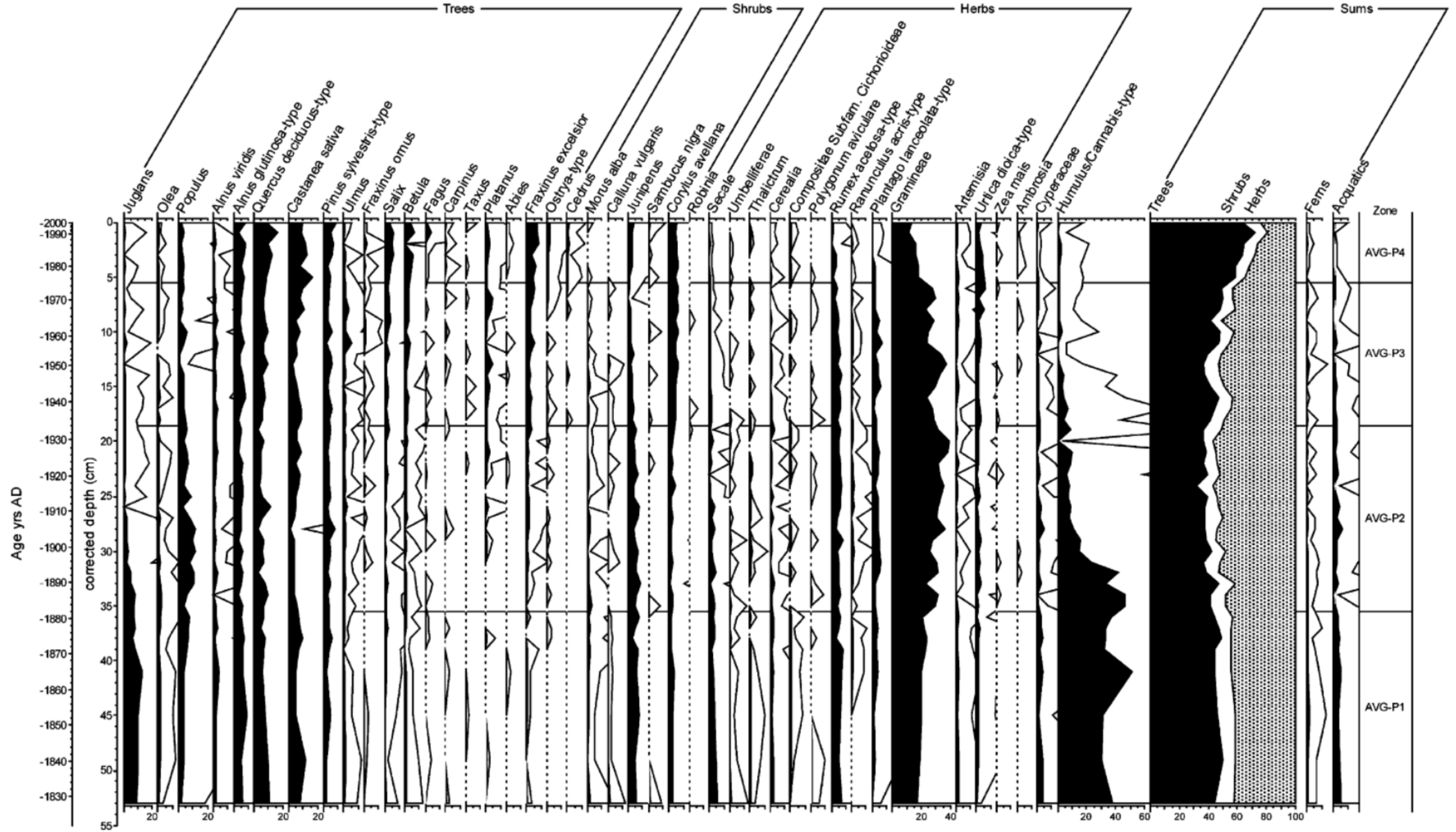

Finsinger et al. (2006)

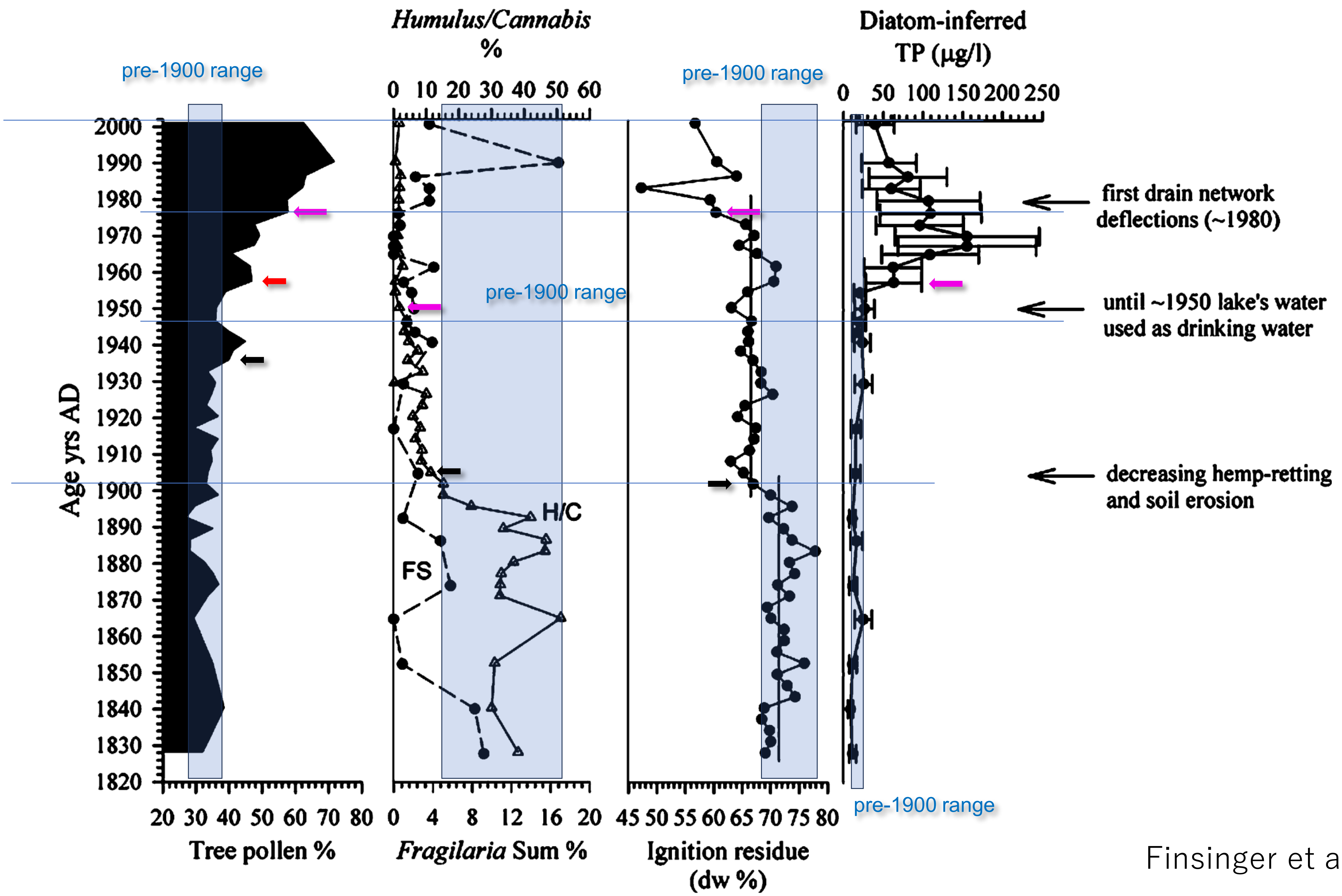

Finsinger et al. (2006)

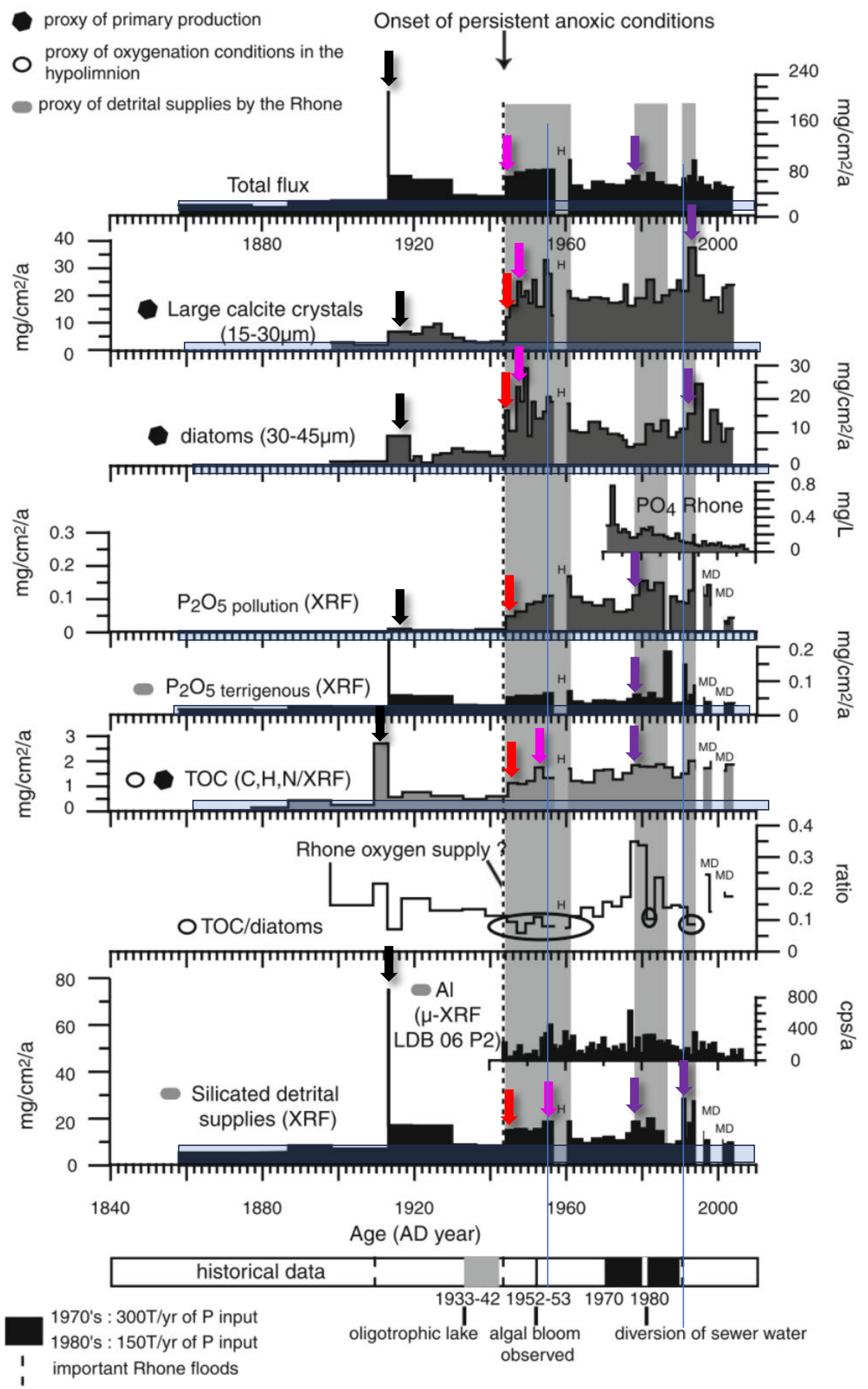

Giguet-Covex et al. (2010)

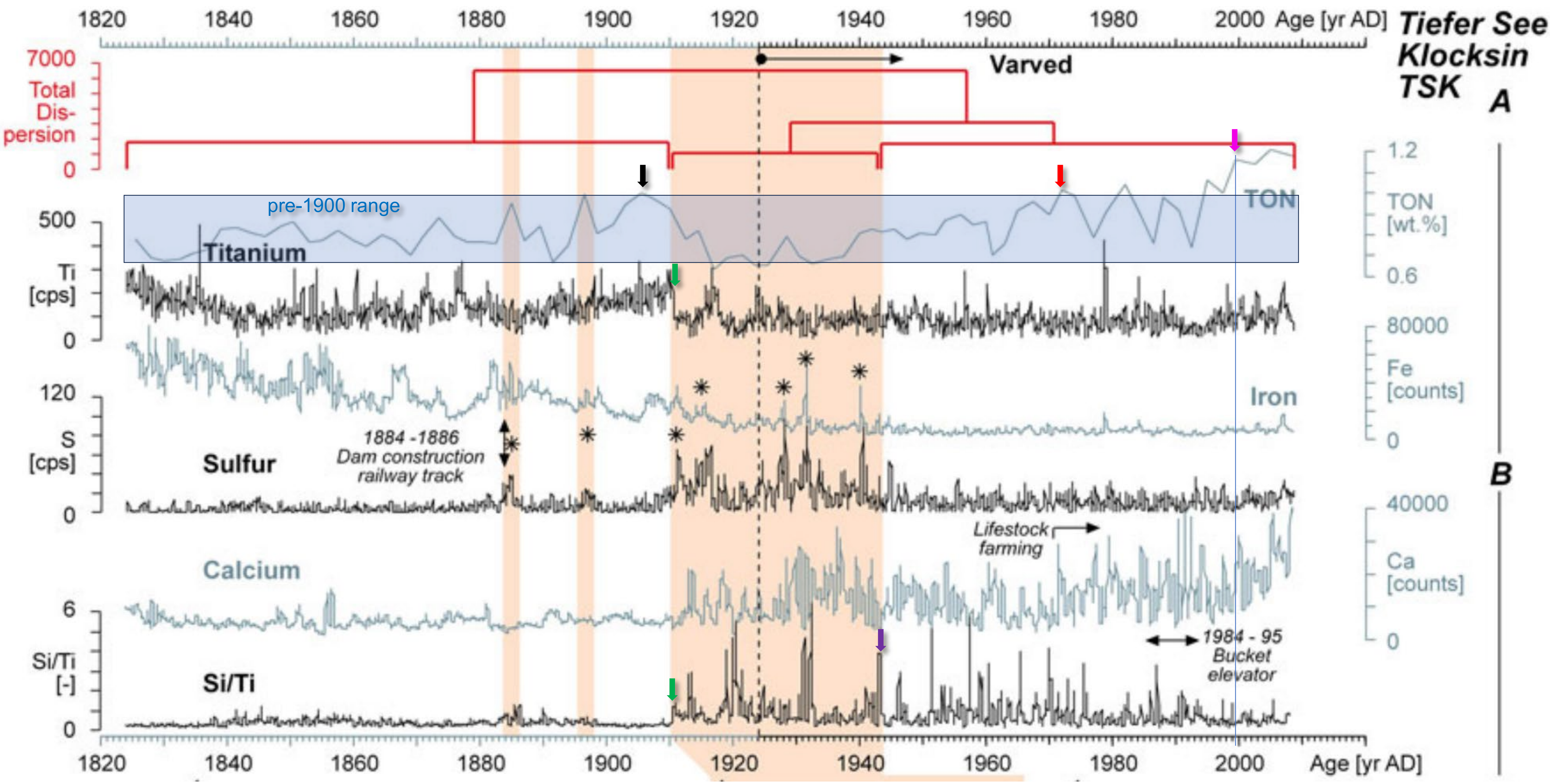

Kienel et al. (2013)

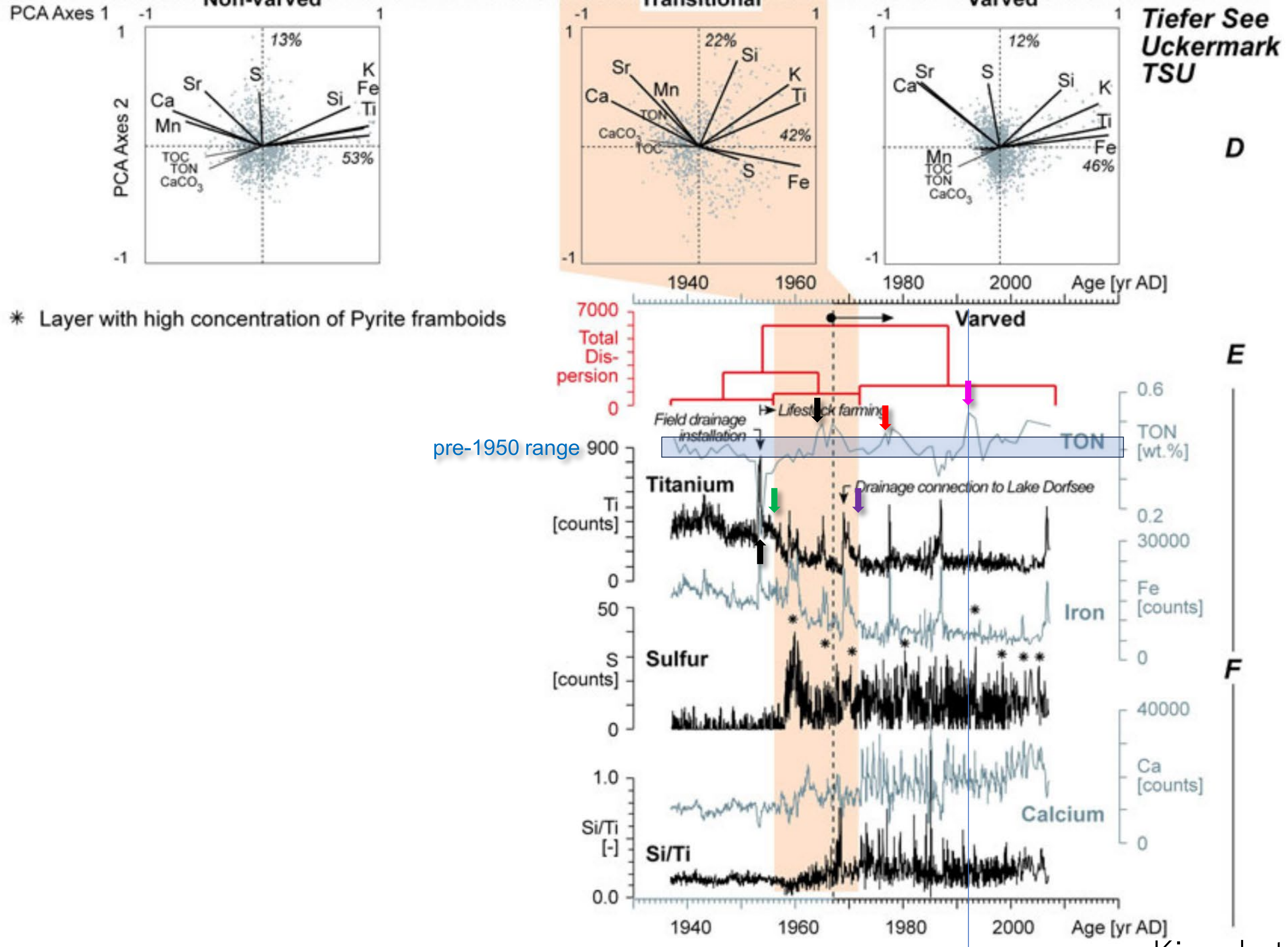

Kienel et al. (2013)

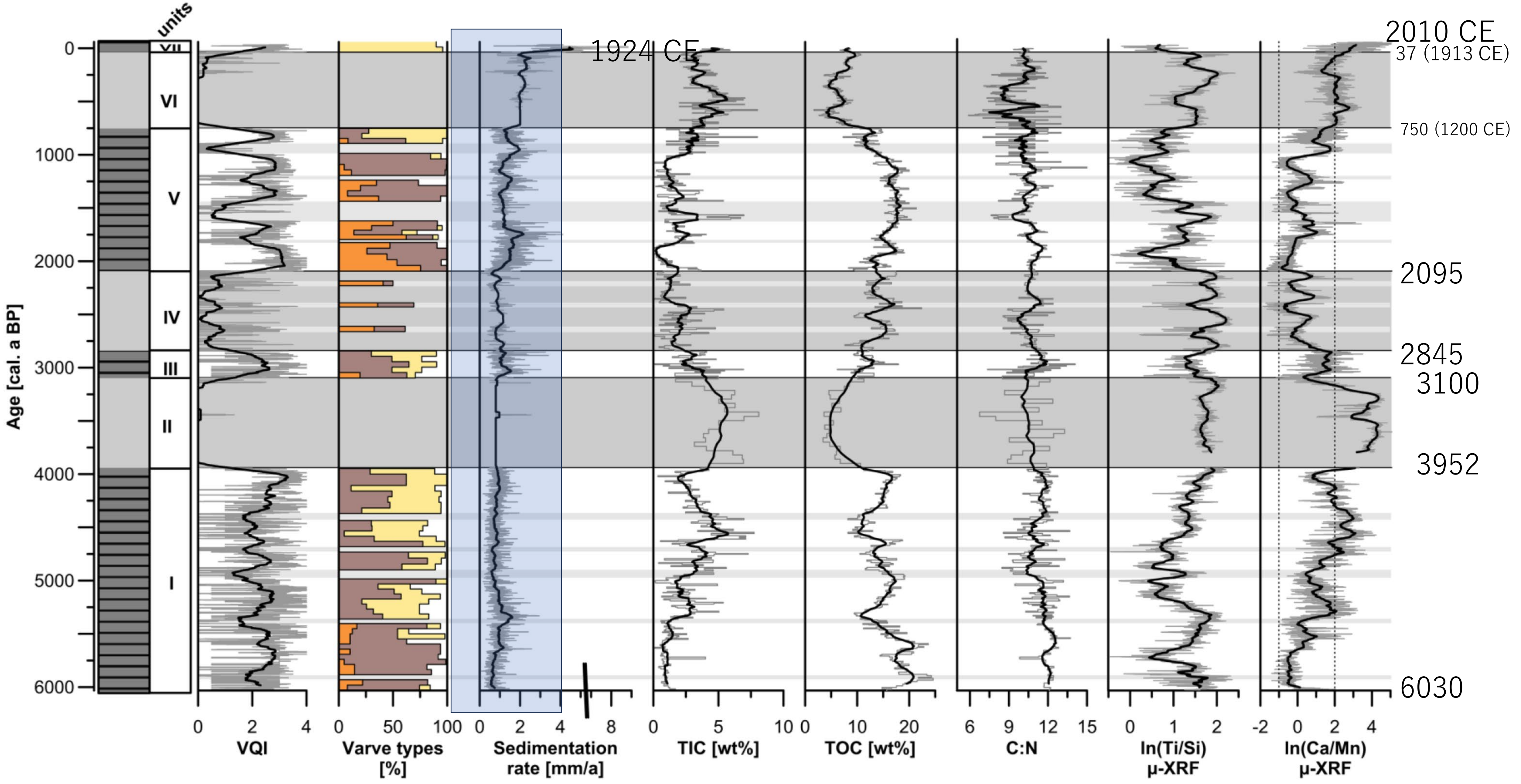

Dräger et al 2016

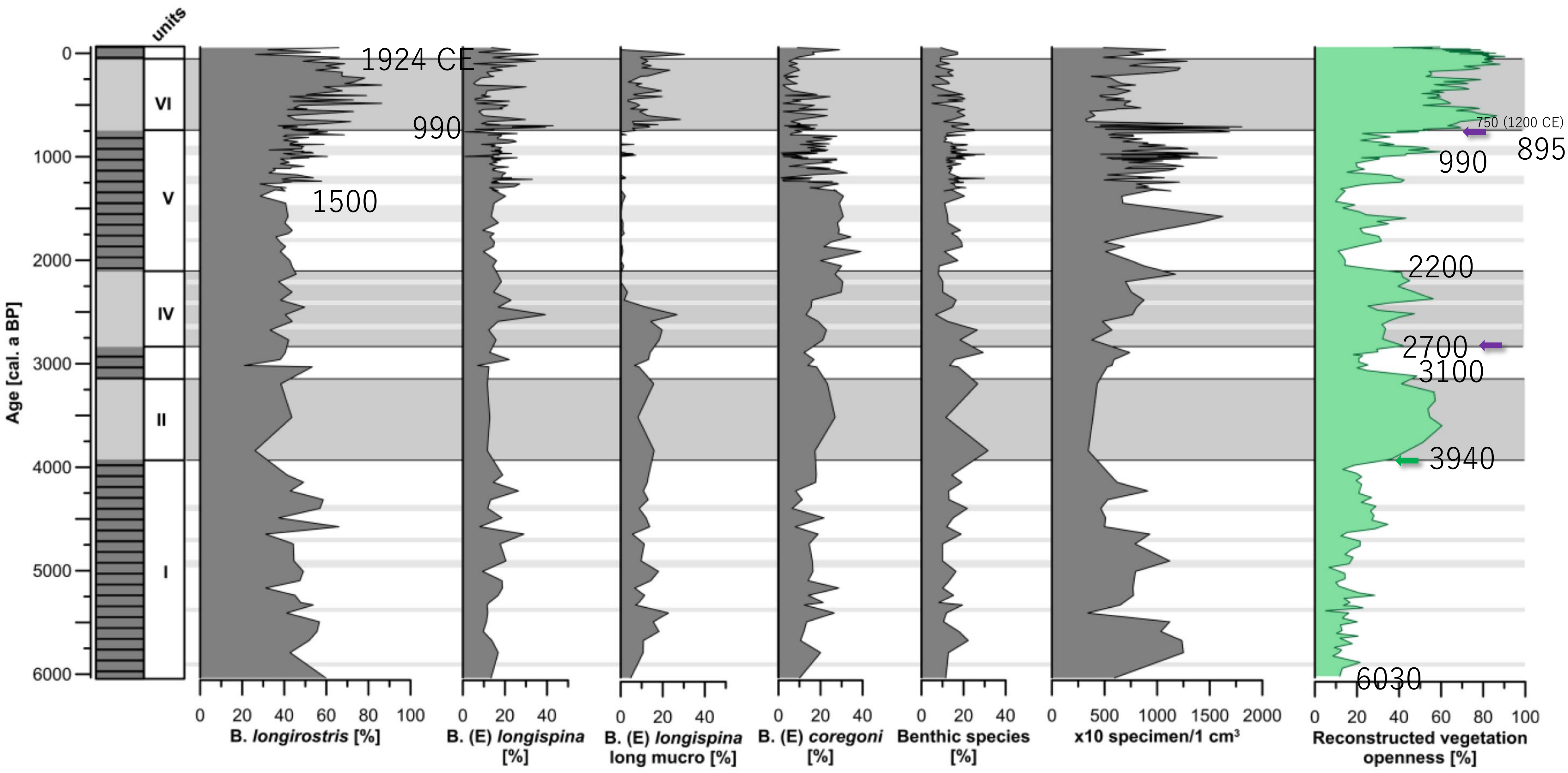

Dräger et al 2016

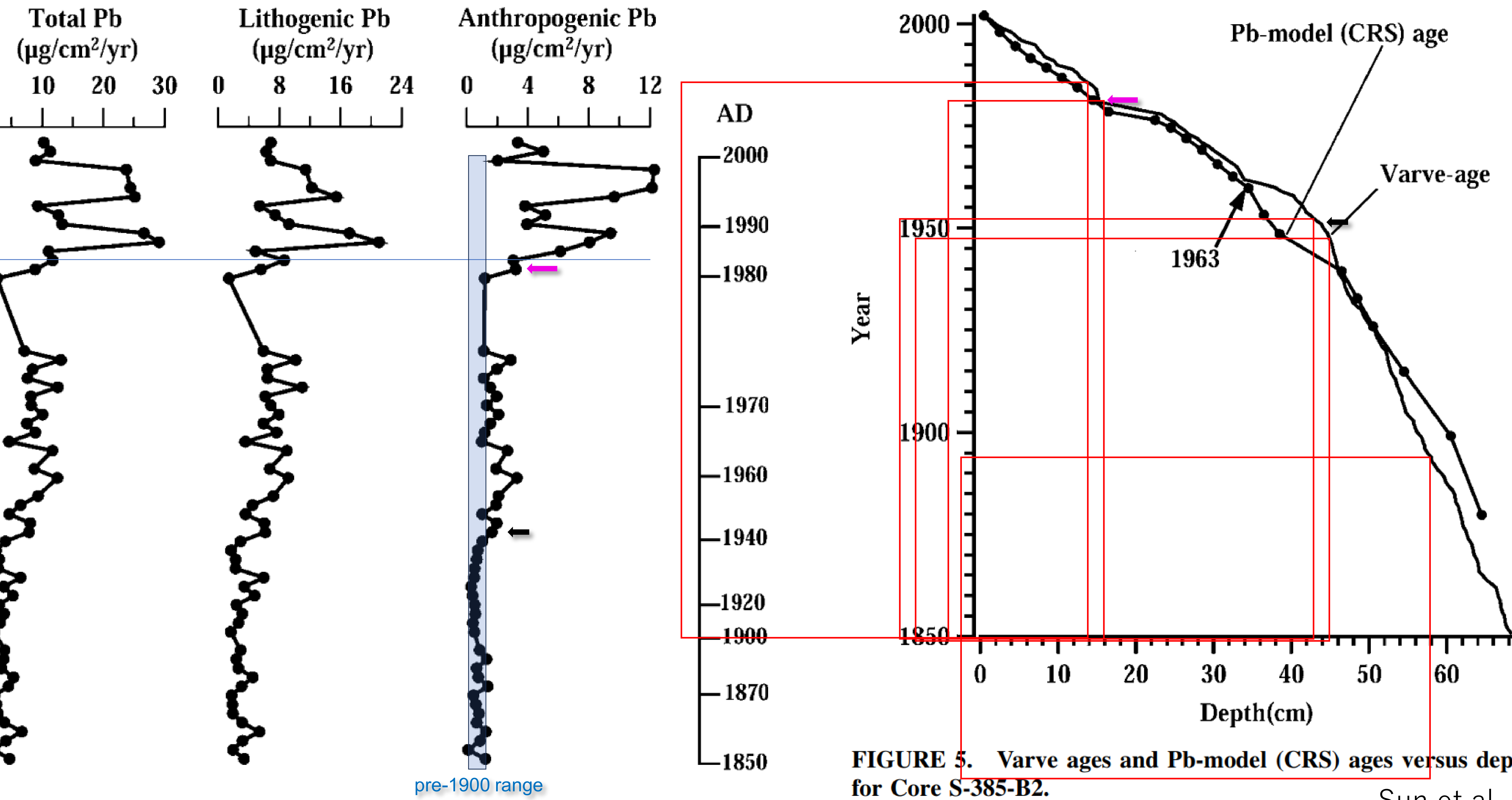

FIGURE 5. Varve ages and Pb-model (CRS) ages versus dep
for Core S-385-B2.

Sun et al.

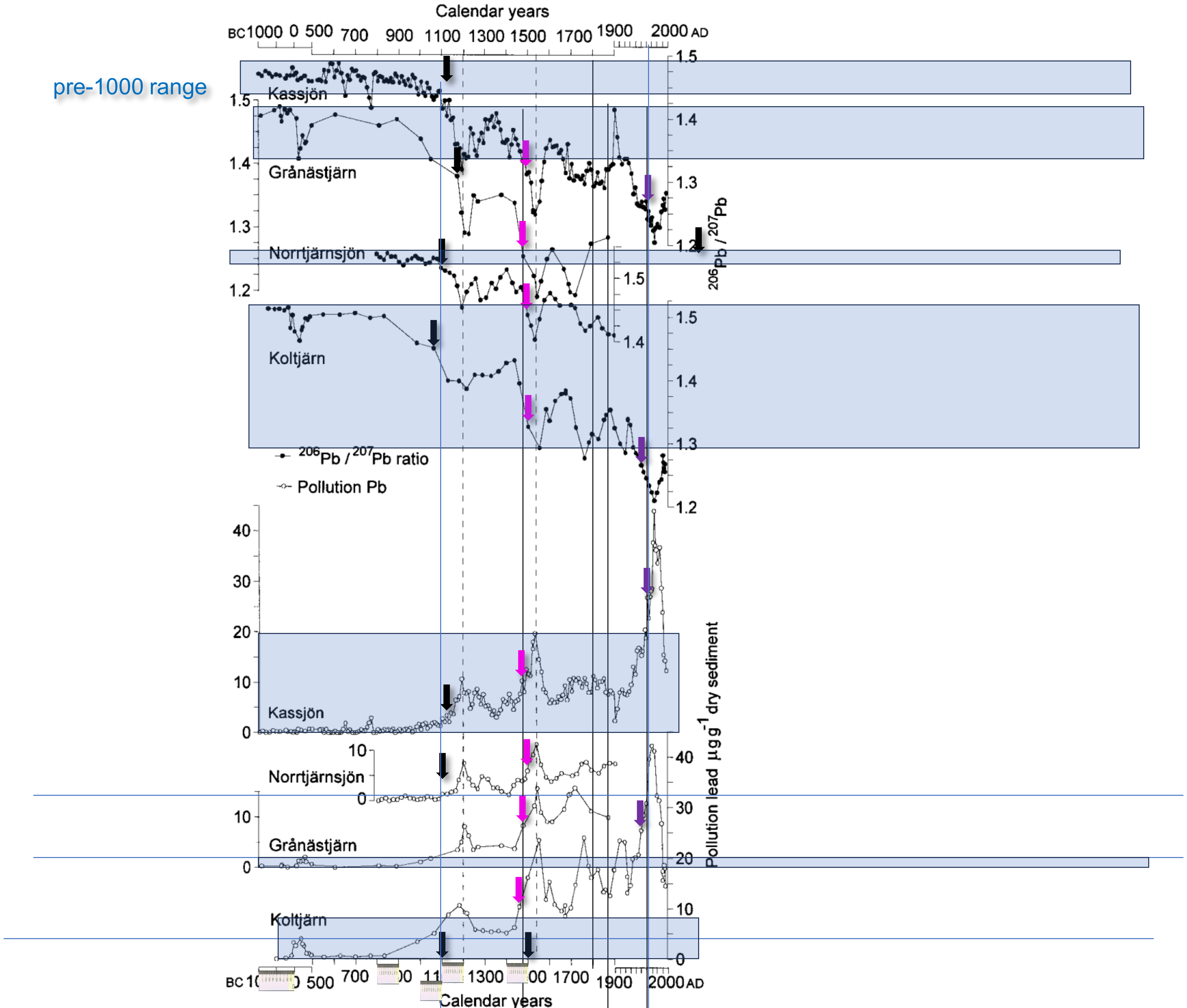

Brännvall et al. (1999)

Fig. 4 Stable nitrogen ($\partial^{15}N$) and stable carbon ($\partial^{13}C$) isotopic values along with carbon to nitrogen (C/N) ratio of the Pettaquamscutt River sediment column over the last *ca.* three centuries. Gaps in C/N plot are due to small samples that inhibited the quantification of nitrogen concentrations

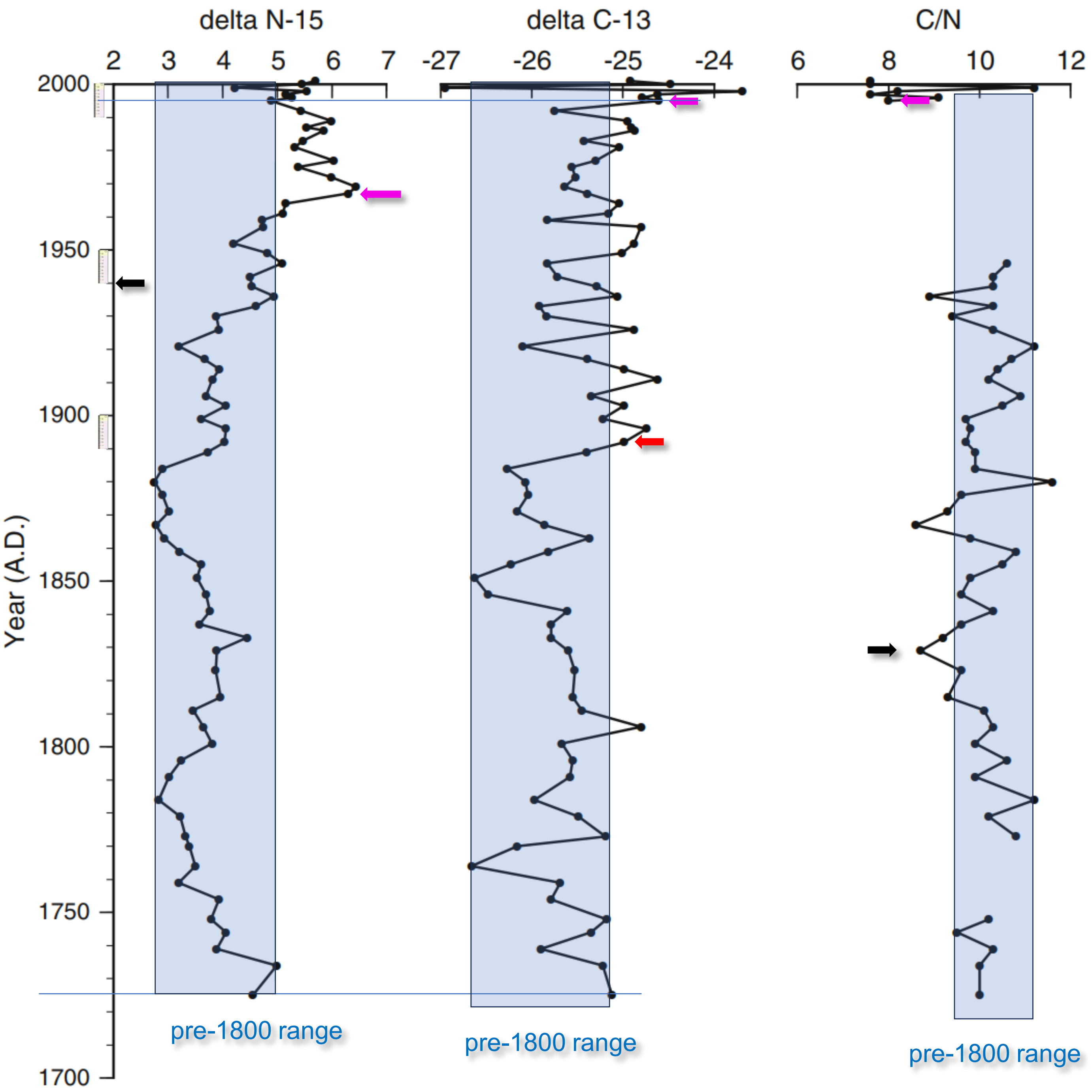

Hubeny et al. (2008)

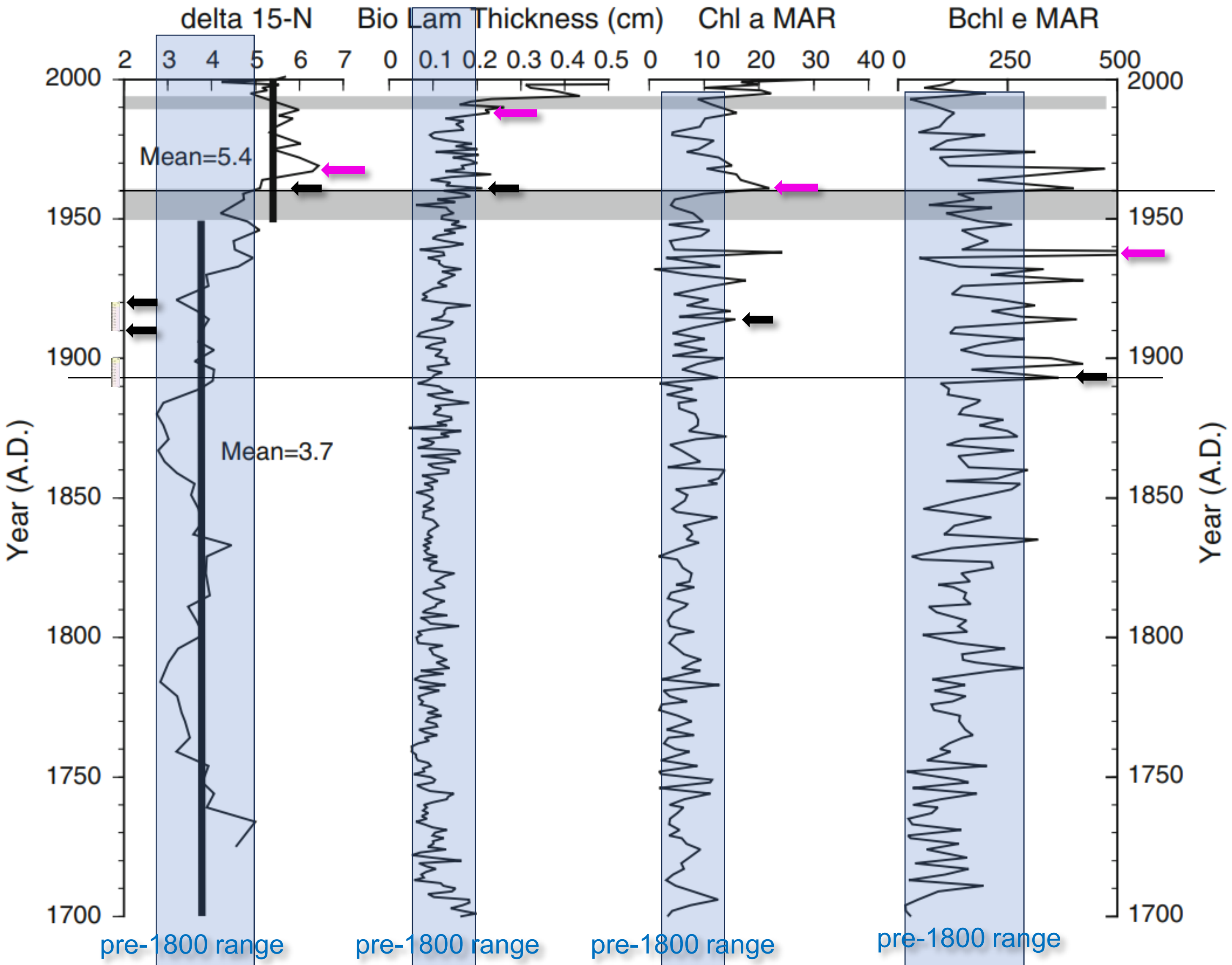

Hubeny et al. (2008)

Fig. 9 Evidence for recent cultural eutrophication in the Pettaquamscutt River. Stable nitrogen isotopic values become more enriched during the period of increased residential development in the estuary (*ca.* 1950–1960). Total productivity exhibited sustained increases during this time, as seen in biogenic laminae thicknesses and chlorophyll *a* mass accumulation rates. Green sulfur bacteria initially increase productivity, then decrease since *ca.* 1970 due to increased turbidity in the water column. Vertical lines with $\partial^{15}N$ are calculated means of the data. Thin upper gray bar represents timing of Sewer Project, while thicker lower gray bar indicates timing of increased residential development in the watershed

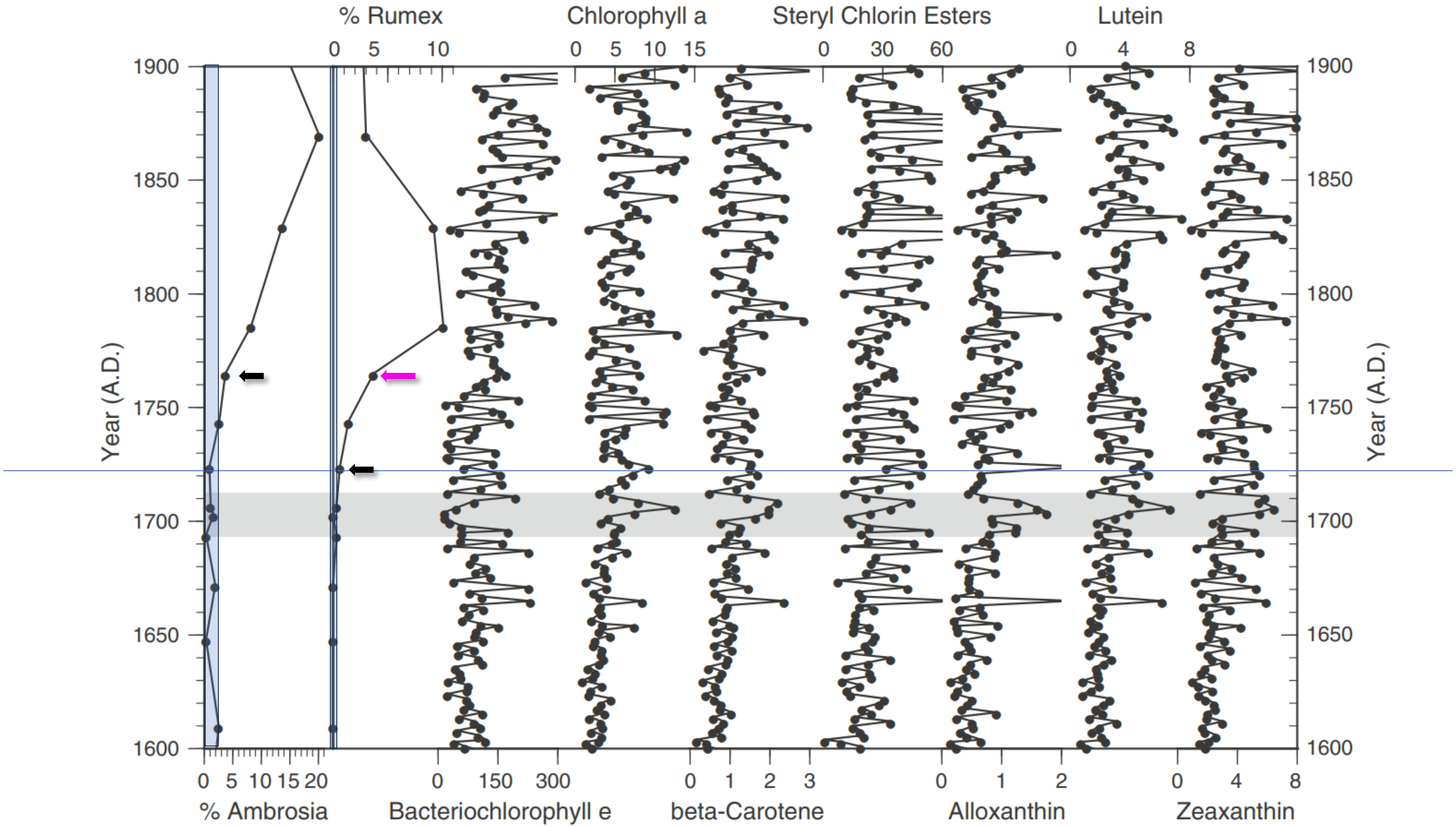

Hubeny et al. (2008)

Fig. 8 European land clearance and its effect on biotic productivity in the Pettaquamscutt River Estuary. *Ambrosia* and *Rumex* pollen percentages are plotted for comparison to land use change. All fossil pigment values are mass accumulation rates (μg/cm^2 yr). Gray bar represents approximate period of anthropogenic influence on autotrophs

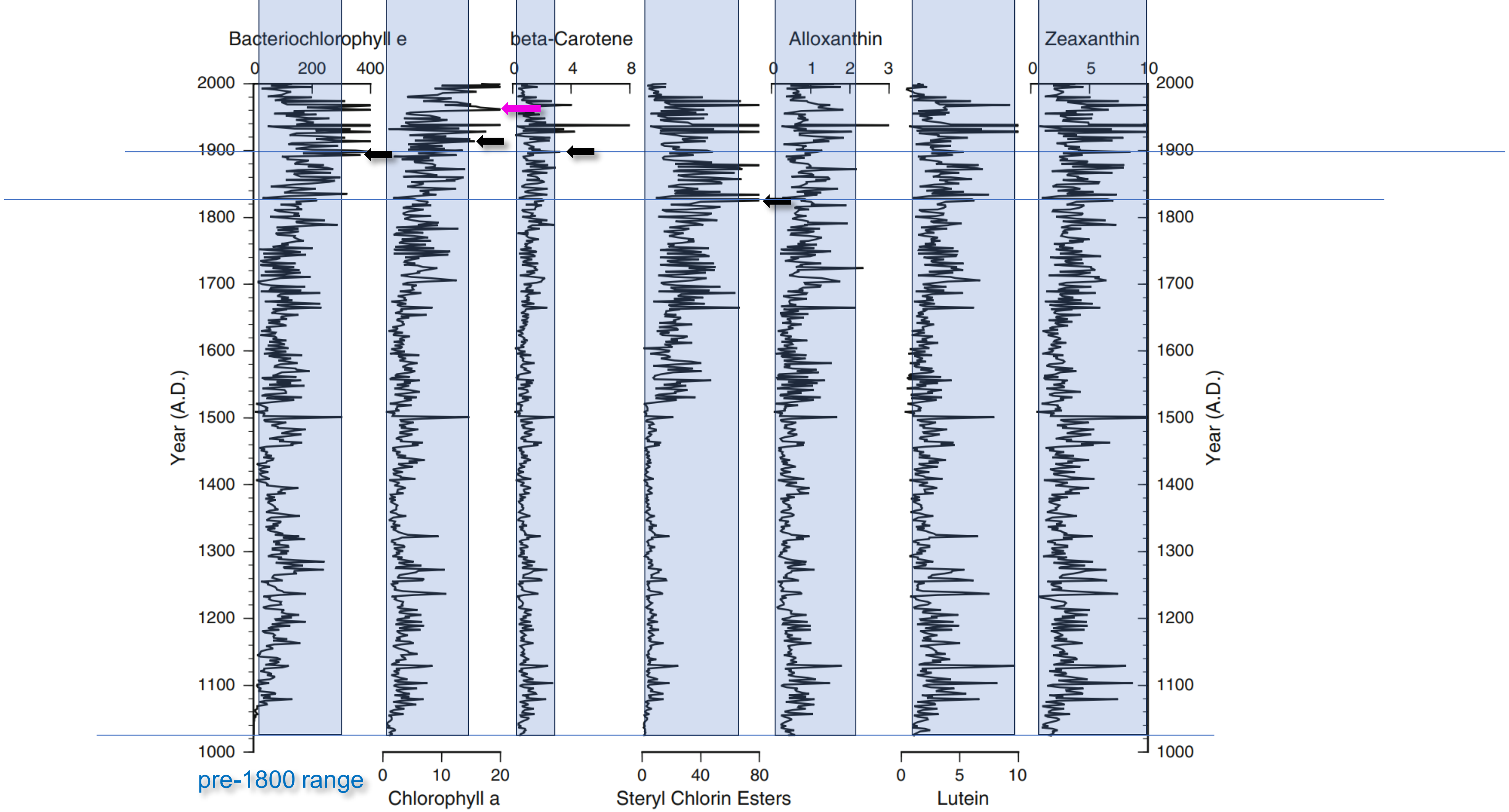

Fig. 5 Fossil pigment mass accumulation rate (μg/cm^2 yr) time series. Pigment affinities given in Table 2

are linked to land-use changes during Colonial times (Fig. 6). Prior to the pollen sample at 1694 A.D., small percentages of *Ambrosia* were observed, but *Rumex* (not native to North America) was nonexistent. Since *Ambrosia* prefers clear fields to forests, these percentages suggest agriculture was present during this time, however the absence of *Rumex* indicates that this period was prior to European settlement. This land-use scenario fits the historical data that the Narragansett Tribe was active in agriculture (Narragansett Indian Tribe 2007). The first occurrence of *Rumex* is observed at 1694 A.D., indicating presence of Europeans in the region at this time. The end of the seventeenth century corresponds

Hubeny et al. (2008)

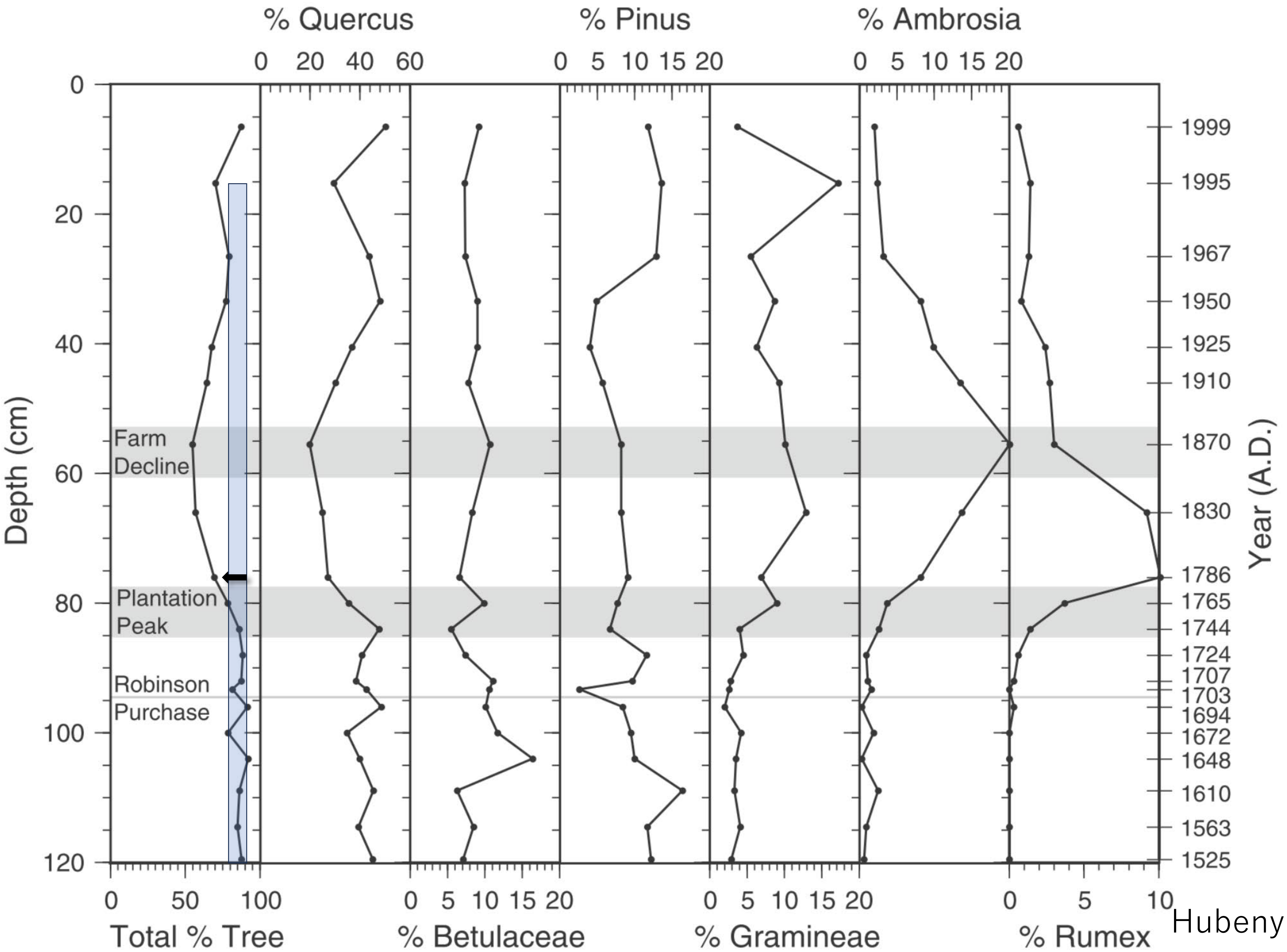

Hubeny et al. (2008)

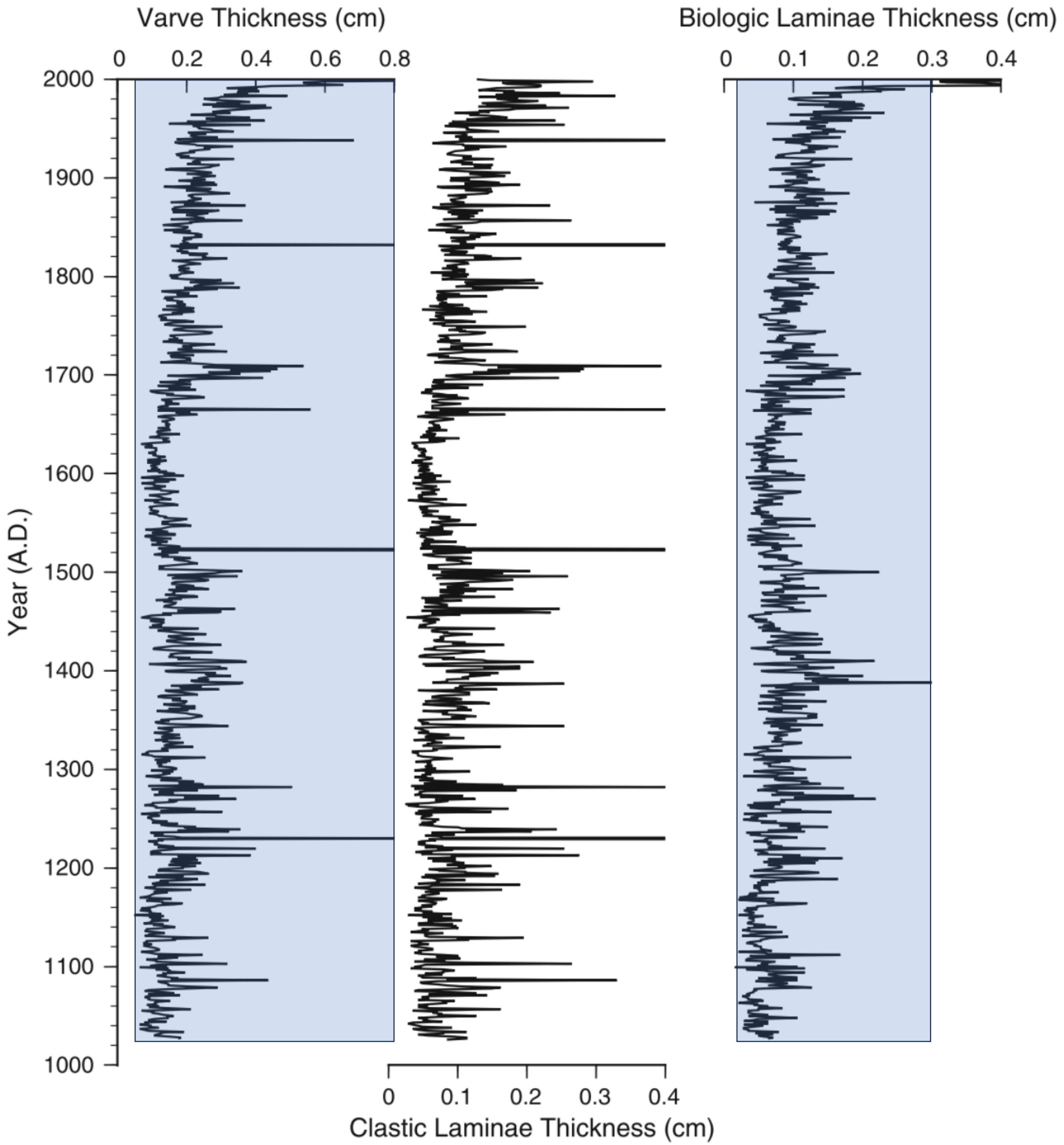

Hubeny et al. (2008)

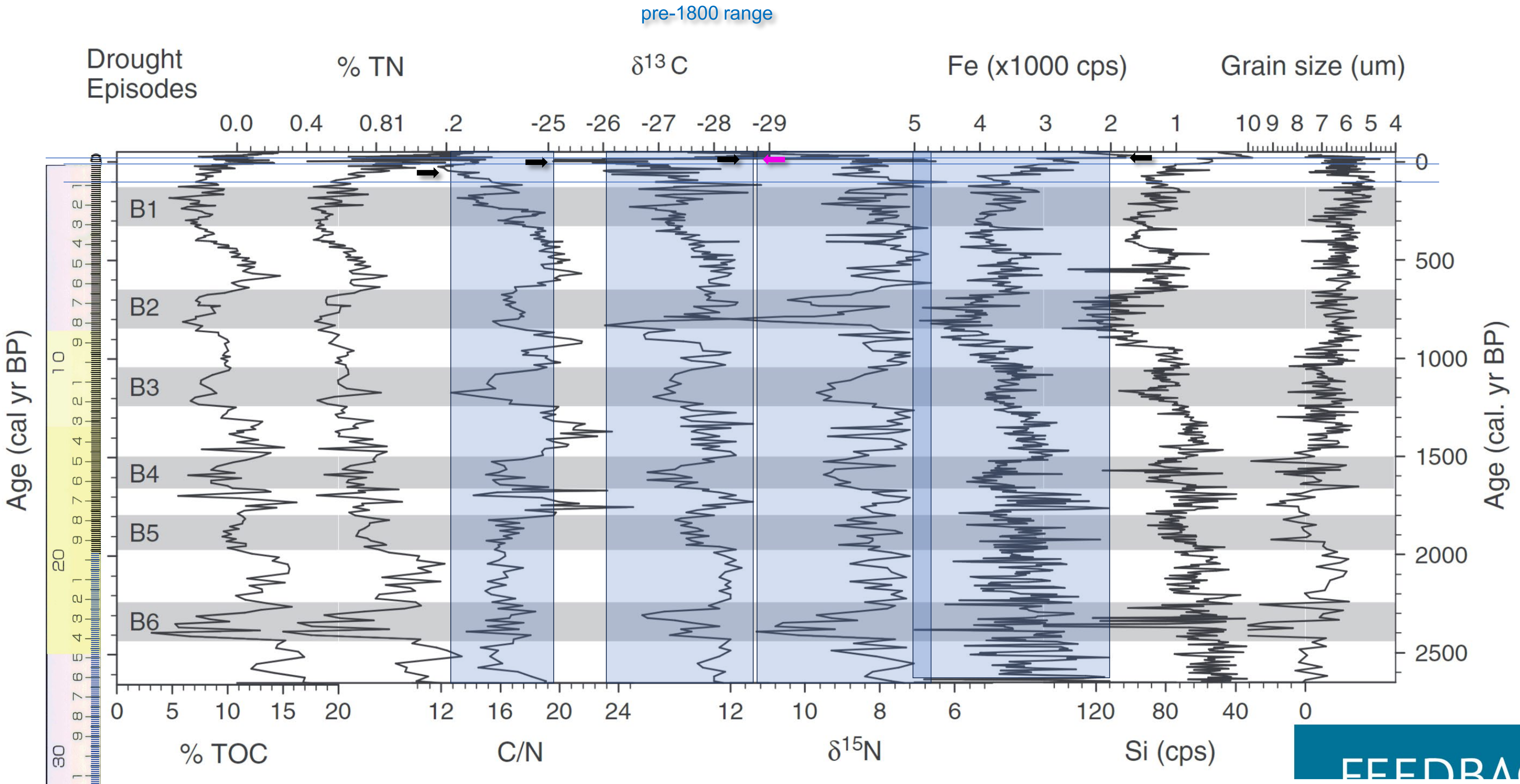

Shanahan et al., 2013

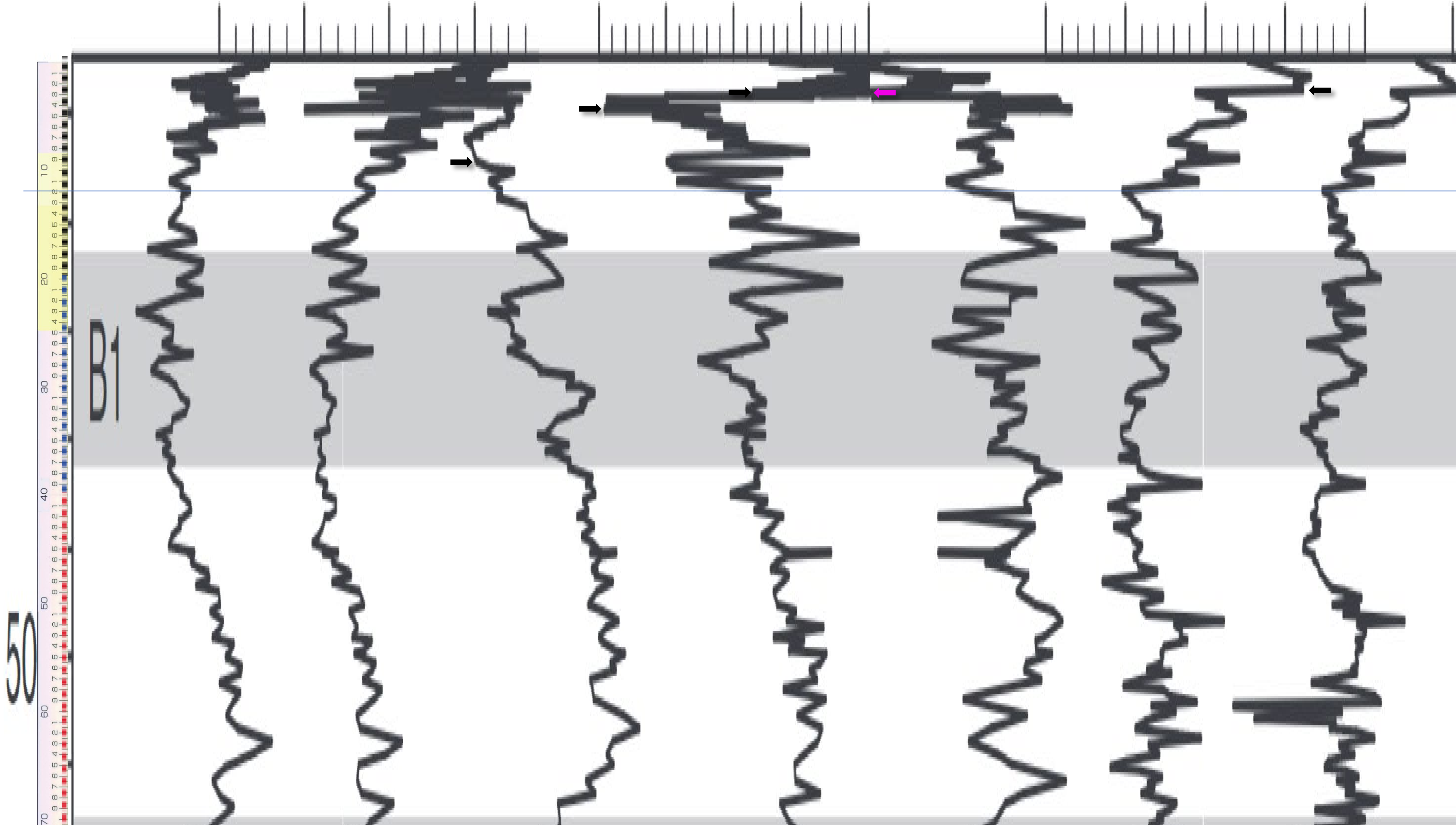
B1
50

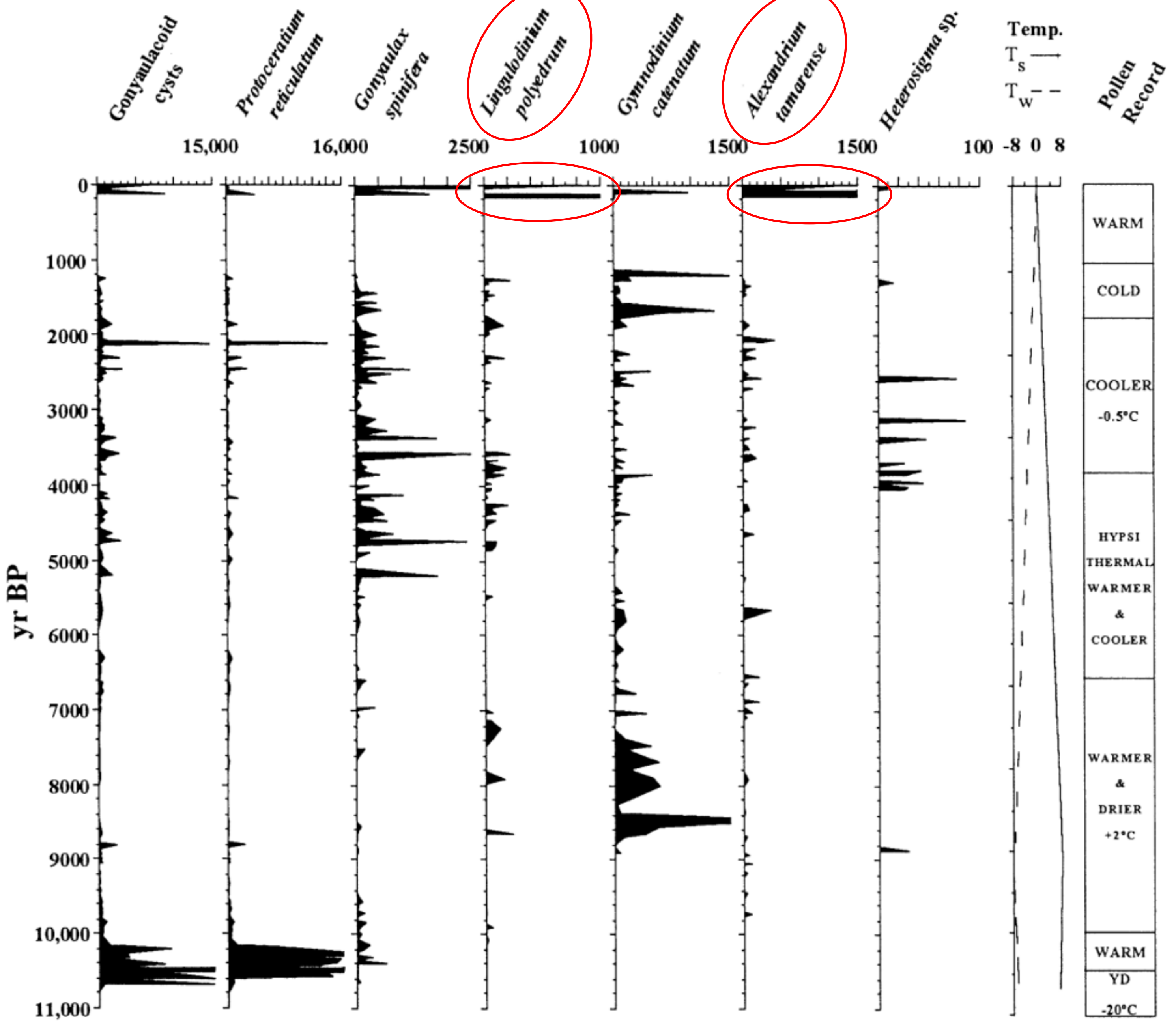

Fig. 10. Geological record from Saanich Inlet at ODP Site 169S, Site 1034B, showing variations in cyst influx (cysts/cm^2/yr) for total gonyaulacoids and for cyst-forming red tide species compared to changes in climate according to the pollen record of Pellatt et al. (2001) and % deviations in orbital insolation for summer (S) and winter (W).

Mudie et al. (2002)

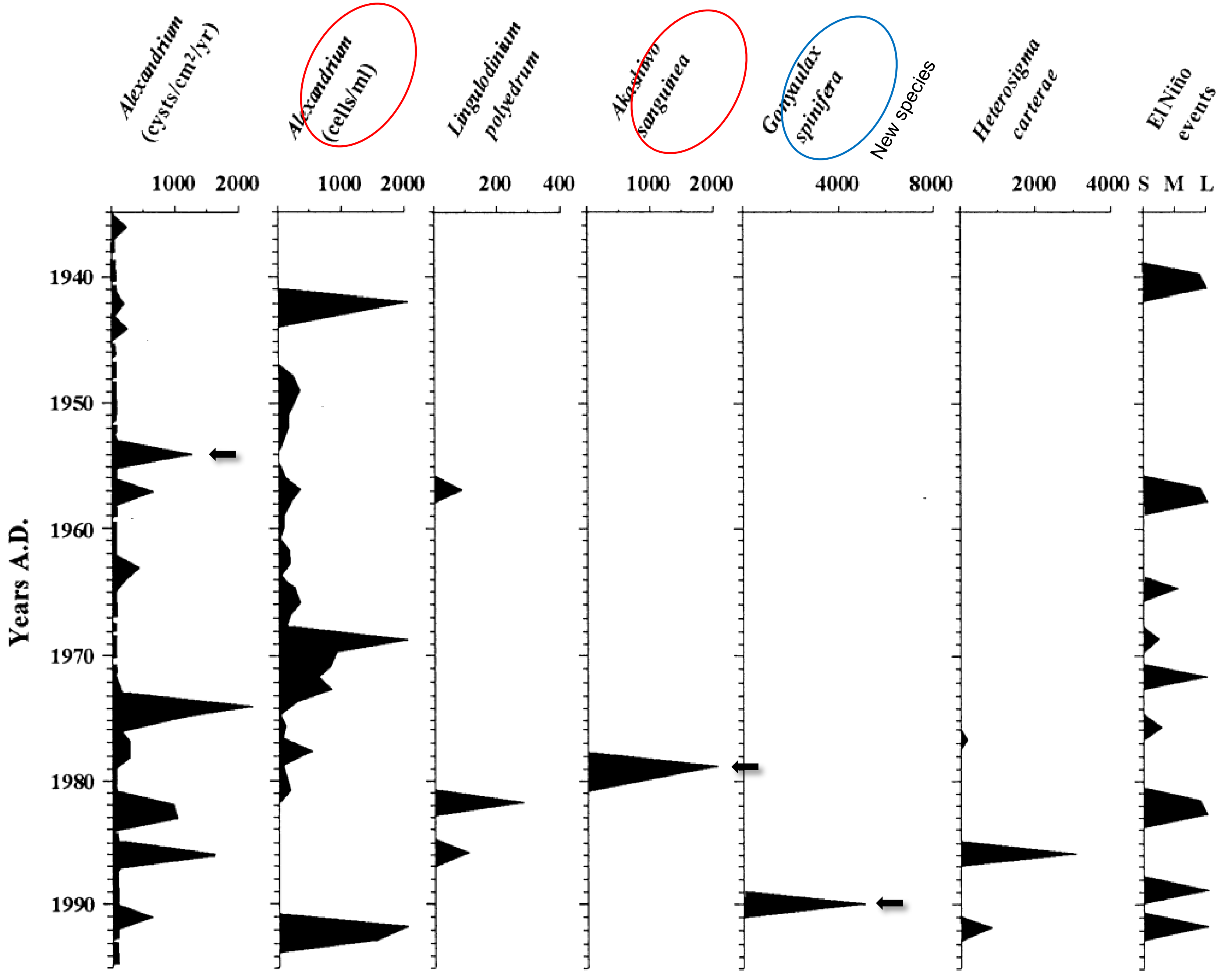

Fig. 7. Saanich Core 11: graphs of annual red tide cyst deposition (cysts/cm^2/yr) from 1935 to 1993, and concentrations of *Alexandrium catenella* and *Akashiwo sanguineum* motile cells (cells/ml) reported for the study area from 1942 to 1993.

Mudie et al. (2002)

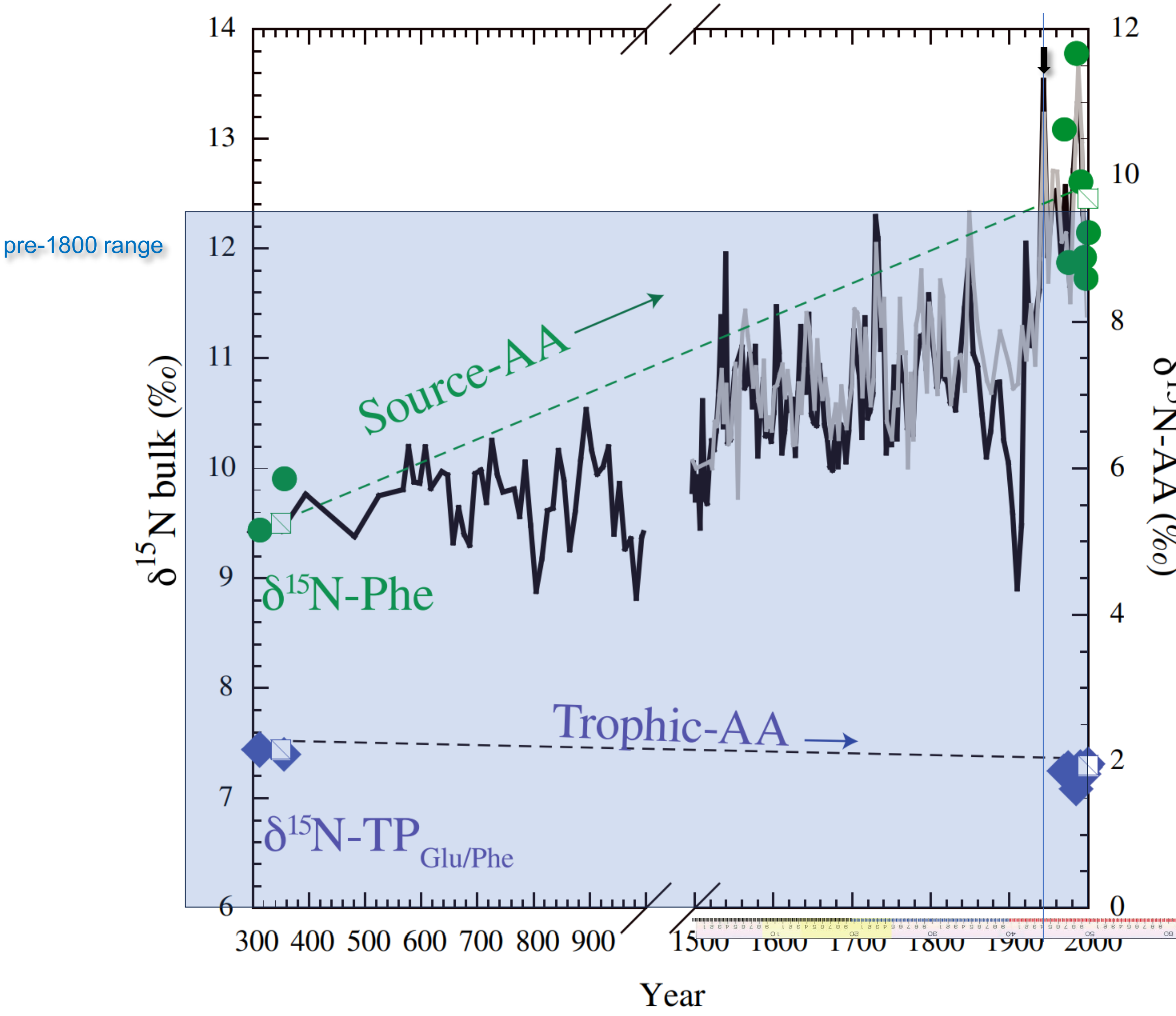

Prouty et al., 2014

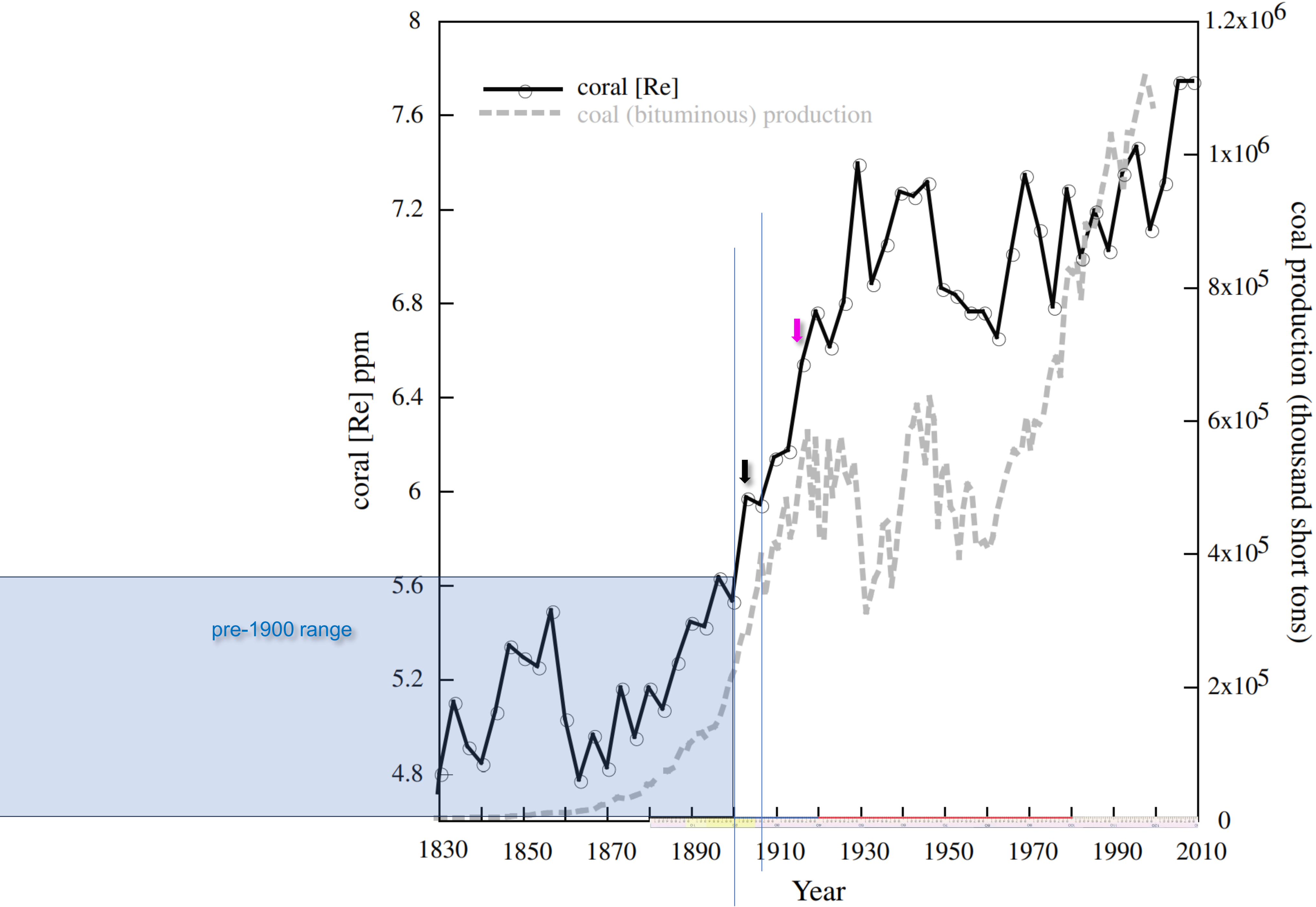

Prouty et al., 2014

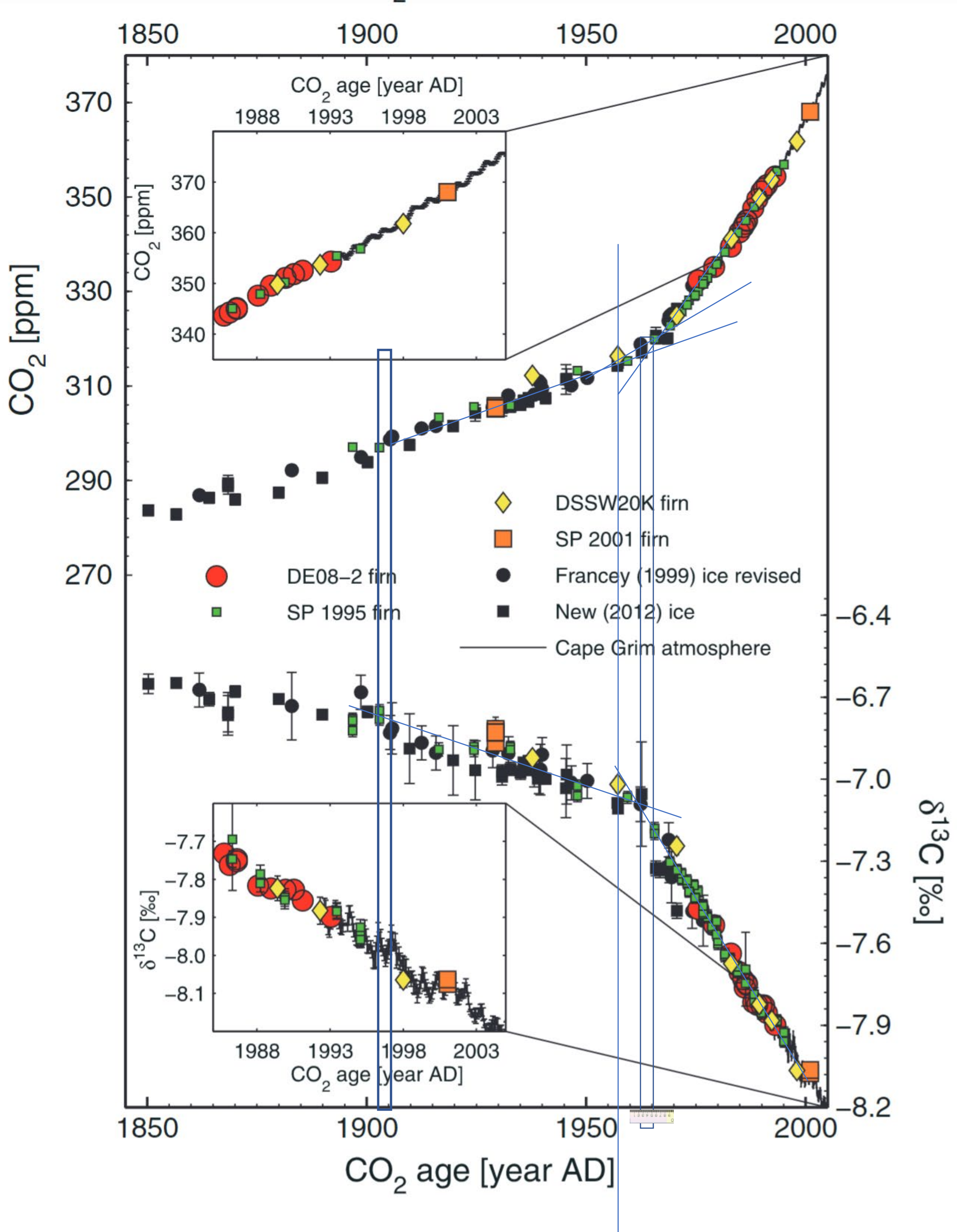

Rubino et al.,2013

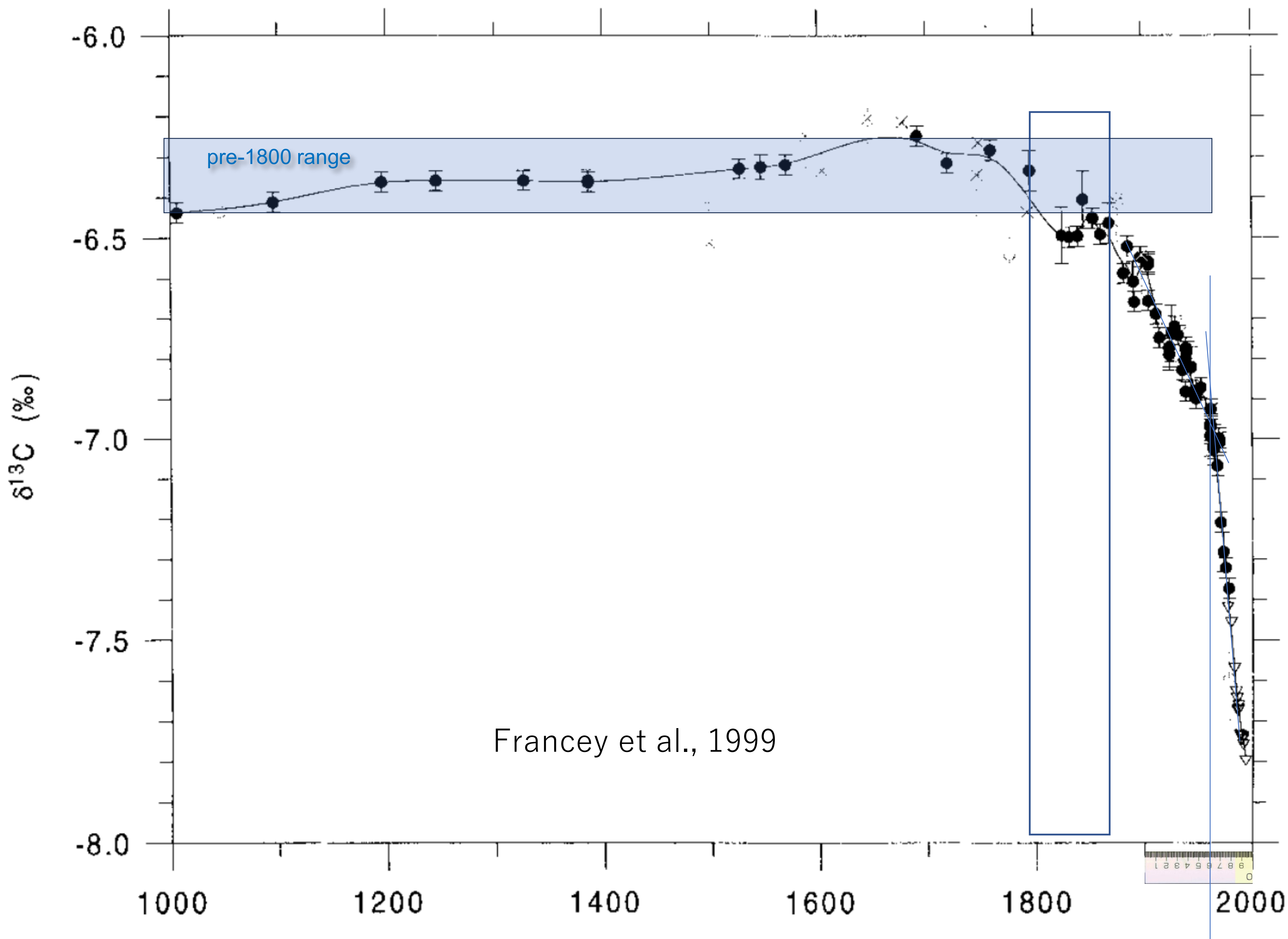

-6.0
-6.5
-7.0
-7.5
-8.0
δ13C (‰)
pre-1800 range
Francey et al., 1999
1000
1200
1400
1600
1800
2000

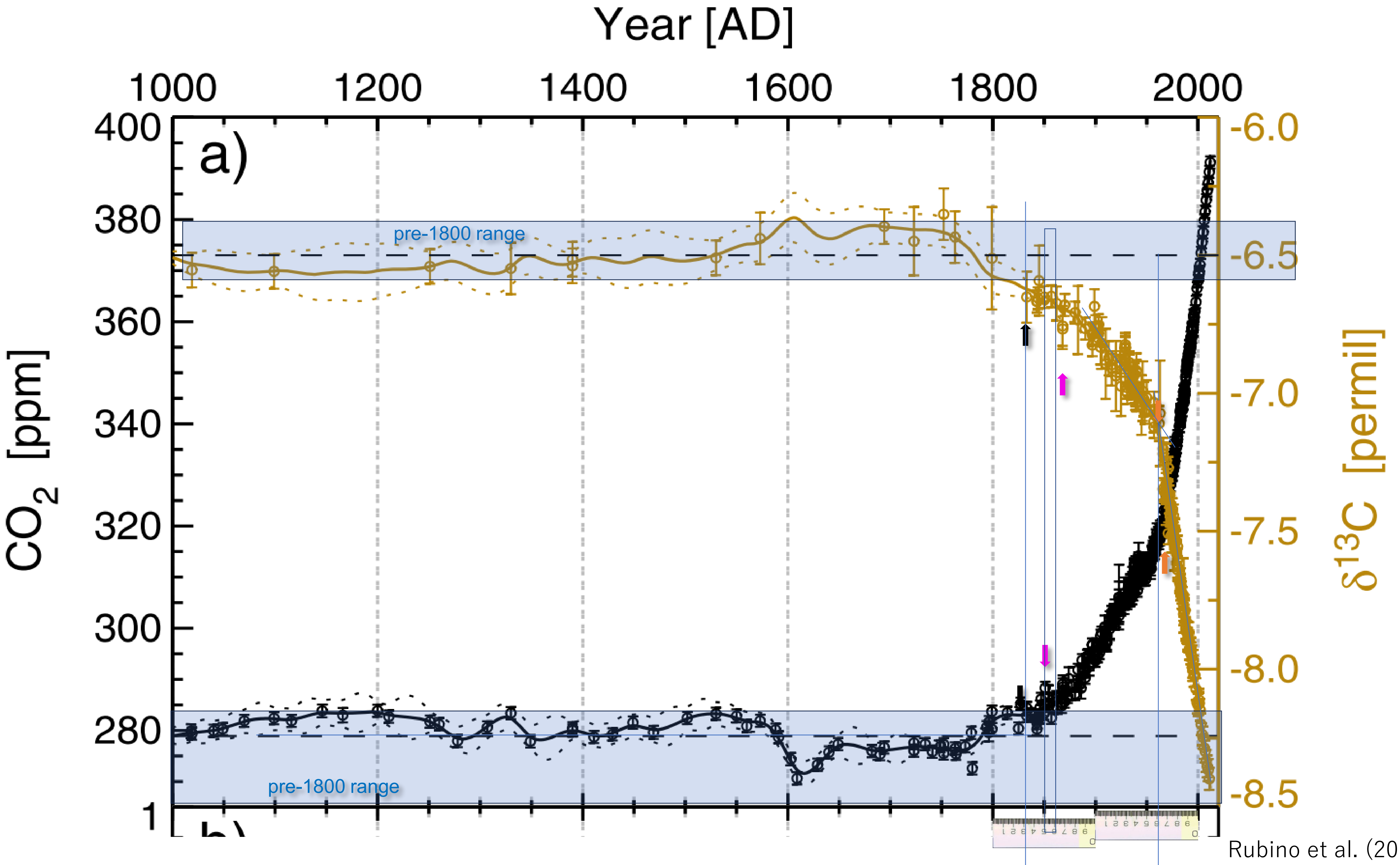

Rubino et al. (2013)

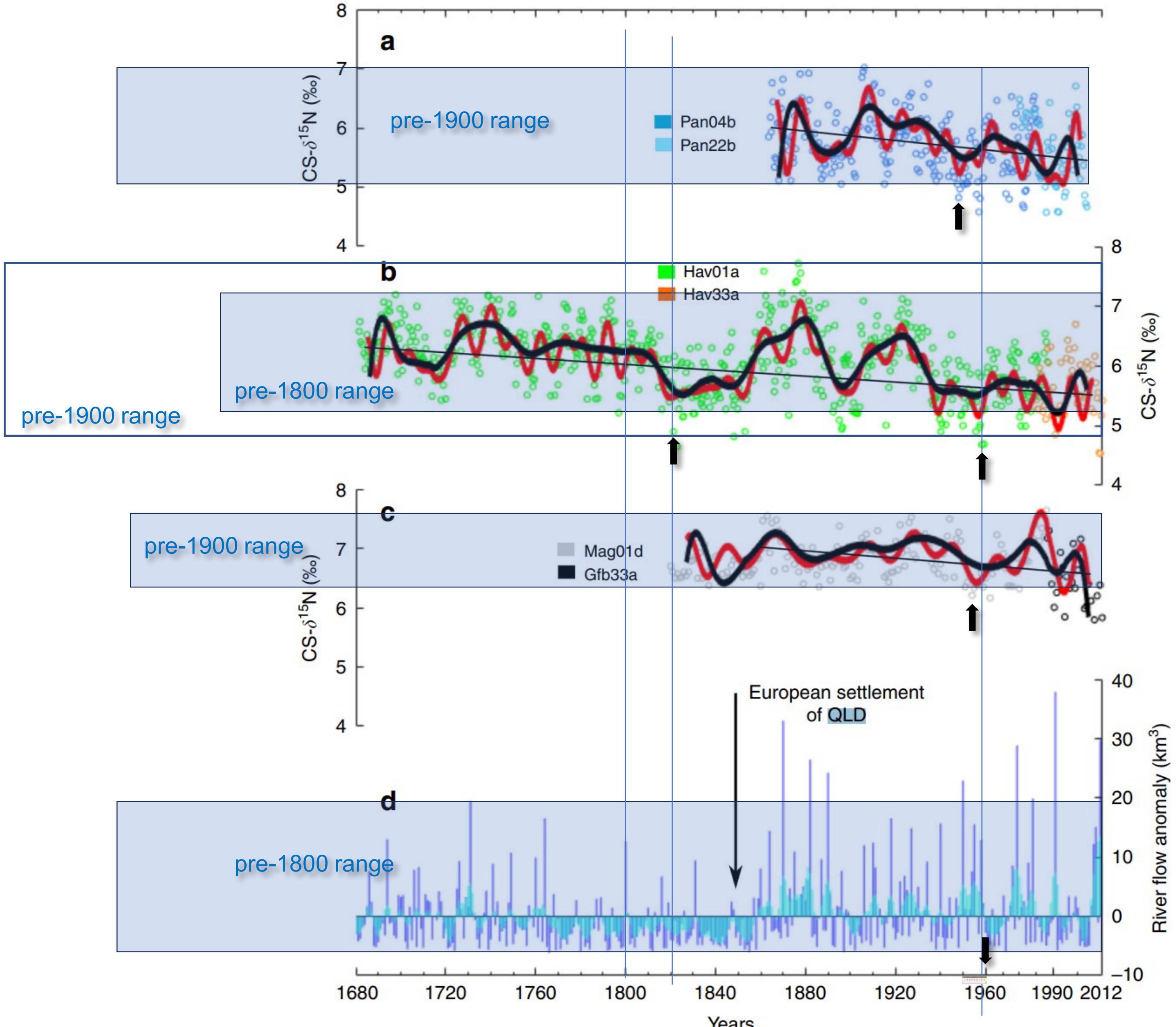

Erler et al. (2020)

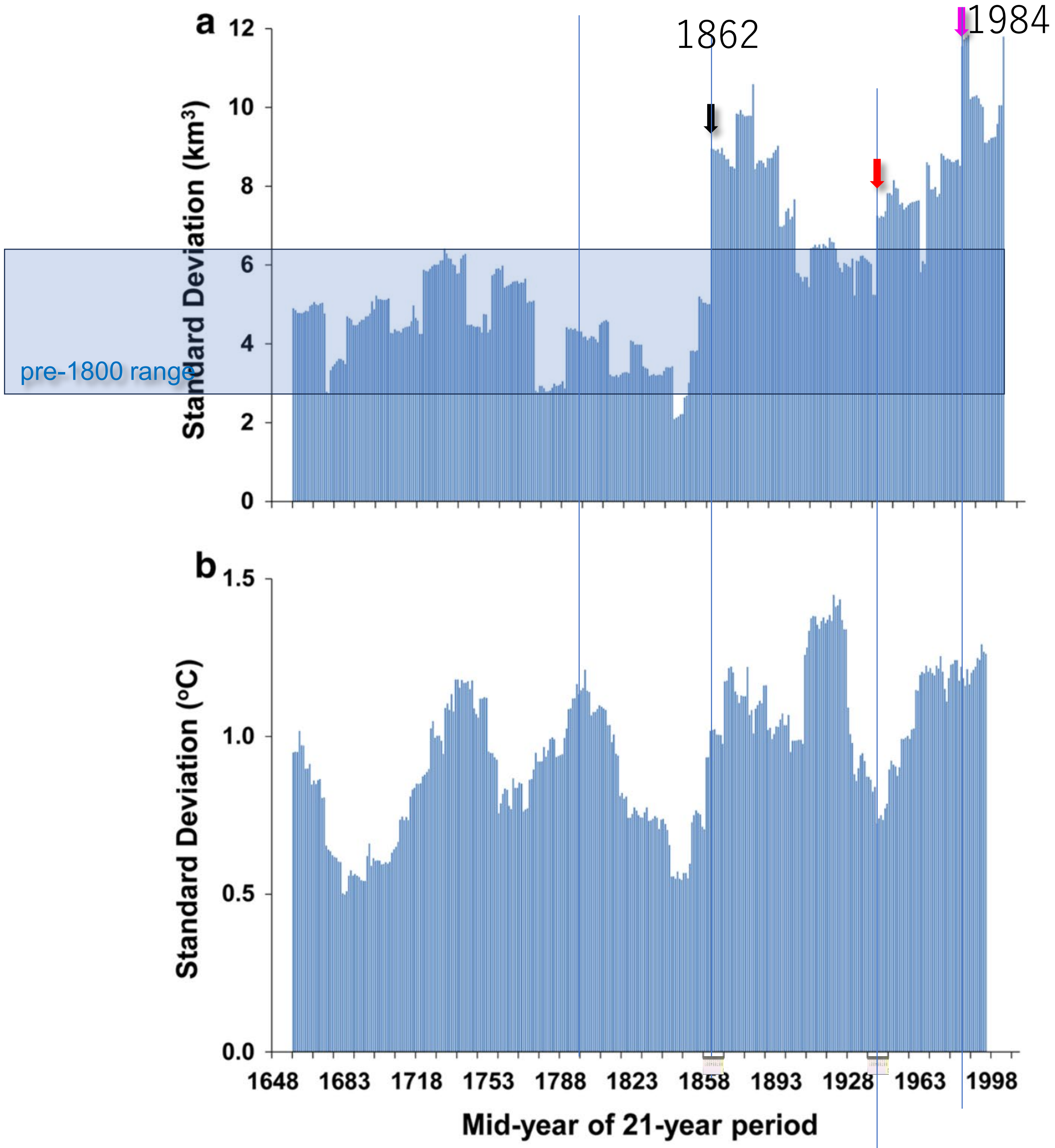

Lough et al., 2015

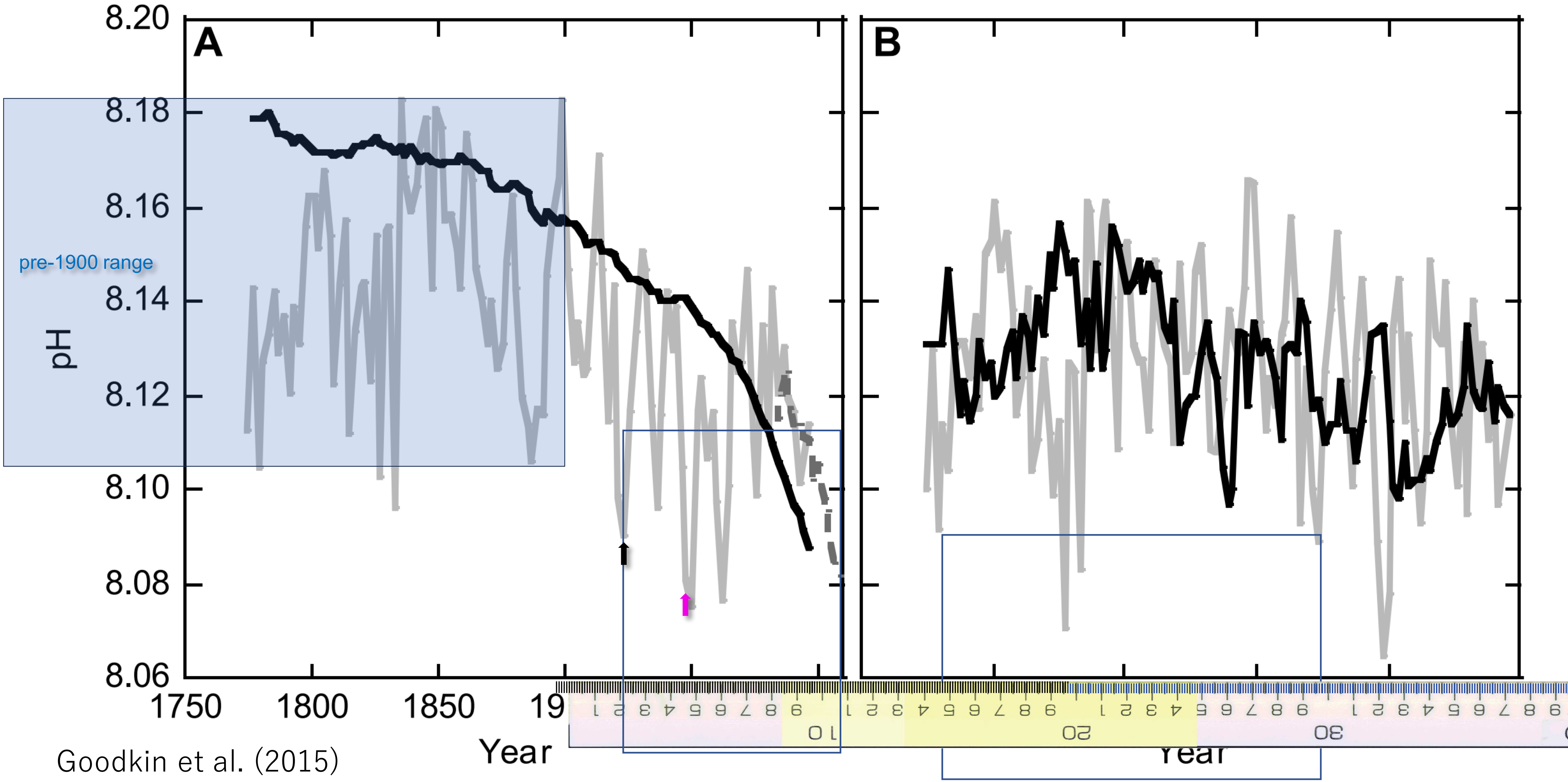

Goodkin et al. (2015)

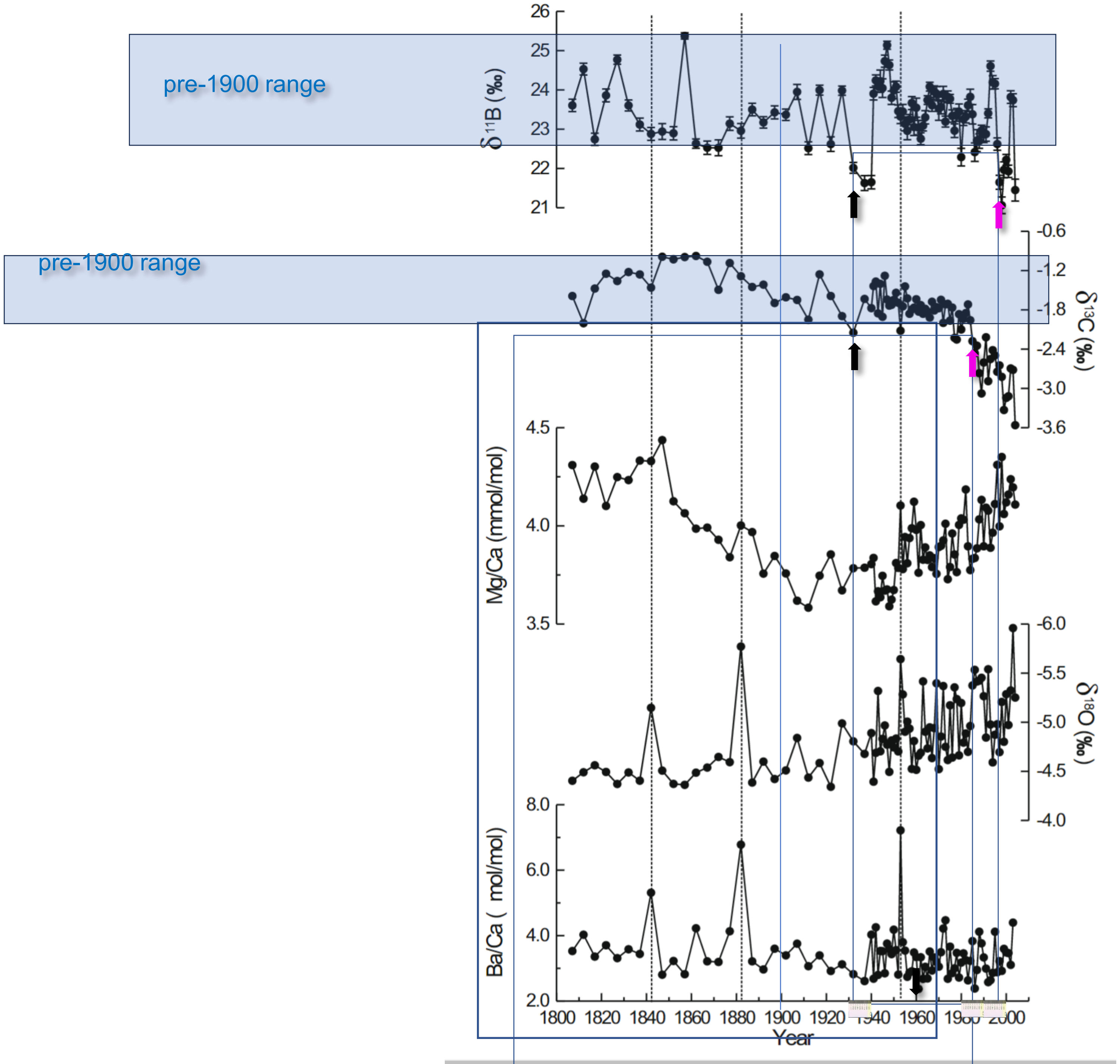

Wei et al., 2009

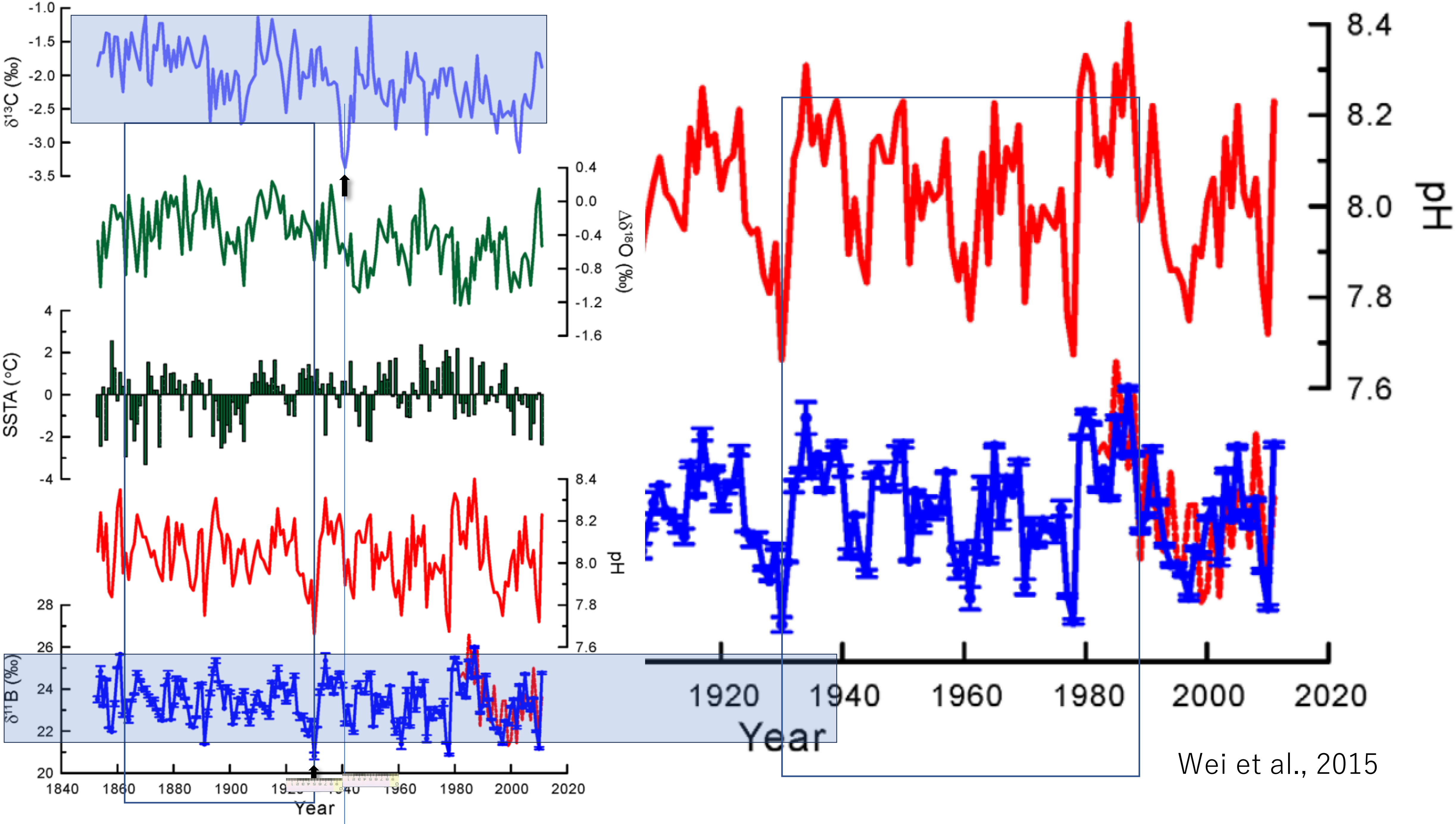

Wei et al., 2015

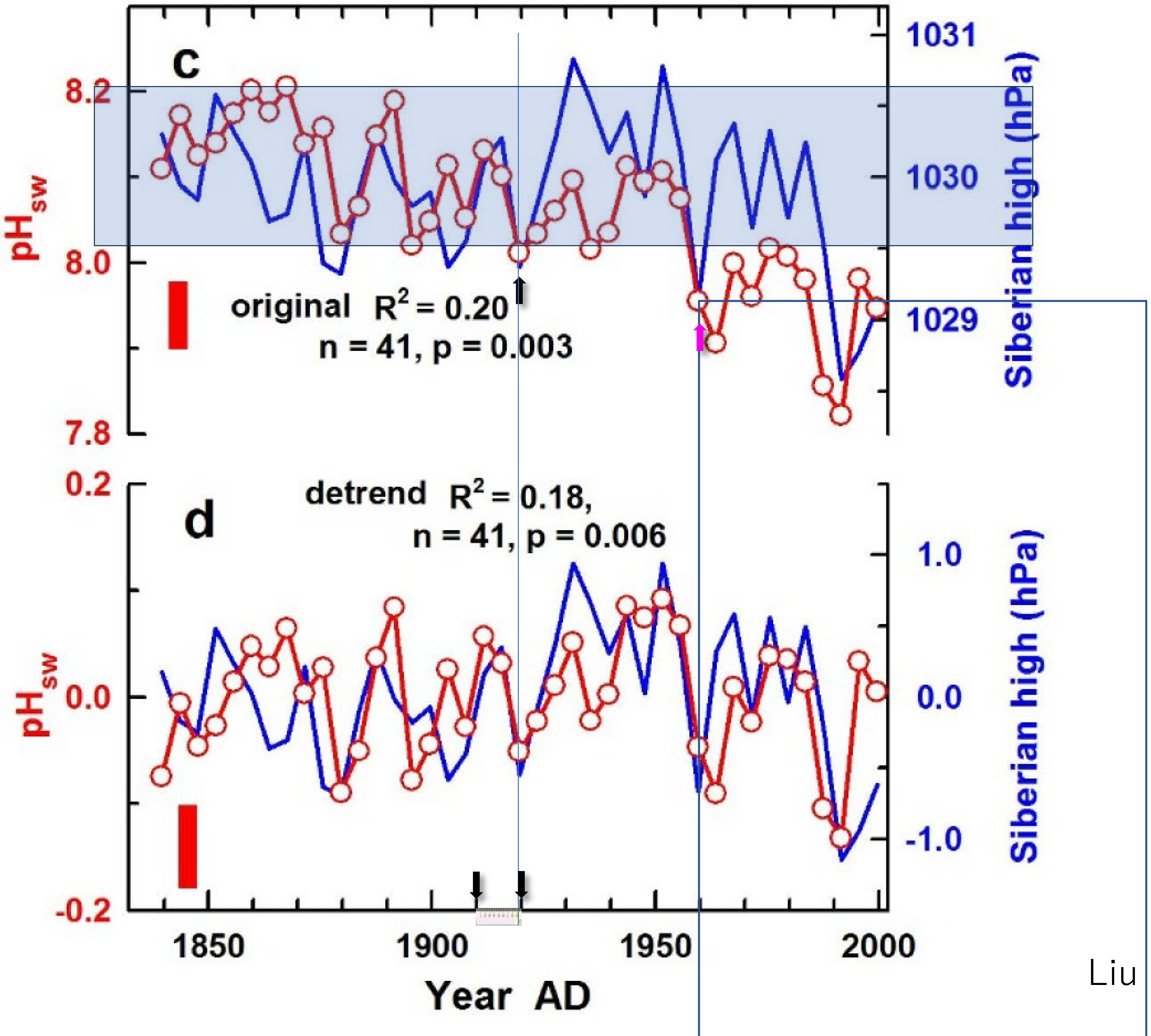

Liu et al., 2014

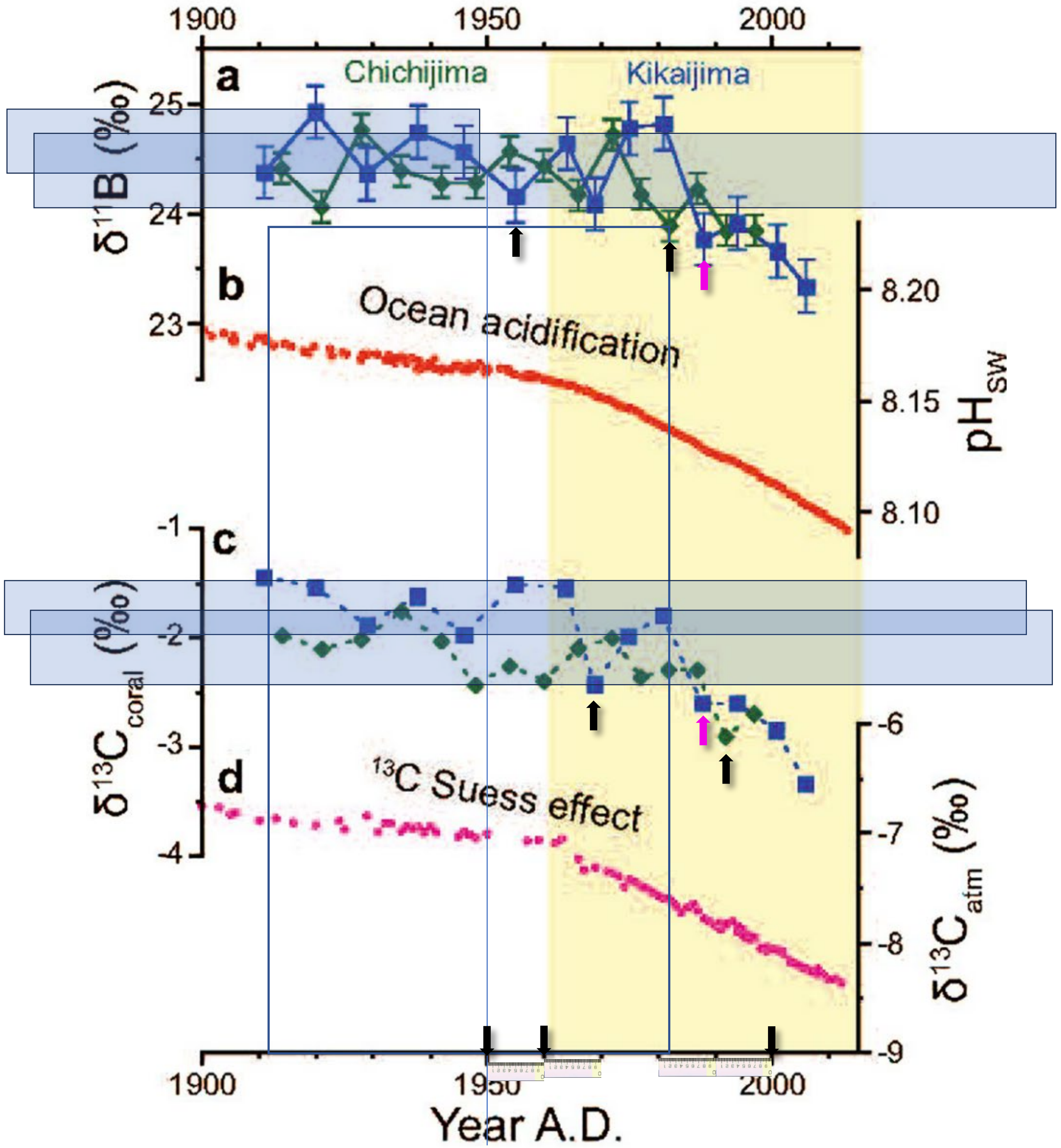

Kubota et al., 2017

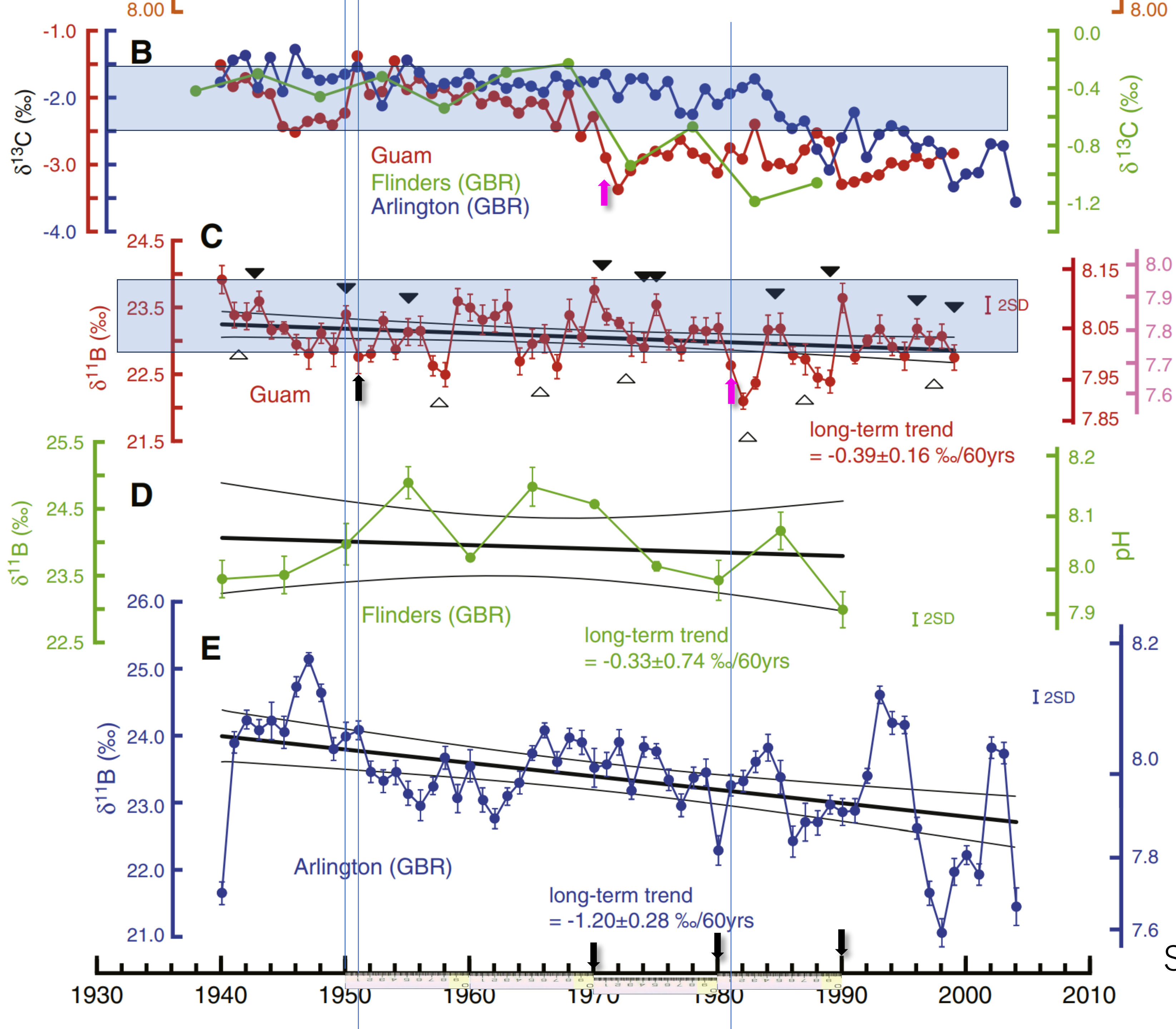

Shinjo et al., 2013

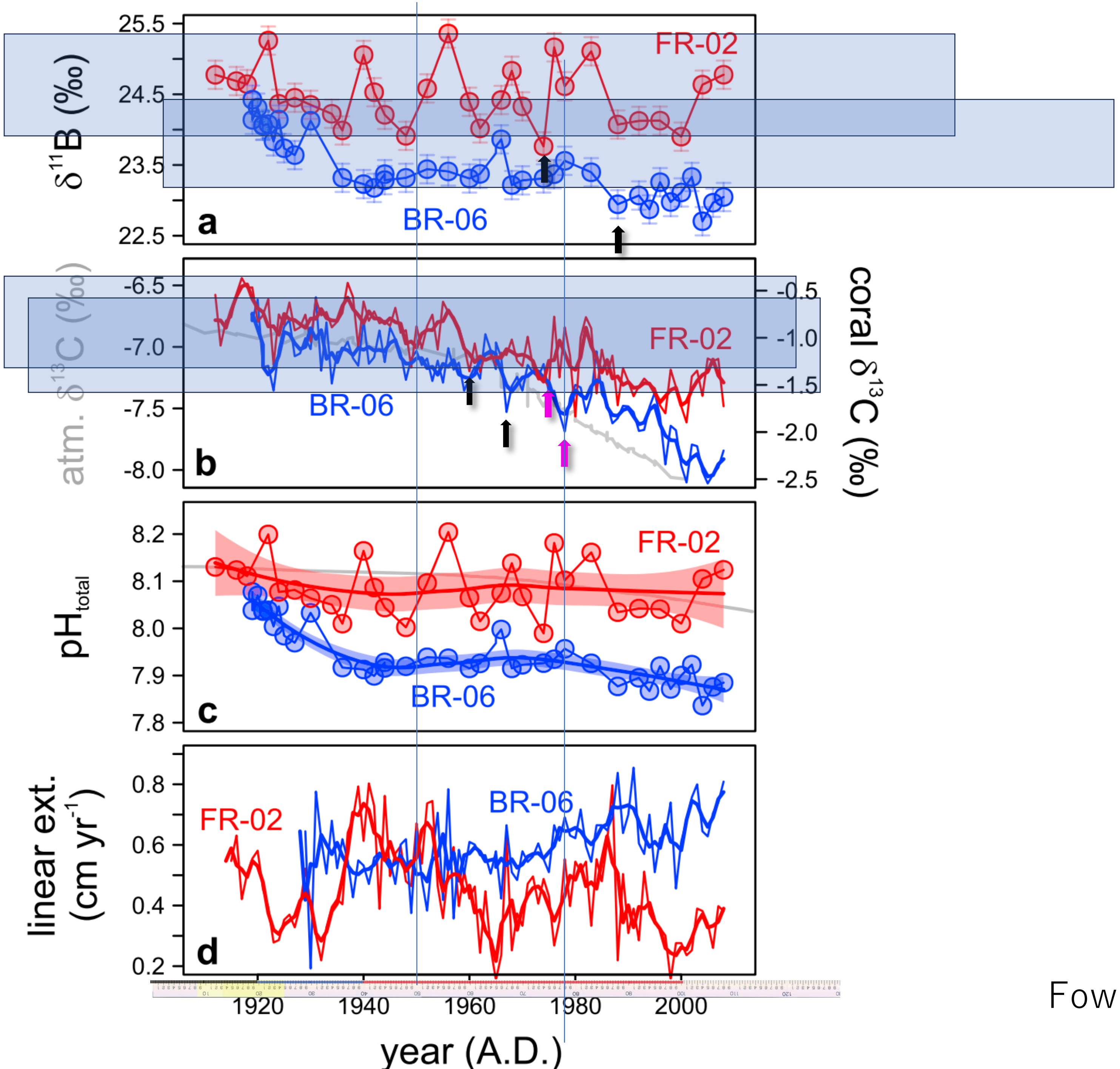

Fowell et al., 2018

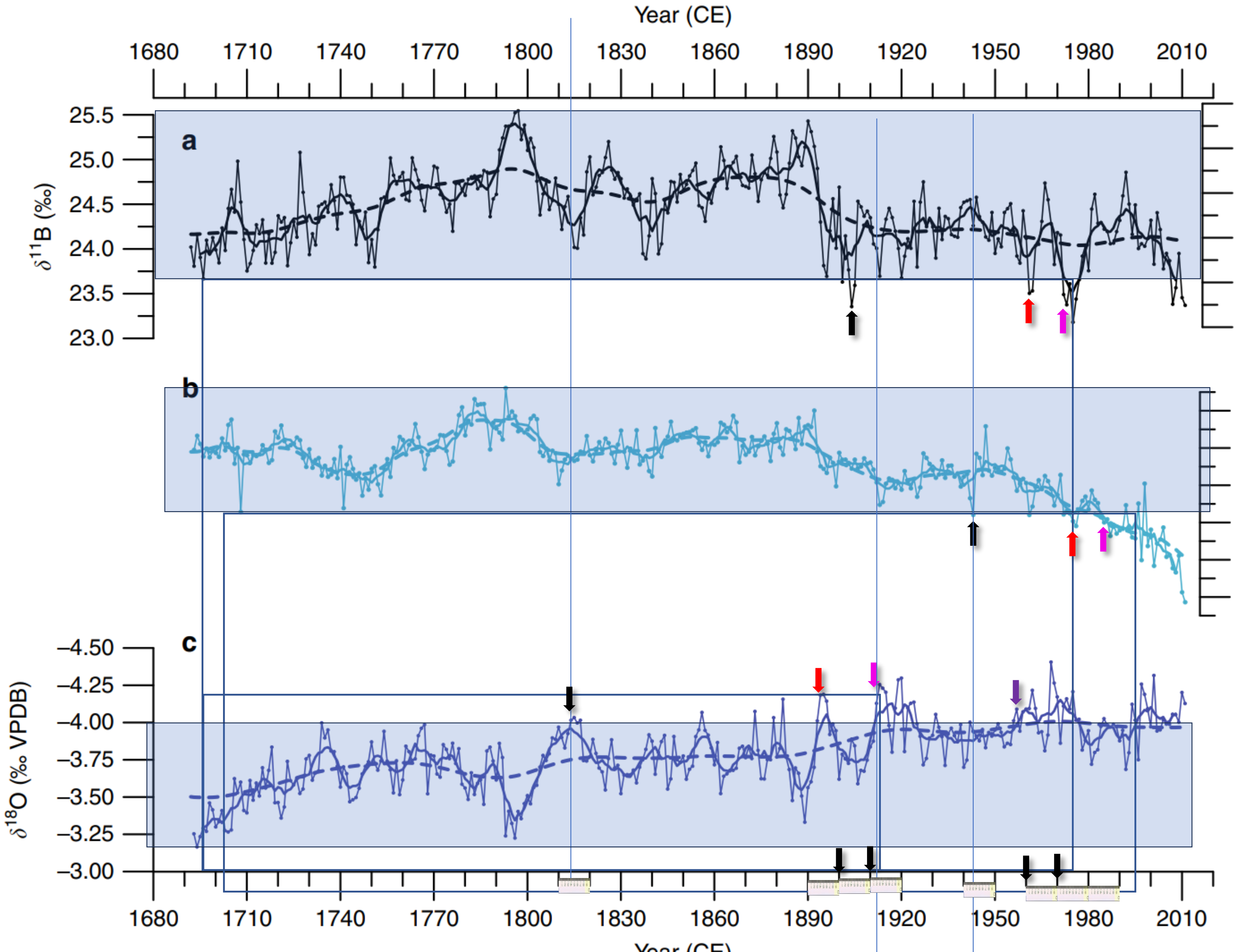
Year (CE)
1680
1710
1740
1770
1800
1830
1860
1890
1920
1950
1980
2010
a
b
c
$\delta^{11}B$ (‰)
25.5
25.0
24.5
24.0
23.5
23.0
8.2
8.1
8.0
7.9
7.8
7.7
Estimated seawater pH
0.8
0.4
0.0
−0.4
−0.8
−1.2
$\delta^{13}C$ (‰ VPDB)
−4.50
−4.25
−4.00
−3.75
−3.50
−3.25
−3.00
$\delta^{18}O$ (‰ VPDB)

Wu et ql., 2018

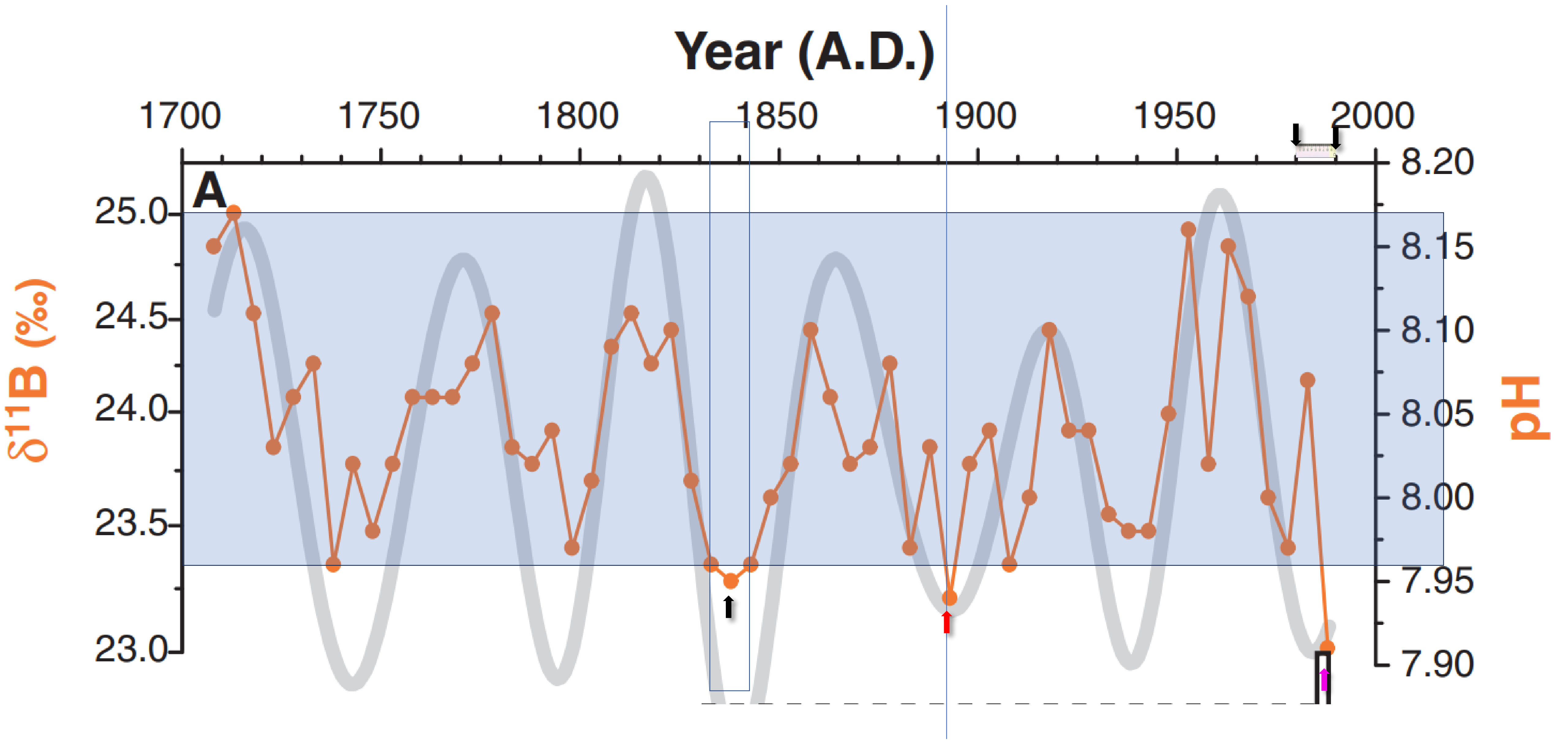

Pelejero et al. 2005

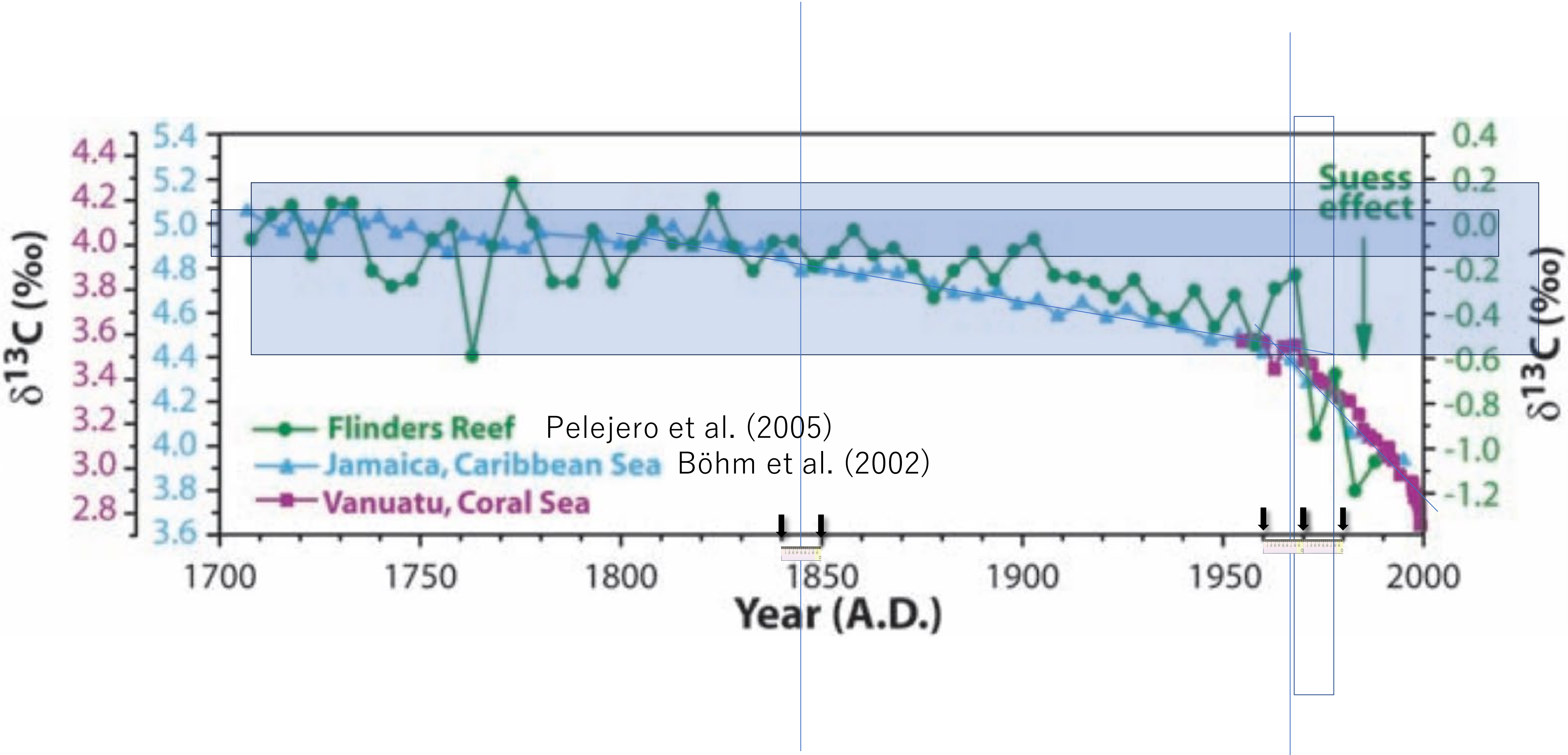

Pelejero et al. (2005)

Böhm et al. (2002)

Pelejero et al. (2005)

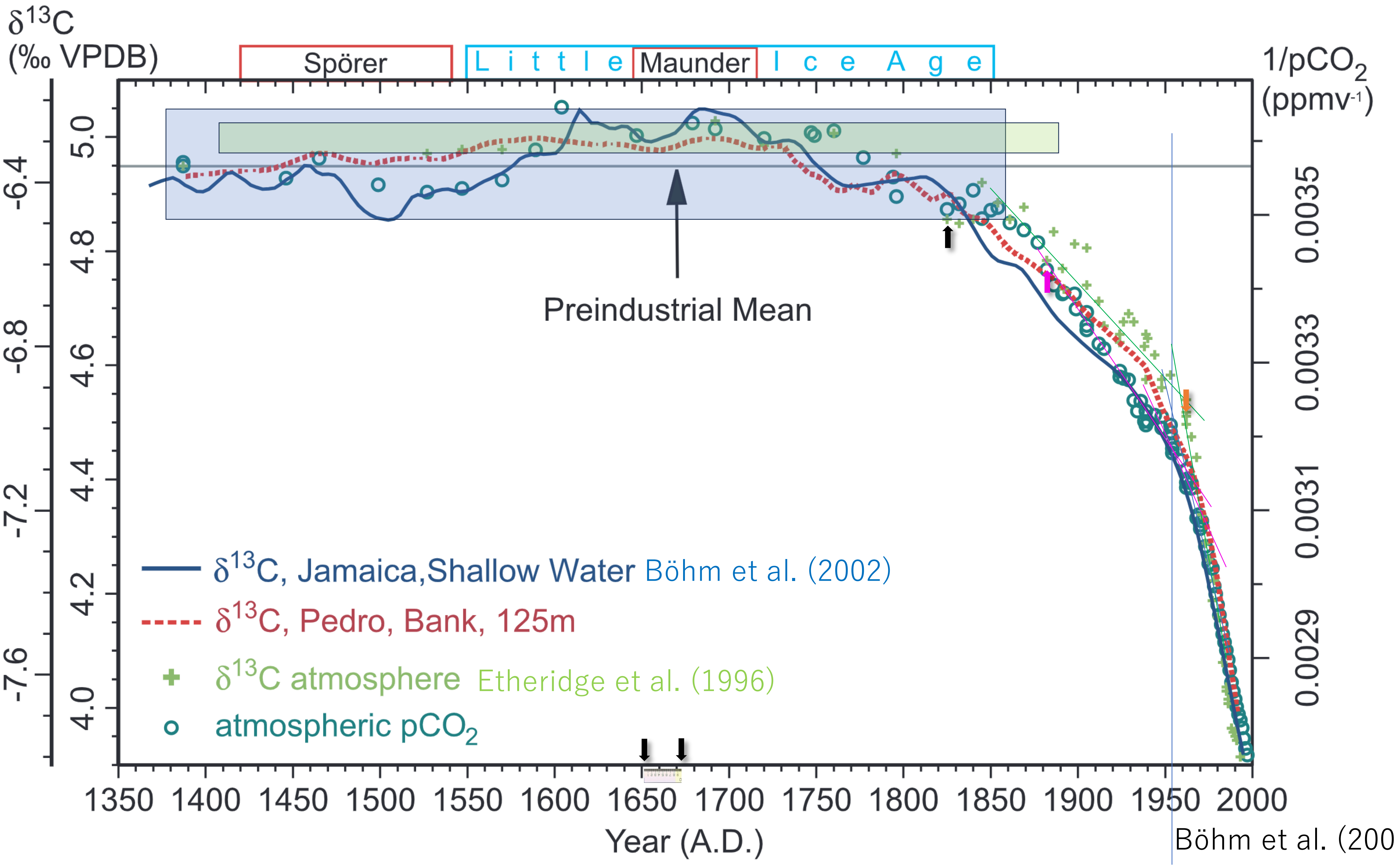

Böhm et al. (2002)

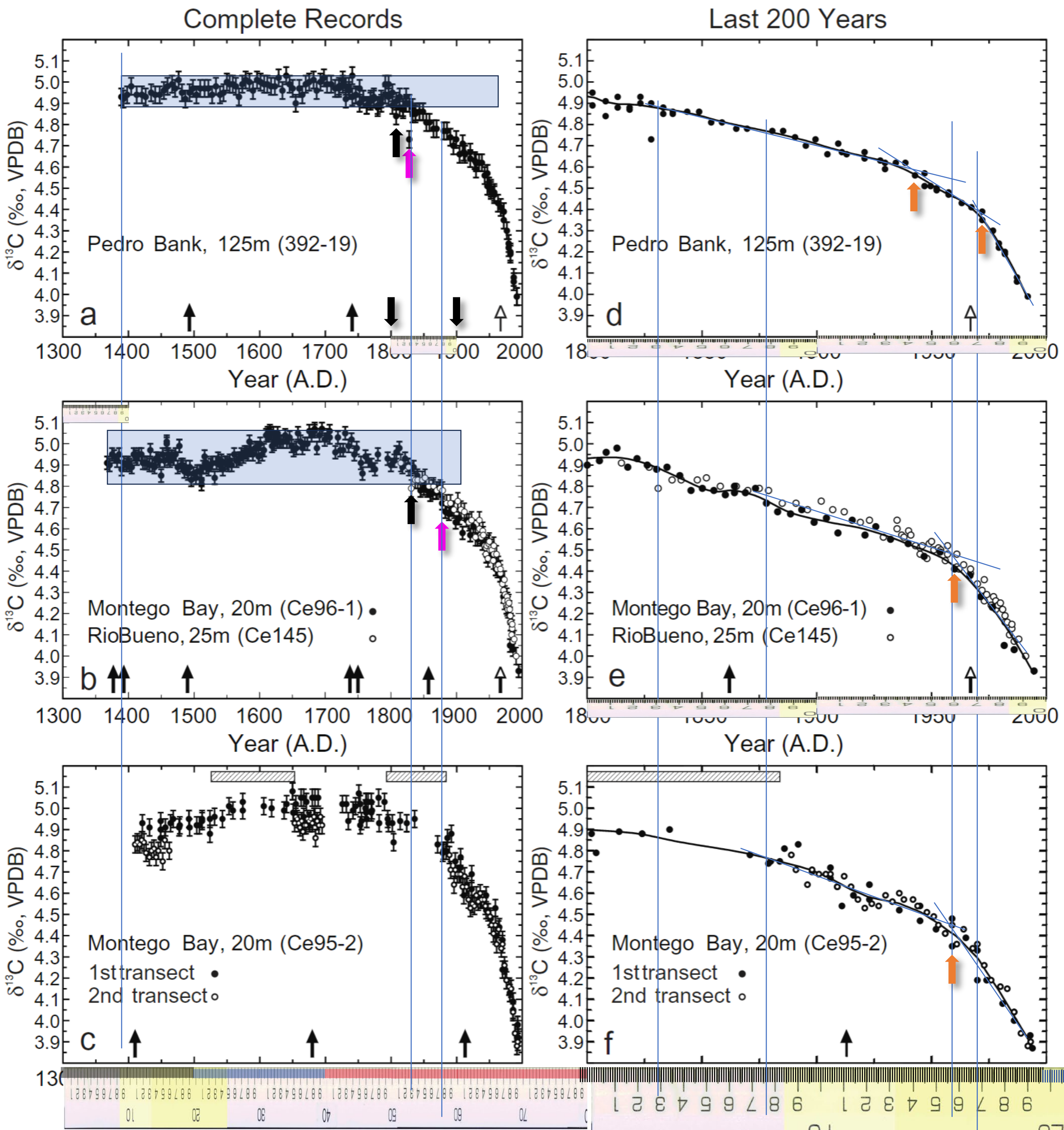

Böhm et al. (2002)

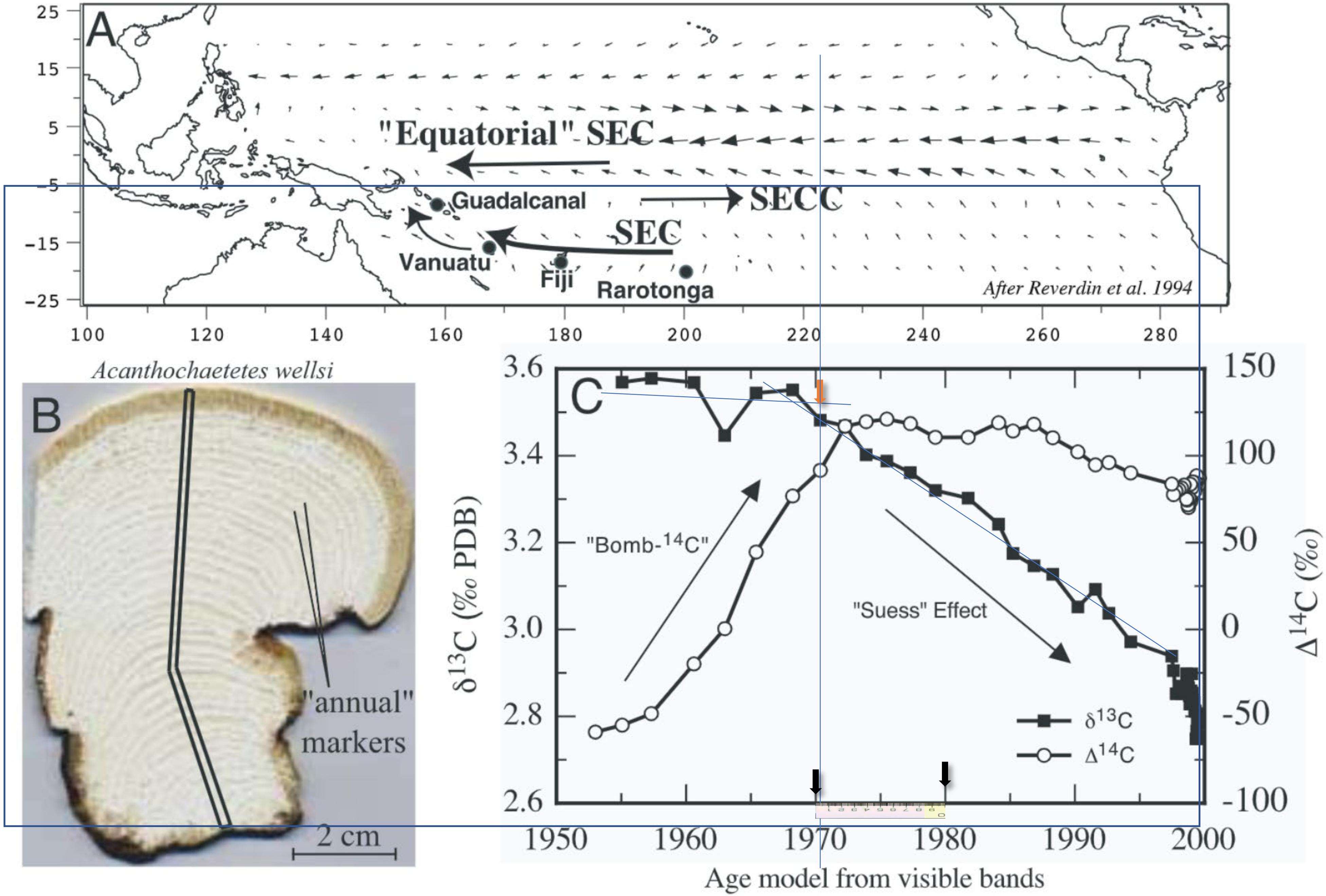

Fallon et al., 2003

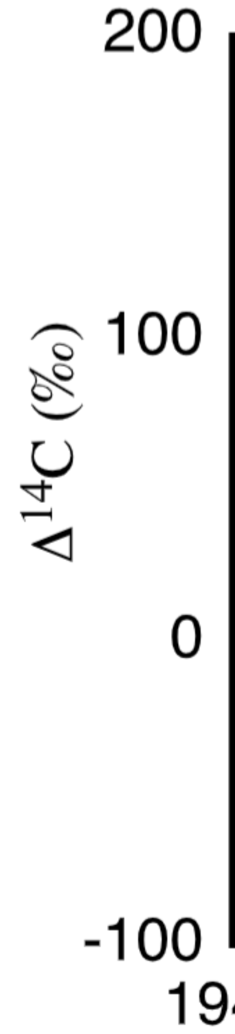

Figure 2.
records to
Raratonga
Tambea c

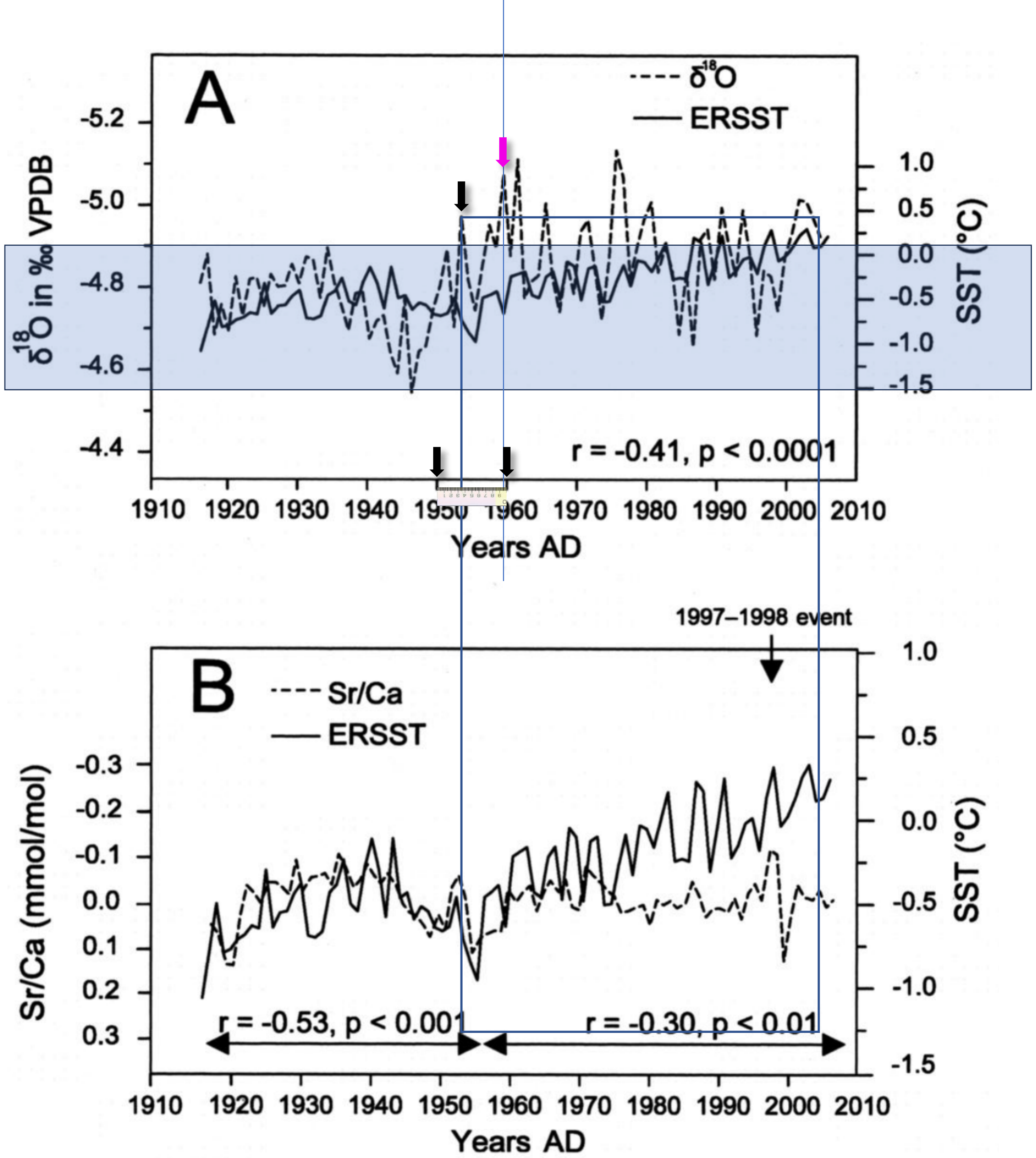

Storz et al., 2013

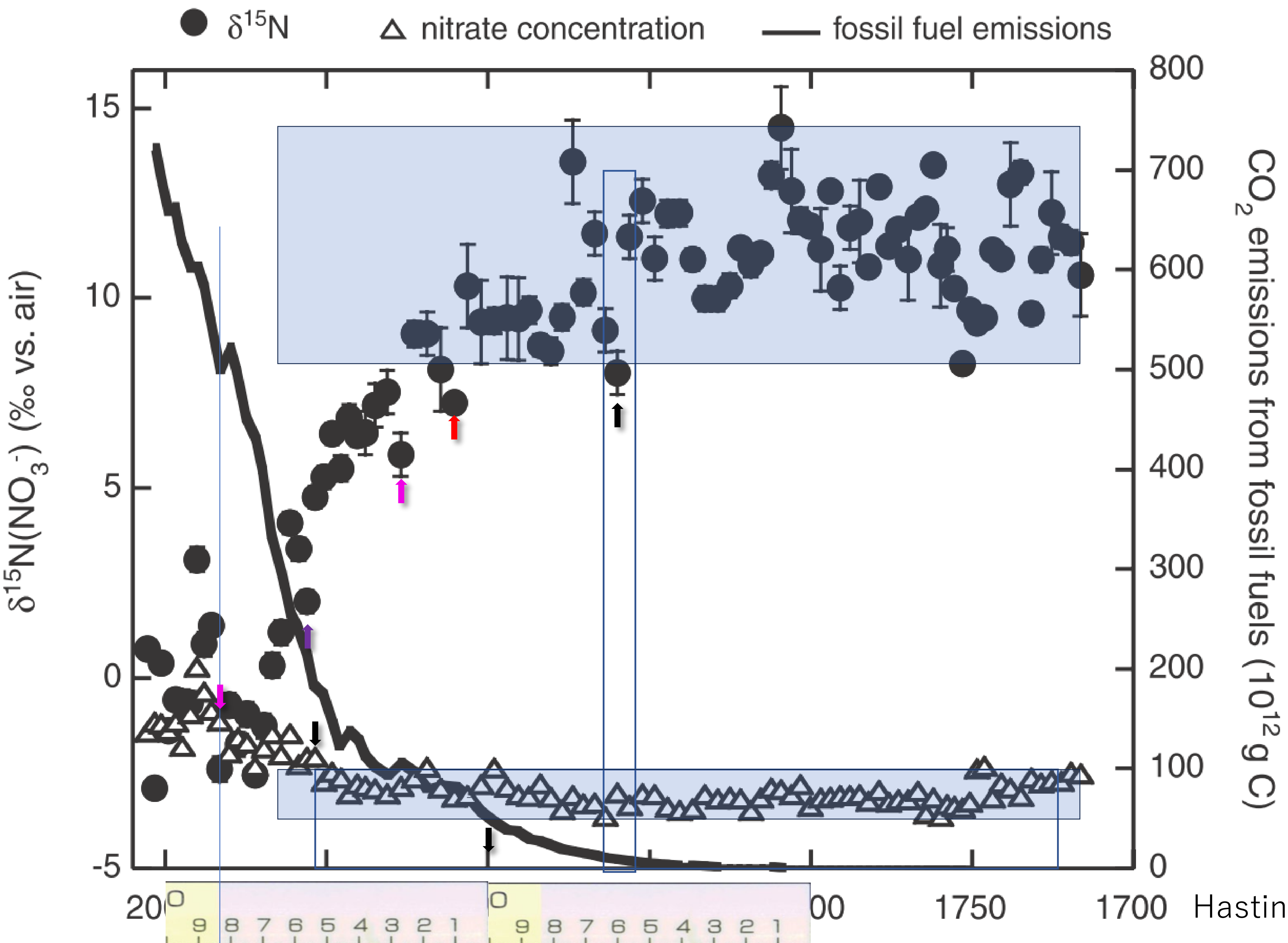

Hastings et al., 2009

Nitrate D17O

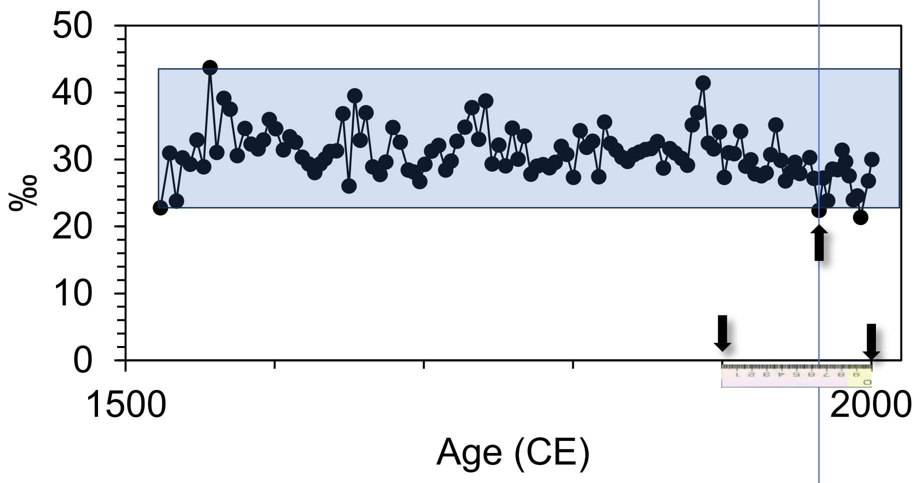

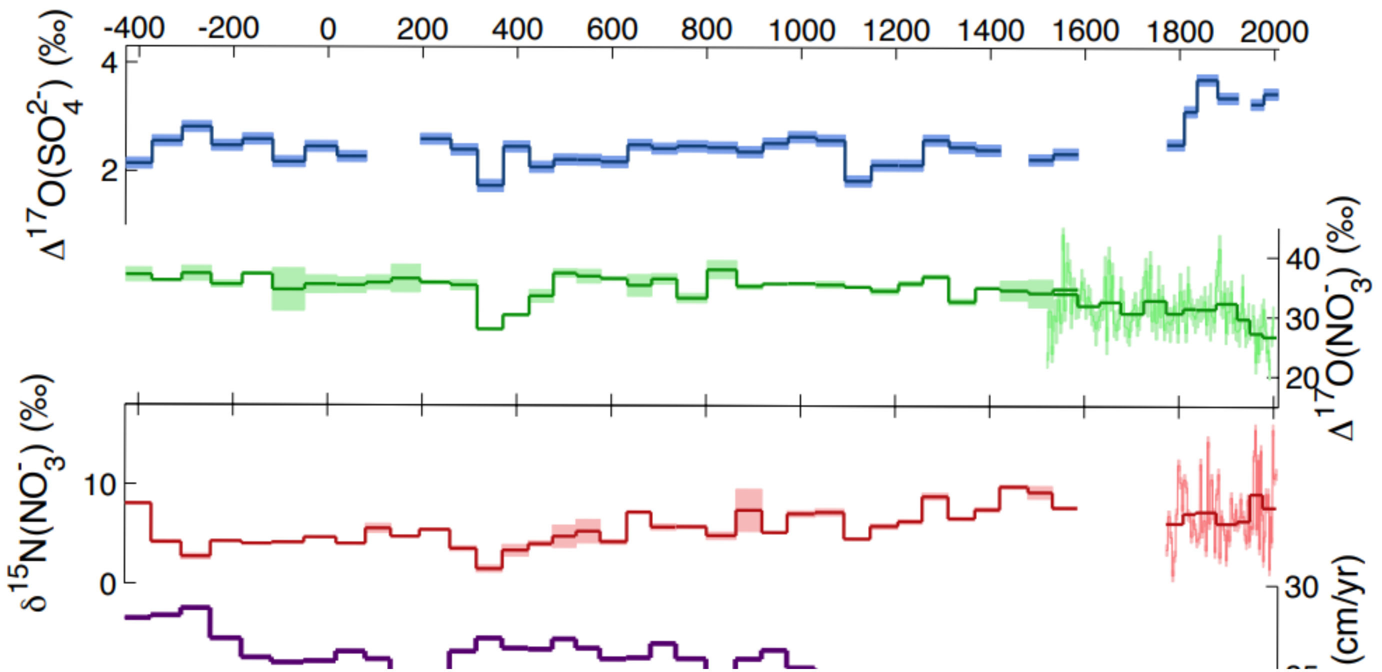

Sofen et al., 2014

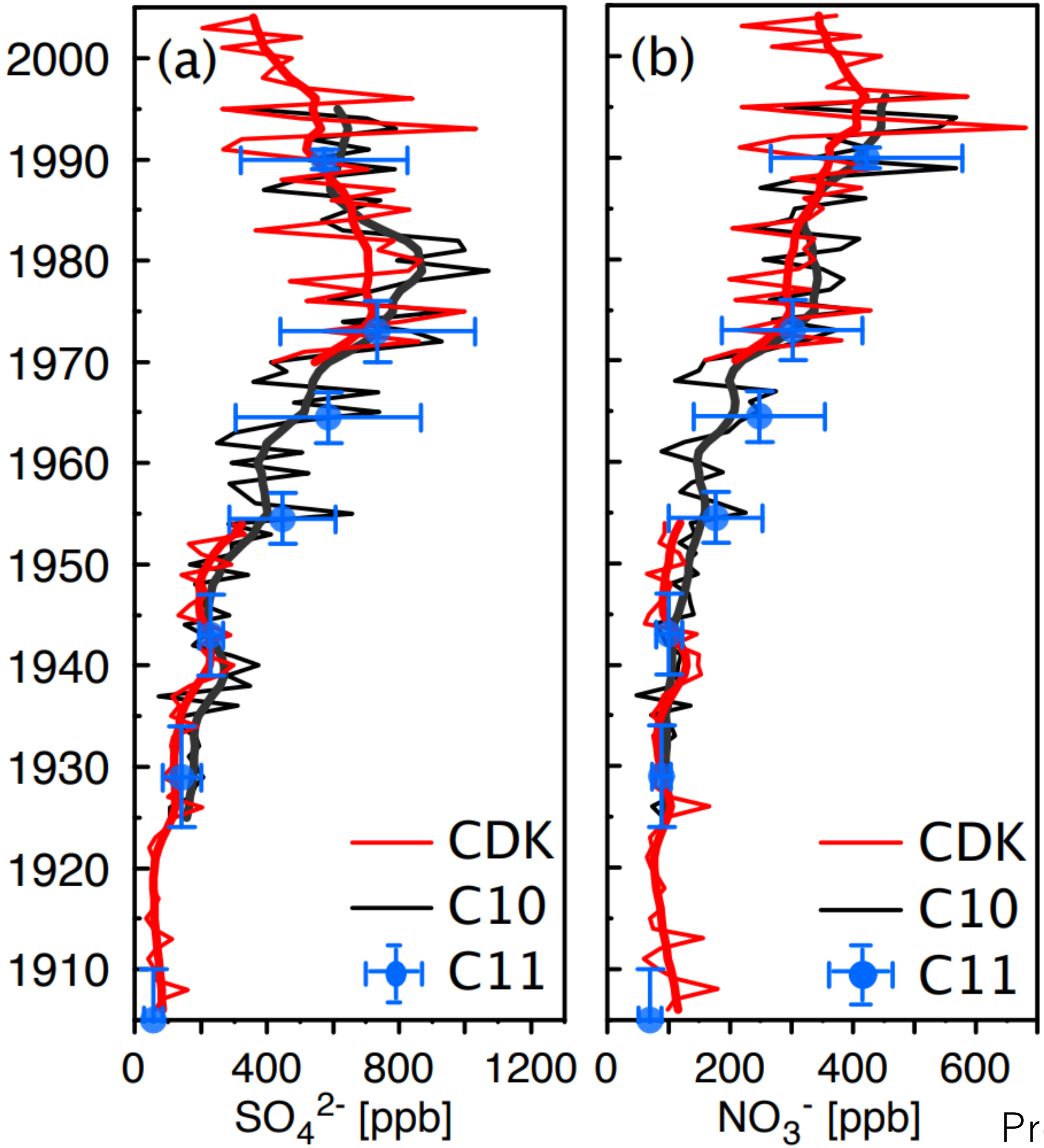

Preunkert et al. (2013)

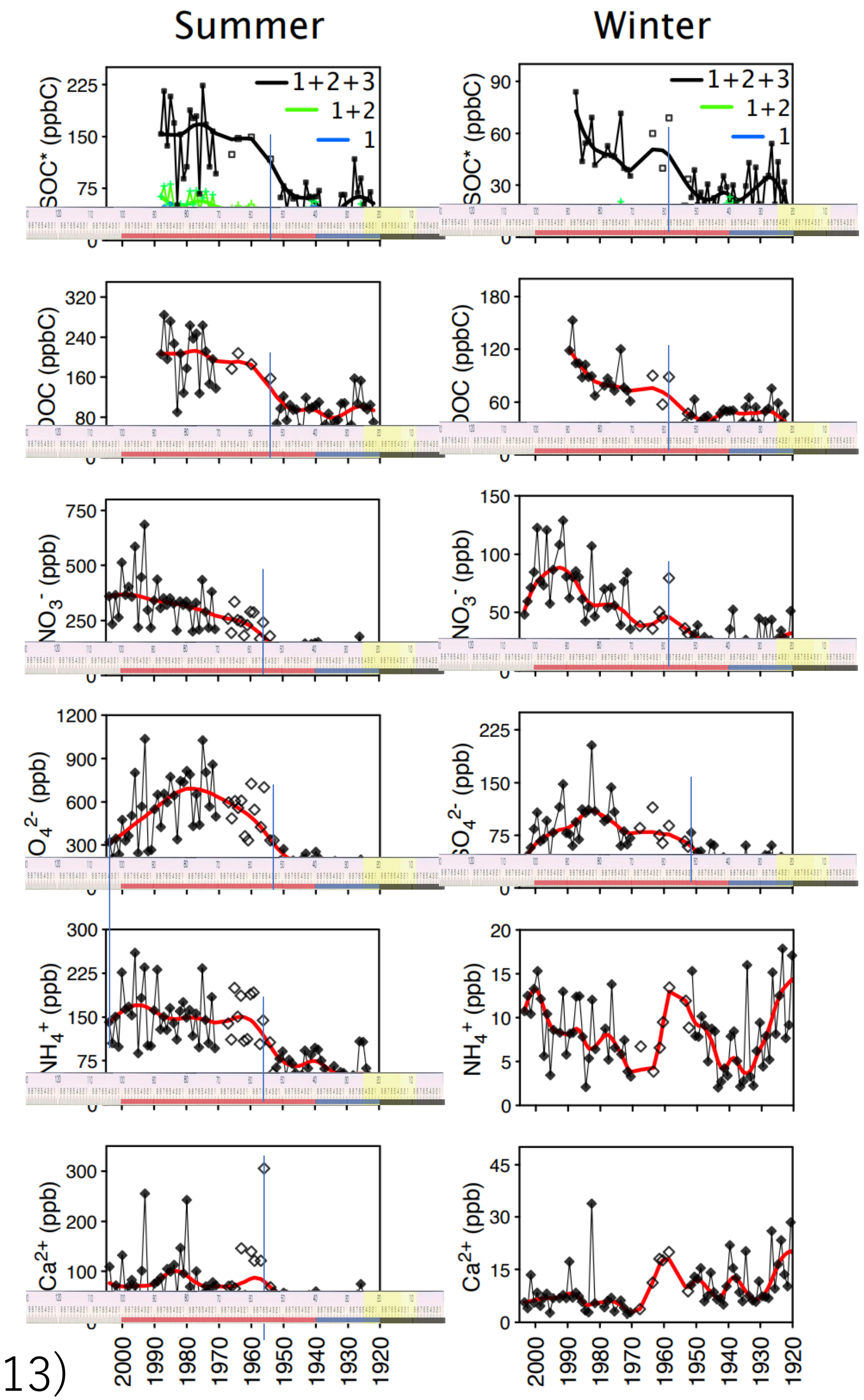

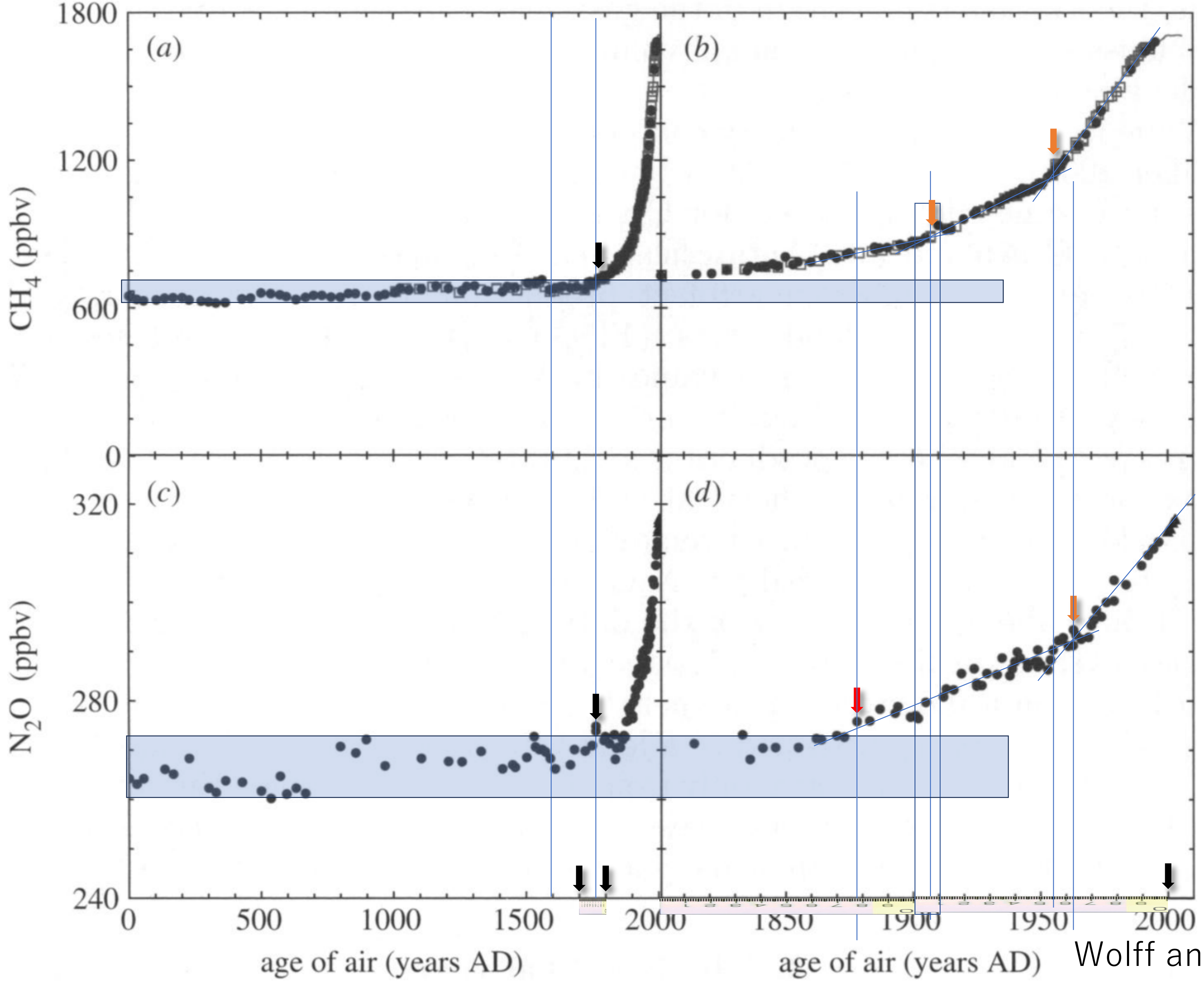

Wolff and Spahni (2007)

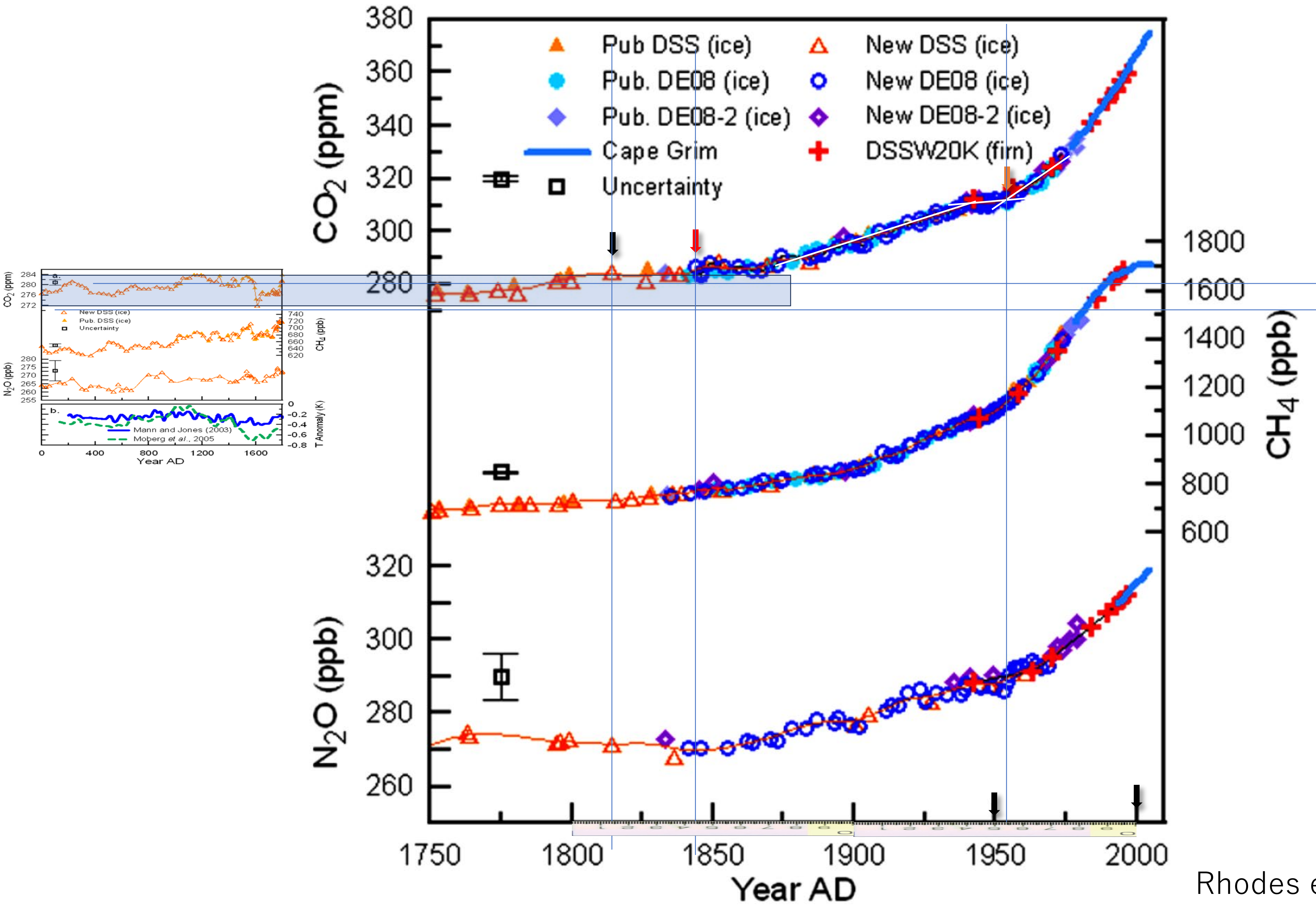

Rhodes et al. (2013)

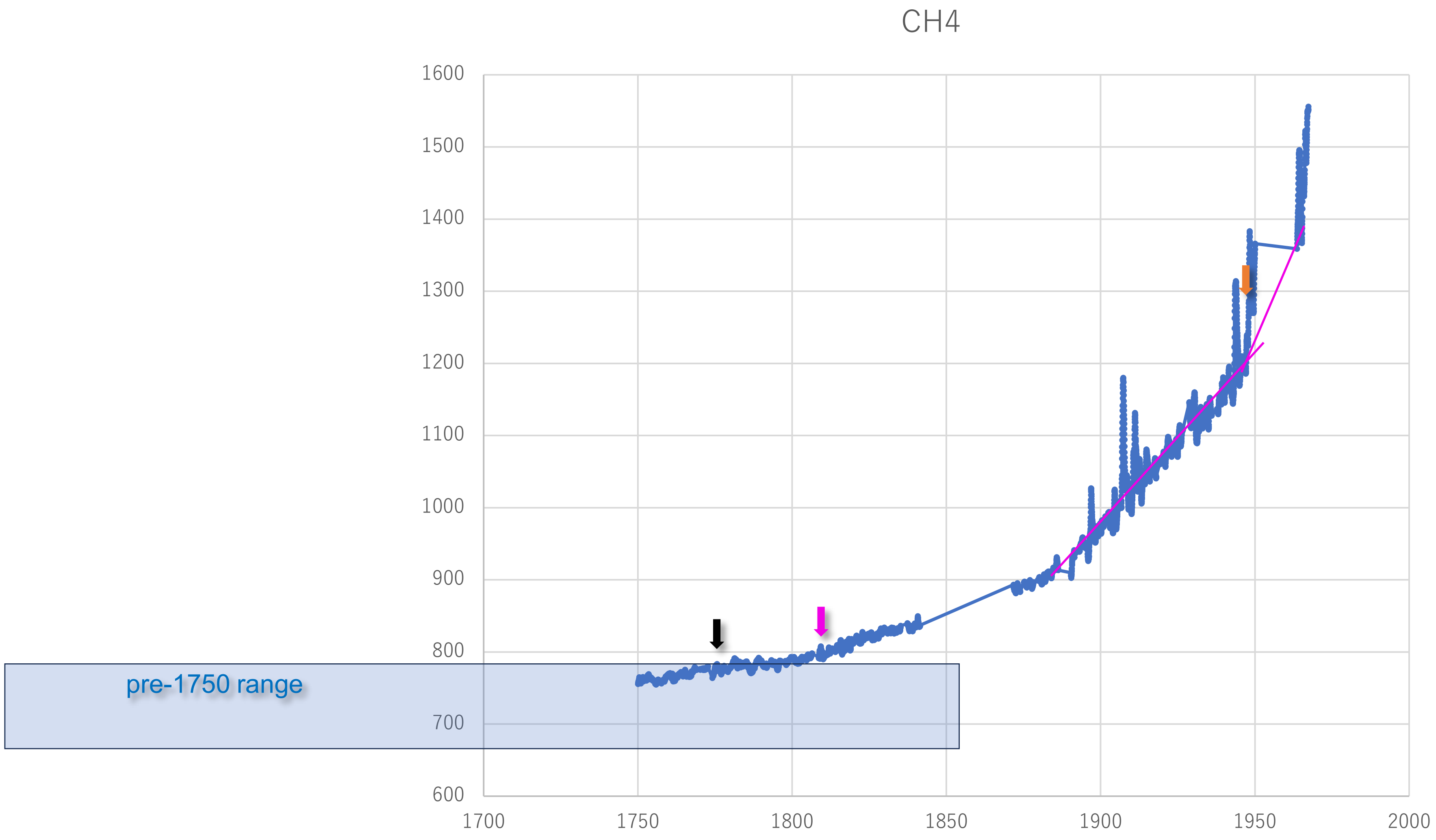

Rhodes et al. (2013)

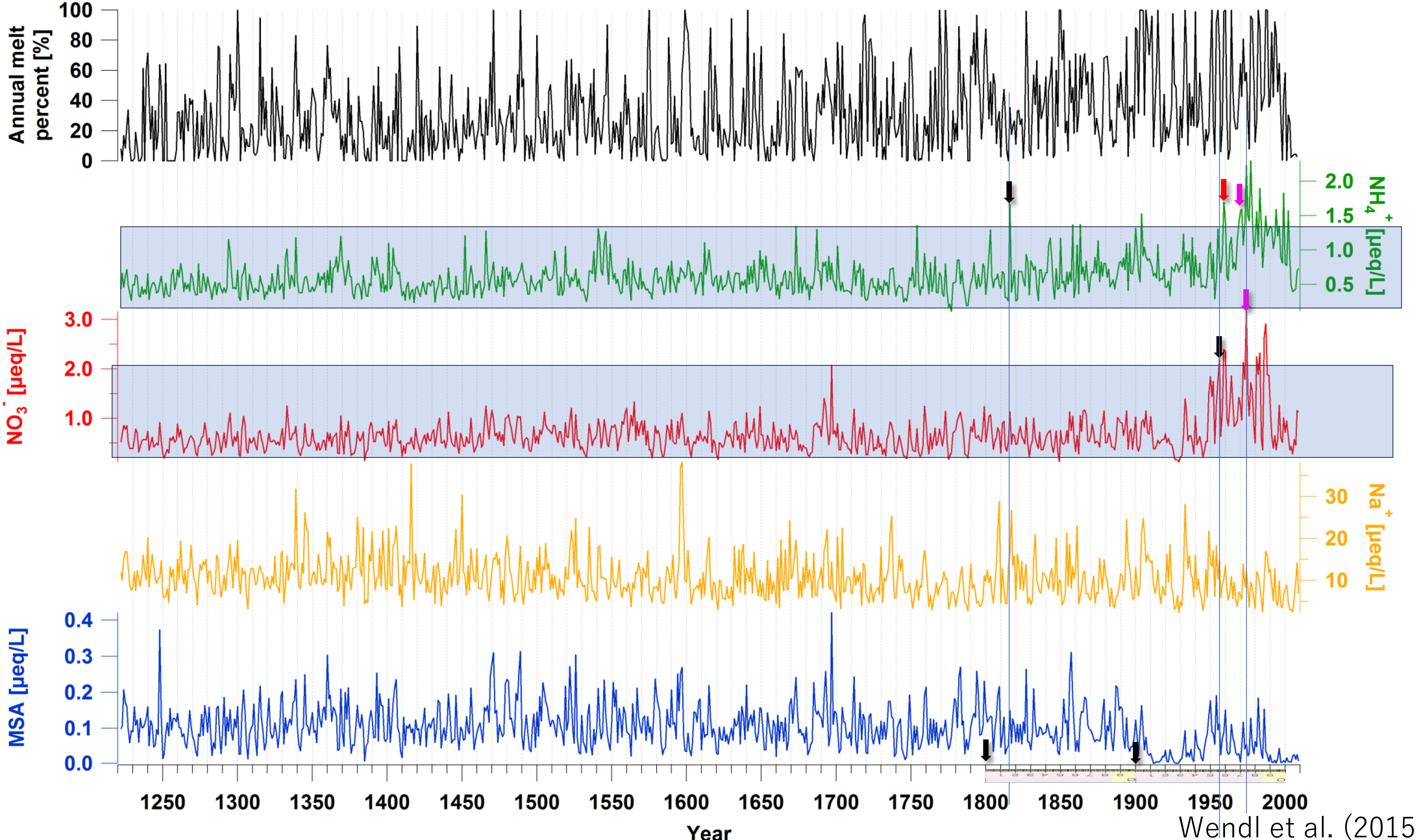

Wendl et al. (2015)

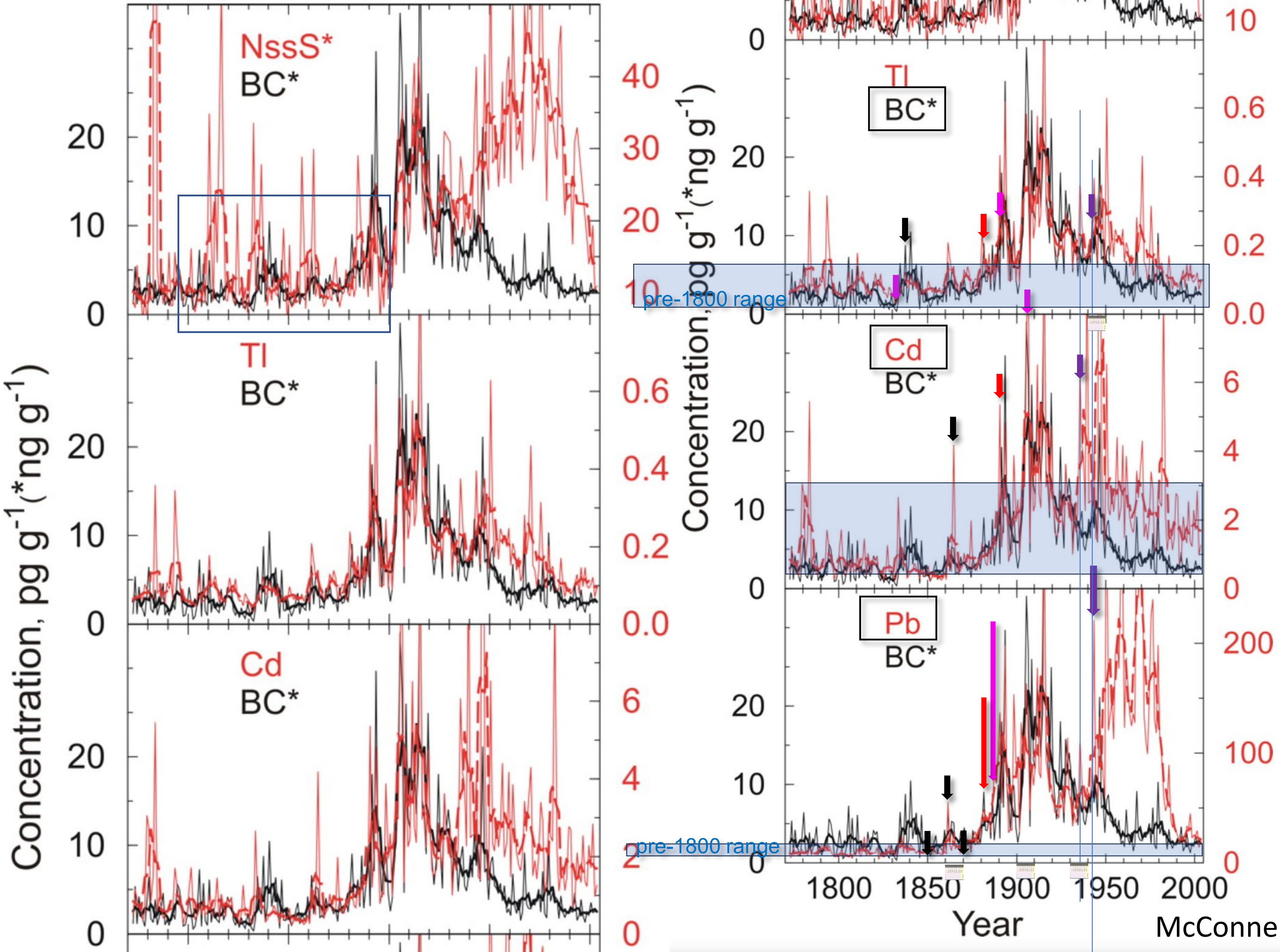

McConnell and Edwards (2008)

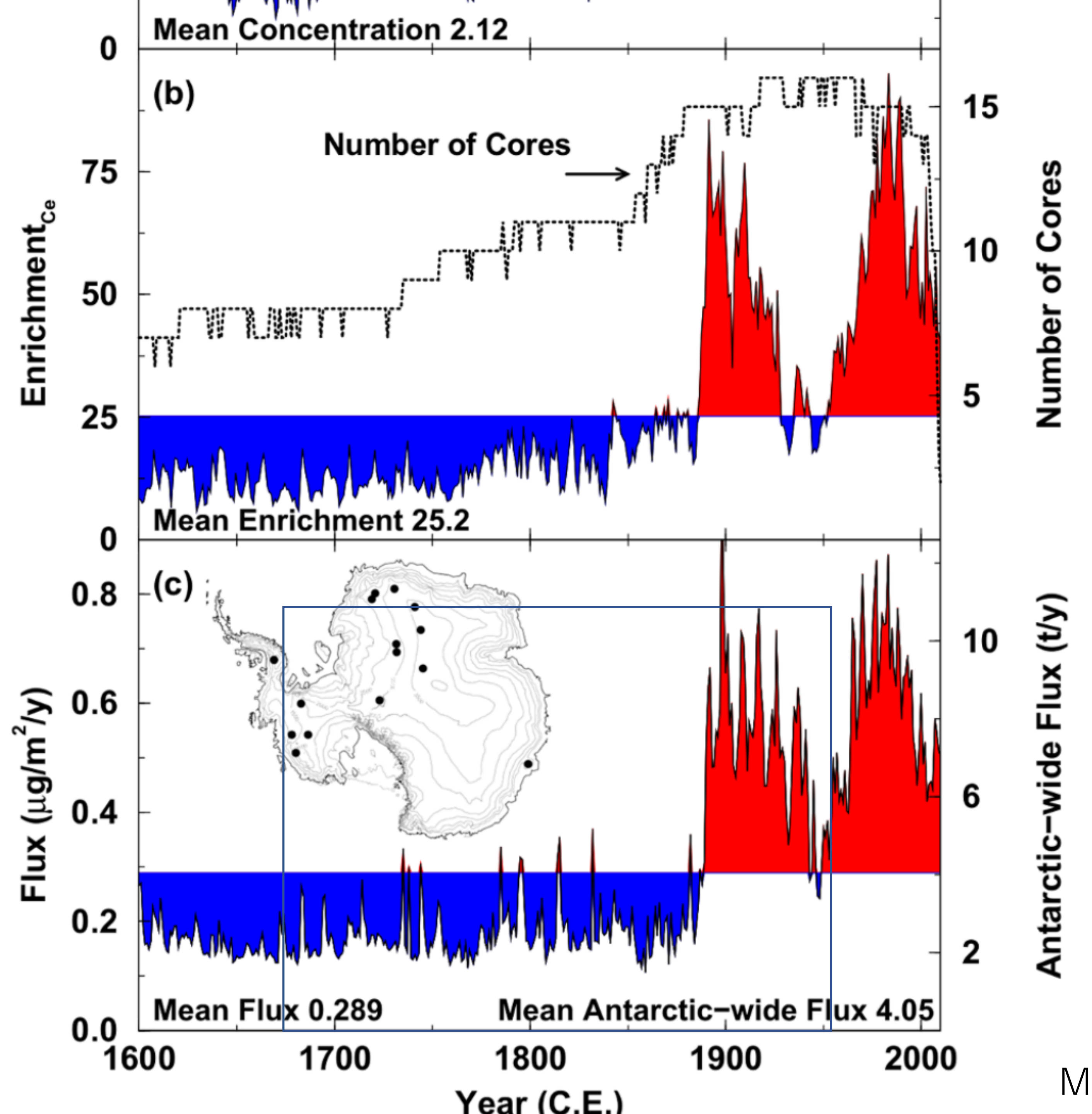

McConnell et al. (2014)

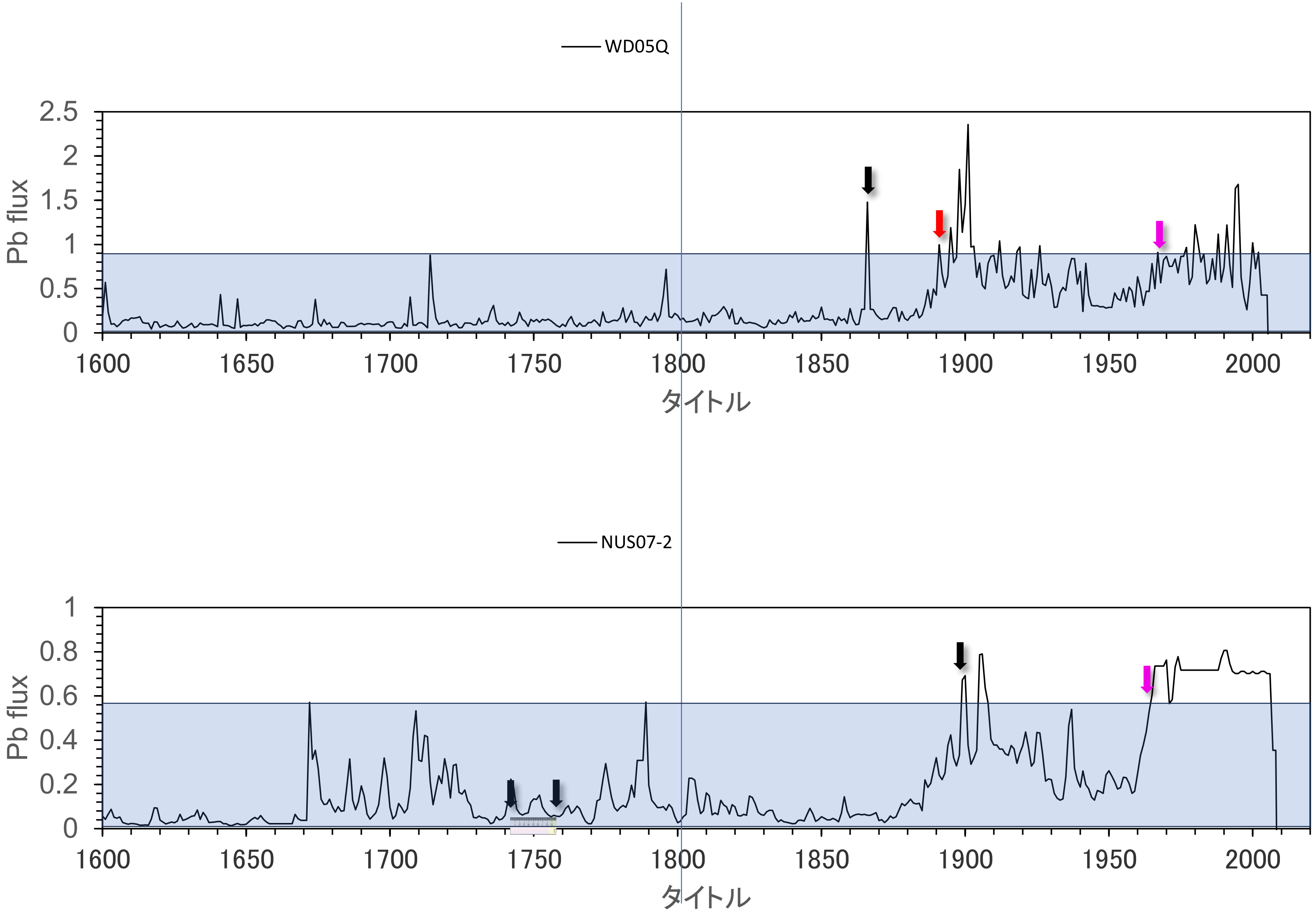

McConnell et al. (2014)

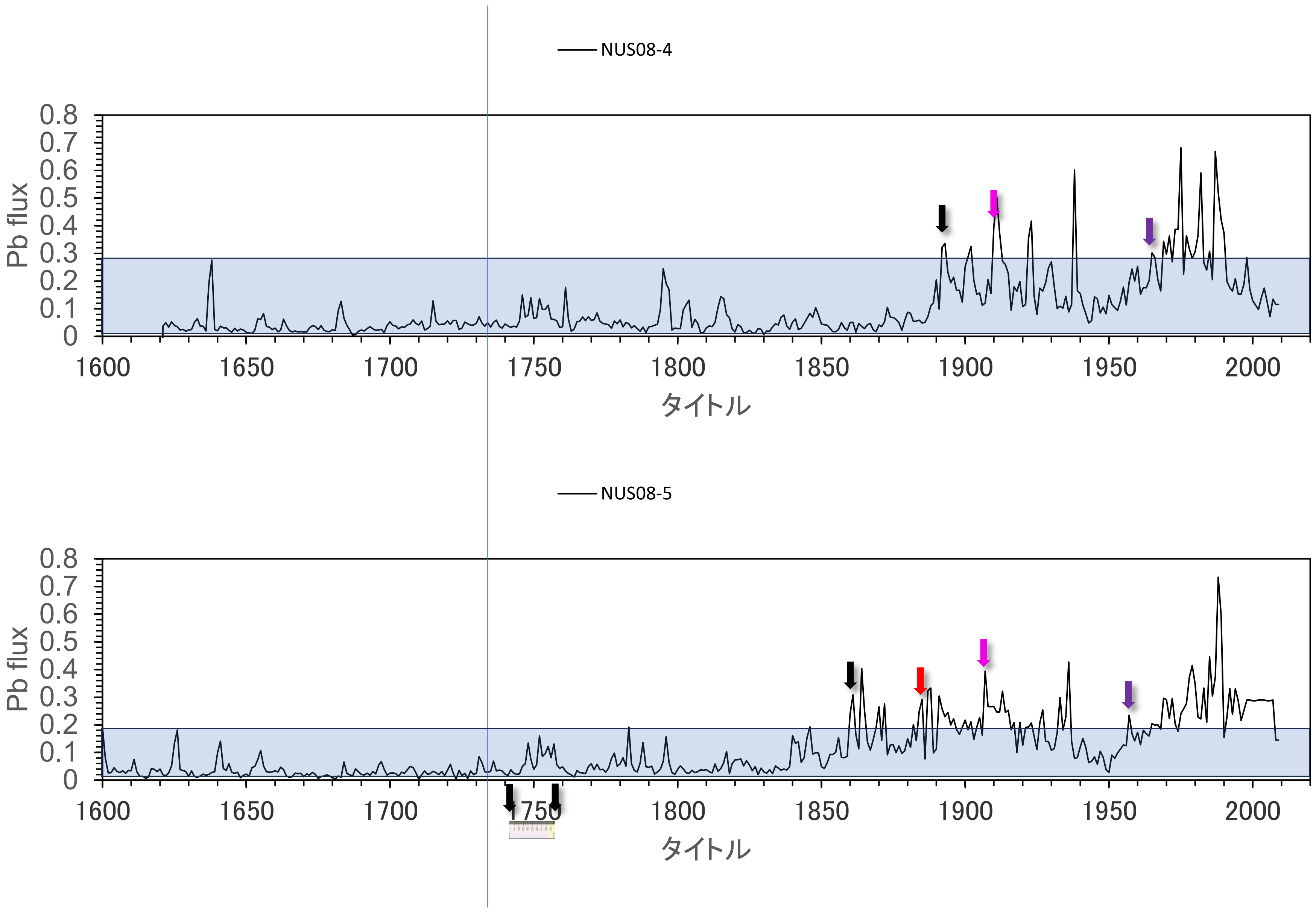

McConnell et al. (2014)

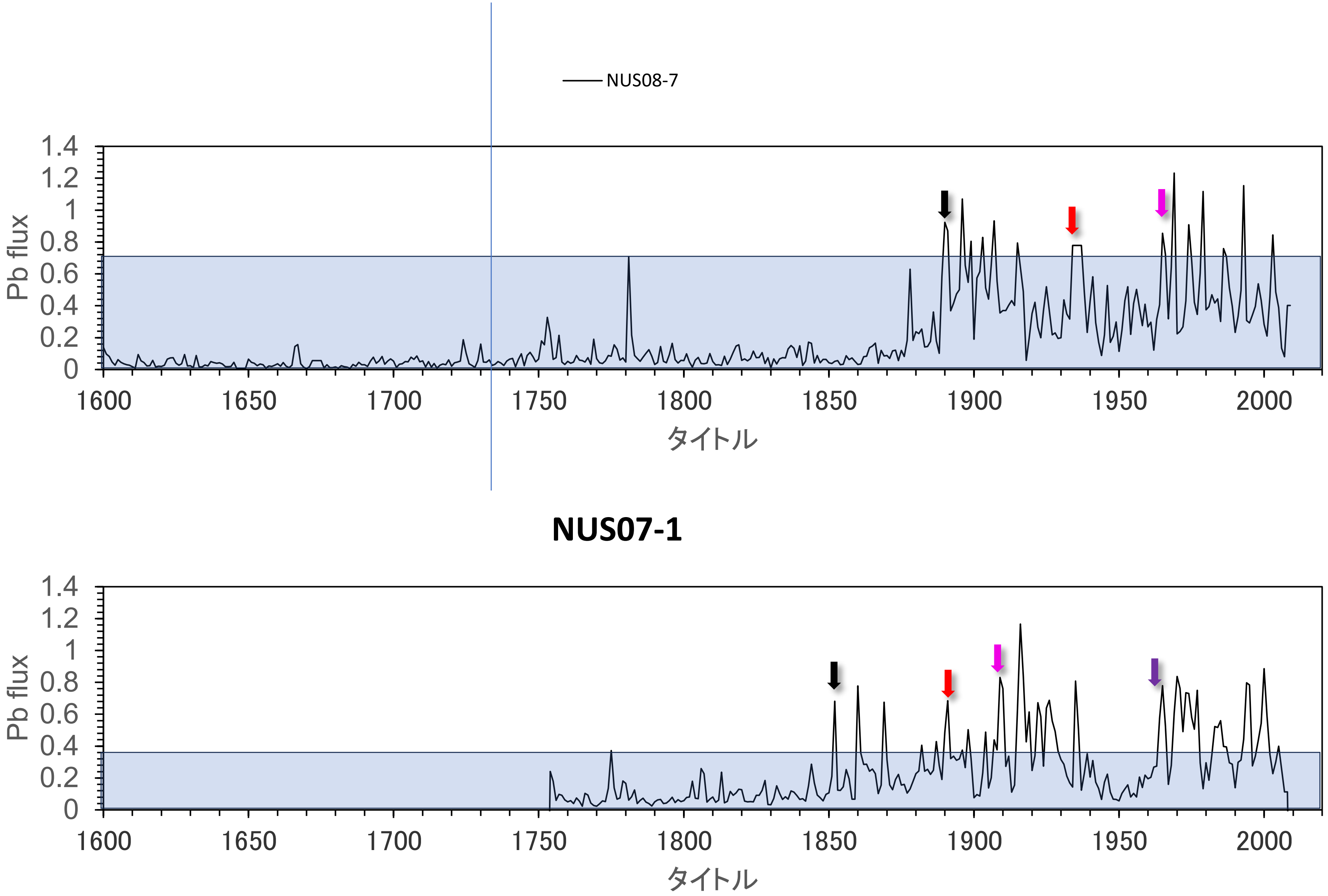

McConnell et al. (2014)

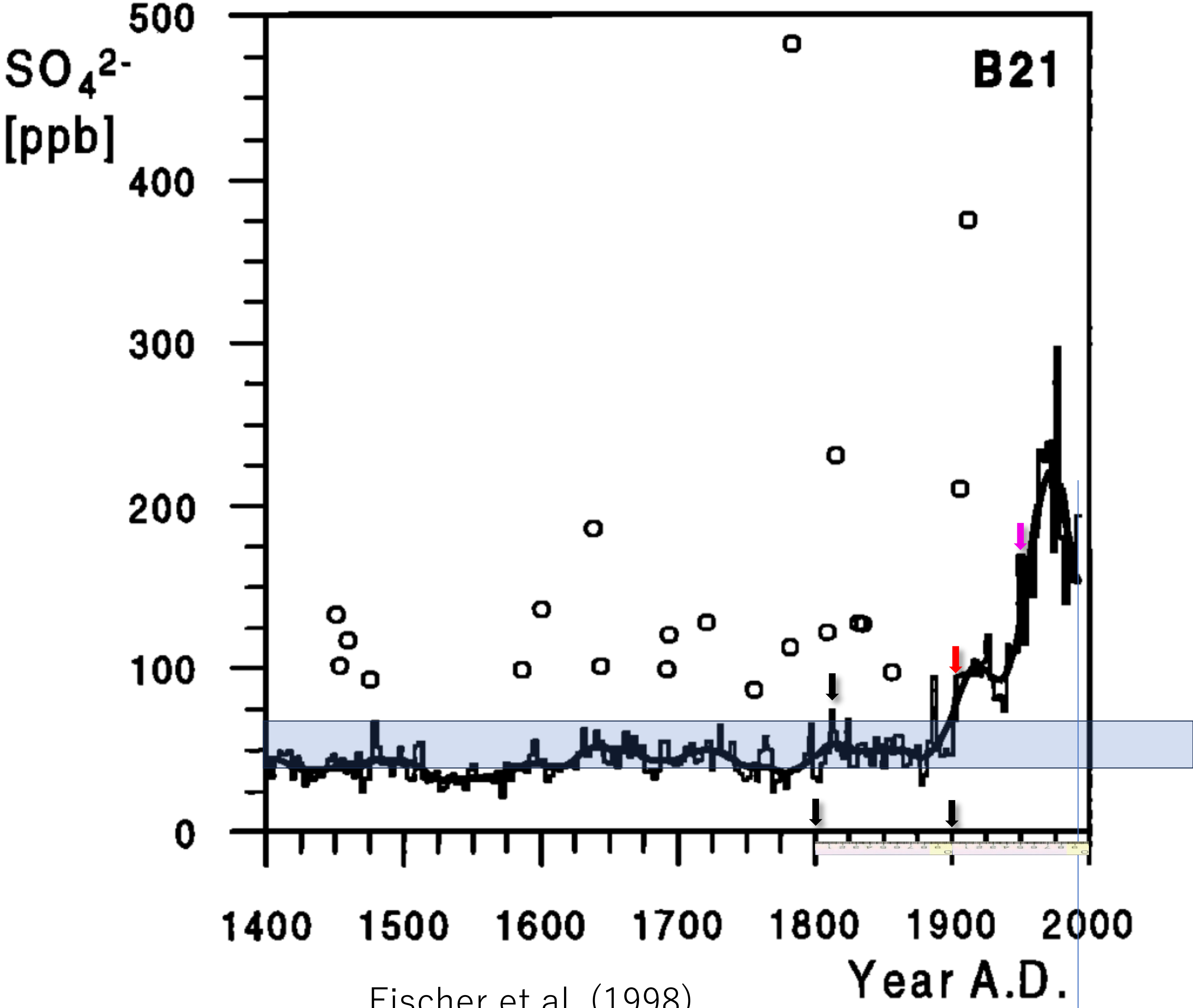

Fischer et al. (1998)

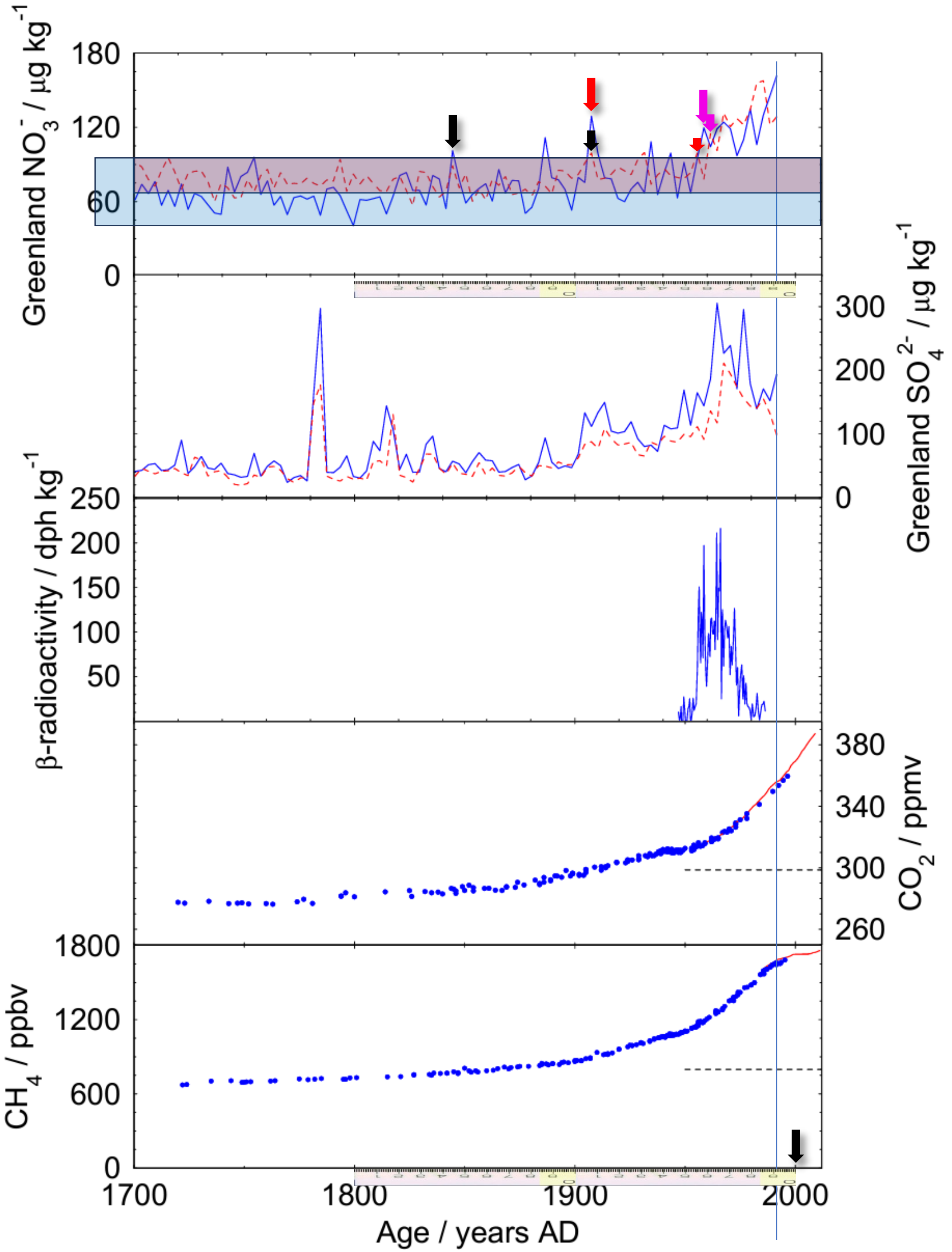

Greenland NO3- / µg kg-1
180
120
60
0
Greenland SO4 2- / µg kg-1
300
200
100
0
β-radioactivity / dph kg-1
250
200
150
100
50
CO2 / ppmv
380
340
300
260
CH4 / ppbv
1800
1200
600
0
1700
1800
1900
2000
Age / years AD

Wolff (2023)

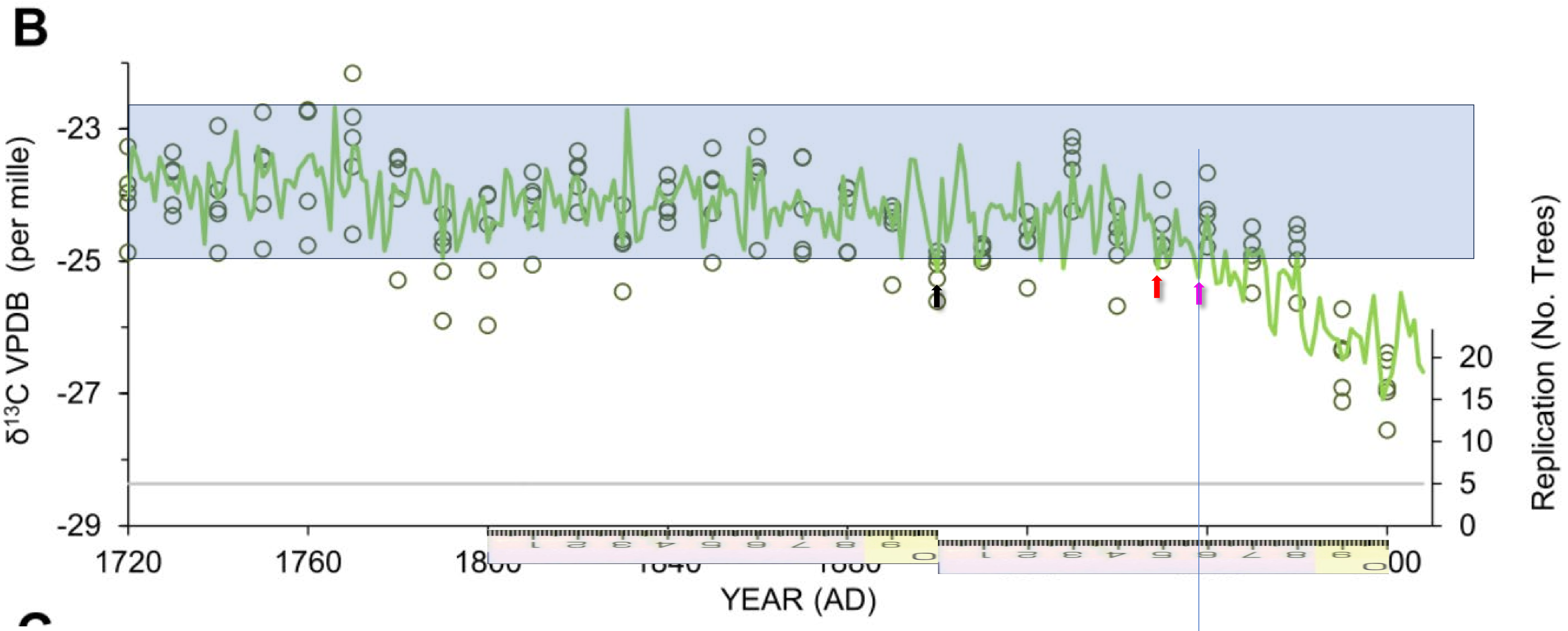

Loader et al. (2013)

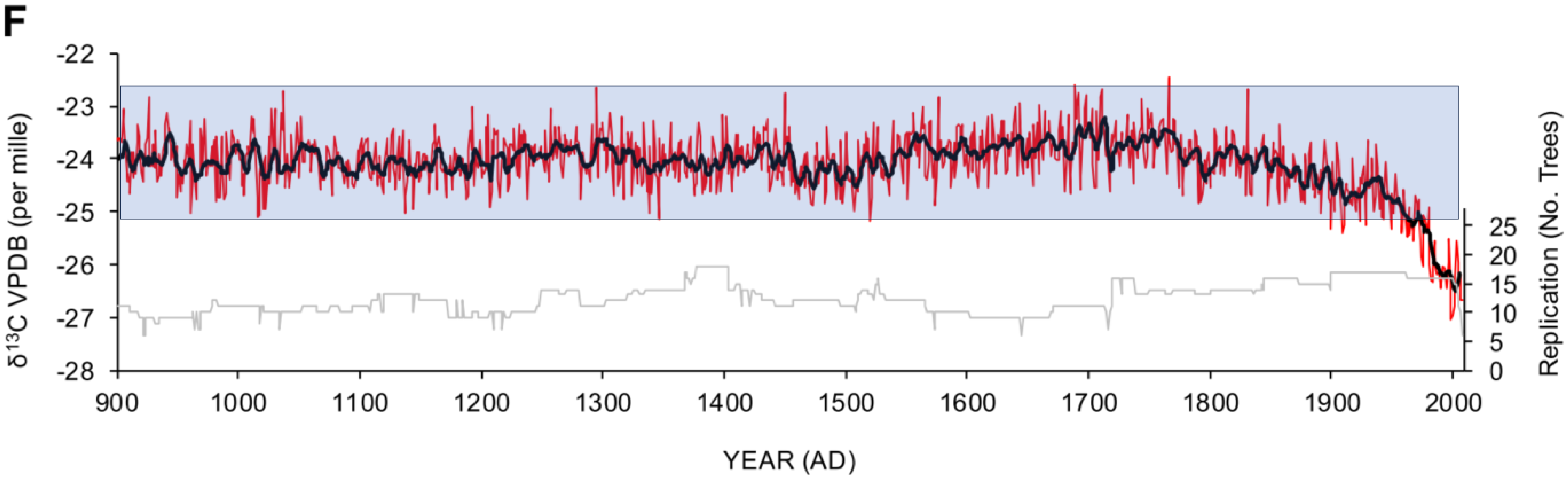

Loader et al. (2013)

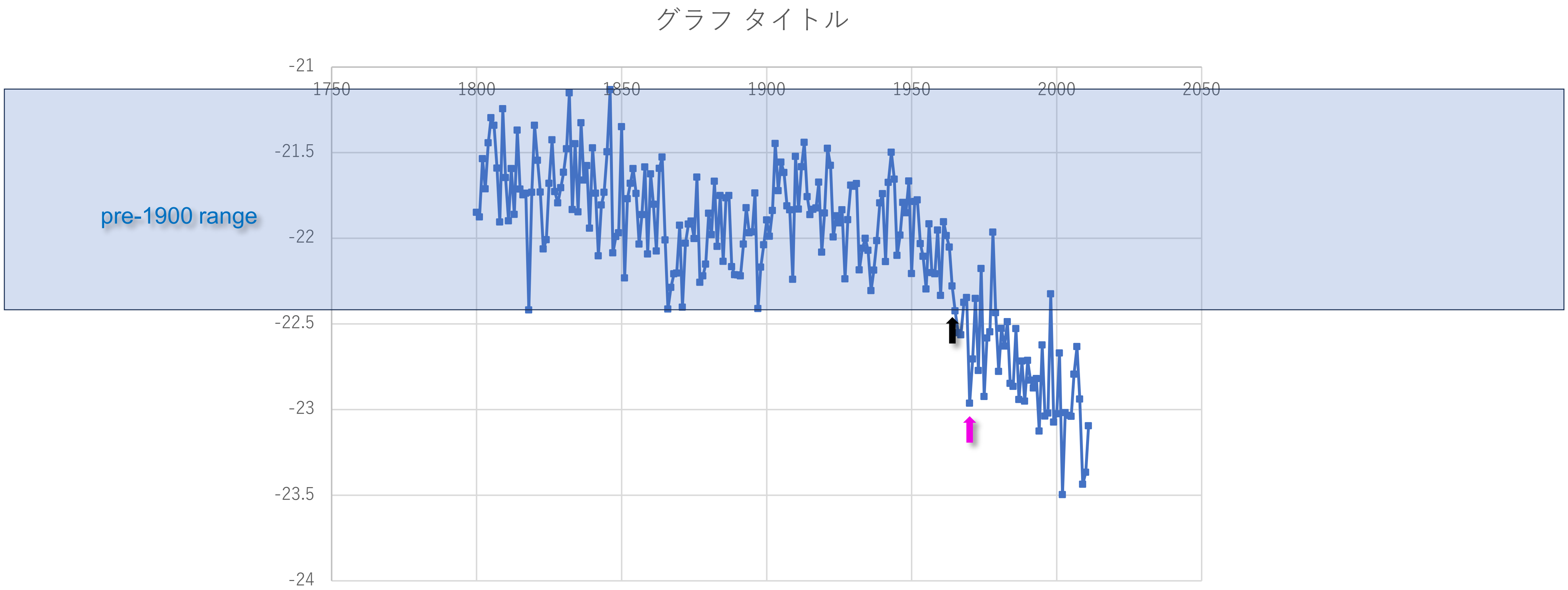

Lavergne et al., 2018

Table 1. Available global-scale databases of $\Delta^{14}C$ and $\delta^{13}C$ in atmospheric CO_2, terrestrial and ocean carbon, and fossil fuel emissions.

Name	Type	Website
Scripps Institution of Oceanography Global CO_2 Program	$\Delta^{14}C$ and $\delta^{13}C$ in CO_2	http://scrippsco2.ucsd.edu
NOAA Global Greenhouse Gas Reference Network	$\Delta^{14}C$ and $\delta^{13}C$ in CO_2	https://www.esrl.noaa.gov/gmd/dv/data/
World Data Centre for Greenhouse Gases (Including CSIRO data)	$\Delta^{14}C$ and $\delta^{13}C$ in CO_2	http://ds.data.jma.go.jp/gmd/wdcgg/
Heidelberg University data centre	$\Delta^{14}C$ in CO_2	https://heidata.uni-heidelberg.de/dataverse/carbon
Carbon Dioxide Information Analysis Center (CDIAC)	$\Delta^{14}C$ and $\delta^{13}C$ in CO_2, and $\delta^{13}C$ in fossil fuel CO_2 emissions	http://cdiac.ess-dive.lbl.gov/
GLobal Ocean Data Analysis Project GLODAP v2	$\Delta^{14}C$ and $\delta^{13}C$ in ocean dissolved inorganic carbon	https://www.nodc.noaa.gov/ocads/oceans/GLODAPv2/
TRY Plant Trait Database	$\delta^{13}C$ in terrestrial plants	https://www.try-db.org/TryWeb/Home.php
International Tree-Ring Data Bank	$\delta^{13}C$ in terrestrial plants	https://www.ncdc.noaa.gov/data-access/paleoclimatology-data/datasets/tree-ring
Soil Carbon Database	$\Delta^{14}C$ and $\delta^{13}C$ in soil carbon	https://github.com/powellcenter-soilcarbon

Graven et al., 2017

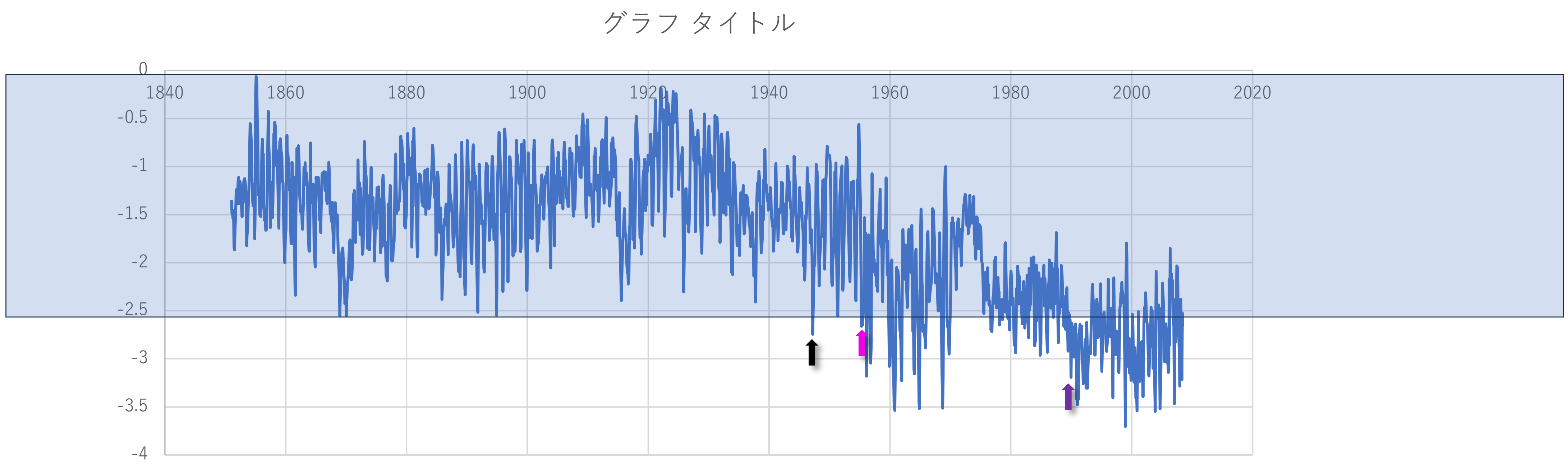

Han et al., 2019

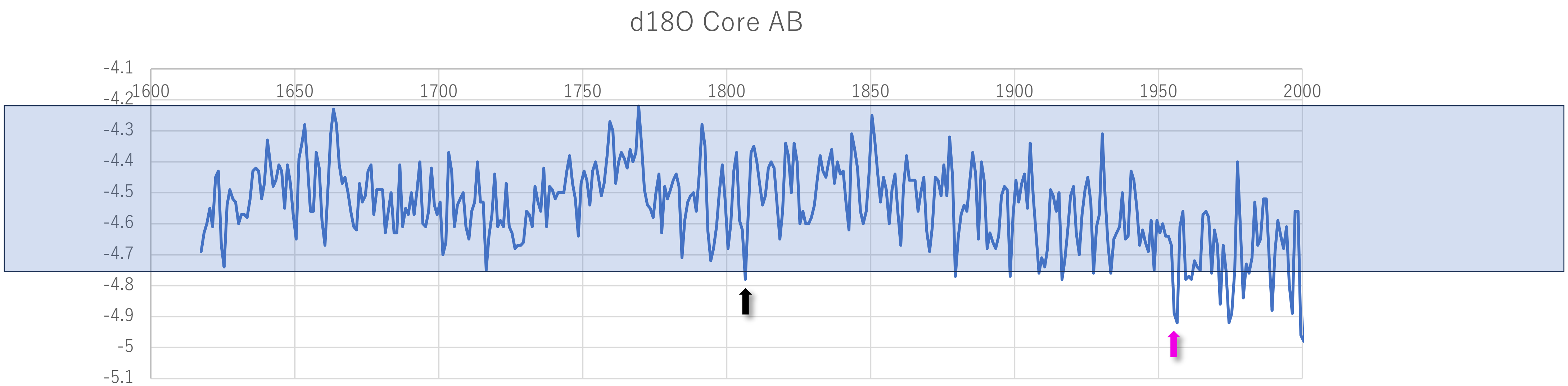

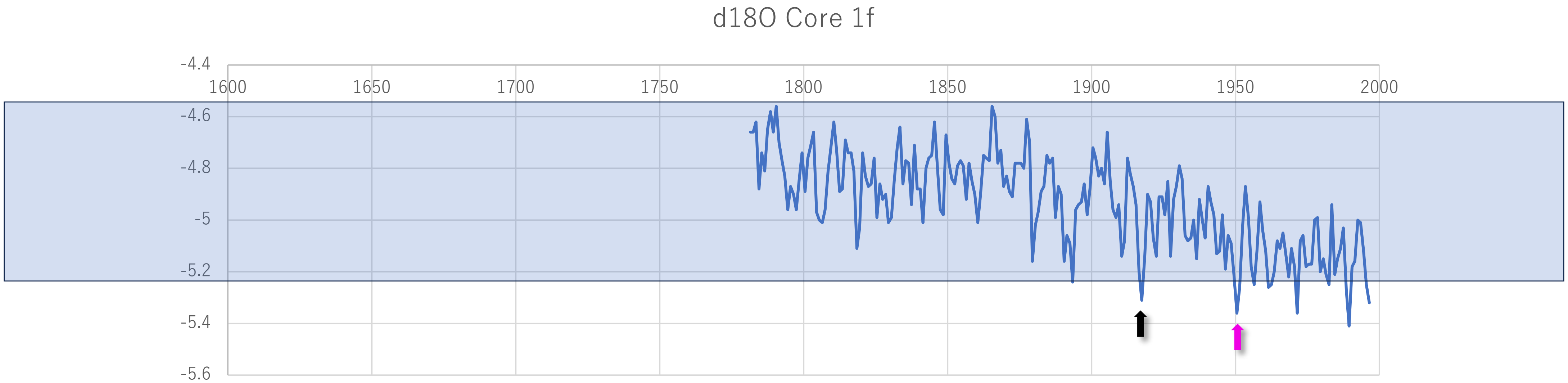

Linsley et al., 2006

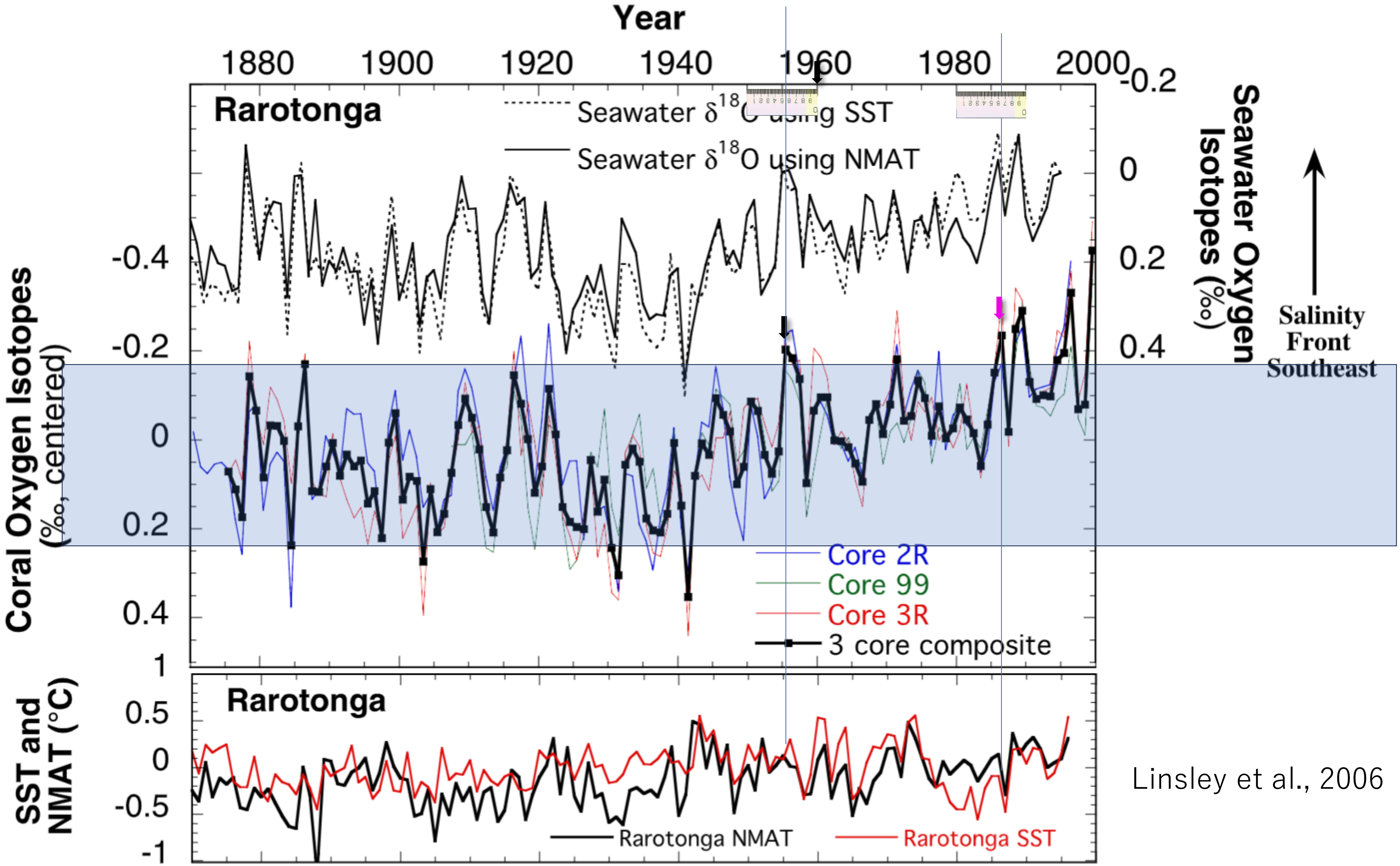

Linsley et al., 2006

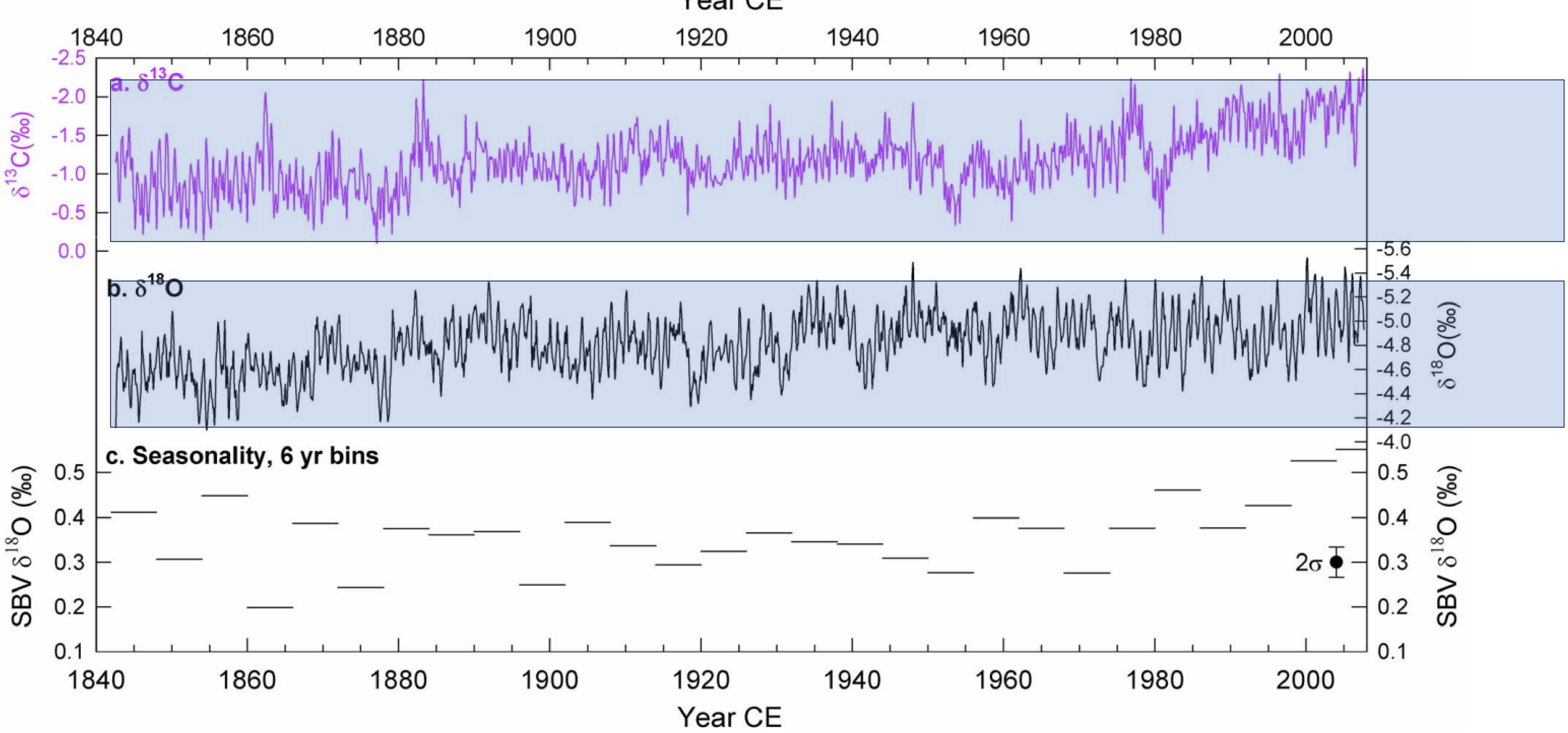

Figure 4. Stable isotopic time series from SBV coral from 1842 to 2007 CE (a) $\delta^{13}C$ (purple), (b) $\delta^{18}O$ (black). There is a strong annual cycle in both time series, as well as a trend of −0.41 ± 0.16 ‰ for coral $\delta^{18}O$ and −0.74 ± 0.41‰ for $\delta^{13}C$ toward depletion in the raw time series. (c) The annual cycles (calculated as the range of each 6-year bin) shows periods of high variability (~1840–1910, 1960–2007 CE) and periods of low variability (~1910–1960). The average annual cycles for each 6-year bin can be seen in Figure S4.

Gorman et al. (2012)

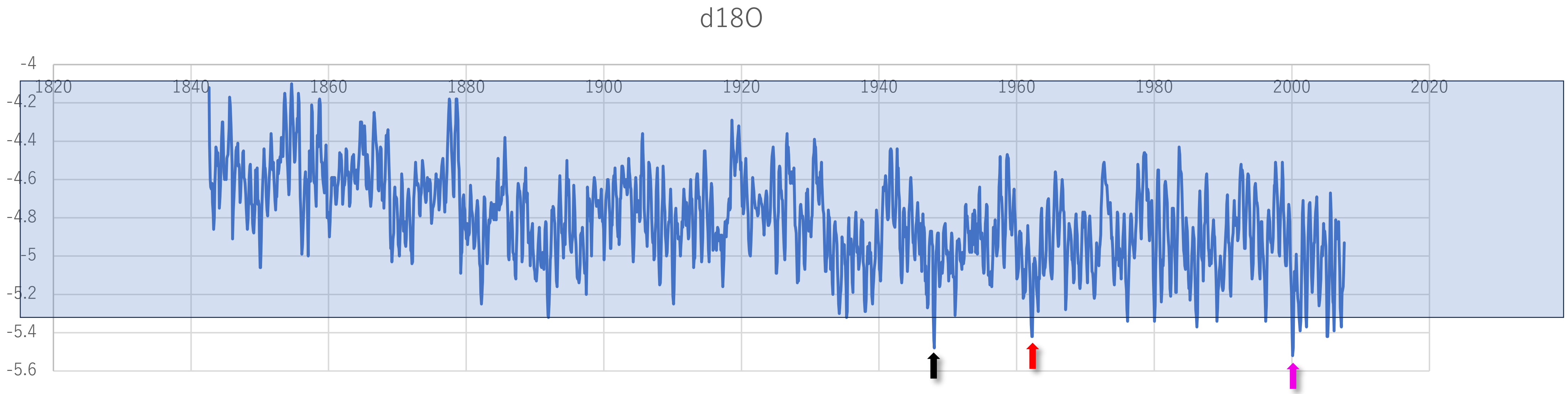

Gorman et al. (2012)

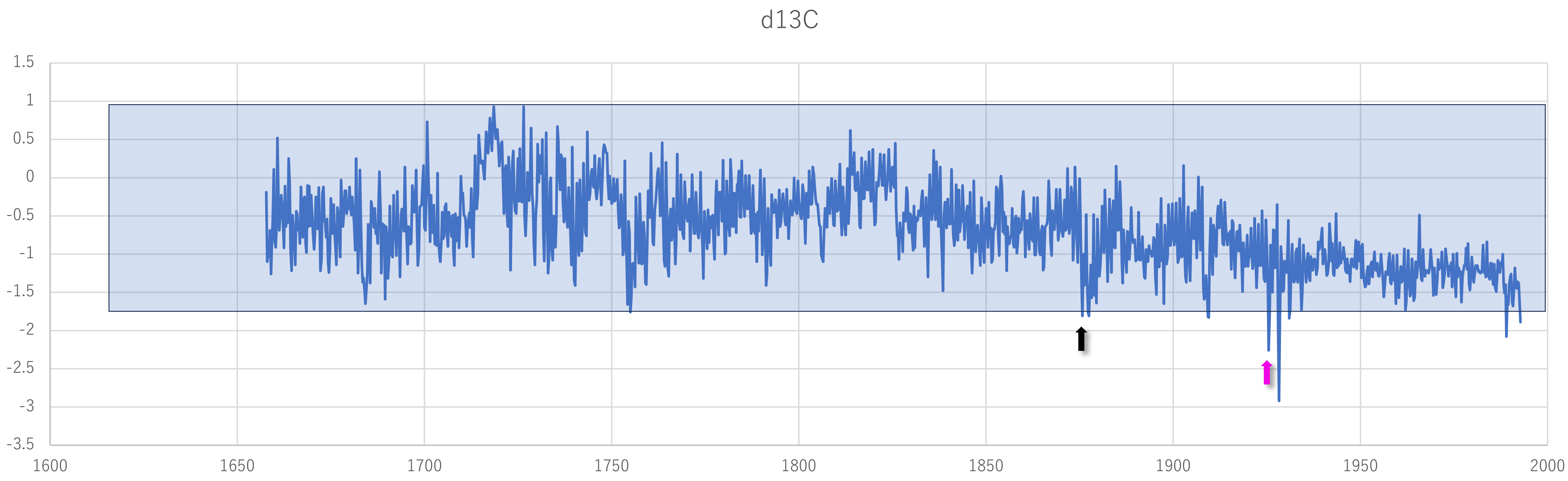

DeLong et al., 2012

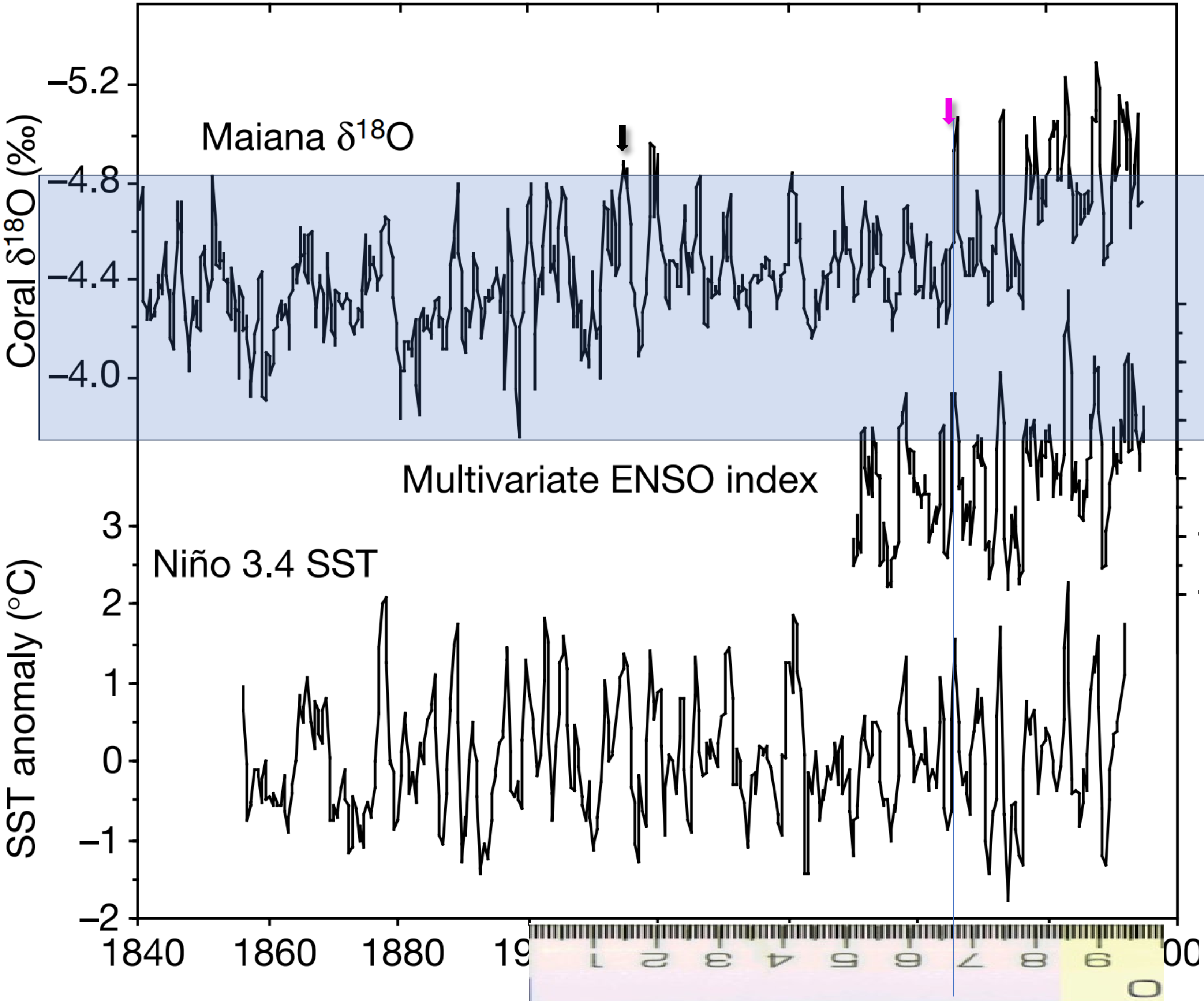

Urban et al., 2000

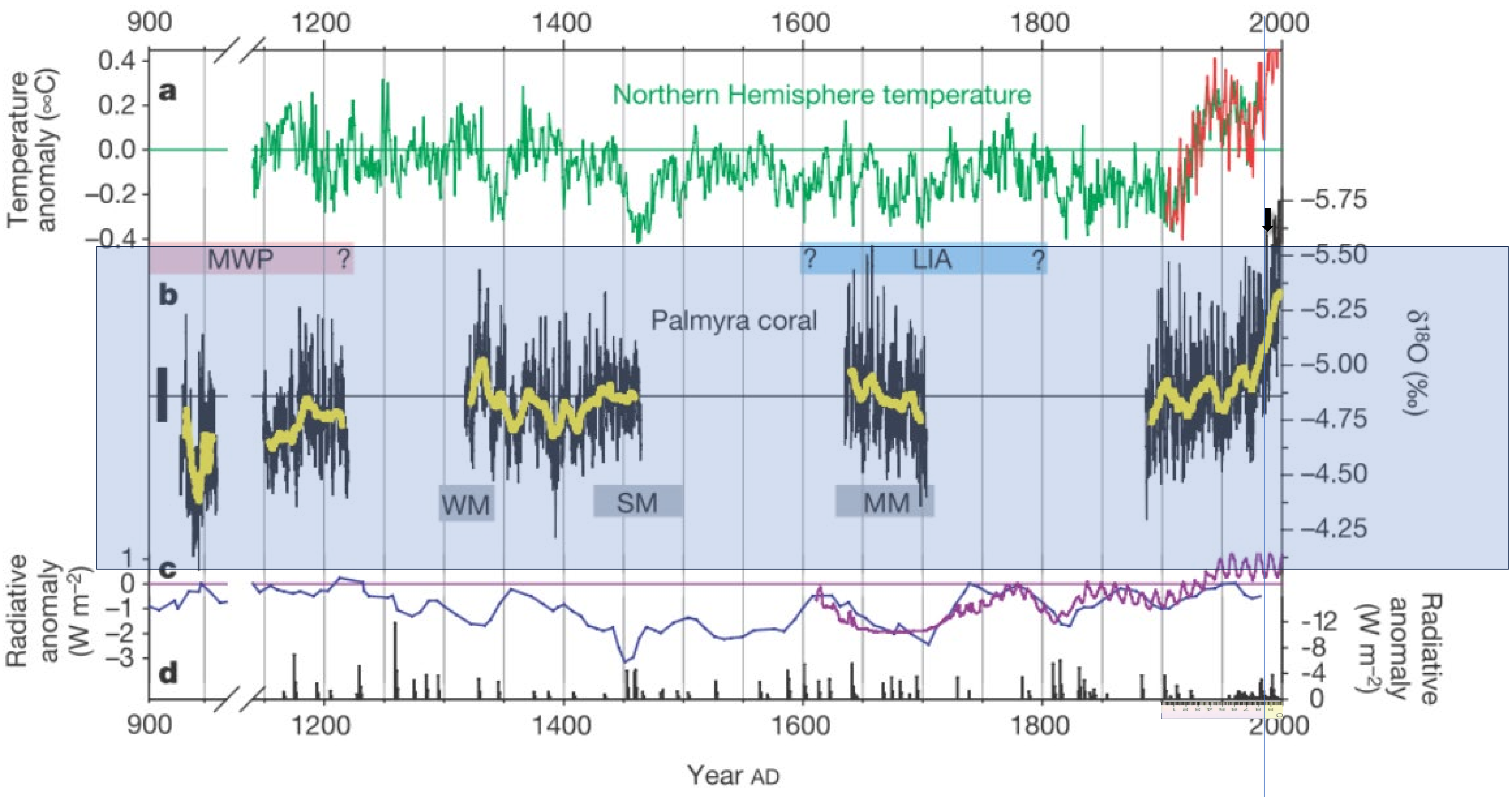

Cobb et al., 2003

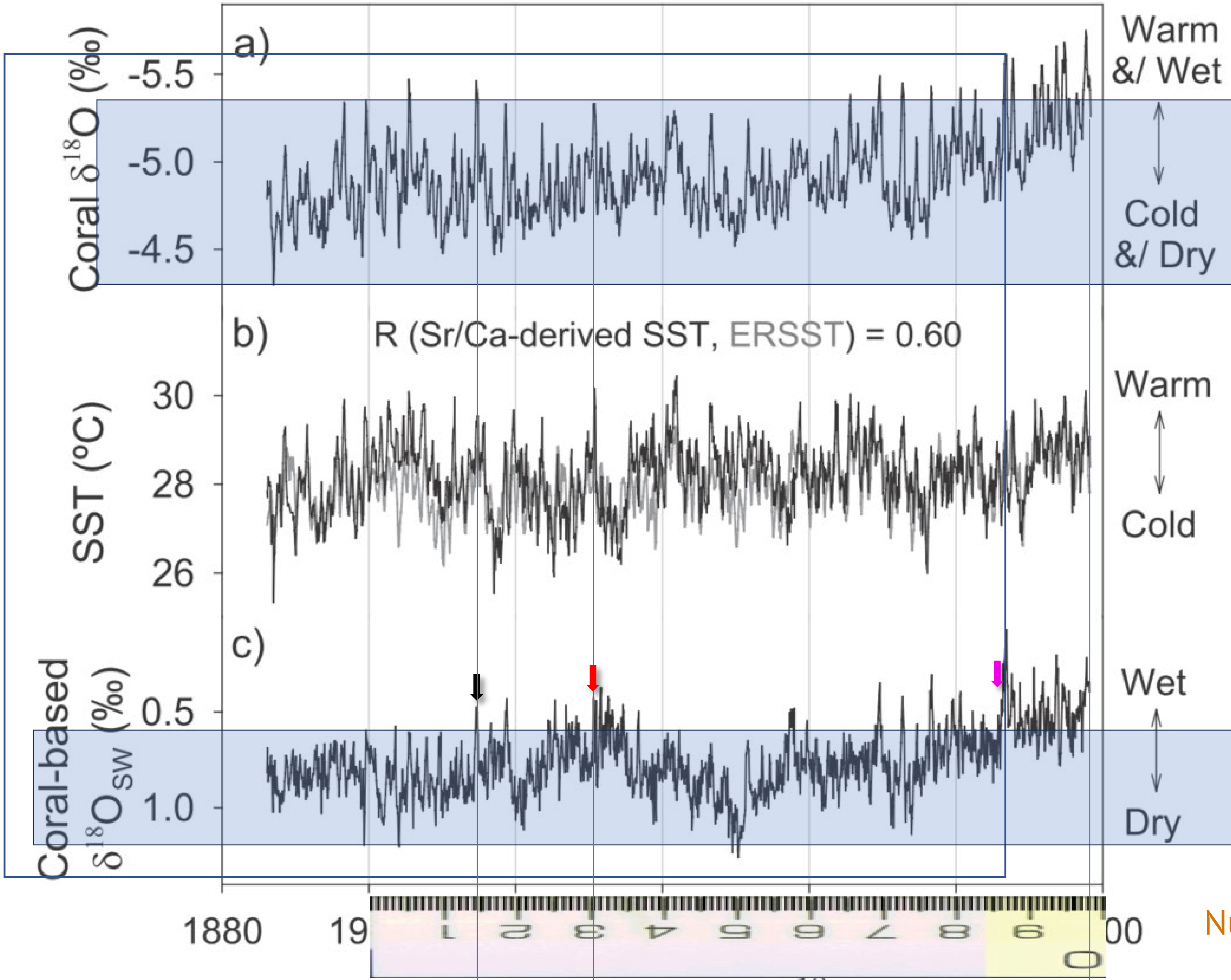

[illegible]

Nurhati et al., 2011

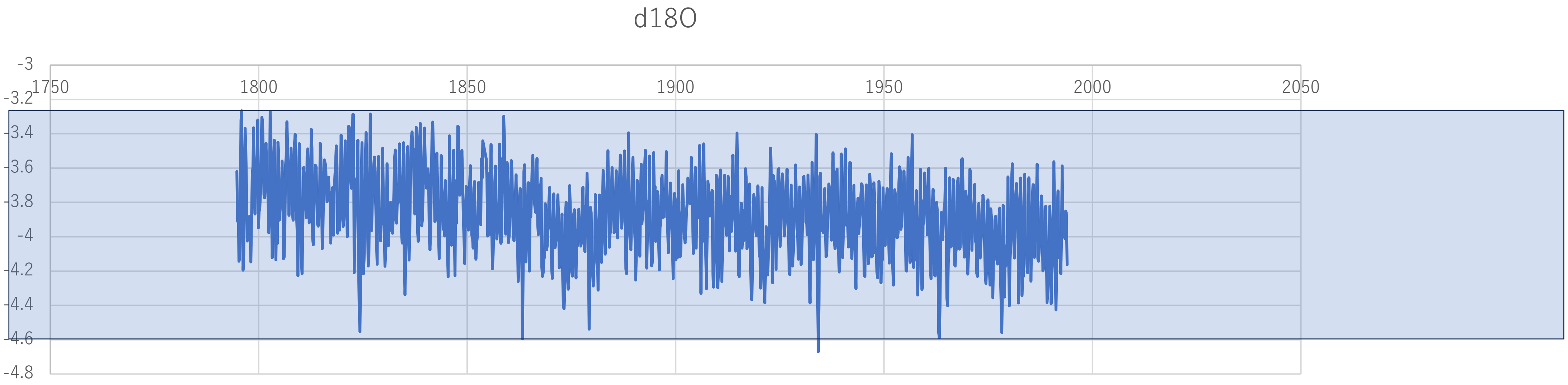

Kuhnert et al., 1999

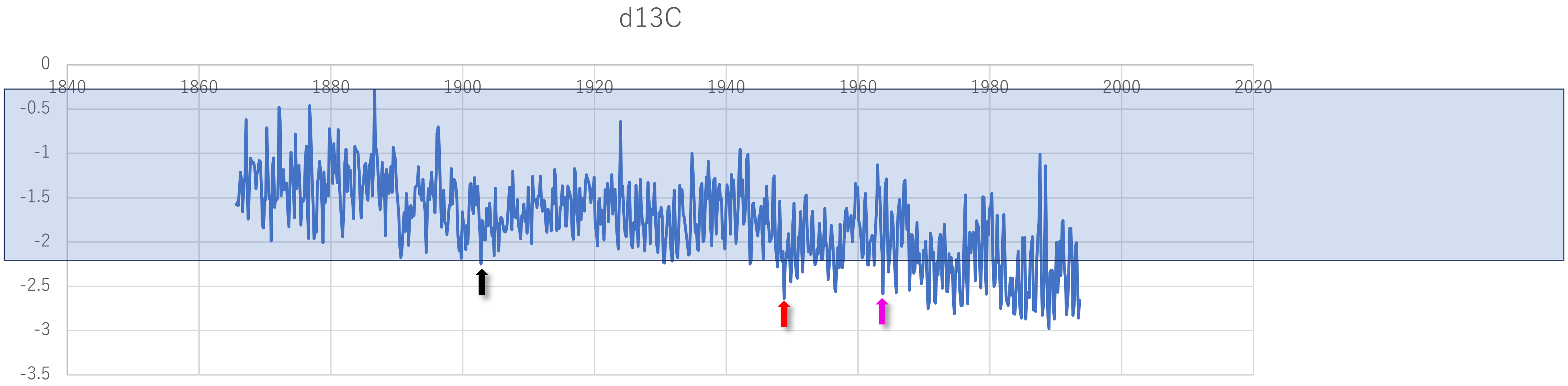

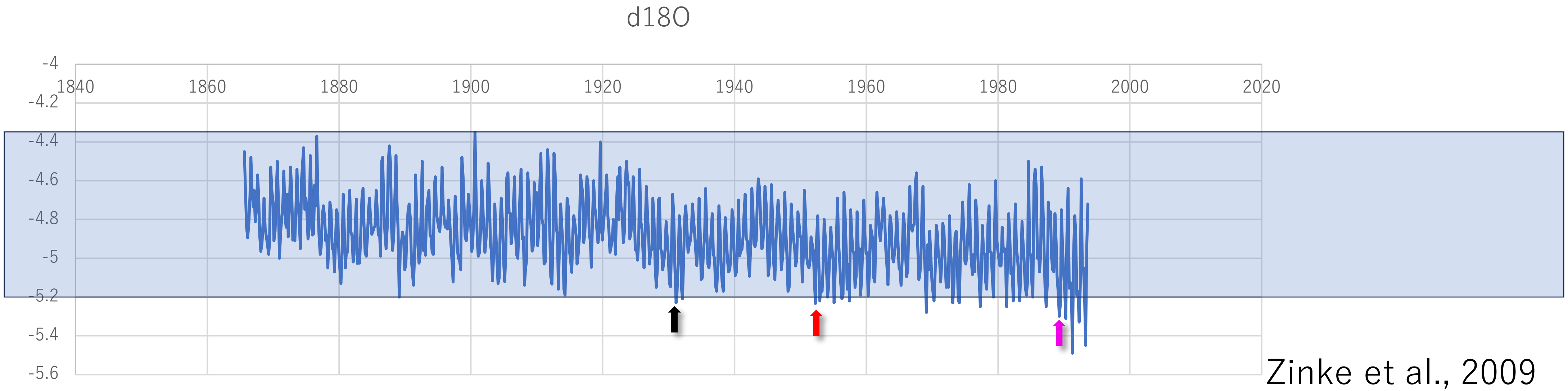

Zinke et al., 2009

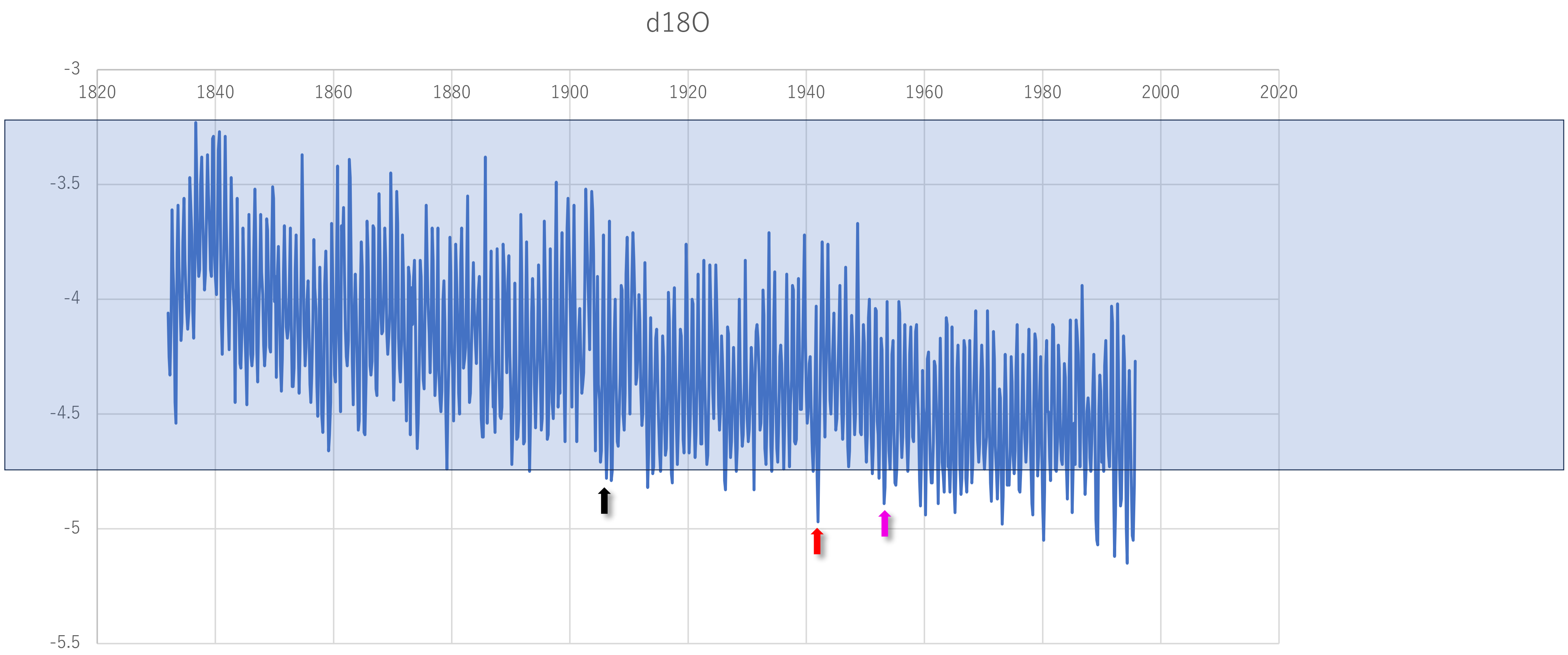

Pfeiffer et al., 2004

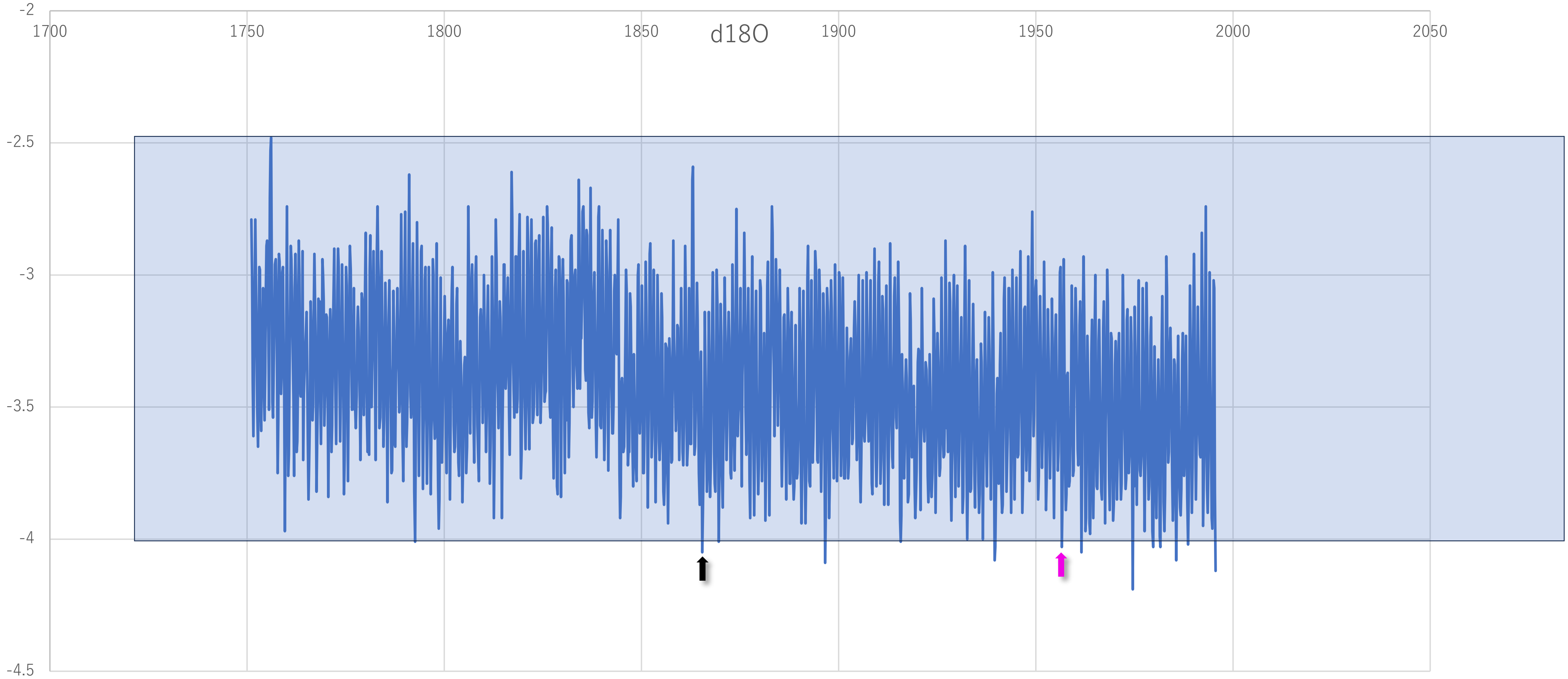

Felis et al., 2000

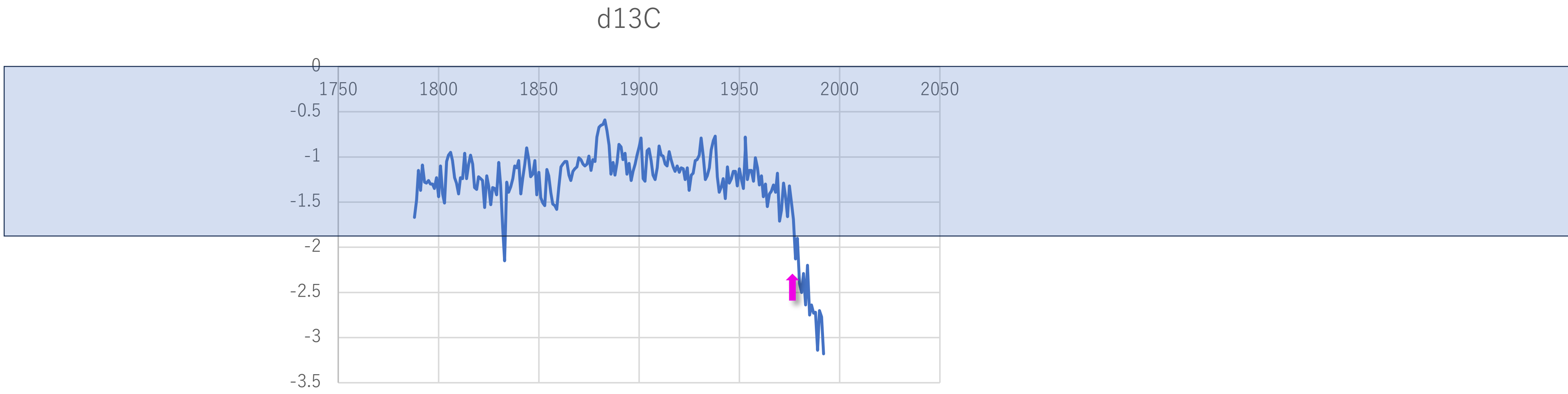

Heiss et al/. 1994

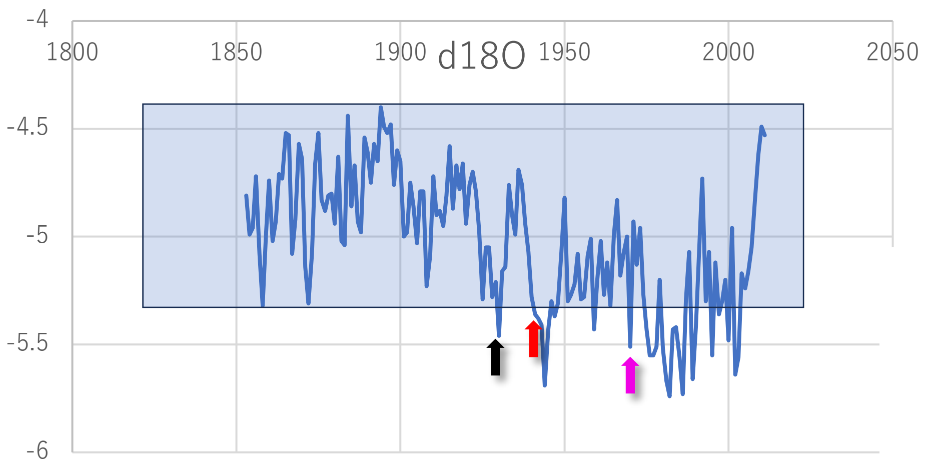

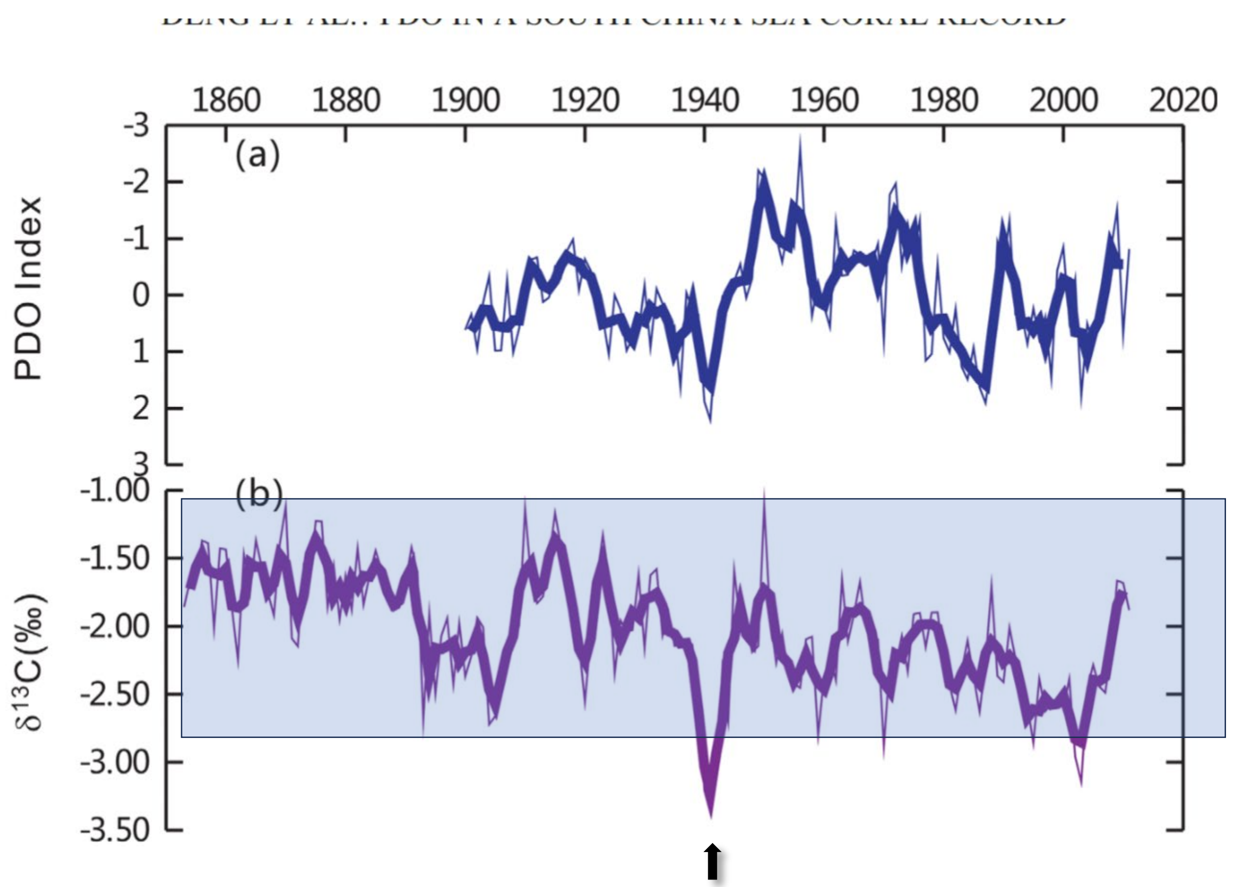

Deng et al., 2013

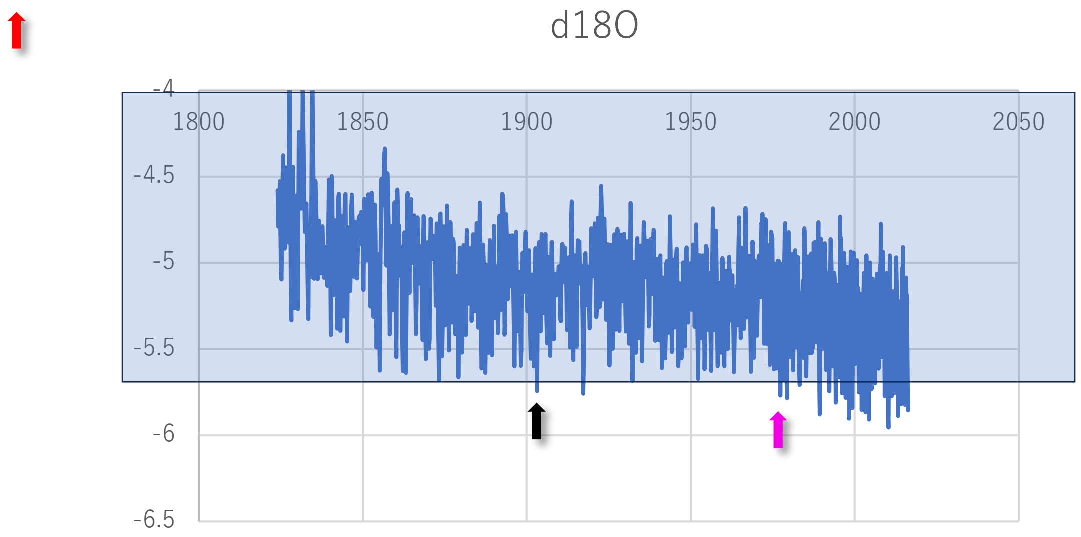

Murty et al., 2018

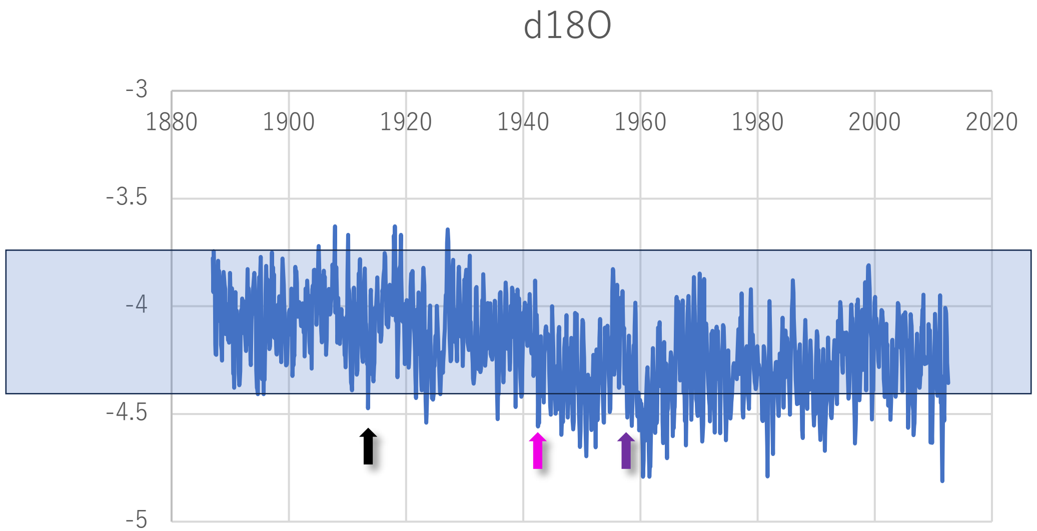

von Reumont et al., 2016

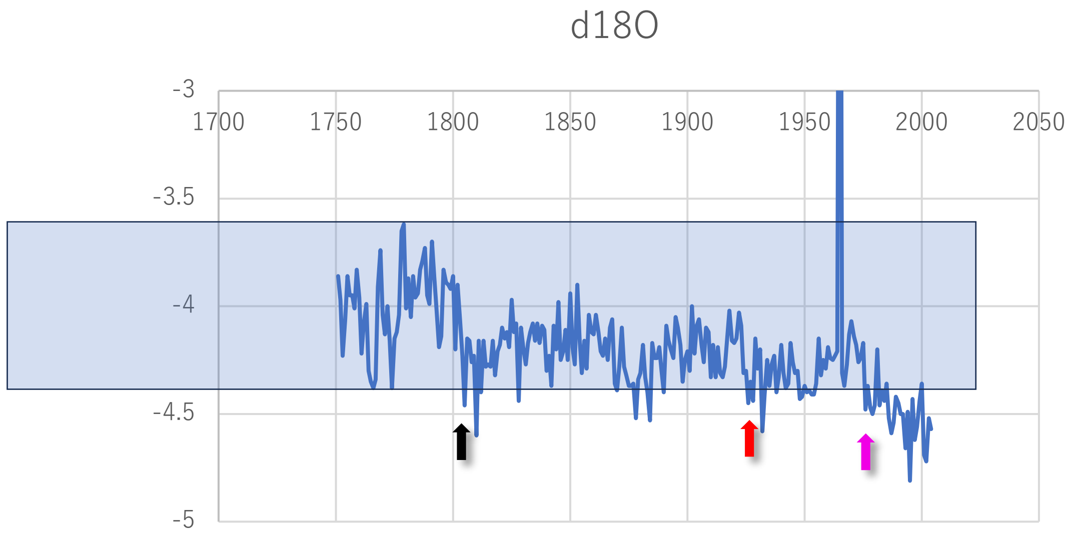

Kilbourne et al., 2008

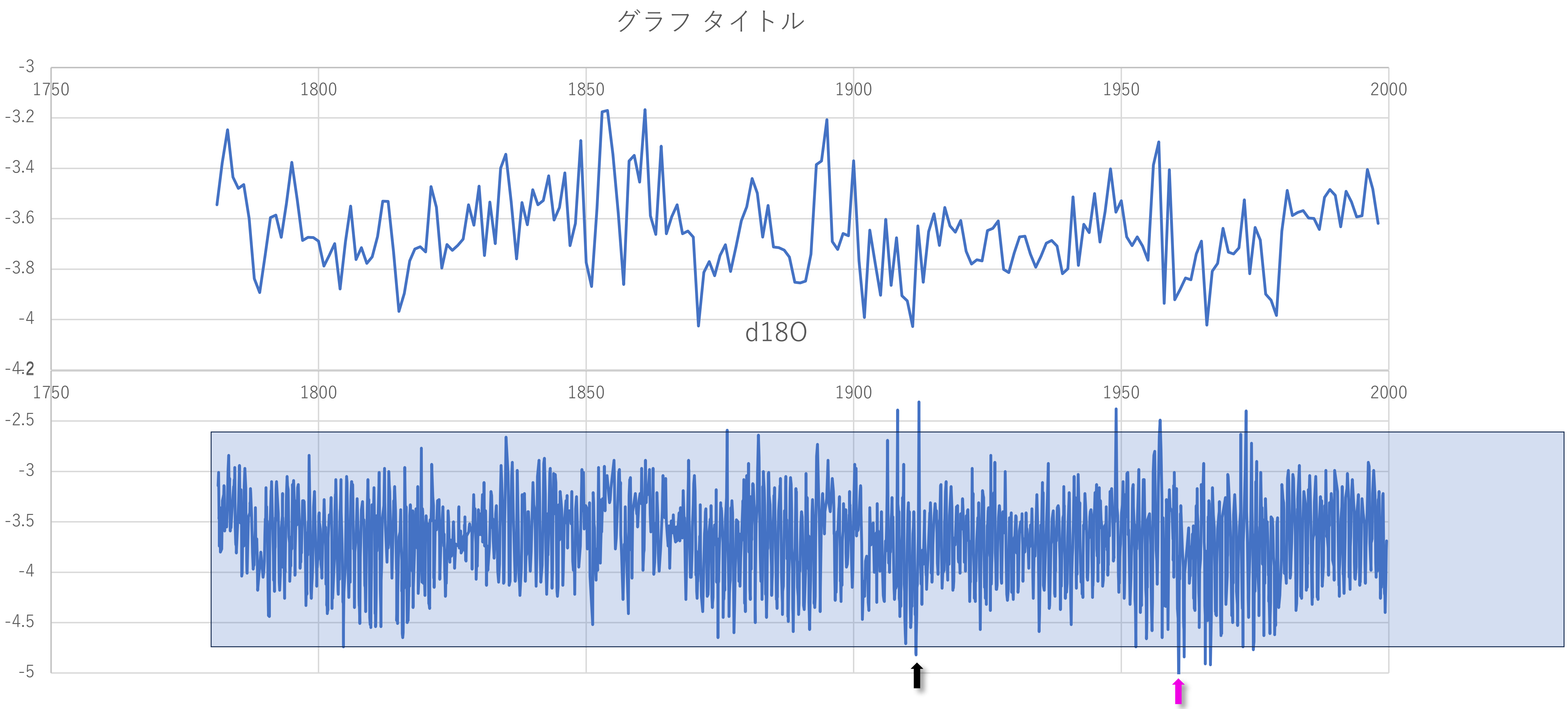

Goodkin et al., 2007

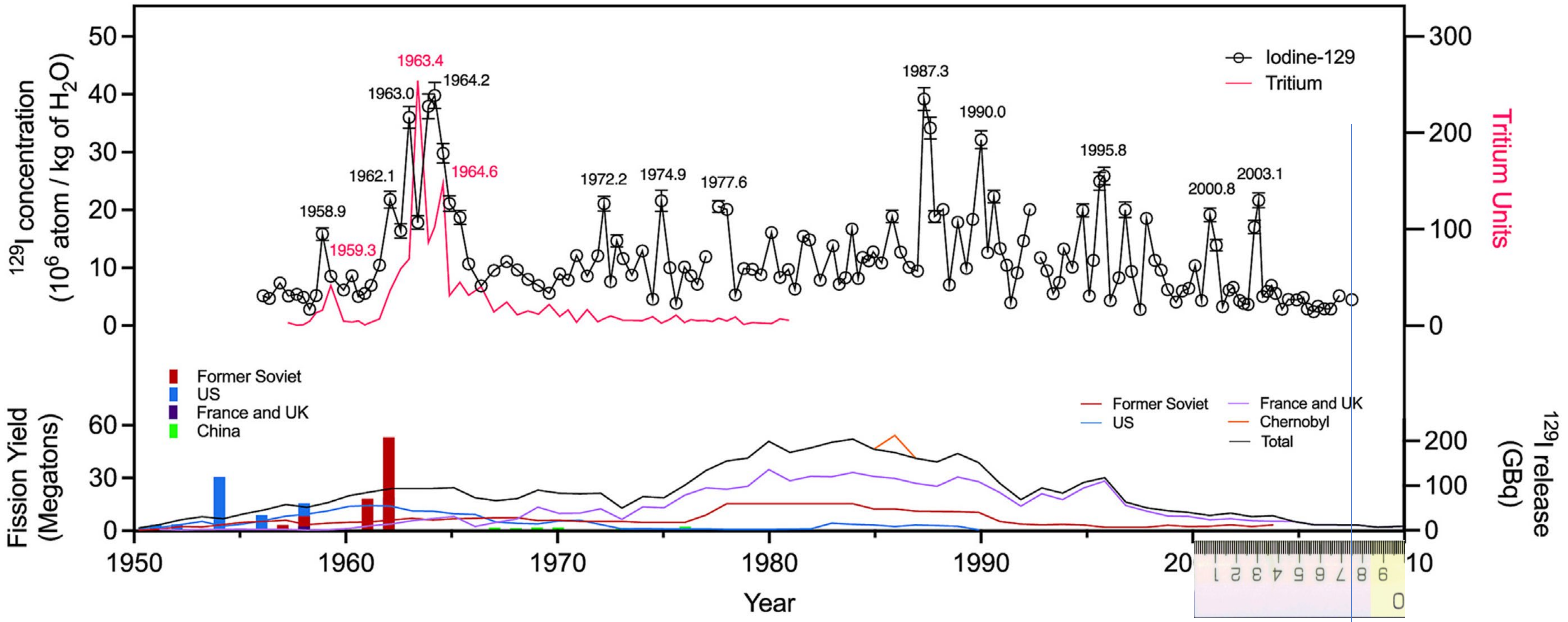

Fig. 2. Iodine-129 in the SE-Dome ice core. Concentrations of ^{129}I (10^6 atoms/kg) and ^{3}H (tritium units or $^3H/^1H$ ratio $\times 10^{-18}$; Iizuka et al., 2017) in the SE-Dome ice core; Fission yield of aboveground nuclear weapons testing or ANWT (megatons; UNSCEAR, 2000) and airborne emissions of ^{129}I from nuclear fuel reprocessing facilities or NFR and the Chernobyl Accident (GBq; Aldahan et al., 2007; Hou et al., 2013; Reithmeier et al., 2010, 2006).

Bautista et al., 2023

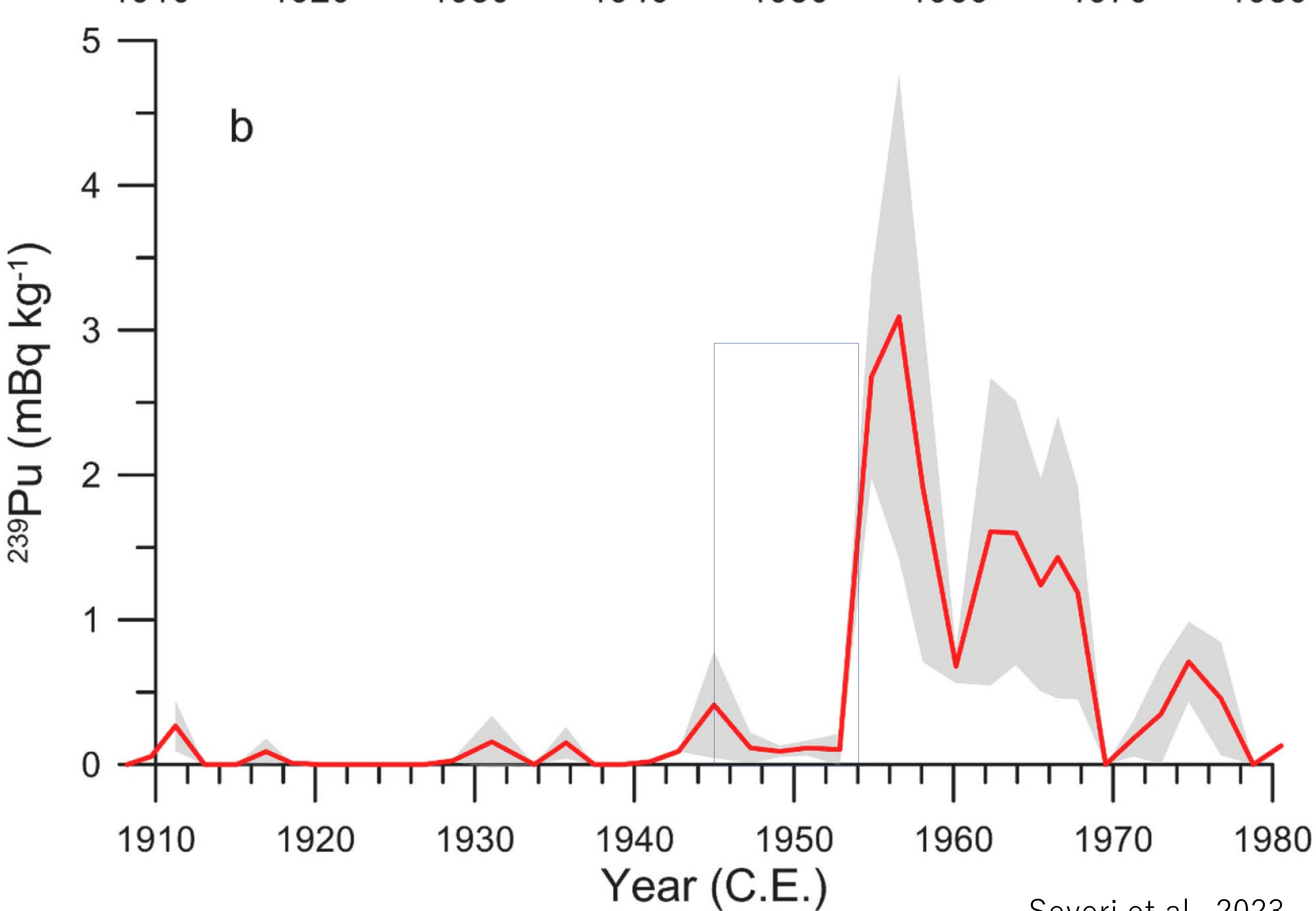

Severi et al., 2023

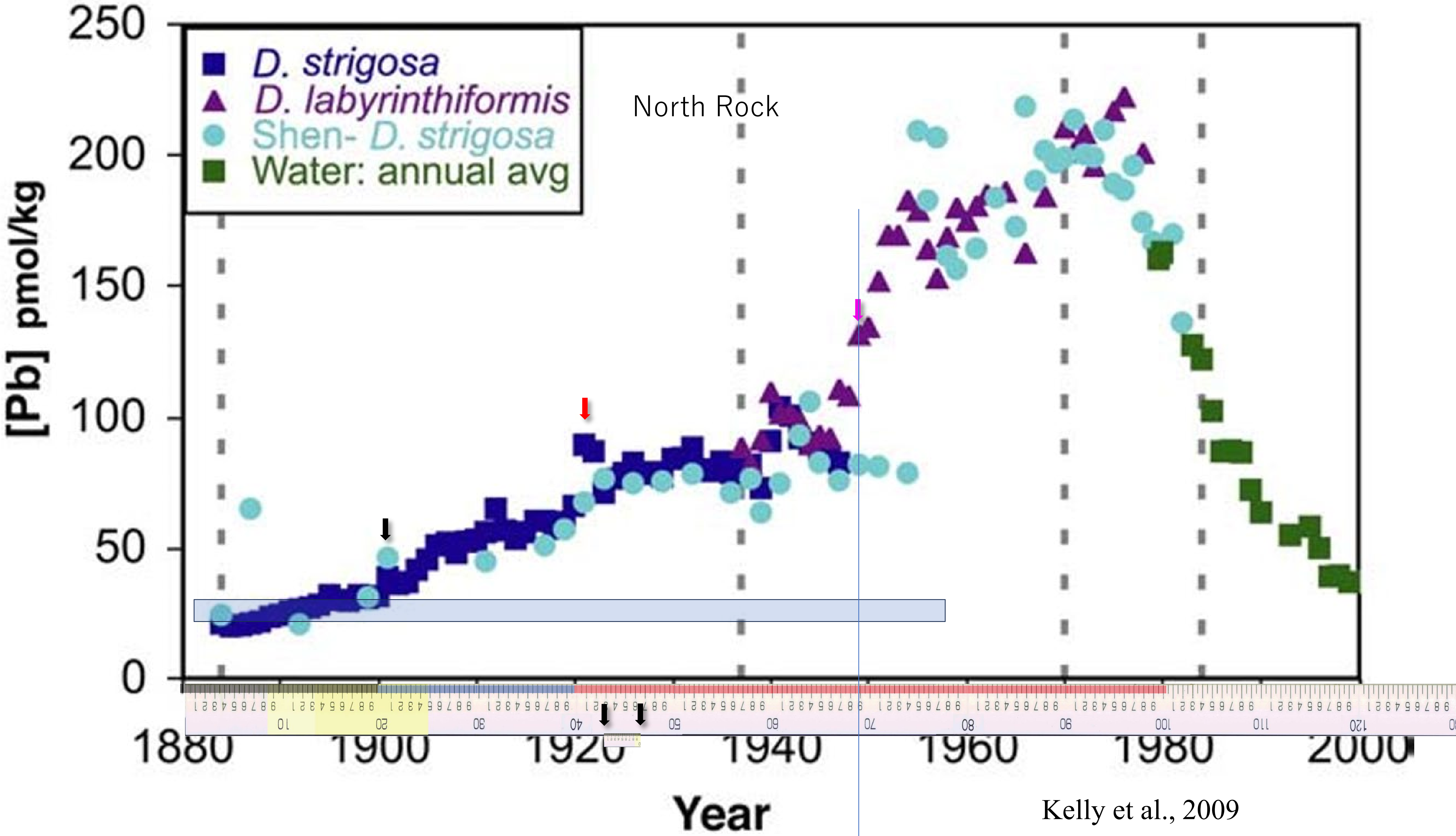

Kelly et al., 2009

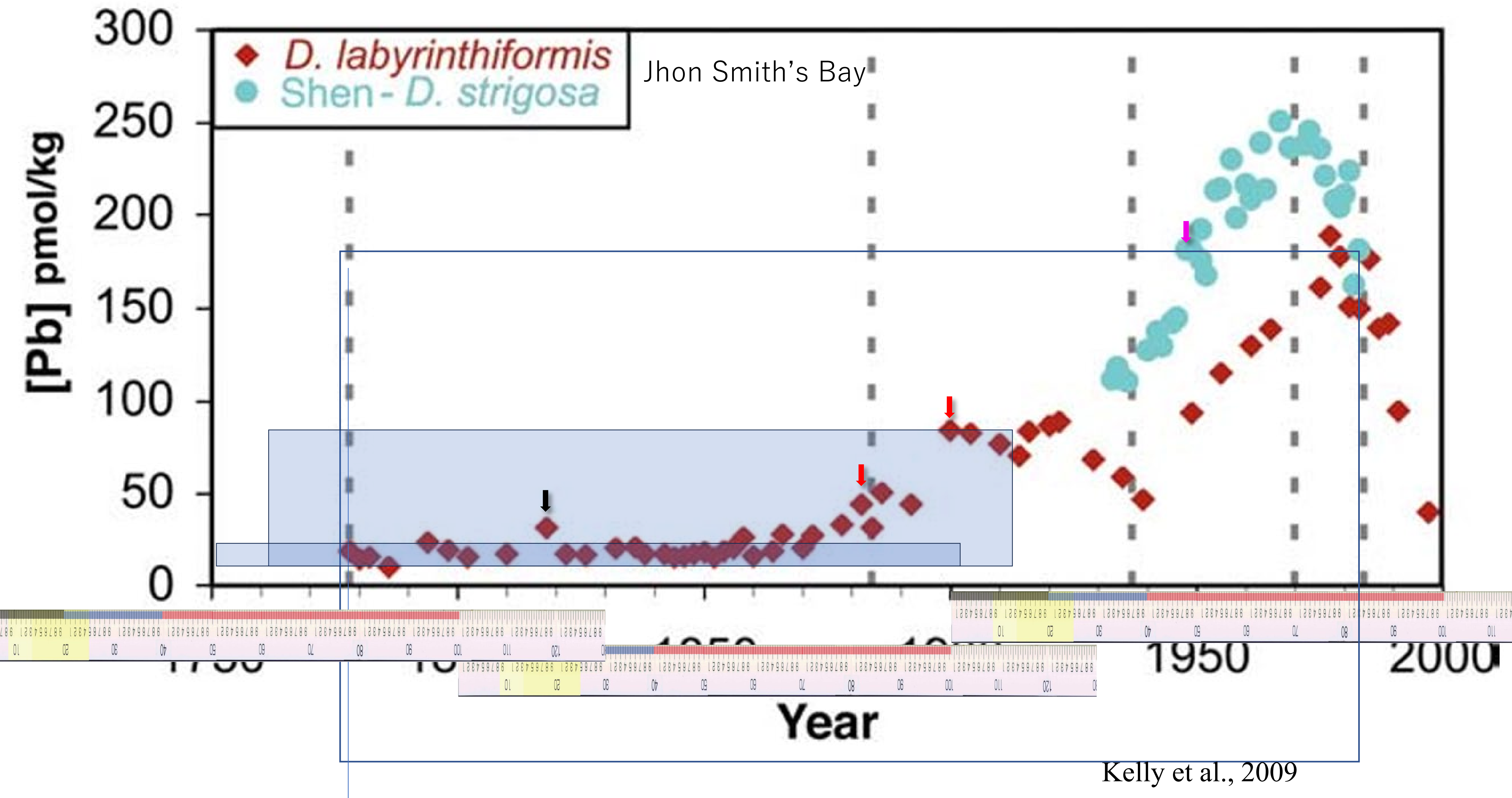

Kelly et al., 2009

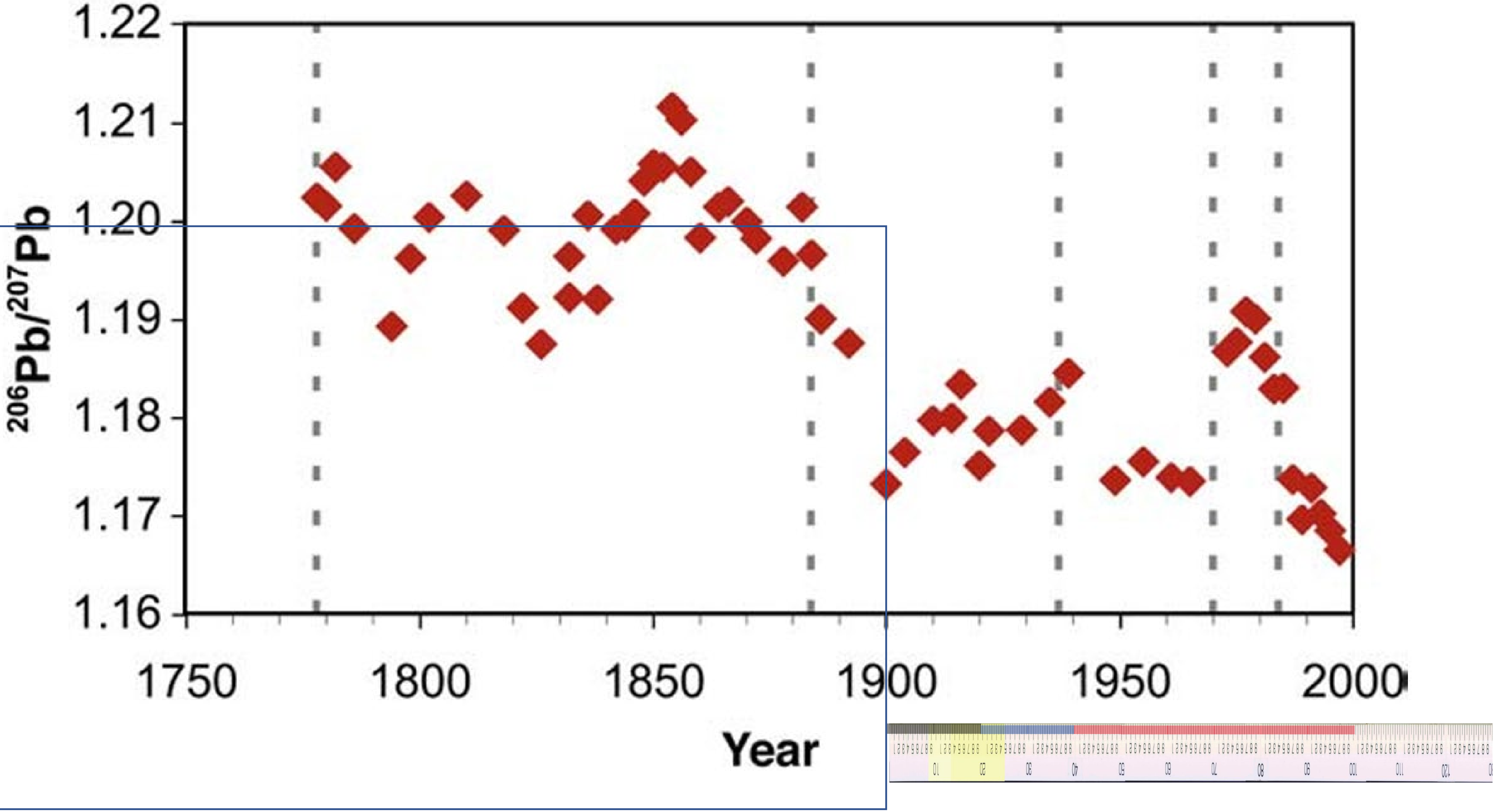

Fig. 5. $^{206}Pb/^{207}Pb$ ratios of Bermudan coral from John Smith's Bay from the late 1700s to 2000.

Kelly et al., 2009

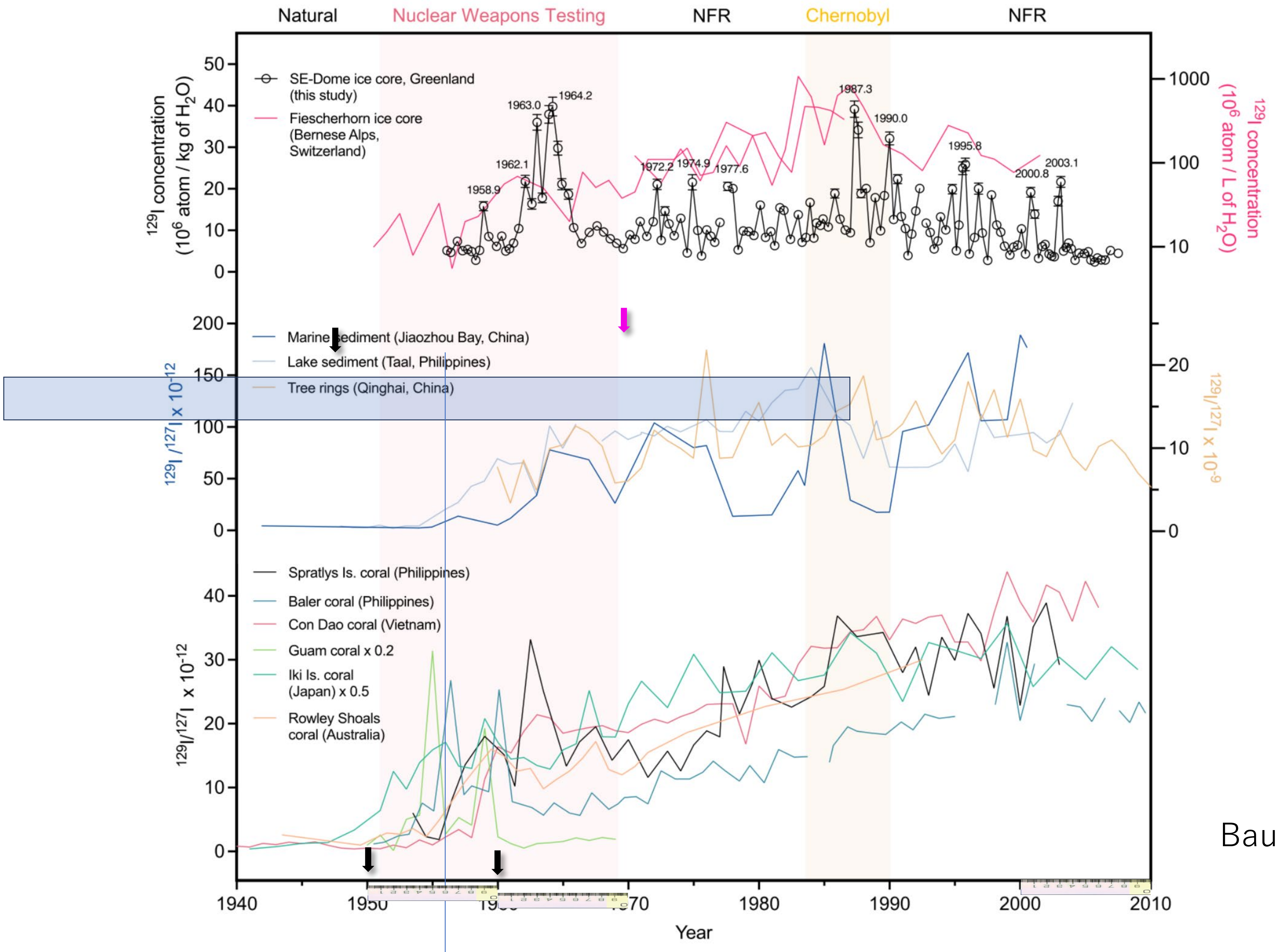

Bautista et al., 2023

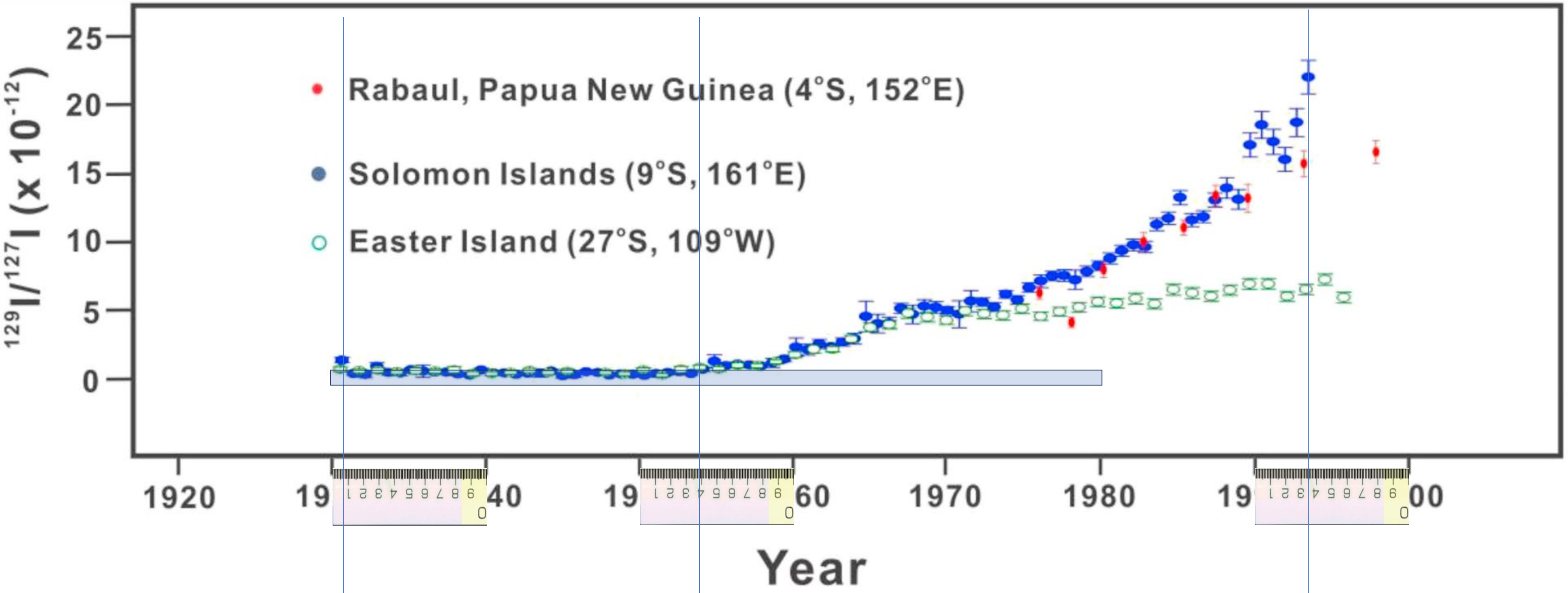

Chang, et al., 2016

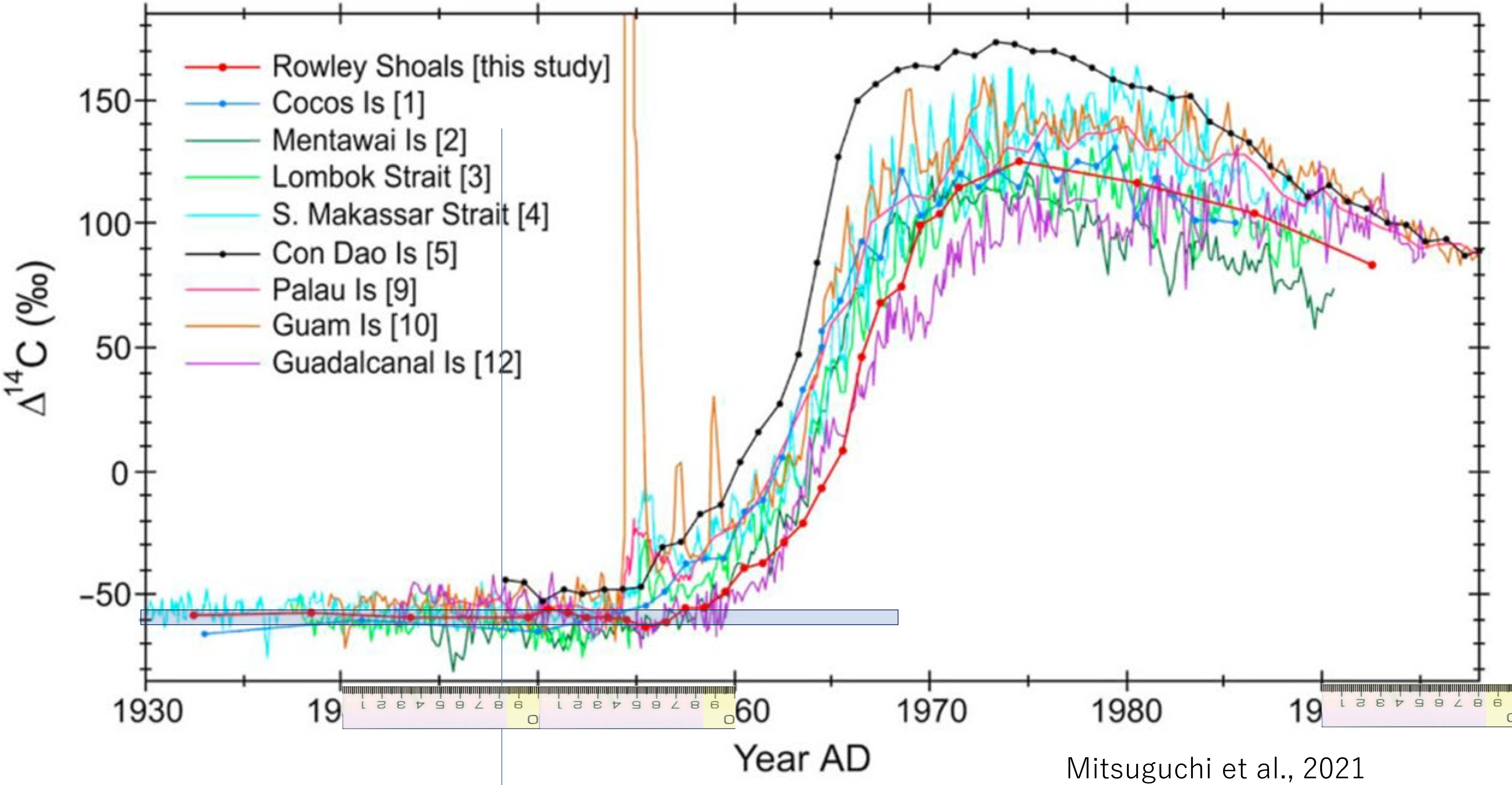

Mitsuguchi et al., 2021

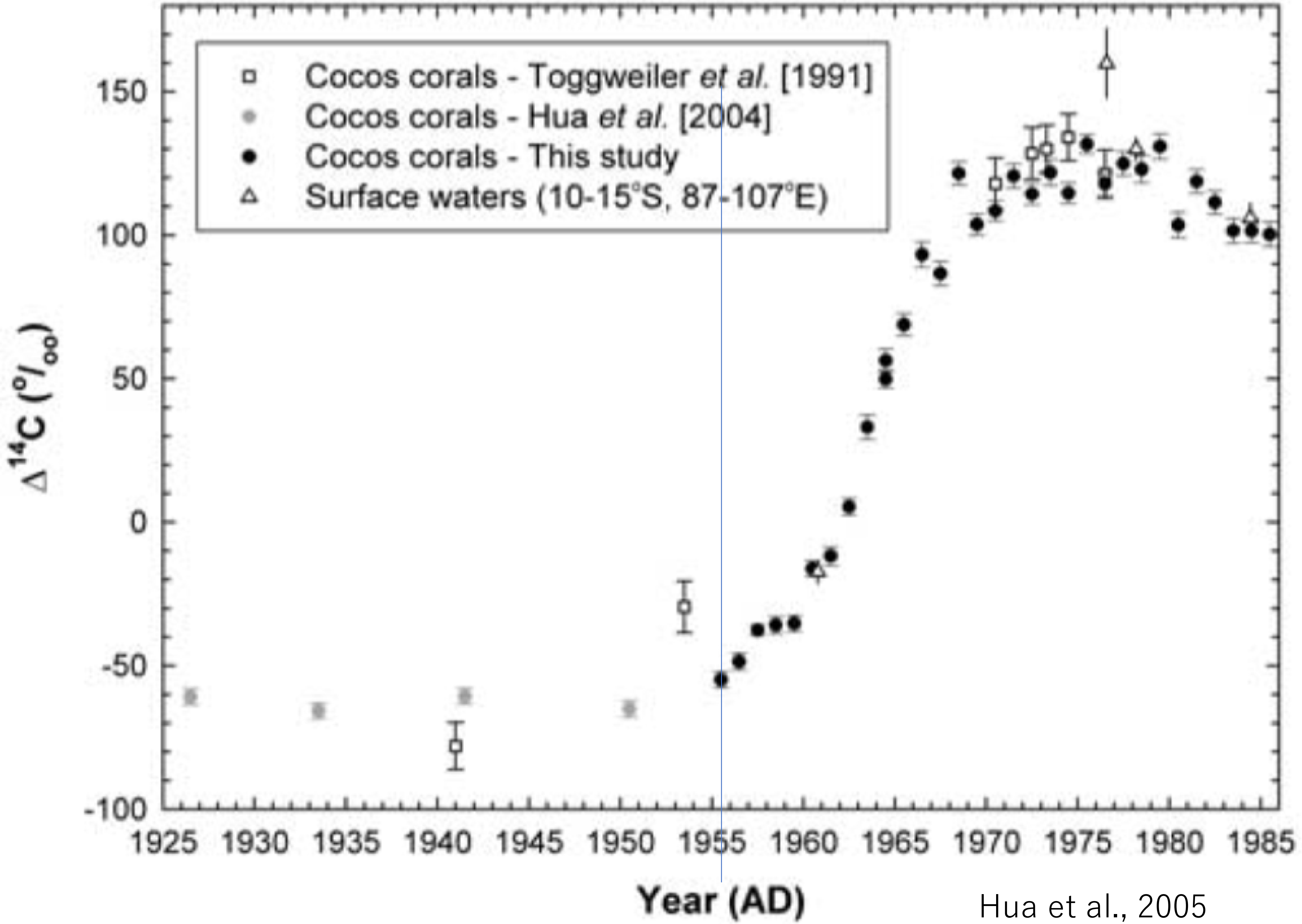

Hua et al., 2005

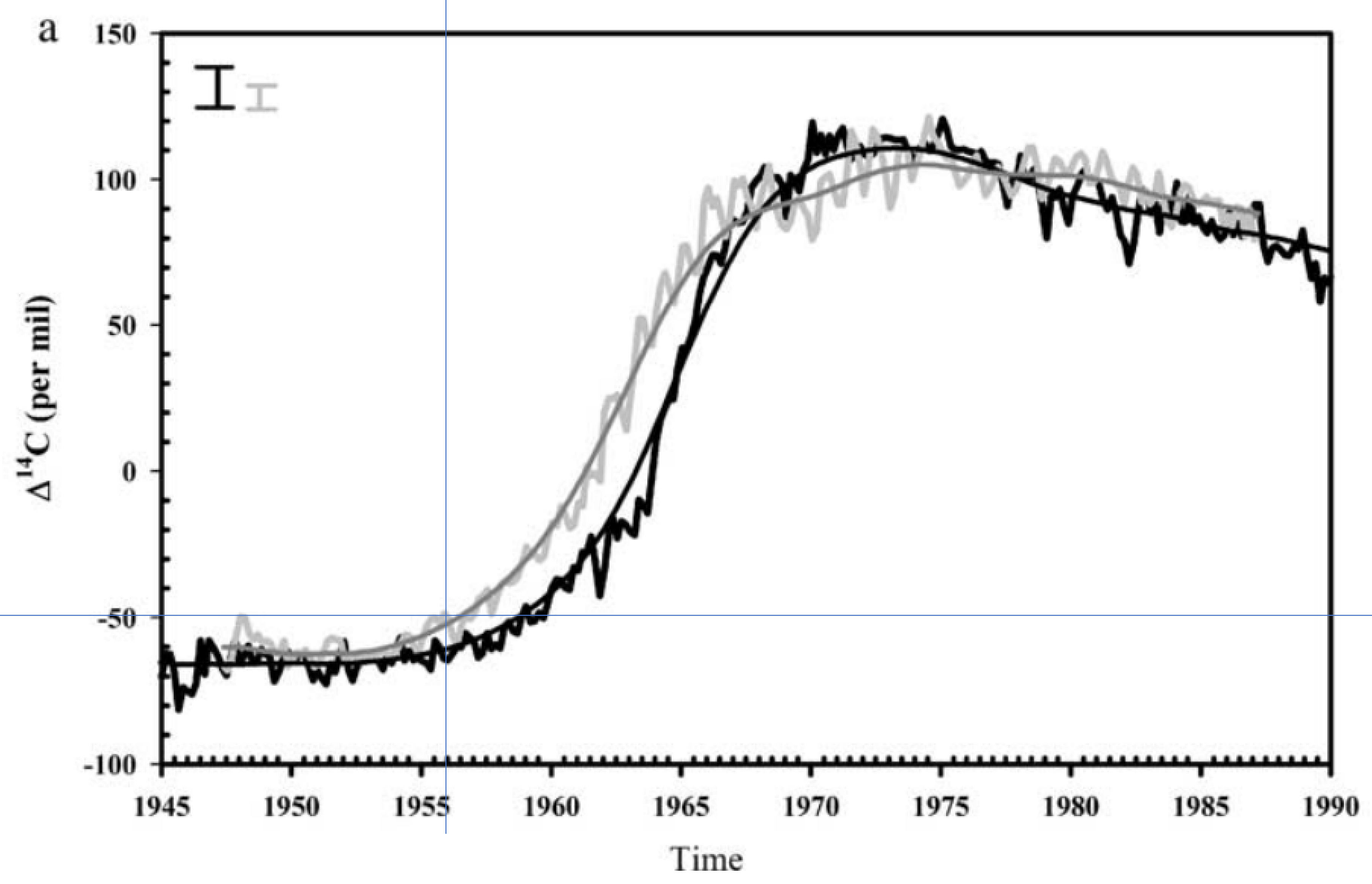

Grumet et al., 2004

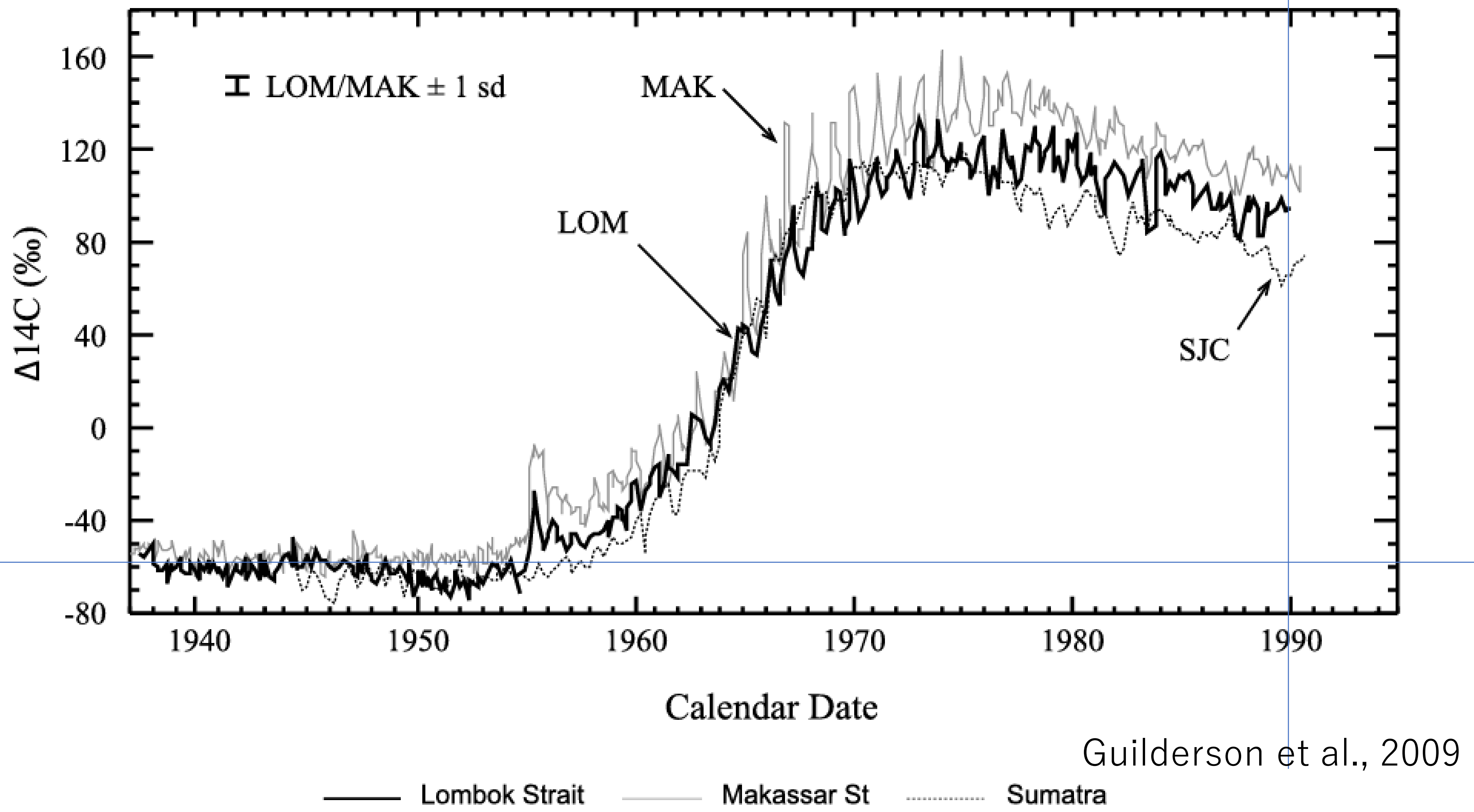

Guilderson et al., 2009

Figure 2. Surface ocean $\Delta^{14}C$ as reconstructed from reef-building hermatypic corals from Lombok Strait (LOM; thick solid line), Makassar Strait (MAK; thin gray line), and Sumatra (SJC; thin dotted line). The Lombok and Makassar data have a 1σ SD of ±3.5‰. Coral chronologies were derived from independent $\delta^{18}O$ records anchored to seasonal extremes in sea surface temperature and salinity ($\delta^{18}O_w$).

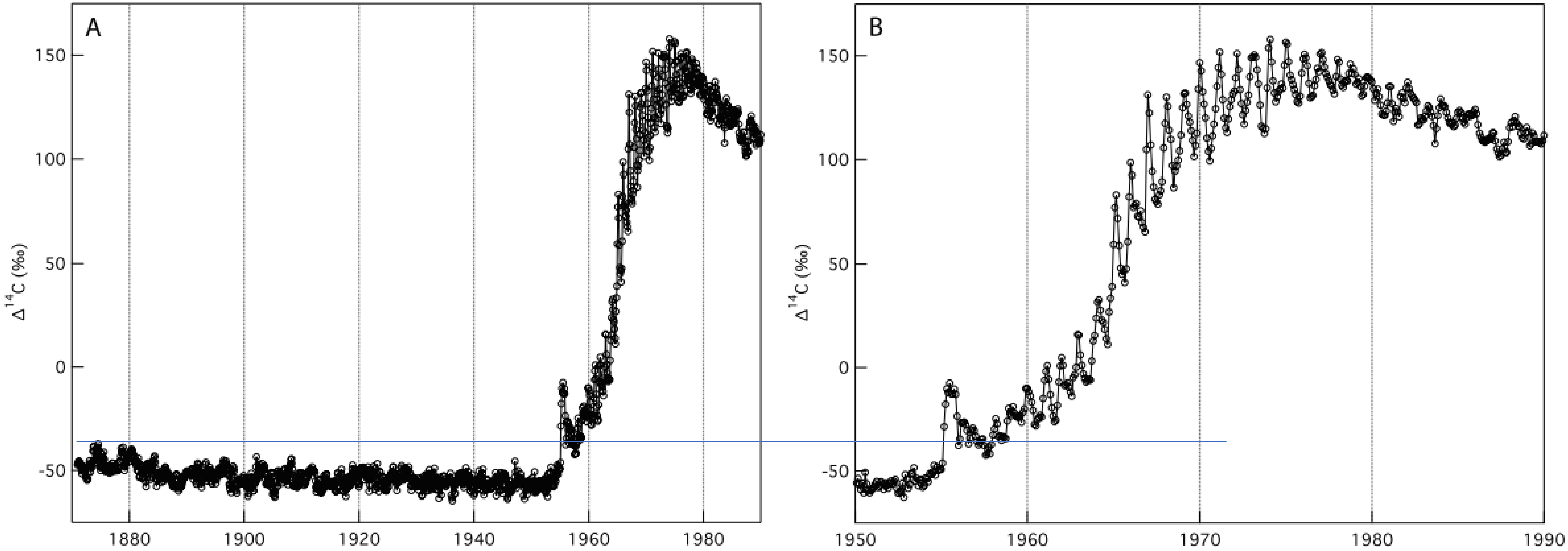

Figure 3. Coral radiocarbon record from Langkai. (a) Time series spans the time frame 1870–1990. (b) Close-up of time frame 1950–1990.

Fallon and Guilderson, 2008

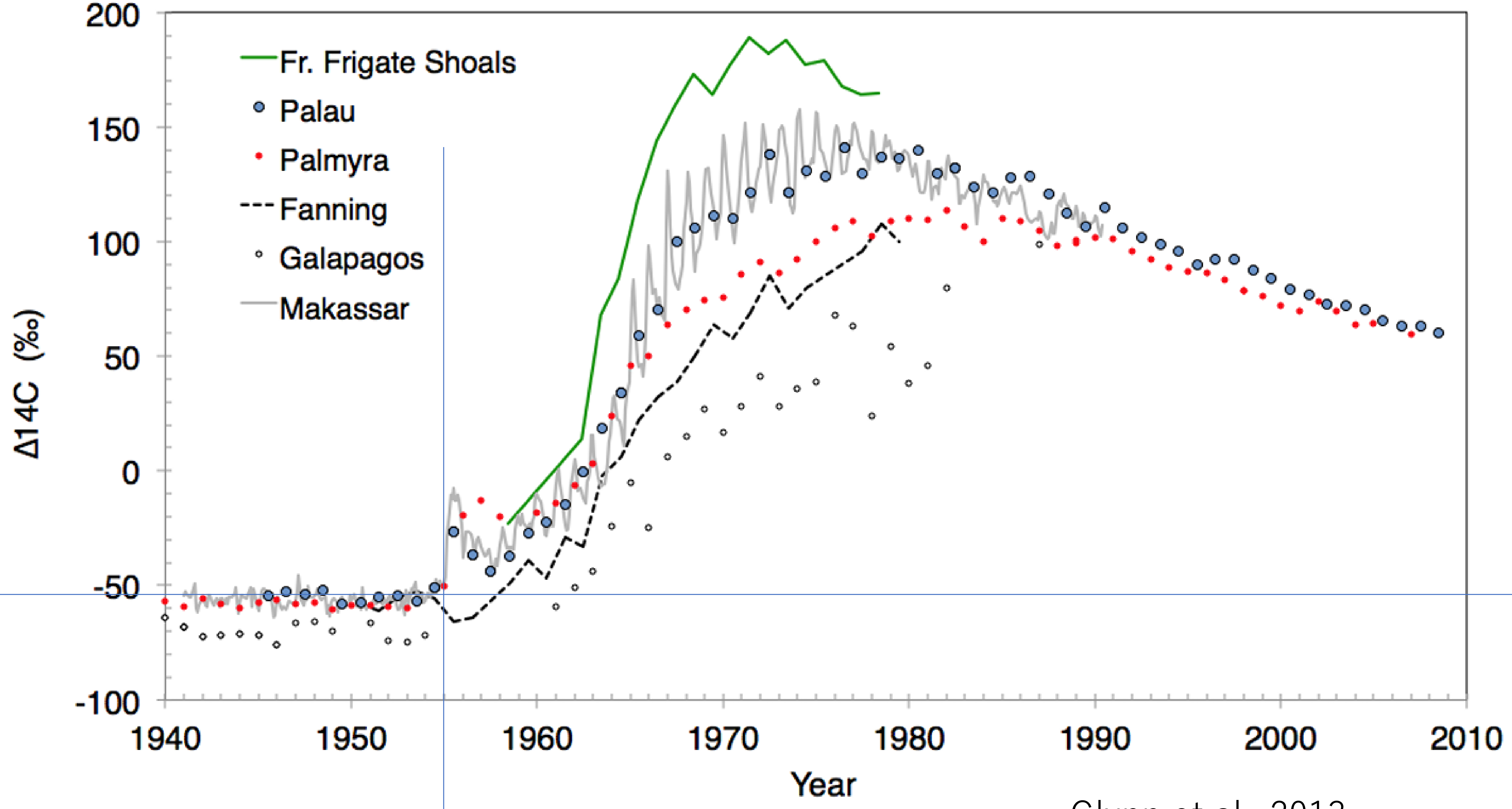

Glynn et al., 2013

Figure 1 $\Delta^{14}C$ records for corals from Palau (this study), French Frigate Shoals (24°N, 166°W), Fanning Island (4°N, 159°W), Galapagos Islands (1°S, 90°W) (Druffel 1987), Palmyra Atoll (6°N, 162°W) (Druffel-Rodriguez et al. 2012), and Makassar Straits (5°S, 119°E) (Fallon and Guilderson 2008). Tic marks indicate 1 January.

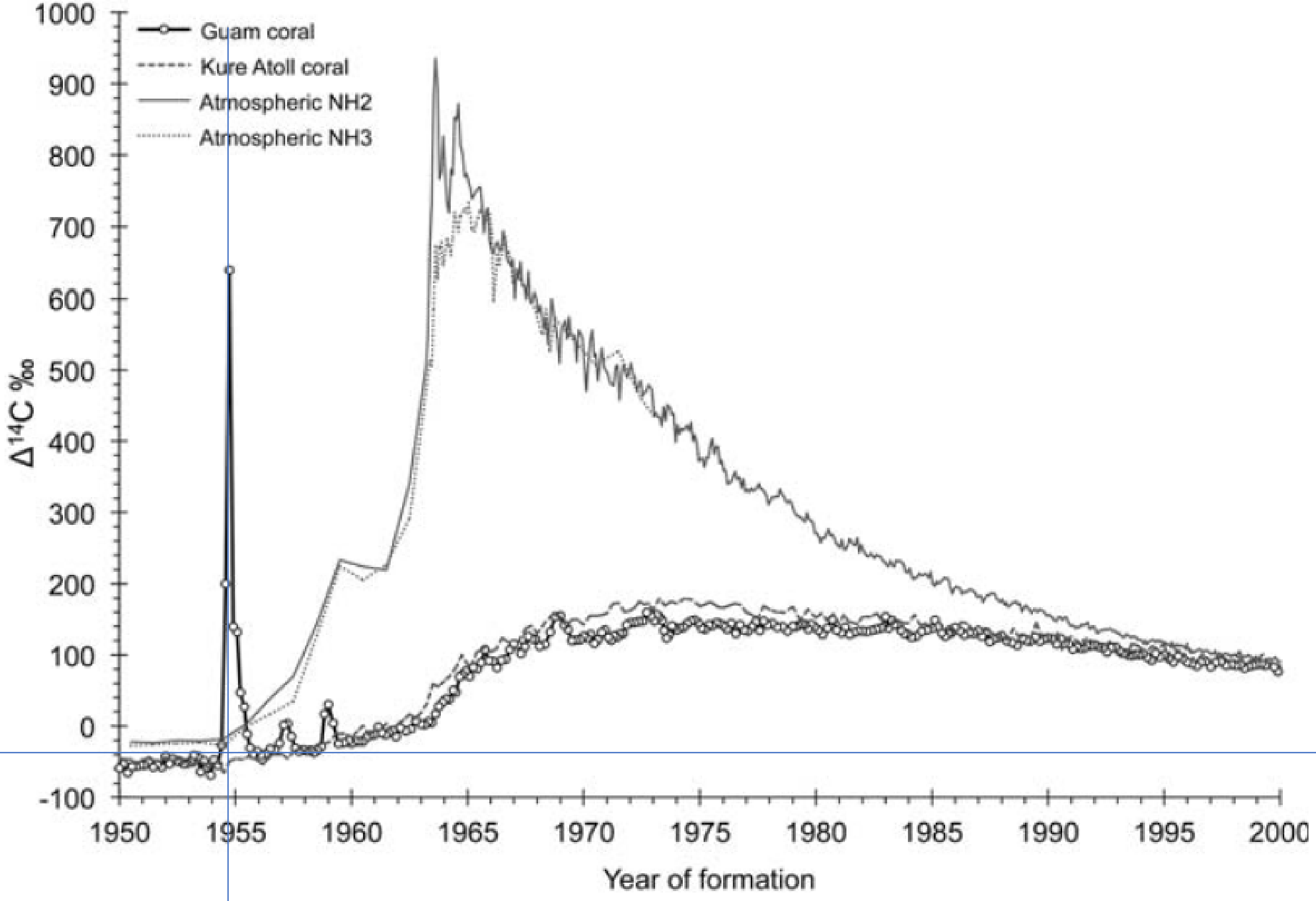

Figure 3. Plot of the new $\Delta^{14}C$ record from Guam with two atmospheric records and the Kure Atoll $\Delta^{14}C$ record [*Andrews et al.*, 2016]. The unexpectedly high spike from the Guam coral, followed by two smaller spikes, was more than can be explained by air-sea diffusion alone. The Kure Atoll record exemplifies the expected marine ^{14}C signal (no spikes). The largest spike approached maximum atmospheric $\Delta^{14}C$ levels at ~640‰ and can be linked to close-in fallout from nuclear testing at the US Pacific Proving Grounds in the Marshall Islands. Atmospheric records were plotted to cover the region of interest (~140°E–170°E) [*Hua et al.*, 2013].

Andrews et al., 2016

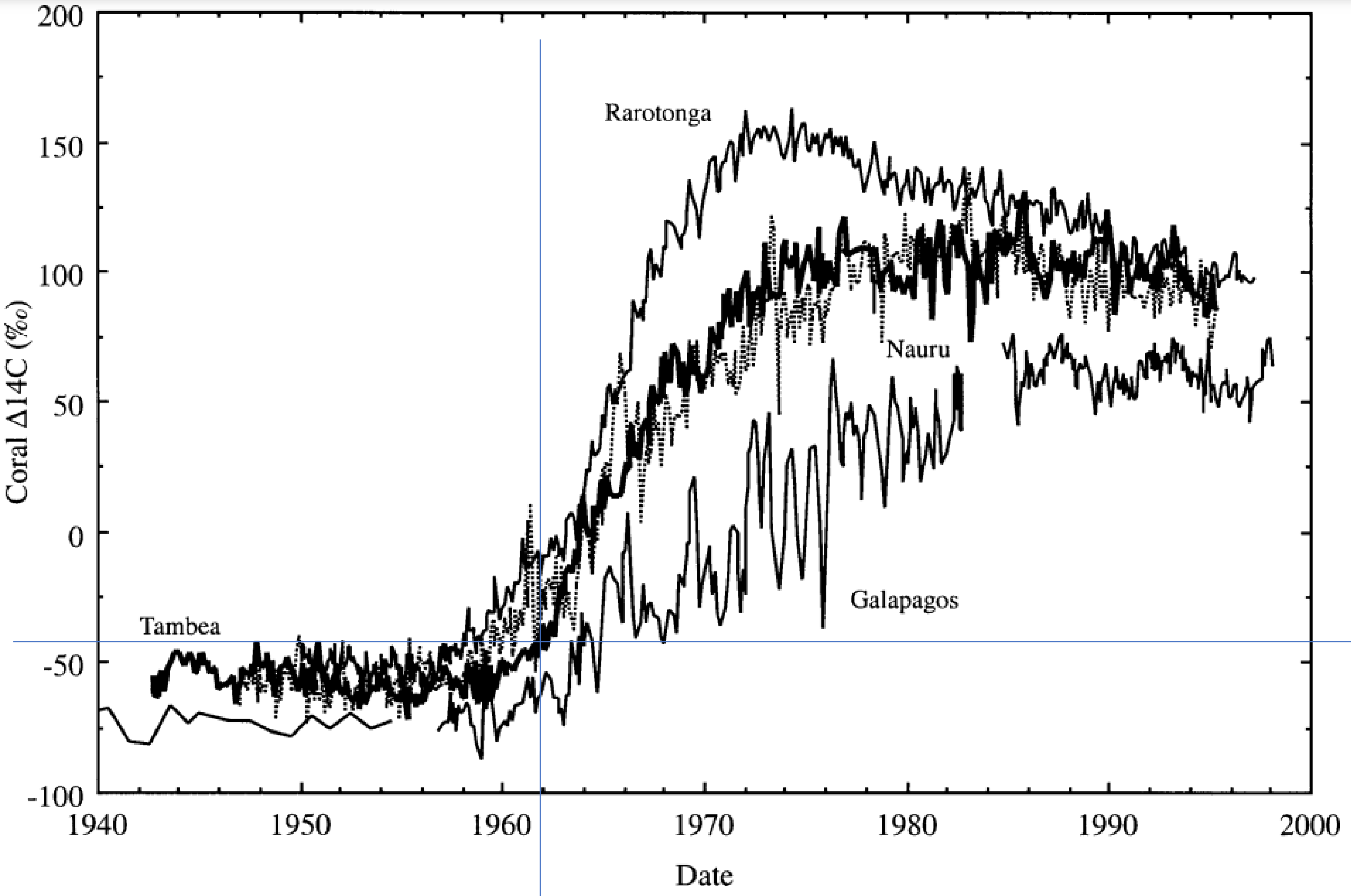

FIG. 4. Solomon Sea surface water $\Delta^{14}C$ (‰) as recorded in the Tambea coral. Individual (average 1.5-month sample resolution) values span a range from a low of −72‰ to a high of 132‰. Mean annual values range from −63‰ (1952) to 117‰ (1985). For comparison we have also plotted results from Nauru (0.5°S, 166°E; Guilderson et al. 1998); Rarotonga (20°S, 160°W; Guilderson et al. 2000b), and the Galapagos (Urvina Bay; Guilderson and Schrag 1998), and Wenman (T. P. Guilderson and D. P. Schrag 2000, unpublished manuscript). In a simple sense, the waters of the Solomon Sea reflect mixing of subtropical waters (Rarotonga) brought in from the southernmost branch of the SEC with water from the equatorward branch of the SEC whose origins lie in the eastern Pacific (Galapagos).

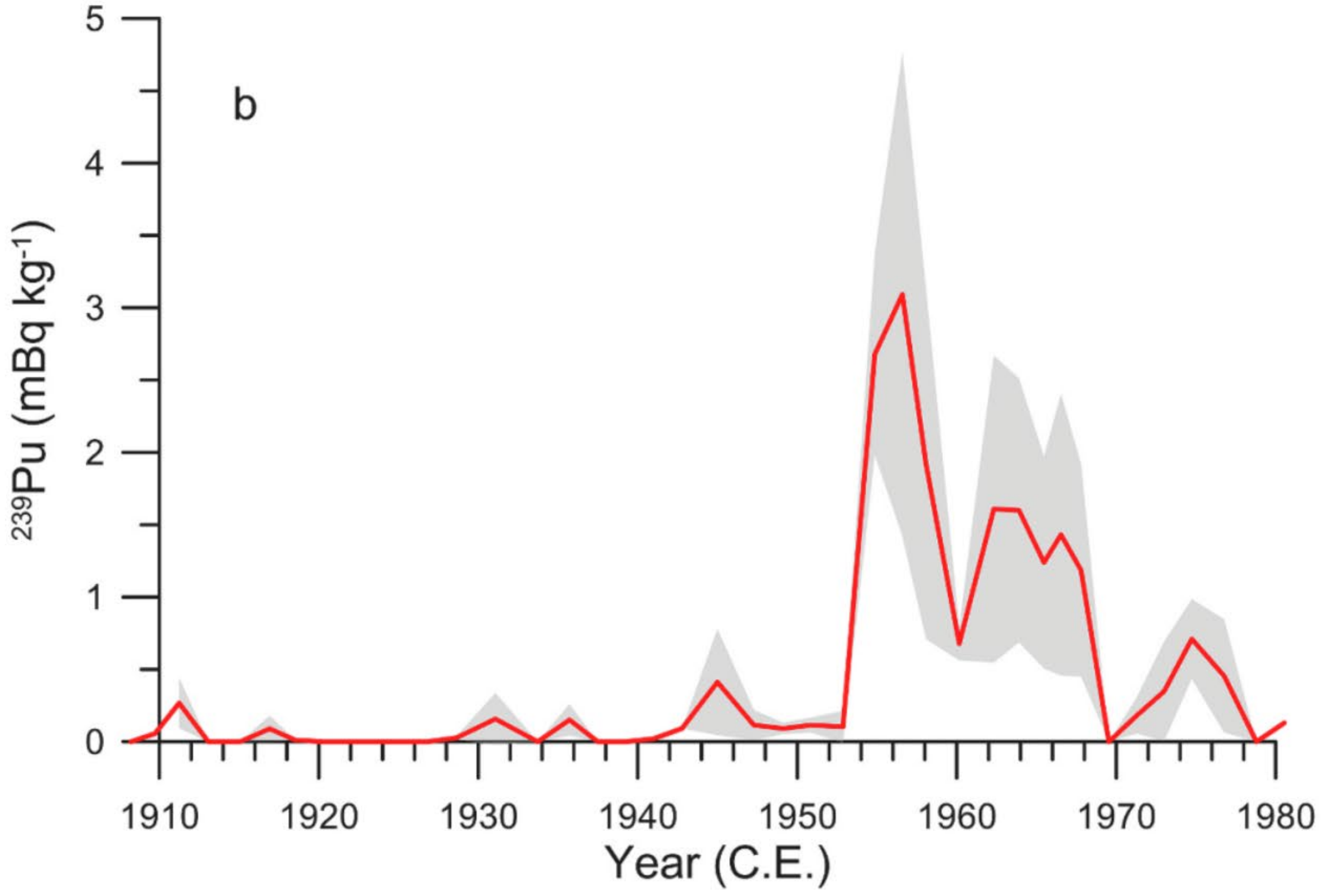

Fig. 4. Concentration profile of ^{239}Pu (panel a) and activity profile (panel b) as recorded in the DC-3D ice core. The shaded areas in both panels show the uncertainties arising from our semi-quantitative method.

Severi et al. (2023)

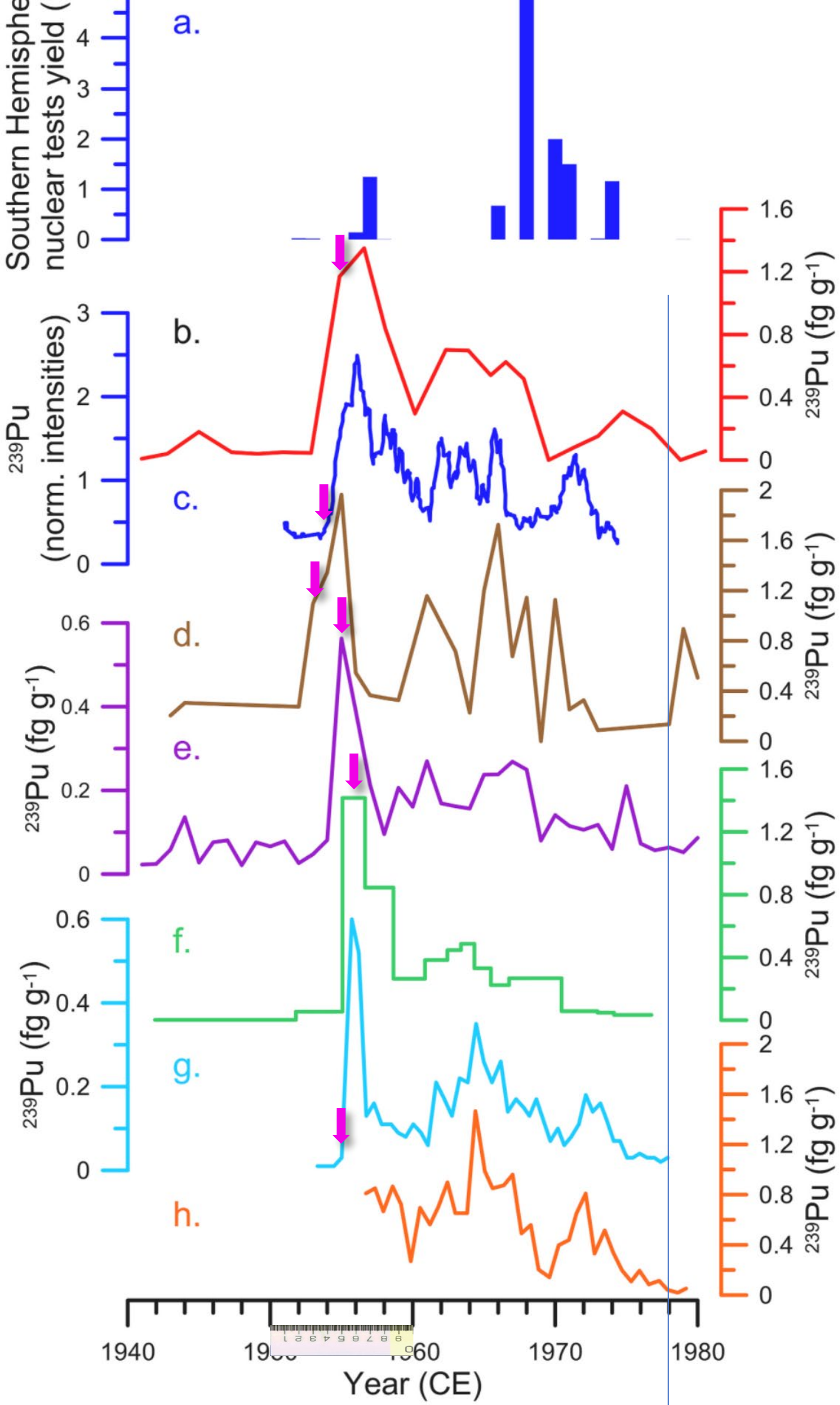

able records. (a) Yearly yield of southern hemisphere nuclear tests run between 1940 and 1980 C.E. (b) DC-3D semi-quantitative ^{239}Pu concentration (red), (c) RICE ^{239}Pu normalized intensities (blue – Winstrup et al., 2019), (d) NUS_8_5 ^{239}Pu concentration (brown- Arienzo et al., 2016)), (e) Antarctic composite record of ^{239}Pu concentration (purple- Arienzo et al., 2016)), (f) Old Dome C calculated ^{239}Pu concentration (green - Koide et al., 1979 and Koide et al., 1985), (g) J-9 calculated ^{239}Pu concentration plotted on the chronology proposed by Hwang et al. (2019) (light blue - Cutter et al., 1979 and Koide et al., 1985), (h) Dome Fuji snow-pit ^{239}Pu concentration (orange- Hwang et al., 2019).

Severi et al. (2023)

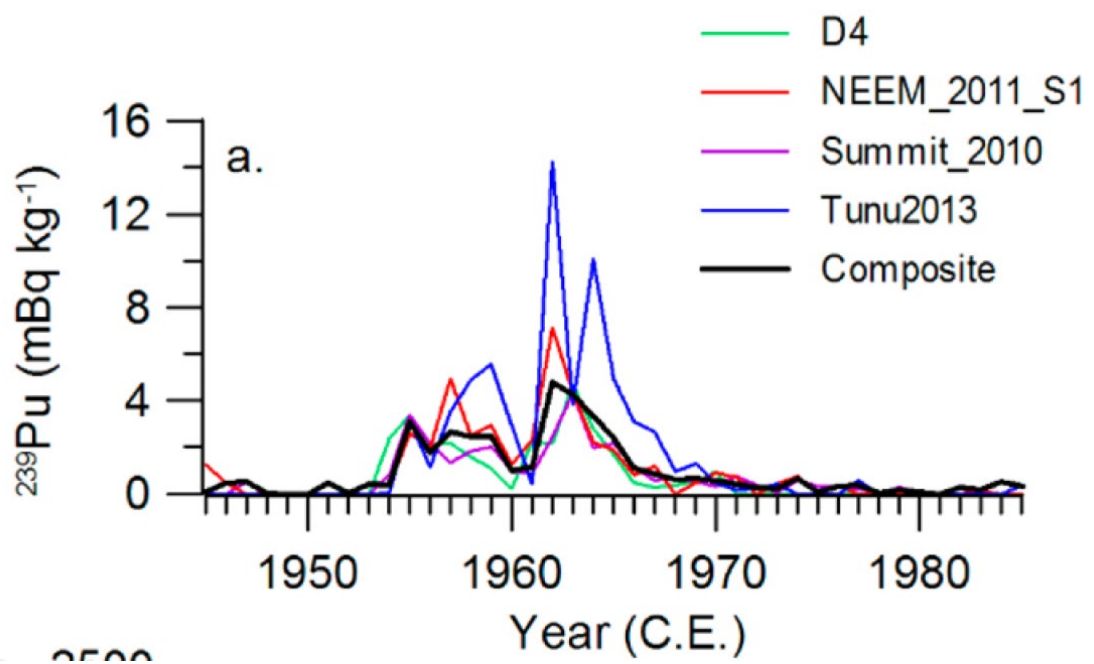

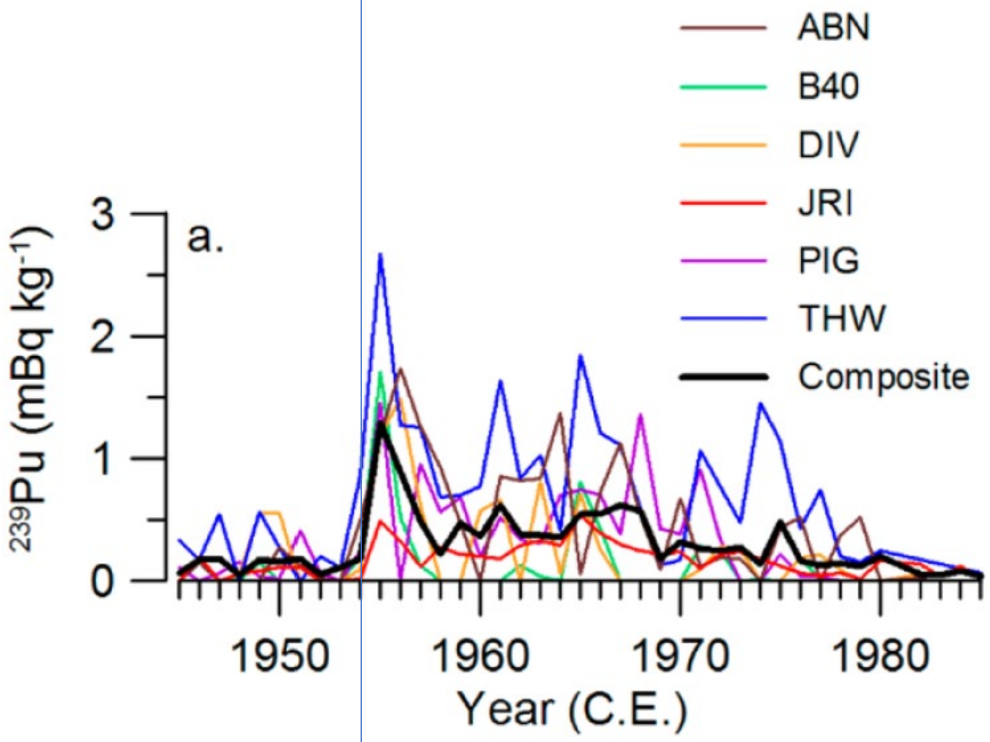

Arienzo et al., 2016

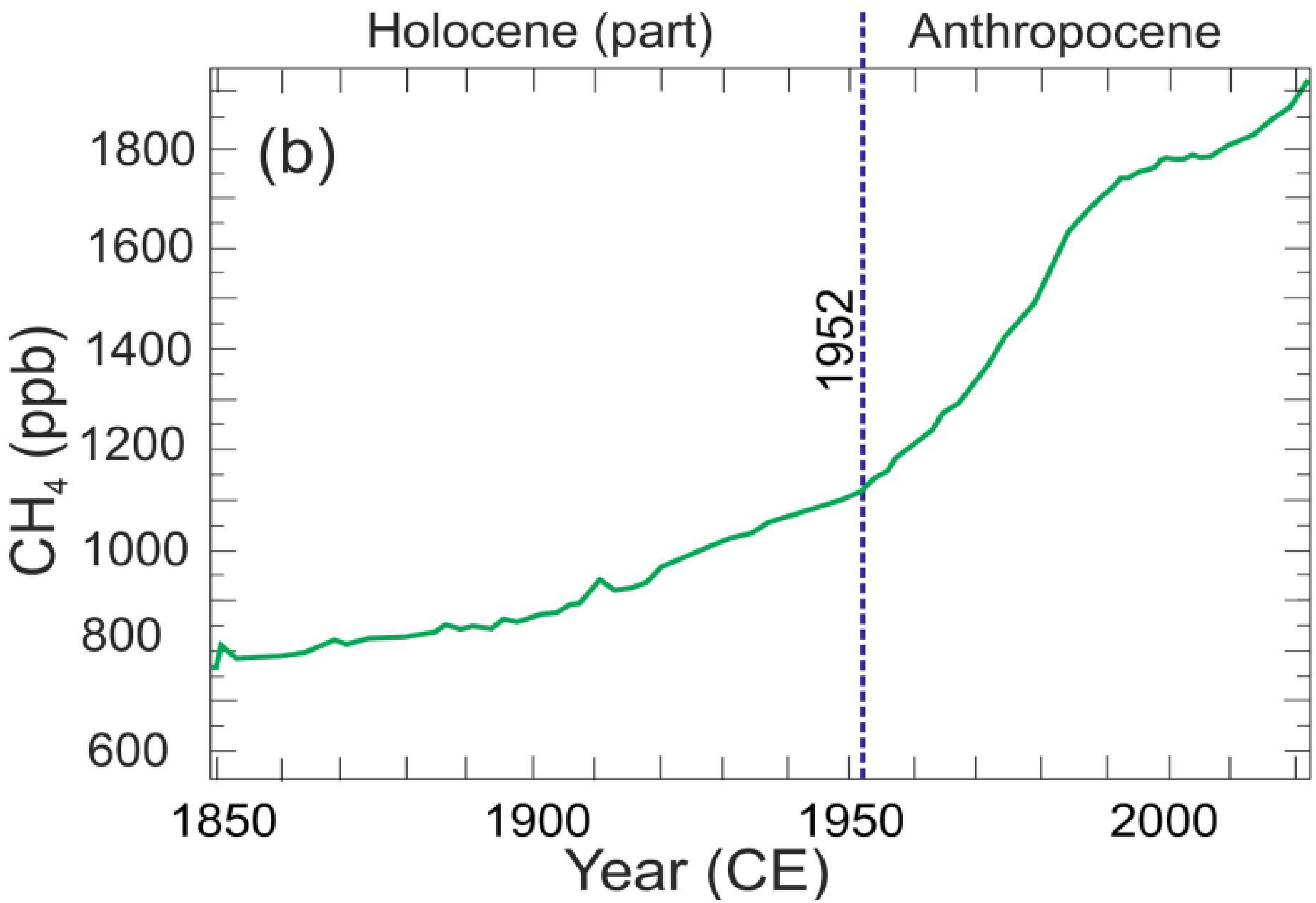

Figure 9. Atmospheric CH_4 records: (a) in ice core from Greenland GISP 2 (Mitchell et al., 2003) and Antarctic Law Dome ice core, firn data and air samples (Ferretti et al., 2005); reproduced from Zalasiewicz et al. (2020); (b) since 1850 CE in Antarctic Law Dome ice core, firn data and air samples (Ferretti et al., 2005, updated with data from Mauna Loa Observatory Hawaii from NOAA Climate.gov.).

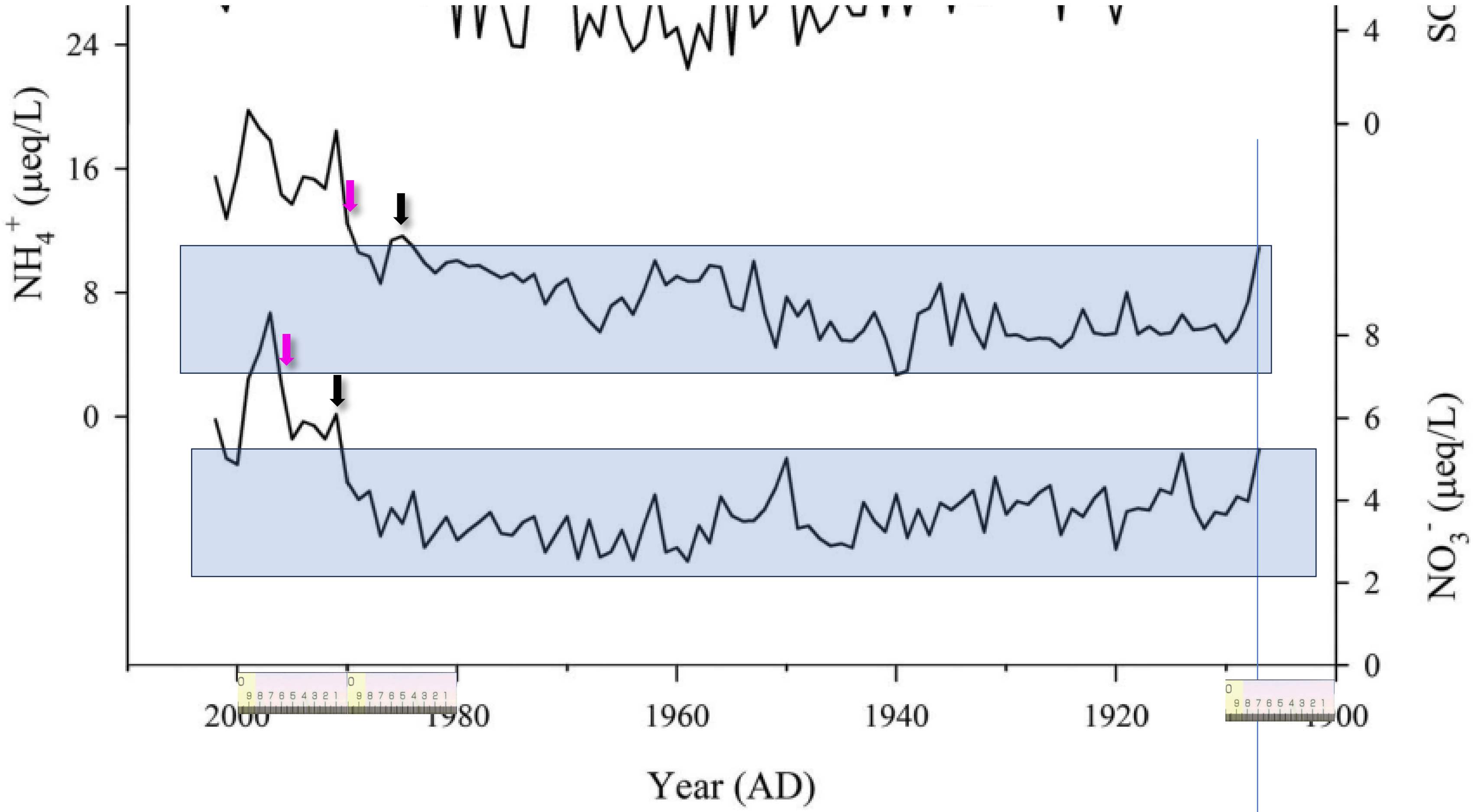

Figure 3. Annual time series of major soluble ion concentrations in Muztagata ice core.

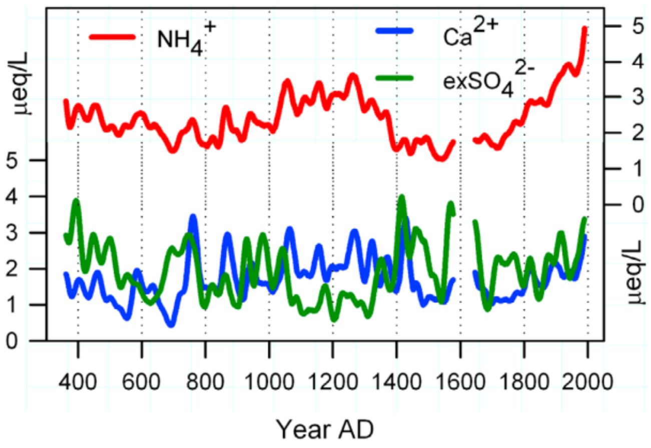

Figure 6. Records (smoothed with a 39-year Gaussian filter) for excess sulfate (green, left-hand scale), calcium (blue, left-hand scale), and ammonium (red, right-hand scale) for the time period 350–1998 AD.

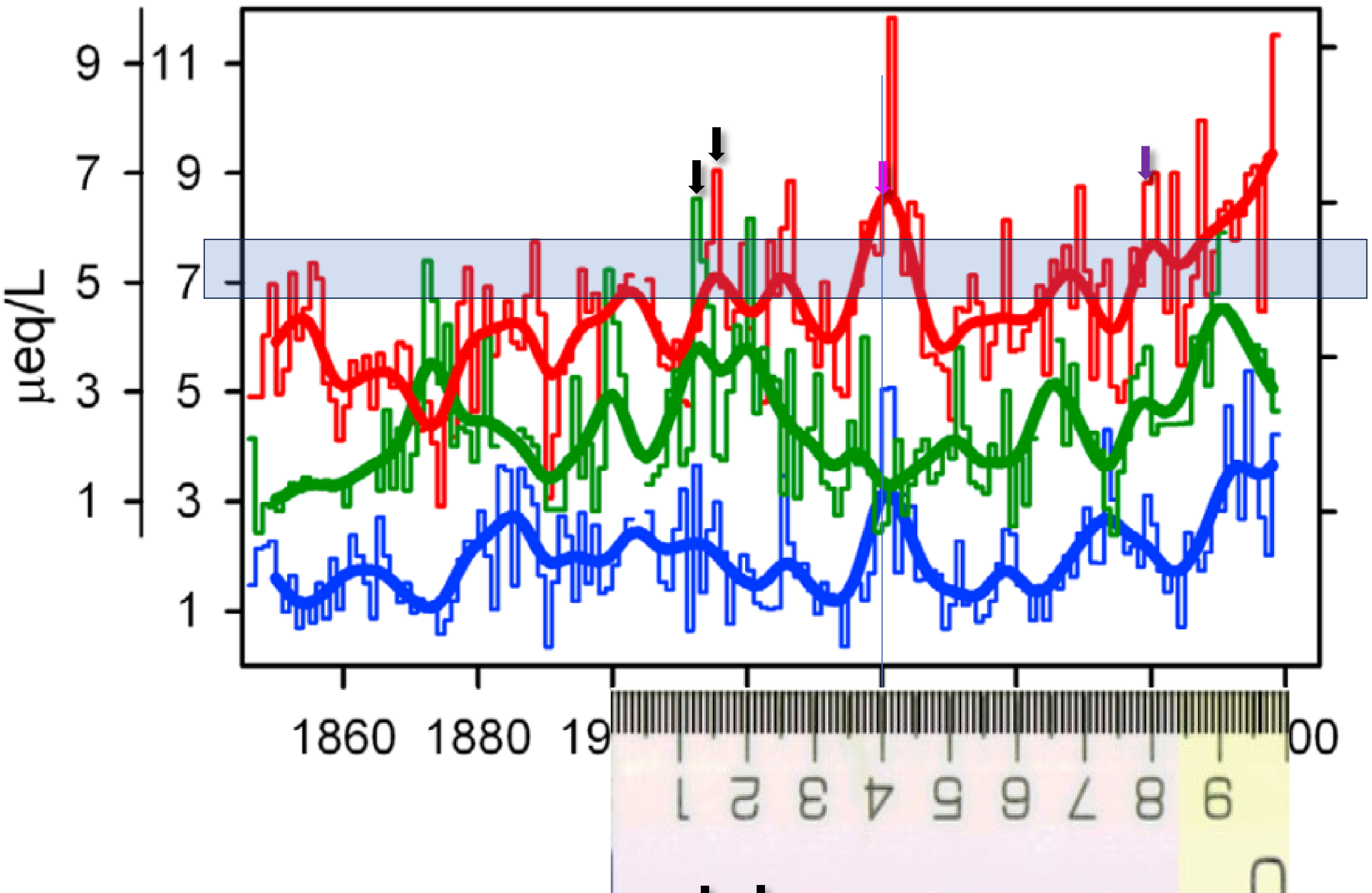

Figure 5. Annually ave[illegible]thed with a 21-point binomial filter (bold curves) records for excess sulfate as a tracer for anthropogenic emissions (green, left-hand scale), calcium as a dust tracer (blue, left-hand scale), and ammonium as a proxy for temperature (red, right-hand scale) for the time period 1850–1998 AD.

Kellerhals et al. (2010)

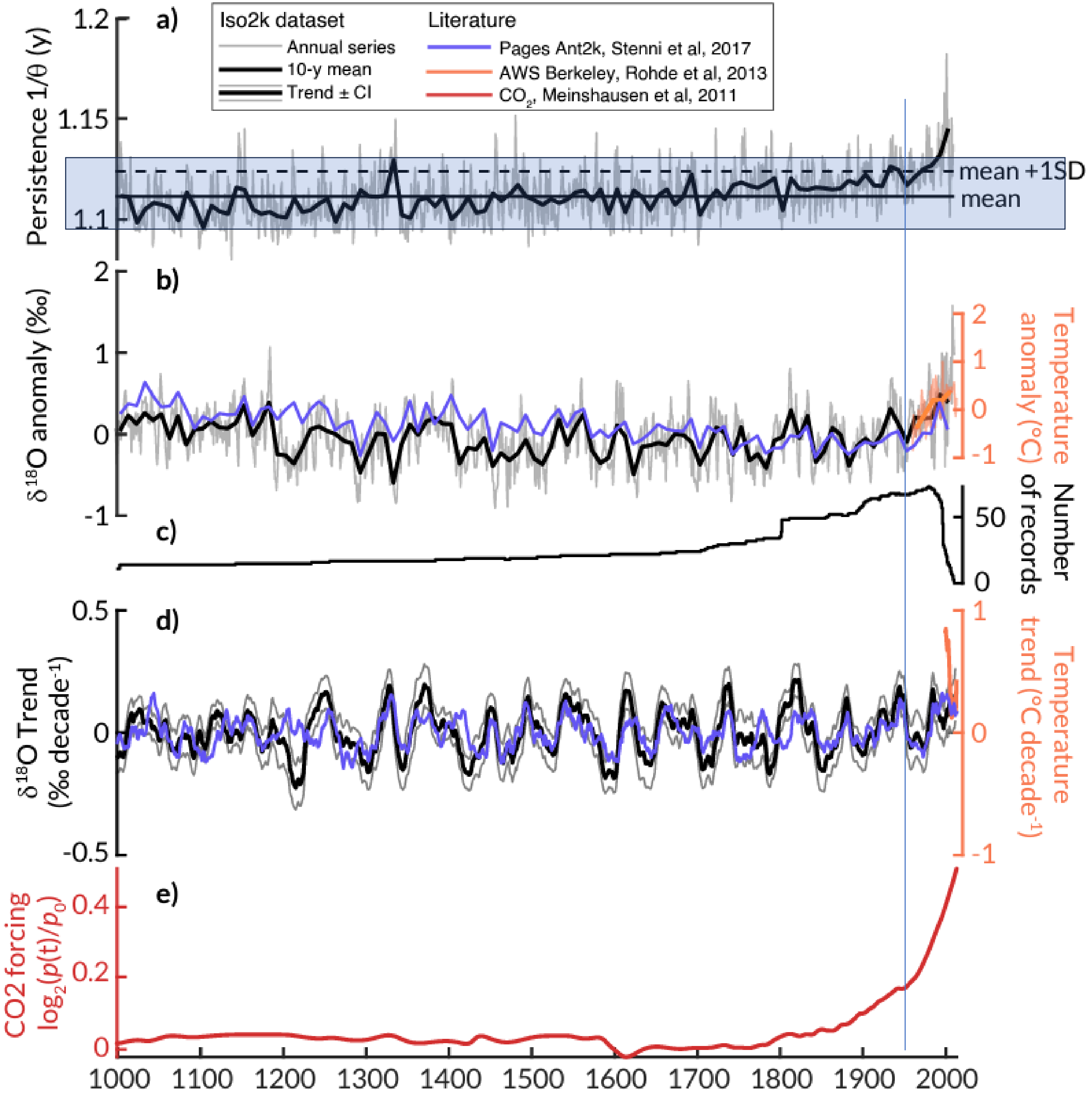
a)
b)
c)
d)
e)
Iso2k dataset
Annual series
10-y mean
Trend ± CI
Literature
Pages Ant2k, Stenni et al, 2017
AWS Berkeley, Rohde et al, 2013
CO2, Meinshausen et al, 2011
Persistence 1/θ (y)
mean +1SD
mean
δ18O anomaly (‰)
Temperature anomaly (°C)
Number of records
δ18O Trend (‰ decade-1)
Temperature trend (°C decade-1)
CO2 forcing log2(p(t)/p0)

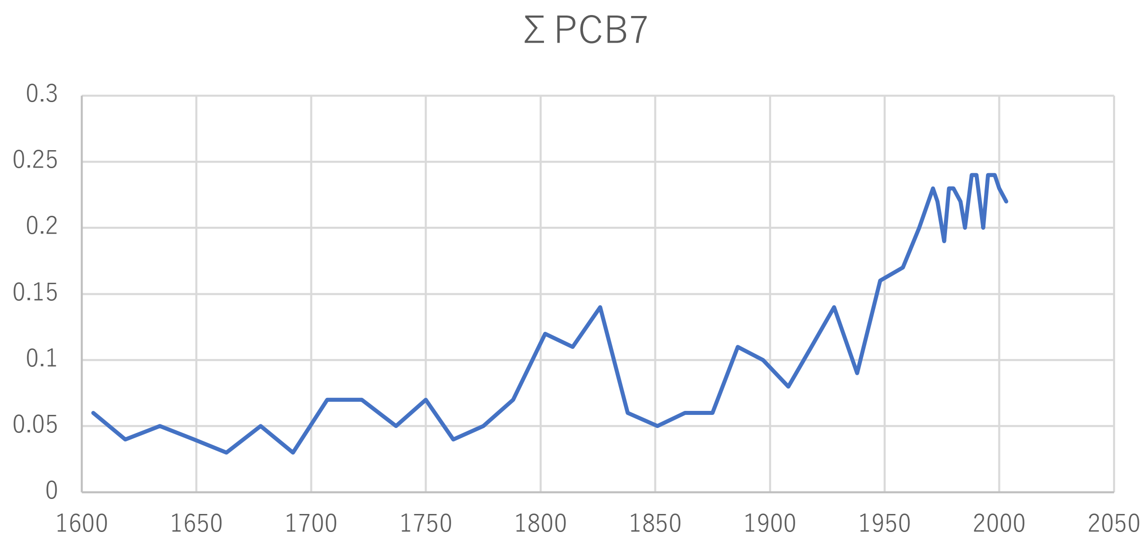

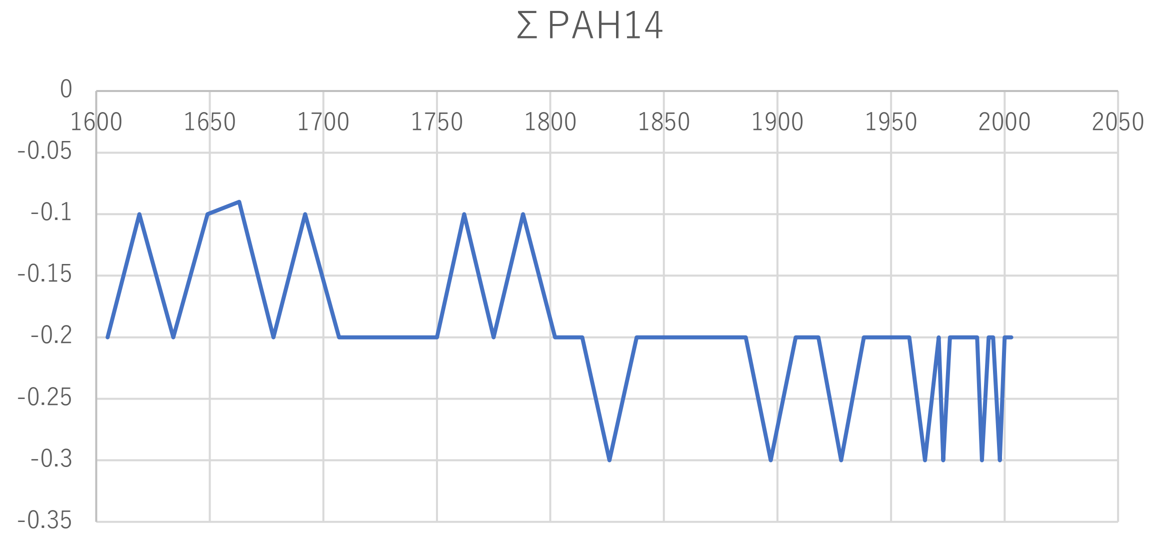

Fuoco et al., 2012

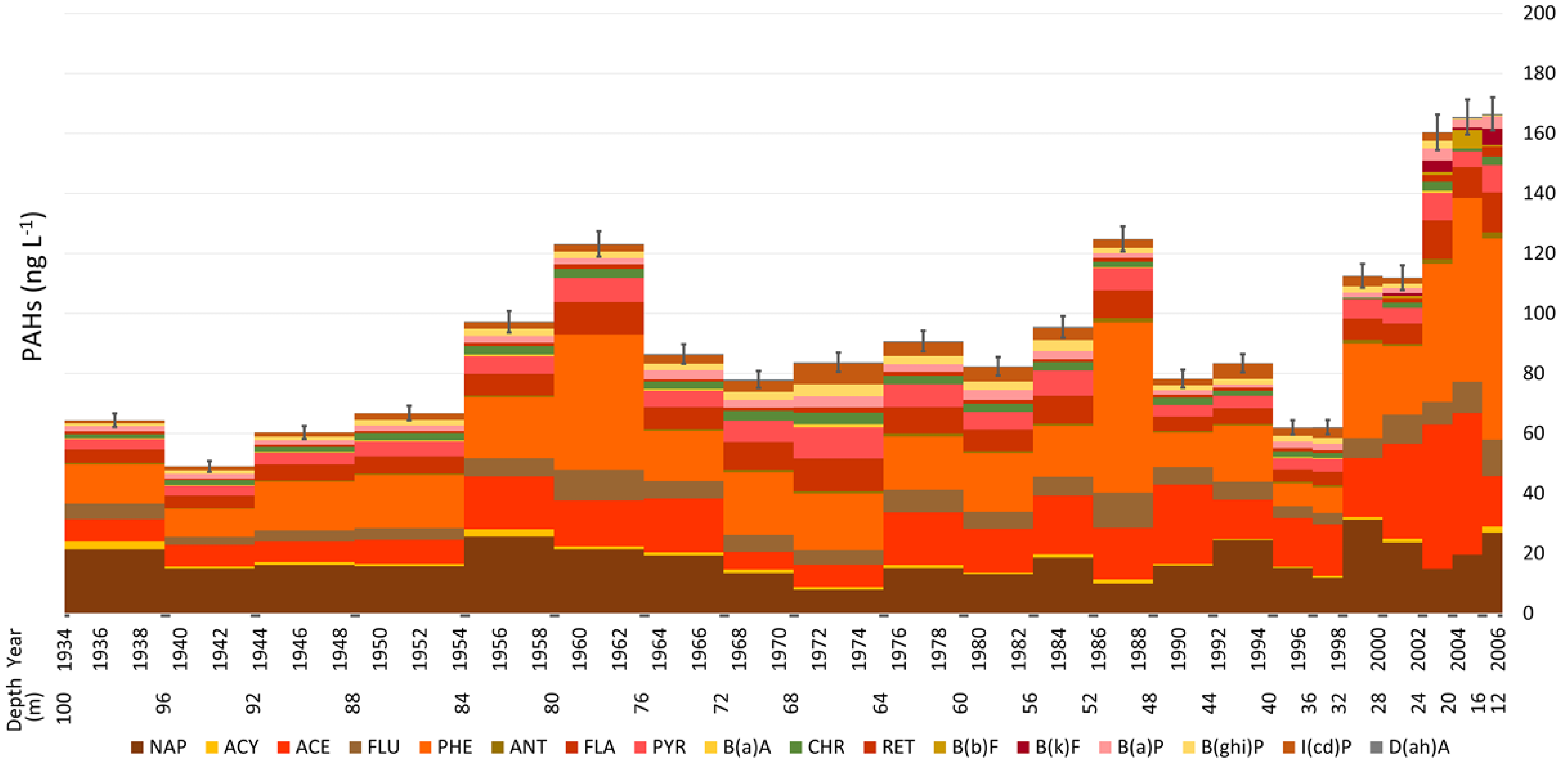

Vecchiato et al., 2020

Figure 2. Fragrances and PAHs (ng L^{-1}) in the Elbrus ice core with the year of snow deposition (CE) and the sample depth (m) reported along the abscissas. Error bars correspond to the standard deviation percentage of the method precision. Seasonal dating uncertainties of the analyzed ice core sections (0.5 years) are shown below the x-axes. Only detected compounds are displayed. Note the different y-axis scales for the fragrances and PAHs.

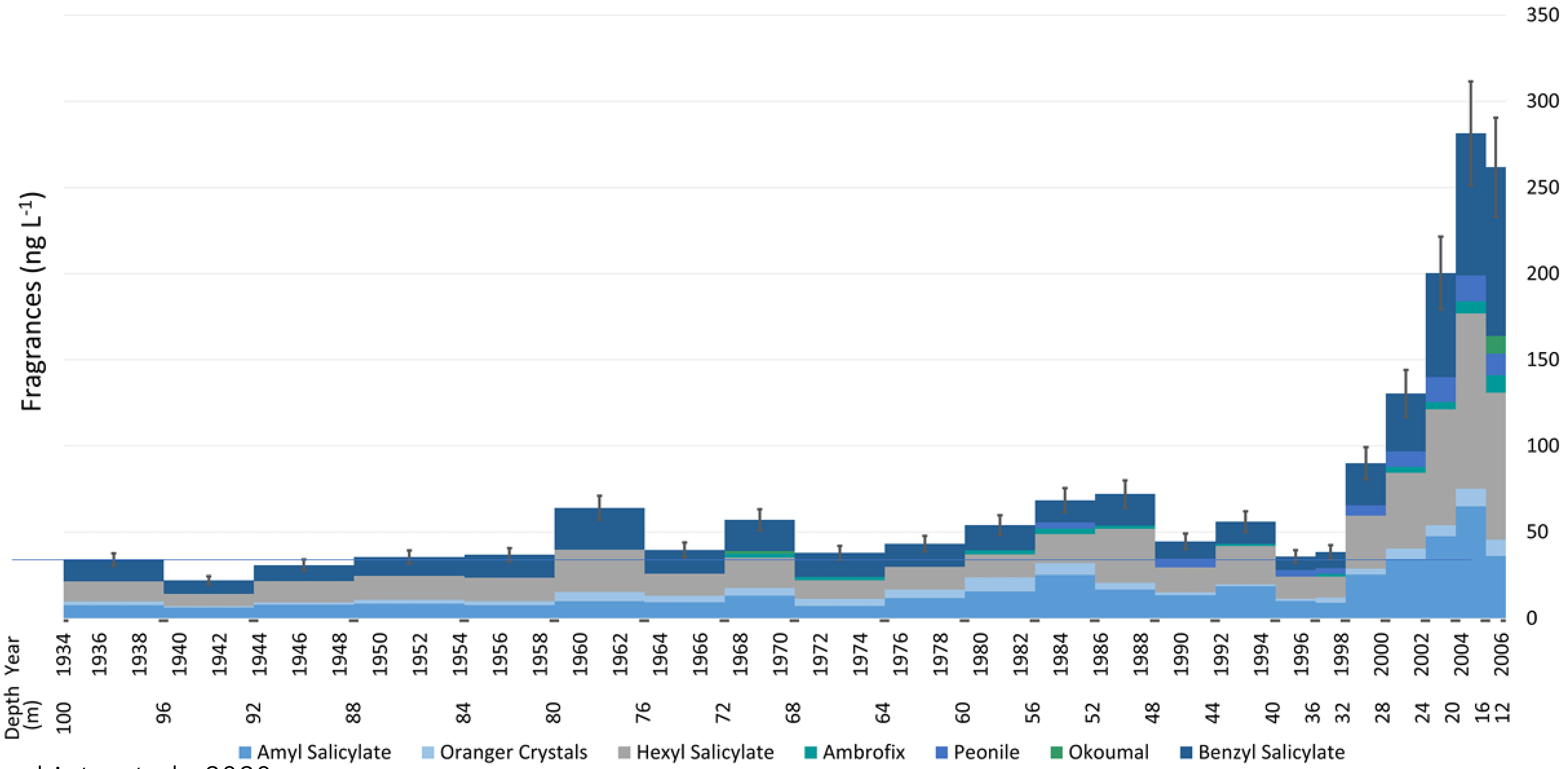

Vecchiato et al., 2020

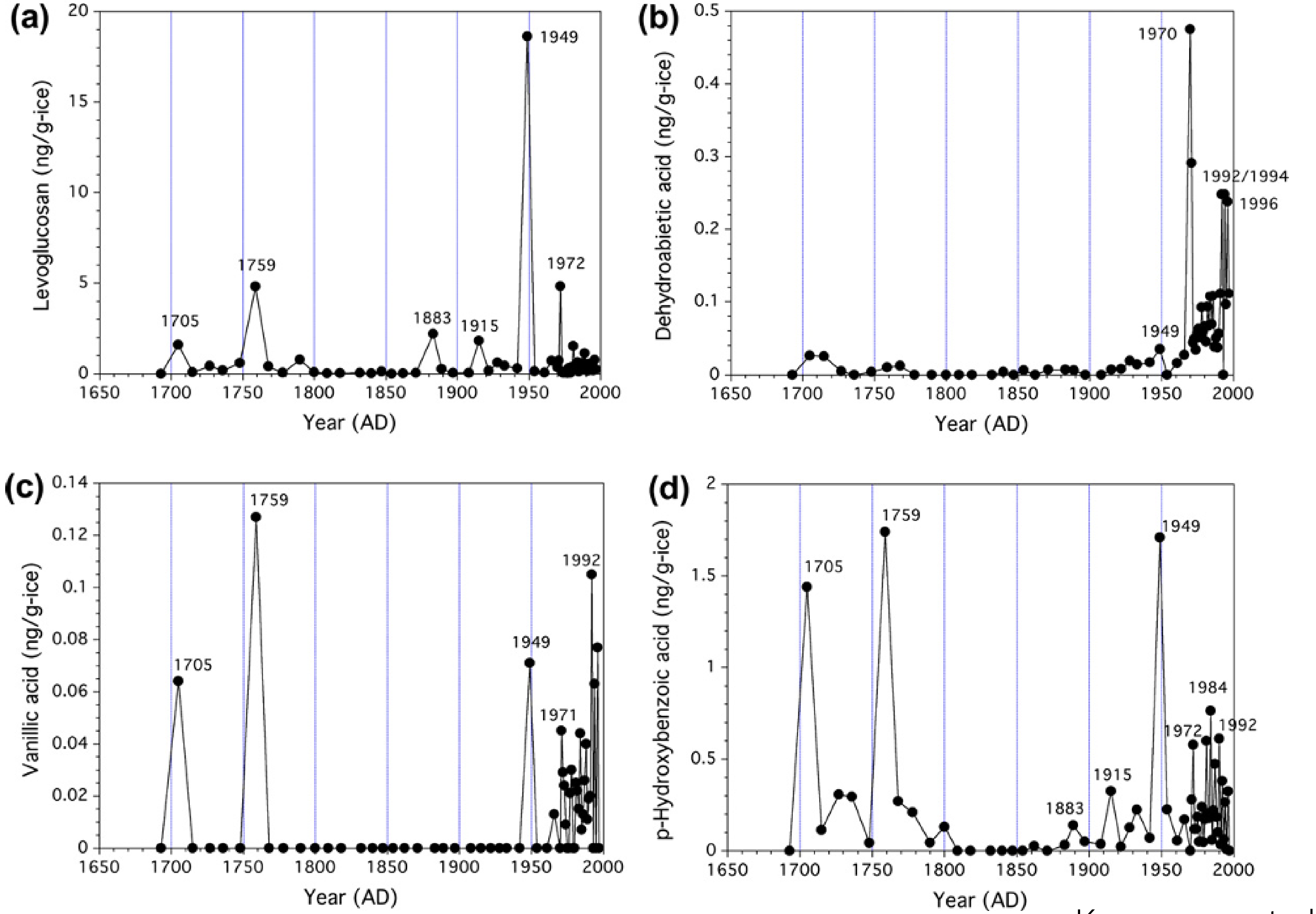

Kawamura et al. (2012)

Fig. 4. Historical variations of (a) levoglucosan, (b) dehydroabietic acid, (c) vanillic acid, and (d) *p*-hydroxybenzoic acid in the Ushkovsky ice core.

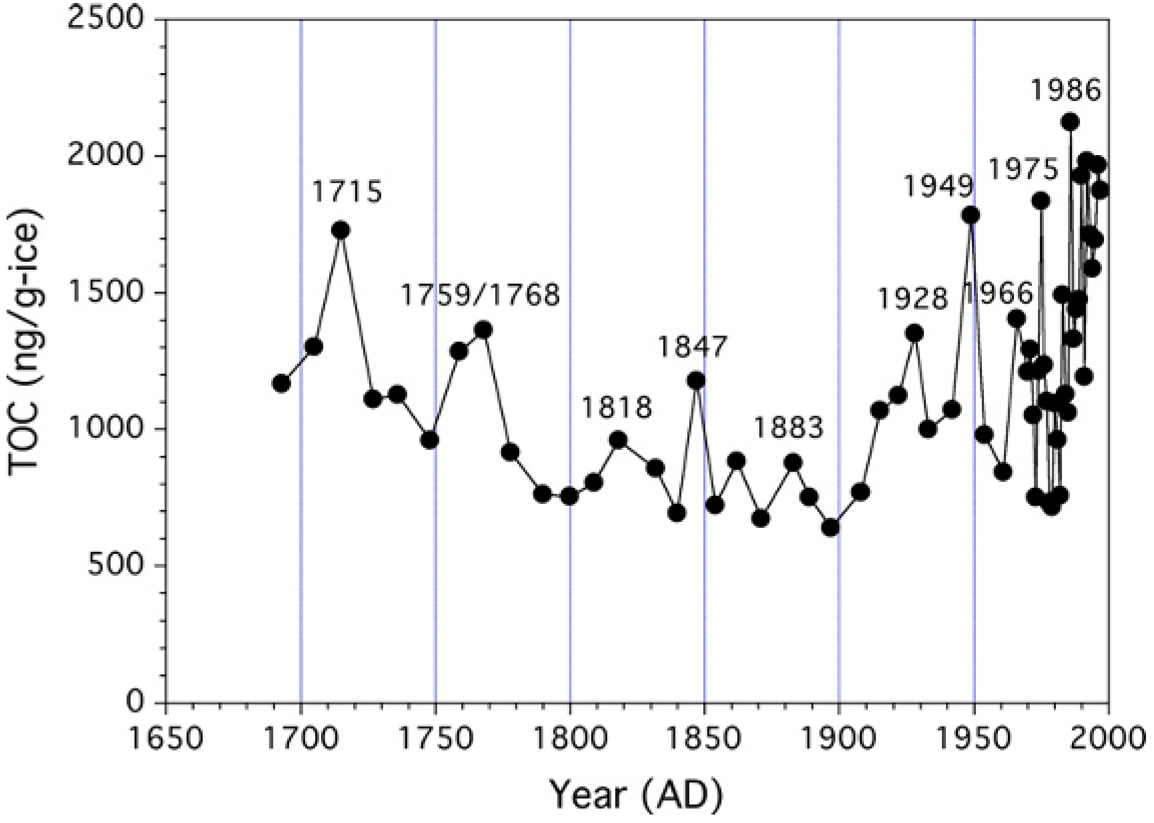

Kawamura et al. (2012)

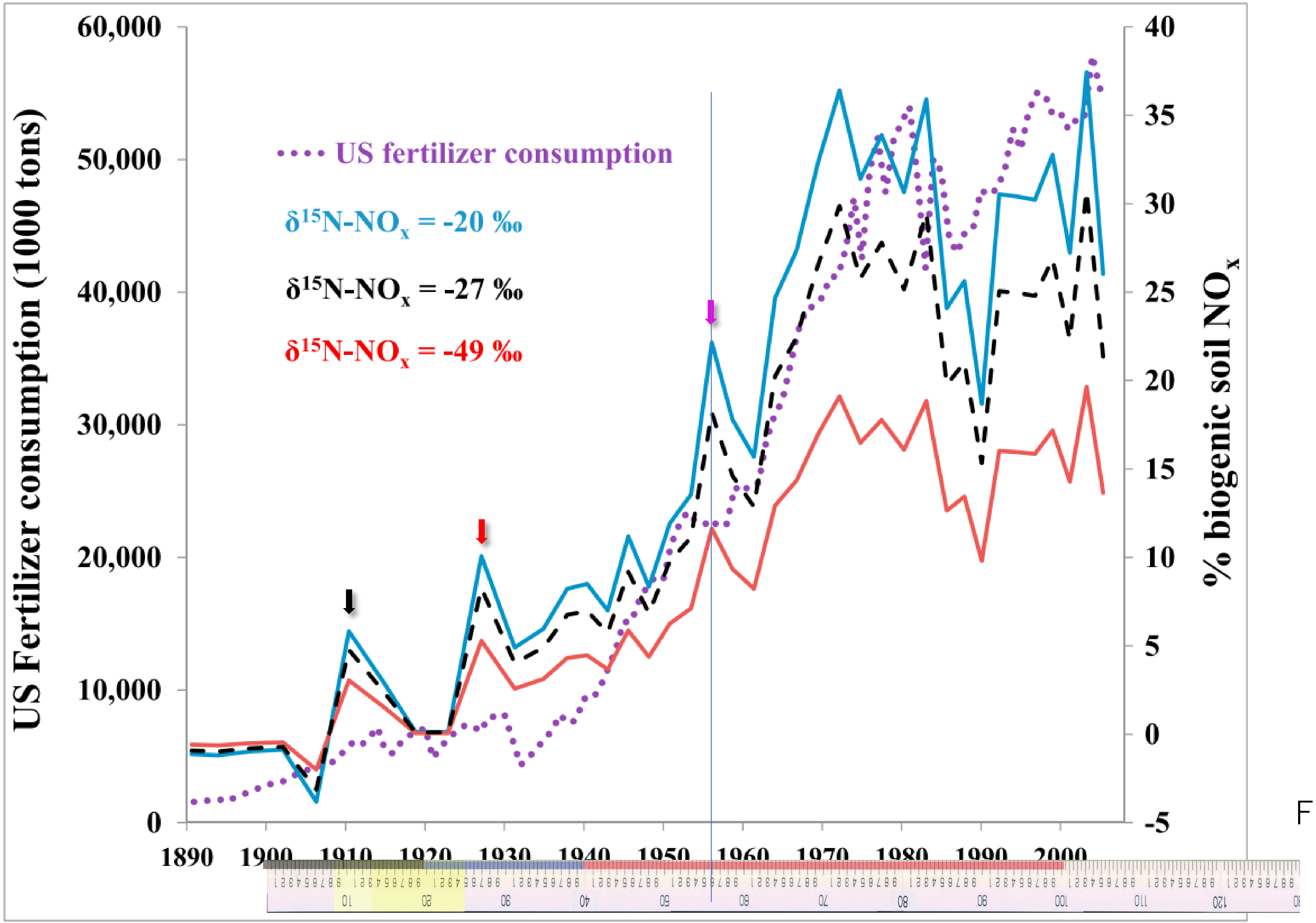

Felix et al., 2013

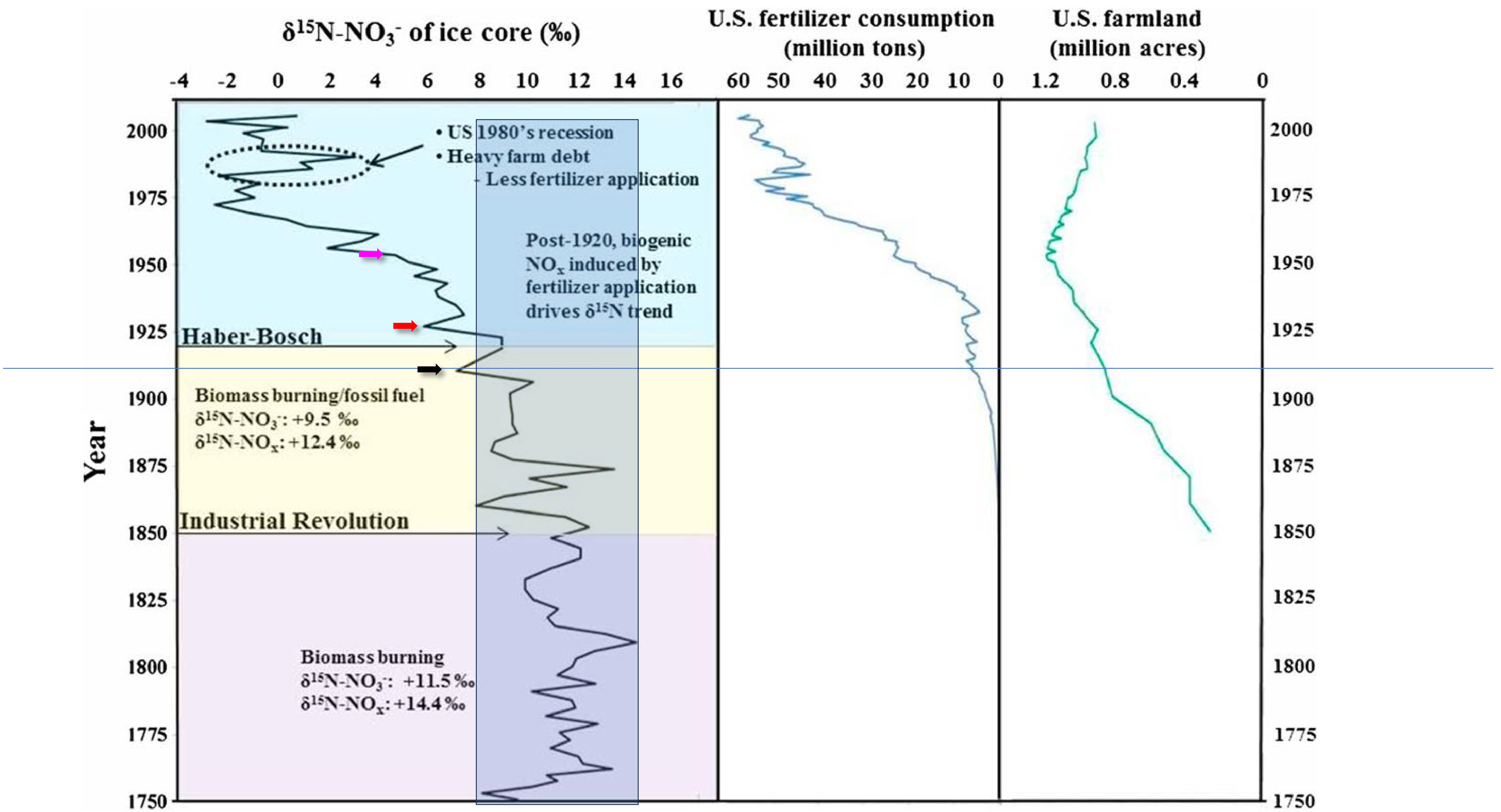

Felix et al., 2013
