# Supplementary material for: Toward defining the Anthropocene onset using a rapid increase in anthropogenic fingerprints in global geological archives
Source: Proc Natl Acad Sci U S A. 2024 Sep 23;121(41):e2313098121. doi: 10.1073/pnas.2313098121 (PMC11474069; doi:10.1073/pnas.2313098121)
Supplement: Supplementary file 4 — Dataset S03 (PDF) [file pnas.2313098121.sd03.pdf]

Criteria for fingerprint detection

(i) First appearance of the signal in a record of anthropogenic novel materials/new species, a lithological markers, as well as disappearance, i.e., a data point just after last appearance of a species

(ii) Beginning of a substantial increase or a substantial decrease in a proxy value

(iii) First appearance of an unprecedented high or low value of a proxy (lower or higher than a range of values before a given age, e.g., 1800 CE)

(iv) Inflection point to the higher rate of change if the record shows a long-term trend

(v) Beginning of a small increase or a small decrease with a jump in the proxy values, if any fluctuations between signals detected in criterion (i) and (ii) or in (iii) and (iv)

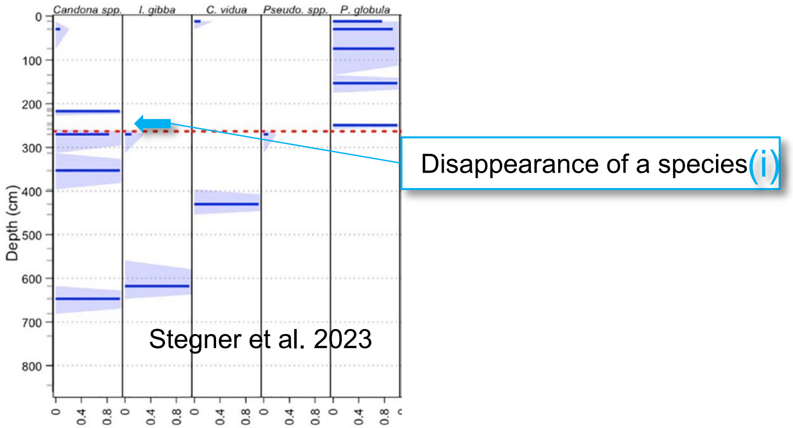

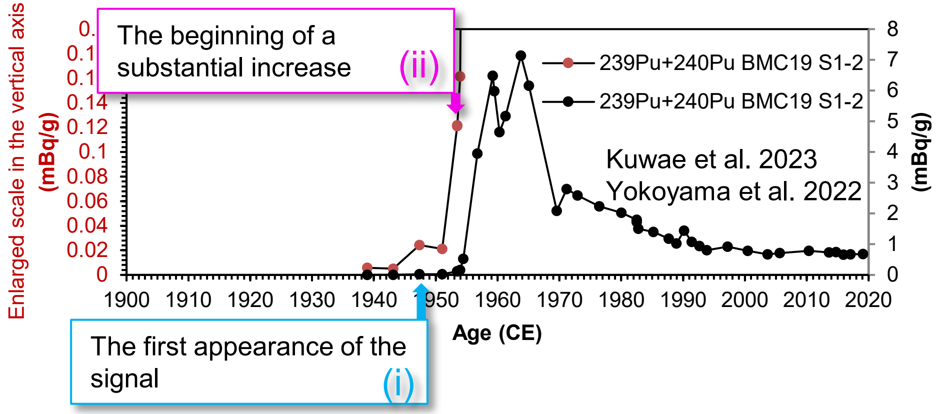

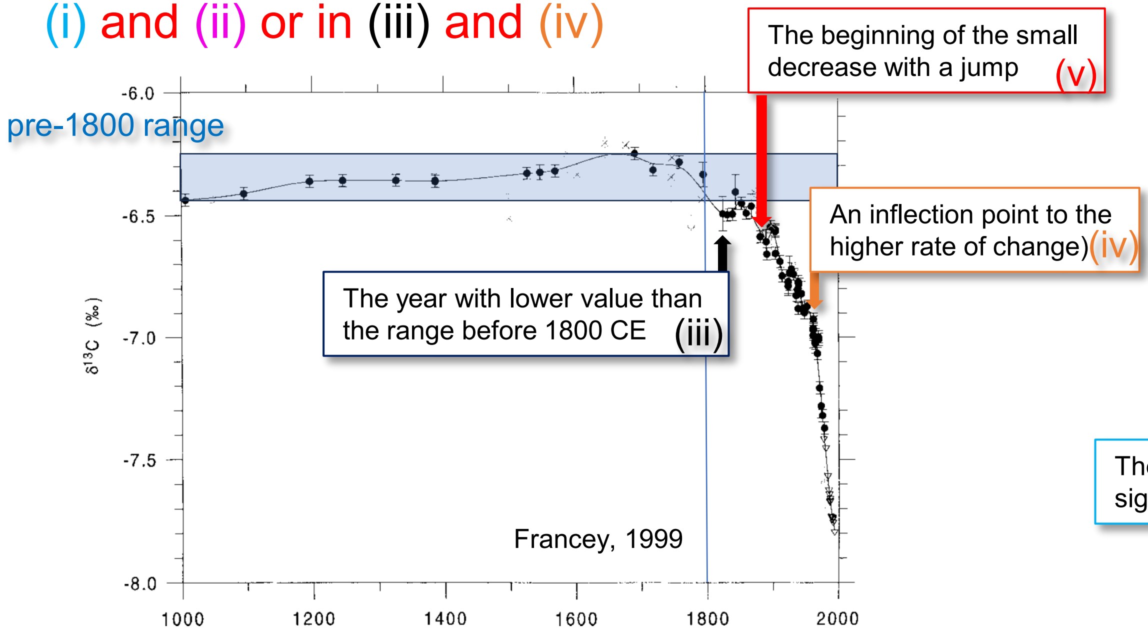

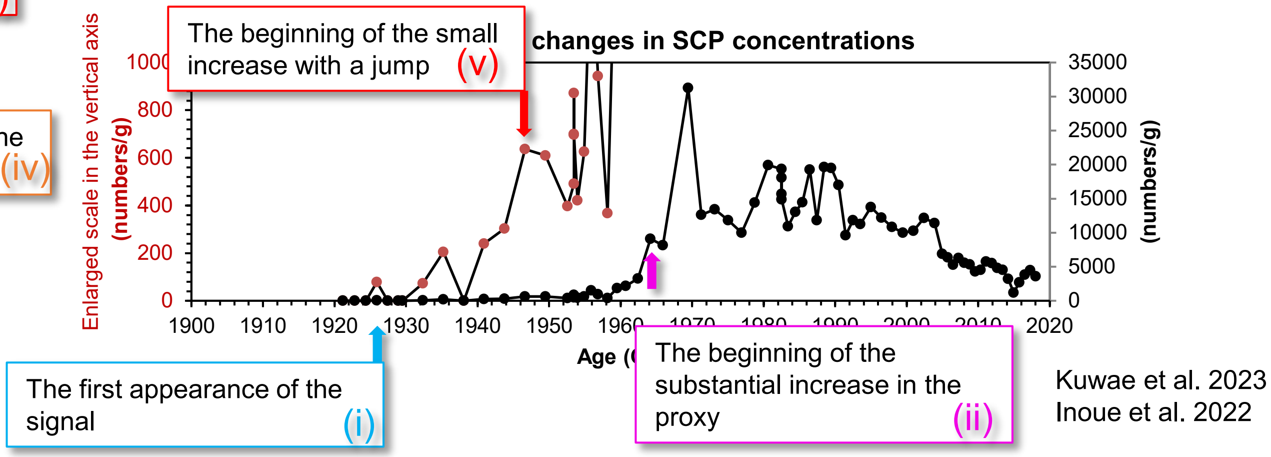

Criteria for fingerprint detection

(vi) Primary major (if, possible, statistically significant) change in microfossil assemblages and chemical compositions

(vii) In some cases, a secondary major change in the assemblages and compositions or the beginning of a secondary substantial increase/decrease (accepted up to a thertiary change)

(viii) If a proxy record shows an unprecedentedly large fluctuation exceeding a given range of background values without an increasing (decreasing) trend, the start of the large fluctuation events should be considered.

(ix) A mass accumulation rate rather than a concentration for an element is adopted to identify a fingerprint

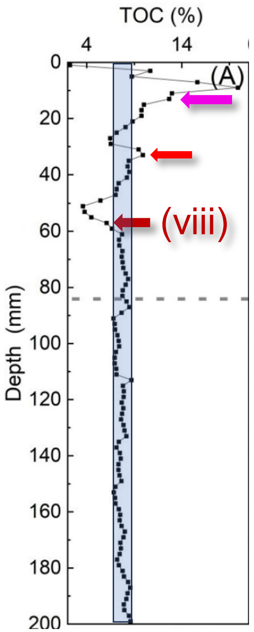

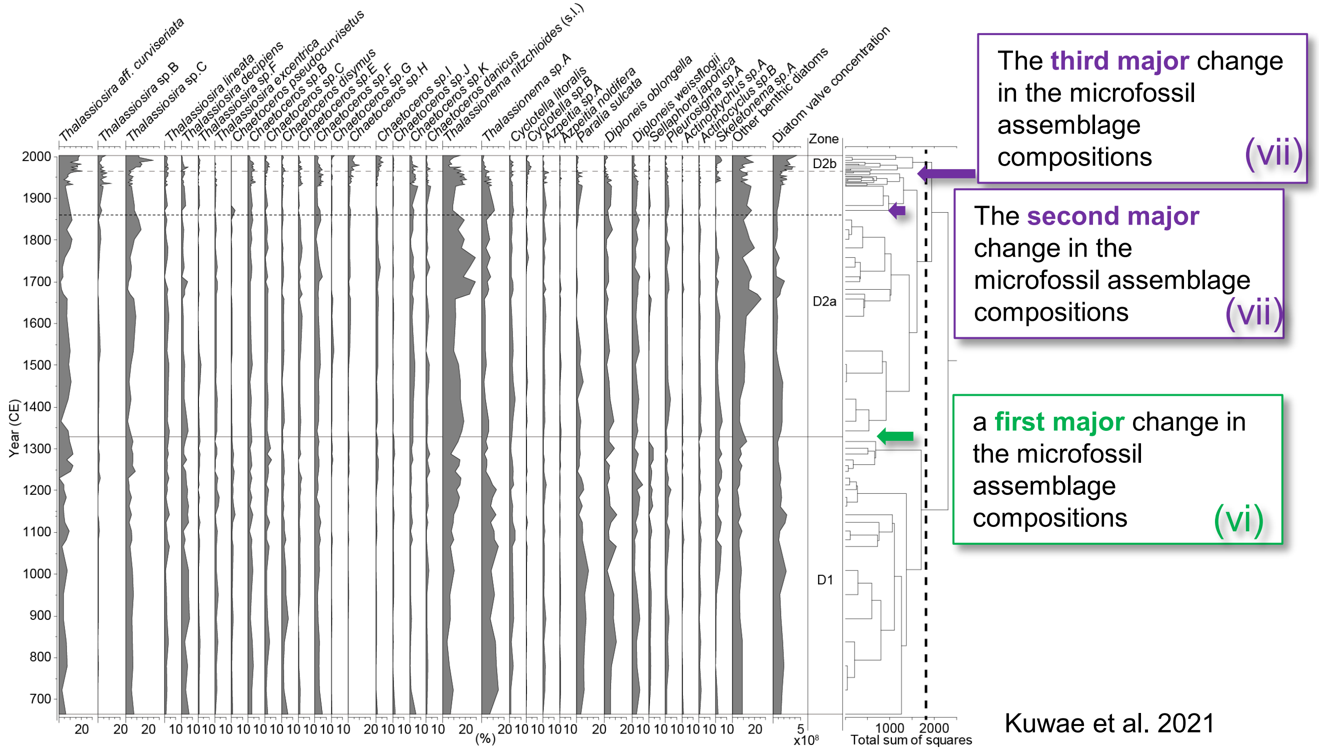

Kuwae et al. 2021

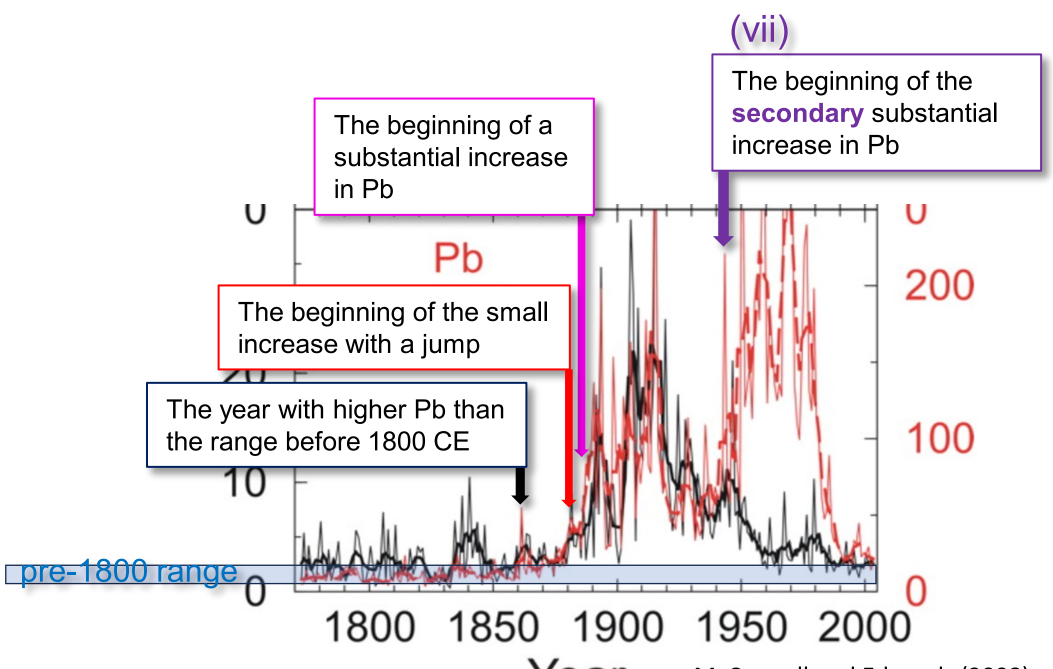

McConnell and Edwards (2008)

Criteria for fingerprint detection

(x) Latest calendar year in an interval for determining the range of background values of a given proxy value is generally assigned to 1800 CE (or 1750 CE for atmospheric CO_2 CH_4 N_2O), because the interval is assumed to be without significant environmental impact from the Industrial Revolution.

(xi) Depending on the length of the record and the existence of an inferred or suspected anthropogenic signal, the last year of the interval for determining the range of background values, 1950, 1940, 1920, 1900, 1850, 1700, 1500, 1400, 1300, 1000, -1000 CE is also permitted.

(xii) For the same record, permitting additional fingerprints detected using the other interval for determining a range of background values (e.g., the pre-1900 range in the right figure), but not permitting double counting.

(xiii) All fingerprints obtained from multiple cores are used even for the same proxy and the same site.

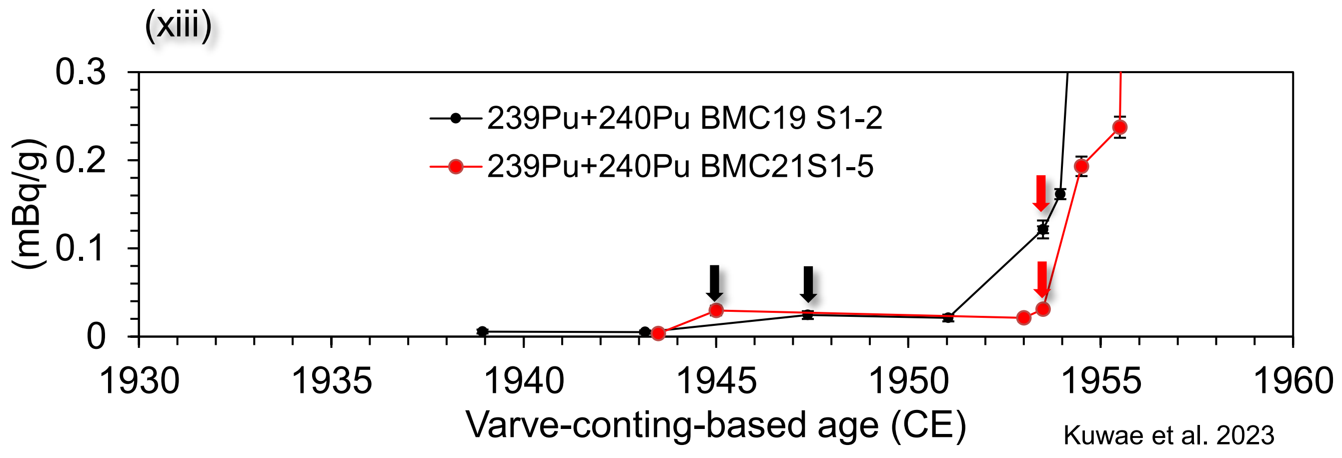

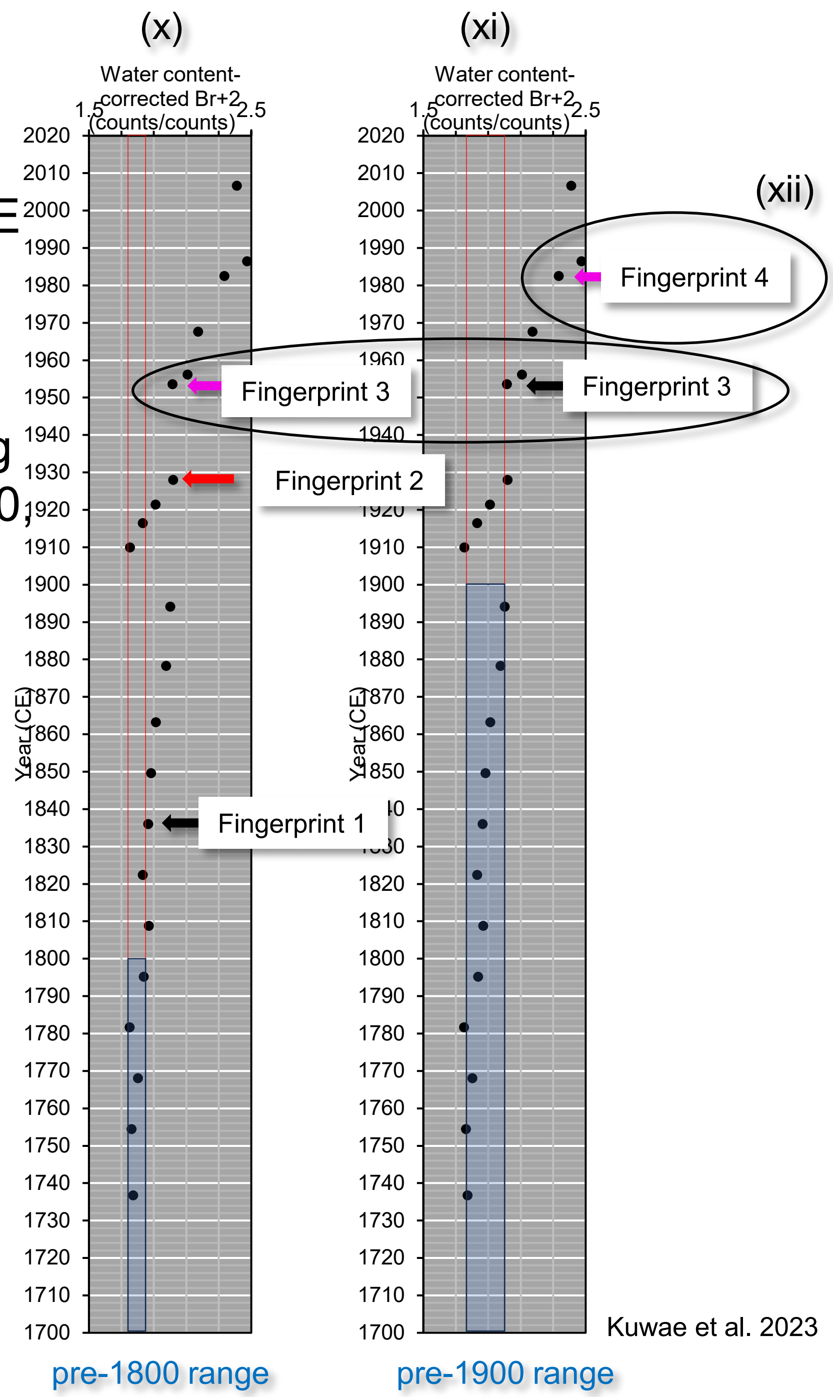

Criteria for fingerprint detection

(xiv) Distinct anthropogenic signals (charcoal: a proxy of usage of fire) were considered even if the signal is detected in the early period of the record

(xv) The beginning of the unprecedented decrease in the value of the number for a species

(xvi) For coral records, ages are principally selected from monthly data; however, in some cases, they are selected from annual data when only annual data are available.

(xiv)

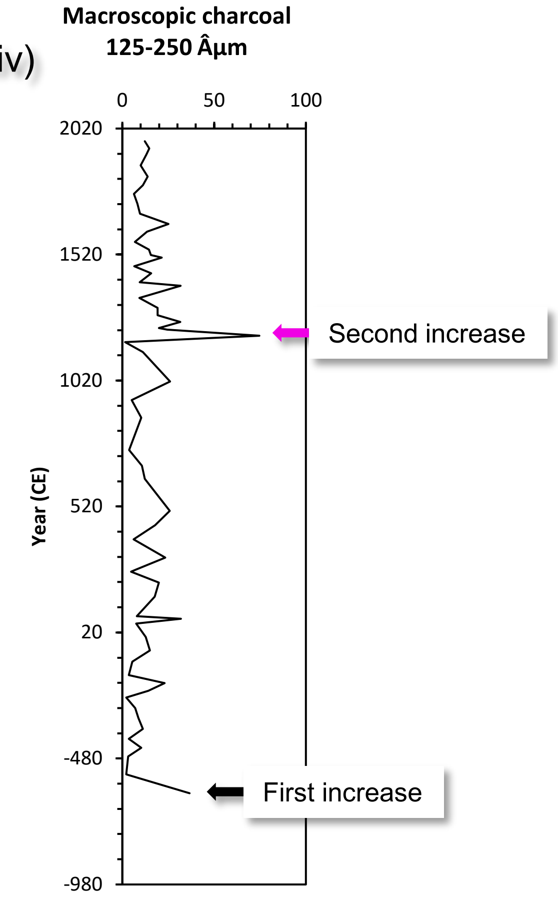

Kuwae et al. 2023

(xiv)

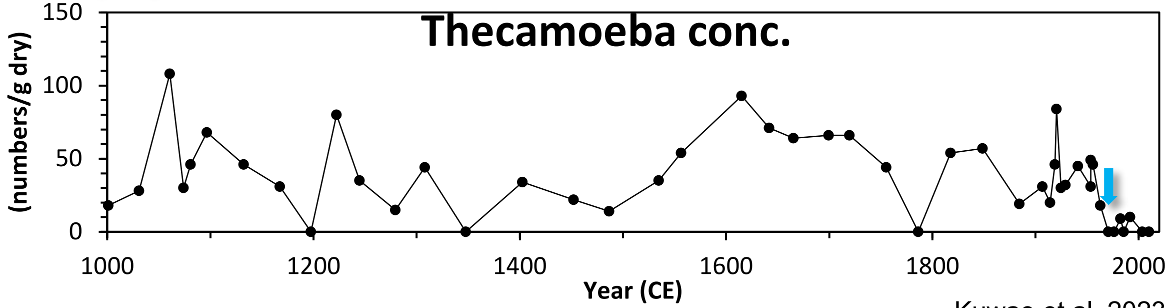

Kuwae et al. 2023
